# Domain-specific physical activity levels and risk of dementia: a systematic review and meta-analysis

**DOI:** 10.1016/S2468-2667(26)00142-8

**Published:** 2026-09

**Authors:** Natan Feter, Paula Iso-Markku, Tiladia Markarian, Dylan M Luong, Jack Gunnink, Natália Schröder, Eduardo Lucia Caputo, Jayne Feter, Anamika Nanda, Sarah Hourihan, Gustavo S da Silva, Ashley Ventura, Daniela Benzano Bumaguin, Yann C Klimentidis, Gene E Alexander, Daniel Umpierre, Pedro C Hallal, Airton José Rombaldi, David A Raichlen

**Affiliations:** Human and Evolutionary Biology Section, Department of Biological Sciences, University of Southern California, Los Angeles, CA, USA (N Feter PhD, T Markarian BSc, D M Luong, J Gunnink, A Nanda BA, S Hourihan BA, A Ventura, Prof D A Raichlen PhD); Center of Research on Physical Activity, Health, and Technology, School of Physical Education, Universidade Federal de Pelotas, Pelotas, Brazil (N Feter, N Schröder MSc, E L Caputo PhD, J Feter PhD, Prof A J Rombaldi PhD); Institute for Molecular Medicine Finland, HiLIFE, University of Helsinki, Helsinki, Finland (P Iso-Markku PhD); Clinical Physiology and Nuclear Medicine, HUS Diagnostic Center, Helsinki University Hospital, Helsinki, Finland (P Iso-Markku); School of Medicine, Universidade Federal do Rio Grande do Sul, Porto Alegre, Brazil (N Feter, N Schröder, J Feter); Center for Evidence Synthesis in Health, School of Public Health, Brown University, Providence, RI, USA (E L Caputo); Instituto de Cardiologia, Fundação Universitária de Cardiologia, Porto Alegre, Brazil (N Feter, Prof D B Bumaguin PhD); Department of Epidemiology and Biostatistics (Prof Y C Klimentidis PhD), Department of Psychology (Prof G E Alexander PhD), and Department of Psychiatry (Prof G E Alexander), University of Arizona, Tucson, AZ, USA; BIO5 Institute, Evelyn F McKnight Brain Institute, Neuroscience Graduate Interdisciplinary Program, and Physiological Sciences Graduate Interdisciplinary Program, University of Arizona, Tucson, AZ, USA (Prof G E Alexander); Arizona Alzheimer’s Consortium, Phoenix, AZ, USA (Prof G E Alexander); Clinical Research Center, Hospital de Clinicas de Porto Alegre, Universidade Federal do Rio Grande do Sul, Porto Alegre, Brazil (Prof D Umpierre PhD); Department of Health and Kinesiology, University of Illinois Urbana-Champaign, Urbana, IL, USA (Prof P C Hallal PhD); School of Physical Education, Universidade Federal de Pelotas, Pelotas, Brazil (G S da Silva MSc, Prof A J Rombaldi); Department of Anthropology, University of Southern California, Los Angeles, CA, USA (Prof D A Raichlen)

## Abstract

**Background:**

Associations between physical activity and distinct health outcomes vary by domain (eg, leisure-time, occupational, household, or commuting); however, comparable evidence on dementia risk remains scarce. We aimed to examine the associations between domain-specific physical activity and risk of all-cause dementia, Alzheimer’s disease, and vascular dementia.

**Methods:**

In this systematic review and meta-analysis, we systematically searched PubMed, CINAHL, Scopus, PsycINFO, SPORTDiscus, and Web of Science for cohort and case–control studies published between Jan 1, 2021, and May 2, 2026. We also included studies from a previous systematic review (from database inception to Oct 21, 2021) with the same search strategy and eligibility criteria. Eligible studies included adults aged 20 years or older with cognitive function assessed at baseline, physical activity measures, and at least 1 year of follow-up. Studies were appraised with the Rayyan web-based platform and summary data (relative risk [RR] and 95% CI) were extracted by up to six independent reviewers. Outcomes were all-cause dementia, Alzheimer’s disease, and vascular dementia, analysed by multilevel random-effects meta-analyses. Study quality was assessed with an adapted scale. This study is registered with PROSPERO (CRD420251010899).

**Findings:**

Among 6270 identified records, 17 studies were eligible for inclusion. Combined with 57 studies in the previous systematic review, we included 74 studies totalling 4 227 297 participants (2 230 046 [52·8%] female and 1 997 251 [47·2%] male; median age 67·3 years [IQR 56·5–74·1]). 40 (54%) studies were of moderate or high quality and 69 (93%) were conducted in high-income countries. Over a median follow-up of 10·0 years (IQR 5·1–21·3), high physical activity levels were associated with reduced risks of all-cause dementia (n=146 effect sizes; RR 0·80 [95% CI 0·75–0·86]; *I*^2^=97·4%), Alzheimer’s disease (n=59; 0·79 [0·70–0·90]; *I*^2^=91·3%), and vascular dementia (n=21; 0·71 [0·58–0·87]; *I*^2^=77·5%); however, associations varied by domain. Leisure-time (*k*=20 studies; n=33 effect sizes; RR 0·76 [95% CI 0·65–0·88]; *I*^2^=96·6%) and household (*k=*1; 0·85 [0·74–0·98]) physical activity were associated with a reduced risk of all-cause dementia. By contrast, occupational (*k*=5; 1·20 [1·01–1·42]; *I*^2^=85·6%) and commuting (*k*=2; 1·10 [1·03–1·18]; *I*^2^=0·0%) physical activity were associated with an increased risk. Dose–response meta-analyses showed non-linear inverse associations for leisure-time and occupational activity. Certainty of evidence ranged from very low to low across domains.

**Interpretation:**

The association between physical activity and dementia risk might be domain-specific. Public health strategies for the prevention of dementia should address equitable access to safe spaces for exercise, adequate leisure time, and mitigation strategies in occupational settings.

## Introduction

Dementia is a leading cause of disability, with 14 modifiable risk factors, including physical inactivity, accounting for 45% of cases.^1–4^ Although physical activity occurs across four domains (leisure-time, occupational, household, and commuting), research primarily focuses on leisure-time physical activity.^4,5^ This emphasis overlooks global behavioural patterns: leisure-time activity represents only 12% of total physical activity worldwide, whereas occupational and household activities (52%) and commuting (36%) predominate.^6^ These disparities are socially patterned, with leisure-time physical activity comprising only 4% of total physical activity in low-income countries compared with 28% in high-income countries (HICs).^6^

Emerging evidence suggests that the association between physical activity and cognitive outcomes varies by the context in which the activity occurs. For instance, leisure-time physical activity is linked with slowed cognitive decline^5^ and reduced risk of dementia,^7^ whereas the association with occupational, household, and commuting activities remains unclear or potentially detrimental.^5,8,9^ Although previous systematic reviews and meta-analyses have suggested a domain-specific association between physical activity and different health outcomes, such as mental health, cardiovascular incidence, and cardiovascular and all-cause mortality,^10–14^ domain-specific association between physical activity and dementia risk remains poorly understood.

Furthermore, although the global distribution of dementia is highly uneven, projections indicate that existing geographical disparities will widen substantially in the coming decades.^1^ A comprehensive synthesis of the existing literature is necessary to clarify the role of domain-specific physical activity in dementia prevention worldwide. We aimed to examine the associations between domain-specific physical activity and risk of all-cause dementia, Alzheimer’s disease, and vascular dementia. Additionally, we visually compared the geographical distribution of studies examining the association between physical activity and dementia and the projected worldwide burden of dementia.

## Methods

### Search strategy and selection criteria

We conducted a systematic review and meta-analysis in line with the PRISMA guidelines.^15^ Minor deviations from the original registered plan included restricting inclusion to peer-reviewed articles, adjusting the database search strategy, and replacing the risk-of-bias tool and publication bias assessment methods. All changes are fully detailed in the appendix (pp 2–4). This study is registered with PROSPERO (CRD420251010899).

We report the search strategy in line with the PRISMA-S guidelines for search updates in systematic reviews.^16^ A previous systematic review and meta-analysis by Iso-Markku and colleagues^4^ synthesised evidence on the association between physical activity and the risk of dementia from database inception to Oct 21, 2021.^4^ In the meta-analysis, the authors identified only two studies^17,18^ that examined the relationship between physical activity and risk of dementia using neither total nor leisure-time physical activity. We replicated the search strategy, including the eligibility criteria, from Iso-Markku and colleagues^4^ and included all studies from the previous systematic review as the foundation of the present synthesis. We then conducted an updated independent search from Jan 1, 2021, to May 2, 2026, to identify additional studies, enabling a domain-specific meta-analysis. The full search strategy is available in the appendix (pp 5–6).

We systematically searched, without language restrictions, the six following electronic databases: PubMed, CINAHL, Scopus, PsycINFO, SPORTDiscus, and Web of Science. We filtered the present systematic search to include full-text articles published between Jan 1, 2021, and May 2, 2026. We included cohort and case–control studies that measured baseline physical activity and assessed dementia risk, with a follow-up of at least 1 year. We excluded experimental studies to fully comply with the eligibility criteria of the previous systematic review.^4^ Considering that Iso-Markku and colleagues^4^ performed their final search on Oct 21, 2021, we anticipated some overlap in the included studies; however, we determined that this approach would reduce the risk of missing potentially eligible studies if we had filtered to a specific date. Duplicate studies, if any, were excluded.

We included studies that assessed all-cause dementia, Alzheimer’s disease, or vascular dementia, identified with validated cognitive assessments or registry data. We excluded studies relying on self-reported diagnoses or cause-of-death data for more than 50% of the participants due to the low sensitivity of death registers to detect dementia cases.^19^

Eligible studies assessed physical activity using objective measures or self-reported questionnaires and included a reference group based on physical activity levels (eg, inactive *vs* active, low *vs* high activity, or continuous). Experimental studies assessing either the acute or chronic effect of structured exercise sessions or measuring fitness rather than physical activity level were excluded. For occupational physical activity, we prioritised summary estimates restricted to working participants over analyses that combined non-working or retired individuals in the low occupational physical activity group to minimise healthy worker bias.

The study population consisted of adults aged 20 years or older at baseline. We excluded studies that restricted enrollment to individuals with a diagnosed disease, as well as cohorts in which dementia or mild cognitive impairment was present at baseline. For older adults (mean or median age >55 years, upper age limit ≥65 years, or mean age plus SD ≥60 years), a valid baseline cognitive assessment was required to reduce the likelihood of including participants in a prodromal phase of dementia and to reflect the long preclinical course of Alzheimer’s disease.^20^

Study inclusion was based on the assessments of six independent reviewers (TM, JF, DML, JG, NS, and GSdS) using the Rayyan web-based platform. Each study was screened by two reviewers. Disagreements were discussed, and if consensus was not reached, an independent researcher (NF) made the final decision. Screening was conducted in two phases. Studies were first excluded on the basis of title and abstract screening, before full-text manuscripts were reviewed. When multiple studies used data from the same cohort, we included data from the study with the highest quality score, the longest follow-up, or the largest study population (in this order).^4,5^ A justification for excluding studies with overlapping data is provided in the appendix (pp 140).

### Statistical analysis

The following data on outcomes, exposures, and moderators were extracted from the included studies: outcome ascertainment (Diagnostic and Statistical Manual of Mental Disorders [DSM]-III-R, DSM-IV, or DSM-5; clinical evaluation; health records; or cognitive tests only); physical activity levels (for each contrast, when reported categorically); estimates of the associations between physical activity level and all-cause dementia, Alzheimer’s disease, or vascular dementia (ie, relative risk [RR], hazard ratio [HR], or odds ratio [OR], with respective 95% CIs); physical activity domain, if reported (non-specific, leisure-time, occupational, household, or commuting); length of follow-up (<5, 5–10, 11–20, or >20 years); age (30–49, 50–64, 65–80, or >80 years) and sex (male or female) of study population; sample size; country of origin; publication year; study design (cohort *vs* case–control); presence of confounders (age, cognition at baseline, presence of at least one chronic disease, education, sex, vascular risk factors, or *APOE*ε*4* status); study quality (low, moderate, or high); and physical activity criteria, based on WHO guidelines^21^ of at least 150–300 min of moderate intensity aerobic physical activity, at least 75–150 min of vigorous intensity aerobic physical activity, or an equivalent combination of moderate and vigorous intensity activity throughout the week.

Four reviewers (TM, JF, DML, and JG) independently performed data extraction, with disagreements resolved through discussion. The estimates with the most extensive adjustments were included.^21^ Study-specific considerations for data extraction are available in the appendix (p 11). Estimates were reported as RRs with respective 95% CIs. For studies that did not report data on RR, ORs or HRs were converted into RRs as previously described^4,22–24^ and reported in the appendix (p 12). A separate RR was calculated for each physical activity category above the reference level by comparing it with the reference category reported in the included studies.

We applied the quality assessment tool originally developed by Iso-Markku and colleagues^4^ (appendix pp 7–10). This instrument evaluates study design, cohort representativeness, method of physical activity assessment, confirmation that participants did not have dementia at baseline, approaches to confounder adjustment, procedures for outcome ascertainment, duration of follow-up, and attrition rates. Each topic was rated as 0·0 stars, 0·5 stars, and 1·0 star. Six reviewers (TM, ELC DML, JG, NS, GSdS) independently performed the assessments, with disagreements resolved through discussion. When methodological details were referenced from other publications, we consulted up to three related articles to obtain the necessary information. The overall quality score was categorised into three levels: high (selection ≥2·5 stars, comparability 1·0 star, and outcome ≥2·5 stars); moderate (selection ≥2·0 stars, comparability ≥0·5 stars, and outcome ≥2·0 stars); and low (selection <2·0 stars, comparability <0·5 stars, and outcome <2·0 stars). Certainty of evidence was independently assessed by two reviewers (NF and GSdS) by use of the GRADE framework, with discordances resolved through discussion. GRADE evaluates evidence across five domains (risk of bias, inconsistency, indirectness, imprecision, and publication bias) to classify the certainty of evidence as high, moderate, low, or very low.

We conducted a multilevel random-effects meta-analysis to address dependence among effect sizes and to prevent overestimation of effects. Random intercepts were specified at the study level and within-study level, and parameters were estimated through restricted maximum likelihood. Summary statistics were RRs, with 95% CIs and p values for overall and moderator effects calculated with Wald-type tests based on the standard normal (Z) distribution. We modelled the dependency at the study sample level to account for results from multiple comparisons across multiple physical activity categories against the same reference category. Heterogeneity was evaluated with the Cochrane Q test and quantified with the *I*^2^ statistic (negligible [0–40%], moderate [30–60%], substantial [50–90%], and considerable [75–100%] heterogeneity).^25^ All meta-analyses were performed with the dosresmeta^26^ and metafor^27^ packages in R (version 4.5.1) within RStudio (version 2025.05.0).

We performed dose–response meta-analyses to explore linear, quadratic, and restricted cubic spline trends between physical activity levels in metabolic equivalent of energy expenditure (MET) min per week and RRs of dementia onset.^4^ Physical activity exposure was harmonised across studies by assigning a quantitative value to each reported exposure category. For categorical measures of physical activity, we used the central tendency of each category (mean or median when available, or the midpoint of the reported range) to represent exposure intensity. When the original studies provided activity-specific MET values, these were retained. Otherwise, standardised MET values were applied: 3·5 METs for walking, 4·5 METs for moderate intensity activity, and 8·0 METs for vigorous or sports-related activity.^28^ To limit the influence of implausibly high exposure levels, physical activity was capped at 21 h of moderate intensity activity per week, corresponding to 5040 MET min per week. A reference value of 200 MET min per week was selected for all dose–response meta-analyses, reflecting the approximate mean exposure level of reference categories across the included studies.^4^ A full description of the dose–response methods and harmonisation procedures is available in the appendix (p 13) and elsewhere.^4^

To explore sources of heterogeneity, we assessed potential moderating effects of age, sex, duration of follow-up, study quality, meeting physical activity guidelines, and dementia criteria, given that previous studies have suggested that these factors might modify the association between physical activity and dementia risk.^4,29^ Moderation analyses were performed only if at least five effect sizes were available within a category.^30^

Small-study effects were assessed visually with funnel plots. Because Egger’s test is not available for multilevel models,^31^ we applied a standardised regression of log RRs on the inverse SE. When asymmetry was detected, precision-effect test and precision-effect estimate with SEs (PET-PEESE) analyses were used to evaluate robustness to small-study bias.^32^

We also performed a series of sensitivity analyses. First, we used the leave-one-out approach, excluding one study at a time, to ensure that the results were not simply due to a single large study or an extreme result. Second, we excluded studies rated as low quality. Third, we assessed whether the association between physical activity and dementia risk differed according to study adjustment for baseline age, education, presence of at least one chronic disease, or *APOE*ε*4* status. Finally, we examined whether the domain-specific associations between physical activity and the risk of all-cause and cause-specific dementia differed according to the covariates included in the adjusted models across studies.

To visually illustrate the mismatch between research output and projected dementia burden, we constructed distorted world maps using the Cartogram R package.^33^ On these maps, country size was rescaled according to two metrics: (1) the number of studies on physical activity and dementia identified in the present meta-analysis; and (2) the projected relative increase in dementia cases between 2019 and 2050 based on estimates from the Global Burden of Diseases, Injuries, and Risk Factors 2019 study.^1^ Countries were additionally grouped by income level by use of World Bank classifications to contextualise disparities across income groups.^34^

### Role of the funding source

The funders of the study had no role in study design, data collection, data analysis, data interpretation, or writing of the report.

## Results

**Figure 1:**
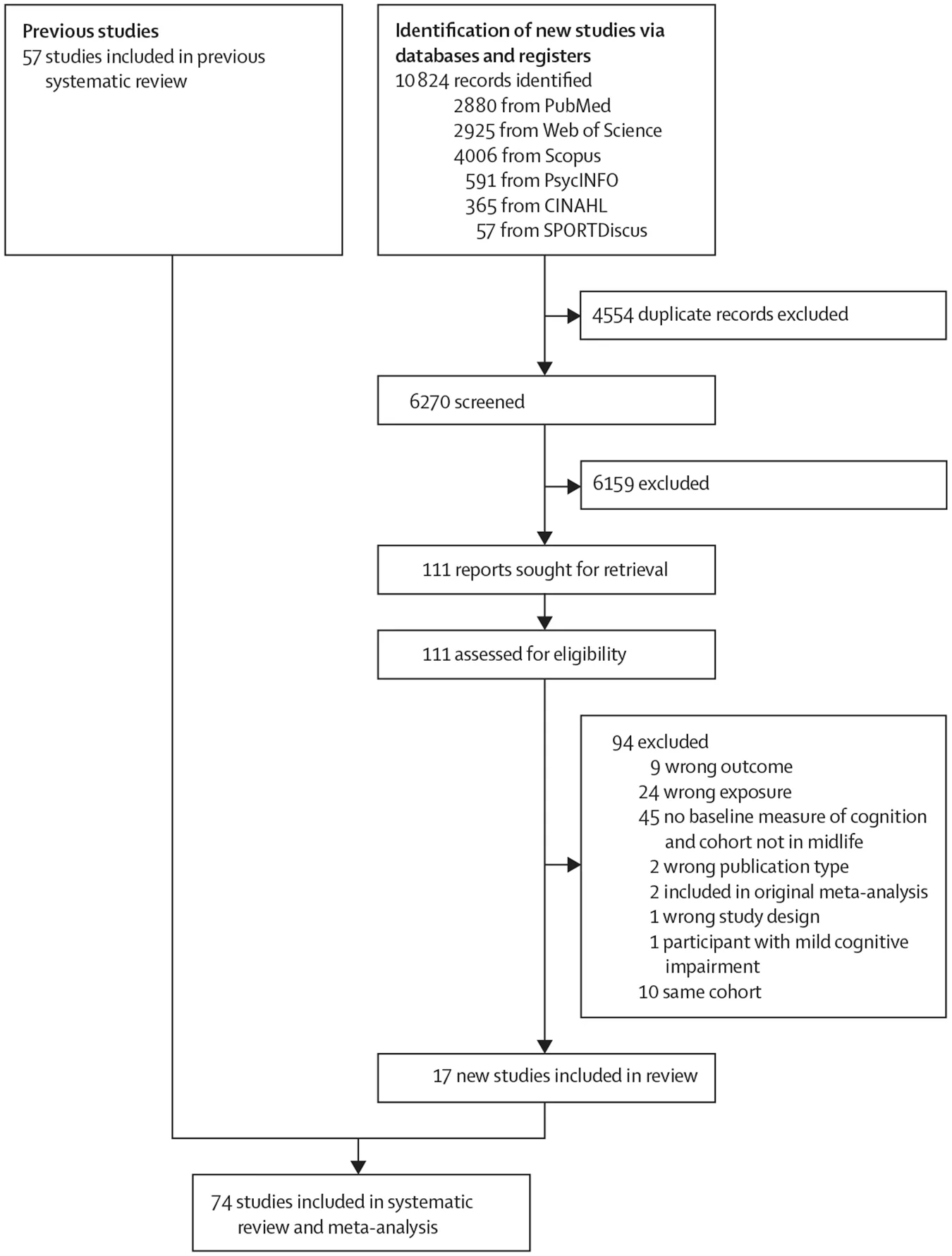
Study selection One study^52^ was excluded because it contained duplicate data and had a shorter follow-up than a study included in the most recent search.^51^

A total of 6270 studies were retrieved and screened for titles and abstracts after removing duplicates (figure 1). After full-text screening, 17 studies were eligible for inclusion.^35–51^ Records screened during title, abstract, and full-text screening are listed in the appendix (pp 14–139). We excluded one study^52^ from the previous systematic review because it contained duplicate data and had a shorter follow-up than a study included in the most recent search.^51^

Combining the remaining 57 studies from the previous systematic review, we included 74 studies in the current systematic review and meta-analysis. The total sample size was 4 227 297 participants, of whom 2 230 046 (52·8%) were female and 1 997 251 (47·2%) were male, with a median age of 67·3 years (IQR 56·5–74·1) at baseline. Most studies were conducted in the USA (*k*=24; three studies included in the study by Li and colleagues,^39,49,51,53–71^ followed by Finland (*k*=8; three studies included in the meta-analysis by Kivimäki and colleagues^7^),^18,72–76^ Sweden (*k*=7),^46,47,72,77–80^ and the UK (*k*=6).^29,42,48,81–83^ Included studies covered leisure-time, occupational, commuting, and household physical activity domains, as well as total or unspecified activity (table). A full description of each included study is provided in the appendix (pp 156–164). Among the 74 studies included in the analysis, 40 (54%) received a moderate or high overall quality score,^17,18,29,36,37,40–42,44–49,51,54,55,58,59,62,64,66,71–74,76,77,79,82,84–93^ whereas the remaining 35 (47%) received low quality scores.^35,38,39,43,50,53,57,60,61,63,65,67,69,70,72,75,78,80,81,83,94–108^ Kivimäki and colleagues^72^ contributed four distinct cohorts with varying quality: one low, two moderate, and one high, and were therefore counted in both categories. For the purpose of the summary percentage, the meta-analysis by Kivimäki and colleagues^74^ is accounted for in both categories based on its constituent cohorts. Most studies received 1·0 star for outcome assessment and baseline cognition, and 0·0 or 0·5 stars for selection and follow-up rate (appendix (pp 141–142).

Over a median follow-up of 10·0 years (IQR 5·1–21·3), there were 64 009 cases of all-cause dementia (median incidence 8·4% [IQR 5·4–14·8%]; 44 083 910 person-years), 5859 cases of Alzheimer’s disease (5·4% [3·7–8·0]; 1 300 946 person-years), and 1607 cases of vascular dementia (3·2% [1·5–6·1]; 6 573 530 person-years). Dementia ascertainment varied across studies: 30 relied on DSM-III, DSM-IV, or DSM-5 criteria;^18,46,51,53,54,57,59,60,62,64,66,67,70,78,80,83–87,90,91,95,96,100,103,105,107,108 17^ on clinical evaluations combining cognitive function and functional capacity;^55,58,61–63,71,73,75,77,82,89,92–94,99,101,106^ 20 on health records;^17,29,35–37,41–44,51,72,74,76,79,98,104,109^ six on cognitive tests alone;^38,39,49,69,97,102^ and six on other methods, including proxy interview, self-reported plus proxy interview, and mixed approaches (ie, health records and cognitive tests; appendix pp 156–164).^40,45,47,48,50,81^ One study relied on self-reported dementia diagnoses alone, but was included because all participants were further assessed through proxy interviews to confirm dementia status with the Informant Questionnaire on Cognitive Decline in the Elderly.^48^ Li and colleagues^51^ analysed data from two independent cohorts that identified dementia cases through DSM-IV criteria (ie, the ARIC study and the Framingham Heart study) and one cohort through health records (ie, the MESA study). All four individual studies included in the meta-analysis by Kivimäki and colleagues^72^ identified dementia cases by use of health records.

Compared with the least active group, high categories of physical activity were associated with a reduced risk of all-cause dementia (n=146 effect sizes; RR 0·80 [95% CI 0·75–0·86]; *I*^2^=97·4%; τ^2^_Level 3_=0·07; between-cluster heterogeneity: *I*^2^_Level 3_=75·5%; τ^2^_Level 2_=0·02; within-cluster heterogeneity: *I*^2^_Level 2_=21·9%; appendix p 143). A three-level model provided a significantly better fit than a two-level model (χ^2^=34·0; p<0·0001). The funnel plot and Egger regression plot (β_0_ −0·25; p=0·34) showed no evidence of publication bias (appendix pp 144–145); however, the PET-PEESE analysis indicated a significant slope (β −0·57; p=0·022). Nevertheless, bias-adjusted estimates remained directionally consistent with the primary findings (RR 0·83 [95% CI 0·78–0·88]).

**Figure 2:**
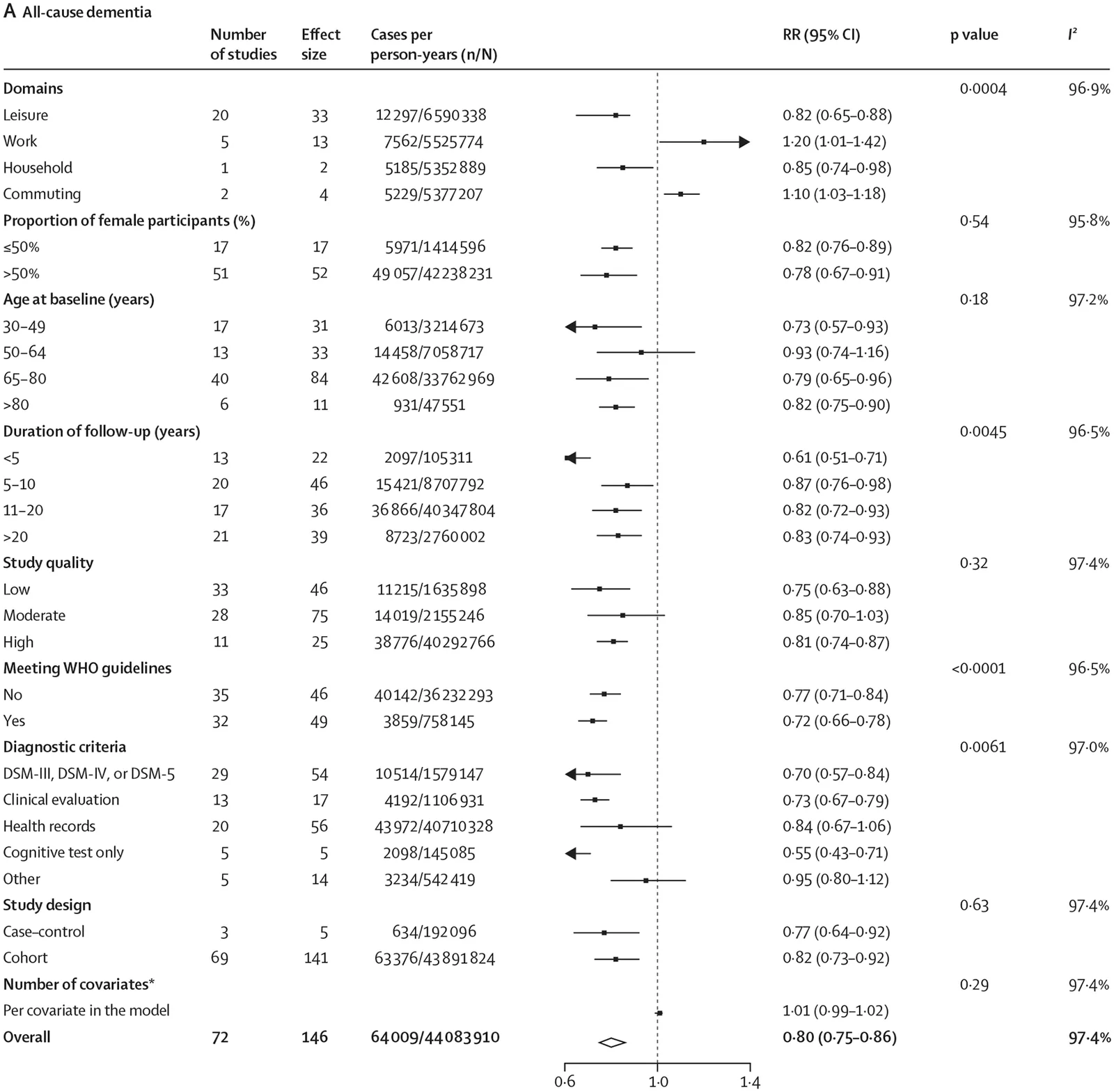

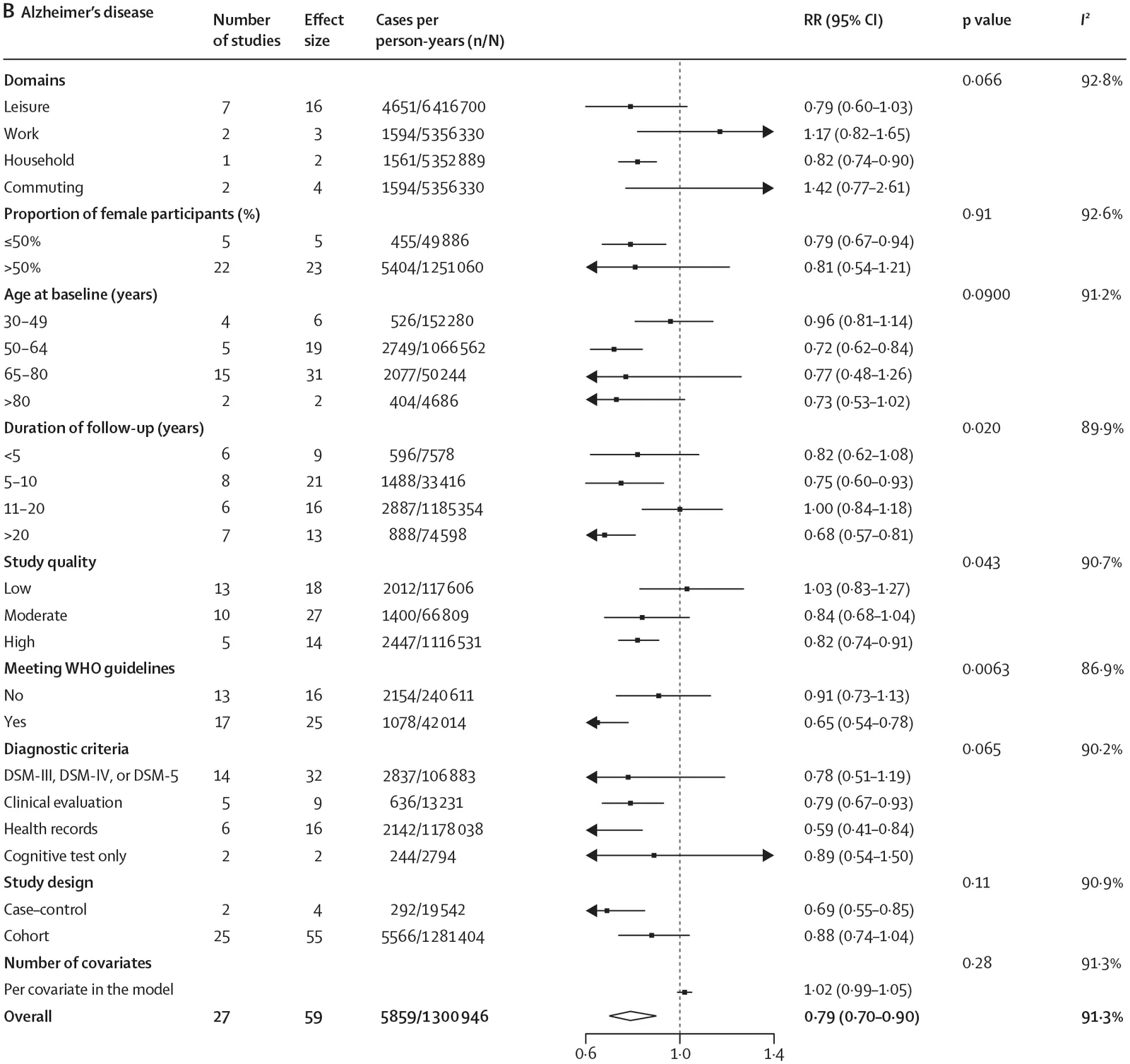

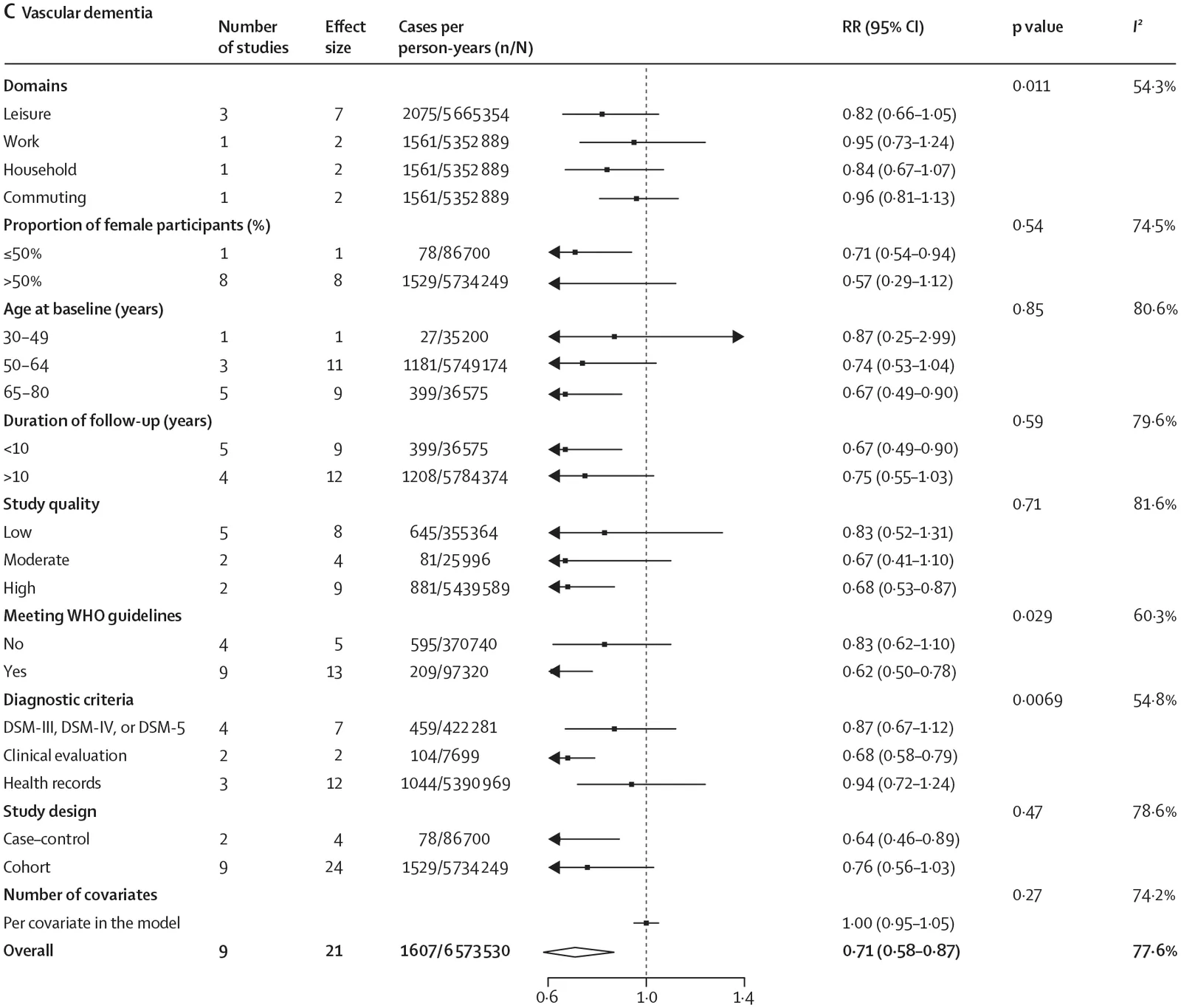
Moderation analyses for the association between physical activity and risk of dementia Associations shown for all-cause dementia (A), Alzheimer’s disease (B), and vascular dementia (C). RR=relative risk. DSM=Diagnostic and Statistical Manual of Mental Disorders. *Excluded in the most recent format.

Physical activity domain significantly moderated the correlation between physical activity and all-cause dementia risk (p=0·0004 for moderating effect; figure 2). Leisure-time (*k*=20; n=33 effect sizes; RR 0·76 [95% CI 0·65–0·88]; *I*^2^=96·6%) and household (*k=*1; n=2; 0·85 [0·74–0·98]) physical activity were associated with a reduced risk of dementia. By contrast, occupational (*k*=5; n=13; 1·20 [1·01–1·42]; *I*^2^=85·6%) and commuting (*k*=2; n=4, 1·10 [1·03–1·18]; *I*^2^=0·0%) physical activity were associated with an increased risk. Of note, findings for household and commuting physical activity were limited to a total of three studies.^18,42,86^ In studies that categorised physical activity on the basis of WHO recommendations, the pooled RR for dementia was lower for those meeting the recommendation compared with those that did not (p<0·0001 for moderating effect). Diagnostic criteria also emerged as a significant moderator; studies that used cognitive screening tests showed a stronger protective association than those that used clinical DSM criteria (p=0·0061 for moderating effect). Follow-up duration also acted as a moderator, with studies reporting a larger protective effect with shorter follow-up (<5 years) compared with longer follow-up (≥5 years). No other moderation effect was observed.

**Figure 3:**
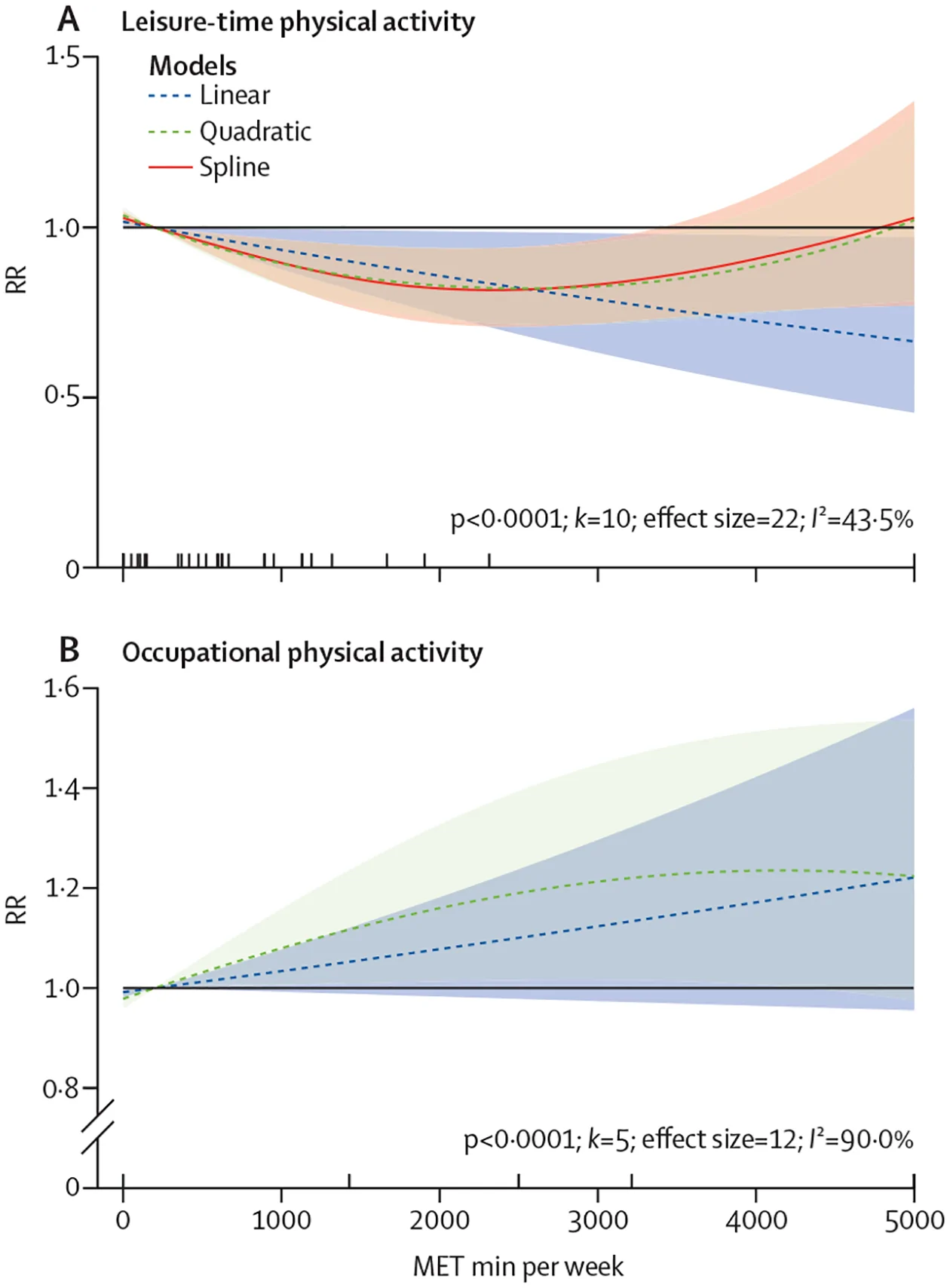
Dose–response meta-analysis of domain-specific physical activity levels and risk of all-cause dementia Dashed lines along the x-axis represent the distribution of the exposure variable, with each mark corresponding to an observed value. The number of studies (*k*) included in the dose–response meta-analysis differs from that included in the systematic review due to an absence of sufficient dose–response data in 39 of the included studies. Further details on the methods used for the dose–response meta-analyses are provided in the appendix (p 13). MET=metabolic equivalent of energy expenditure. RR=relative risk.

In the domain-specific dose–response meta-analyses, leisure-time physical activity showed a non-linear inverse association with all-cause dementia (p<0·0001 for nonlinearity), beyond which the association plateaued (figure 3). For occupational physical activity, limited exposure categories precluded spline modelling; however, a non-linear positive association was observed (p<0·0001), with risk increasing from 200 MET min per week to approximately 3000 MET min per week. Dose–response analyses for household and commuting activity were not undertaken because of an insufficient number of studies (*k*=3).

High physical activity levels were associated with a lower risk of Alzheimer’s disease (n=59 effect sizes; RR 0·79 [95% CI 0·70–0·90]; *I*^2^=91·3%; appendix p 146) than the category of lowest physical activity (τ^2^_Level 3_=0·08; between-cluster heterogeneity: *I*^2^_Level3_=66·1%; τ^2^_Level 2_=0·03; within-cluster heterogeneity: *I*^2^_Level 2_ =25·2%). The funnel plot and Egger regression plot (β_0_ −1·23; p=0·0027) showed small-study effects (appendix pp 147–148). In PET-PEESE analyses, bias-adjusted estimates remained consistent with the primary three-level random-effects analysis (RR 0·80 [95% CI 0·70–0·90]).

High physical activity levels were also associated with a reduced risk of vascular dementia (n=21 effect sizes; RR 0·71 [95% CI 0·58–0·87]; *I*^2^=77·5%; appendix p 149). The estimated variance components were τ^2^_Level 3_ =0·06 and τ^2^_Level 2_=0·01, corresponding to *I*^2^_Level 3_=63·4% of total variation attributed to between-cluster heterogeneity and *I*^2^_Level 2_=14·15% to within-cluster heterogeneity. The funnel plot and Egger regression plot (β_0_ −0·68; p=0·49) showed no evidence of publication bias or small-study effects (appendix pp 150–151). These findings were confirmed as robust in the PET-PEESE analysis (RR 0·80 [95% CI 0·68–0·93]).

For all-cause dementia, meeting WHO physical activity recommendations was associated with reduced risks of Alzheimer’s disease (RR 0·65 [95% CI 0·54–0·78]; p=0·0063 for moderating effect) and vascular dementia (0·62 [0·50–0·78]; p=0·0069). For Alzheimer’s disease, duration of study follow-up was also a moderator, with follow-up exceeding 20 years showing the lowest risk (0·68 [0·57–0·81]). For vascular dementia, the association was moderated by physical activity domain and diagnostic criteria, with the lowest risk observed with leisure-time activity (0·82 [0·66–1·05]) and clinical evaluations (0·68 [0·58–0·79]) relative to other domains and criteria. No other moderating effects were observed.

Across studies examining the correlation between physical activity and risk of Alzheimer’s disease and vascular dementia (*k*=27),^18,42,54,57–59,61,62,66,67,71–73,75–79,84,86,88,91,92,106–108^ seven assessed leisure-time physical activity^42,61,73,76–78,107^ and three assessed three other domains of physical activity: occupational,^18,42^ commuting,^42,86^ and household.^42,86^ Zhu and colleagues^42^ observed a non-significant trend towards an increased risk of Alzheimer’s disease associated with commuting physical activity, whereas household physical activity was associated with a reduced risk. For vascular dementia, household physical activity was associated with a reduced risk of Alzheimer’s disease, whereas commuting physical activity showed no significant association.^42^ Rovio and colleagues^18^ reported no association between commuting physical activity and risk of Alzheimer’s disease.

The sensitivity analyses yielded consistent findings. The moderating effect of physical activity domain remained in the leave-one-out analyses (appendix pp 152–153). Leisure-time physical activity consistently showed a protective association, whereas occupational physical activity remained directionally associated with increased risk (RR >1·07), and the association was attenuated to the null (appendix p 154). There was no evidence that the observed domain-specific associations were driven by individual studies.

For all-cause dementia, findings were consistent across studies using distinct covariates, although pooled estimates were attenuated in studies adjusting for baseline age, education, and *APOE*ε*4* status (appendix p 165). For Alzheimer’s disease, pooled estimates were attenuated in studies in which estimates were adjusted for education and strengthened when adjusting for presence of at least one chronic disease (appendix p 165). For vascular dementia, pooled estimates were strengthened when adjusted for education (appendix p 165).

Domain-specific findings were consistent across covariate-adjusted models (appendix p 166). In analyses adjusting for baseline age, education, or presence of at least one chronic disease, leisure-time physical activity remained inversely associated with all-cause dementia. By contrast, occupational physical activity was consistently associated with an increased risk of all-cause dementia in analyses adjusting for age and education, with reduced precision in models adjusting for presence of at least one chronic disease. Analyses adjusting for *APOE*ε*4* status were limited by the small number of contributing studies; however, point estimates remained directionally consistent with the main findings, albeit with wider 95% CIs. Despite reduced precision, adjusting for age, education, and presence of at least one chronic disease yielded estimates consistent with the main findings for leisure-time physical activity. Analyses of the associations between occupational physical activity for Alzheimer’s disease and vascular dementia were limited due to few contributing studies. The removal of low-quality studies produced similar estimates, consistently showing reduced risk with leisure-time activity and increased risk with occupational activity (appendix p 167). The increased risk associated with occupational physical activity remained significant after adjusting for total activity volume (appendix p 168). Certainty of evidence ranged from very low to low across domains, primarily due to high statistical heterogeneity and a low number of studies investigating specific dementia subtypes (appendix p 169).

Our findings revealed that 69 (93%) of all 74 studies included in the meta-analysis were conducted in HICs. The only low-income and middle-income countries (LMICs) with data available were Brazil, China, Cuba, and Nigeria (appendix p 155).

## Discussion

In this meta-analysis, including data from more than 4 million adults across 30 countries, the association between physical activity and risk of all-cause dementia was domain-specific. Leisure-time physical activity was associated with a reduced risk of dementia, whereas occupational physical activity was linked with an increased risk of dementia. Evidence for other domains of physical activity was scarce, derived from only a small number of studies. Dose–response meta-analyses and multiple sensitivity analyses were largely consistent with these domain-specific patterns.

The protective association between physical activity and dementia risk has been well documented.^4,5,110^ However, most of this evidence has originated from individual studies that assessed either total physical activity (ie, total min per week, regardless of domain) or leisure-time physical activity only. Previous studies have highlighted a protective association between leisure-time physical activity and improved brain health, potentially through mechanisms including upregulation of neurotrophic factors (eg, brain-derived neurotrophic factor)^111^ and improved cardiovascular health.^112^ Considering the feasibility of promoting physical activity in leisure compared with other domains, these findings have supported the development of public health policies, including WHO’s guidelines on physical activity and sedentary behaviour.^113^ However, much of the world population performs physical activity in other domains, especially occupational and household activities,^8^ reinforcing the need for a better understanding of how physical activity is associated with dementia risks in the context in which activity occurs.

Occupational physical activity has been linked to increased all-cause and cardiovascular mortality,^11,12,14^ although null or protective associations have also been reported for cardiovascular^13^ and brain health outcomes.^36,114^ Inconsistencies likely reflect how occupational activity and reference groups are defined, particularly the inclusion of non-working individuals. Nabe-Nielsen and colleagues^36^ examined the association between both leisure-time and occupational physical activity and risk of all-cause dementia. In one analysis, when the reference category included non-working participants and those working in sedentary jobs at baseline, occupational physical activity was associated with a reduced risk among men and women. However, the association was inverted in women and attenuated in men when only working participants were included in the analysis. Similarly, Huang and colleagues^114^ combined UK Biobank data from working and non-working participants to show that both leisure and occupational physical activity were associated with a reduced risk of dementia. The inclusion of non-working individuals in reference groups might introduce healthy worker bias. In studies assessing occupational physical activity in the current meta-analysis, the study population was restricted to working participants. Restricting analyses to employed populations and the use of detailed metrics of job exposure (eg, O*NET) might better clarify associations between occupational demands and brain health.

The deleterious association between occupational physical activity and adverse health outcomes could reflect broader adverse life circumstances, including socioeconomic disadvantage and stressful working conditions.^115,116^ Many physically demanding jobs are characterised by heightened psychosocial stress, hazardous occupational exposures, and reduced autonomy,^117–120^ which could also constrain opportunities for leisure-time physical activity.^115^ Additionally, these occupations are also associated with increased exposure to air and noise pollution, both of which have been linked to an increased risk of dementia.^3,44^ Consequently, the observed associations might partly reflect an accumulation of social, economic, and environmental adversities rather than be fully attributable to occupational physical activity per se. This potential aspect of risk should be interpreted through a structural lens, recognising these stressors as sociocultural drivers of health, rather than biological traits. Furthermore, much manual labour involves repetitive, low-complexity tasks that could limit the accrual of cognitive reserve, a factor independently linked to dementia risk.^121–123^

We observed considerable heterogeneity across domains, with high *I*^2^ values indicating that not all leisure-time or occupational physical activity exposures are equivalent.^124^ These exposures might differ meaningfully in intensity, context, duration, and the populations who perform them.^125^ This heterogeneity and the small number of studies for specific dementia subtypes were the primary drivers of the low to very low certainty of evidence across domains. Several factors could explain this variability. We identified methodological differences, for example, in how physical activity was measured and categorised and in how dementia outcomes were assessed as sources of heterogeneity in the moderation analysis. Contextually, the heterogeneity might also reflect the evolving research landscape spanning 1995–2026. This is a period marked by substantial shifts in occupational structures, the accelerating rise of automation, and the emergence of novel environmental risk factors, all of which might moderate the relationship between physical activity and dementia risk in ways that vary across settings and time periods. Taken together, these findings suggest the need to reorient prevention efforts away from individual-level behavioural changes and towards structural determinants, prioritising worker-centred policies and equitable urban planning that expand meaningful access to leisure-time physical activity.

The evidence on the association between both commuting and household physical activities and risk of dementia was limited to two studies: the UK Biobank study and the CAIDE study.^18,42^ Although these initial findings also suggest a domain-dependent association, with increased risk associated with active commuting and reduced risk linked to household activities, more studies are required to better understand these associations. For example, the increased risk of dementia associated with active commuting is consistent with previous evidence showing an elevated risk of cognitive impairment in middle-aged and older adults in Brazil (aged 35–74 years).^9^ Air pollution and socioeconomic position are factors that might explain this harmful link. Air pollution, which is independently associated with an increased risk of dementia,^126^ attenuated the beneficial association between physical activity and dementia risk in UK Biobank participants.^127^ Additionally, active commuting is often a necessity rather than a choice-led behaviour, especially in low-resource settings.^128^

The current study also revealed that countries where the number of dementia cases is expected to have a sharp increase are also those with the lowest number of studies investigating the role of physical activity in dementia prevention. This mismatch can be viewed through two complementary lenses. On one hand, the evidence base remains poorly representative of much of the world, particularly LMICs. On the other hand, the accumulated evidence provides a strong theoretical basis to justify and guide expanded longitudinal research across diverse sociocultural settings. Countries where the link between physical activity and dementia risk is most investigated (eg, the UK and the USA) are also showing a reduced incidence of dementia.^129,130^ Increased physical activity levels have been proposed as one of the contributors for this decrease.^129^ Studies on physical activity and dementia in these countries might have supported the development of effective public health initiatives to promote healthy ageing. However, the transferability of such insights cannot be assumed. Context-specific evidence is needed to understand which domains and promotion strategies are most feasible and effective, and under what conditions, across countries with distinct physical activity profiles and structural constraints.

This study has several limitations. First, most of the included studies relied on self-reported measures of physical activity, which are prone to recall and social desirability bias. Currently, objective measurements of physical activity do not reliably distinguish between domains of physical activity, although emerging algorithms based on machine learning have shown promise in classifying domain-specific activity patterns.^131^ Second, although bias-adjusted estimates remained consistent with the primary analysis, small-study effects cannot be ruled out. Third, the opposite direction in the association between both occupational and leisure-time physical activity and dementia risk persisted in the leave-one-out sensitivity analyses, although significance was lost in some exclusions. This finding suggests that the significance of the pooled estimate depends on the full body of evidence, rather than being robust to the exclusion of individual studies. Importantly, no single study materially altered the direction of the association, indicating that no influential study drives the observed effect. Fourth, evidence for other physical activity domains, such as active commuting and household, remains sparse, precluding robust dose–response meta-analyses and inferences. Fifth, we acknowledge the small number of high-quality studies and scarcity of investigations specifically addressing vascular dementia as an outcome. Sixth, the low response rates in some of the included studies might have led to an underestimation of effect sizes by under-representing frail, less physically active, and potentially more vulnerable individuals. Seventh, the possibility of reverse causation cannot be excluded, given the observational nature of the included studies and the relatively short follow-up periods in some cohorts. Among studies with follow-up exceeding 20 years, only one reported a sensitivity analysis excluding early incident cases (≥5 years) and assessed a specific domain (leisure-time physical activity).^76^ Although findings were consistent with the main analysis, the scarce availability of such analyses across the included studies precludes a definitive assessment of reverse causation, and future studies should incorporate this approach as a standard methodological safeguard. Furthermore, the competing risk of mortality warrants consideration, especially in long-term cohorts where cardiometabolic diseases can occur before a diagnosis of dementia. These limitations are underscored by the scarcity of data from randomised controlled trials, which are yet to replicate the robust associations observed in longitudinal cohorts.^132^ Finally, 69 (93%) included studies were conducted with samples from HICs, highlighting a substantial mismatch between where evidence on physical activity and dementia risk has been generated and where the projected burden of dementia is expected to increase most rapidly (ie, LMICs).

Despite these limitations, to our knowledge, this is the first meta-analysis to synthesise the evidence on the association between all major domains of physical activity and dementia risk in a large, international body of longitudinal evidence. Additionally, our approach aligns with recently proposed standards aimed at improving the quality and interpretability of meta-analyses of observational data. In this regard, our methodology addresses several criteria identified as indicators of high-quality evidence synthesis.^21^ Our findings suggest that the association between physical activity and risk of all-cause dementia is domain-specific. These results have public health implications, supporting the equitable access to leisure-time physical activity in prevention strategies and highlighting the need to consider worker-focused health policies aimed at mitigating potential harms in specific occupational contexts.

## Supplementary Material

1

## Figures and Tables

**Table:** Summary of studies included in the meta-analysis by physical activity domain

	Number of studies (*k*)*	Number of participants diagnosed with dementia	Follow-up, years	Quality score, % (poor; moderate; good)†	Diagnostic criteria‡
Leisure-time	20	12 297/596 677 (2·1%)	11·1 (5·6–20·5)	35%; 50%; 15%	DSM-III, DSM-IV, or DSM-V (26%); clinical evaluation (39%); health records (26%); cognitive tests (4%); and other§ (4%)
Occupational	5	7562/519 757 (1·5%)	21·0 (15·9–25·2)	0%; 60%; 40%	DSM-III, DSM-IV, or DSM-V (20%); and health records (80%)
Household	1	5185/500 270 (1·4%)	10·7 (10·7–10·7)	0%; 0%; 100%	Health records (100%)
Commuting	2	5229/501 719 (1·0%)	15·9 (13·3–18·4)	0%; 50%; 50%	Clinical evaluation (50%); and health records (50%)
Total or unspecified¶	55	49 630/3 630 887 (1·4%)	8·8 (5·0–21·8)	49%; 40%; 11%	DSM-III, DSM-IV, or DSM-V (45%); clinical evaluation (16%); health records (18%); cognitive tests (9%); and other§ (11%)

Data are n, n/N (%), or median (IQR), unless otherwise indicated. DSM=Diagnostic and Statistical Manual of Mental Disorders.

* Data are aggregated from 83 observations; some studies provided data for multiple cohorts or physical activity domains.

† Percentages for quality represent the proportion of studies within each domain. Quality assessment considered study design, cohort representativeness, method of assessing physical activity, confirmation that participants did not have dementia at baseline, approaches to confounder adjustment, procedures for outcome ascertainment, duration of follow-up, and attrition rates. Overall study quality was classified into three categories: high (selection ≥2·5 stars, comparability 1·0 star, and outcome ≥2·5 stars); moderate (selection ≥2·0 stars, comparability ≥0·5 stars, and outcome ≥2·0 stars); and low (selection <2·0 stars, comparability <0·5 stars, and outcome <2·0 stars).

‡ Includes all-cause dementia, Alzheimer’s disease, and vascular dementia. §Includes proxy interview, self-reported plus proxy interview, and mixed approaches (ie, health records and cognitive tests).

¶ Unspecified refers to studies that did not assess physical activity within a specific domain.

## Data Availability

Data, analysis codes, outputs, and associated study materials can be found at https://osf.io/takhb/overview?view_only=03434437735e4aee96983d6731a62d08.

## Research in context

### Evidence before this study

In a previous systematic review and meta-analysis, we searched PubMed, CINAHL, Scopus, PsycINFO, SPORTDiscus, and Web of Science from database inception to Oct 26, 2021, for prospective observational studies using keywords related to physical activity (eg, physical activity, sport, walking, running, athletics, and physical training), dementia outcomes (eg, dementia and Alzheimer’s disease), and study design (eg, prospective, longitudinal, follow-up, observational, and cohort). Eligible studies assessed the associations between physical activity and all-cause dementia, Alzheimer’s disease, or vascular dementia. In the study, physical activity was associated with reduced risk of dementia across 58 studies (16% classified as high quality) and 257 983 individuals. However, this evidence came primarily from studies investigating the correlation between total or leisure-time physical activity and dementia. For much of the world’s population, physical activity is performed in other domains, such as commuting, occupational, and household activities, where the association with dementia risk remains unclear.

### Added value of this study

In this systematic review and meta-analysis, we quantitatively synthesised data from 74 cohort and case–control studies comprising around 4 million individuals in 30 countries. Our findings show that the association between physical activity and dementia risk is not uniform across activity contexts, highlighting the importance of considering where and how physical activity is accumulated rather than relying on total physical activity alone. These findings help to explain inconsistencies in the existing literature and provide an evidence base to inform future epidemiological research and public health recommendations. Furthermore, we identified substantial geographical gaps in the evidence, particularly in regions projected to experience the greatest increases in dementia burden by 2050, underscoring priorities for future research.

### Implications of all the available evidence

The association between physical activity and dementia risk might be domain-specific. Therefore, public health strategies might benefit from considering the context of physical activity, given that the potential protective associations were most consistent for leisure-time physical activities. By contrast, occupational physical activity might be associated with an increased risk of dementia. This deleterious association might reflect not only physical demands, but also socioeconomic disadvantage, psychosocial stress, and hazardous environmental exposures that frequently accompany physically demanding occupations. Given the projected uneven rise in dementia incidence worldwide over the next 25 years, future researchers should focus on how the context of physical activity influences brain health across various societies to provide refined estimates of the true burden of dementia attributable to physical inactivity.
