# Supplementary material for: Domain-specific physical activity levels and risk of dementia: a systematic review and meta-analysis
Source: Lancet Public Health. Author manuscript; Available in PMC 2026 Sep 1. (PMC13532401; doi:10.1016/S2468-2667(26)00142-8)
Supplement: Supplementary file 1 [file NIHMS2205277-supplement-1.pdf]

THE LANCET
Public Health

Supplementary appendix

This appendix formed part of the original submission and has been peer reviewed. We post it as supplied by the authors.

Supplement to: Feter N, Iso-Markku P, Markarian T, et al. Domain-specific physical activity levels and risk of dementia: a systematic review and meta-analysis. *Lancet Public Health* 2026; **11:** e650–64.

APPENDIX

Supplementary Methods S1. Deviation from the original protocol. 2
Supplementary Methods S2. Search strategy. 5
Supplementary Methods S3. Search strategy in the updated literature search. 6
Supplementary Methods S4. A quality assessment tool developed by Iso-Markku et al.[1] for the quality assessment of cohort studies addressing the association of physical activity and dementia. 7
Supplementary Methods S5. A quality assessment tool developed by Iso-Markku et al.[1] for the quality assessment of case-control studies addressing the association of physical activity and dementia. 9
Supplementary Methods S6. Study-specific considerations during data extraction. 11
Supplementary Methods S7. Conversion of hazard ratio and odds ratio to relative risks 12
Supplementary Methods S8. Methods description for dose-response meta-analyses. 13
Supplementary Methods S9. Records screened and excluded during title, abstract, and full-text screening. 14
Supplementary Methods S10. Selection and Exclusion of Overlapping Cohort Studies 140
Supplementary Figure S1: Study quality assessment across methodological domains 141
Supplementary Figure S2. Distribution of study quality ratings by methodological domain 142
Supplementary Figure S3. Pooled association between physical activity and all-cause dementia risk. 143
Supplementary Figure S4. Contour-enhanced funnel plot for assessment of publication bias in studies assessing the association between physical activity and the risk of all-cause dementia. 144
Supplementary Figure S5. Egger regression plot assessing small-study effects in the association between physical activity and all-cause dementia. 145
Supplementary Figure S6. Pooled association between physical activity and risk of Alzheimer's disease. 146
Supplementary Figure S7. Contour-enhanced funnel plot for assessment of publication bias in studies assessing the association between physical activity and the risk of Alzheimer's disease. 147
Supplementary Figure S8. Egger regression plot assessing small-study effects in the association between physical activity and Alzheimer's disease. 148
Supplementary Figure S9. Pooled association between physical activity and risk of vascular dementia. 149
Supplementary Figure S10. Contour-enhanced funnel plot for assessment of publication bias in studies assessing the association between physical activity and the risk of vascular dementia. 150
Supplementary Figure S11. Egger regression plot assessing small-study effects in the association between physical activity and vascular dementia. 151
Supplementary Figure S12. Leave-one-out sensitivity analysis for the association between physical activity and risk of all-cause dementia. 152
Supplementary Figure S13. Influence of individual studies on the statistical significance of the moderating effect of physical activity domains. 153
Supplementary Figure S14: Association between domain-specific physical activity and dementia risk, including leave-one-out sensitivity analyses. 154
Supplementary Figure S15. Mismatch between the projected number of cases of dementia from 2019 to 2050 (upper map) and the number of studies conducted in each country (lower map) by the World Bank's country income level. 155
Supplementary Table S1. Characteristics of the included studies. 156
Supplementary Table S2. Sensitivity analysis estimating the pooled relative risk for all-cause dementia, Alzheimer's disease, and vascular dementia associated with physical activity accounting for distinct covariates. 165
Supplementary Table S3. Sensitivity analysis estimating the risk of all-cause and specific-cause dementia associated with physical activity using studies that used baseline age, education, chronic diseases, or APOE ε4 status as covariates. 166
Supplementary Table S4. Sensitivity analysis excluding studies rated as low quality. 167
Supplementary Table S5. Multivariate meta-regression of physical activity domains on dementia risk, adjusted for physical activity volume. 168
Supplementary Table S6. Summary of Findings Table (GRADE) 169
REFERENCES 170

Supplementary Methods S1. Deviation from the original protocol.

Published protocol - https://www.crd.york.ac.uk/PROSPERO/view/CRD420251010899.

Review objectives
To systematically evaluate the association between domain-specific physical activity and the risk of dementia.
Deviation: None.

To assess potential dose-response relationships between physical activity and dementia risk.
Deviation: None.

To examine the influence of demographic factors, such as age and sex, on these associations.
Deviation: None.

To identify gaps in the literature and suggest future research directions.
Deviation: None.

ELIGIBILITY CRITERIA
Population
Included
Adults with no prevalent dementia
Excluded
Adults with dementia
Deviation: None.

Intervention(s) or exposure(s)
Included
Domain-specific physical activity
Deviation: None.

Comparator(s) or control(s)
This review does not have any comparators
Deviation: None.

Study design
Only nonrandomized study types will be included.
Included
Cohort studies
Excluded
Non-cohort studies.
Deviation: To keep consistent with the meta-analysis conducted by Iso-Markku et al.[1], we also included case-control studies.

Context
No restriction regarding research context.
Deviation: None.

SEARCHING AND SCREENING
Search for unpublished studies
Both published and unpublished studies will be sought.
Deviation: To keep consistent with the meta-analysis conducted by Iso-Markku et al.[1], we only included peer-reviewed articles.

Main bibliographic databases that will be searched
The main databases to be searched are *CINAHL*, *Embase*, *LILACS*, *PsycInfo*, *PubMed* and *Scopus*.

Deviation: To keep consistent with the meta-analysis conducted by Iso-Markku et al.[1], we searched for potential articles in Web of Science and SPORTDiscuss, CINAHL, PsycInfo, Scopus, and MEDLINE/PubMed. We did not include LILACS and EMBASE.

Search language restrictions
There are no language restrictions.
Deviation: None.

Search date restrictions
Databases will be searched for articles published by 1 January 2021, there are no search end date restrictions. Search first took place on September 03, 2025 and was updated on May 2, 2026.
Deviation: None.

Other methods of identifying studies
Other studies will be identified by: *looking through all the articles that cite the papers included in the review ("snowballing")*.
Deviation: Forward citation tracking was not performed. The review relied on a comprehensive multi-database search combined with manual screening of reference lists from all included studies. Given the specificity of the exposure and outcome definitions and the high overlap of retrieved records across databases, this approach was deemed sufficient to identify the relevant evidence base.

Link to search strategy
A full search strategy is not available.
Deviation: The full search strategy is available in the Supplementary Methods 2.

Selection process
Studies will be screened independently by at least two people (or person/machine combination) with a process to resolve differences.
Deviation: None.

DATA COLLECTION PROCESS
Data extraction from published articles and reports
Data will be extracted independently by at least two people (or person/machine combination) with a process to resolve differences. Authors will be asked to provide any required data not available in published reports. Study datasets/IPD will be obtained from study investigators or via a data repository We will contact corresponding authors from the included studies.
Deviation: None.

Study risk of bias or quality assessment
Risk of bias will be assessed using: *Newcastle-Ottawa*
Deviation: We used the quality assessment tool developed by Iso-Markku et al.[1]

Data will be assessed independently by at least two people (or person/machine combination) with a process to resolve differences.
Deviation: None.

Additional information will be sought from study investigators if required information is unclear or unavailable in the study publications/reports.
Deviation: None.

Reporting bias assessment
Publication bias will be assessed using Egger test and funnel plot.
Deviation: Small-study effects were assessed visually using funnel plots. Because Egger's test is not available for multilevel models,[2] we applied a standardized regression of logRRs on the inverse standard error. When asymmetry was detected, PET–PEESE analyses were used to evaluate robustness to small-study bias.[3]

Certainty assessment
Certainty of the evidence will be assessed using GRADE assessment.
Deviation: None.

OUTCOMES TO BE ANALYSED

Main outcomes
Incident Dementia; Incident Alzheimer's disease
Deviation: We added vascular dementia.

Additional outcomes
There are no additional outcomes.
Deviation: None.

PLANNED DATA SYNTHESIS

Strategy for data synthesis
"Data synthesis will involve multi-level meta-analysis and dose-response meta-analysis for quantitative analysis. The multi-level meta-analysis will account for the hierarchical structure of the data, allowing the inclusion of multiple effect sizes from the same study while addressing within- and between-study variance. The dose-response meta-analysis will be conducted using a restricted cubic spline model to assess potential non-linear relationships between exposure and outcome. Statistical analyses will be performed using the 'metafor' and 'dosresmeta' packages in R."
Deviation: None.

AUTHORS

The systematic review and meta-analysis protocol was initially registered with PROSPERO listing Natan Feter and Jayne Feter as the primary investigators. As the research questions evolved and the project scope expanded, from a targeted search to a comprehensive international meta-analysis of over 80 cohorts, the research team was progressively expanded to incorporate specialized expertise.

This collaborative expansion was essential to ensure the methodological rigor of the current work, including advanced statistical dependency modeling, systematic data extraction across multiple language sources, and domain-specific epidemiological expertise. The final authorship team, listed below, reflects this expanded collaborative effort:

Natan Feter, PhD · Paula Iso-Markku, PhD · Tiladia Markarian, Dylan M. Luong, Jack Gunnink, Natália Schröder, MSc · Eduardo Caputo, PhD · Jayne Feter, PhD · Anamika Nanda, Sarah Hourihan, Gustavo S da Silva, MSc · Ashley Ventura, Daniela Benzano Bumaguin, PhD · Yann C. Klimentidis, PhD · Gene E. Alexander, PhD · Daniel Umpierre, PhD · Pedro C. Hallal, PhD · Airton José Rombaldi, PhD · David A. Raichlen, PhD.

Supplementary Methods S2. Search strategy.

The full search strategy was replicated from Iso-Markku et al.[1] We chose not to include keywords related to cognitive function (e.g., TELE, reasoning) due to the scope of the present analysis and because Iso-Markku et al.[1] reported that a search excluding those terms and focusing on dementia-related keywords identified the same studies when using a broader search strategy.

Database	Outcomes	Physical activity	Study design	Filters	Records
PubMed	Dementia OR Alzheimer* OR "Vascular dementia"	"physical activity" OR "aerobic exercise" OR sport* OR walking OR cycling OR "physical training" OR "physical training" OR "physical training" OR "physical inactivity" OR exercise	Prospective OR longitudinal OR follow-up OR observational OR cohort*	01/01/2021 onwards	2,053
Web of Science	dementia (Topic) or Alzheimer* (Topic) or vascular dementia (Topic)	physical activity (Topic) OR aerobic exercise (Topic) OR sport* (Topic) OR walking (Topic) OR physical training (Topic) OR cycling (Topic) OR physical inactivity (Topic) OR exercise (Topic)	prospective (Topic) OR longitudinal (Topic) OR "follow-up" (Topic) OR observational (Topic) OR cohort* (Topic)	01/01/2021 onwards	2,439
Scopus	TITLE-ABS-KEY(dementia OR Alzheimer* OR "vascular dementia")	TITLE-ABS-KEY("physical activity" OR "aerobic exercise" OR sport* OR walking OR "physical training" OR cycling OR "physical inactivity" OR exercise)	TITLE-ABS-KEY(prospective OR longitudinal OR "follow-up" OR observational OR cohort*)	01/01/2021 onwards	2,842
PsycInfo	Any Field: dementia OR Any Field: Alzheimer* OR Any Field: vascular dementia	Any Field: physical activity OR Any Field: aerobic exercise OR Any Field: sport* OR Any Field: walking OR Any Field: physical training OR Any Field: cycling OR Any Field: physical inactivity OR Any Field: exercise	Any Field: prospective OR Any Field: longitudinal OR Any Field: follow-up OR Any Field: observational OR Any Field: cohort*	01/01/2021 onwards	440
CINAHL	XB dementia OR XB Alzheimer* OR XB "vascular dementia"	XB "physical activity" OR XB "aerobic exercise" OR XB sport* OR XB walking OR XB "physical training" OR XB cycling OR XB "physical inactivity" OR XB exercise	(prospective OR longitudinal OR "follow-up" OR observational OR cohort*)	01/01/2021 onwards	327
SPORTDiscus	XB dementia OR XB Alzheimer* OR XB "vascular dementia"	XB "physical activity" OR XB "aerobic exercise" OR XB sport* OR XB walking OR XB "physical training" OR XB cycling OR XB "physical inactivity" OR XB exercise	(prospective OR longitudinal OR "follow-up" OR observational OR cohort*)	01/01/2021 onwards	46

Supplementary Methods S3. Search strategy in the updated literature search.

Database	Outcomes	Physical activity	Study design	Filters	Records
PubMed	Dementia OR Alzheimer* OR “Vascular dementia”	"physical activity" OR "aerobic exercise" OR sport* OR walking OR cycling OR "physical training" OR "physical training" OR "physical training" OR "physical inactivity" OR exercise	Prospective OR longitudinal OR follow-up OR observational OR cohort*	01/01/2025 onwards	827
Web of Science	dementia (Topic) or Alzheimer* (Topic) or vascular dementia (Topic)	physical activity (Topic) OR aerobic exercise (Topic) OR sport* (Topic) OR walking (Topic) OR physical training (Topic) OR cycling (Topic) OR physical inactivity (Topic) OR exercise (Topic)	prospective (Topic) OR longitudinal (Topic) OR "follow-up" (Topic) OR observational (Topic) OR cohort* (Topic)	09/03/2025 onwards	486
Scopus	TITLE-ABS-KEY(dementia OR Alzheimer* OR "vascular dementia")	TITLE-ABS-KEY("physical activity" OR "aerobic exercise" OR sport* OR walking OR "physical training" OR cycling OR "physical inactivity" OR exercise)	TITLE-ABS-KEY(prospective OR longitudinal OR "follow-up" OR observational OR cohort*)	01/01/2025 onwards	1,164
PsycInfo	Any Field: dementia OR Any Field: Alzheimer* OR Any Field: vascular dementia	Any Field: physical activity OR Any Field: aerobic exercise OR Any Field: sport* OR Any Field: walking OR Any Field: physical training OR Any Field: cycling OR Any Field: physical inactivity OR Any Field: exercise	Any Field: prospective OR Any Field: longitudinal OR Any Field: follow-up OR Any Field: observational OR Any Field: cohort*	01/01/2025 onwards	151
CINAHL	XB dementia OR XB Alzheimer* OR XB "vascular dementia"	XB "physical activity" OR XB "aerobic exercise" OR XB sport* OR XB walking OR XB "physical training" OR XB cycling OR XB "physical inactivity" OR XB exercise	(prospective OR longitudinal OR "follow-up" OR observational OR cohort*)	09/01/2025 onwards	38
SPORTDiscus	XB dementia OR XB Alzheimer* OR XB "vascular dementia"	XB "physical activity" OR XB "aerobic exercise" OR XB sport* OR XB walking OR XB "physical training" OR XB cycling OR XB "physical inactivity" OR XB exercise	(prospective OR longitudinal OR "follow-up" OR observational OR cohort*)	09/01/2025 onwards	11

Supplementary Methods S4. A quality assessment tool developed by Iso-Markku et al.[1] for the quality assessment of cohort studies addressing the association of physical activity and dementia.

Note: A study can be given a maximum of one star for each numbered item within the Selection, Comparability and Outcome categories.

Selection

1) Representativeness of the exposed cohort
   a) Truly representative (represents well the whole age group or the whole age group of any particular race and is not selected in regards to some disease, socio-economic status or for example only inhabitants of a nursing home, participation rate > 70% (of those alive) and sample size at least 1000 *(one star)*
   b) Truly or somewhat representative (participation rate 50-70%) and sample size >1000 *(half a star)*
   c) Selected group in regards to some characteristic or participation rate < 50% or sample size < 1000
   d) No description of the derivation of the cohort or participation rate lacks

2) Performance quality (adapted from (Br J Sports Med 2017;51:1410-18))
   a) Good: PA assessed with a structured questionnaire of the duration, frequency and intensity of PA or the intensity of PA assessed with a structured question. Or PA assessed with an objective measure of PA (eg, accelerometer) (one star)
   b) Moderate: Participation only in some types of sports assessed but other activities not considered or assessment of intensity lacks. Frequency or duration are assessed. (half a star)
   c) Low: A "yes" or "no" question used. Frequency and duration not assessed. Or physical activity index on versatility of sports and somewhat physical household chores but not assessing intensity, frequency or duration. Or not described how exercise or physical activity was measured. (no star)

3) Demonstration that outcome of interest was not present at start of study
   a) Yes. In a study population whose average age > 55 years, valid measure of cognition is used and demented individuals and individuals with mild cognitive impairment at baseline according to baseline cognition screening have been excluded or population is in midlife (mean age or median < 55 years and maximum age 65 years or +1 SD < 60 years) *(one star)*
   b) No

Comparability

4) Comparability of cohorts on the basis of the design or analysis controlled for confounders
   a) The study controls for the following four factors: age, sex (or all cohort members represent the same sex), some vascular risk factor† and education or a measure of general cognitive ability at baseline (education criterion is not needed if all cohort members have the same education level). In addition, the results have been adjusted with baseline cognition in study population whose average age > 55 years (one star)
   b) The study controls only for three of the factors presented above (age, sex, some vascular risk factor and education or a measure of general intelligence) or/and in study populations whose mean age > 55 years the results have not been adjusted with baseline cognition or the sociodemographic and health behaviors controlled for are not specified further (half a star)
   c) Cohorts are not comparable on the basis of covariates controlled for (no star)

Outcome

5) Assessment of outcome
   a) A validated measure of dementia ***(one star)***
   b) Record linkage ***(half a star)***
   c) Self-report or other
   d) No description
6) Was follow-up long enough for outcomes to occur
   a) Yes (***one star)***
   b) No

NOTE: Indicate the median duration of follow-up and a brief rationale for the assessment above: 10 years in dementia studies.

7) Adequacy of follow-up of cohorts
   a) Complete follow up- all subject accounted for ***(one star)***
   b) Subjects lost to follow up unlikely to introduce bias- number lost less than or equal to 20%* (when follow-up less than 10 years) or 30%* (when follow-up at least 10 years) of those alive or description of those lost suggested no different from those followed. ***(one star)***
   c) Follow up rate less than 80%* (when follow-up less than 10 years) or less than 70%* (when follow-up longer than 10 years) and no description of those lost.
   d) No statement

* Follow-up rates are calculated taking into account only the cohort members who have been alive at the time of the follow-up

† Vascular risk factor signifies a cardiovascular disease or information on smoking, body mass index, cholesterol levels, diet, blood pressure, diabetes or blood glucose. Thresholds for converting the Newcastle-Ottawa scales to AHRQ standards (good, moderate, and poor):

High quality: Selection: 2,5 - 3 stars, Comparability: 1 star, Outcome: 2,5 - 3 stars
Moderate quality: Selection: 2-3 stars, Comparability: 0,5 -1 star, Outcome: 2-3 stars,
Low quality: studies not reaching Moderate/Good quality

Supplementary Methods S5. A quality assessment tool developed by Iso-Markku et al.[1] for the quality assessment of case-control studies addressing the association of physical activity and dementia.

Note: A study can be given a maximum of one star for each numbered item within the Selection, Comparability, and Outcome categories.

Baseline

1) Selection of controls:
   a) Community controls **(half a star)**
   b) Hospital controls
   c) No description

2) Definition of controls:
   a) No history of disease (endpoint) **(half a star)**
   b) No description of source

3) Demonstration that outcome of interest was not present at start of study
   a) Yes. In a study population whose average age > 55 years, valid measure of cognition is used and demented individuals and individuals with mild cognitive impairment at baseline according to baseline cognition screening have been excluded or population is in midlife (mean age or median < 55 years and maximum age 65 years or +1 SD < 60 years) **(one star)**
   b) No

4) Performance quality (adapted from (Br J Sports Med 2017;51:1410-18))
   a) Good: PA assessed with a structured questionnaire of the duration, frequency and intensity of PA or the intensity of PA assessed with a structured question. Or PA assessed with an objective measure of PA (eg. accelerometer). Additionally same method is used for cases and controls and non-response rate is the same for cases and controls. **(one star)**
   b) Moderate: Participation only in some types of sports assessed but other activities not considered or assessment of intensity lacks. Frequency or duration are assessed. **(half a star)**
   c) Low: A "yes" or "no" question used. Frequency and duration not assessed. Or physical activity index on versatility of sports and somewhat physical household chores but not assessing intensity, frequency or duration. (no star)

Comparability

5) Comparability of cohorts on the basis of the design or analysis controlled for confounders
   a) The study controls for the following four factors: age, sex (or all cohort members represent same sex), some vascular risk factor† and education or a measure of general cognitive ability at baseline. In addition, the results have been adjusted with baseline cognition in study population whose average age > 55 years **(one star)**
   b) The study controls only for three of the factors presented above (age, sex, some vascular risk factor and education or a measure of general intelligence) or in study populations whose mean age > 55 years the results have not been adjusted with baseline cognition **(half a star)**
   c) Cohorts are not comparable on the basis of covariates controlled for (no star)

Outcome

6) Assessment of outcome
   a) A validated measure of dementia (if many cognitive tests used, most validated) ***(one star)***
   b) Record linkage ***(half a star)***
   c) Self-report or other
   d) No description

7) Was follow-up long enough for outcomes to occur

a) Yes (***one star)***
b) No

NOTE: Indicate the median duration of follow-up and a brief rationale for the assessment above: 10 years in dementia studies.

8) Is the case definition adequate?
   a) Yes, with a valid measure of dementia **(half a star)**
   b) No, does not fulfil the criteria defined above

9) Representativeness of the cases:
   a) Consecutive or obviously representative series of cases **(half a star)**
   b) Potential for selection biases or not stated

† Vascular risk factor signifies a cardiovascular disease or information on smoking, body mass index, cholesterol levels, diet, blood pressure, diabetes, or blood glucose. Thresholds for converting the Newcastle-Ottawa scales to AHRQ standards (good, moderate, and poor):

High quality: Baseline: 2,5 - 3 stars, Comparability: 1 star, Outcome: 2,5 - 3 stars
Moderate quality: Baseline: 2-3 stars, Comparability: 0,5 -1 star, Outcome: 2-3 stars,
Low quality: studies not reaching Moderate/Good quality

Supplementary Methods S6. Study-specific considerations during data extraction.

For selected studies, additional procedures were applied during data extraction to ensure comparability and methodological consistency across the meta-analysis. In Zotcheva et al.,[4] relative risks for dementia were calculated directly from published cell counts, as the authors reported only relative risk ratios estimated from multinomial logistic regression. For Jung et al.,[5] we restricted the analysis to participants aged 40–59 years, as the older cohort (≥60 years) lacked baseline cognitive assessment, limiting the ability to account for potential reverse causality. Additionally, in one study previously classified as not adjusting for education,[6] the authors adjusted for socioeconomic position using a composite indicator reflecting the educational requirements of the occupation and supervisory responsibility,[7] which we considered as a valid proxy for educational attainment. In Li et al.,[8] the authors pooled data from six US-based cohort studies: the Atherosclerosis Risk in Communities (ARIC) study, the Cardiovascular Health Study (CHS), the Framingham Heart Study (FHS), the Kuakini Honolulu-Asia Aging Study (HAAS), the Multi-Ethnic Study of Atherosclerosis (MESA), and the Sacramento Area Latino Study on Aging (SALSA). However, we included only the ARIC, FHS, and MESA cohorts, as these studies had a mean baseline age below 55 years. The remaining cohorts had baseline ages ≥66·5 years and did not include baseline cognitive function as a covariate in the models, limiting comparability with the eligibility criteria adopted in our review.

Supplementary Methods S7. Conversion of hazard ratio and odds ratio to relative risks

Summary statistics were relative risks (RRs) with 95% CIs. For studies that did not report RR data, ORs or HRs were converted into RRs. For studies where dementia incidence was less than 10%, OR data were converted to RRs using the formula below:[9]

$RR = \frac{OR}{(1-p_{0+}\ (p_0 * OR)}$, where p_0 is the dementia incidence in the whole study sample.

When dementia incidence was >10%, we used the square root transformation of OR, as recommended.[10]

We transformed HRs into RRs using the following formula:

$$RR = \frac{(1 - e^{HR*\ln(1-r)}}{r}$$

where r is the incidence rate of dementia for the reference group.[11] A separate RR was calculated for each higher PA category reported in the included study by comparing each higher PA category to the lowest PA level in the study (i.e., reference category)

Supplementary Methods S8. Methods description for dose-response meta-analyses.

Assessment and harmonization of physical activity exposure

Physical activity exposure was harmonized across studies by assigning a quantitative value to each reported exposure category. For categorical physical activity measures, we used the central tendency of each category (mean or median when available, or the midpoint of the reported range) to represent exposure intensity. When the original studies provided activity-specific metabolic equivalent (MET) values, these were retained. Otherwise, standardized MET values were applied: 3.5 METs for walking, 4.5 METs for moderate-intensity activity, and 8.0 METs for vigorous or sports-related activity.[12] For studies reporting PA frequency as sessions or bouts per week, each bout was assumed to last 30 minutes unless otherwise specified.[1]

In instances where direct conversion to MET-minutes per week was not feasible, exposure values were imputed, when possible, using mean estimates derived from studies with comparable populations and PA assessment methods.[1] To limit the influence of implausibly high exposure levels, physical activity was capped at 21 hours of moderate-intensity activity per week, corresponding to 5040 MET-minutes per week.

Dose–response meta-analysis

For studies reporting three or more distinct exposure categories, linear, quadratic, and non-linear dose–response relationships were evaluated. Dose–response associations were examined using a two-stage random-effects meta-analytic framework implemented with the *dosresmeta* package in R. Pooled relative risks were estimated using restricted maximum likelihood methods. Non-linear associations were modelled using restricted cubic splines with knots placed at the 10th and 90th percentiles of the overall physical activity exposure distribution. A reference value of 200 MET-minutes per week was selected for all dose–response analyses, reflecting the approximate mean exposure level of reference categories across the included studies.

Supplementary Methods S9. Records screened and excluded during title, abstract, and full-text screening.

Record Title
Protocol of a randomized, single-blind, controlled trial of an 18-month, home-based, high-intensity, exercise intervention in older, cognitively unimpaired APOE Œµ4 carriers.
Voluntary Swimming Reduces Amyloid Pathology in an Alzheimer's Mouse Brain: An Integrated Amyloid PET/CT and Light-Sheet Microscopy Evaluation.
Cognitive reserve and its impact on cognitive and functional abilities, physical activity and quality of life following a diagnosis of dementia: longitudinal findings from the Improving the experience of Dementia and Enhancing Active Life (IDEAL) study.
Nomogram for predicting changes in cognitive function in community dwelling older adults with mild cognitive impairment based on Korea Longitudinal Study of Ageing Panel Data: a retrospective study.
Motoric cognitive risk syndrome and incident dementia in older adults from the Qu√©bec NuAge cohort.
Dementia onset among community-dwelling older adults with mild cognitive impairment: A longitudinal study.
A Program for Reinforcing Lifestyle Change Motivation and Lifestyle Behavior to Prevent Dementia in Community-Dwelling Middle-Aged and Older Adults: Applying the Health Belief Model.
The Association of Apathy With Incident Dementia: A Multiple Mediation Analysis of Cardiovascular Risk Factors.
Predicting Exercise Behavior Among Caregivers of Persons With Dementia,ÄîA Longitudinal Investigation Using an Extended Health Belief Model.
Socioeconomic Inequalities in Dementia Risk Among a Population-Based Cohort: Quantifying the Role of a Broad Combination of Lifestyle Factors.
Dementia incidence and population-attributable fraction for dementia risk factors in Republic of Korea: a 12-year longitudinal follow-up study of a national cohort.
Associations of Accelerometer,ÄêMeasured Physical Activity With Dementia, Anxiety, and Depression Among Older Adults.
Associations between the 24-h Activity Daily Cycle and Incident Dementia.
Association between trajectories of life-course group sports participation and dementia: A 3-year longitudinal study.
High-Density Lipoprotein Cholesterol and Cognitive Function in Older Korean Adults Without Dementia: Apolipoprotein E4 as a Moderating Factor.
Active Travel Mode and Incident Dementia and Brain Structure.
Associations Between Walking Pace, APOE-Œµ4 Genotype, and Brain Health in Middle-Aged to Older Adults.
Physical activity initiated from midlife on risk of dementia and cognitive impairment: The Health and Retirement Study.
RCT-based Social Return on Investment (SROI) of a Home Exercise Program for People With Early Dementia Comparing In-Person and Blended Delivery Before and During the COVID-19 Pandemic.
Cognitive Effects of Aerobic Exercise in Alzheimer's Disease: A Pilot Randomized Controlled Trial.
Relationship between physical activity and incidence of dementia in people aged 50 and over in Europe.
Cognitive Performance following Single- or Multi-Session Exercise Intervention in Middle Age: A Systematic Review.
Variation of the Risk Associated With Potentially Modifiable Risk Factors for Dementia Between Ethnic Groups Within One Country. A Retrospective Cohort Study Using Routinely Collected Health Data in Aotearoa New Zealand.
Sarcopenia association with physical and psychological indices in community-dwelling aged population, Birjand Longitudinal Aging Study (BLAS).
The association between dietary patterns, plasma lipid profiles, and inflammatory potential in a vascular dementia cohort.
Physical exercise frequency and cognition: a multicenter cross-sectional cohort study.
Cardiovascular Disease Risk Burden, Cognitive Impairments and Incident Dementia among Community-Dwelling Middle-Aged and Older Adults: An 8-Year Longitudinal Follow-up Study.
Determinants of physical activity engagement in older adults.
ENHANCE: a comparative prospective longitudinal study of cognitive outcomes after 3 years of hearing aid use in older adults.
Impact of dementia on post-hip fracture walking ability: a stratified analysis based on pre-fracture mobility in Swedish cohorts of older adults.
Psychosocial factors associated with physical activity in people with dementia: A pilot cross,Äêsectional study.
Changes in Physical Activity and the Risk of Dementia in Patients With New-Onset Type 2 Diabetes: A Nationwide Cohort Study.
Community-based going-out program and dementia onset in Japanese older adults: A longitudinal observational study.
Chronic physical activity and the prevention of Alzheimer's disease.
Individual changes in anthropometric measures after age 60¬†years: a 15-year longitudinal population-based study.
A feasibility randomized controlled trial of a community-level physical activity strategy for older adults with motoric cognitive risk syndrome.
Latent profile analysis of sleep characteristics of patients with mild cognitive impairment and influencing factors.
Atua√ß√£o fisioterap√™utica nos d√©ficits de cogni√ß√£o e incapacidades funcionais em pacientes com doen√ßa de Alzheimer: uma revis√£o integrativa.
Exploring the association of physical activity on cognitive function in older adults from observational and genetic insights: a combined NHANES and Mendelian randomization study.
Effects of 12-Weeks of Home-Based Exercise Training on Physical and Cognitive Function of Older Adults: Randomized Trial Comparing Virtual Versus Minimal Supervision in the Context of the Covid-19 Pandemic in Brazil.
Cardiovascular health, measured using Life's Essential 8, is associated with reduced dementia risk among older men and women.
Online longitudinal monitoring of brain health in former contact sport athletes: A study of acceptability and ethicality.
Dose-response association between device-measured physical activity and incident dementia: a prospective study from UK Biobank.
Effects of curved path walking compared to straight path walking in older adults with cognitive deficits: A systematic review and network meta,Äêanalysis.
Influence of individualized prevention recommendations after one year on the control of hypertension in 3,868 follow-up participants of the ELITE study.

Effectiveness of a brain exercise program using gamebased cognitive enhancement to reduce mild cognitive impairment among older adults in Pathum Thani Province, Thailand: a quasi-experimental study.
Metabolic Pathways Associated With Obesity and Hypertension in Black Caregivers of Persons Living With Dementia.
Cognitive Function, Healthy Lifestyle, and All-Cause Mortality among Chinese Older Adults: A Longitudinal Prospective Study.
Salivary Antimicrobial Peptide in Patients With Dementia Before and After Clinical Oral Rehabilitation Programme: A Randomised Controlled Trial.
Longitudinal, Bidirectional Association between Gait Speed and Cognitive Function in Community-Dwelling Older Adults without Dementia.
Assessment of the link between life purpose and health.
Association between playing cards/mahjong and risk of incident dementia among the Chinese older adults: a prospective cohort study.
Effectiveness of individually tailored exercise on functional capacity and mobility in nursing home residents.
Role of raising domestic animals in decreasing the risk of dementia occurrence in Chinese older adults: Evidence of a 10-year prospective cohort study.
Association of peak expiratory flow with motoric cognitive risk syndrome among older adults.
INTERVENCIONES FARMACOL√ìGICAS Y NO FARMACOL√ìGICAS PARA MEJORAR EL SUE√ëO EN EL DETERIORO COGNITIVO LEVE Y LA ENFERMEDAD DE ALZHEIMER EN FASE LEVE: UNA REVISI√ìN SISTEM√ÅTICA [EVIDENCIA COMENTADA].
Exercise program to reduce the risk of cognitive decline and physical frailty in older adults: study protocol for an open label double-arm clinical trial.
Joint effect of cognitive decline and walking ability on incidence of wandering behavior in older adults with dementia: A cohort study.
Modifiable dementia risk factors and AT(N) biomarkers: findings from the EPAD cohort.
Association between Perceived Stress and Motoric Cognitive Risk Syndrome in an Elderly Population: Rugao Longevity and Aging Study.
Triglycerides as a Shared Risk Factor between Dementia and Atherosclerotic Cardiovascular Disease: A Study of 125 727 Individuals.
The management of urinary incontinence in nursing homes: a scoping review.
Trajectory of Cardiometabolic Disease‚ÄìRelated Hospitalizations Before and After Dementia Diagnosis: A Matched Cohort Study.
Risk of Dementia After Hospitalization Due to Traumatic Brain Injury: A Longitudinal, Population-Based Study.
Isotemporal Substitution of Sedentary Behavior With Moderate to Vigorous Physical Activity Is Associated With Lower Risk of Disability: A Prospective Longitudinal Cohort Study.
Physical activity trajectories and their associations with health outcomes in older adults with mild cognitive impairment or dementia: a national cohort study.
Physical Activity, Alzheimer Plasma Biomarkers, and Cognition.
Sensory impairments and the risk of cognitive decline and dementia across sex, age, and regions: Longitudinal insights from Europe.
Physical Function, An Adjunct to Brain Health Score for Phenotyping Cognitive Function Trajectories in Older Age: Findings From The Irish Longitudinal Study on Ageing (TILDA).
Promoting independence in Lewy body dementia through exercise: the PRIDE study.
Design and Rationale of a Two-Armed Randomized Controlled Trial on Yoga/Brisk Walking-Based Lifestyle Modification on Dementia Risk Reduction, and Influence of ApoE Genotypes on the Intervention.
Comparing the care environment at farm‚Äêbased and regular day care for people with dementia in Norway‚ÄîAn observational study.
Development and External Validation of a Mortality Prediction Model for Community-Dwelling Older Adults With Dementia.
Western Diet and Cognitive Decline: A Hungarian Perspective‚ÄîImplications for the Design of the Semmelweis Study.
Impact of frailty status on the effect of a multidomain lifestyle intervention on cognition.
Cardiovascular Health and Biomarkers of Neurodegenerative Disease in Older Adults.
Late-Life Depressive Symptomatology, Motoric Cognitive Risk Syndrome, and Incident Dementia: The "NuAge" Study Results.
Cognitive reserve, modifiable-risk-factor profile and incidence of dementia: results from a longitudinal study of CFAS Wales.
Dietary carotenoids related to risk of incident Alzheimer dementia (AD) and brain AD neuropathology: a community-based cohort of older adults.
Cardiovascular Risk Factors, Alzheimer's Disease, and the MIND Diet: A Narrative Review from Molecular Mechanisms to Clinical Outcomes.
Differences between private and public primary health care centers and differences between men and women in antihypertensive care and cardiovascular prevention in all patients with hypertension treated in primary care in Stockholm County, Sweden.
Abdominal myosteatosis is associated with lower processing speed in a multiethnic cohort of older adults.
Neurologic Music Therapy with a Habilitative Approach for Older Adults with Dementia: A Feasibility Study.
Neuroprotective Dietary Patterns and Longitudinal Changes in Cognitive Function in Older Adults.
Mediating Factors Associated With Physical Activity in Older Adults at Increased Dementia Risk.
Cognition, Behavior, and Caregiver Stress in Dementia during the COVID-19 Pandemic: An Indian Perspective.
Does a Reduction in Receiving Assistance With Daily Activities Among Older Adults in Mexico Indicate An Increase in Unmet Needs or a Decrease in Needs for Care?
Personality and Transition From Mild Cognitive Impairment to Normal Cognition vs Dementia.
Physical Fitness and Cognitive Function in Persons with Dementia and their Caregiver.
Body Mass Index Trajectories Preceding Incident Mild Cognitive Impairment and Dementia.
Cycling Linked to 19% Lower Risk of Dementia in Large UK Biobank Study.
The feasibility of a sleep education program for informal dementia care dyads: A pilot randomized controlled trial.
"WOOP is my safe haven": A qualitative feasibility and acceptability study of the Wish Outcome Obstacle Plan (WOOP) intervention for spouses of people living with early‚Äêstage dementia.
Effectiveness and Feasibility of Nonpharmacological Interventions for People With Parkinson's Disease and Cognitive Impairment on Patient‚ÄêCentred Outcomes: Systematic Review and Meta‚ÄêAnalysis.

The Prevalence of Cognitive Impairment Among Medicare Beneficiaries Who Use Outpatient Physical Therapy.
Reduction of physical activity during the COVID-19 pandemic is related to increased neuropsychiatric symptoms in memory clinic patients.
Purpose in Life and Motoric Cognitive Risk Syndrome: Replicable Evidence from Two National Samples.
Dual-task performance in seniors with mild cognitive impairment and Alzheimer's disease: a longitudinal study.
The role of cognitive reserve in mediating HIV,Äêassociated neurocognitive disorders in older adults living with,Äêtreated HIV in Mbeya, Tanzania: A cross,Äêsectional observational study.
Exercise effects on backward walking speed in people with dementia: A randomized controlled trial.
The feasibility of a stepping exergame prototype for older adults with major neurocognitive disorder residing in a long-term care facility: a mixed methods pilot study.
Moderate-to-Vigorous Physical Activity at any Dose Reduces All-Cause Dementia Risk Regardless of Frailty Status.
Rest,Äêactivity rhythms and cognitive impairment and dementia in older women: Results from the Women's Health Initiative.
Sex-dependent association between selenium status and cognitive performance in older adults.
Association of Combined Healthy Lifestyle Factors With Incident Dementia in Patients With Type 2 Diabetes.
Exploring cognitive and brain oxygenation changes over a 1-year period in physically active individuals with mild cognitive impairment: a longitudinal fNIRS pilot study.
Association between physical activity and episodic memory and the moderating effects of the apolipoprotein Œµ4 allele and age.
Salivary Cortisol and Melatonin, Sleep, and Behavioral Patterns in Older Adults Living With Dementia.
Association of Physical Activity with Incidence of Dementia Is Attenuated by Air Pollution.
Factors Associated With Behavioral and Psychological Symptoms of Dementia: Prospective Observational Study Using Actigraphy.
The Relationship Between Physical Activity and Health-Related Quality of Life in People With Dementia: An Observational Study.
The Association Between Second-Line Oral Antihyperglycemic Medication on Types of Dementia in Type 2 Diabetes: A Nationwide Real-World Longitudinal Study.
Cognitive Function and the Relationship With Health Literacy and Secondary Prevention in Patients With Acute Coronary Syndrome at Early Discharge: A Prospective Observational Study.
Prediction of Institutionalization for Patients With Dementia in Taiwan According to Condition at Entry to Dementia Collaborative Care.
Associations of Social Isolation and Loneliness With Later Dementia.
Pelvic floor muscle training for urinary incontinence in older adults: a best practice implementation project.
Energetic Cost of Walking and Brain Atrophy in Mid-to-Late Life.
Tackling Dementia: A Systematic Review of Interventions Based on Physical Activity.
Community-Dwelling Older Adults and Physical Activity Recommendations: Patterns of Aerobic, Strengthening, and Balance Activities.
Eliciting brain waves of people with cognitive impairment during meditation exercises using portable electroencephalography in a smart-home environment: a pilot study.
Promoting Physical Activity in a Primary Care Practice in People Living with Dementia and Their Family Caregivers.
Backward relative to forward walking speed and falls in older adults with dementia.
Can mild cognitive impairment with depression be improved merely by exercises of recall memories accompanying everyday conversation? A longitudinal study 2016-2019.
Low Risk of Dementia in Patients With Newly Diagnosed Atrial Fibrillation and a Clustering of Healthy Lifestyle Behaviors: A Nationwide Population-Based Cohort Study.
Association of objectively measured physical activity with incidence disability in older adults with/without social isolation.
Association Between Subjective Age and Motoric Cognitive Risk Syndrome: Results From a Population-Based Cohort Study.
Nurse-led physical activity interventions for people with dementia in nursing homes: A systematic review on intervention characteristics and implementation facilitators/barriers.
Five-year changes in objectively measured cardiorespiratory fitness, physical activity, and sedentary time in mid-to-late adulthood.
Risk factors for decline in gait speed during walking while talking in older adults.
Association of coffee, green tea, and caffeine with the risk of dementia in older Japanese people.
NeuroExercise: The Effect of a 12-Month Exercise Intervention on Cognition in Mild Cognitive Impairment,ÄîA Multicenter Randomized Controlled Trial.
Predictors of attrition in a longitudinal population-based study of aging.
Balancing fracture risk versus risk of mortality before fracture among women aged 80 years or older.
Mild Traumatic Brain Injuries and Future Risk of Developing Alzheimer's Disease: Systematic Review and Meta-Analysis.
Impact of various intensities and frequencies of non-occupational physical activity on the risk of dementia among physically independent older adults: the Japan Gerontological Evaluation Study.
Physical activity moderates the association between white matter hyperintensity burden and cognitive change.
Educational programs and interventions for health care staff to prevent and manage aggressive behaviors in acute hospitals: a systematic review.
Association of open skill exercise and long-chain polyunsaturated fatty acid intake with brain volume changes among older community-dwelling Japanese individuals.
Associations Between Neighborhood Park Access and Longitudinal Change in Cognition in Older Adults: The Multi-Ethnic Study of Atherosclerosis.
Reshaping the path of mild cognitive impairment by refining exercise prescription: a study protocol of a randomized controlled trial to understand the "what," "for whom," and "how" of exercise to promote cognitive function.
Pet Caretaking and Risk of Mild Cognitive Impairment and Dementia in Older US Adults.

Adherence to the Class-Based Component of a Tai Chi Exercise Intervention for People Living With Dementia and Their Informal Carers.
The Ability of Physical Performance Measures to Identify Fall Risk in Older Adults Living With Dementia: A Systematic Review and Meta-Analysis.
Increase in skeletal muscular adiposity and cognitive decline in a biracial cohort of older men and women.
Collecting data on the experiences and perspectives of people with dementia in the acute care hospital setting: A systematic scoping review.
Development and preliminary validation of a virtual reality memory test for assessing visuospatial memory.
Neighborhood Sidewalk Environment and Incidence of Dementia in Older Japanese Adults: The Japan Gerontological Evaluation Study Cohort.
Physical Activity in Early- and Mid-Adulthood Are Independently Associated With Longitudinal Memory Trajectories in Later Life.
Animal-assisted and robotic animal-assisted interventions within dementia care: A systematic review.
Strategies used by people with Alzheimer's disease for outdoor wayfinding: A repeated observational study.
Immediate effects of treadmill walking in individuals with Lewy body dementia and Huntington's disease.
Changes in time spent walking and the risk of incident dementia in older Japanese people: the Ohsaki Cohort 2006 Study...Tomata Y, Zhang S, Sugiyama K, et al. Changes in time spent walking and the risk of incident dementia in older Japanese people: the Ohsaki Cohort 2006 Study. Age & Ageing. ...
Sense of Purpose in Life Is Associated with Lower Risk of Incident Dementia: A Meta-Analysis.
Social health, social reserve and dementia.
Long‚Äêterm impact of the COVID‚Äê19 pandemic on facility‚Äê and home‚Äêdwelling people with dementia: Perspectives from professionals involved in dementia care.
Poor Sleep Predicts Increased Pain Perception Among Adults With Mild Cognitive Impairment.
The Association Between Alzheimer's Disease-Related Markers and Physical Activity in Cognitively Normal Older Adults.
Physical Activity and its Influencing Factors in Community-Dwelling Older Adults With Dementia: A Path Analysis.
Clinical‚Äêbased oral rehabilitation programme improved the oral diadochokinesis and swallowing function of older patients with dementia: A randomised controlled trial.
Association of Type 2 Diabetes, According to the Number of Risk Factors Within Target Range, With Structural Brain Abnormalities, Cognitive Performance, and Risk of Dementia.
Changes in objectively measured lifestyle factors during the COVID-19 pandemic in community-dwelling older adults.
Longitudinal Assessment of Physical Activity and Cognitive Outcomes Among Women at Midlife.
Obesity and Oxidative Stress in Older Adults At Risk for Dementia: A Magnetic Resonance Spectroscopy Study.
Plasma Brain-Derived Neurotropic Factor Levels Are Associated with Aging and Smoking But Not with Future Dementia in the Rotterdam Study.
Deconditioning in people living with dementia during the COVID-19 pandemic: qualitative study from the Promoting Activity, Independence and Stability in Early Dementia (PrAISED) process evaluation.
Incidence and Risk Factors of Falls Among Older People in Nursing Homes: Systematic Review and Meta-Analysis.
Physical Activity Engagement After Tai Ji Quan Intervention Among Older Adults With Mild Cognitive Impairment or Memory Concerns: A Secondary Analysis of a Randomized Clinical Trial.
From evidence to practice: Developing best practice guidelines for the delivery of activities to people living with moderate to advanced dementia using a pragmatic observational study.
Measuring Physical Activity Levels in People With Mild Cognitive Impairment or Mild Dementia.
6-month consequences of COVID-19 in patients discharged from hospital: a cohort study.
Does playing a musical instrument reduce the incidence of cognitive impairment and dementia? A systematic review and meta-analysis.
Association of APOE Œµ4 genotype and lifestyle with cognitive function among Chinese adults aged 80 years and older: A cross-sectional study.
What Are the Benefits of Pet Ownership and Care Among People With Mild-to-Moderate Dementia? Findings From the IDEAL programme.
Healthy Lifestyle, Genetic Risk and Brain Health: A Gene-Environment Interaction Study in the UK Biobank.
What Can Longitudinal Observational Studies of Physical Activity Teach Us About Prevention of Dementia?
Evaluating the effectiveness of stepwise swallowing training on dysphagia in patients with Alzheimer's disease: study protocol for a randomized controlled trial.
Rivastigmine improves dual-task gait velocity in patients with Alzheimer's disease.
Specific cognitive domains and frailty trajectories among older Taiwanese adults.
Leisure Time Physical Activities' Association With Cognition and Dementia: A 19 Years' Life Course Study.
What Muscle Signals Mediate the Beneficial Effects of Exercise on Cognition?
Age Moderates Differences in Performance on the Instrumented Timed Up and Go Test Between People With Dementia and Their Informal Caregivers.
The Multi-Domain Intervention Trial in Older Adults With Diabetes Mellitus for Prevention of Dementia in Japan: Study Protocol for a Multi-Center, Randomized, 18-Month Controlled Trial.
Associations of the Lifestyle for Brain Health Index With Structural Brain Changes and Cognition: Results From the Maastricht Study.
A New Hospital-At-Home Model for Integrated Geriatric Care: Data from a Preliminary Italian Experience.
Age-related changes in gait domains: Results from the LonGenity study.
Measurement Practice of Slow Gait Speed for Motoric Cognitive Risk Syndrome: A Systematic Review.
Characterizing the Individuals Who Regain or Maintain Walking Ability after a Hip Fracture: Insights Into Physical Resilience.
Examining the cognitive benefits of probiotic supplementation in physically active older adults: A randomized clinical trial.
The effect of a multimodal dementia prevention program involving community‚Äêdwelling elderly.
Genetically identical twins show comparable tau PET load and spatial distribution.

A feasibility study of the impact of a communication-skills course, 'Empowered Conversations', for care partners of people living with dementia.
Differential Effects of and Modifiable Risk Factors on Hippocampal Volume Loss and Memory Decline in AŒ≤- and AŒ≤+ Older Adults.
Moderate-Intensity Physical Activity, Music and Art Activities Preserved Cognitive Health in Older Adults: An Argument for Social Prescribing Solution.
Total Sedentary Time and Cognitive Function in Middle-Aged and Older Adults: A Systematic Review and Meta-analysis.
Associations of Modifiable Behavioral Risk Factor Combinations at 65 to 74 Years Old With Cognitive Health Span for 20 Years.
Nutrition and exercise interventions could ameliorate age-related cognitive decline: a meta-analysis of randomized controlled trials.
Preliminary study on the benefits of using the robot PALRO¬Æ in facilitating leisure programs for older adults with demen.
Relative Trajectories of Gait and Cognitive Decline in Aging.
Social determinants of memory change: A three-year follow-up of the Canadian Longitudinal Study on Aging (CLSA).
Influence of comorbidities on functional outcomes in patients with surgically treated fragility hip fractures: a retrospective cohort study.
Permanent Deactivation of Inflatable Penile Prosthesis via Puncture.
Physical performance across the cognitive spectrum and between dementia subtypes in a population-based sample of older adults: The HUNT study.
Association of Physical Activity Level With Risk of Dementia in a Nationwide Cohort in Korea.
Repetitive Transcranial Magnetic Stimulation and Tai Chi Chuan for Older Adults With Sleep Disorders and Mild Cognitive Impairment: A Randomized Clinical Trial.
Association Between Psychological Distress and Incident Dementia in a Population-Based Cohort in Finland.
Factors Affecting Quality of Life at Discharge among Patients with Osteoporotic Vertebral Fractures.
Factors Associated With Discharge Destination in Community-Dwelling Adults Admitted to Acute General Medical Units.
Gait Speed and Evolution of Verbal Fluencies in Frail or Prefrail Older People with Type 2 Diabetes. A Pilot Study.
Sarcopenia and motoric cognitive risk syndrome: a moderated mediation model.
Cognitive, physical and disability trajectories in community-dwelling elderly people.
Influence of Physical Activity Levels and Functional Capacity on Brain Œ≤-Amyloid Deposition in Older Women.
Leisure-Time and Non‚ÄìLeisure-Time Physical Activities are Dose-Dependently Associated With a Reduced Risk of Dementia in Community-Dwelling People Aged 40-74¬†Years: The Murakami Cohort Study.
Constricting Life Space and Likelihood of Neurodegenerative Disease in Community-Dwelling Older Men.
Association Between Physical Activity and Risk of Disabling Dementia in Japan.
Fall prevention in community-dwelling adults with mild to moderate cognitive impairment: a systematic review and meta-analysis.
Effects of a staged integral art-based cognitive intervention (SIACI) program in older adults with cognitive impairments: protocol for a randomized controlled trial.
Free-Living Standing Activity as Assessed by Seismic Accelerometers and Cognitive Function in Community-Dwelling Older Adults: The MIND Trial.
Apolipoprotein E4 Moderates the Association Between Vascular Risk Factors and Brain Pathology.
Feasibility of improving strength and functioning and decreasing the risk of falls in older adults with Alzheimer's dementia: a randomized controlled home-based exercise trial.
Learning to play golf for elderly people with subjective memory complaints: feasibility of a single-blinded randomized pilot trial.
Association of Combined Slow Gait and Low Activity Fragmentation With Later Onset of Cognitive Impairment.
How are people with mild cognitive impairment or subjective memory complaints managed in primary care? A systematic review.
Self-perceived functional ability and performance-based testing of physical function in older women with or without long-term back pain - results of the H70 study.
Alcohol consumption and sleep quality: a community-based study.
The Nordic prudent diet prolongs survival with good mental and physical functioning among older adults: The role of healthy lifestyle.
Slow Gait, Subjective Cognitive Decline and Motoric Cognitive Risk Syndrome: Prevalence and Associated Factors in Community Dwelling Older Adults.
Longitudinal Association of Total Tau Concentrations and Physical Activity With Cognitive Decline in a Population Sample.
Midlife Cardiovascular Fitness Is Reflected in the Brain's White Matter.
Effects of combined training on neuropsychiatric symptoms and quality of life in patients with cognitive decline.
Taiwanese Vegetarians Are Associated with Lower Dementia Risk: A Prospective Cohort Study.
Late-onset Cognitive Impairment and Modifiable Risk Factors in Adult Childhood Cancer Survivors.
PREVALENCE AND CLINICAL CORRELATES OF FRAILTY IN HYPERTENSIVE OLDER ADULTS.
Diabetes and Dementia: Spotlight on Multidomain Lifestyle Strategies.
Behavioural change, weight loss and risk of dementia: A longitudinal study.
Two Decades of the Walking While Talking Test: A Narrative Review.
Polygenic Score For Physical Activity Provides Odds For Multiple Common Diseases...2021 ACSM Annual Meeting & World Congresses [Virtual], June 1-5, 2021
Exploration of Cognitive Outcomes and Risk Factors for Cognitive Decline Shared by Couples.
Effects of Exercise Alone or Combined With Cognitive Training and Vitamin D Supplementation to Improve Cognition in Adults With Mild Cognitive Impairment: A Randomized Clinical Trial.
Computer use and cognitive decline among Japanese older adults: A prospective cohort study.
Neighborhood built environment associated with cognition and dementia risk among older adults: A systematic literature review.
Reducing falls through a multifactorial intervention in frail older adults...21st International Conference on Integrated Care (Virtual), May 1-31, 2021.

Patterns and risk factors of cognitive decline among community-dwelling older adults in South Korea.
Analysing Realistic effects of modifiable risk factors for dementia in a large National Dataset.
Cardiorespiratory Fitness Mitigates Age-Related Brain Atrophy And Cognitive Decline In Preclinical Alzheimer's Disease...2021 ACSM Annual Meeting & World Congresses [Virtual], June 1-5, 2021.
Life-Space Mobility in Aged Care Residents: Frailty In Residential Sector over Time (FIRST) Study Findings.
Motor Dysfunction in REM Sleep Behavior Disorder: A Rehabilitation Framework for Prodromal Synucleinopathy.
Analysis of Clinical Traits Associated With Cardiovascular Health, Genomic Profiles, and Neuroimaging Markers of Brain Health in Adults Without Stroke or Dementia.
Physical activity trajectories, autonomic balance and cognitive function: The Coronary Artery Risk Development in Young Adults (CARDIA) study.
SUPERBRAIN (The South Korean study to PrEvent cognitive impaiRment and protect BRAIN health through lifestyle intervention in at-risk elderly people.) : A randomized controlled feasibility trial...International Society for Gerontechnology 13th World Conference, October 22-26, 2022, Daegu, South K...
Designing smart display table for senior care service based on service design research...International Society for Gerontechnology 13th World Conference, October 22-26, 2022, Daegu, South Korea.
Association Of Vigorous-intensity Physical Activity With The Risk Of Dementia...2021 ACSM Annual Meeting & World Congresses [Virtual], June 1-5, 2021.
Promoting Successful Cognitive Aging: A Ten-Year Update.
Three contactless sleep technologies compared with actigraphy and polysomnography in a heterogeneous group of older men and women in a model of mild sleep disturbance: Sleep laboratory study.
Statins, enzyme CoQ,ÇÅ,ÇÄ supplement use, and cognitive functioning.
Evaluation of novel data-driven metrics of amyloid Œ≤ deposition for longitudinal PET studies.
Investigating the neurological underpinnings of accelerated cognitive decline in a rodent model of Alzheimer,Äôs disease following ethanol exposure in adolescence.
No association between age beginning tackle football, or years played and neurocognitive performance later-in-life among older National Football League retirees.
Gender/sex differences in the association of mild behavioral impairment with cognitive aging.
Mitochondrial dysfunction correlates with brain amyloid binding, memory, and executive function in Down syndrome: Implications for Alzheimer,Äôs disease in Down syndrome.
Exp√©rience d,Äôune unit√© pilote sp√©cialis√©e en soins m√©dicaux et de r√©adaptation d√©di√©e aux patients souffrant d,Äôune maladie neuro-√©volutive au d√©cours d,Äôune d√©compensation somatique aigu√´. [Rehabilitation after intercurrent morbid event of patients suffering from neurocognitive disorders in a pilo...
Social determinants of health and mild cognitive impairment in a diverse community sample.
Association of changes in C-reactive protein level trajectories through early adulthood with cognitive function at midlife: The CARDIA study.
Associations between major depressive disorders and Parkinson's Disease and impact of their comorbidity sequence.
Phonemic restoration in Alzheimer,Äôs disease and semantic dementia: A preliminary investigation.
Brain volume change following anti-amyloid Œ≤ immunotherapy for Alzheimer's disease: Amyloid-removal-related pseudo-atrophy.
Nutrition as a modifiable risk factor for cognitive decline: Associated cognitive and physical health changes.
Cognitive function and its longitudinal predictability by intensity of physical activity in Chinese middle-aged and older adults.
Cannabidiol and its potential evidence-based psychiatric benefits ,Äì A critical review.
Fractional amplitude of low-frequency fluctuations in right dorsal cingulum bundle associated with depression symptoms in AD patients: Effects of donepezil intervention.
Exploration of acupuncture therapy in the treatment of mild cognitive impairment based on the brain-gut axis theory.
Insulin homeostasis mediates the relationship between cardiorespiratory fitness and cognitive speed in aging adults.
Adiponectin, leptin, and resistin and the risk of dementia.
Diet quality and nutritional status variability predictors for neurodegenerative diseases and cognitive decline among older adults.
Predicting the reversion from mild cognitive impairment to normal cognition based on magnetic resonance imaging, clinical, and neuropsychological examinations.
Association of physical activity, sedentary behavior, cardiorespiratory fitness and body mass index with brain health.
Hearing loss and its relation to longitudinal changes in white matter microstructure in older adults: The Rotterdam Study.
Physical performance and amyloid-Œ≤ in humans: A systematic review and meta-analysis of observational studies.
Impact of the COVID-19 pandemic and lockdown on anxiety, depression and nursing burden of caregivers in Alzheimer's disease, Dementia with Lewy Bodies and mild cognitive impairment in China: A 1-year follow-up study.
Motoric cognitive risk syndrome among Chinese older adults with white matter lesions: A cross-sectional observational study.
Association of social support with mild cognitive impairment and dementia among older women: The women,Äôs health initiative memory study.
Pharmacological and nonpharmacological approaches to reduce disinhibited behaviors in dementia: A systematic review.
Air pollution and the risk of dementia: The Rotterdam study.
Plasma biomarkers for predicting the development of dementia in a community,Äêdwelling older Japanese population.
Association of cardiovascular events with spouse,Äôs subsequent dementia.
Neurodegeneration in the cortical sulcus is a feature of chronic traumatic encephalopathy and associated with repetitive head impacts.
Study on the effect of 40 Hz non-invasive light therapy system. A protocol for a randomized, double-blinded, placebo-controlled clinical trial.
Correlates of physical activity and sedentary behavior among cancer survivors and cancer-free women: The Women,Äôs Health Accelerometry Collaboration.
Genetically predicted social connections and risk of cognitive impairment: A Mendelian randomization study.

Sex-specific decline in prefrontal cortex mitochondrial bioenergetics in aging baboons correlates with walking speed.
Self-rated health and incident dementia over two decades: Replication across two cohorts.
Physical activity is associated with lower long-term incidence of anxiety in a population-based, large-scale study.
Association between sedentary time and cognitive function: A focus on different domains of sedentary behavior.
Is prosocial behaviour a missing link between Green space quality and child health-related outcomes?
Subtle changes in medication-taking are associated with incident mild cognitive impairment.
Gut microbiome predicts cognitive function and depressive symptoms in late life.
Pilot feasibility study of cognitive training exercises for Egyptian adults: Proof of concept.
Neighborhood physical activity facilities predict risk of incident mixed and vascular dementia: The cardiovascular health cognition study.
Association between physical exercise, executive function, and cerebellar cortex: A cross-sectional study among the elderly in Chinese communities.
Risk factors for young-onset dementia in the UK Biobank.
Association between metabolic syndrome and risk of incident dementia in UK Biobank.
Decoding expectation and surprise in dementia: The paradigm of music.
Association of systolic and diastolic blood pressure with all-cause mortality among community-dwelling older adults: A prospective observational study.
Brain metabolites are associated with sleep architecture and cognitive functioning in older adults.
Neighbourhood walkability in relation to cognitive functioning in patients with disorders along the heart-brain axis.
BrainAGE, brain health, and mental disorders: A systematic review.
Indicators of improvement in performing activities of daily living among older patients undergoing rehabilitation following hip fractures.
TaiChi-MSS protocol: Enhancing cognitive and brain function in MCI patients through Tai Chi exercise combined with multisensory stimulation.
Incidence of memory complaints during the COVID-19 pandemic in Southern Brazil: Findings from PAMPA cohort.
Balance, strength, and risk of dementia: Findings from the health and retirement study and the English Longitudinal Study of Ageing.
Exploration of the mechanism underlying the association of incident microinfarct and motor deficit: A preliminary functional MRI study.
Unhealthy foods may attenuate the beneficial relation of a Mediterranean diet to cognitive decline.
Performance under fire: Older adult cognitive risks and protections under heat strain.
Exploring factors influencing willingness of older adults to use assistive technologies: Evidence from the Cognitive Function and Ageing Study II.
Distinct white matter abnormalities and cognitive impairments in deficit schizophrenia: A cross-sectional diffusion tensor imaging study.
Increased [18F]fluorodeoxyglucose uptake in the left pallidum in military veterans with blast-related mild traumatic brain injury: Potential as an imaging biomarker and mediation with executive dysfunction and cognitive impairment.
Disparities in depression among Chinese older adults with neurodegenerative diseases.
Patient-reported outcomes in multiple sclerosis: A prospective registry cohort study.
Development of fidelity of delivery and enactment measures for interventions in communication disorders.
Prevalence, incidence and modifiable factors for subtypes of mild cognitive impairment: Results from the Longitudinal Ageing Study in China.
Association between use of sodium-glucose co-transporter-2 (SGLT2) inhibitors and cognitive function in a longitudinal study of patients with type 2 diabetes.
Better subjective sleep quality partly explains the association between self-reported physical activity and better cognitive function.
Online chair yoga and digital learning for rural underserved older adults at risk for alzheimer‚Äôs disease and related dementias.
The effectiveness of community aging care centers on global function for people living with dementia.
Associations between head injury and subsequent risk of falls: Results from the Atherosclerosis Risk in Communities (ARIC) study.
Joint effect of polysocial risk score, lifestyle and genetic susceptibility with the risk of dementia: A prospective cohort study.
No associations between glucosamine supplementation and dementia or Parkinson‚Äôs disease: Findings from a large prospective cohort study.
Life-space mobility: Normative values from a national cohort of U.S. older adults.
The association between reallocations of time and health using compositional data analysis: A systematic scoping review with an interactive data exploration interface.
Do cardiovascular risk factors and cardiovascular disease explain sex differences in cognitive functioning in old age?
Glymphatic system dysfunction in middle-aged and elderly chronic insomnia patients with cognitive impairment evidenced by diffusion tensor imaging along the perivascular space (DTI-ALPS).
An 18‚Äêmonth multimodal intervention trial for preventing dementia: J‚Äêmint prime tamba.
Slowing cognitive decline in major depressive disorder and mild cognitive impairment: A randomized clinical trial.
Age-dependent phenotypes of cognitive impairment as sequelae of SARS-CoV-2 infection.
Sense of purpose in life and beliefs and knowledge of Alzheimer‚Äôs disease.
Racial and ethnic differences in the population-attributable fractions of Alzheimer disease and related dementias.
Effects of a physical activity program that incorporates exercises targeting balance, strength, and proprioception on cognitive functions and physical performance in old adults with mild cognitive impairment.
Searching for responders to multidomain dementia prevention in late life: A pooled analysis of individual participant data from the MAPT and preDIVA trials.
APOE genotype disclosure influences decisions about future planning but not adoption of healthy lifestyle changes in cognitively unimpaired individuals.
The association between non-communicable disease and hearing aid adoption in older adults with hearing loss.
Utilization of dental care and the incidence of dementia: A longitudinal study of an older Japanese cohort.

Association of previous concussion with hippocampal volume and symptoms in collegiate-aged athletes.
Effects of a 6-month multifaceted diet and exercise intervention on cognition in older adults at risk of cognitive decline: The PONDER double-blind, placebo-controlled randomized trial.
Fear of falling impairs spatiotemporal gait parameters, mobility, and quality of life in Parkinson's disease: A cross-sectional study.
Catecholamine dysregulation in former American football players: Findings from the DIAGNOSE CTE research project.
Hypothesis: Dementia diminishes interdependence in health and quality of life among spousal partners.
Longitudinal changes in clinical symptoms and proposed biomarkers for prodromal dementia with Lewy bodies in an older patient with depression.
Response to ,ÄúDifficulties in diagnosing phenoconversion to dementia in psychiatric,Äêonset prodromal dementia with Lewy bodies,Äù.
Exploring the contribution of myelin content in normal appearing white matter to cognitive outcomes in cerebral small vessel disease.
Association between hospital-treated infectious diseases and risk of neurodegenerative disease among patients with prediabetes and diabetes: A prospective cohort study in UK Biobank.
Regional brain structural alterations in reward and salience networks in asthma.
The Healthy Brain Initiative (HBI): A prospective cohort study protocol.
Efficacy of non,Äêpharmacological interventions to reduce pain in people with dementia: A systematic review and meta,Äêanalysis.
Long,Äêterm outcomes after rehabilitation in Medicare Advantage and fee,Äêfor,Äêservice beneficiaries.
Implementing a real,Äêworld dementia care training program for nursing assistants in the acute care setting.
Unveiling the link between physical activity levels and dementia risk: Insights from the UK Biobank study.
Changes in executive function in the Canadian longitudinal study on aging over 3-years: A focus on social determinants of health.
The risk of developing dementia in the COVID,Äê19 pandemic; a cohort study.
Associations and correlates of general versus specific successful ageing components.
Mediation of the association between APOE Œµ4 genotype, cognition, and dementia by neuropathology imaging markers in the Rotterdam study.
Primitive reflexes and dementia in older adults: A meta,Äêanalysis of observational and cohort studies.
Geriatric conditions and healthcare utilization among older adults living in subsidized housing.
Comment on ,ÄúPlasma leptin is associated with amyloid CSF biomarkers and Alzheimer,Äôs disease diagnosis in cognitively impaired patients,Äù.
Increase in blood pressure precedes distress behavior in nursing home residents with dementia.
Self-rated health in old age, related factors and survival: A 20-year longitudinal study within the Silver-MONICA cohort.
FAST functional connectivity implicates P300 connectivity in working memory deficits in Alzheimer,Äôs disease.
The architecture of abnormal reward behaviour in dementia: Multimodal hedonic phenotypes and brain substrate.
Ten,Äêyear outcomes of anticholinergic use among older adults with intellectual disability: Findings from the intellectual disability supplement to the irish longitudinal study on ageing (ids,Äêtilda).
Individualization of mhealth interventions for children and adolescents.
Impact of apolipoprotein E4 on the locus coeruleus functional connectivity in preclinical Alzheimer,Äôs disease.
Development and validation of a convenient dementia risk prediction tool for diabetic population: A large and longitudinal machine learning cohort study.
Association between a healthy lifestyle and dementia in older adults with obesity: A prospective study in the UK biobank.
Blood mRNA expression in Alzheimer's disease and dementia with lewy bodies.
Poor oral health and dementia risk under time,Äêvarying confounding: A cohort study based on marginal structural models.
Circulating cell-free genomic DNA is associated with an increased risk of dementia and with change in cognitive and physical function.
Patterns of limitation in physical function in late midlife associated with late-onset Alzheimer,Äôs disease and related dementias: A cluster analysis.
Objective sleep quality and the underlying functional neural correlates among older adults with possible mild cognitive impairment.
A cross,Äêsectional study of potentially modifiable risk factors for dementia and cognitive function in India: A secondary analysis of 10/66, LASI, and SAGE data.
Everyday life meaningfulness for the community-dwelling oldest old during the COVID-19 pandemic.
Activity of choline alphoscerate on adult-onset cognitive dysfunctions: A systematic review and meta-analysis.
Effect of early menopause on cognition and brain morphology in an urban Indian cohort.
Correlates of memory and executive function in middle-aged and older adults in the CLSA: A minority stress approach.
Life's essential 8 and poor brain health outcomes in middle-aged adults.
Pain and associated factors in nursing home residents.
Menopausal hormone therapy is associated with worse levels of Alzheimer's disease biomarkers in APOE Œµ4,Äêcarrying women: An observational study.
Association between subclinical thyroid dysfunction and cognitive decline: Findings from the ELSA-Brasil study.
Progression along data-driven disease timelines is predictive of Alzheimer,Äôs disease in a population-based cohort.
Long-term neuropsychiatric complications and ¬π,Å∏F-FDG-PET hypometabolism in the brain from prolonged infection of COVID-19.
Diagnostic accuracy of the Montreal Cognitive Assessment in screening for cognitive impairment in initially hospitalized COVID-19 patients: Findings from the prospective multicenter NeNeSCo study.
Blood biomarkers to detect Alzheimer disease,ÄîReply.
Loneliness, not social support, is associated with cognitive decline and dementia across two longitudinal population-based cohorts.
Depression and agitation factors are related to regional brain atrophy and faster longitudinal cognitive decline in mild cognitive impairment.
Short communication: Lifetime musical activity and resting-state functional connectivity in cognitive networks.

Interaction between arteriosclerosis and amyloid-Œ≤ on cognitive function.
Can physical activity attenuate the impact of cardiovascular risk factors in the incidence of dementia? Findings from a population-based cohort study.
The impact of physical exercise on hippocampal atrophy in mild cognitive impairment and Alzheimer‚Äôs disease: A meta-analysis.
Adherence to dietary guidelines and risk of dementia: A prospective cohort study of 94 184 individuals.
Sleep quality during and after severe acute respiratory syndrome coronavirus 2 (COVID‚Äê19) lockdowns in the UK: Results from the sleepquest study.
Critical periods for cognitive reserve building activities for late life global cognition and cognitive decline: The Sydney memory and aging cohort study.
Modifiable dementia risk factors associated with objective and subjective cognition.
Longitudinal associations of anticholinergic medications on cognition and possible mitigating role of physical activity.
Short-term medication effect on fall risk in multimorbid inpatients with dementia.
Post-acute COVID-19 neuropsychiatric symptoms are not associated with ongoing nervous system injury.
A culturally and linguistically adapted text-message Diabetes Prevention Program for Latinos: Feasibility, acceptability, and preliminary effectiveness.
Hippocampal-subfield macro- and microstructural changes in cerebral small vessel disease with mild cognitive impairment.
Neuroimaging biomarkers in understanding the relationship between metabolic health and Alzheimer‚Äôs disease.
Long-term impact of a 10-year intensive lifestyle intervention on a deficit accumulation frailty index: Action for Health in Diabetes trial.
Cardiovascular and lifestyle risk factors of mild cognitive impairment in UK veterans and non-veterans.
Physical activity intensity and risk of dementia.
Antidepressant use in relation to dementia risk, cognitive decline, and brain atrophy.
Pragmatic questionnaire-based evaluation of auditory function in individuals with major neurocognitive disorders and hearing loss in diverse contexts.
Effects of mindfulness based interventions in adults and older adults caregivers of patients with early stage Alzheimer‚Äôs disease: A randomized pilot study.
Longitudinal associations between exercise and biomarkers in autosomal dominant alzheimer's disease.
Determinants of cognitive decline in older European men.
Disentangling in-vivo microstructural changes of white and gray matter in mild cognitive impairment and Alzheimer‚Äôs disease: A systematic review and meta-analysis.
Frailty subtypes and white matter alterations in older adults without dementia.
The assessment of the impact of antiepileptic drugs on cognitive functions via N-200/P-300 potentials and neuropsychological measures.
CERAD (Consortium to Establish a Registry for Alzheimer‚Äôs Disease) neuropsychology assessment battery: 35 years and counting.
The association of life stress with subsequent brain and cognitive reserve in middle-aged women.
A song for the mind: A literature review on singing and cognitive health in aging populations.
Time-based and event-based prospective memory in mild cognitive impairment and Alzheimer‚Äôs disease patients: A systematic review and meta-analysis.
Midlife vascular risk factors and risk of incident dementia: Longitudinal cohort and Mendelian randomization analyses in the UK Biobank.
Neighbourhood nursing: Connection, place and meaning in the everyday experience of dementia.
Do physical fitness and cognitive function mediate the relationship between basic activities of daily living and quality of life in older adults with dementia?.
Clinical trajectories preceding incident dementia up to 15 years before diagnosis: A large prospective cohort study.
Reduced social activities and networks, but not social support, are associated with cognitive decline among older Chinese adults: A prospective study.
Pain intensity, physical function, and depressive symptoms associated with discontinuing long‚Äêterm opioid therapy in older adults with Alzheimer's disease and related dementias.
Widespread white matter abnormalities in concussed athletes detected by 7T diffusion magnetic resonance imaging.
Association of type 1 diabetes and hypoglycemic and hyperglycemic events and risk of dementia.
The bidirectional relationship between brain structure and physical activity: A longitudinal analysis in the UK biobank.
South Korean study to prevent cognitive impairment and protect brain health through multidomain interventions via face‚Äêto‚Äêface and video communication platforms in mild cognitive impairment (SUPERBRAIN‚ÄêMEET): A randomized controlled trial.
Comparison of face to face and online delivery of a dementia-specific experiential learning activity.
A multidomain intervention against cognitive decline in an at‚Äêrisk‚Äêpopulation in Germany: Results from the cluster‚Äêrandomized AgeWell.de trial.
Association between chemosensory impairment with neuropsychiatric morbidity in post-acute COVID-19 syndrome: Results from a multidisciplinary cohort study.
Age-related eye diseases and subsequent risk of mental disorders in older adults: A real-world multicenter study.
Feasibility and preliminary impact of a web-based mind body intervention for older dementia caregivers.
Association between dementia, change in home-care use, and depressive symptoms during the COVID-19 pandemic: A longitudinal study using data from three cohort studies.
Does incident cardiovascular disease lead to greater odds of functional and cognitive impairment? Insights from the Health and Retirement Study.
Connecting through caregiving: Reappraising intergeneration relationship in dementia caregiving.
Neuroinflammation, neuron loss, and their contribution to clinical symptoms in chronic traumatic encephalopathy.
Association of psychosocial factors with physical activity among Japanese adults aged 65 and older: A 6-year repeated cross-sectional study from the Nakanojo Study.
Global coherence and cognition in Parkinson‚Äôs disease.
GLP-1RA and SGLT2i medications for type 2 diabetes and Alzheimer disease and related dementias.
Lifestyle, biological, and genetic factors related to brain iron accumulation across adulthood.

Associations of 24‚Äêhour rest‚Äêactivity rhythm fragmentation, cognitive decline, and postmortem locus coeruleus hypopigmentation in Alzheimer's disease.
Post-discharge mortality in patients with delirium and dementia: A 3-year follow up study.
Longitudinal associations between optimism and objective measures of physical functioning in women.
Cognitive function in Mexican older adults 6-months after recovering from SARS-CoV-2 infection.
Low or declining gait speed is associated with risk of developing dementia over 5 years among people aged 85 years and over.
Examining the independent and moderating effects of arterial stiffness and cerebral blood flow on total hippocampal and hippocampal subfield volumes.
Social activity and cognitive decline in older residents of long-term care facilities: A cohort study.
Psychoeducation versus psychoeducation integrated with yoga for family caregivers of people with Alzheimer's disease: A randomized clinical trial.
The mediating role of life course cognitive reserve-enhancing factors in the relationship between adverse childhood experiences and dementia among older adults: Evidence from a prospective cohort study in the united states.
Correlates of the HIV-associated neurocognitive disorders among adults living with HIV in Dodoma region, central Tanzania: A cross-sectional study.
Pharmacological interventions and telomere length in patients with schizophrenia and bipolar disorder: A systematic review and meta-analysis.
Ambiguous loss and disenfranchised grief in formal caregivers of people with dementia: Effectiveness of a training intervention with psychodrama.
The association between psychological resilience and cognitive function in longitudinal data: Results from the community follow-up survey.
Association between plasma L-carnitine and cognitive impairment in patients with acute ischemic stroke.
Low-grade systemic inflammation is associated with domain-specific cognitive performance and cognitive decline in older adults: Data from the TUDA study.
Midlife physical activity engagement is associated with later-life brain health.
Effects of aerobic, resistance, or combined exercise training among older adults with subjective memory complaints: A randomized controlled trial.
Effects of western diet or exercise exposure in a novel rat model of late-onset Alzheimer‚Äôs disease.
Association between sleep microarchitecture and cognition in obstructive sleep apnea.
Healthy lifestyle and cognition in older adults with common neuropathologies of dementia.
Is the pentagon-copying task more than a cognitive feature? Associations with handgrip strength, gait speed, and frailty in older adults.
Feasibility, usability, and acceptance of ‚ÄúBrain-IT‚Äù‚ÄîA newly developed exergame-based training concept for the secondary prevention of mild neurocognitive disorder: A pilot randomized controlled trial.
Heart failure with comorbid dementia: Association with clinical characteristics and outcomes.
Actigraphy estimated sleep moderates the relationship between physical activity and cognition in older adults.
Associations between regional cerebral blood flow and psychiatric symptoms in dementia with Lewy bodies without parkinsonism.
Links of previous incarceration with geriatric syndromes and chronic health conditions among older adults in the United States.
Freezing of gait is associated with daily activity limitations among individuals with Parkinson‚Äôs disease and mild cognitive impairment.
Contributions of loneliness to cognitive impairment and dementia in older adults are independent of other risk factors and Alzheimer‚Äôs pathology: A narrative review.
Therapeutic singing-induced swallowing exercise for dysphagia in advanced-stage Parkinson‚Äôs disease.
Increased Œ≤‚Äêsite APP cleaving enzyme 1‚Äêmediated insulin receptor cleavage in type 2 diabetes mellitus with cognitive impairment.
Eat more copper?
Dementia arts mapping: Observational methods for documenting impacts of poetry and recreation in care settings.
Low plasma 25-hydroxyvitamin D levels are associated with an increased risk of depressive symptoms in community-dwelling Japanese people aged between 40 and 74 years: The Murakami cohort study.
Sex differences in the symptom network structure of depression: Findings from a nationwide sample of the Spanish adult population.
Physical activity is low before and during hospitalisation: A secondary observational study in older Australian general medical patients.
A randomized controlled trial of Tai Chi Chih or health education for geriatric depression.
Virtual reality-based training has potential to improve dementia knowledge, attitudes, and sense of competence of physical therapists and occupational therapists.
Chronic use of benzodiazepine in older adults and its relationship with dementia: A systematic review and meta-analysis.
Effect of cognitive training on cognitive function in community‚Äêdwelling older people with mild‚Äêto‚Äêmoderate dementia: A single‚Äêblind randomised controlled trial.
Brain artery diameters and risk of dementia and stroke.
Associations of relative intensity of physical activity with incident cardiovascular events and all-cause mortality.
Dyadic associations between conscientiousness facets, health, and health behavior over time.
Facets of conscientiousness and motoric cognitive risk syndrome.
Is there a relationship between ‚Äògetting up and dressed‚Äô and functional and physical outcomes in geriatric rehabilitation inpatients? A quasi-experimental study.
Longitudinal associations of social support and gait speed decline in aging.
Exercise and the risk of mild cognitive impairment: Does the effect depend on vascular factors?
REM sleep without atonia and neurocognitive function in isolated REM sleep behaviour disorder: Cross‚Äêsectional and longitudinal study.
Assessment of gait, balance, and falls in individuals with neurodegenerative diseases.
Gait speed according to cognitive status in older adults: A cross-sectional study in a resource-limited country.
Walking and social reminiscence in gentrifying neighborhoods: Feasibility and impact on cognitive, physical, and mental health among older Black adults in the SHARP study.

Effects of a 12-week aerobic exercise programme on human immunodeficiency virus-Associated neurocognitive disorder in Nigeria.
Linking type and extent of head trauma to cavum septum pellucidum in older adults with and without Alzheimer disease and related dementias.
Repetitive blast injury and CSF Alzheimer disease biomarkers: Navigating a complex narrative.
Dementia in Taiwan.
Risk factors of dementia in a cohort of individuals with mild cognitive impairment in the Italian interceptor project.
A promising subject-level classification model for acute concussion based on cerebrovascular reactivity metrics.
Social, behavioural, and functional characteristics of community,Äêdwelling South Korean adults with moderate and severe cognitive impairment.
Association versus prediction and open question on what sleep,Äìwake factors to target for dementia prevention: Letter to the editor regarding ,ÄúEarlier chronotype in midlife as a predictor of accelerated brain aging: A population-based longitudinal cohort study,Äù by Kim et al. (https://doi-org.libpr...
Longitudinal post-shunt outcomes in idiopathic normal pressure hydrocephalus with and without comorbid Alzheimer,Äôs disease.
Relation of motor impairments to neuropathologic changes of limbic-predominant age-related TDP-43 encephalopathy in older adults.
Data,Äêdriven lifestyle patterns and risk of dementia in older Australian women.
Walking time and genetic predisposition for Alzheimer,Äôs disease: Results from the HELIAD study.
Clinical trials for cognition in Parkinson's disease: Where are we and how can we do better?
Cognitive reserve proxies are associated with age-related cognitive decline,ÄîNot age-related gait speed decline.
The impact of the COVID-19 pandemic and lockdown on mild cognitive impairment, Alzheimer's disease and dementia with Lewy bodies in China: A 1-year follow-up study.
Association between sleep quantity and quality in early adulthood with cognitive function in midlife.
Correlating continuously captured home-based digital biomarkers of daily function with postmortem neurodegenerative neuropathology.
Effects of right unilateral ultra,Äêbrief pulse electroconvulsive therapy in a depressed patient with dementia with Lewy bodies: A longitudinal case report.
Force fluctuations during role-differentiated bimanual movements reflect cognitive impairments in older adults: A cohort sequential study.
Evaluating disparity of subjective cognitive decline between male veterans and non-veterans in the United States using propensity score matching estimation: A behavioral risk factor surveillance system survey cross-sectional study.
Football and dementia: Understanding the link.
Deferiprone in Alzheimer disease: A randomized clinical trial.
Frailty trajectories preceding dementia in the US and UK.
BIOFACE: A prospective study of risk factors, cognition, and biomarkers in a cohort of individuals with early-onset mild cognitive impairment. Study rationale and research protocols.
Midlife financial stress and cognitive and physical impairments in older age: The role of potentially modifying factors.
Vigorous physical activity and cognitive trajectory later in life: Prospective association and interaction by apolipoprotein E e4 in the nurses,Äô health study.
Latent profile analysis of cognition in a non-demented diverse cohort: A focus on modifiable cardiovascular and lifestyle factors.
Plasma biomarkers of Alzheimer,Äôs disease are associated with physical functioning outcomes among cognitively normal adults in the multiethnic HABS-HD cohort.
Consumption of ultra-processed food and cognitive decline among older adults with type-2 diabetes.
Associations of bioavailable serum testosterone with cognitive function in older men: Results from the National Health and Nutrition Examination Survey.
Combinations of physical and cognitive training for subcortical neurodegenerative diseases with physical, cognitive and behavioral symptoms: A systematic review.
Early diagnosis of prodromal dementia with Lewy bodies using clinical history of probable REM sleep behaviour disorder and cardiac ¬π¬≤¬≥I-MIBG scintigraphy in memory clinics.
Correlates of gait speed among older adults from 6 countries: Findings from the COSMIC Collaboration.
A data-driven framework for biomarker discovery applied to optimizing modern clinical and preclinical trials on Alzheimer,Äôs disease.
Association between carotid intima,Äêmedia thickness and cognitive decline differs by race.
Predicting internet use and digital competence among older adults using performance tests of visual, physical, and cognitive functioning: Longitudinal population-based study.
Association of multiple metabolic and cardiovascular markers with the risk of cognitive decline and mortality in adults with Alzheimer,Äôs disease and AD-related dementia or cognitive decline: A prospective cohort study.
What is normal ageing? Heterogeneity in age-related trajectories of hippocampal volume.
The association between dual-task walking and counting responses and cognitive function and disability after severe head injury: A preliminary study.
Longitudinal patterns of systolic blood pressure, diastolic blood pressure, cardiorespiratory fitness, and their association with dementia risk: The HUNT study.
Neuroanatomical and prognostic associations of depression in Parkinson,Äôs disease.
Establishing the feasibility and preliminary efficacy of a health belief model based educational training program on health belief perceptions and dementia,Äêpreventive behaviors in people with type 2 diabetes.
Associations between accelerated forgetting, amyloid deposition and brain atrophy in older adults.
Cognitive and physical declines and falls in older people with and without mild cognitive impairment: A 7-year longitudinal study.
Lifestyle factors counteract the neurodevelopmental impact of genetic risk for accelerated brain aging in adolescence.
Machine learning-based personalized composite score dissects risk and protective factors for cognitive and motor function in older participants.
Cognitive prescriptions for reducing dementia risk factors among Black/African Americans: Feasibility, acceptability, and preliminary efficacy.
Genetics contributes to concomitant pathology and clinical presentation in dementia with Lewy bodies.

Gait and/or balance disturbances associated with Alzheimer's dementia among older adults with amnestic mild cognitive impairment: A longitudinal observational study.
Impact of a multidomain lifestyle intervention on regional spontaneous brain activity.
Videogame and computer intervention effects on older adults' mental rotation performance.
Multidimensional health heterogeneity of Chinese older adults and its determinants.
Motor,Äìcognitive treadmill training with virtual reality in Parkinson,Äôs disease: The effect of training duration.
Association of fruit and vegetable consumption with mild cognitive impairment in low- and middle-income countries.
Use of digital health technology among older adults with cancer in the United States: Findings from a national longitudinal cohort study (2015-2021).
Handgrip strength and cognitive performance in a multiethnic cohort in Singapore.
Modifiable lifestyle factors influencing neurological and psychiatric disorders mediated by structural brain reserve: An observational and Mendelian randomization study.
From physical activity patterns to cognitive status: Development and validation of novel digital biomarkers for cognitive assessment in older adults.
Association of daily sleep duration with the incident dementia by serum soluble TREM2 in a community.
Predicting on-road driving skills, fitness to drive, and prospective accident risk in older drivers and drivers with mild cognitive impairment: The importance of non-cognitive risk factors.
Association of persistent musculoskeletal pain with dementia risk score in adults aged 45 years or older: The China health and retirement longitudinal study.
Profiles of lifestyle health behaviors and cognitive decline in older adults.
Accelerometer,Äêmeasured physical activity timing with incident dementia.
Assessing patterns of delirium symptoms reveals a novel subtype among elective surgical patients with postoperative delirium.
Dementia in people with severe/profound intellectual (and multiple) disabilities, and its natural history.
Dual decline in subjective gait speed and domain-specific cognition is associated with higher risk of incident dementia in older Japanese adults: A 15-year age-specific cohort study.
Associations between physical activity, blood-based biomarkers of neurodegeneration, and cognition in healthy older adults: The MAPT study.
Association between GlycA and cognitive function: Cross-sectional results from the ELSA,ÄîBrasil study.
Physical activity attenuates the risk for dementia associated with aging in older adults with mild cognitive impairment. Findings from a population-based cohort study.
Relative contributions of mixed pathologies to cognitive and functional symptoms in brain donors exposed to repetitive head impacts.
Associations between prior head injury, physical functioning, and frailty in the atherosclerosis risk in communities study.
Systematic review of actigraphy measures associated with behavioral and psychological symptoms of dementia.
Characterising the covariance pattern between lifestyle factors and structural brain measures: A multivariable replication study of two independent ageing cohorts.
Association of combined healthy lifestyle factors with incident dementia in participants with and without multimorbidity: A large population-based prospective cohort study.
Psychological resilience trajectories after the covid-19 pandemic: The role of lifestyle and psychosocial factors in a cohort at increased alzheimer,Äôs disease risk.
Clinical manifestations, fluid changes and neuroimaging alterations in patients with general paresis of the insane.
Post,Äêacute sequelae of SARS-CoV-2 (PASC) in nursing home residents: A retrospective cohort study.
Diagnostic accuracy of heart rate variability as a screening tool for mild neurocognitive disorder.
Mortality risks and causes of death by dementia types in a Japanese cohort with dementia: NCGG-STORIES.
Why might urban tree canopy reduce dementia risk? A causal mediation analysis of 109,688 adults with 11 years of hospital and mortality records.
Age and beta amyloid deposition impact gait speed, stride length, and gait smoothness while transitioning from an even to an uneven walking surface in older adults.
Validation of the traditional Chinese version of the Pittsburgh Fatigability Scale for older adults.
Relationship between social activities and cognitive impairment in Chinese older adults: The mediating effect of depressive symptoms.
Connecting the dots: Microstructural properties of white matter hyperintensities predict longitudinal cognitive changes in ageing.
Study design and methods: U.S. study to protect brain health through lifestyle intervention to reduce risk (U.S. POINTER).
Prevalence and associated factors of cognitive frailty in community,Äêdwelling older adults: Results from the Korean longitudinal study of aging.
Hearing loss, social isolation and depression in participants aged 50 years or over in Tasmania, Australia.
E-mail-based health care in patients with dementia during the pandemic.
Statistical approaches for the analysis of combined health-related factors in association with adult cognitive outcomes: A scoping review.
The effect of exercise on falls in people living with dementia: A systematic review.
Population-based mini-mental state examination norms in adults of Mexican heritage in the Cameron County Hispanic Cohort.
Impact of a short-duration experiential learning activity on DPT students,Äô attitudes toward patients living with a dementia.
Facteurs associ√©s √† l,Äôobservance d,Äôune intervention non m√©dicamenteuse au cours d,Äôune recherche men√©e en √âtablissement d,Äôh√©bergement pour personnes √¢g√©es d√©pendantes (Ehpad): Une analyse secondaire de l,Äô√©tude LEDEN. [Factors predictive of adherence to a non-pharmacological intervention in nursing...
Race, sex, and mid-life changes in brain health: Cardia MRI substudy.
The relationship between participation in leisure activities and incidence of falls in residential aged care.
Dynamics of oral language and speech production through neuromodulation: A systematic review of non-invasive brain stimulation in neurodegeneration.
Independent associations of sleep and physical activity with cognition are mediated by hippocampal microstructure in middle-aged and older adults.

Predictors of cognitive aging profiles over 15 years: A longitudinal population-based study.
Physical activity patterns within dementia care dyads.
A coordinated analysis of bidirectional associations between life satisfaction and cognitive function.
Cardiovascular health and rate of cognitive decline in preclinical dementia: A 12-year population-based study.
Enlarged cavum septum pellucidum as a neuroimaging signature of head impact exposure.
Regional brain volume changes in Hakim,Äôs disease versus Alzheimer,Äôs and mild cognitive impairment.
Correction to ,ÄúCardiovascular health and rate of cognitive decline in preclinical dementia: A 12-year population-based study,Äù by Speh et al. (2024).
Lifestyle and incident dementia: A COSMIC individual participant data meta,Äêanalysis.
Two-year changes in physical activity and concurrent changes in cognitive function in a cohort of adults with metabolic syndrome.
Neuroimaging-based data-driven subtypes of spatiotemporal atrophy due to Parkinson,Äôs disease.
The effect of resistance training for older adults with cognitive frailty: a randomized controlled trial.
Prevalence and co-occurrence of dementia risk factors in Denmark: A nationwide study.
Interventions for preventing falls in older people in care facilities.
Motor features that distinguish isolated REM sleep behavior disorder patients from healthy controls: A systematic review.
Dementia risk reduction in China: Country-specific estimates of modifiable risk factors and population attributable fractions (PAFs).
Effects of a Digital, Person-Centered, Photo-Activity Intervention on the Social Interaction of Nursing Home Residents with Dementia, Their Informal Carers and Formal Carers: An Explorative Randomized Controlled Trial.
Transcranial Pulse Stimulation in Alzheimer's: Long-Term Feasibility and a Multifocal Treatment Approach.
Ten-Year Cognitive Trajectories and Determinants in Chinese Older Adults Without Formal Schooling: Chinese Longitudinal Healthy Longevity Survey 2008-2018.
Sustained Improvement of Cognition, Mood and Plasma Markers Three Years After Metabolic Bariatric Surgery. The BARICO Study.
Association of body shape index and body fat percentage with geriatric assessment outcomes.
Integrative machine learning approach to risk prediction for dementia and Alzheimer's disease.
Relationships among young adult general cognitive ability, midlife physical activity, and early late-life cognitive functioning: A four-decade longitudinal cohort study in men.
Rural-urban differences in lipid abnormalities among middle-aged and older Indians.
Cognitive decline and dementia in chronic widespread pain: A longitudinal population-based study.
Development of a predictive model for long-term care and support eligibility certification associated with dementia-related functional impairments: the IRIDE cohort study.
Examining changes in neurofilament light chain following a behavioral weight loss intervention for adults with multiple sclerosis and obesity.
Development and validation of screening tools for motoric cognitive risk syndrome in community settings.
Modeling the importance of life exposure factors on memory performance in diverse older adults: A machine learning approach.
Association of high-sensitivity cardiac troponin I levels below the sex-specific 99th percentile with late-life dementia: the Perth Longitudinal Study of Ageing Women.
Independent and joint association of accelerometer-measured sedentary behaviour and physical activity with mild cognitive impairment and intrinsic capacity decline among Chinese older adults: study protocol for a cross-sectional study.
Association between marital status and dementia among older adults population: the Chinese longitudinal healthy longevity survey.
Physical activity and blood-based biomarkers of neurodegeneration in community dwelling Australians from ISLAND (Island Study Linking Ageing and Neurodegenerative Disease).
Decision-making involvement and quality of life in people with dementia: the mediating role of psychological needs.
Identifying the optimal combinations of modifiable dementia risk factors to target in multidomain intervention - Three-year longitudinal findings from the Canadian longitudinal study on aging.
Biological Ageing Acceleration and Functional Capacities Across the Lifespan in the INSPIRE-T Cohort.
Data from multi-national aging cohorts show dysregulated metabolic pathways in people with physio-cognitive decline.
The effect of tablet-based multimodal training on cognitive functioning in Alzheimer's disease: A randomized controlled trial.
Association of pulmonary function with the risk of incident Alzheimer's disease: a prospective cohort and Mendelian randomization study.
Chronic pain in mental disorders: An umbrella review of the prevalence, risk factors, and treatments across 957,168 people with mental disorders and 16,606,910 controls.
Associations of handgrip strength, walking times, and dementia among Chinese adults: A cross-sectional analysis from CHARLS.
Are sleep disturbances modifiable risk factors for mild cognitive impairment and dementia? A systematic review of large studies.
Associations of Longitudinal Changes in Blood Biomarkers of Dementia With the Proportion of Genetically Inferred African Ancestry.
Plasma brain-derived tau: analytical and clinical validation of the first commercial immunoassay.
Arterial stiffness moderates the link between NfL and cognition: The IGNITE study.
Association of Physical Exercise With Structural Brain Changes and Cognitive Decline in Patients With Early Parkinson Disease.
The 24-Hour Movement Behavior Composition and the Risk of Dementia.
Trends and forecasts of the prevalence and mortality of Alzheimer's disease and other dementias in China.
Do Predictors of Life Satisfaction Change in the Last Years Leading Up to Retirement? The Case of Job Satisfaction and Leisure Satisfaction.
High estimated pulse-wave velocity is associated with lower brain white matter microstructural integrity twelve years later.

Sensory Difficulty, Social and Physical Activity, and Dementia Risk Among Older Adults.
Dose-Response Association of Handgrip Strength With Alzheimer's Disease: A Longitudinal Study Involving 85,979 Adults.
Plant-Based Diets and Mental and Neurocognitive Health Outcomes: A Systematic Review With Meta-Analysis.
Structured vs Self-Guided Multidomain Lifestyle Interventions for Global Cognitive Function: The US POINTER Randomized Clinical Trial.
Association between dynapenic abdominal obesity and mild cognitive impairment among middle-aged and older community-dwelling adults.
Daily steps and health outcomes in adults: a systematic review and dose-response meta-analysis.
Dementia Moves: protocol for a feasibility study testing a physical rehabilitation program for long-term care residents with moderate to severe dementia.
Effects of maximal speed locomotor training on spatiotemporal gait changes in individuals with chronic stroke: A secondary analysis of a randomized controlled trial.
Associations of proteomic age with mortality and incident chronic diseases in the European Prospective Investigation into Cancer and Nutrition (EPIC).
Predicting mortality risk in Alzheimer's disease using machine learning based on lifestyle and physical activity.
Investigating Syndemic Effects of Air Pollution and Physical Inactivity on Cognitive Decline in Older Adults.
Polygenic prediction of body mass index and obesity through the life course and across ancestries.
Longitudinal relationships between daily activity and hippocampal atrophy in Japanese dwellers.
Population attributable fractions of modifiable dementia risk factors for cognitive impairment-the ELSA-Brasil cohort study.
The Telephysiotherapy for Older People (TOP-UP) program for improving mobility in people receiving aged care: a hybrid type 1 effectiveness-implementation randomised controlled trial.
Associations between life course exposure to ambient air pollution with cognition and later-life brain structure: a population-based study of the 1946 British Birth Cohort.
Role of B vitamins in modulating homocysteine and metabolic pathways linked to brain atrophy: Metabolomics insights from the VITACOG trial.
Double-edged sword effect of Sutterella in neurological disorders: implications for the gut-brain axis and neuroimmune interactions.
Biomarkers for Alzheimer's disease across diverse biological domains: an umbrella review and evidence map.
Associations between night/shift working and late-life brain health.
Trajectories of muscle strength and physical performance preceding dementia in older US and European populations.
Prevalence of cognitive frailty among Chinese older adults: A systematic review and meta-analysis.
Adherence to four dietary indices and the risk of all-cause and cause-specific dementia: Findings from the UK Biobank study.
Exploring causal gut-brain axes in Alzheimer's disease using mediation Mendelian randomization analysis.
Brain amyloid-Œ≤ deposition, severity of subjective cognitive decline and gait speed in cognitively unimpaired oldest-old.
Cardiovascular risk factors are associated with lower posterior-medial network functional connectivity in older adults.
Comparing the incidence rates of dementia: Association of social participation among older adults in Japan.
Habitual physical activity and sedentary behavior among women with and without premenopausal bilateral oophorectomy: an exploratory study.
Do exercise interventions improve functional mobility and balance in Alzheimer's patients? A systematic review.
Minimum physical activities protective against Alzheimer's disease in late life: a systematic review.
Combined effects of physical activity, cognitive activity, and dietary patterns on cognitive impairment in older adults with consideration of APOE genotype: a cohort study.
Differential effects of a multidomain intervention on cognitive decline in older adults with type 2 diabetes according to white matter hyperintensity status: A secondary analysis of the J-MIND-Diabetes.
Effects of Combined Physical and Cognitive Interventions on Older Adults With Dementia: A Systematic Review and Meta-Analysis.
Factors related to blood-based biomarkers for neurodegenerative diseases and their intergenerational associations in the Young Finns Study: a cohort study.
Development and validation of a machine learning model for early screening of high-risk mild cognitive impairment from the multi-cohort data.
Age-dependent remodeling of the sciatic proteome in 5xFAD mice can be attenuated by exercise or donepezil treatment to maintain neuromuscular function.
Subcortical gray matter volumes and 5-year dementia risk in individuals with subjective cognitive decline or mild cognitive impairment: A multi-cohort analysis.
Caffeine Intake and Cognitive Function Among US Adults Aged 60+: Insights From NHANES 2011-2014.
A physically and mentally active lifestyle relates to younger brain and cognitive age.
Enhancing Alzheimer's disease prediction using random forest: A novel framework combining backward feature elimination and ant colony optimization.
Preoperative identification of low-risk patients for major complications after acute hip fracture surgery in geriatric patients: an observational cohort study.
An international multi-cohort investigation of self-reported sleep and future depressive symptoms in older adults.
The Role of Fatty Acid Binding Proteins in Neuropsychiatric Diseases: A Narrative Review.
Bidirectional associations of Physical Activity and Cognitive Function in Midlife Adults: A Longitudinal Analysis across 26 years follow-up.
Impact of Alzheimer's disease on sleep in adults with Down syndrome.
Biomarker evidence of neurodegeneration in mid-life former rugby players.
Brain health concerns in former rugby players: clinical and cognitive phenotypes.
Regular physical activity affects brain activities in old individuals: an observational study.
Intervention Effects of the Holistic Physio-Cognitive Rehabilitation for Alzheimer Disease and Mild Cognitive Impairment.
Determinants of medical costs in patients with and without fractures using Korean Senior Cohort Study.
Amyloid-Beta Pathology and Cognitive Performance in Centenarians.

Physical activity as a resistance or resilience mechanism in Down syndrome Alzheimer's disease.
Value of muscle strength in improving the predictive capability for cognitive decline in older women by established risk factors: A prospective cohort study in the Otassha Study.
Gait variability as a marker of white matter integrity in individuals with Down syndrome.
The CAIDE dementia risk score indicates elevated cognitive risk in late adulthood: a structural and functional neuroimaging study.
The Impact of Chronic Pain on Cognitive Function.
Green tea consumption and dementia risk in community-dwelling Japanese people aged 40-74 years: A 12-year cohort study.
Correlation Between Irisin and Cognitive Functions in Alzheimer Dementia.
Predictors of Fall-Related Injuries in Fallers-A Study in Persons with Cognitive Impairment.
Longitudinal study of body mass index in relation to Alzheimer's disease pathology and symptomatology in Down syndrome.
Genetic mechanisms, brain structures, and peripheral biomarkers mediate the relationship between physical frailty and neuropsychiatric disorders.
Associations of physical activity, sedentary behavior, and sleep patterns with cognitive function among middle-aged and older adults.
Longitudinal associations between self-reported exercise levels and cognition in ADAD.
Implementing innovative technology promoting self-awareness of brain health and self-determination in obtaining a timely dementia diagnosis: protocol for a multimethods, concurrent, two-part observational study.
The effect of aerobic exercise on cerebral perfusion in patients with vascular cognitive impairment, the Excersion-VCI randomised controlled clinical trial.
Dose- and pattern- physical activity is associated with lower risk of dementia.
The association between sugar- and artificially sweetened beverages and risk of Alzheimer's disease: systematic review and dose-response meta-analysis of prospective cohort studies.
A longitudinal study of frailty reversibility through a multi-component dementia prevention program.
Digital Health Literacy and Physical Activity Programme for Improvement of Quality of Life in Caregivers of People with Dementia (CAREFIT): Study Protocol.
Impact of lifestyle factors on quantitative motor and cognitive performance: insights from a longitudinal study on healthy ageing.
Effectiveness of Walking Prescription Using Mobile Health Technology on the Changes in Daily Steps in Older Adults With Cognitive Impairment: Randomized Controlled Study.
The role of Life's Crucial 9 in cardiovascular disease incidence and dynamic transitions to dementia.
Trajectories of Cardiorespiratory Fitness Measured by Metabolic Equivalents and the Risk of Alzheimer's and Related Dementias.
Impact of smoking, diabetes, and exercise on dementia risk in women with osteoporosis: a nationwide cohort study.
Association of Alzheimer's Disease Blood Biomarkers With Sarcopenia Incidence and Progression: A 12-Year Population-Based Study.
Genetic background and multidomain interventions in mild cognitive impairment.
A rapid scoping review on operationalizing cognitive and social activities in research on dementia risk reduction.
Plasma and MRI biomarkers capture neuronal damage in former professional boxers.
Wearables-derived risk score for unintrusive detection of Œ±-synuclein aggregation or dopaminergic deficit.
Dexterity and Bimanual Coordination, Cognitive Function, and Mental and Cognitive Well-Being in People with Young Onset Dementia: A Case-Control Study.
Risk factors for surgical site infection following treatment of proximal femoral fracture: a matched-pair analysis.
Development of a neural network-based risk prediction model for mild cognitive impairment in older adults with functional disability.
Polyphenol Intake and Risk of Dementia and Cognitive Decline in Older Adults: Principal Component Analysis Approach.
Association between Obesity and Cognitive Function in Chinese Older Adults: The Mediating Effects of Sleep Quality and Blood Pressure.
Physical activity may mitigate sleep-related cognitive deficits in older adults: Findings from the IGNITE study.
Sex and gender differences in access, quality of care, and effectiveness of treatment in dementia: a scoping review of studies up to 2024.
Association of motoric cognitive risk syndrome with falls in older adults: findings from three longitudinal studies.
Associations between physical activity and brain structure in a large community cohort.
Apathy is distinct from depression or fatigue and is associated with poor physical health in an older community cohort.
Effect of happiness and positive emotions on dementia.
Menopausal hormone therapy and the female brain: Leveraging neuroimaging and prescription registry data from the UK Biobank cohort.
Comparative efficacy of cognitive training modalities in cognitive impairment: A systematic review and network meta-analysis.
Association Between Dietary Patterns and Cognitive Function in Midlife Adults: The Bogalusa Heart Study.
Functional Recovery Predictors in Hip Fractures: Insights from a Community Clinical Pathway.
Associations Between 10-Year Physical Performance and Activities of Daily Living Trajectories and Physical Behaviors in Older Adults.
Cognitive Impairment-Associated Risk Factors of Parkinson's Disease: A Hospital-Based Study in a Cohort of Upper Egypt Parkinson's Patients.
Association among cardiorespiratory fitness, plasma biomarkers of pathology and astrocyte reactivity, and cognition in autosomal-dominant Alzheimer's disease.
Loss of microstructural integrity in left hemispheric white matter tracts is associated with poorer digits in noise understanding.
Alzheimer's Disease Sequencing Project release 4 whole genome sequencing dataset.
Disparities in lifestyle among community-dwelling older adults with or without mild cognitive impairment: a population-based study in north-western China.
The simple task of lifting five kilograms serves as a predictor of age-related disorders in old adults.
The association between lifestyle factors and mortality in Huntington's disease.
Recent advances in the detection and management of motor dysfunction in Alzheimer's disease.

Evaluating the Accuracy and Reliability of Real-World Digital Mobility Outcomes in Older Adults After Hip Fracture: Cross-Sectional Observational Study.
Dynamic neurocognitive adaptation: A follow-up exposome investigation in aging.
Lifetime walking and Alzheimer's pathology: A longitudinal study in older adults.
Relationship of Alzheimer's Disease and Related Dementias Plasma Biomarkers With Mobility in Cognitively Unimpaired Older Adults.
Self-reported motoric cognitive risk syndrome predicts long-term mortality in older adults.
Targeting brain health in subjective cognitive decline: insights from a multidomain randomized controlled trial.
Effects of self-managed lifestyle behavioral changes on cognitive impairment control in Chinese older adults: a population-based prospective study.
Increased sedentary behavior is associated with neurodegeneration and worse cognition in older adults over a 7-year period despite high levels of physical activity.
Public attitudes toward dementia risk prediction: A mixed-methods study.
Rationale and protocol of the LEAD 2.0 study: a randomized controlled trial assessing the feasibility of a virtually delivered 6-month exercise and nutrition intervention in older adults with subjective cognitive decline (SCD).
Faecal mucoprotein MUC2 is decreased in multiple sclerosis and is associated with mucin degrading bacteria.
The impact of unpaid caring on cognitive function: a rapid review.
Cognitive Resilience for the Prevention of Mild Cognitive Impairment in Subjective Cognitive Decline: A Monte Carlo Simulation of a Digital Therapeutic Targeting Dementia Risk Factors.
Characterising acute and chronic care needs: insights from the Global Burden of Disease Study 2019.
Reduced Complexity of Pulse Rate Is Associated With Faster Cognitive Decline in Older Adults.
Dementia, Systemic Biomarkers, and Risk of 30-Day Readmission After Pneumonia: A Multi-Center Cohort Study.
Use of posterior probabilities from a long short-term memory network for characterizing dance behavior with multiple accelerometers.
Changes in older persons' lifestyle and perceived health over time and during the COVID-19 pandemic: findings from the extended follow-up of the FINGER randomized controlled trial from 2009 to 2020.
Relationship between physical function at admission and walking ability at discharge in older adults with vertebral compression fractures: an analysis using propensity score matching.
Long-term cumulative physical activity associated with less cognitive decline: Evidence from a 16-year cohort study.
Knowledge and practice of healthy behaviors for dementia and stroke prevention in a United States cohort.
Physical activity changes during midlife link to brain integrity and amyloid burden.
The overlooked trio: sleep duration, sampling time and physical exercise alter levels of olink-assessed blood biomarkers of cardiovascular risk.
Agile Implementation of a Digital Cognitive Assessment for Dementia in Primary Care.
Changes in Clinical Parameters During Low-Frequency Outpatient Pulmonary Rehabilitation for Male Patients With Chronic Obstructive Pulmonary Disease.
Combined mind-body physical exercise, cognitive training, and nurse-led risk factor modification to enhance cognition among older adults with mild cognitive impairment in primary care: a¬†three-arm randomised controlled trial.
Walking reduces the risk of dementia in patients with Parkinson's disease: a longitudinal follow-up study.
Longitudinal investigation of gait and Alzheimer's disease in adults with Down syndrome.
Imaging brain fluid dynamics and waste clearance involving perivascular spaces in cerebral small vessel disease.
Associations of Mid- and Late-Life Physical Activities With New-Onset Depression and Anxiety Among Older Adults.
Association between high plasma p-tau181 level and gait changes in patients with mild cognitive impairment.
Higher dietary vitamin D intake influences brain and mental function in elderly Americans: a cross-sectional analysis.
Methods for measuring interpersonal behavioral and neural synchrony during group music therapy for individuals with dementia and their caregivers: A case series study.
Individual Sleep Problems Are Associated With an Accelerated Decline in Multiple Cognitive Functions in Older Adults.
Longitudinal deterioration of subjective cognitive decline in apolipoprotein Œµ4 carriers and improvement of subjective cognitive decline by multi-domain intervention for prevention of dementia: The cognitive function instrument assessment.
Mild cognitive impairment, Alzheimer's disease dementia, and predictors of driving cessation: A 7-year longitudinal prospective study.
A pilot study investigating the effectiveness, appreciation, and feasibility of a cognitive stimulation program in dementia patients: online versus face-to-face.
Modified Cued Recall Test: Longitudinal Analysis of Test Versions and Item Recall in Adults With Down Syndrome.
Ambulatory blood pressure monitoring, European guideline targets, and cardiovascular outcomes: an individual patient data meta-analysis.
Risk factors for Alzheimer's disease and cognitive function before middle age in a U.S. representative population-based study.
Individual and Institutional Factors Associated With Urinary Incontinence Among Nursing Home Residents: A Multilevel Analysis.
Patterns of Factors in the National Institute on Aging Health Disparities Research Framework Domains and Mild Cognitive Impairment Risk.
Physical activity complexity, cognition, and risk of cognitive impairment and dementia in the Baltimore Longitudinal Study of Aging.
Activity variability: A novel physical activity metric and its association with cognitive impairment.
Directionality between cognitive function and daily physical activity patterns.
Handgrip strength and gait speed relationships with cognitive impairment in Iranian older adults: Birjand longitudinal aging study.
Chronotype as a potential risk factor for cognitive decline: The mediating role of sleep quality and health behaviours in a 10-year follow-up study.
Behavioral and psychological symptoms of dementia and Alzheimer's disease progression in Down syndrome.
Comparisons of four cognitive-frailty measures in predicting dementia and disability.
Mediators of educational differences in dementia risk later in life: evidence from the HUNT study.

Selected social and lifestyle correlates of brain health markers: the Cross-Cohort Collaboration Consortium.
Clinical characteristics of pathological confirmed DLB without parkinsonism.
The relationship between age and physical activity as objectively measured by accelerometers in older adults with and without dementia.
The cost-effectiveness of an online intervention to prevent dementia: Results from the Maintain Your Brain (MYB) randomised controlled trial.
Physical activity and the risk of dementia in end-stage renal disease patients undergoing hemodialysis: a nationwide population-based study.
Marijuana use and subjective cognitive decline in middle-aged and older adults: Analysis of the behavioral risk factor surveillance system survey.
Prevalence and symptoms of incidental meningiomas: a population-based study.
Preoperative Cognitive Optimization and Postoperative Cognitive Outcomes: A Narrative Review.
One-year exercise improves cognition and fitness and decreases vascular stiffness and reactivity to CO2 in amnestic mild cognitive impairment.
Microbiome functional gene pathways predict cognitive performance in older adults with Alzheimer's disease.
Impaired sleep, depressive symptoms, and pain as determinants of physical activity and exercise intervention adherence: an exploratory analysis of a randomized clinical trial.
Effects of Hand Strength and Walking Speed Combined and in Isolation on the Prediction of Cognitive Decline and Dementia in Middle-Aged and Older Adults: A Systematic Review and Meta-Analysis.
Sex-specific modulation of FOLR1 and its cycle enzyme genes in Alzheimer's disease brain regions.
Association of Physical Activity with Dementia Risk in Cancer Survivors: A Korean Nationwide Cohort Study.
DXA and cardiovascular disease in rheumatoid arthritis: A scoping review.
The Clinical Trial Landscape in Autoimmune Encephalitis: Challenges and Opportunities.
Association of impaired olfactory identification with prevalent mild cognitive impairment and regional brain atrophy: the Hisayama Study.
Association between indoor ventilation frequency and low muscle mass among older adults in China: a national cross-sectional research.
Fall risk stratification in older adults: low and not-at-risk status still associated with falls and injuries.
Critical Review of the Methodological Shortcoming of Ambulatory Blood Pressure Monitoring and Cognitive Function Studies.
Œônnovative Health Promotion Strategies: A 6-Month Longitudinal Study on Computerized Cognitive Training for Older Adults with Minor Neurocognitive Disorders.
Time-restricted eating in early-stage Huntington's disease: A 12-week interventional clinical trial protocol.
Longitudinal Association Between Multimorbidity, Participating Activity, and Cognitive Function: Cross-lagged Mediation Analysis.
Multimodal Associations of Modifiable Risk Factors on White Matter Injury: The SOL-INCA-MRI Study (HCHS/SOL).
Accelerometer-derived "weekend warrior" physical activity, sedentary behavior, and risk of dementia.
Associations between white matter hyperintensities and physical activity: A sectional study in UK Biobank participants.
Sociodemographic Factors in Older Adults' Gait Speed Decline: A Gender Disaggregate Growth Curve Analysis of the Ibadan Longitudinal Study of Ageing.
Physical activity and APOE neuropathology score modify the association of age and [(11)C]-PiB-PET amyloid burden in a cohort enriched with risk for Alzheimer's disease.
Older more fit KL-VS heterozygotes have more favorable AD-relevant biomarker profiles.
The impact of cardiorespiratory fitness on Alzheimer's disease biomarkers and their relationships with cognitive decline.
Western Mediterranean diet predicts 9-year changes in episodic memory in an adult lifespan sample of Americans.
The COVID-19 pandemic's effects on cognition and 24-hour movement behaviours: Findings from the CLSA.
Cognitive remediation for patients with late-life schizophrenia: A follow-up pilot study.
Sleep characteristics and brain structure: A systematic review with meta-analysis.
Domain-specific longitudinal associations between brain volume, white matter lesions, and cognitive function changes.
Older Adults with Diabetes in Korea: Latest Clinical and Epidemiologic Trends.
CNM-Au8 in Amyotrophic Lateral Sclerosis: The HEALEY ALS Platform Trial.
Associations of plasma Neurofilament light chain with sarcopenia and physical capacity in patients with Alzheimer's diseases.
Chronic Traumatic Encephalopathy Neuropathologic Change is Infrequent Following Isolated Moderate-Severe Traumatic Brain Injury.
Biomarkers of cellular senescence predict risk of mild cognitive impairment: Results from the lifestyle interventions for elders (LIFE) study.
A dose-response meta-analysis of physical activity and the risk of alzheimer's disease in prospective studies.
Healthy lifestyle habits, educational attainment, and the risk of 45 age-related health and mortality outcomes in the UK: A prospective cohort study.
Prevalence of dementia risk factors in the Oxford Brain Health Clinic.
Post-cardiovascular disease healthy lifestyle, inflammation and metabolic biomarkers, and risk of dementia: a population-based longitudinal study.
Daily physical activity and trajectories of care service use among older adults: the HUNT4 Trondheim 70+ study.
Differences in Long COVID severity by duration of illness, symptom evolution, and vaccination: a longitudinal cohort study from the INSPIRE group.
Long-Term Exposure to Non-Steroidal Anti-Inflammatory Medication in Relation to Dementia Risk.
All are not created equal: Method descriptions in an epidemiology publication differ among media summaries - A case study comparison.
The relationship between levels of physical activity, adherence to the MIND diet, and cognitive impairment in adults aged 65 years or older in Pakistan.
Development and validation of a population-based risk algorithm for premature mortality in Canada: the Premature Mortality Population Risk Tool (PreMPoRT).
Longitudinal Changes of Quantitative Brain Tissue Properties Induced by Balance Training.
Hearing loss, diet, and cognitive decline: interconnections for dementia prevention.

Protocol for an intergenerational randomized controlled trial to enhance physical activity in older adults at risk for Alzheimer's disease.
Associations between moderate-to-vigorous physical activity, p-tau181, and cognition in healthy older adults with memory complaints: a secondary analysis from the MAPT.
Clinical and cost-effectiveness of a home-based health promotion intervention for older people with mild frailty in¬†England: a multicentre, parallel-group, randomised controlled trial.
Longitudinal Proteomic Profiling of Cognition across an Aerobic Exercise Intervention.
Association Between Dietary Variety and Masticatory Behaviors Measured Using Wearable Device Among Community-Dwelling Older Adults in Japan: A Multilevel Meal-by-Meal Analysis.
Feasibility of assessing cognitive impairment via distributed camera network and privacy-preserving edge computing.
Physical activity may protect myelin via modulation of high-density lipoprotein.
Mortality following hip fracture surgery in patients with dementia: a Swedish multiple national register study.
Changing life expectancy in European countries 1990-2021: a subanalysis of causes and risk factors from the Global Burden of Disease Study 2021.
Association of memory function with COVID-19 outcomes in adults aged 50 years and older: Analysis of three prospective cohorts.
Clinical prognostic factors in progressive supranuclear palsy: Implications for clinical trials.
Differential association of ultraprocessed food categories with risk of developing cognitive impairment in middle-aged and older adults in a longitudinal panel study.
Testing the iMplementation Framework fOr behavioral and LIfestyLe interventions in AlZheimer's DiseasE (MOBILIZE) via the ACT randomized controlled trial.
Association of Gait Speed With Cognitive Outcomes in Older Adults With Hypertension: A Secondary SPRINT MIND Analysis.
Behavioral Monitoring in Transient Ischemic Attack and Stroke Patients: Exploratory Micro- and Macrostructural Imaging Insights for Identifying Post-Stroke Depression with Accelerometers in UK Biobank.
Light-Sheet Fluorescence Imaging Reveals Three-Dimensional Amyloid Burden Reduction Following Five Weeks of Swimming Exercise in Alzheimer's Mouse.
Accelerometry-derived features of physical activity, sleep and circadian rhythm relate to non-motor symptoms in individuals with isolated REM sleep behavior disorder.
Long-Term Physical Exercise for Preventing CKD in Older Adults: A Randomized Clinical Trial.
Effectiveness of digital screening tools in detecting cognitive impairment among community-dwelling elderly in Northern China: A large cohort study.
A two-year longitudinal study of the impact of cognitive status and depression on frailty status in older adults following hip fracture.
A systematic review and meta-analysis of the impact of transcranial direct current stimulation on cognitive function in older adults with cognitive impairments: the influence of dosage parameters.
Individual and additive effects of vitamin D, omega-3 and exercise on DNA methylation clocks of biological aging in older adults from the DO-HEALTH trial.
mHealth Intervention for Dementia Prevention through lifestyle Optimisation (MIND-PRO) in a primary care setting: protocol for a randomised controlled trial in people with low SES and/or migration background.
Predictors of Mortality in a Large Cohort of Elders with Essential Tremor.
Middle-age cerebral small vessel disease and cognitive function in later life: a population-based prospective cohort study.
Physical and mental demands of work associated with dementia risk in later life.
Moving beyond the prevalent exposure design for causal inference in dementia research.
Alzheimer's disease may develop from changes in the immune system, cell cycle, and protein processing following alterations in ribosome function.
Characterization of cognitive decline in long-duration type 1 diabetes by cognitive, neuroimaging, and pathological examinations.
Associations of modifiable dementia risk factors with dementia and cognitive decline: evidence from three prospective cohorts.
Assessing Caregiver Burden, Tasks, and Heart Rate Using Wearable Sensors: A Longitudinal Study of Informal Caregivers of Persons With Dementia and Older Adults.
Number of children and dementia risk: a causal mediation analysis using data from the HUNT study linked with national registries in Norway.
Prevalence of Pathogenic Variants and Eligibility Criteria for Genetic Testing in Patients Who Visit a Memory Clinic.
Plasma Proteomic Signatures of Physical Activity Provide Insights into Biological Impacts of Physical Activity and its Protective Role Against Dementia.
Ultra-processed food consumption and risk of dementia and Alzheimer's disease: The Framingham Heart Study.
Enhancing dementia prediction: A 19-year validation of the CAIDE risk score with insulin resistance and APOE Œµ4 integration in a population-based cohort.
Social Connectedness in Older Adults: The Potential of Social Internet Use to Maintain a Strong and Stable Personal Network.
Analysis of Preventive Effect of Bisphosphonate for Osteoporotic Fracture in Patients with Alzheimer's Disease and Patient Mortality.
OAS1: A Protective Mechanism for Alzheimer's Disease? An Exploration of Data and Possible Mechanisms.
Is the freezing index a valid outcome to assess freezing of gait during turning in Parkinson's disease?
The brain structure underlying the nonlinear association between early-life tobacco smoke exposure and the risk for cognitive decline and dementia in adulthood: a large prospective cohort study.
Daily-life walking speed, running duration and bedtime from wrist-worn sensors predict incident dementia: A watch walk - UK biobank study.
Effects of traditional Thai folk dance combined with cognitive stimulation program on behavior and cognition among older adults with cognitive decline: A randomized controlled trial.
Amyloid-related changes in fluency in patients with subjective cognitive decline.
Association of Alzheimer's Disease Polygenic Risk Score with Concussion Severity and Recovery Metrics.
Modifiable risk factors for early- and late-onset dementia using the Korean national health insurance service database.
Subjective motoric cognitive risk syndrome: Preliminary prevalence from an online survey of a German cohort aged 50.
Circulating Endocannabinoids and Cognitive Function in Older Adults.

Longitudinal Trends in Physical Activity Among Older Adults With and Without HIV in Uganda.
Maintaining level of modifiable dementia risk scores is associated with better cognitive outcomes than increasing risk scores: A population-based prospective cohort study.
Brain health in Norwegian female former top-level football players: a protocol for a longitudinal cohort study.
Baseline predictors of cross-sectional and longitudinal performance on the symbol digit modalities test in individuals with multiple sclerosis.
Relationship between social determinants of health and cognitive performance in an older American population: a cross-sectional NHANES study.
Care for older adults living with dementia in the emergency department: a systematic review and meta-synthesis of care partner roles and perspectives.
Development and evaluation of a free e-learning program on dementia risk reduction for the general public: A pre-post study.
Predicting cognitive decline from neuropsychiatric symptoms and Alzheimer's disease biomarkers: A machine learning approach to a population-based data.
Body composition differences in older adults with and without Alzheimer's disease: A systematic review and meta-analysis of observational studies.
"I Aim to Fulfill My Promise": Dementia Caregiving from the Perspective of Spouses and Partners.
Mapping the neural substrate of high dual-task gait cost in older adults across the cognitive spectrum.
Neuropathology-based approach reveals novel Alzheimer's Disease genes and highlights female-specific pathways and causal links to disrupted lipid metabolism: insights into a vicious cycle.
Relationship between physical activity and biomarkers of pathology and neuroinflammation in preclinical autosomal-dominant Alzheimer's disease.
Global consensus on optimal exercise recommendations for enhancing healthy longevity in older adults (ICFSR).
Effects of multidomain lifestyle intervention on frailty among older men and women - a secondary analysis of a randomized clinical trial.
Body understanding measure for pregnancy scale (BUMPS): Psychometric properties and predictive validity with postpartum anxiety, depression and body appreciation among Italian peripartum women.
Dietary changes following a lifestyle-based intervention for dementia risk reduction - results from the AgeWell.de study.
Higher skeletal muscle mitochondrial oxidative capacity is associated with preserved brain structure up to over a decade.
Guidelines for the standardization of pre-analytical variables for salivary biomarker studies in Alzheimer's disease research: An updated review and consensus of the Salivary Biomarkers for Dementia Research Working Group.
Everyday life and boredom of people living with dementia in residential long-term care: a merged methods study.
Diagnostic abnormalities, disease severity and immunotherapy responsiveness in individuals with Down syndrome regression disorder.
Reallocating Time Between 24-h Movement Behaviors for Obesity Management Across the Lifespan: A Pooled Data Meta-Analysis of More Than 9800 Participants from Seven Countries.
Foods, dietary patterns, and risk of vascular dementia: a systematic review.
Individual and joint associations between sleep duration and physical activity with cognitive function: A longitudinal analysis among middle-aged and older adults in China.
Brain change trajectories in healthy adults correlate with Alzheimer's related genetic variation and memory decline across life.
Differences in lean mass and sarcopenia between individuals with Alzheimer's disease and those without dementia: A systematic review and meta-analysis of observational studies.
Meta-Analysis of Transcriptomic Studies of Blood and Six Brain Regions Identifies a Consensus of 15 Cross-Tissue Mechanisms in Alzheimer's Disease and Suggests an Origin of Cross-Study Heterogeneity.
Adherence to Life's Essential 8 is associated with delayed white matter aging.
Objectively Measured Physical Activity Using Wrist-Worn Accelerometers as a Predictor of Incident Alzheimer's Disease in the UK Biobank.
Cognition, Depression, Pain, and Exercise Motives as Predictors of Longitudinal Profiles of Physical Activity During a Seven-Year Follow-Up Among Older Adults.
agcounts: An R Package to Calculate ActiGraph Activity Counts From Portable Accelerometers.
Global, regional, and national progress towards the 2030 global nutrition targets and forecasts to 2050: a systematic analysis for the Global Burden of Disease Study 2021.
Modifiable risk factors for dementia in India: A cross-sectional study revisiting estimates and reassessing prevention potential and priorities.
Optimizing treatment of cardiovascular risk factors in cerebral small vessel disease using genetics.
Transethnic analysis identifies SORL1 variants and haplotypes protective against Alzheimer's disease.
Associations of accelerometer-measured physical activity, sedentary behaviour, and sleep with next-day cognitive performance in older adults: a micro-longitudinal study.
Time-restricted eating for prevention of age-related vascular cognitive decline in older adults: A protocol for a single-arm open-label interventional trial.
The Impact of Lesser Trochanter Displacement on Hip Flexor Strength Recovery in Patients With Trochanteric Fracture.
Association between composite dietary antioxidant index and cognitive function impairment among the US older adults: a cross-sectional study based on the NHANES 2011-2014.
Dietary inflammatory index scores and cognitive aging: results from the Framingham heart Study offspring cohort.
The burden of diseases, injuries, and risk factors by state in the USA, 1990-2021: a systematic analysis for the Global Burden of Disease Study 2021.
Neuroimaging Correlates of 3 Distinct Physical-Cognitive Phenotypes in Cognitively Normal Older Adults: The Gothenburg H70 Cohort Study.
Association of sarcopenia, ambient air pollution and cognitive function in a community-dwelling middle-aged and elderly Korean population: a community-based cohort study.
Survival of the fittest? Peak oxygen uptake and all-cause mortality among older adults in Norway.
Impact of a national dementia research consortium: The Canadian Consortium on Neurodegeneration in Aging (CCNA).
Association of early life cardiovascular risk factors with grey matter structure in young adults in the United Kingdom: the ALSPAC study.

The African American Dementia and Aging Project: an Oregon-based longitudinal study.
Cognitive frailty and functional disability in older adults: A 10-year prospective cohort study in Japan.
Sex-stratified mortality estimates in people with schizophrenia: A systematic review and meta-analysis of cohort studies of 2,700,825 people with schizophrenia.
Evaluating the Impact of a Daylight-Simulating Luminaire on Mood, Agitation, Rest-Activity Patterns, and Social Well-Being Parameters in a Care Home for People With Dementia: Cohort Study.
Health characteristics and health behaviours in male former contact sports participants: comparison with general population controls in a Finnish cohort study.
Vientiane Multigenerational Birth Cohort Project in Lao People's Democratic Republic: Protocol for Establishing a Longitudinal Multigenerational Birth Cohort to Promote Population Health.
Chronic Traumatic Encephalopathy, Family History of Mental Illness, and Aggression in Brain Donors With Repetitive Head Impact Exposure.
A Simple Physical Examination Predicts Cognitive Decline in Very Mild Dementia.
Neurologic dance training and home exercise improve motor-cognitive dual-task function similarly, but through potentially different mechanisms, among breast cancer survivors with chemotherapy-induced neuropathy: Initial results of a randomized, controlled clinical trial.
Associations Between Physical Activity and Symptoms of Mental Health Disorders in Adolescence: Evidence From the Longitudinal Swedish Twin Register.
Transcriptomic and epigenomic profiling reveals altered responses to diesel emissions in Alzheimer's disease both in vitro and in population-based data.
Timeline to symptomatic Alzheimer's disease in people with Down syndrome as assessed by amyloid-PET and tau-PET: a longitudinal cohort study.
A Multivariable Prediction Model for Mild Cognitive Impairment and Dementia: Algorithm Development and Validation.
World delirium awareness and quality survey in 2023-a worldwide point prevalence study.
The psychosocial effects of the COVID-19 pandemic on Turkish older adults: is there a difference between males and females?
Association of cardiorespiratory fitness with dementia risk across different levels of genetic predisposition: a large community-based longitudinal study.
Association between Cognitive Reserve Indicator and Chronic Disease-Free Survival: A Large Community-Based Longitudinal Study.
Hypothetical Interventions on Cardiovascular Health Metrics for Abnormal Cognitive Aging: An Application of the Parametric g-formula in the CLHLS Cohort Study with 12 Years Follow-Up.
Multidomain Intervention Trial for Preventing Cognitive Decline among Older Adults with Type 2 Diabetes: J-MIND-Diabetes.
Structural and social determinants of health: The multi-ethnic study of atherosclerosis.
Effect of choline alfoscerate in older adult patients with dementia: an observational study from the claims data of national health insurance.
Pain assessment and management in patients with dementia in Taiwan: a best practice implementation project.
Beyond the Heart: The Predictive Role of Coronary Artery Calcium Scoring in Non-Cardiovascular Disease Risk Stratification.
Does Sleep Moderate the Effects of Exercise Training or Complex Mental and Social Activities on Cognitive Function in Adults With Chronic Stroke? Secondary Analysis of a Randomized Trial.
Cerebral hyperactivation across the Alzheimer's disease pathological cascade.
The Temporal Relation of Physical Function with Cognition and the Influence of Brain Health in the Oldest-Old.
The Effect of Levels of Leisure-Time Physical Activity on Cognitive Functions Among Older Adults with Mild Cognitive Impairment: A Longitudinal Analysis.
Association of oral frailty with falls in long-term care residents.
Smaller cingulate grey matter mediates the association between dual-task gait and incident dementia.
Causal cardiovascular risk factors for dementia: insights from observational and genetic studies.
The role of exercise in the prevention and treatment of Alzheimer's disease and mild cognitive impairments.
Incident dementia in ischaemic stroke patients with early cardiac complications: A propensity-score matched cohort study.
Sleep efficiency in community-dwelling persons living with dementia: exploratory analysis using machine learning.
Predicting mid- and late-life dementia risk in primary care: A prognostic study from a national health screening cohort.
White matter integrity and motor function: a link between cerebral myelination and longitudinal changes in gait speed in aging.
Changes in physical activity and all-cause mortality among individuals with dementia: a cohort study using the National Health Insurance Service Database in Korea.
Associations of the 'weekend warrior' physical activity pattern with mild dementia: findings from the Mexico City Prospective Study.
Hospital stays and probable dementia as predictors of relocation to long-term care facilities.
Evidence of the Long-Term Protective Effect of Moderate-Intensity Physical Activity on Cognitive Function in Middle-Aged and Elderly Individuals: A Predictive Analysis of Longitudinal Studies.
Subjective Memory Complaints and the Effect of a Multidomain Lifestyle Intervention on Cognition: The FINGER Trial.
Advances in VNS efficiency and mechanisms of action on cognitive functions.
A comprehensive study of genetic regulation and disease associations of plasma circulatory microRNAs using population-level data.
A novel clinical prediction model for hip fractures: a development and validation study in the total population of Sweden.
Prevalence, aetiology, and service mapping of dementia in rural Uganda. Part of DEPEND Uganda (Dementia Epidemiology, unmet Need and co-Developing Solutions in Uganda).
Biomarkers of endothelial dysfunction and cognition: A two-step IPD meta-analysis.
Motoric cognitive risk syndrome as a predictor of mortality in older male adults with cancer: A prospective cohort study in Peru.
Comorbid health outcomes in patients with schizophrenia: an umbrella review of systematic reviews and meta-analyses.
Alzheimer's Disease and (Phyto) Estrogen Treatment: Modification of Effects by Age, Type of Treatment, and Duration of Use.
The influence of frailty: How the associations between modifiable risk factors and dementia vary.

Specific vegetable types are associated with lower long-term risk for late-life dementia: the Perth Longitudinal Study of Aging Women.
Artificial intelligence-assisted oculo-gait measurements for cognitive impairment in cerebral small vessel disease.
The impact of living environmental factors on cognitive function and mild cognitive impairment: evidence from the Chinese elderly population.
Effects of controlled supramaximal high-intensity interval training on muscle capacities and physical functions for older adults: analysis of secondary outcomes from the Ume√• HIT study-a randomised controlled trial.
What contributes to a decline in cognitive performance among home care clients? Analysis of interRAI data from across Canada.
Investigating the effects of indoor lighting on measures of brain health in older adults: protocol for a cross-over randomized controlled trial.
Long-term breast cancer response to CDK4/6 inhibition defined by TP53-mediated geroconversion.
Impact of Mouthwash-Induced Oral Microbiome Disruption on Alzheimer's Disease Risk: A Perspective Review.
Gait speed, handgrip strength, and their combination, and risk of depression in later life: Evidence from a prospective study of community-dwelling older adults.
The frequency and types of resident relocations in Dutch nursing homes: a nationwide cohort study of electronic health record data.
Later-Life Cognitive Trajectories and Risk of Death: Results from a 6-Year Longitudinal Study of 7082 Chinese.
Diagnostic test accuracy of screening tools for the detection of neurocognitive disorders in older adults post-trauma: A protocol for a systematic review.
The impact of expiratory muscle strength training on voluntary cough effectiveness in Huntington's disease.
The Efficacy of a Home-Based, Augmented Reality Dual-Task Platform for Cognitive-Motor Training in Elderly Patients: A Pilot Observational Study.
Feasibility of a person-centred multidimensional interdisciplinary rehabilitation programme in community-dwelling people with dementia: a randomised controlled pilot trial.
Lipids, lipoproteins, and apolipoproteins: Associations with cognition and dementia.
Mobility Abilities Mediate the Association of a More Active Lifestyle With Mobility Disability in Older Adults.
Exercise Barriers in Older Individuals with Alzheimer's Disease: A Cross-Sectional Study.
Dietary patterns and cardiorespiratory fitness in midlife and subsequent all-cause dementia: findings from the cooper center longitudinal study.
Non-Pharmacological Exercise Randomized Controlled Trials in Alzheimer's Disease.
Artificial intelligence detection of cognitive impairment in older adults during walking.
High-dimensional proteomic analysis for pathophysiological classification of traumatic brain injury.
Associations of socioeconomic status and healthy lifestyle with incident dementia and cognitive decline: two prospective cohort studies.
Effects of Occupational and Leisure-Time Physical Activity on the Risk of Dementia: Results From the Copenhagen City Heart Study.
Cognitive impairment mediates the white matter injury load and gait disorders in subcortical ischemic vascular disease.
Masticatory Function, Sex, and Risk of Dementia Among Older Adults: A Population-Based Cohort Study.
A useful cognitive motor dual task paradigm in prodromal and manifest Huntington's disease.
What have observational studies taught us about brain health? An exploration of select cardiovascular risks and cognitive function.
Safety and efficacy of memantine and trazodone versus placebo for motor neuron disease (MND SMART): stage two interim analysis from the first cycle of a phase 3, multiarm, multistage, randomised, adaptive platform trial.
Caregiving-Related Depression Increases Neuroinflammation in Spousal Caregivers to Individuals With Cognitive Impairment: A Longitudinal Study.
Global, regional, and national burden of stroke and its risk factors, 1990-2021: a systematic analysis for the Global Burden of Disease Study 2021.
Prescribing Trends and Associated Outcomes of Antiepileptic Drugs and Other Psychotropic Medications in US Nursing Homes: Proposal for a Mixed Methods Investigation.
Metabolic Surgery and Chronic Traumatic Encephalopathy: Perceptions of Former NFL Players.
Large-scale exome array summary statistics resources for glycemic traits to aid effector gene prioritization.
Epidemiological Principles in Claims of Causality: An Enquiry into Repetitive¬†Head Impacts (RHI) and Chronic Traumatic Encephalopathy (CTE).
Effects of a nurse-led staged integral art-based cognitive intervention for older adults on the Alzheimer's disease spectrum: A randomized controlled trial.
Transcriptomic profiles in major depressive disorder: the role of immunometabolic and cell-cycle-related pathways in depression with different levels of inflammation.
Integrating traditional Chinese Medicine reduced the risk of hip fracture in patients with dementia.
The Role of Physical Exercise in Cognitive Preservation: A Systematic Review.
Estimating expected years of life lost of psychiatric disorders in Taiwan: A Nationwide cohort study.
Prevalence of cognitive impairment and its associated factors in middle-aged and elderly people in Anhui Province, China: An observational study.
Motoric cognitive risk syndrome as a predictive factor of cognitive impairment and dementia - A systematic review and meta-analysis.
The effect of multi-component exercise intervention in older people with Parkinson's disease and mild cognitive impairment: A randomized controlled study.
Self-supervised learning of wrist-worn daily living accelerometer data improves the automated detection of gait in older adults.
The influence of ageism on the hallmarks of aging: Where age stigma and biology collide.
Total physical activity, plant-based diet and neurodegenerative diseases: A prospective cohort study of the UK biobank.
Plasma Biomarkers of Traumatic Brain Injury in Adolescents With Sport-Related Concussion.
Motor and Nonmotor Measures and Declining Daily Physical Activity in Older Adults.
Plant but not animal sourced nitrate intake is associated with lower dementia-related mortality in the Australian Diabetes, Obesity, and Lifestyle Study.
Characterization and individual-level prediction of cognitive state in the first year after 'mild' stroke.
Association Between Ethylene Oxide Exposure and Cognitive Function in US Older Adults: NHANES 2013-2014.

Characterizing Gut Microbiota in Older Chinese Adults with Cognitive Impairment: A Cross-Sectional Study.
Identification of the Shared Gene Signatures Between Alzheimer's Disease and Diabetes-Associated Cognitive Dysfunction by Bioinformatics Analysis Combined with Biological Experiment.
Slow Gait Speed Predicts Incident Dementia, Mortality, and Long-Term Functional Outcome in Cerebral Small-Vessel Disease.
Gait performance in older adults across the cognitive spectrum: Results from the GAIT cohort.
Association between the Intake/Type of Cheese and Cognitive Function in Community-Dwelling Older Women in Japan: A Cross-Sectional Cohort Study.
Radiographic Knee Osteoarthritis Is a Risk Factor for the Development of Dementia: Locomotive Syndrome and Health Outcomes in the Aizu Cohort Study.
Diabetes, Prediabetes, and Brain Aging: The Role of Healthy Lifestyle.
Leisure activity engagement attenuates the risks of cognitive impairment induced by unhealthy plant-based diets: a nationwide cohort study.
Reliable Contactless Monitoring of Heart Rate, Breathing Rate, and Breathing Disturbance During Sleep in Aging: Digital Health Technology Evaluation Study.
The Associations of Sensory Impairment With 10-Year Risk of Dementia and Alzheimer's Disease: The Health and Retirement Study, 2010-2020.
Sedentary behavior and associated factors on admissions to internal medicine wards.
Tamsulosin use in benign prostatic hyperplasia and risks of Parkinson's disease, Alzheimer's disease and mortality: An observational cohort study of elderly Medicare enrollees.
Process evaluation of a New psychosocial goal-setting and manualised support intervention for Independence in Dementia (NIDUS-Family).
Accelerometer-derived 'weekend warrior' physical activity pattern and brain health.
Assessment of cognitive function in elderly patients with heart failure.
The Importance of Racially and Ethnically Inclusive Gait Speed Reference Values in Individuals 90 Years and Older: LifeAfter90.
Association between "weekend warrior" physical activity and the incidence of neurodegenerative diseases.
Impact of Helicobacter pylori eradication on age-specific risk of incident dementia in patients with peptic ulcer disease: a nationwide population-based cohort study.
Association between frailty and cognitive status among ambulating Korean elderly: An observational study.
Association of Muscle Quantity and Quality With Knee Extension Strength in Knee Osteoarthritis: The Nagahama Study.
Long-Term Effects of the Multicomponent Program BrainProtect(¬Æ) on Cognitive Function: One-Year Follow-Up in Healthy Adults.
Predictors of the onset of low handgrip strength in Europe: a longitudinal study of 42,183 older adults from 15 countries.
Physical activity and cognition: longitudinal findings from the Thinking and Living with Cancer Study.
Effect of home-based and remotely supervised combined exercise and cognitive intervention on older adults with mild cognitive impairment (COGITO): study protocol for a randomised controlled trial.
Cognitive reserve as a modulator of cognitive decline and of behavioral symptoms in patients with amyotrophic lateral sclerosis.
Blood biomarkers for neuroaxonal injury and astrocytic activation in chemotherapy-induced peripheral neuropathy.
Benefits of population-level interventions for dementia risk factors: an economic modelling study for England.
Association of Moderate and Vigorous Physical Activity With Cognitive Performance: Evidence From Brazil.
Associations of Cognitively Active Versus Passive Sedentary Behaviors and Cognition in Older Adults.
Assessing the Effectiveness of eHealth Interventions to Manage Multiple Lifestyle Risk Behaviors Among Older Adults: Systematic Review and Meta-Analysis.
Tracking neuroinflammatory biomarkers in Alzheimer's disease: a strategy for individualized therapeutic approaches?
Mild Cognitive Impairment and Suicidal Ideation Among Adults Aged 65 Years or Older From Low- and Middle-Income Countries.
A lifetime perspective on risk factors for cognitive decline with a special focus on early events.
Causal relationships involving brain imaging-derived phenotypes based on UKB imaging cohort: a review of Mendelian randomization studies.
Female Reproductive Factors and Risk of Mild Cognitive Impairment and Dementia: The HUNT Study.
Gait Parameters Change can be an Early Marker of Cognitive Impairment.
Standardization and clinical applications of retinal imaging biomarkers for cardiovascular disease: a Roadmap from an NHLBI workshop.
The Impact of Misaligned Perceived and Objective Fall Risk in Cognitively Impaired Older People.
Associations of Physical Activity Engagement with Cerebral Amyloid-Œ≤ and Tau from Midlife.
Association between adult education, brain volume and dementia risk: longitudinal cohort study of UK Biobank participants.
Longitudinal relationships between depressive symptoms, functional impairment, and physical activity in later late life.
Predictors of hip fracture in 15 European countries: a longitudinal study of 48,533 geriatric adults using SHARE dataset.
Toward better understanding and management of chemobrain: the potential utilities of the MemTrax memory test.
Midlife Residential Greenness and Late-Life Cognitive Decline among Nurses' Health Study Participants.
Relations Between Cardiorespiratory Fitness and Cognition in Older Adults With Amnestic Mild Cognitive Impairment From the Aerobic Exercise and Cognitive Training (ACT) Trial: Sex Differences.
Differential impacts of fat and muscle mass on cardiovascular and non-cardiovascular mortality in individuals with type 2 diabetes.
Association of triglyceride glucose-body mass index with Alzheimer's disease pathology, cognition and brain structure in non-demented people.
The identification of biomarkers for Alzheimer's disease using a systems biology approach based on lncRNA-circRNA-miRNA-mRNA ceRNA networks.
Association of Anxiety and Unspecified Emotional Distress Obtained from a Medical Records Linkage System with Incident Cognitive Outcomes in a Population-Based Setting.
Comorbid physical health outcomes in patients with bipolar disorder: An umbrella review of systematic reviews and meta-analyses.
Identifying determinants for falls among Iranian older adults: insights from the Bushehr Elderly Health Program.

Geographical variation in dementia prevalence across China: a geospatial analysis.
Associations of ambient air pollution exposure and lifestyle factors with incident dementia in the elderly: A prospective study in the UK Biobank.
Association between alcohol consumption and all-cause mortality, cardiovascular disease, and chronic kidney disease: A prospective cohort study.
Association of physical function with connectivity in the sensorimotor and dorsal attention networks: why examining specific components of physical function matters.
Brain and serum lipidomic profiles implicate Lands cycle acyl chain remodeling association with APOEŒµ4 and mild cognitive impairment.
Discrete Relationships between Spatiotemporal Gait Characteristics and Domain-Specific Neuropsychological Performance in Midlife.
Associations of modifiable and non-modifiable risk factors with cognitive functions - a prospective, population-based, 17 years follow-up study of 3,229 individuals.
The Association of Mitral Annular Calcification With Cardiovascular and Noncardiovascular Outcomes: The Multi-Ethnic Study of Atherosclerosis.
1-Norm waveform analysis for MR elastography-based quantification of inhomogeneity: Effects of the freeze-thaw cycle and Alzheimer's disease.
The impact of six-week dihydrogen-pyrroloquinoline quinone supplementation on mitochondrial biomarkers, brain metabolism, and cognition in elderly individuals with mild cognitive impairment: a randomized controlled trial.
Mediating effects of predictability between caregiving demands and caregiving consequences for persons living with dementia: A longitudinal study.
Association between institutionalization by 4,Äâmonths post-discharge walking capacity and lean body mass in elderly hip fracture patients: Evidence from a Swedish Registry Based Study.
A review of the literature on wellbeing and modifiable dementia risk factors.
Resistance Exercise Reduces Sarcopenia by Repairing Leaky Gut in Patients with Alzheimer's Disease.
Eye Adult Changes in Thought (Eye ACT) Study: Design and Report on the Inaugural Cohort.
Metformin-induced changes in the gut microbiome and plasma metabolome are associated with cognition in men.
Mid-to-Late Life Healthy Lifestyle Modifies Genetic Risk for Longitudinal Cognitive Aging among Asymptomatic Individuals.
Does cardiorespiratory fitness mediate or moderate the association between mid-life physical activity frequency and cognitive function? findings from the 1958 British birth cohort study.
Biomarkers of Neurobiologic Recovery in Adults With Sport-Related Concussion.
Linking Dementia Pathology and Alteration in Brain Activation to Complex Daily Functional Decline During the Preclinical Dementia Stages: Protocol for a Prospective Observational Cohort Study.
High-Intensity Aphasia Intervention Is Minimally Fatiguing in Chronic Aphasia: An Analysis of Participant Self-Ratings From a Large Randomized Controlled Trial.
Shared plasma metabolomic profiles of cognitive and mobility decline predict future dementia.
Real-world use of glucocorticoids and clinical outcomes in adults hospitalized with community-acquired pneumonia on medical wards.
Relationship Between Physical Exercise and Cognitive Function Among Older Adults in China: Cross-Sectional Population-Based Study.
Exploring the in vitro stability of insulin degrading enzyme as a potential biomarker for neurocognitive disorders and Alzheimer's disease risk.
Digital outcome measures from smartwatch data relate to non-motor features of Parkinson's disease.
Exploring the impact of computer game playing on cognitive function, Alzheimer's disease risk, and brain-derived neurotrophic factor levels: Basic evidence from Mendelian randomization.
Association between physical activity, peak expiratory flow, and cognitive function in aging: a cross-sectional analysis.
Higher surface folding of the human premotor cortex is associated with better long-term learning capability.
Associations between Vegetable Nitrate Intake and Cardiovascular Disease Risk and Mortality: A Systematic Review.
The Role of Physical Activity in Adjunctive Nursing Management of Neuro-Degenerative Diseases among Older Adults: A Systematic Review of Interventional Studies.
Alzheimer disease-related biomarkers and cancer-related cognitive decline: the Thinking and Living with Cancer study.
The Efficacy of a Metacognitive Training Program in Amnestic Mild Cognitive Impairment: A 6-Month Follow-Up Clinical Study.
Brain health index as a predictor of possible vascular dementia in the Mexican health and aging study 2012-2015.
Depression and the Risk of Dementia and All-Cause Mortality Among Japanese Older Adults: A 9-Year Longitudinal Study From JAGES.
Apolipoprotein …õ4 Is Associated With Increased Risk of Fall- and Fracture-Related Hospitalization: The Perth Longitudinal Study of Ageing Women.
Interventions to prevent obesity in children aged 5 to 11 years old.
ZOom Delivered Intervention Against Cognitive decline (ZODIAC) COVID-19 pandemic adaptations to the Post-Ischaemic Stroke Cardiovascular Exercise Study (PISCES): protocol for a randomised controlled trial of remotely delivered fitness training for brain health.
Sarcopenia in older patients with idiopathic normal pressure hydrocephalus: an observational study from a single geriatric clinic in Turkey.
Socio-Cognitive Determinants of Lifestyle Behavior in the Context of Dementia Risk Reduction: A Population-Based Study in the Netherlands.
Loneliness predicts decreased physical activity in widowed but not married or unmarried individuals.
The association of ultra-processed food intake with neurodegenerative disorders: a systematic review and dose-response meta-analysis of large-scale cohorts.
Social Determinants of Health, Risk and Resilience against Alzheimer's Disease and Related Dementias: The Healthy Brain Initiative.
Differences in Daily Physical Activity by Alzheimer's Risk Markers Among Older Adults.
Association between loneliness and cognitive function, and brain volume in community-dwelling elderly.
Nonpharmacological intervention therapies for dementia: potential break-even intervention price and savings for selected risk factors in the European healthcare system.
Metformin mitigates dementia risk among individuals with type 2 diabetes.
Intensive antihypertensive treatment does not lower cerebral blood flow or cause orthostatic hypotension in frail older adults.

Meta-analysis on the interrelationship between sarcopenia and mild cognitive impairment, Alzheimer's disease and other forms of dementia.
Association of Rest-Activity Rhythm and Risk of Developing Dementia or Mild Cognitive Impairment in the Middle-Aged and Older Population: Prospective Cohort Study.
Systemic inflammation, lifestyle behaviours and dementia: A 10-year follow-up investigation.
Critical Values of Daily Sedentary Time and Its Longitudinal Association with Mild Cognitive Impairment Considering APOE Œµ4: A Prospective Cohort Study.
Hippocampal aggregation signatures of pathogenic UBQLN2 in amyotrophic lateral sclerosis and frontotemporal dementia.
Neighborhood physical environments and change in cardiometabolic risk factors over 14 years in the study of Women's health across the nation.
Gait and heart rate: do they measure trait or state physical fatigue in people with multiple sclerosis?
[Not Available].
Microbial Indoles: Key Regulators of Organ Growth and Metabolic Function.
Self-Reported Sleep Duration and Bedtime Are Associated with Dementia Risk in Community-Dwelling People Aged 40-74 Years: The Murakami Cohort Study.
Association of midlife body-weight variability and cycles with earlier dementia onset: a nationwide cohort study.
Alzheimer's disease prevention: Apolipoprotein e4 moderates the effect of physical activity on brain beta-amyloid deposition in healthy older adults.
Prospective cohort study of long-term neurological outcomes in retired elite athletes: the Advanced BiomaRker, Advanced Imaging and Neurocognitive (BRAIN) Health Study protocol.
Monitoring risk factors for dementia in middle-aged and older adults: a longitudinal study.
Associations between regular physical exercise and physical, emotional, and cognitive health of older adults in China: an 8-year longitudinal study with propensity score matching.
Longitudinal validation of cognitive reserve proxy measures: a cohort study in a rural Chinese community.
Exercised-enriched blood plasma rescues hippocampal impairments and cognitive deficits in an Alzheimer's disease model.
Barriers and motivators to undertaking physical activity in adults over 70-a systematic review of the quantitative literature.
C/EBPŒ≤: A transcription factor associated with the irreversible progression of Alzheimer's disease.
Molecular mechanisms linking type 2 diabetes mellitus and late-onset Alzheimer's disease: A systematic review and qualitative meta-analysis.
Establishment of a mild cognitive impairment risk model in middle-aged and older adults: a longitudinal study.
Association of Basal Forebrain Volume with Amyloid, Tau, and Cognition in Alzheimer's Disease.
Cross-sectional interactive associations of physical activity and sedentary behaviour with physical capacity across adulthood.
Clinical and radiographic outcomes of shoulder hemiarthroplasty for patients with glenoid medialization.
Growth-Associated Protein¬†43 and Tensor-Based Morphometry Indices in Mild Cognitive Impairment.
Gestational diabetes mellitus is associated with greater incidence of dementia during long-term post-partum follow-up.
Development of a smartphone screening test for preclinical Alzheimer's disease and validation across the dementia continuum.
Food resources and kitchen skills plus aerobic training (FoRKS+) for black adults with hypertension: A pilot trial protocol.
Loneliness Predicts Progression of Frailty in Married and Widowed, but Not Unmarried Community Dwelling Older Adults.
Circulating Lipoproteins Mediate the Association Between Cardiovascular Risk Factors and Cognitive Decline: A Community-Based Cohort Study.
Association of 8-hydroxy-2'-deoxyguanosine with motoric cognitive risk in elderly Chinese people: RUGAO longevity and aging cross-sectional study.
[Effects of a One-Week Inpatient Health Programme for Family Caregivers and Persons in Need of Care (SVLFG Care Tandem) - A Quasi-Experimental Study].
The associations of daytime napping and motoric cognitive risk syndrome: Findings from the China Health and Retirement Longitudinal Study.
Personal value of Alzheimer's disease biomarker testing and result disclosure from the patient and care partner perspective.
Systematic Review of Longitudinal Evidence and Methodologies for Research on Neighborhood Characteristics and Brain Health.
Adherence to the Eatwell Guide and cardiometabolic, cognitive and neuroimaging parameters: an analysis from the PREVENT dementia study.
The time course of motor and cognitive decline in older adults and their associations with brain pathologies: a multicohort study.
Global burden of 288 causes of death and life expectancy decomposition in 204 countries and territories and 811 subnational locations, 1990-2021: a systematic analysis for the Global Burden of Disease Study 2021.
A critical appraisal of blood-based biomarkers for Alzheimer's disease.
ROBot-assisted physical training of older patients during acUte hospitaliSaTion-study protocol for a randomised controlled trial (ROBUST).
Gender differences in the associations of psychosocial trauma and acute medical stressors with immune system activation and dementia risk.
Greater white matter degeneration and lower structural connectivity in non-amnestic vs. amnestic Alzheimer's disease.
Recent insights from non-mammalian models of brain injuries: an emerging literature.
Impaired kidney function, cerebral small vessel disease and cognitive disorders: the Framingham Heart Study.
CSF proteomic profiling with amyloid/tau positivity identifies distinctive sex-different alteration of multiple proteins involved in Alzheimer's disease.
Spirituality for Social Isolation in a Patient with Cognitive Impairment: A Case Report.
Intelligent Alzheimer's Diseases Gene Association Prediction Model Using Deep Regulatory Genomic Neural Networks.
Changes in Brain Structure and Function in a Multisport Cohort of Retired Female and Male Athletes, Many Years after Suffering a Concussion: Implications for Neuroplasticity and Neurodegenerative Disease Pathogenesis.
A Longitudinal Dose-Response Curve Between Leisure-Time Physical Activity and the Prevalence of Diabetes Based on the Different Levels of Cognitive Function Among Older Adults.
Physical Activity Moderates the Relationship between Cardiovascular Disease Risk Burden and Cognition in Older Adults.
Antineoplastics for treating Alzheimer's disease and dementia: Evidence from preclinical and observational studies.

Large-scale animal model study uncovers altered brain pH and lactate levels as a transdiagnostic endophenotype of neuropsychiatric disorders involving cognitive impairment.
Behavioral and Psychological Symptoms of Dementia and Mortality Risk Among People With Cognitive Impairment: An 8-year Longitudinal Study From the NCGG-STORIES.
Role of cardiovascular health factors in mediating social inequalities in the incidence of dementia in the UK: two prospective, population-based cohort studies.
Psychological therapies for depression in older adults residing in long-term care settings.
Characterizing Heart Rate Variability Response to Maximal Exercise Testing in People with Huntington's Disease.
The impact of exercise on blood-based biomarkers of Alzheimer's disease in cognitively unimpaired older adults.
Sex differences in cognitive function trajectories and their determinants in older adults: Evidence from the Chinese longitudinal healthy longevity survey.
Potential role of hippocampal neurogenesis in spinal cord injury induced post-trauma depression.
Global age-sex-specific mortality, life expectancy, and population estimates in 204 countries and territories and 811 subnational locations, 1950-2021, and the impact of the COVID-19 pandemic: a comprehensive demographic analysis for the Global Burden of Disease Study 2021.
Association of Neurotrophic Factors at Midlife With In Vivo Measures of Œ≤-Amyloid and Tau Burden 15 Years Later in Dementia-Free Adults.
Serum soluble alpha-klotho klotho and cognitive functioning in older adults aged 60 and 79: an analysis of cross-sectional data of the National Health and Nutrition Examination Survey 2011 to 2014.
Bone scan findings of Paget's disease of bone in patients with VCP Multisystem Proteinopathy 1.
Pilot Study of a Digital Behavioral Therapy for Overactive Bladder in Women.
Unraveling the mechanisms of NK cell dysfunction in aging and Alzheimer's disease: insights from GWAS and single-cell transcriptomics.
Measurement properties of the backward walk test in people with balance and mobility deficits: A systematic review.
Association Between Serum NT-proBNP and Gray Matter Atrophy Patterns in an Older Japanese Population: The Hisayama Study.
DO ORAL CARE AND REHABILITATION IMPROVE COGNITIVE FUNCTION? A SYSTEMATIC REVIEW OF CLINICAL STUDIES.
Blood levels of glial fibrillary acidic protein for predicting clinical progression to Alzheimer's disease in adults without dementia: a systematic review and meta-analysis protocol.
Patient and Public Involvement in Technology-Related Dementia Research: Scoping Review.
Early-life cumulative exposure to excess bodyweight and midlife cognitive function: longitudinal analysis in three British birth cohorts.
Purpose in Life and Its Association to Parkinsonism.
Visual processing speed and its association with future dementia development in a population-based prospective cohort: EPIC-Norfolk.
Can motor decline be a modifiable marker of clinical progression in subjective cognitive decline? A national prospective cohort study.
Mortality Outcomes in a Large Population with and without Covert Cerebrovascular Disease.
Nutrient Intake and Its Association with Appendicular Total Lean Mass and Muscle Function and Strength in Older Adults: A Population-Based Study.
Neuroimaging abnormalities associated with immunotherapy responsiveness in Down syndrome regression disorder.
Roles of Baseline Intrinsic Capacity and its Subdomains on the Overall Efficacy of Multidomain Intervention in Promoting Healthy Aging among Community-Dwelling Older Adults: Analysis from a Nationwide Cluster-Randomized Controlled Trial.
Associations of carotid atherosclerosis with cognitive function and brain health: Findings from a UK tri-ethnic cohort study (Southall and Brent Revisited).
Day-to-day sleep variability with Alzheimer's biomarkers in at-risk elderly.
Cognition is selectively impaired in males with spinal pain: A retrospective analysis of data from the Longitudinal Study of Ageing Danish Twins.
Plasma metabolites of a healthy lifestyle in relation to mortality and longevity: Four prospective US cohort studies.
Association between dietary vitamin B1 intake and cognitive function among older adults: a cross-sectional study.
Impact of Concurrent Visual and Hearing Impairment on Incident Alzheimer's Disease: The LIFE Study.
Cognitive and immunological effects of yoga compared to memory training in older women at risk for alzheimer's disease.
Quantification of blood glial fibrillary acidic protein using a second-generation microfluidic assay. Validation and comparative analysis with two established assays.
Pre-asthma: a useful concept for prevention and disease-modification? A EUFOREA paper. Part 1-allergic asthma.
[Overview of the Guidelines for Managing Idiopathic Normal Pressure Hydrocephalus].
"Cognitive" Criteria in Older Adults With Slow Gait Speed: Implications for Motoric Cognitive Risk Syndrome.
Early Detection Screening of Cognitive Decline in Patients Over 60 Years: ELDERCARE Study.
Effects of certain pre-analytical factors on the performance of plasma phospho-tau217.
Physical Exercise and Health, 6: Sedentary Time, Independent of Health-Related Physical Activity, as a Risk Factor for Dementia in Older Adults.
Tooth loss mediates the association between smoking and an increased risk of dementia among older adults: The JAGES prospective cohort study.
Knowledge structure and future research trends of body-mind exercise for mild cognitive impairment: a bibliometric analysis.
Study protocol of the Hungarian Longitudinal Study of Healthy Brain Aging (HuBA).
Chronic disease and multimorbidity in the Chinese older adults' population and their impact on daily living ability: a cross-sectional study of the Chinese Longitudinal Healthy Longevity Survey (CLHLS).
The Potential of Chronic Remote Ischemic Conditioning in Cerebral Small Vessel Disease.
Dementia risk factors in former contact sports participants: prospective cohort study.
Cross-sectional associations between 24-hour time-use composition, grey matter volume and cognitive function in healthy older adults.
Validation of ActiGraph and Fitbit in the assessment of energy expenditure in Huntington's disease.
Effects of Multicomponent Exercise on Community-Dwelling Older Adults With Mild Cognitive Impairment.

Investigating the individual and joint effects of socioeconomic status and lifestyle factors on mild cognitive impairment in older Italians living independently in the community: results from the NutBrain study.
Assessing dynamical resilience indicators in older adults: a prospective feasibility cohort study.
Brief webcam test of hand movements predicts episodic memory, executive function, and working memory in a community sample of cognitively asymptomatic older adults.
Predicting the incidence of mild cognitive impairment with a computer-based cognitive assessment tool in community-dwelling older adults: The Otassha study.
Reforming allied health service provision in residential aged care to improve the rehabilitation reach: a feasibility study.
Mobilise-D insights to estimate real-world walking speed in multiple conditions with a wearable device.
Relationship between mortality and vitality in patients with mild cognitive impairment/dementia: An 8-year retrospective study.
Cannabis and Driving in Older Adults.
Meaning in life and Parkinson's disease in the UK Biobank.
Occupation-Based Interventions to Improve Occupational Performance Among Older Adults Living in Long-Term Care: A Systematic Review.
Modifiable Risk Factors for Accelerated Decline in Processing Speed: Results from Three Dutch Population Cohorts.
Recognition of cognitive dysfunction in hospitalised older patients: a flash mob study.
[Effects of cognition-related lifestyles on early cognitive decline in community older adults in China].
Determinants of Bladder Care at Night in a Subacute Ward for Aged Patients: An Observational Study.
Cardiometabolic diseases and early cognitive decline: Mitigated by integrated active lifestyle for brain health.
Interventions to Reduce Loneliness in Community-Living Older Adults: a Systematic Review and Meta-analysis.
Prospective study design and data analysis in UK Biobank.
Occupational characteristics and disability-free survival after retirement age: an exploratory analysis from the ASPREE study.
Prioritizing determinants of cognitive function in healthy middle-aged and older adults: insights from a machine learning regression approach in the Canadian longitudinal study on aging.
Prognostic accuracy of 70 individual frailty biomarkers in predicting mortality in the Canadian Longitudinal Study on Aging.
Lower-Body Fractures and the Risk of Dementia: A Nationwide Population-Based Study.
Associations of polypharmacy with frailty severity and each frailty phenotype in community-dwelling older adults: Itabashi Longitudinal Study on Aging.
The association of longitudinal diet and waist-to-hip ratio from midlife to old age with hippocampus connectivity and memory in old age: a cohort study.
Prevalence of Possible Idiopathic Normal Pressure Hydrocephalus in Sweden: A Population-Based MRI Study in 791 70-Year-Old Participants.
Motoric cognitive risk syndrome trajectories and incident dementia over 10 years.
Mechanisms of Post-Stroke Fatigue: A Follow-Up From the Third Stroke Recovery and Rehabilitation Roundtable.
Multi-Domain Interventions for Dementia Prevention - A Systematic Review.
Evaluating motor progression of juvenile-onset Huntington's Disease: An Enroll-HD analysis.
Method to determine whether sleep phenotypes are driven by endogenous circadian rhythms or environmental light by combining longitudinal data and personalised mathematical models.
Multimodal lifestyle engagement patterns support cognitive stability beyond neuropathological burden.
Reduced overnight memory consolidation and associated alterations in sleep spindles and slow oscillations in early Alzheimer's disease.
Rationale and design of Juntendo Sarcopenia Registration to explore the predictors and prognosis of sarcopenia and frailty in the elderly in TOKYO (JUSTICE-TOKYO).
Within-person changes in the aging white matter microstructure and their modifiers: A meta-analysis and systematic review of longitudinal diffusion tensor imaging studies.
The methodology of the Agile Nudge University.
Atomic insights into the inhibition of R3 domain of tau protein by epigallocatechin gallate, quercetin and gallic acid.
Implication of grip strength assessment for the management of body weight in disability prevention in older adults.
Age at cardiovascular disease onset, dementia risk, and the role of lifestyle factors.
A cross-sectional survey on the effects of ambient temperature and humidity on health outcomes in individuals with chronic respiratory disease.
The MEDITAGING study: protocol of a two-armed randomized controlled study to compare the effects of the mindfulness-based stress reduction program against a health promotion program in older migrants in Luxembourg.
Evaluating the ability of a predictive vision-based machine learning model to measure changes in gait in response to medication and DBS within individuals with Parkinson's disease.
Cerebrovascular CO(2) reactivity and dynamic cerebral autoregulation through the eighth decade of life and their implications for cognitive decline.
Associations that Cardiorespiratory Fitness and Body Mass Index Loss Have with Deficit Accumulation Frailty.
Associations of socioeconomic status and healthy lifestyle with incident early-onset and late-onset dementia: a prospective cohort study.
Memory performance mediates subjective sleep quality associations with cerebrospinal fluid Alzheimer's disease biomarker levels and hippocampal volume among individuals with mild cognitive symptoms.
Economic Evaluation of Exercise or Cognitive and Social Enrichment Activities for Improved Cognition After Stroke.
Reducing fear and avoidance of memory loss improves mood and social engagement in community-based older adults: a randomized trial.
Exploring shared neural substrates underlying cognition and gait variability in adults without dementia.
Protective effects of apigenin on the brain transcriptome with aging.
Genome-Wide Association Analysis across Endophenotypes in Alzheimer's Disease: Main Effects and Disease Stage-Specific Interactions.

Sarcopenia Screened with SARC-F and Subjective Memory Complaints Are Independently Associated with Increased Risk of Incident Dementia among Cognitively Unimpaired Older Adults.
Tracing the temporal trends of modifiable risk factors in dementia: insights from the English Longitudinal Study of Ageing (2004-2019).
NMR metabolomic modelling of age and lifespan: a multi-cohort analysis.
Effect of physical activity on risk of Alzheimer's disease: A systematic review and meta-analysis of twenty-nine prospective cohort studies.
Circadian disturbances and frailty risk in older adults.
Effects of a 1 year aerobic and strength training on cognitive functioning after transient ischemic attack or minor stroke: A randomized controlled trial.
TWINGEN - protocol for an observational clinical biobank recall and biomarker study to identify individuals with high risk of Alzheimer's disease.
Association of Vitamin E and Cognitive Decline in Older Adults with and without the APOE…õ4 Allele: A Biracial Population-Based Community Study.
Relationship between baseline plasma p-tau181 and longitudinal changes in cognition and structural brain measures in a cohort of cognitively unimpaired older adults.
A longitudinal study of polygenic score and cognitive function decline considering baseline cognitive function, lifestyle behaviors, and diabetes among middle-aged and older US adults.
Improving Sleep Quality, Daytime Sleepiness, and Cognitive Function in Patients with Dementia by Therapeutic Exercise and NESA Neuromodulation: A Multicenter Clinical Trial.
A pilot observational study of gait changes over time before and after an unplanned hospital visit in long-term care residents with dementia.
Effect of dancing on freezing of gait in¬†patients with Parkinson's disease: A systematic review and meta-analysis.
Neuroticism, physical activity, and cognitive functioning in a population-based cohort of older adults.
Different types of screen time, physical activity, and incident dementia, Parkinson's disease, depression and multimorbidity status.
Associations of screen-based sedentary activities with all cause dementia, Alzheimer's disease, vascular dementia: a longitudinal study based on 462,524 participants from the UK Biobank.
Association of multimorbidity patterns with motoric cognitive risk syndrome among older adults: Evidence from a China longitudinal study.
Long-term Tai Chi practice in older adults is associated with "younger" functional abilities.
MyCOACH (COnnected Advice for Cognitive Health): a digitally delivered multidomain intervention for cognitive decline and risk of dementia in adults with mild cognitive impairment or subjective cognitive decline-study protocol for a randomised controlled trial.
Clinical Effectiveness of Cognitively Enhanced Tai Ji Quan Training on Global Cognition and Dual-Task Performance During Walking in Older Adults With Mild Cognitive Impairment or Self-Reported Memory Concerns : A Randomized Controlled Trial.
Imaging Brain Injury in Former National Football League Players.
Increased risk of dementia differs across cardiovascular diseases and types of dementia - Data from a nationwide study.
Mechanistic Intimate Insights into the Role of Hydrogen Sulfide in Alzheimer's Disease: A Recent Systematic Review.
Alteration of Metabolic Profiles during the Progression of Alzheimer's Disease.
Benzodiazepine Use and the Risk of Dementia in the Elderly Population: An Umbrella Review of Meta-Analyses.
A detection model of cognitive impairment via the integrated gait and eye movement analysis from a large Chinese community cohort.
Association of cardiometabolic multimorbidity and adherence to a healthy lifestyle with incident dementia: a large prospective cohort study.
Changes in Cortical Microstructure of the Human Brain Resulting from Long-Term Motor Learning.
Association of community-level social capital with dementia: A multilevel nine-year longitudinal study using data from the Japan Gerontological Evaluation Study.
The influence of baseline sleep on exercise-induced cognitive change in cognitively unimpaired older adults: A randomised clinical trial.
Protective effects of dog ownership against the onset of disabling dementia in older community-dwelling Japanese: A longitudinal study.
Volumetric brain correlates of gait associated with cognitive decline in community-dwelling older adults.
Differential Longitudinal Associations Between Domains of Cognitive Function and Physical Function: A 20-Year Follow-Up Study.
Genetic overlap between ALS and other neurodegenerative or neuromuscular disorders.
Education is power: preserving cognition in the UK biobank.
Prodromal Motoric Cognitive Risk Syndrome and Everyday Function.
A Remotely Coached Multimodal Lifestyle Intervention for Alzheimer's Disease Ameliorates Functional and Cognitive Outcomes.
Changes in Physical Activity and Incidence of Nonfatal Cardiovascular Events in 47,Äâ153 Survivors of Myocardial Infarction.
Assessment of technical and clinical utility of a bead-based flow cytometry platform for multiparametric phenotyping of CNS-derived extracellular vesicles.
Larger ischemic cores and poor collaterals among large vessel occlusions presenting in the late evening.
No phenotypic or genotypic evidence for a link between sleep duration and brain atrophy.
Change in physical activity and systolic blood pressure trajectories throughout mid-life and the development of dementia in older age: the HUNT study.
A Metabolomics Analysis of a Novel Phenotype of Older Adults at Higher Risk of Dementia.
Association of Pork Intake with Cognitive Performance in Older Adults Enrolled in the National Health and Nutrition Examination Survey (NHANES), 2011-2014 Data Cycles.
Objective sleep was longitudinally associated with brain amyloid burden in mild cognitive impairment.
Retrospective observational study of a novel smartphone app on the management of patients with mild cognitive impairment or mild dementia.
Objective Physical Function in the Alzheimer's Disease Continuum: Association with Cerebrospinal Fluid Biomarkers in the ALBION Study.
Metacognitive Strategy Training Improves Decision-Making Abilities in Amnestic Mild Cognitive Impairment.
The impacts of social restrictions during the COVID-19 pandemic on the physical activity levels of over 50-year olds: The CHARIOT COVID-19 Rapid Response (CCRR) cohort study.

Advanced Alzheimer's Disease Patients Show Safe, Significant, and Persistent Benefit in 6-Month Bryostatin Trial.
Effects of Brain Pathologies on Spatiotemporal Gait Parameters in Patients with Mild Cognitive Impairment.
Plant-based dietary patterns and the risk of dementia: a population-based study.
Using Cognitive-Motor Dual-Tasks and Functional Near-Infrared Spectroscopy to Characterize Older Adults with and without Subjective Cognitive Decline.
Exercise as an add-on treatment in individuals with schizophrenia: Results from a large multicenter randomized controlled trial.
The association of circulating endocannabinoids with neuroimaging and blood biomarkers of neuro-injury.
Risk factors and cognitive correlates of white matter hyperintensities in ethnically diverse populations without dementia: the COSMIC consortium.
Voluntary wheel exercise ameliorates cognitive impairment, hippocampal neurodegeneration and microglial abnormalities preceded by demyelination in a male mouse model of noise-induced hearing loss.
Association between dietary inflammatory index score and incident dementia: results from the Framingham heart Study offspring cohort.
Gait dysfunction in Alzheimer disease.
Lifestyle factors that affect cognitive function-a longitudinal objective analysis.
Whole genome deconvolution unveils Alzheimer's resilient epigenetic signature.
European Projects for Patients with Dementia and Their Caregivers.
The Mediterranean diet is associated with better cardiometabolic health for women in mid-life but not men: A PREVENT dementia cohort cross-sectional analysis.
Challenges in Using mHealth Data From Smartphones and Wearable Devices to Predict Depression Symptom Severity: Retrospective Analysis.
Association of Intensive Lifestyle Intervention for Type 2 Diabetes With Labor Market Outcomes.
Exploring shared genetics between maximal oxygen uptake and disease: the HUNT study.
Physical activity and amyloid beta in middle-aged and older adults: A systematic review and meta-analysis.
The Effect of the Human Brainstem Myelination on Gait Speed in Normative Aging.
Association between housework and the risk of dementia among older Chinese adults: a prospective cohort study.
Factors Associated With Neurocognitive Impairment Following Chemoradiotherapy in Patients With High-Grade Glioma: Results of a Prospective Trial.
Dance movement therapy for dementia.
Behavioral and Psychological Factors in Buffering Diabetes-related Disability Development.
Prolonged exposure to lung-derived cytokines is associated with inflammatory activation of microglia in patients with COVID-19.
Cardiovascular health and cognitive outcomes: Findings from a biracial population-based study in the United States.
Gender and age influence the association between gait speed and mild cognitive impairment in community-dwelling Japanese older adults: from the Japan Prospective Studies Collaboration for Ageing and Dementia (JPSC-AD).
Skeletal muscle mitochondrial function predicts cognitive impairment and is associated with biomarkers of Alzheimer's disease and neurodegeneration.
Prognostic relevance of gait-related cognitive functions for dementia conversion in amnestic mild cognitive impairment.
Estimating presymptomatic episodic memory impairment using simple hand movement tests: A cross-sectional study of a large sample of older adults.
The impact of neck pain on gait health: a systematic review and meta-analysis.
Cholesterol Paradox in Older People with Type 2 Diabetes Mellitus Regardless of Lipid-Lowering Drug Use: A Cross-Sectional Cohort Study.
Dietary Patterns and Alzheimer's Disease: An Updated Review Linking Nutrition to Neuroscience.
Exploratory Longitudinal Study of Ocular Structural and Visual Functional Changes in Subjects at High Genetic Risk of Developing Alzheimer's Disease.
Operational Modal Analysis of Near-Infrared Spectroscopy Measure of 2-Month Exercise Intervention Effects in Sedentary Older Adults with Diabetes and Cognitive Impairment.
Artificial intelligence for dementia-Applied models and digital health.
The multifaceted benefits of walking for healthy aging: from Blue Zones to molecular mechanisms.
Cerebrospinal Fluid Flow in Patients with Huntington's Disease.
An Exploratory Study of Nutrition Knowledge and Challenges Faced by Informal Carers of Community-Dwelling People with Dementia: Online Survey and Thematic Analysis.
Different Patterns of Locus Coeruleus MRI Alteration in Alzheimer's and Dementia with Lewy Bodies.
Personalized Integrated Care Promoting Quality of Life for Older People: Protocol for a Multicenter Randomized Controlled Trial.
Tea intake or consumption and the risk of dementia: a meta-analysis of prospective cohort studies.
Effectiveness of neurobic exercise program on memory performance in community-dwelling older adults with mild cognitive impairment: A randomized controlled crossover trial.
Misdiagnosis of rarest subtype of sporadic Creutzfeldt Jakob Disease: a case report.
Parkinson's Progression Markers Initiative: A Milestone-Based Strategy to Monitor Parkinson's Disease Progression.
No moderating influence of education on the association between changes in hippocampus volume and memory performance in aging.
Brain structure and phenotypic profile of superagers compared with age-matched older adults: a longitudinal analysis from the Vallecas Project.
The impact of COVID-19 pandemic on elderly with neurocognitive disorders.
Identification of peripheral blood immune infiltration signatures and construction of monocyte-associated signatures in ovarian cancer and Alzheimer's disease using single-cell sequencing.
Physical Activity Behavior and Its Association With Global Cognitive Function Three Months After Stroke: The Nor-COAST Study,Ä†.
Dementia in former amateur and professional contact sports participants: population-based cohort study, systematic review, and meta-analysis.
Association Between the AHA Life's Essential 8 Score and Incident All-Cause Dementia: A Prospective Cohort Study from UK Biobank.

Joint associations of physical activity and sleep duration with cognitive ageing: longitudinal analysis of an English cohort study.
Genetic associations vary across the spectrum of fasting serum insulin: results from the European IDEFICS/I.Family children's cohort.
Role of light walking pace on cognition: Findings from the Maine-Syracuse Longitudinal Study.
Suicide and depression in former contact sports participants: population-based cohort study, systematic review, and meta-analysis.
Potential neuroprotective effects of fermented foods and beverages in old age: a systematic review.
Brain Health Indicators Following Acute Neuro-Exergaming: Biomarker and Cognition in Mild Cognitive Impairment (MCI) after Pedal-n-Play (iPACES).
Trajectories of Physical Function and Behavioral, Psychological, and Social Well-Being in a Cohort of Swedish Older Adults.
Physical Activity Is Associated with Slower Cognitive Decline in Older Adults with Type 2 Diabetes.
Polygenic risk for termination of the 'healthspan' and its interactions with lifestyle factors: A prospective cohort study based on 288,359 participants.
Cognitive impairment is associated with gait variability and fall risk in amyotrophic lateral sclerosis.
The LEISURE Study: A Longitudinal Randomized Controlled Trial Protocol for a Multi-Modal Lifestyle Intervention Study to Reduce Dementia Risk in Healthy Older Adults.
A unique circular RNA expression pattern in the peripheral blood of myalgic encephalomyelitis/chronic fatigue syndrome patients.
Group leisure activities are associated with a lower risk of dementia than individual leisure activities: A 6-year longitudinal study from the Japan Gerontological Evaluation Study (JAGES).
Risk assessment and stratification of mild cognitive impairment among the Chinese elderly: attention to modifiable risk factors.
The role of lifestyle factors in cognitive health and dementia in oldest-old: A systematic review.
Assessing real-world gait with digital technology? Validation, insights and recommendations from the Mobilise-D consortium.
What tests and measures accurately diagnose persisting post-concussive symptoms in children, adolescents and adults following sport-related concussion? A systematic review.
When should an athlete retire or discontinue participating in contact or collision sports following sport-related concussion? A systematic review.
Correlated decline of cognitive and motor phenotypes and ADRD pathologies in old age.
Associations of rest-activity rhythm disturbances with stroke risk and post-stroke adverse outcomes.
Neurocognitive Test Performance and Concussion-Like Symptom Reporting Among Adolescent Athletes With Self-Reported Autism on Preseason Assessments.
Association of Telomere Length with Cognitive Impairment.
Does the Combination of Finnish Sauna Bathing and Other Lifestyle Factors Confer Additional Health Benefits? A Review of the Evidence.
Multi-domain prognostic models used in middle-aged adults without known cognitive impairment for predicting subsequent dementia.
Factors Associated With Cognitive Improvement After Bariatric Surgery Among Patients With Severe Obesity in the Netherlands.
Retinal imaging biomarkers of Alzheimer's disease: A systematic review and meta-analysis of studies using brain amyloid beta status for case definition.
Yoga, aerobic and stretching exercise effects on neurocognition: Randomized controlled trial protocol.
Author Correction: The power of genetic diversity in genome-wide association studies of lipids.
GP delivered brief weight loss advice: associations between in-consultation behaviour change techniques and patient weight loss in recorded primary care discussions.
Effects of multidomain lifestyle interventions on cognitive decline and Alzheimer's disease prevention: A literature review and future recommendations.
Technology-Assisted Cognitive Motor Dual-Task Rehabilitation in Chronic Age-Related Conditions: Systematic Review.
Using Digital Technology to Quantify Habitual Physical Activity in Community Dwellers With Cognitive Impairment: Systematic Review.
Associations between cognitive function and a range of significant life events in an elderly essential tremor cohort study.
Apoptosis in Alzheimer's disease: insight into the signaling pathways and therapeutic avenues.
Functional variants identify sex-specific genes and pathways in Alzheimer's Disease.
The Effects of Methylfolate on Cognitive Decline and Dementia: A Protocol for Systematic Review and Meta-Analysis.
Serum TCA cycle metabolites in Lewy bodies dementia and Alzheimer's disease: Network analysis and cognitive prognosis.
Social isolation and subsequent health and well-being in older adults: A longitudinal outcome-wide analysis.
The association of allopurinol with persistent physical disability and frailty in a large community based older cohort.
[Influencing factors of mild cognitive impairment among the Chinese elderly: a meta-analysis].
Cardiometabolic multimorbidity, lifestyle behaviours, and cognitive function: a multicohort study.
Equating norms between the ALS Cognitive Behavioral Screen (ALS-CBS,Ñ¢) and the Edinburgh Cognitive and Behavioural ALS Screen (ECAS) in non-demented ALS patients.
NREM sleep as a novel protective cognitive reserve factor in the face of Alzheimer's disease pathology.
Machine Learning-Based Prediction of Functional Disability: a Cohort Study of Japanese Older Adults in 2013-2019.
Correlates of Person-Specific Rates of Change in Sensor-Derived Physical Activity Metrics of Daily Living in the Rush Memory and Aging Project.
Trajectories of care of community-dwelling people living with dementia: a multidimensional state sequence analysis.
Association between nutrition and cognition in a multi-ethnic cohort from Singapore.
Serum neurofilament light chain levels are associated with all-cause mortality in the general US population.
Age- and sex-specific associations between sarcopenia severity and poor cognitive function among community-dwelling older adults in Japan: The IRIDE Cohort Study.
A 12-month randomised pilot trial of the Alzheimer's and music therapy study: a feasibility assessment of music therapy and physical activity in patients with mild-to-moderate Alzheimer's disease.

Association of Handgrip Strength Weakness and Asymmetry With Incidence of Motoric Cognitive Risk Syndrome in the China Health and Retirement Longitudinal Study.
Interplay of Metabolome and Gut Microbiome in Individuals With Major Depressive Disorder vs Control Individuals.
Impaired muscle function, including its decline, is related to greater long-term late-life dementia risk in older women.
Revealing the correlations between brain cortical characteristics and susceptibility genes for Alzheimer disease: a cross-sectional study.
Geriatric Cognitive Decline and Polypharmacy.
Ambient Monitoring of Gait and Machine Learning Models for Dynamic and Short-Term Falls Risk Assessment in People With Dementia.
Associations of physical function and body mass index with functional brain networks in community-dwelling older adults.
A decade of aging in healthy older adults: longitudinal findings on cerebrovascular and cognitive health.
Plasma metabolomic signatures of dual decline in memory and gait in older adults.
Serious Games for Learning Among Older Adults With Cognitive Impairment: Systematic Review and Meta-analysis.
Change in functional disability and its trends among older adults in Korea over 2008-2020: a 4-year follow-up cohort study.
The effect of weight loss following 18 months of lifestyle intervention on brain age assessed with resting-state functional connectivity.
Correction: Walking pace, handgrip strength, age, APOE genotypes, and new-onset dementia: the UK Biobank prospective cohort study.
Association of Brain Age, Lesion Volume, and Functional Outcome in Patients With Stroke.
Objective assessment of patients with idiopathic normal pressure hydrocephalus following ventriculoperitoneal shunt placement using activity-monitoring data: pilot study.
Association between physical activity, growth differentiation factor 15 and bodyweight in older adults: A longitudinal mediation analysis.
Prevalence and Characteristics Associated With Post-COVID-19 Condition Among Nonhospitalized Adolescents and Young Adults.
Plasma inflammation for predicting phenotypic conversion and clinical progression of autosomal dominant frontotemporal lobar degeneration.
Cognitive Function, and Its Relationships with Comorbidities, Physical Activity, and Muscular Strength in Korean Older Adults.
Modulating arousal to overcome gait impairments in Parkinson's disease: how the noradrenergic system may act as a double-edged sword.
The multiple roles of GH in neural ageing and injury.
Detection of visuomotor dysfunction in mild traumatic brain injury using binocular retinal polarization scanning.
Association of Bone Mineral Density and Dementia: The Rotterdam Study.
Cardiorespiratory fitness and risk of Alzheimer's disease and related dementias among American veterans.
Prevalence of physical frailty, including risk factors, up to 1 year after hospitalisation for COVID-19 in the UK: a multicentre, longitudinal cohort study.
Neurodegenerative disease among male elite football (soccer) players in Sweden: a cohort study.
Effect of atherosclerosis on 5-year risk of major vascular events in patients with transient ischaemic attack or minor ischaemic stroke: an international prospective cohort study.
A Longitudinal Study of Acculturation in Context and Cardiovascular Health and Their Effects on Cognition Among Older Latino Adults.
The Bidirectional Association Between Cognitive Function and Gait Speed in Chinese Older Adults: Longitudinal Observational Study.
Mediterranean diet adherence is associated with lower dementia risk, independent of genetic predisposition: findings from the UK Biobank prospective cohort study.
Physical Frailty, Genetic Predisposition, and Incident Parkinson Disease.
Interactions Between Neuropsychiatric Symptoms and Alzheimer's Disease Neuroimaging Biomarkers in Predicting Longitudinal Cognitive Decline.
Exploration of the Shared Molecular Mechanisms between COVID-19 and Neurodegenerative Diseases through Bioinformatic Analysis.
Dementia prevention in memory clinics: recommendations from the European task force for brain health services.
Association between exercise habit changes and incident dementia after ischemic stroke.
Association of Mediterranean-DASH Intervention for Neurodegenerative Delay and Mediterranean Diets With Alzheimer Disease Pathology.
Lower Myelin Content Is Associated With Lower Gait Speed in Cognitively Unimpaired Adults.
The impact of stroke on the ability to live an independent life at old age: a community-based cohort study of Swedish men.
The importance of high quality real-life social interactions during the COVID-19 pandemic.
Effects of 12-week Tai Chi program on physical function, depression, and quality of life among cognitively impaired older adults: a feasibility study.
Predictors of life-space mobility in patients with fracture 3 months after discharge from convalescent rehabilitation ward: a prospective longitudinal study.
Identification of High Likelihood of Dementia in Population-Based Surveys using Unsupervised Clustering: a Longitudinal Analysis.
Effect of physical activity intervention on cognitive function in China: A cluster randomized trial.
Football (Soccer) as a Probable Cause of Long-Term Neurological Impairment and Neurodegeneration: A Narrative Review of the Debate.
Association of Cardiovascular Health With Life Expectancy Free of Cardiovascular Disease, Diabetes, Cancer, and Dementia in UK Adults.
Lifestyle Changes Reduced Estimated White Matter Hyperintensities Based on Retinal Image Analysis.
The Role of Gut Microbiota in Various Neurological and Psychiatric Disorders-An Evidence Mapping Based on Quantified Evidence.
Timing of physical activity across adulthood on later-life cognition: 30 years follow-up in the 1946 British birth cohort.
Cardiovascular health profiles, systemic inflammation, and physical function in older adults: A population-based study.
Factors predictive of functional outcomes and quality of life in patients with fragility hip fracture: A retrospective cohort study.
Health Behavior Patterns and Associated Risk of Memory-Related Disorders Among Middle-Aged and Older Chinese Couples.

Effects of community-based frailty-preventing intervention on all-cause and cause-specific functional disability in older adults living in rural Japan: A propensity score analysis.
Preventable risk factors of dementia: Population attributable fractions in a Brazilian population-based study.
Conventional Synthetic Disease-Modifying Anti-Rheumatic Drugs and the Risk of Vascular Dementia in Patients with Spondyloarthritis: A Database Cohort Study.
Socioeconomic status as a risk factor for motoric cognitive risk syndrome in a community-dwelling population: A longitudinal observational study.
The Study of Muscle, Mobility and Aging (SOMMA): A Unique Cohort Study About the Cellular Biology of Aging and Age-related Loss of Mobility.
Investigation of the gait parameters after donepezil treatment in patients with alzheimer' s disease.
The effect of bright light therapy in migraine patients with sleep disturbance: A prospective, observational cohort study protocol.
Associations of healthy lifestyle with depression and post-depression dementia: A prospective cohort study.
Vigorous, regular physical exercise may slow disease progression in Alzheimer's disease.
Transcranial Color-Coded Doppler Cerebral Hemodynamics Following Aerobic Exercise Training: Outcomes From a Pilot Randomized Clinical Trial.
Association of white matter lesions and brain atrophy with the development of dementia in a community: the Hisayama Study.
The beneficial effect on cognition of noninvasive brain stimulation intervention in patients with dementia: a network meta-analysis of randomized controlled trials.
Sex Differences in Dementia.
Accelerometer-measured physical activity and sitting with incident mild cognitive impairment or probable dementia among older women.
Mediterranean diet is associated with a lower probability of prodromal Parkinson's disease and risk for Parkinson's disease/dementia with Lewy bodies: A longitudinal study.
Exploring the associations of daily movement behaviours and mid-life cognition: a compositional analysis of the 1970 British Cohort Study.
Modifiable healthy behaviours and incident disability in older adults: Analysis of combined data from two cohort studies in Japan.
Rationale for a Multi-Factorial Approach for the Reversal of Cognitive Decline in Alzheimer's Disease and MCI: A Review.
Trifluridine/Tipiracil in the Real-World Management of Metastatic Gastric and Gastroesophageal Junction Cancers in Canada.
Predictors of incident reversible and potentially reversible cognitive frailty among Taiwanese older adults.
Machine learning for predicting neurodegenerative diseases in the general older population: a cohort study.
Impact of pre-analytical sample handling factors on plasma biomarkers of Alzheimer's disease.
A personhood and citizenship training workshop for care home staff to potentially increase wellbeing of residents with dementia: intervention development and feasibility testing of a cluster randomised controlled trial.
Barriers and facilitators of providing standard of care diabetes management at primary care level in geriatric population.
Chronic pain increases the risk of motoric cognitive risk syndrome at 4,Äâyears of follow-up: evidence from the China Health and Retirement Longitudinal Study.
MIND Dietary Pattern and Its Association with Cognition and Incident Dementia in the UK Biobank.
Influence of a 12-Month Structured Exercise Program on the Micronutrient-Cognitive Fitness-Physical Association Profiles in Mild Cognitive Impairment.
Water-Reaching Platform for Longitudinal Assessment of Cortical Activity and Fine Motor Coordination Defects in a Huntington Disease Mouse Model.
Age- and sex-specific modifiable risk factor profiles of dementia: evidence from the UK Biobank.
Incidence and burden of injury at the Tokyo 2020 Paralympic Games held during the COVID-19 pandemic: a prospective cohort study of 66 045 athlete days.
Healthspan and chronic disease burden among young adult and middle-aged male former American-style professional football players.
Social network and severe lower respiratory tract infections in older adults: findings from a Swedish longitudinal population-based study.
Matching science to reality: how to deploy a participant-driven digital brain health platform.
Effects of DementiaNet's Community Care Network Approach on Admission Rates and Healthcare Costs: A Longitudinal Cohort Analysis.
Implicating genes, pleiotropy, and sexual dimorphism at blood lipid loci through multi-ancestry meta-analysis.
Dose-response Associations of Physical Activity and Sitting Time With All-cause Mortality in Older Japanese Adults.
Impact of exercising alone and exercising with others on the risk of cognitive impairment among older Japanese adults.
Home-Based Individualized Cognitive Stimulation (iCS) Therapy in Portuguese Psychiatric Patients: A Randomized Controlled Trial.
Cross-sectional and prospective associations between homocysteine and a frailty index: A post-hoc analysis of the multidomain Alzheimer's prevention trial (MAPT).
Environmental and lifestyle risk factors for early-onset dementia: a systematic review.
Association between grip strength and cognitive impairment in older American adults.
Can timed up and go subtasks predict functional decline in older adults with cognitive impairment?
Deep Grey Matter Volume is Reduced in Amateur Boxers as Compared to Healthy Age-matched Controls.
Genetic expression changes and pathologic findings associated with hyperhomocysteinemia in human autopsy brain tissue.
APOE alleles are associated with sex-specific structural differences in brain regions affected in Alzheimer's disease and related dementia.
Operationalization of Intrinsic Capacity in Older People and Its Association With Subsequent Disability, Hospital Admission and Mortality: Results From The English Longitudinal Study of Ageing.
Association of polypharmacy and motoric cognitive risk syndrome in older adults: A 4-year longitudinal study in China.
Exposure to Ambient Air Pollution and the Incidence of Dementia in the Elderly of England: The ELSA Cohort.
Relationships of Physical Activity, Depression, and Sleep with Cognitive Function in Community-Dwelling Older Adults.
Loneliness and risk of all-cause, Alzheimer's, vascular, and frontotemporal dementia: a prospective study of 492,322 individuals over 15 years.
Relating American Football Age of First Exposure to Patient-Reported Outcomes and Medical Diagnoses Among Former National Football League Players: An NFL-LONG study.

Adherence to a Mediterranean Diet is associated with physical and cognitive health: A cross-sectional analysis of community-dwelling older Australians.
Feasibility and preliminary effects of exercise interventions on plasma biomarkers of Alzheimer's disease in the FIT-AD trial: a randomized pilot study in older adults with Alzheimer's dementia.
Handgrip strength is associated with learning and verbal fluency in older men without dementia: insights from the NHANES.
Effect of antioxidant intake patterns on risks of dementia and cognitive decline.
Association between systemic rheumatic diseases and dementia risk: A meta-analysis.
The interrelationship between physical activity intensity, cardiorespiratory fitness, and executive function in middle-aged adults: An observational study of office workers.
Sex- and Neuropsychiatric-Dependent Circadian Alterations in Daily Voluntary Physical Activity Engagement and Patterns in Aged 3xTg-AD Mice.
Accelerometer Cut-Points for Physical Activity Assessment in Adults with Mild to Moderate Huntington's Disease: A Cross-Sectional Multicentre Study.
Time-varying risk of death after SARS-CoV-2 infection in Swedish long-term care facility residents: a matched cohort study.
Association of Dietary Intake of Flavonols With Changes in Global Cognition and Several Cognitive Abilities.
Understanding how people with Parkinson's disease turn in gait from a real-world in-home dataset.
Repeated performance of spatial memory tasks ameliorates cognitive decline in APP/PS1 mice.
Longitudinal association between falls and motoric cognitive risk syndrome among community-dwelling older adults.
The interplay between the gut-brain axis and the microbiome: A perspective on psychiatric and neurodegenerative disorders.
Participant characteristics associated with the effects of a physical and cognitive training program on executive functions.
Longitudinal effects of exercise according to the World Health Organization guidelines on cognitive function in middle-aged and older adults.
High consumption of whole grain foods decreases the risk of dementia and Alzheimer's disease: Framingham Offspring Cohort.
CCR5-Œî32 polymorphism-a possible protective factor from gait impairment amongst post-stroke patients.
Replacement of leisure-time sedentary behavior with various physical activities and the risk of dementia incidence and mortality: A prospective cohort study.
Non-drug Therapies for Alzheimer's Disease: A Review.
Plasma Amyloid-Œ≤ in Relation to Cardiac¬†Function and Risk of Heart¬†Failure¬†in General Population.
Can dementia risk be reduced by following the American Heart Association's Life's Simple 7? A systematic review and dose-response meta-analysis.
Exercise Training Prevents the Loss of Wall Thickness and Lowers Expression of Alzheimer's Related Proteins in 3xTg Mouse Jejunum.
Comparison of Rehabilitative Interventions That Ameliorate Post-stroke Working Memory Deficit: A Systematic Review.
Patterns of depressive symptoms over 16 Years with incident dementia: The Health and Retirement Study.
Relationship between arterial stiffness and cognitive function in outpatients with dementia and mild cognitive impairment compared with community residents without dementia.
Association of plasma brain-derived neurotrophic factor levels and frailty in community-dwelling older adults.
Objective and Subjective Measurements of Motor Function: Results from the HELIAD Study.
Association of Preeclampsia with Incident Dementia and Alzheimer's Disease among Women in the Framingham Offspring Study.
Metabolic risk factors and psychosocial problems independently explain poor sleep quality and obstructive sleep apnea symptoms among adults in urban India.
Cognitive improvement effects of Tian Wang Bu Xin Dan (Cheonwangbosimdan) in patients with mild cognitive impairment: protocol for a randomized placebo-controlled pilot clinical trial.
The Effect of Agomelatine in Behavioral and Psychological Symptoms of Dementia.
Association of Early Weight Change With Cognitive Decline in Patients With Parkinson Disease.
Physical activity, the quadrangle of health, and dementia risk.
A saturated map of common genetic variants associated with human height.
Network analysis to identify symptoms clusters and temporal interconnections in oncology patients.
Association Between Dietary Habits in Midlife With Dementia Incidence Over a 20-Year Period.
Risk factors, ethnicity and dementia: A UK Biobank prospective cohort study of White, South Asian and Black participants.
Built Environment Design Interventions at the Exits of Secured Dementia Care Units: A Review of the Empirical Literature.
Intra-beat biomarker for accurate continuous non-invasive blood pressure monitoring.
Connecting real-world digital mobility assessment to clinical outcomes for regulatory and clinical endorsement-the Mobilise-D study protocol.
Functional Constipation is Associated with a Decline in Word Recognition 2 Years Later in Community-Dwelling Older Adults: The Korean Frailty and Aging Cohort Study.
Diabetes Mellitus is Associated With Higher Serum Neurofilament Light Chain Levels in the General US Population.
Digital culturally tailored marketing for enrolling Latino participants in a web-based registry: Baseline metrics from the Brain Health Registry.
Community-based mental health and well-being interventions for older adults in low- and middle-income countries: a systematic review and meta-analysis.
Potential association between frailty and pTau in community-dwelling older adults.
Online medical history taking course: Opportunities and limitations in comparison to traditional bedside teaching.
Risk of subsequent disabling or fatal stroke in patients with transient ischaemic attack or minor ischaemic stroke: an international, prospective cohort study.
Prevalence and development of idiopathic normal pressure hydrocephalus: A 16-year longitudinal study in Japan.
Fitness is positively associated with hippocampal formation subfield volumes in schizophrenia: a multiparametric magnetic resonance imaging study.
Longitudinal Associations Between Falls and Risk of Gait Decline: Results From the Central Control of Mobility and Aging Study.

The Influence of Context on Implementation and Improvement: Protocol for a Mixed Methods, Secondary Analyses Study.
Effectiveness of a complex intervention of group-based nutrition and physical activity to prevent frailty in pre-frail older adults (SUPER): a randomised controlled trial.
Effects of a Multicomponent Exercise Program on Improving Frailty in Post-COVID-19 Older Adults after Intensive Care Units: A Single-Group Retrospective Cohort Study.
Musical Activity During Life Is Associated With Multi-Domain Cognitive and Brain Benefits in Older Adults.
Metformin use is associated with a reduced risk of cognitive impairment in adults with diabetes mellitus: A systematic review and meta-analysis.
Association of Daily Step Count and Intensity With Incident Dementia in 78,ÄØ430 Adults Living in the UK.
Genome-wide meta-analysis for Alzheimer's disease cerebrospinal fluid biomarkers.
Fragmentation, circadian amplitude, and fractal pattern of daily-living physical activity in people with multiple sclerosis: Is there relevant information beyond the total amount of physical activity?
The prevention of falls in patients with Parkinson's disease with in-home monitoring using a wearable system: a pilot study protocol.
Rationale and design of the "NEurodegeneration: Traumatic brain injury as Origin of the Neuropathology (NEwTON)" study: a prospective cohort study of individuals at risk for chronic traumatic encephalopathy.
Physical activity in leisure time and at work and risk of dementia: A prospective cohort study of 117,616 individuals.
Using Interaction between Cognitive and Motor Impairment for Risk Screening of Major Neurocognitive Disorders: Results of the EPIDOS Observational Cohort Study.
Predicting systemic therapy toxicity in older adult patients with advanced non-small cell lung cancer: A prospective multicenter study of National Hospital Organization in Japan.
Diet, nutrition, and cognitive function: A narrative review of Japanese longitudinal studies.
Prevalence and Determinants of Restless Legs Syndrome (Willis-Ekbom Disease) in an Older Greek Population.
Cognitive reserve and risk of mobility impairment in older adults.
Relation of CDC42, Th1, Th2, and Th17 cells with cognitive function decline in Alzheimer's disease.
Incidence, early case fatality and determinants of stroke in Iran: Golestan Cohort Study.
Identification of Major Cognitive Disorders in Self-Reported versus Administrative Health Data: A Cohort Study in Quebec.
Factors Influencing Long-Term Physical Activity Maintenance: A Qualitative Evaluation of a Physical Activity Program for Inactive Older Adults at Risk of Cognitive Decline: The INDIGO Follow-Up Study.
The New Zealand Parkinson's progression programme.
Effect of Wearable Sensor-Based Exercise on Musculoskeletal Disorders in Individuals With Neurodegenerative Diseases: A Systematic Review and Meta-Analysis.
Gait Characteristics and Cognitive Function in Middle-Aged Adults with and without Type 2 Diabetes Mellitus: Data from ENBIND.
Association of Playing Cards or Mahjong with Cognitive Function in Chinese Older Adults.
Leisure Activities and the Risk of Dementia: A Systematic Review and Meta-analysis.
Lifestyle and Cognitive Decline in Community-Dwelling Stroke Survivors.
A multi-layer functional genomic analysis to understand noncoding genetic variation in lipids.
Influence of cardiovascular risk burden on pulmonary function trajectory: role of physical and social activities.
[Factors predictive of adherence to a non-pharmacological intervention in nursing home research: a substudy of the LEDEN trial].
Association between depression and subjective cognitive complaints in 47 low- and middle-income countries.
Effect of Graded Sensorimotor Retraining on Pain Intensity in Patients With Chronic Low Back Pain: A Randomized Clinical Trial.
Sedentary behaviour and brain health in middle-aged and older adults: A systematic review.
The Longitudinal Association of Lifestyle with Cognitive Health and Dementia Risk: Findings from the HELIAD Study.
Alzheimer's Disease Risk Variant rs3865444 in the CD33 Gene: A Possible Role in Susceptibility to Multiple Sclerosis.
Dementia and Risk Factors: Results from a Prospective, Population-Based Cohort Study.
Telomere Length and Risk of Incident Fracture and Arthroplasty: Findings From UK Biobank.
Phototherapy for Cognitive Function in Patients With Dementia: A Systematic Review and Meta-Analysis.
Long-term outcomes of edaravone in amyotrophic lateral sclerosis in South Korea: 72-week observational study.
Physical activity and exercise outcomes in Huntington's disease (PACE-HD): results of a 12-month trial-within-cohort feasibility study of a physical activity intervention in people with Huntington's disease.
Dementia risk and dynamic response to exercise: A non-randomized clinical trial.
Grip Strength, Gait Speed and Plasma Markers of Neurodegeneration in Asymptomatic Middle-aged and Older Adults.
Association between Social Activity and Development of Dementia in Hearing Impairment: A Cohort Study in Japan from Japan Gerontological Evaluation Study.
Association of Plasma Neurofilament Light Chain With Glycaemic Control and Insulin Resistance in Middle-Aged Adults.
Feasibility, Safety, and Effectiveness of Telerehabilitation in Mild-to-Moderate Parkinson's Disease.
APOE Œµ4 and exercise interact in a sex-specific manner to modulate dementia risk factors.
Study protocol for the Alzheimer and music therapy study: An RCT to compare the efficacy of music therapy and physical activity on brain plasticity, depressive symptoms, and cognitive decline, in a population with and at risk for Alzheimer's disease.
The early impacts of COVID-19 on people living with dementia: part I of a¬†mixed-methods systematic review.
Effect of Simultaneous Dual-Task Training on Regional Cerebral Blood Flow in Older Adults with Amnestic Mild Cognitive Impairment.

Natural Pattern of Cognitive Aging.
Non-Modifiable Factors as Moderators of the Relationship Between Physical Activity and Brain Volume: A Cross-Sectional UK Biobank Study.
Investigating the use of plasma pTau181 in retired contact sports athletes.
Emergency department visits among people with predementia highly predicts conversion to dementia.
An Algorithm for Accurate Marker-Based Gait Event Detection in Healthy and Pathological Populations During Complex Motor Tasks.
Older adults' preferences for and actual situations of artificial hydration and nutrition in end-of-life care: An 11-year follow-up study in a care home.
Longitudinal associations of mid-life employment status with impaired physical function in the Study of Women's Health Across the Nation.
Differential and shared genetic effects on kidney function between diabetic and non-diabetic individuals.
Interplay between gait and neuropsychiatric symptoms in Parkinson's Disease.
Risk factors for falls in community-dwelling older people with mild cognitive impairment: a prospective one-year study.
Serum neurofilament as a predictor of 10-year grey matter atrophy and clinical disability in multiple sclerosis: a longitudinal study.
Association Between Internet Use, Cognitive Function, and Globus Pallidus Volumes: A Study Among the Elderly in Chinese Communities.
Relationships of Cerebral Perfusion With Gait Speed Across Systolic Blood Pressure Levels and Age: A Cohort Study.
Association of Dual Decline in Cognition and Gait Speed With Risk of Dementia in Older Adults.
Transcriptomic Profiling Reveals Discrete Poststroke Dementia Neuronal and Gliovascular Signatures.
A retrospective analysis of all-cause and cause-specific mortality rates in French male professional footballers.
Depressive Symptoms as Mediator on the Link between Physical Activity and Cognitive Function: Longitudinal Evidence from Older Adults in China.
Mixed Evidence of an Association between Self-Rated Hearing Difficulties and Falls: Prospective Analysis of Two Longitudinal Studies.
Lifestyle Affects Amyloid Burden and Cognition Differently in Men and Women.
Comparison of long-term effects of exergaming (Xbox one kinet) and companionship programs on attitude towards dementia and the older adults among adolescents: a quasi-experimental longitudinal study.
Chronic Stroke Sensorimotor Impairment Is Related to Smaller Hippocampal Volumes: An ENIGMA Analysis.
Migraine, Cognitive Decline, and Dementia in Older Adults: A Population-Based Study.
Do the Impacts of Mentally Active and Passive Sedentary Behavior on Dementia Incidence Differ by Physical Activity Level? A 5-year Longitudinal Study.
Measuring Recovery and Understanding Long-Term Deficits in Balance, Ankle Mobility and Hip Strength in People after an Open Reduction and Internal Fixation of Bimalleolar Fracture and Their Impact on Functionality: A 12-Month Longitudinal Study.
Nationwide Prevalence and Outcomes of Long-Term Nasogastric Tube Placement in Adults.
Adherence to the Mediterranean diet and incident frailty: Results from a longitudinal study.
Study Protocol of a Comprehensive Activity Promotion Program for the Prevention of Dementia: A Randomized Controlled Trial Protocol.
Development of a Quality Assessment Tool for Outpatient Infusion Clinics: A Literature Review and Pilot Survey.
Patterns of lifestyle behaviours from mid- through later-life in relation to exceptional episodic memory performance in older women: the Nurses' Health Study.
Do P2Y12 receptor inhibitors prescribed poststroke modify the risk of cognitive disorder or dementia? Protocol for a target trial using multiple national Swedish registries.
Biopsychosocial Correlates of Subjective Cognitive Decline and Related Worry in the Canadian Longitudinal Study on Aging.
Cognitive and Functional Abilities in an Older Adult Veteran Before and After Contracting COVID-19.
Sex differences in the association between cardiovascular diseases and dementia subtypes: a prospective analysis of 464,616 UK Biobank participants.
Cerebrovascular function and its association with systemic artery function and stiffness in older adults with and without mild cognitive impairment.
Body-Worn Sensors for Parkinson's disease: A qualitative approach with patients and healthcare professionals.
Latin Dance and Working Memory: The Mediating Effects of Physical Activity Among Middle-Aged and Older Latinos.
Physical, social, and dietary behavioral changes during the COVID-19 crisis and their effects on functional capacity in older adults.
Test-retest reliability of multi-parametric maps (MPM) of brain microstructure.
Longitudinal changes in cerebral blood flow and their relation with cognitive decline in patients with dementia: Current knowledge and future directions.
Global population attributable fraction of potentially modifiable risk factors for mental disorders: a meta-umbrella systematic review.
Modifying effects of race and ethnicity and APOE on the association of physical activity with risk of Alzheimer's disease and related dementias.
Residential Green Space and Cognitive Function in a Large Cohort of Middle-Aged Women.
Amateur Boxing and Dementia: Cognitive Impairment Within the 35-Year Caerphilly Cohort Study.
Longitudinal study of the effect of a 5-year exercise intervention on structural brain complexity in older adults. A Generation 100 substudy.
Handgrip strength and all-cause dementia incidence and mortality: findings from the UK Biobank prospective cohort study.
Gender gap in deep brain stimulation for Parkinson's disease.
Asymptomatic COVID-19 in the elderly: dementia and viral clearance as risk factors for disease progression.
The Survey of the Health of Wisconsin (SHOW) Program: An Infrastructure for Advancing Population Health.
Stability of Montreal Cognitive Assessment in Individuals with Mild Cognitive Impairment: Potential Influence of Practice Effect.
Environmental stimulation in Huntington disease patients and animal models.
Chemical genetic activation of the cholinergic basal forebrain hippocampal circuit rescues memory loss in Alzheimer's disease.
The Cure VCP Scientific Conference 2021: Molecular and clinical insights into neurodegeneration and myopathy linked to multisystem proteinopathy-1 (MSP-1).

Roles of physical activity and diet in cognitive aging: is more better?
Development and validation of prediction model for incident overactive bladder: The Nagahama study.
The economic burden of dementia in low- and middle-income countries (LMICs): a systematic review.
Dietary Fiber Intake is Associated with Cognitive Function in Older Adults: Data from the National Health and Nutrition Examination Survey.
Normative data for three physical frailty parameters in an aging, rural Indian population.
Dietary Inflammatory Index score and prodromal Parkinson's disease incidence: The HELIAD study.
Examination of Neurofilament Light Chain Serum Concentrations, Physical Activity, and Cognitive Decline in Older Adults.
Risk of conversion from mild cognitive impairment to dementia in low- and middle-income countries: A systematic review and meta-analysis.
Factors associated with non-walking 4 months after hip fracture. A prospective study of 23,759 fractures.
Prevalence and risk factors of fall-related injury among older adults in India: evidence from a cross-sectional observational study.
Physical activity as a protective factor for dementia and Alzheimer's disease: systematic review, meta-analysis and quality assessment of cohort and case-control studies.
A Multimodal Intervention to Improve Cognition in Community-dwelling Older Adults.
Evaluation of strategies to recruit and retain older people with dementia and their informal carers into a Tai Chi Trial to improve balance and prevent falls.
Occasions for laughter and dementia risk: Findings from a six-year cohort study.
An exploration of trifluridine/tipiracil in combination with irinotecan in patients with pretreated advanced gastric cancer.
Feasibility and Acceptability of a Dietary Intervention to Reduce Salt Intake and Increase High-Nitrate Vegetable Consumption in Malaysian Middle-Aged and Older Adults with Elevated Blood Pressure: Findings from the DePEC-Nutrition Trial.
Prospective Associations of Tea Consumption With Risk of Cognitive Decline in the Elderly: A 1-Year Follow-Up Study in China.
The effect of physical activity interventions on cognitive function of older adults: A systematic review of clinical trials.
Sixteen Weeks of Aerobic Exercise does not Alter Resting-state Connectivity of the Precuneus in Patients with Alzheimer's Disease.
Effects of Changes in Physical Activity with Cognitive Decline in Korean Home-Dwelling Older Adults.
Association between orthostatic blood pressure dysregulation and geriatric syndromes: a cross-sectional study.
Efficacy of a Virtual Education Program for Family Caregivers of Persons Living with Dementia.
Dose-response relationships of sarcopenia parameters with incident disability and mortality in older Japanese adults.
Profiling the Skeletal Muscle Proteome in Patients on Atypical Antipsychotics and Mood Stabilizers.
Alzheimer's disease research progress in the Mediterranean region: The Alzheimer's Association International Conference Satellite Symposium.
Dual-task gait and mild behavioral impairment: The interface between non-cognitive dementia markers.
Types of Elder Abuse and Dementia Onset among Older Adults in Japan: A 6-year Longitudinal Study from the Japan Gerontological Evaluation Study.
Multiomics Analysis of Structural Magnetic Resonance Imaging of the Brain and Cerebrospinal Fluid Metabolomics in Cognitively Normal and Impaired Adults.
Association between CSF biomarkers of Alzheimer's disease and neuropsychiatric symptoms: Mayo Clinic Study of Aging.
Blood pressure screening in midlife aids in prediction of dementia later in life.
Relationship of fasting glucose and longitudinal Alzheimer's disease imaging markers.
The prevalence and associated factors of motoric cognitive risk syndrome in multiple ethnic middle-aged to older adults in west China: a cross-sectional study.
Air quality improvement and cognitive decline in community-dwelling older women in the United States: A longitudinal cohort study.
The Effects of Attention-Deficit/Hyperactivity Disorder Symptoms on the Association between Head Impacts and Post-Season Neurocognitive and Behavioral Outcomes.
Oophorectomy and rate of dementia: a prospective cohort study.
Development of a standard of care for patients with valosin-containing protein associated multisystem proteinopathy.
A New Perspective on the Treatment of Alzheimer's Disease and Sleep Deprivation-Related Consequences: Can Curcumin Help?
Real-time individual benefit from social interactions before and during the lockdown: the crucial role of personality, neurobiology and genes.
Physical Activity and Aging: Exploring Motivations of Masters Swimmers.
Motor cortex plasticity response to acute cardiorespiratory exercise and intermittent theta-burst stimulation is attenuated in premanifest and early Huntington's disease.
Poor Diet, Long Sleep, and Lack of Physical Activity Are Associated with Inflammation among Non-Demented Community-Dwelling Elderly.
A new paradigm in sarcopenia: Cognitive impairment caused by imbalanced myokine secretion and vascular dysfunction.
Prospective Associations of Plasma Growth Differentiation Factor 15 With Physical Performance and Cognitive Functions in Older Adults.
Neurofilament light chain concentration in an aging population.
Is there an association between non-alcoholic fatty liver disease and cognitive function? A systematic review.
Temporal transcriptomic changes in long non-coding RNAs and messenger RNAs involved in the host immune and metabolic response during Toxoplasma gondii lytic cycle.
Personal Social Networks of Community-Dwelling Oldest Old During the Covid-19 Pandemic-A Qualitative Study.
HomeCoRe for Telerehabilitation in Mild or Major Neurocognitive Disorders: A Study Protocol for a Randomized Controlled Trial.
Dual-Task Gait as a Predictive Tool for Cognitive Impairment in Older Adults: A Systematic Review.
The landscape of human tissue and cell type specific expression and co-regulation of senescence genes.
Plasma MCP-1 and changes on cognitive function in community-dwelling older adults.

Association of the Polygenic Risk Score With the Probability of Prodromal Parkinson's Disease in Older Adults.
Known in the nursing home: development and evaluation of a digital person-centered artistic photo-activity intervention to promote social interaction between residents with dementia, and their formal and informal carers.
Physical activity, health and well-being among a nationally representative population-based sample of middle-aged and older adults in India, 2017-2018.
Sleep problems and subjective cognitive complaints among middle-aged and older adults in 45 low- and middle-income countries.
Transcriptional regulation of neural stem cell expansion in the adult hippocampus.
Wearable GPS and Accelerometer Technologies for Monitoring Mobility and Physical Activity in Neurodegenerative Disorders: A Systematic Review.
Exercise Dosage in Reducing the Risk of Dementia Development: Mode, Duration, and Intensity-A Narrative Review.
SARS-CoV-2 Exacerbates Beta-Amyloid Neurotoxicity, Inflammation and Oxidative Stress in Alzheimer's Disease Patients.
Relationship between Depressive Symptoms and Cognitive, Psychological, and Physical Performance in Community-Dwelling Older People with Cognitive Impairment.
Wearables in rugby union: A protocol for multimodal digital sports-related concussion assessment.
A randomized clinical trial to investigate the effect of dietary protein sources on periodontal health.
Combined Impacts of Physical Activity, Dietary Variety, and Social Interaction on Incident Functional Disability in Older Japanese Adults.
Association of rs3027178 polymorphism in the circadian clock gene PER1 with susceptibility to Alzheimer's disease and longevity in an Italian population.
Heart rate response and recovery during exercise predict future delirium risk-A prospective cohort study in middle- to older-aged adults.
Platelet Total PLA(2) Activity, Serum Oxidative Level, and Plasma Cu/Zn Ratio: A Vicious Cycle with a Potential Role to Monitor MCI and Alzheimer's Disease Progression.
Correction to: Community-level educational attainment and dementia: a 6-year longitudinal multilevel study in Japan.
Effects of Physical and Cognitive Training on Falls and Concern About Falling in Older Adults: Results From a Randomized Controlled Trial.
Tenderness of the knee is associated with thinning of the articular cartilage evaluated with ultrasonography in a community-based cohort: The Nagahama study.
Incidence of and Mortality From Amyotrophic Lateral Sclerosis in National Football League Athletes.
Risk factors for incidence of dementia in primary care practice: a retrospective cohort study in older adults.
The Moo'D Study: protocol for a randomised controlled trial of A2 beta-casein only versus conventional dairy products in women with low mood.
Physical and functional measures predicting long-term mortality in community-dwelling older adults: a comparative evaluation in the Singapore Longitudinal Ageing Study.
The power of genetic diversity in genome-wide association studies of lipids.
A longitudinal investigation of AŒ≤, anxiety, depression, and mild cognitive impairment.
Identifying the specific associations between participation in social activities and healthy lifestyle behaviours in older adults.
Quality of Life and Caregiver Burden of Alzheimer's Disease Among Community Dwelling Patients in Europe: Variation by Disease Severity and Progression.
Leisure Activities and Their Relationship With MRI Measures of Brain Structure, Functional Connectivity, and Cognition in the UK Biobank Cohort.
Anticholinergic medication burden and cognitive function in participants of the ASPREE study.
Accelerometer-Measured, Habitual Physical Activity and Circulating Brain-Derived Neurotrophic Factor: A Cross-Sectional Study.
Ageing rate of older adults affects the factors associated with, and the determinants of malnutrition in the community: a systematic review and narrative synthesis.
Influenza vaccination reduces dementia risk: A systematic review and meta-analysis.
Loneliness and its predictors among older adults prior to and during the COVID-19 pandemic: cross-sectional and longitudinal survey findings from participants of the Atherosclerosis Risk in Communities (ARIC) Study cohort in the USA.
The effect of the 2018 Japan Floods on cognitive decline among long-term care insurance users in Japan: a retrospective cohort study.
Physical Exercise Improves Cognitive Function in Older Adults with Stage 3-4 Chronic Kidney Disease: A Randomized Controlled Trial.
Sex Differences in the Association between Different Obesity Parameters and Cognitive Function in Older Adults: A Cross-Sectional Study in Rural China.
Embelin: A novel XIAP inhibitor for the prevention and treatment of chronic diseases.
Physical and Cognitive Functioning of Institutionalized Elderly People in Rural Areas. Preventive Actions Using Physical Activity and Music Therapy.
Association Between Antemortem FLAIR White Matter Hyperintensities and Neuropathology in Brain Donors Exposed to Repetitive Head Impacts.
A Randomized Controlled Trial of the Effect of Repetitive Transcranial Magnetic Stimulation of the Motor Cortex on Lower Extremity Spasticity in Hereditary Spastic Paraplegia.
Community-level educational attainment and dementia: a 6-year longitudinal multilevel study in Japan.
Self-reported behaviour change among multiple sclerosis community members and interested laypeople following participation in a free online course about multiple sclerosis.
Serum Neurofilament Light Chain as a Marker of Progression in Parkinson's Disease: Long-Term Observation and Implications of Clinical Subtypes.
Bi-allelic variants in OGDHL cause a neurodevelopmental spectrum disease featuring epilepsy, hearing loss, visual impairment, and ataxia.
Exploring Effects of Aerobic Exercise and Mindfulness Training on Cognitive Function in Older Adults at Risk of Dementia: A Feasibility, Proof-of-Concept Study.
Physical Work and Exercise Reduce the Risk of Cognitive Impairment in Older Adults: A Population-based Longitudinal Study.
Physical activity and sleep during the first week of anorexia nervosa inpatient care.
Consumption of coffee and tea and risk of developing stroke, dementia, and poststroke dementia: A cohort study in the UK Biobank.
Effective detection of abnormal gait patterns in Parkinson's disease patients using kinematics, nonlinear, and stability gait features.
Adherence to Mediterranean diet and its associations with circulating cytokines, musculoskeletal health and incident falls in community-dwelling older men: The Concord Health and Ageing in Men Project.

Effects of a physical activity intervention on brain atrophy in older adults at risk of dementia: a randomized controlled trial.
Comparative Value of Cholinesterase Inhibitors and Memantine in Persons with Moderate-to-Severe Alzheimer's Disease in the United States: A Cost-Effectiveness Analysis.
The genetic case for cardiorespiratory fitness as a clinical vital sign and the routine prescription of physical activity in healthcare.
A pilot study of multiple time points and multidomain assessment in cerebrospinal fluid tap test for patients with idiopathic normal pressure hydrocephalus.
Walking While Talking and Prefrontal Oxygenation in Motoric Cognitive Risk Syndrome: Clinical and Pathophysiological Aspects.
High-intensity physical activity is not associated with better cognition in the elder: evidence from the China Health and Retirement Longitudinal Study.
Association of C-Reactive Protein and Motoric Cognitive Risk Syndrome in Community-Dwelling Older Adults: The China Health and Retirement Longitudinal Study.
Improving Patient Outreach by Defining Telehealth Suitability in a Tertiary Huntington's Disease Clinic.
Insulin resistance and muscle weakness are synergistic risk factors for silent lacunar infarcts: the Bunkyo Health Study.
Self-reported cataract surgery and 10-year all-cause and cause-specific mortality: findings from the National Health and Nutrition Examination Survey.
Plasma Glutathione and Prodromal Parkinson's Disease Probability.
Relationship between objectively measured physical activity on neuropathology and cognitive outcomes in older adults: Resistance versus resilience?
Leukocyte-derived ratios are associated with late-life any type dementia: a cross-sectional analysis of the Mugello study.
Developing a Dementia Platform Databank Using Multiple Existing Cohorts.
Association of body mass index and its long-term changes with cardiometabolic diseases: A nationwide twin study.
Subthreshold repetitive transcranial magnetic stimulation drives structural synaptic plasticity in the young and aged motor cortex.
High Glycemic Diet Is Related to Brain Amyloid Accumulation Over One Year in Preclinical Alzheimer's Disease.
The effects of a physical and cognitive training intervention vs. physical training alone on older adults' physical activity: A randomized controlled trial with extended follow-up during COVID-19.
Soft tissue radiodensity parameters mediate the relationship between self-reported physical activity and lower extremity function in AGES-Reykjav√≠k participants.
Targeting Vascular Risk Factors to Reduce Dementia Incidence in Old Age: Extended Follow-up of the Prevention of Dementia by Intensive Vascular Care (preDIVA) Randomized Clinical Trial.
Engaging people with long-term health conditions in a community-based physical activity initiative: a qualitative follow-up study evaluating the parkrun PROVE project.
Effects of 12-Week Supervised Early Resistance Training (SEcReT) Versus Aerobic-Based Rehabilitation on Cognitive Recovery Following Cardiac Surgery via Median Sternotomy: A Pilot Randomised Controlled Trial.
Cerebrovascular Response during Acute Exercise in Kidney Transplant Recipients.
Fish intake, n-3 fatty acid body status, and risk of cognitive decline: a systematic review and a dose-response meta-analysis of observational and experimental studies.
Association of gait with global cognitive function and cognitive domains detected by MoCA-J among community-dwelling older adults: a cross-sectional study.
Highly Crosslinked Polyethylene Liners Have Negligible Wear at 10 Years: A Radiostereometric Analysis Study.
The Japan-Multimodal Intervention Trial for Prevention of Dementia (J-MINT): The Study Protocol for an 18-Month, Multicenter, Randomized, Controlled Trial.
The Cost-Effectiveness of Three Prevention Strategies in Alzheimer's Disease: Results from the Multidomain Alzheimer Preventive Trial (MAPT).
Using the Alzheimer's Disease Neuroimaging Initiative to improve early detection, diagnosis, and treatment of Alzheimer's disease.
Data-driven approaches to executive function performance and structure in aging: Integrating person-centered analyses and machine learning risk prediction.
Disparities in the participation and adherence of older adults in lifestyle-based multidomain dementia prevention and the motivational role of perceived disease risk and intervention benefits: an observational ancillary study to a randomised controlled trial.
Certain dietary patterns are associated with GLIM criteria among Chinese community-dwelling older adults: a cross-sectional analysis.
Cognitive Trajectories and Dementia Risk: A Comparison of Two Cognitive Reserve Measures.
Predictors of short-term impulsive and compulsive behaviour after subthalamic stimulation in Parkinson disease.
Mobility and Depressive Symptoms in Persons With Mild Cognitive Impairment and Alzheimer Dementia.
REPIMPACT - a prospective longitudinal multisite study on the effects of repetitive head impacts in youth soccer.
Risk and protective factors for mental disorders beyond genetics: an evidence-based atlas.
History of Falls, Dementia, Lower Education Levels, Mobility Limitations, and Aging Are Risk Factors for Falls among the Community-Dwelling Elderly: A Cohort Study.
Association of Thyroid Dysfunction With Cognitive Function: An Individual Participant Data Analysis.
Associations of Diet Quality with Midlife Brain Volume: Findings from the UK Biobank Cohort Study.
Interplay between cognition and weight reduction in individuals following a Mediterranean Diet: Three-year follow-up of the PREDIMED-Plus trial.
The Association of Reproductive Aging with Cognitive Function in Sub-Saharan African Women.
AŒ≤ misfolding in blood plasma is inversely associated with body mass index even in middle adulthood.
Urinary proteomics combined with home blood pressure telemonitoring for health care reform trial: rational and protocol.
Association of walking energetics with amyloid beta status: Findings from the Baltimore Longitudinal Study of Aging.
Risk factors for falls in older people with cognitive impairment living in the community: Systematic review and meta-analysis.
Physical Activity Interventions and Their Effects on Cognitive Function in People with Dementia: A Systematic Review and Meta-Analysis.
Dementia and Dependency vs. Proxy Indicators of the Active Ageing Index in Indonesia.
White matter abnormalities in active elite adult rugby players.

Physical activity and cognitive and imaging biomarkers of Alzheimer's disease in down syndrome.
Memorable Food: Fighting Age-Related Neurodegeneration by Precision Nutrition.
A Multi-Omic Huntington's Disease Transgenic Sheep-Model Database for Investigating Disease Pathogenesis.
An Online, Person-Centered, Risk Factor Management Program to Prevent Cognitive Decline: Protocol for A Prospective Behavior-Modification Blinded Endpoint Randomized Controlled Trial.
Engaging older adults in an online physical activity programme to improve cognition: A qualitative study.
Amyotrophic lateral sclerosis transcriptomics reveals immunological effects of low-dose interleukin-2.
Effectiveness of technology-based interventions in detection, prevention, monitoring and treatment of patients at risk or diagnosed with mild cognitive impairment: protocol for a systematic review.
Cognitive stimulation in the workplace, plasma proteins, and risk of dementia: three analyses of population cohort studies.
Biodanza as a Nonpharmacological Dance Movement-Based Treatment in Older People With Alzheimer's Disease: An Italian Pilot Study in 2 Tuscan Nursing Homes.
Urinary metabolomic changes and microbiotic alterations in presenilin1/2 conditional double knockout mice.
A deep learning approach for monitoring parietal-dominant Alzheimer's disease in World Trade Center responders at midlife.
Incidental findings of typical iNPH imaging signs in asymptomatic subjects with subclinical cognitive decline.
Case Report: Probable Cerebral Amyloid Angiopathy-Related Inflammation During Bevacizumab Treatment for Metastatic Cervical Cancer.
Developing methods to detect and diagnose chronic traumatic encephalopathy during life: rationale, design, and methodology for the DIAGNOSE CTE Research Project.
Wrist actigraphic approach in primary, secondary and tertiary care based on the principles of predictive, preventive and personalised (3P) medicine.
Effectiveness of gait aid prescription for improving spatiotemporal gait parameters and associated outcomes in community-dwelling older people: a systematic review.
Air Pollution: A Neglected Risk Factor for Dementia in Latin America and the Caribbean.
Longitudinal Associations between the Neighborhood Built Environment and Cognition in US Older Adults: The Multi-Ethnic Study of Atherosclerosis.
Prominent health problems, socioeconomic deprivation, and higher brain age in lonely and isolated individuals: A population-based study.
Social engagement and cognitive function among middle-aged and older adults: gender-specific findings from the Korean longitudinal study of aging (2008-2018).
Amyloid Burden and Depressive Symptom Trajectories in Older Adults at Risk of Developing Cognitive Decline.
Clinical classification systems and long-term outcome in mid- and late-stage Parkinson's disease.
Heart Rate Variability During Physical Exercise Is Associated With Improved Cognitive Performance in Alzheimer's Dementia Patients-A Longitudinal Feasibility Study.
Focused Ultrasound Thalamotomy for the Treatment of Essential Tremor: A 2-Year Outcome Study of Chinese People.
The effectiveness of cognitive-motor training on reconstructing cognitive health components in older male adults, recovered from the COVID-19.
Neurotrauma investigation through spatial omics guided by mass spectrometry imaging: Target identification and clinical applications.
Prospective associations of chronic and intrusive pain with sarcopenia and physical disability amongst older Australian men: The Concord Health and Ageing in Men Project.
Asymmetric left-right hippocampal glutamatergic modulation of cognitive control in ApoE-isoform subjects is unrelated to neuroinflammation.
An intervention to increase physical activity in care home residents: results of a cluster-randomised, controlled feasibility trial (the REACH trial).
Exercise and the Risk of Dementia in Patients with Newly Diagnosed Atrial Fibrillation: A Nationwide Population-Based Study.
A systems-biology clinical trial of a personalized multimodal lifestyle intervention for early Alzheimer's disease.
Physiologic Frailty and Neurocognitive Decline Among Young-Adult Childhood Cancer Survivors: A Prospective Study From the St Jude Lifetime Cohort.
Timed "Up & Go" Dual-Task Tests: Age- and Sex-Specific Reference Values and Test-Retest Reliability in Cognitively Healthy Controls.
Cardiorespiratory fitness mitigates brain atrophy and cognitive decline in adults at risk for Alzheimer's disease.
Is there a bidirectional association between sedentary behaviour and cognitive decline in older adults? Findings from the Irish Longitudinal Study on Ageing.
Verbal intelligence is a more robust cross-sectional measure of cognitive reserve than level of education in healthy older adults.
Unipolar versus bipolar hemiarthroplasty for hip fractures in patients aged 90 years or older: A bi-centre study comparing 209 patients.
Sensor-based fall risk assessment in older adults with or without cognitive impairment: a systematic review.
Upper limb dysfunction and activities in daily living in idiopathic normal pressure hydrocephalus.
Joint trajectories of episodic memory and odor identification in older adults: patterns and predictors.
Association of Apolipoprotein E …õ4 Allele With Clinical and Multimodal Biomarker Changes of Alzheimer Disease in Adults With Down Syndrome.
From Normal Cognition to Cognitive Impairment and Dementia: Impact of Orthostatic Hypotension.
Sarcopenia and Mild Cognitive Impairment in Older Adults from Six Low- and Middle-Income Countries.
Case Report: Anti-flotillin 1/2 Autoantibody-Associated Atypical Dementia.
Anemia and the Risk of Cognitive Impairment: An Updated Systematic Review and Meta-Analysis.
Multi-Component Intervention Program on Habitual Physical Activity Parameters and Cognitive Function in Patients with Mild Cognitive Impairment: A Randomized Controlled Trial.
A Simple Bedside Exercise Method to Enhance Lower Limb Muscle Strength in Moderate Alzheimer's Disease Patients with Sarcopenia.
Head Injury Exposure in Veterans Presenting to Memory Disorders Clinic: An Observational Study of Clinical Characteristics and Relationship of Event-Related Potentials and Imaging Markers.
[Decreased falls through multifactorial intervention in frail older adults].

Excess Mortality After COVID-19 in Swedish Long-Term Care Facilities.
Physical exercise is effective for neuropsychiatric symptoms in Alzheimer's disease: a systematic review.
Early manifestation of gait alterations in the Tg2576 mouse model of Alzheimer's disease.
Cross-sectional and longitudinal associations between adherence to Mediterranean diet with physical performance and cognitive function in older adults: A systematic review and meta-analysis.
Fitness vs Fatness as Determinants of Survival in Noninstitutionalized Older Adults: The EXERNET Multicenter Study.
Associations Between Polypharmacy and Gait Speed According to Cognitive Impairment Status: Cross-Sectional Study in a Japanese Memory Clinic.
Participation in Collision Sports and Cognitive Aging Among Swedish Twins.
Impact of COVID-19 lockdown on psychosocial factors, health, and lifestyle in Scottish octogenarians: The Lothian Birth Cohort 1936 study.
Exposure to Repetitive Head Impacts Is Associated With Corpus Callosum Microstructure and Plasma Total Tau in Former Professional American Football Players.
Cerebral Small Vessel Disease Associated With Atrial Fibrillation Among Older Adults: A Population-Based Study.
Implementation Science in Thailand: Design and Methods of a Geriatric Mental Health Cluster-Randomized Trial.
Subjective and objective health predicting mortality and institutionalization: an 18-year population-based follow-up study among community-dwelling Finnish older adults.
Risk Assessment During Longitudinal Progression of Cognition in Older Adults: A Community-based Bayesian Networks Model.
Impact of sex and APOE Œµ4 on age-related cerebral perfusion trajectories in cognitively asymptomatic middle-aged and older adults: A longitudinal study.
A novel cognitive-motor exercise program delivered via a tablet to improve mobility in older people with cognitive impairment - StandingTall Cognition and Mobility.
Physical Activity as a Predictor of Cognitive Decline in an Elderly Essential Tremor Cohort: A Prospective, Longitudinal Study.
Interaction Between APOE Genotype and Diabetes in Longevity.
Mind Diet Adherence and Cognitive Performance in the Framingham Heart Study.
Community social support and onset of dementia in older Japanese individuals: a multilevel analysis using the JAGES cohort data.
Nutrition Module design in Maintain Your Brain: an internet-based randomised controlled trial to prevent cognitive decline and dementia.
Plasma microRNAs are associated with domain-specific cognitive function in people with HIV.
Obstructive Sleep Apnea and Cognitive Decline: A Review of Potential Vulnerability and Protective Factors.
Associations between physical activity types and multi-domain cognitive decline in older adults from the Three-city cohort.
Longitudinal Cognitive and Physical Training Effectiveness in MCI, Based on the Experience of the Alzheimer's Hellas Day Care Centre.
[The psychological process and its longitudinal of family caregivers involved in associations for families of dementia patients].
Circadian rhythm of ischaemic core progression in human stroke.
Non-pharmacological interventions a feasible option for addressing dementia-related sleep problems in the context of family care.
Measuring resting cerebral haemodynamics using MRI arterial spin labelling and transcranial Doppler ultrasound: Comparison in younger and older adults.
A Western-style dietary pattern is associated with cerebrospinal fluid biomarker levels for preclinical Alzheimer's disease-A population-based cross-sectional study among 70-year-olds.
Change in modifiable dementia risk factors during COVID-19 lockdown: The experience of over 50s in Tasmania, Australia.
Identifying degenerative effects of repetitive head trauma with neuroimaging: a clinically-oriented review.
Obesity is associated with reduced cerebral blood flow - modified by physical activity.
Long-term Intake of Gluten and Cognitive Function Among US Women.
The joint association of physical activity and fine particulate matter exposure with incident dementia in elderly Hong Kong residents.
Sleep Timing and Risk of Dementia Among the Chinese Elderly in an Urban Community: The Shanghai Aging Study.
Predicting outcome following mild traumatic brain injury: protocol for the longitudinal, prospective, observational Concussion Recovery (CREST) cohort study.
Exploring the association of early life physical activity and risk of dementia: a systematic review.
Feasibility of reducing frailty components in older adults with Alzheimer's dementia: a randomized controlled home-based exercise trial (AD-HOMEX).
Detecting Sensitive Mobility Features for Parkinson's Disease Stages Via Machine Learning.
Exercise, Arterial Stiffness, and Cerebral Vascular Function: Potential Impact on Brain Health.
Profiling Social Cognition in Premanifest Huntington's Disease.
Association of Life's Simple 7 with incident dementia and its modification by the apolipoprotein E genotype.
Risk factors and consequences of depression in later life: Findings from the health in men study (HIMS).
The Association between Physical Activity and CAMDEX-DS Changes Prior to the Onset of Alzheimer's Disease in Down Syndrome.
Needs of Alzheimer's Charges' Caregivers in Poland in the Covid-19 Pandemic-An Observational Study.
Possible Neuroprotective Effects of l-Carnitine on White-Matter Microstructural Damage and Cognitive Decline in Hemodialysis Patients.
Age-dependent instability of mature neuronal fate in induced neurons from Alzheimer's patients.
Changes in executive function and gait in people with mild cognitive impairment and Alzheimer disease.
Early life involvement in C9orf72 repeat expansion carriers.
Memory enhancement by multidomain group cognitive training in patients with Parkinson's disease and mild cognitive impairment: long-term effects of a multicenter randomized controlled trial.
Physical and cognitive profiles in motoric cognitive risk syndrome in an older population from Southern Italy.

Tau aggregation and increased neuroinflammation in athletes after sports-related concussions and in traumatic brain injury patients - A PET/MR study.
Motoric cognitive risk syndrome, physio-cognitive decline syndrome, cognitive frailty and reversibility with dual-task exercise.
Cortical Thickness, Volume, and Surface Area in the Motoric Cognitive Risk Syndrome.
Hemodynamic and structural brain measures in high and low sedentary older adults.
Late life psychotic features in prodromal Parkinson's disease.
Multi-ancestry genome-wide gene-sleep interactions identify novel loci for blood pressure.
Efficacy of Tai Chi-Style Multi-Component Exercise on Frontal-Related Cognition and Physical Health in Elderly With Amnestic Mild Cognitive Impairment.
Sex differences in brain aging among adults with family history of Alzheimer's disease and APOE4 genetic risk.
Proteomic analysis of aged and OPTN E50K retina in the development of normal tension glaucoma.
The Survey of the Health of Wisconsin (SHOW) Program: An infrastructure for Advancing Population Health Sciences.
Gains in insurance coverage following the affordable care act and change in preventive services use among non-elderly US immigrants.
Association of sex differences in dementia risk factors with sex differences in memory decline in a population-based cohort spanning 20-76¬†years.
Is neuroticism differentially associated with risk of Alzheimer's disease, vascular dementia, and frontotemporal dementia?
Neuroticism and Risk of Parkinson's Disease: A Meta-Analysis.
Prevention of cognitive decline in subjective cognitive decline APOE Œµ4 carriers after EGCG and a multimodal intervention (PENSA): Study design.
Blood Pressure and Risk of Cognitive Impairment: The Role of Vascular Disease in Neurodegeneration.
Prevalence, Lifestyle Correlates, and Psychosocial Functioning Among Multi-Ethnic Older Adults with Mild Cognitive Impairment in Singapore: Preliminary Findings from a 10/66 Population Study.
Omega-3 Long-Chain Polyunsaturated Fatty Acids: Are They Beneficial for Physical and Cognitive Functioning in Older Adults?
Efficacy of Multidomain Intervention Against Physio-cognitive Decline Syndrome: A Cluster-randomized Trial.
Association between lifetime depression history, hippocampal volume and memory in non-amnestic mild cognitive impairment.
Hair and salivary cortisol and their relationship with lifestyle, mood and cognitive outcomes in premanifest Huntington's disease.
Longitudinal associations between exposure to anesthesia and neurocognitive functioning in pediatric medulloblastoma.
Dorsolateral Prefrontal Functional Connectivity Predicts Working Memory Training Gains.
Publisher Correction: Discovery of rare variants associated with blood pressure regulation through meta-analysis of 1.3 million individuals.
Neuropsychiatric symptoms and the outcome of cognitive trajectories in older adults free of dementia: The Mayo Clinic Study of Aging.
Depression in Alzheimer's Disease: A Delphi Consensus on Etiology, Risk Factors, and Clinical Management.
Frequency and Predictors of Traumatic Encephalopathy Syndrome in a Prospective Cohort of Retired Professional Athletes.
A Novel Program to Improve Cognitive Function in Individuals With Dementia Using Transcranial Alternating Current Stimulation (tACS) and Tutored Cognitive Exercises.
27-Hydroxycholesterol, cognition, and brain imaging markers in the FINGER randomized controlled trial.
Association between Sour Taste SNP KCNJ2-rs236514, Diet Quality and Mild Cognitive Impairment in an Elderly Cohort.
Associations between activity fragmentation and subjective memory complaints in middle-aged and older adults.
Body weight changes and longitudinal associations with cognitive decline among community-dwelling older adults.
The association between objective vision impairment and mild cognitive impairment among older adults in low- and middle-income countries.
Application of Cerebrospinal Fluid AT(N) Framework on the Diagnosis of AD and Related Cognitive Disorders in Chinese Han Population.
Gait Speed and Instrumental Activities of Daily Living in Older Adults After Hospitalization: A Longitudinal Population-Based Study.
Risk Factors for Incident Falls and Fractures in Older Men With and Without Type 2 Diabetes Mellitus: The Concord Health and Ageing in Men Project.
COllaborative Neuropathology NEtwork Characterizing ouTcomes of TBI (CONNECT-TBI).
Responsiveness and interpretability of commonly used outcome assessments of mobility capacity in older hospital patients with cognitive spectrum disorders.
Bidirectional Association between Visual Impairment and Dementia Among Older Adults in the United States Over Time.
Specific Activities and the Trajectories of Cognitive Decline Among Middle-Aged and Older Adults: A Five-Year Longitudinal Cohort Study.
Nonmotor symptom burden grading as predictor of cognitive impairment in Parkinson's disease.
Changes in Lifestyle, Behaviors, and Risk Factors for Cognitive Impairment in Older Persons During the First Wave of the Coronavirus Disease 2019 Pandemic in Finland: Results From the FINGER Study.
CSF and Blood Neurofilament Levels in Athletes Participating in Physical Contact Sports: A Systematic Review.
Physical Performance and Cognition in a Diverse Cohort: Kaiser Healthy Aging and Diverse Life Experiences (KHANDLE) Study.
Associations of the Mediterranean-Dietary Approaches to Stop Hypertension Intervention for Neurodegenerative Delay diet with cardiac remodelling in the community: the Framingham Heart Study.
Temporal emergence of age-associated changes in cognitive and physical function in vervets (Chlorocebus aethiops sabaeus).
Prospective Role of Polyphenolic Compounds in the Treatment of Neurodegenerative Diseases.
COmmuNity-engaged SimULation Training for Blood Pressure Control (CONSULT-BP): A study protocol.
Validation of a Machine Learning Brain Electrical Activity-Based Index to Aid in Diagnosing Concussion Among Athletes.
Cost-Effectiveness of Dementia Prevention Interventions.
Cognitive reserve in amyotrophic lateral sclerosis (ALS): a population-based longitudinal study.
Does Low Subjective Social Status Predict Cognitive Decline in Chinese Older Adults? A 4-Year Longitudinal Study From Hong Kong.

Feasibility and acceptability of a multi-domain intervention to increase Mediterranean diet adherence and physical activity in older UK adults at risk of dementia: protocol for the MedEx-UK randomised controlled trial.
Motor function is the primary driver of the associations of sarcopenia and physical frailty with adverse health outcomes in community-dwelling older adults.
Pre-stroke physical activity and admission stroke severity: A systematic review.
Shared risk and protective factors between Alzheimer's disease and ischemic stroke: A population-based longitudinal study.
High-intensity exercise and cognitive function in cognitively normal older adults: a pilot randomised clinical trial.
Assessment of causal effects of physical activity on neurodegenerative diseases: A Mendelian randomization study.
Efficacy of a Smart Antisnore Pillow in Patients with Obstructive Sleep Apnea Syndrome.
Genome-wide meta-analysis of muscle weakness identifies 15 susceptibility loci in older men and women.
Evaluation of Wearable Technology in Dementia: A Systematic Review and Meta-Analysis.
Cognitive reserve and brain maintenance in aging and dementia: An integrative review.
Evidence of association between obesity and lower cerebral myelin content in cognitively unimpaired adults.
Longitudinal Reproducibility of Neurite Orientation Dispersion and Density Imaging (NODDI) Derived Metrics in the White Matter.
Disorientation in Time and Place in Old Age: Longitudinal Evidence from Three Old Age Cohorts in Germany (AgeDifferent.de Platform).
How Does Participation in Formal Education or Learning for Older People Affect Wellbeing and Cognition? A Systematic Literature Review and Meta-Analysis.
Targeted metabolomics analysis of postoperative delirium.
Effect of aerobic exercise on amyloid accumulation in preclinical Alzheimer's: A 1-year randomized controlled trial.
Imaging-based indices of Neuropathology and gait speed decline in older adults: the atherosclerosis risk in communities study.
Anterior callosal angle correlates with gait impairment and fall risk in iNPH patients.
Stacked autoencoders as new models for an accurate Alzheimer's disease classification support using resting-state EEG and MRI measurements.
Synthesis and Biological Evaluation of Benzimidazole Derivatives as Potential Neuroprotective Agents in an Ethanol-Induced Rodent Model.
An Active Lifestyle Is Associated with Better Cognitive Function Over Time in APOE …õ4 Non-Carriers.
Glutathione Peroxidase Activity Is Altered in Vascular Cognitive Impairment-No Dementia and Is a Potential Marker for Verbal Memory Performance.
[Endoprosthetic treatment of pertrochanteric femoral fractures with concomitant coxarthrosis : A consecutive observational study with a control group].
Type 2 diabetes mellitus-associated transcriptome alterations in cortical neurones and associated neurovascular unit cells in the ageing brain.
Associations between metabolic syndrome and type of dementia: analysis based on the National Health Insurance Service database of Gangwon province in South Korea.
The insulin resistance by triglyceride glucose index and risk for dementia: population-based study.
Differential effects of sex on longitudinal patterns of cognitive decline in Parkinson's disease.
Influence of Cardiorespiratory Fitness on Risk of Dementia and Dementia Mortality: A Systematic Review and Meta-Analysis of Prospective Cohort Studies.
Association Between Dietary Fish and PUFA Intake in Midlife and Dementia in Later Life: The JPHC Saku Mental Health Study.
Influence of a¬†multidimensional music-based exercise program on selected cognitive and motor skills in dementia patients-a¬†pilot study.
Psychotropic Medication and Cognitive, Functional, and Neuropsychiatric Outcomes in Alzheimer's Disease (AD).
Analysis of Risk Factors in Dementia Through Machine Learning.
Mental stress-induced myocardial ischemia and cognitive impairment in coronary atherosclerosis.
Effects of physical activity on brain function and structure in older adults: A systematic review.
The sleep and circadian problems of Huntington's disease: when, why and their importance.
Nut Consumption for Cognitive Performance: A Systematic Review.
Sensitivity and Specificity of Computer-Based Neurocognitive Tests in Sport-Related Concussion: Findings from the NCAA-DoD CARE Consortium.
Vascular health across young adulthood and midlife cerebral autoregulation, gait, and cognition.
Recovery Profiles after Concussion among Male Student-Athletes and Service Cadets with a Family History of Neurodegenerative Disease: Data from the NCAA-DoD CARE Consortium.
Balance and gait performance in older adults with early-stage cognitive impairment.
[Potentials and limits of aging cohort studies for geriatric psychiatry].
Association of sleep disturbance and freezing of gait in Parkinson disease: prevention/delay implications.
Physical Activity and Trajectory of Cognitive Change in Older Persons: Mayo Clinic Study of Aging.
Exercise SBP response and incident depressive symptoms: The Maastricht Study.
What do we know about the risks of developing dementia after traumatic brain injury?
Cognitive and neuroimaging profiles of older adults with dual decline in memory and gait speed.
Retinal Microvascular Alterations as the Biomarkers for Alzheimer Disease: Are We There Yet?
Suicide risk within 1 year of dementia diagnosis in older adults: a nationwide retrospective cohort study.
Cognitive effects and acceptability of non-invasive brain stimulation on Alzheimer's disease and mild cognitive impairment: a component network meta-analysis.
Body mass index and risk of Parkinson, Alzheimer, Dementia, and Dementia mortality: a systematic review and dose-response meta-analysis of cohort studies among 5 million participants.
The Importance of Engaging in Physical Activity in Older Adulthood for Transitions Between Cognitive Status Categories and Death: A Coordinated Analysis of 14 Longitudinal Studies.

Plasma GSH levels and Alzheimer's disease. A prospective approach.: Results from the HELIAD study.
Association of anthropometric and nutrition status indicators with cognitive functions in centenarians.
Association of Cortical and Subcortical Œ≤-Amyloid With Standardized Measures of Depressive and Anxiety Symptoms in Adults Without Dementia.
Age-dependency in mortality of family caregivers: a nationwide register-based study.
The effect of occupational physical activity on dementia: Results from the Copenhagen Male Study.
Immune reconstitution and clinical recovery following anti-CD28 antibody (TGN1412)-induced cytokine storm.
A Genome-wide Association Study for Concussion Risk.
Promoting Active Aging: Lessons Learned in an Implementation Pilot in Primary Care.
Physical activity may not be associated with long-term risk of dementia and Alzheimer's disease.
Cultural Engagement and Incidence of Cognitive Impairment: A 6-year Longitudinal Follow-up of the Japan Gerontological Evaluation Study (JAGES).
Slowed driving-reaction time following concussion-symptom resolution.
Predicting Short-Term Risk of Falls in a High-Risk Group With Dementia.
PER2 C111G polymorphism, cognitive reserve and cognition in subjective cognitive decline and mild cognitive impairment: a 10-year follow-up study.
Dietary diversity is associated with longitudinal changes in hippocampal volume among Japanese community dwellers.
Feasibility and acceptability evaluation of the Promoting Independence in Dementia (PRIDE) intervention for living well with dementia.
Underlying genetic variation in familial frontotemporal dementia: sequencing of 198 patients.
Earlier life leisure-time physical activity in relation to age-related frailty syndrome.
Gait speed, life-space mobility and mild cognitive impairment in patients with coronary artery disease.
Frailty and Prodromal Parkinson's Disease: Results From the HELIAD Study.
A Randomized Controlled Trial of High-Intensity Exercise and Executive Functioning in Cognitively Normal Older Adults.
A New Look at Cohort Trend and Underlying Mechanisms in Cognitive Functioning.
The ENIGMA sports injury working group:- an international collaboration to further our understanding of sport-related brain injury.
Comprehensive assessment of levodopa-carbidopa intestinal gel for Turkish advanced Parkinson,Äôs disease patients.
Primary hemiarthroplasty for the elderly patient with cognitive dysfunction and a displaced femoral neck fracture: a prospective, observational cohort study.
Plasma metabolites associated with biomarker evidence of neurodegeneration in cognitively normal older adults.
Function-based dementia severity assessment for vascular cognitive impairment.
Brain Regional Glucose Metabolism, Neuropsychiatric Symptoms, and the Risk of Incident Mild Cognitive Impairment: The Mayo Clinic Study of Aging.
Does the Impact of Intensive Lifestyle Intervention on Cardiovascular Disease Risk Vary According to Frailty as Measured via Deficit Accumulation?
The Effects of Tau, Amyloid, and White Matter Lesions on Mobility, Dual Tasking, and Balance in Older People.
The Great Escape. Centenarians' exceptional health.
Vegetarianism and veganism compared with mental health and cognitive outcomes: a systematic review and meta-analysis.
Participation in cognitive activities is associated with foot reaction time and gait speed in older adults.
Gene-educational attainment interactions in a multi-ancestry genome-wide meta-analysis identify novel blood pressure loci.
Patient-related and anesthesia-dependent determinants for postoperative delirium after oral and maxillofacial surgery. Results from a register-based case-control study.
Effectiveness of Dementia Care Mapping,Ñ¢ to reduce agitation in care home residents with dementia: an open-cohort cluster randomised controlled trial.
Spatial-Temporal Parameters of Gait Associated With Alzheimer Disease: A Longitudinal Analysis.
Towards an active and happy retirement? Changes in leisure activity and depressive symptoms during the retirement transition.
The relationship between social cognition and participation in the long term after stroke.
Music Training Improves Depressed Mood Symptoms in Elderly People: A Randomized Controlled Trial.
Local Health Jurisdiction Staff Deliver Health Promotion to Small Worksites, Washington.
Adherence to a Mediterranean diet is associated with cognitive function in an older non-Mediterranean sample: findings from the Maine-Syracuse Longitudinal Study.
Engagement and experience of older people with socially assistive robots in home care.
Association of Medial Temporal Lobe Cerebrovascular Reactivity and Memory Function in Older Adults With and Without Cognitive Impairment
Vitality index predicts walking independence in patients with hip fracture A retrospective study
Sleep Disordered Breathing and Subsequent Neuroimaging Markers of Brain Health in Hispanic/Latino Adults
Plasma uric acid levels and risk of dementia in a population-based cohort study
Disentangling the Effects of Comorbidity and Polypharmacy on Cognitive Function and Physical Frailty in Individuals With HIV
Blood Pressure Variability and Cognition in Black and White Older Adults Over 18 Years of Follow-up A Population-Based Cohort Study
Integrating accelerometry, GPS, GIS and molecular data to investigate mechanistic pathways of the urban environmental exposome and cognitive outcomes in older adults: a longitudinal study protocol
Health and Lifestyle Factors and Dementia Risk among Former Professional Soccer Players
Duration of Ice Hockey Play and Chronic Traumatic Encephalopathy
Patient-Relevant Digital-Motor Outcomes for Clinical Trials in Hereditary Spastic Paraplegia Type 7: A Multicenter PROSPAX Study

Functional Dependency as a Marker for Positive SARC-F Screen among Older Persons at the Emergency Department
Risk of Stroke or Heart Attack in Mild Cognitive Impairment and Subjective Cognitive Impairment
Stumbles, Gait, and Cognition: Risk Factors Associated with Falls in Older Adults with Subjective Memory Complaints
Witnessing Self-Affirming Moments in Persons with Dementia While Interacting with Therapy Dogs: A Case Report
Identification of Causal Genes and Potential Drug Targets for Restless Legs Syndrome: A Comprehensive Mendelian Randomization Study
Association of immune-mediated diseases with the risk of dementia and brain structure in UK Biobank participants
Effectiveness and feasibility of a theory-informed intervention to improve Mediterranean diet adherence, physical activity and cognition in older adults at risk of dementia: the MedEx-UK randomised controlled trial
Associations among healthy lifestyle characteristics, neuroinflammation, and cerebrospinal fluid core biomarkers of Alzheimer's disease in cognitively intact adults: The CABLE study
Structured tailored rehabilitation after hip fragility fracture: The 'Stratify' feasibility and pilot randomised controlled trial protocol
Health disparities in the risk of severe acidosis: real-world evidence from the All of Us cohort
Proactive frailty in primary care: A primary care system fit for frailty
Association of Tailpipe-Related and Nontailpipe‚ÄìRelated Air Pollution Exposure with Cognitive Decline in the Chicago Health and Aging Project
Diets and leisure activities are associated with curiosity
Machine learning based metabolomic and genetic profiles for predicting multiple brain phenotypes
Body Roundness Index Trajectories and the Risk of Cancer: A Cohort Study
Multi-Trajectories of Intrinsic Capacity Decline and Their Impact on Age-Related Outcomes: A 20-Year National Longitudinal Cohort Study
The Comprehensive Resilience-building psychosocial Intervention (CREST) for people with dementia in the community: a feasibility and acceptability study
Estimation of physiological aging based on routine clinical biomarkers: a prospective cohort study in elderly Chinese and the UK Biobank
Associations between sex and lifestyle activities with cognitive reserve in mid-life adults with genetic risk for Alzheimer‚Äôs disease
Distinctive Gait Variations and Neuroimaging Correlates in Alzheimer's Disease and Cerebral Small Vessel Disease
A comprehensive evaluation on the associations between hearing and vision impairments and risk of all-cause and cause-specific dementia: results from cohort study, meta-analysis and Mendelian randomization study
Association of cognitive performance with overall, dosage, intensity, and domain physical activity in aging: NHANES 2011‚Äì2014
Lifestyle empowerment for Alzheimer's prevention prescribed by physicians: Methods and adaptations to COVID-19
Leisure-time physical activity, cardiorespiratory fitness, and cognitive decline in middle-aged and older adults - The ELSA-Brasil study
Verbal training can improve neurocognitive and reading performance by increasing white matter integrity and grey matter volume
Association between childhood parental literacy and late-life cognitive function: The Guangzhou Biobank Cohort Study
A single risk assessment for the most common diseases of ageing, developed and validated on 10 cohort studies
Observational and genetic associations between cardiorespiratory fitness and age-related diseases: longitudinal analyses in the UK Biobank study
The association between voluntary health insurance and health outcomes in older adults in Europe: A survival analysis
The relationship between micronutrients and cognitive ability in an elderly population with mild cognitive impairment and Alzheimer‚Äôs disease: a cross-sectional study
Physical activity, amyloid-Œ≤, and tau protein: A systematic review and meta-analysis
Childhood maltreatment, cognitive performance, and cognitive decline in middle-aged and older adults with chronic disease: A prospective study
Exploring Chronic Pain Patterns and Associations With All-Cause Dementia: Results From UK Biobank
Prescription of Nonpharmacologic Interventions in Memory Clinics: Data from the Clinical Pathway for Alzheimer's Disease in China (CPAD) Study
Artificial Intelligence for Prediction of Shunt Response in Idiopathic Normal Pressure Hydrocephalus: A Systematic Review
Association of MIND diet with cognitive decline among Black¬†and White older adults
Prospective study of bipolar disorder and neurodegenerative diseases
Effects of depression and cognitive impairment on increased risks of incident dementia: a prospective study from three elderly cohorts
Integrating the Memory Support Intervention into the Transdiagnostic Intervention for Sleep and Circadian Dysfunction (TranS-C): can improving memory for treatment in midlife and older adults improve patient outcomes? Study protocol for a randomized controlled trial
Associations of metabolic syndrome with cognitive function and dementia risk: Evidence from the UK Biobank cohort
Associations of combined accelerated biological aging and genetic susceptibility with incident dementia: a prospective study in the UK Biobank
Higher scores of the Kessler Psychological Distress Scale (K10) are associated with lower serum ergothioneine and higher serum asymmetric dimethyl-L-arginine concentrations in a cohort of middle-aged and older adults
Predictors of symptom improvement in patients with chronic coronary syndrome after percutaneous coronary intervention
Toxoplasma Gondii infection and cardiovascular mortality: sex-specific differences in a United States population-based cohort study
Effects of physical and psychological multimorbidity on the risk of dementia: multinational prospective cohorts and a meta-analysis
Transition of Physical, Psychological, and Cognitive Frailty and Its‚Äô Associated Determinants in Malaysian Older Adults: A 5-Year Follow-up Study
Impaired health-related QoL perception is associated with hospitalization in patients undergoing peritoneal dialysis
Cross-Sectional and Prospective Associations between Parkinsonism and Parkinson's Disease with Frailty in Latin America
Association of oral frailty and gait characteristics in patients with cerebral small vessel disease
Sleep fragmentation despite intact rest-activity patterns in premanifest Huntington's disease: An actigraphy study
Effects of oral health interventions on cognition of people with dementia: a systematic review with meta-analysis

Clinical implications of head trauma in frontotemporal dementia and primary progressive aphasia
The evaluation of nightshade elimination diet (NED) on inflammatory and rheumatologic markers of rheumatoid arthritis patients: study protocol for a randomized controlled trial
Change in Cardiorespiratory Fitness and Risk of Depression, Anxiety, and Cerebrovascular Disease
Older adults exercising ON TIME: protocol for a randomized controlled cross-over study to assess the effect of physical activity timing on insomnia severity
Dementia with Lewy bodies and gait neural basis: a cross-sectional study
Plasma biomarkers of neurodegeneration in patients and high risk subjects with Lewy body disease
Prevalence and population attributable fractions of potentially modifiable risk factors for dementia in Canada: A cross-sectional analysis of the Canadian Longitudinal Study on Aging
Associations of sugar intake, high-sugar dietary pattern, and the risk of dementia: a prospective cohort study of 210,832 participants
Effects of bottom-up versus top-down digital cognitive training in older adults: A randomized controlled trial
Risk and protective factors for cognitive decline in Brazilian lower educated older adults: A 15-year follow-up study using group-based trajectory modelling
The performance of metabolomics-based prediction scores for mortality in older patients with solid tumors
PEMOCS: Evaluating the effects of a concept-guided, PErsonalised, MOtor-Cognitive exergame training on cognitive functions and gait in chronic Stroke,Äîstudy protocol for a randomised controlled trial
Nutrition guidance within a multimodal intervention improves diet quality in prodromal Alzheimer,Äôs disease: Multimodal Preventive Trial for Alzheimer,Äôs Disease (MIND-ADmini)
Temporal trends in population attributable fractions of modifiable risk factors for dementia: a time-series study of the English Longitudinal Study of Ageing (2004,Äì2019)
Cognitive training and promoting a healthy lifestyle for individuals with isolated REM sleep behavior disorder: study protocol of the delayed-start randomized controlled trial CogTrAiL-RBD
Social isolation and the risk of Parkinson disease in the UK biobank study
Associations of serum uric acid variability with neuroimaging metrics and cognitive decline: a population-based cohort study
Exercise: Just What the Doctor Ordered, But Why? Elucidating Mechanisms for Women,Äôs Increased High-Density Lipoprotein Benefit From Exercise and for the Health ABC Study
Integrating a multimodal lifestyle intervention with medical food in prodromal Alzheimer,Äôs disease: the MIND-ADmini randomized controlled trial
Physical frailty, genetic predisposition, and incident dementia: a large prospective cohort study
White matter alterations and their associations with biomarkers and behavior in subjective cognitive decline individuals: a fixel-based analysis
Spontaneous miscarriage and social support in predicting risks of depression and anxiety: a cohort study in UK Biobank
Improving mental health in chronic care in general practice: study protocol for a cluster-randomised controlled trial of the Healthy Mind intervention
Exploring vascular contributions to cognitive impairment and dementia (ENIGMA): protocol for a prospective observational study
Development and validation of cognitive ageing risk score (CARS) for early detection of subtle cognitive deficits in older people
Daoyin therapy in chronic neck pain: study protocol for a randomized controlled trial
Investigating the nexus of metabolic syndrome, serum uric acid, and dementia risk: a prospective cohort study
Effects of neighborhood built environment on cognitive function in older adults: a systematic review
Cognitive impairment as a predictor of long-term psychological distress in patients with polysubstance use disorders: a prospective longitudinal cohort study
Impaired 24-h activity patterns are associated with an increased risk of Alzheimer,Äôs disease, Parkinson,Äôs disease, and cognitive decline
Correlation between preoperative CT scan of the paraspinal, psoas, and gluteus muscles and postoperative ambulatory status in patients with femoral neck fractures
Factors affecting cognitive frailty improvement and progression in Taiwanese older adults
An Investigation into the Relationship of Circulating Gut Microbiome Molecules and Inflammatory Markers with the Risk of Incident Dementia in Later Life
Baseline Characteristics of Participants in STAREE: A Randomized Trial for Primary Prevention of Cardiovascular Disease Events and Prolongation of Disability-Free Survival in Older People
Data-driven sequence of cognitive decline in people with Parkinson's disease
Association of Sleep Disturbances With Prevalent and Incident Motoric Cognitive Risk Syndrome in Community-Residing Older Adults
Associations Between Atherosclerosis and Subsequent Cognitive Decline: A Prospective Cohort Study
The effect of dietary magnesium intake on cognitive decline related to olfactory impairment in older adults: a cross-sectional study from the NHANES database
The partner paradox: How can we better understand shared cognitive decline in couples?
The Relationship Between Dietary Patterns, Cognition, and Cardiometabolic Health in Healthy, Older Adults
Personality Feedback With Tailored Self-Care Recommendations Improves Self-Efficacy for Cancer Management: A Randomized Controlled Trial
Nut consumption and disability-free survival in community-dwelling older adults: a prospective cohort study
Association between Alzheimer,Äôs disease and Toxocara infection/exposure: a case,Äìcontrol study
Mendelian randomization studies in ankylosing spondylitis: A systematic review
Self-reported physical activity and gait in older adults without dementia: A longitudinal study
Cognitive status and demographics modify the association between subjective cognition and amyloid
Predicting physical functioning status in older adults: insights from wrist accelerometer sensors and derived digital biomarkers of physical activity
APOE Œµ4 carrier status moderates the effect of lifestyle factors on cognitive reserve
A multilayer network analysis of Alzheimer's disease pathogenesis: Roles for p-tau, synaptic peptides, and physical activity

Tachypnea in response to hypoxemia decreases with age in older patients
Insights into the associated risk factors of malnutrition among nursing home residents: A longitudinal study
The association between selenium status and global and attention-specific cognition in very old adults in the Newcastle 85+ Study: cross-sectional and longitudinal analyses
Lumbar puncture tap test in iNPH: Does extracting different volumes of CSF change the clinical response?
Brain hemodynamic responses and fall prediction in older adults with multiple sclerosis
Combining muscle strengthening activity and aerobic exercise: a prescription for better health in patients with hypertension
Mortality in people living with dementia who self-harmed: An Australian data linkage study
Factors associated with cognitive reserve according to education level
Independent and joint role of inflammatory diet and physical activity with cognitive function in aging: Evidence from a population-based survey
Cumulative sum (CUSUM) analysis: A 10 year experience of outcome monitoring in a regional burn centre
Evaluating the safety and effectiveness of direct oral anticoagulants compared with warfarin in very elderly patients with atrial fibrillation with and without low bodyweight
General Principles, Etiologies, Evaluation, and Management in Older Adults
Comparison of the effect of online physical exercise and computerized cognitive stimulation in patients with Alzheimer's disease during the Covid-19 pandemic
A nomogram for predicting disability-free survival in older adults over 15 years: A population-based cohort study
Diabetes Microvascular Complications Are Associated with a Reduction in Gait Speed in High-Functioning Older Adults for Outpatient Clinics
Unveiling DNA methylation in Alzheimer,Äôs disease: a review of array-based human brain studies
Association Between Dietary Antioxidant Quality Score (DAQS) and All-Cause Mortality in Hypertensive Adults: A Retrospective Cohort Study from the NHANES Database
Association between whole egg consumption and cognitive function in older adults: Birjand longitudinal aging study
Special issue on ,ÄúA focus on brain,Äìbody communication in understanding the neurobiology of diseases,Äù
Changes in Alzheimer Disease Blood Biomarkers and Associations with Incident All-Cause Dementia
The interaction between physical activity and ambient particulate matters on cognitive function among Chinese community-dwelling older adults
Study on the correlation between healthy lifestyle practices and stroke in elderly people
Forget About Memory: Disentangling the Amnestic Syndrome in Idiopathic Normal Pressure Hydrocephalus
Physical Activity in Mild Vascular Cognitive Impairment: Results of the AFIVASC Randomized Controlled Trial at 6 Months
Clinical Factors Affecting the Remaining Activity of Daily Living in Severe Dementia
Thinking outside the brain: Gut microbiome influence on innate immunity within neurodegenerative disease
Estimating the Prevalence of Cognitive Impairment and Its Associated Factors in Albania: A Nationwide Cross-Sectional Study
Prevalence and associated factors of dyslipidemia in elderly people: Results from the Ardakan Cohort Study on Ageing, Iran
Inflammation in Older Poles with Localized and Widespread Chronic Pain,ÄîResults from a Population-Based PolSenior Study
Reduction in the incidence of cognitive impairment and related costs through an innovative health awareness programme in rural Japan
The impact of education as a proxy for lifestyle habits on reducing the association with dementia prevalence in the Southern Region of Brazil
Comment on ,ÄúThe Relationship Between Antipsychotics, Cognitive Enhancers, and Major Adverse Cardiovascular/Cerebrovascular Events (MACCE) in Older Adults with Behavioral and Psychological Symptoms of Dementia,Äù
Total physical activity matters for brain health
Pre-Admission Predictors of Walking Independence in Critically Ill Patients
Awareness of Being Prescribed Antihypertensive Medications and Cardiovascular Outcomes
Independent and joint associations of cardiometabolic multimorbidity and depression on cognitive function: findings from multi-regional cohorts and generalisation from community to clinic
Characteristics of fragility hip fracture-related falls in the older adults: A systematic review
Undernutrition, cognitive decline and dementia: The collaborative PROMED-COG pooled cohorts study
Association between alcohol consumption and incidence of dementia in current drinkers: linear and non-linear mendelian randomization analysis
Post-Acute Care Rehabilitation for Persons Living With Dementia: A Systematic Review
Sex differences in reduction of trunk muscle mass related to falls and cognitive function during the COVID-19 pandemic in older adults
Psychosocial Health and the Association Between Cerebral Small Vessel Disease Markers With Dementia: The ARIC Study
Blood-based multivariate methylation risk score for cognitive impairment and dementia
Risk factor of disability as new certification of long-term care needs in older Japanese adults with diabetes mellitus: A longitudinal study
AAV-aMTD-Parkin, a therapeutic gene delivery cargo, enhances motor and cognitive functions in Parkinson's and Alzheimer's diseases
The effect of deprescribing interventions on mortality and health outcomes in older people: An updated systematic review and meta-analysis
Histidine-containing dipeptide supplementation improves delayed recall: A systematic review and meta-Analysis
Impact of disabilities in activities of daily living on opioid use for chronic pain in older adults: an exploratory secondary analysis from ELSI-Brazil
A website to support people with dementia and their family caregivers in advance care planning: Results of a mixed-method evaluation study
Age of First Exposure Does Not Relate to Post-Career Health in Former Professional American-Style Football Players
Can weekend catch-up sleep decrease the risk of cognitive dysfunction in older adults?

Potentially Modifiable Dementia Risk Factors in Canada: An Analysis of Canadian Longitudinal Study on Aging with a Multi-Country Comparison
Association of Plasma Markers of Alzheimer‚Äôs Disease, Neurodegeneration, and Neuroinflammation with the Choroid Plexus Integrity in Aging
The association between blood biological age at rehabilitation admission and physical activity during rehabilitation in geriatric inpatients: RESORT
Lifestyle approaches to hypertension for prevention of stroke and vascular cognitive impairment: A realist review protocol
Exergaming for dementia and mild cognitive impairment
Associations of Rest-Activity Rhythm Disturbances With Stroke Risk and Poststroke Adverse Outcomes
Association of Resilience-Related Life Experiences on Variability on Age of Onset in Dominantly Inherited Alzheimer Disease
Intracranial Atherosclerotic Disease and Incident Dementia: The ARIC Study (Atherosclerosis Risk in Communities)
Increased incidence of neurodegenerative diseases in Finnish individuals with type 1 diabetes
Relation of Cognitive Reserve Indicator to Heart Disease and Cardiac Structure and Function: A Large Community-Based Longitudinal Study
Depressive symptoms score predicts incident type 2 diabetes in community dwelling old Icelandic people
Age-Related Differences in the Effectiveness of Rehabilitation to Improve Activities of Daily Living in Patients with Stroke: A Cross-Sectional Study
The Association between Physical Frailty and Cognitive Performance in Older Adults Aged 60 to 96 Years: Data from the ‚ÄúGood Aging in Sk√•ne‚Äù (G√ÖS) Swedish Population Study
‚ÄúIt‚Äôs just getting the word out there‚Äù: Self-disclosure by people with young-onset dementia
Mediterranean Diet Modulation of Neuroinflammation-Related Genes in Elderly Adults at High Cardiovascular Risk
MIND Diet Impact on Multiple Sclerosis Patients: Biochemical Changes after Nutritional Intervention
Associations between Aircraft Noise, Sleep, and Sleep‚ÄìWake Cycle: Actimetric Data from the UK Biobank Cohort near Four Major Airports
What Explains the Link Between Hearing and Vision Impairment and Cognitive Function? Analysis of Mediating Effects in the USA, England and Ireland
The OASIS walking study‚ÄîOlder adults with cognitive impairment performing sit to stands and walking in transitional care programs: Protocol for a feasibility study
Implementation of COGNIVITRA, an Information- and Communications-Technology-Based Solution for Dual-Task Training, in Patients at Risk of Cognitive Impairment
Age of Diabetes Diagnosis and Lifetime Risk of Dementia: The Atherosclerosis Risk in Communities (ARIC) Study
Effect of long-term physical exercise and multidomain interventions on cognitive function and the risk of mild cognitive impairment and dementia in older adults: A systematic review with meta-analysis
Microvascular Disease, Cardiovascular Health, and Risk of Coronary Heart Disease in Type 2 Diabetes: A UK Biobank Study
Balance and strength measures are associated with incident dementia in older men
Association Between Cognitive Function and Risk of Chronic Kidney Disease: A Longitudinal Cohort and Mendelian Randomization Study
Machine Learning‚ÄìBased Perivascular Space Volumetry in Alzheimer Disease
18F-Flortaucipir (AV1451) imaging identifies grey matter atrophy in retired athletes
Association of sleep duration with excess risk of dementia among shift workers in the UK biobank: a population-based cohort study
Association of the serum levels of saturated fatty acids and mild cognitive impairment: a cross-sectional study
Interactions between age, sex and visceral adipose tissue on brain ageing
Risk factors associated with the Motoric Cognitive Risk syndrome: A meta-analysis of data from a cross-national study
Association and prediction of Life's Essential 8 score, genetic susceptibility with MCI, dementia, and MRI indices: A prospective cohort study
Nutritional Considerations During Major Weight Loss Therapy: Focus on Optimal Protein and a Low-Carbohydrate Dietary Pattern
Uncovering Predictors of Low Hippocampal Volume: Evidence from a Large-Scale Machine-Learning-Based Study in the UK Biobank
Association of Regular Opioid Use With Incident Dementia and Neuroimaging Markers of Brain Health in Chronic Pain Patients: Analysis of UK Biobank
Associations of accelerometry-measured and self-reported physical activity and sedentary behavior with skeletal muscle energetics: The Study of Muscle, Mobility and Aging (SOMMA)
A randomized controlled trial of repetitive transcranial magnetic stimulation plus donepezil vs donepezil alone for mild to moderate cognitive impairment due to small vessel cerebrovascular disease
Micronutrients, Frailty, and Cognitive Impairment: Design and Preliminary Results from the CogLife 2.0 Study
Non-Pharmacological Treatment of Autonomic Dysfunction in Parkinson's Disease and Other Synucleinopathies
Prevalence of Dementia and Cognitive Impairment in East Africa Region: A Scoping Review of Population-Based Studies and Call for Further Research
Association between residential greenness and incident delirium: A prospective cohort study in the UK Biobank
Propelling Nurse-Led Structured Intervention to Enhance Self-Care among Patients with Chronic Heart Failure (PROACT-HF): A Cluster Randomized Controlled Trial Study Protocol
DiADEM‚ÄîDance against Dementia‚ÄîEffect of a Six-Month Dance Intervention on Physical Fitness in Older Adults with Mild Cognitive Impairment: A Randomized, Controlled Trial
Differentiating Left Ventricular Remodeling in Aortic Stenosis From Systemic Hypertension
Physical function is associated with cognitive status, brain amyloid-beta deposition, and blood biomarkers in Chinese Han population
Comparing the effects of biguanides and dipeptidyl peptidase-4 inhibitors on cardio-cerebrovascular outcomes, nephropathy, retinopathy, neuropathy, and treatment costs in diabetic patients
Association of remnant cholesterol with risk of dementia: a nationwide population-based cohort study in South Korea
Association between geniquin therapy and the risk of developing periodontal disease in patients with primary Sj√∂gren's syndrome: A population-based cohort study from Taiwan

Group lasso priors for Bayesian accelerated failure time models with left-truncated and interval-censored data
Associations between socioeconomic inequalities and progression to psychological and cognitive multimorbidities after onset of a physical condition: a multicohort study
Association of changes in predicted body composition with subsequent risk of dementia
Interplay between orthopedic interventions and dementia: Dementia risk after total knee replacement
Telework and 24-hour movement behaviours among adults living in Canada during the COVID-19 pandemic
Pneumococcal vaccination, but not influenza vaccination, is negatively associated with incident dementia among Japanese older adults: The JAGES 2013,Äì2022 prospective cohort study
Healthy aging meta-analyses and scoping review of risk factors across Latin America reveal large heterogeneity and weak predictive models
Gardening and cognitive ageing: Longitudinal findings from the lothian birth cohort of 1921
Using the Outcome and Assessment Information Set to Measure Patient Health Status in Research: A Systematic Review
Psychosocial distress among spouses of persons with dementia before and after their partner's death
Associations of empirically derived dietary patterns and cognitive performance in older men: Results of the Osteoporotic Fractures in Men (MrOS) study
Long-term changes in frailty and incident type 2 diabetes: A prospective cohort study based on the UK Biobank
Cross-sectional association of continuous glucose monitoring-derived metrics with cerebral small vessel disease in older adults with type 2 diabetes
Determinants of serious health outcome-free status in middle-aged and older people with dysglycaemia: Exploratory analysis of the ORIGIN trial
Mechanical Ventilation in Older Adults With Dementia: Opportunities to Promote Goal-Concordant Care
Ductal carcinoma in situ and cause-specific mortality among younger and older postmenopausal women: the Women,Äôs Health Initiative
Predicting Brain Amyloid Status Using the National Institute of Health Toolbox (NIHTB) for Assessment of Neurological and Behavioral Function
Association of Depression With the Progression of Multimorbidity in Older Adults: A Population-Based Cohort Study
Association of birthweight and risk of incident dementia: a prospective cohort study
Factors Associated With Psychological Wellbeing in Home Care Older Adults With Alzheimer,Äôs Disease: A Longitudinal Analysis
Effectiveness of Combined Aerobic and Resistance Exercise on Cognition, Metabolic Health, Physical Function, and Health-related Quality of Life in Middle-aged and Older Adults With Type 2 Diabetes Mellitus: A Systematic Review and Meta-analysis
Brain Care Score and Neuroimaging Markers of Brain Health in Asymptomatic Middle-Age Persons
Association between Body Composition Patterns, Cardiovascular Disease, and Risk of Neurodegenerative Disease in the UK Biobank
Safety of TNF-Œ± inhibitors: A real-world study based on the US FDA Adverse Event Reporting System Database
Diabetes and Fasting in Ramadan
Marital History and Cognition in a Chinese Longevity Cohort
Digital Biomarkers for the Assessment of Non-Cognitive Symptoms in Patients with Dementia with Lewy Bodies: A Systematic Review
Nonarteritic Anterior Ischemic Optic Neuropathy and the Risk of Dementia: A Nationwide Cohort Study
Predictors of Mortality after Hip Fracture: A Systematic Overview of Secondary Studies
Dementias. What are we doing today and what could we do? Analysis of a cohort in the real world of patients with cognitive and behavioral disorders
Interplay of physical and recognition performance using hierarchical continuous-time dynamic modeling and a dual-task training regime in Alzheimer's patients
Associations between accelerometer-derived sedentary behavior and physical activity with white matter hyperintensities in middle-aged to older adults
Reprint of: The impact of alpha-1-adrenergic receptor antagonists on the progression of Parkinson disease
Multiparameter cortical surface morphology in former amateur contact sport athletes
The Effectiveness of an Exercise Program on Muscle Strength and Range of Motion on Upper Limbs, Functional Ability and Depression at Early Stage of Dementia
Concussion-Related Biomarker Variations in Retired Rugby Players and Implications for Neurodegenerative Disease Risk: The UK Rugby Health Study
Atrial fibrillation accelerates functional decline in older adults: a 15-year follow-up population-based study
Association of lifestyle modification with the development of cardiovascular disease in gastric cancer patients who underwent gastrectomy: A nationwide population-based study
Stimulating leisure-time activities and the risk of dementia: A multi-cohort study
Mitochondrial Health, Physical Functioning, and Daily Affect: Bioenergetic Mechanisms of Dementia Caregiver Well-Being
Social Induction via a Social Behavioral Intervention on Changes in Metabolic Risk Factors: A Randomized Controlled Trial in Rural Appalachia, United States
Motoric Cognitive Risk Syndrome as a Predictor of Adverse Health Outcomes: A Systematic Review and Meta-Analysis
Changes in prevalence and incidence of dementia and risk factors for dementia: an analysis from cohort studies
Concurrent nutrient deficiencies are associated with dementia incidence
Effect of vigorous-intensity physical activity on incident cognitive impairment in high-risk hypertension
Cognitive reserve, cortisol, and Alzheimer's disease biomarkers: A memory clinic study
The associations between nutritional status and physical frailty and Alzheimer's disease plasma biomarkers in older cognitively unimpaired adults with positive of amyloid-Œ≤ PET
Irisin Levels in Cerebrospinal Fluid Correlate with Biomarkers and Clinical Dementia Scores in Alzheimer Disease
Single-session catheter-directed lysis using adjunctive clot fragmentation with power pulse spray only is a fast, safe, and effective option for acute pulmonary embolism
Concomitant autoimmunity and risk of multiple sclerosis disability worsening
Vision-based motion capture for the gait analysis of neurodegenerative diseases: A review

Association of accelerated aging with the incidence risk of Cardiometabolic diseases in people with severe mental illnesses: A prospective analysis in UK Biobank
Association between changes in cardiovascular health and the risk of multimorbidity: community-based cohort studies in the UK and Finland
Symptom-based staging for logopenic variant primary progressive aphasia
Self-administered generational surveys combine with genetic analysis to reveal foundations of depression in Japanese adults
Association of diabetes mellitus with dementia- and non-dementia-related mortality amongst women: a secondary competing risks analysis of the California Teachers Study
Impact of demographic factors, comorbidities, and co-medication on progression and survival in a large Chinese ALS cohort
,ÄúGood Care Is Slow Enough to Be Able to Pay Attention,Äù: Primary Care Time Scarcity and Patient Safety
Predicting life span of type 2 diabetes patients through alkaline phosphatase and vitamin D: Results from NHANES 1999,Äì2018
Characterization of elderly adults for healthy aging
Lung Function and Brain MRI Outcomes in the Atherosclerosis Risk in Communities Neurocognitive Study
Dementia Ideal Care: Ecosystem Map of Best Practices and Care Pathways Enhanced by Technology and Community
Protocol for a double-blind placebo-controlled randomised controlled trial assessing the impact of oral semaglutide in amyloid positivity (ISAP) in community dwelling UK adults
Potential modifying effect of grip strength on the association between glycated hemoglobin (HbA1c) and all-cause mortality in older adults with type 2 diabetes: Evidence from UK Biobank
Association of Cardiovascular Health With Brain Age Estimated Using Machine Learning Methods in Middle-Aged and Older Adults
Analysis of epidemic status and influencing factors of mild cognitive impairment in Chinese people ,â• 65 years old
The usability and reliability of a smartphone application for monitoring future dementia risk in ageing UK adults
Lifetime History of Low Birth Weight Delivery and Cognitive Function in Middle-Aged Parous Women
Intrinsic Capacity, Polygenic Risk Score, APOE Genotype, and Risk of Dementia: A Prospective Cohort Study Based on the UK Biobank
Predictors of 5,Äëyear Survival of Elderly with Hypertension. A Prospective Cohort Study
Role of Ketogenic Diets and Intermittent Fasting in Neurologic Diseases, Cancers, and Obesity: A Systematic Review of Human Studies
Atrial Fibrillation and Other Cardiovascular Factors and the Risk of Dementia: An Italian Case,ÄìControl Study
Discriminant Accuracy of Standing Balance Tests for the Level of Gait Dependency in Hospitalized Patients with Alzheimer,Äôs Disease
Associations of Testosterone and Related Hormones With All-Cause and Cardiovascular Mortality and Incident Cardiovascular Disease in Men Individual Participant Data Meta-analyses
Guideline for the cognitive assessment and follow-up in the Guangdong-Hong Kong-Macao Greater Bay Area (2024 edition)
Technology-based group exercise interventions for people living with dementia or mild cognitive impairment: A scoping review
Associations between fasting glucose rate-of-change and the missense variant, rs373863828, in an adult Samoan cohort
Psychosocial Support in Pulmonary Rehabilitation
Percutaneous needle tenotomies: indications, procedures, efficacy and safety. A systematic review
Association between Hypertensive Disorders of Pregnancy and Interval Neurocognitive Decline: An Analysis of the Hispanic Community Health Study/Study of Latinos
Associations between information and communication technology use and frailty in community-dwelling old-old adults: results from the ILSA-J
Long-term exposure to air pollution and road traffic noise and incidence of dementia in the Danish Nurse Cohort
Associations of hospital-treated infections with subsequent dementia: nationwide 30-year analysis
Associations between housing and psychological damage by earthquake and modifiable risk factors for dementia in general older adults: Tohoku Medical Megabank community-based cohort study
The 2022 symposium on dementia and brain aging in low- and middle-income countries: Highlights on research, diagnosis, care, and impact
Effects of home-based and telerehabilitation exercise on mental and physical health, and disease cost in people with Alzheimer's disease: A meta-analysis
Predicting cognitive function and dementia risk in patients with hypertension
Does Your Patient Need to Lose Weight? Weight Change Before and After Total Hip Arthroplasty Does Not Affect Health Care Utilization and Achievement of the Minimal Clinically Important Difference
Prevalence and impact of depression and anxiety among older myocardial infarction survivors: A nationwide cohort study
The Association between cognitive impairment and anthropometric indices among the elderly: birjand longitudinal aging study
Microvascular burden and long-term risk of stroke and dementia in type 2 diabetes mellitus
Choroid plexus calcifications are not associated with putative markers of glymphatic dysfunction: A population study in middle-aged and older adults
Physical function trajectory after wrist or lower arm fracture in postmenopausal women: results from the Women,Äôs Health Initiative Study
Performance of digital technologies in assessing fall risks among older adults with cognitive impairment: a systematic review
Diet, Pace of Biological Aging, and Risk of Dementia in the Framingham Heart Study
Prevalence and risk factors for impaired activities of daily living in patients with neo-vascular age-related macular degeneration who present for anti-VEGF treatment
Understanding Health Beliefs and Health Behaviors in Older Adults at Risk for Alzheimer's Disease
Socioeconomic Status Transition Throughout Life and Risk of Dementia
Associations Between Ultra-Processed Food Consumption and Adverse Brain Health Outcomes
Loneliness, Dementia Status, and Their Association with All-Cause Mortality Among Older US Adults

Association between glycaemic control and malnutrition in older adults with type 2 diabetes mellitus: A cross-sectional study
A Review of the Current Status of Disease-Modifying Therapies and Prevention of Alzheimer‚Äôs Disease
Metabolites and Cognitive Decline in a Puerto Rican Cohort
Cardiovascular Health, Race, and Decline in Cognitive Function in Midlife Women: The Study of Women‚Äôs Health Across the Nation
Consumption of Olive Oil and Diet Quality and Risk of Dementia-Related Death
Subacute sclerosing panencephalitis presenting as severe depression in an adult
Effect of Controlling the Cardiovascular Risk Factors on the Cognitive Decline Prevention in the Elderly: A Systematic Review
Exploring the potential relationship between short sleep risks and cognitive function from the perspective of inflammatory biomarkers and cellular pathways: Insights from population-based and mice studies
Editor's view
Development and validation of the Michigan Chronic Disease Simulation Model (MICROSIM)
Association of dietary and nutritional factors with cognitive decline, dementia, and depressive symptomatology in older individuals according to a neurogenesis-centred biological susceptibility to brain ageing
Dietary characteristics of community-dwelling older adults with poor appetite: a cross-sectional analysis
Evaluating a novel 24-hour rest/activity rhythm marker of preclinical Œ≤-amyloid deposition
Leg strength and incidence of heart failure in patients with acute coronary syndrome
Preserving cognitive vitality: Value of cognitive rehabilitation in addressing cognitive deficits in the elderly
Intraindividual difference in estimated GFR by creatinine and cystatin C, cognitive trajectories and motoric cognitive risk syndrome
Neuropathologic changes at age 90+ related to sleep duration 19 to 40 years earlier: The 90+ Study
China Initiative for Multi-Domain Intervention (CHINA-IN-MUDI) to Prevent Cognitive Decline: Study Design and Progress
Mendelian Randomization Analysis Reveals Causal Factors behind Alzheimer‚Äôs Disease Risk: Evidence, Opportunities, and Challenges
The role of cardiovascular health and vascular events in the relationship between excessive daytime sleepiness and dementia risk
Go4cognition: Evaluation of a Newly Developed Multicomponent Intervention in Mild Cognitive Impairment
Trajectories of Occupational Cognitive Demands and Risk of Mild Cognitive Impairment and Dementia in Later Life The HUNT4 70+ Study
Factors Associated with Lower Social Activity in German Older Adults at Increased Risk of Dementia: A Cross-Sectional Analysis
Associations Between Age at Menopause, Vascular Risk, and 3-Year Cognitive Change in the Canadian Longitudinal Study on Aging
Caloric Restriction Improves Spatial Learning Deficits in Tau Mice
Communicating Alzheimer's biomarker results to cognitively unimpaired research participants: Satisfaction, utility, and impact on research attitudes
The Association Between Physical Activity and Cognition in a Racially/Ethnically Diverse Cohort of Older Adults: Results From the Kaiser Healthy Aging and Diverse Life Experiences Study
Cognitive impairment and neurocognitive profiles among people living with HIV and HIV-negative individuals older over 50 years: a comparison of IHDS, MMSE and MoCA
How Lifetime Evolution of Parkinson‚Äôs Disease Could Shape Clinical Trial Design: A Shared Patient‚ÄìClinician Viewpoint
Sex differences in cognitive decline among middle-aged and older adults: a cohort study in Europe
Deep phenotyping of dementia in a multi-ethnic cardiovascular cohort: The Multi-Ethnic Study of Atherosclerosis (MESA)
Plasma metabolomic markers underlying skeletal muscle mitochondrial function relationships with cognition and motor function
The association between cannabis use and neuroimaging measures in older adults: findings from the UK biobank
Benefits of Music Therapy in the Cognitive Impairments of Alzheimer‚Äôs-Type Dementia: A Systematic Review
Association between hemoglobin trajectories and the incidence of dementia in a cohort of females aged 55-79 years
Physical Activity-Induced Modification of the Association of Long-Term Air Pollution Exposure with the Risk of Depression in Older Adults
Cognitive Function in Atrial Fibrillation: A Narrative Review of Evidence and Mechanisms
The clinical utility of three frailty measures in identifying HIV-associated neurocognitive disorders
Effects of cognitive reserve on cognitive frailty among older adults: A population-based prospective cohort study
The associations of serum vitamin D status and vitamin D supplements use with all-cause dementia, Alzheimer's disease, and vascular dementia: a UK Biobank based prospective cohort study
CKD could be a new risk factor of dementia
Altered cerebellar and caudate gray-matter volumes and structural covariance networks preceding dual cognitive and mobility impairments in older people
SPTLC2 variants are associated with early-onset ALS and FTD due to aberrant sphingolipid synthesis
Social frailty and the incidence of motoric cognitive risk syndrome in older adults
Implications of metabolism on multi-systems healthy aging across the lifespan
Family dementia caregivers with suicidal ideation improve with mentalizing imagery therapy: Results from a pilot study
Associations between sleep-behavioral traits and healthspan: A one-sample Mendelian randomization study based on 388,909 participants of the UK-Biobank
Occupational and domestic exposure associations with cerebral small vessel disease and vascular dementia: A systematic review and meta-analysis
Comment on: Association of sulfonylureas with the risk of dementia: A population-based cohort study
Socioeconomic status, lifestyle and risk of incident dementia: a prospective cohort study of 276730 participants
Explaining the Variability of Alzheimer Disease Fluid Biomarker Concentrations in Memory Clinic Patients Without Dementia

Association of Coffee Consumption and Prediagnostic Caffeine Metabolites With Incident Parkinson Disease in a Population-Based Cohort
Seasonal Variations in Vitamin D Levels and the Incident Dementia among Older Adults Aged ,â•60 Years in the UK Biobank
Characterizing Multimorbidity Prevalence and Adverse Outcomes in Ethnically and Culturally Diverse Sub-Populations in India: Gaps, Opportunities, and Future Directions
Association Between Baseline Gait Parameters and Future Fall Risk in Patients With De Novo Parkinson,Äôs Disease: Forward Versus Backward Gait
Long-term exposure to transportation noise in relation to global cognitive decline and cognitive impairment: Results from a Swedish longitudinal cohort
Presymptomatic Profiles of Cognitive Impairment with Prior Mobility Impairment
Lifetime Physical Activity and Late-Life Mild Cognitive Impairment in Community-Dwelling Older Adults
The impact of alpha-1-adrenergic receptor antagonists on the progression of Parkinson disease
Life-Course Socioeconomic Position and Mild Cognitive Impairment in Midlife: Evidence from the 1958 British Birth Cohort
Umbrella review and Delphi study on modifiable factors for dementia risk reduction
Social Determinants of Intrinsic Capacity: A National Cohort Study
The burden of head trauma in rural and remote North Queensland, Australia
Exploring Potential Predictors of Treadmill Training Effects in People With Parkinson Disease
Association between regular physical activity and pneumonia-related hospitalization according to pneumococcal vaccination status: The VENUS study
Brain perfusion change in patients with mild cognitive impairment after 12 months of aerobic exercise training
Multimorbidity, Social Engagement, and Age-Related Cognitive Decline in Older Adults from the Rancho Bernardo Study of Healthy Aging
Mild Behavioral Impairment and Cerebrovascular Profiles Are Associated with Early Cognitive Impairment in a Community-Based Southeast Asian Cohort
Long-Term Brain Structure and Cognition Following Bariatric Surgery
Sedentary Behavior and Dementia-Reply
Metabolic-dysfunction associated steatotic liver disease-related diseases, cognition and dementia: A two-sample mendelian randomization study
Radiotherapy-induced vascular cognitive impairment 20 years after childhood brain tumor
Variation in care and outcomes for people after hip fracture with and without cognitive impairment; results from the Australian and New Zealand Hip Fracture Registry
Investigating the synergistic effects of hormone replacement therapy, apolipoprotein E and age on brain health in the UK Biobank
Maintaining independence in individuals with dementia at home after a fall: a protocol for the UK pilot cluster randomised controlled trial MAINTAIN
Glial reactivity and T cell infiltration in frontotemporal lobar degeneration with tau pathology
The plasma derived exosomal miRNA-483-5p/502-5p serve as potential MCI biomarkers in aging
Clinical effectiveness of pharmacological and non-pharmacological treatments for the management of anxiety in community dwelling people living with dementia: A systematic review and meta-analysis
Association of plasma high-density lipoprotein cholesterol level with risk of incident dementia: a cohort study of healthy older adults
Risk of dementia in survivors of active tuberculosis in Korea: A nationwide cohort study
Associations Between Cardiac Function and Brain Health in Diverse Middle-Aged Adults: The Dallas Heart Study-2
Optimal cardiovascular health is associated with slower cognitive decline
Physical activity and brain amyloid beta: A longitudinal analysis of cognitively unimpaired older adults
Associations of the Mediterranean-DASH Intervention for Neurodegenerative Delay diet with brain structural markers and their changes
Functional Decline in Long-Term Care Homes in the First Wave of the COVID-19 Pandemic: A Population-based Longitudinal Study in Five Canadian Provinces
Diabetes self-management education on the sustainability of metabolic control in type 2 diabetes patients: Diabetes share care program in Taiwan
Association Between Speech Reception Threshold in Noise and Multimorbidity: The UK Biobank Study
Smoking and home oxygen therapy: a review and consensus statement from a multidisciplinary Swedish taskforce
Cardiovascular risk burden, dementia risk and brain structural imaging markers: a study from UK Biobank
Association of the Sleep Regularity Index with Incident Dementia and Brain Volume
Impact of comorbidity on cognitive function of possible vascular origin
Shifting From Active to Passive Monitoring of Alzheimer Disease: The State of the Research
Interventional treatment of giant tracheal lymphoma under rigid bronchoscopy: A case report and literature review
Inflammatory Risk Status Shapes the Association Between Uric Acid and Cognitive Function in Non-Hyperuricemia Middle Aged and Elderly
A multidisciplinary collaborative care model involving family members in the treatment of type 2 diabetes mellitus and associated diabetes distress in young and middle-aged patients
The Effect of the Mediterranean Diet-Integrated Gamified Home-Based Cognitive-Nutritional (GAHOCON) Training Programme for Older People With Cognitive Frailty: Pilot Randomized Controlled Trial
Environmental enrichment is associated with favorable memory-related functional brain activity patterns in older adults
Alternative Therapies for Non-Motor Symptoms in Parkinson,Äôs Disease: A Mini Review
Associations of NETs with inflammatory risk and clinical predictive value in large artery atherosclerosis stroke: a prospective cohort study
A fusion analytic framework for investigating functional brain connectivity differences using resting-state fMRI
Postural imbalance without visual input is associated with specific neuropsychological deficits in older adults ,Äì results from the LIFE-adult study
ISLAND Campus: a fee-free formal university educational intervention in mid- to later-life to reduce modifiable risk factors for dementia and improve cognition

Pathologic Function of Cyclin-dependent Kinase 5 and Its Relationship With Exercise
Association between calculated remnant cholesterol levels and incident risks of Alzheimer‚Äôs disease among elderly patients with type 2 diabetes: a real-world study
Social participation and cognitive activities as explanation factor for the association between income and self-rated health for older adults
The association between medical fitness facility attendance and incident mental disorders
Physical Activity and Neurodegenerative Diseases: Potential Role in Prevention and Therapy
Embracing Reablement as an Essential Support Approach for Dementia Care in the 21st Century: A Position Paper
Trajectories of cognitive function and frailty in older adults in China: a longitudinal study
Unveiling the hidden risks: albumin-corrected anion gap as a superior marker for cardiovascular mortality in type 2 diabetes: insights from a nationally prospective cohort study
Directions of Longitudinal Relationships between Housing-related Control Beliefs and Activities of Daily Living among People with Parkinson‚Äôs disease
Assessment of Wearable Device Adherence for Monitoring Physical Activity in Older Adults: Pilot Cohort Study
Towards a Non-pharmacological Intervention on Apathy in Korsakoff‚Äôs Syndrome: A Systematic Narrative Review Across Different Clinical Conditions
Dual-Plate Fixation in Treatment of Clavicle Fractures in Dementia Patients: A Case Series
The role of inflammation in neurological disorders: a brief overview of multiple sclerosis, Alzheimer‚Äôs, and Parkinson‚Äôs disease‚Äô
Association of gait speed and handgrip strength with falls in older adults: the role of cognition
Digital phenotyping for changes in activity at the end of life in people with dementia
Usability of remote digital intervention: An analysis from the perspective of caregivers of elderly individuals with dementia
Nested case control study of prevalence and aetiology of dementia in a rural Ugandan population, and a situational analysis of services available for affected families: a protocol. Part of the DEPEND Uganda study (Dementia EPidemiology, unmet Need and co-Developing Solutions)
The Trail Walking Test to predict probable mild cognitive impairment in older adults
From Editor-in-Chief: Our Hirsch index, current issue, news from ESC 2024, trials and new guidelines, protection and long COVID
Digital Phenotyping of Mental and Physical Conditions: Remote Monitoring of Patients Through RADAR-Base Platform
Validation of the Updated ‚ÄúLIfestyle for BRAin health‚Äù (LIBRA) Index in the English Longitudinal Study of Ageing and Maastricht Aging Study
Antidepressant medications in women aged 40 and older and the risk of fragility fractures: a systematic literature review and meta-analysis
Can integration of Alzheimer's plasma biomarkers with MRI, cardiovascular, genetics, and lifestyle measures improve cognition prediction?
Appraising motivation as a predictor of independence in individual activities of daily living in patients with subacute stroke: a retrospective study
The Wellbeing Index for Persons with Dementia‚ÄîAn Observational Study Based on the Group Observational Measurement of Engagement (GOME)
An exploratory analysis of the associations between accelerometer-derived measures and future health-related changes in older adults: Preliminary results on prospective cohort study
A smoking cessation intervention for rural veterans tailored to individual risk factors: A multicenter randomized clinical trial
The Importance of Long-term Partner Observation in Cognitive Evaluation: A Very Early Creutzfeldt-Jakob Disease in a Patient with Mild Cognitive Impairment
Prevention of Age-Related-Increases in the Risks of Incident Functional Disability and Dementia by Home-Delivered Functional Dairy Product Consumption in Japanese Older Adults
Effect of adult day care centre attendance on hypertension management
Advancing stroke diagnosis and management through nuclear medicine: a systematic review of clinical trials
Behavioral marker-based predictive modeling of functional status for older adults with subjective cognitive decline and mild cognitive impairment: Study protocol
Independent Walking Disability After Fragility Hip Fractures: A Prognostic Factors Analysis
Association between a combination of cognitively stimulating leisure activities and long-chain polyunsaturated fatty acid intake on cognitive decline among community-dwelling older Japanese individuals
Effects of standardized environmental enrichment on cognitive function and serum BDNF level in patients with post-stroke dementia
The predictive validity of a Brain Care Score for late-life depression and a composite outcome of dementia, stroke, and late-life depression: data from the UK Biobank cohort
Case management in a web-based cognitive and motor treatment for patients with mild cognitive impairment: A study protocol of a multicentric, randomized, two parallel arms controlled clinical trial
Association of modified dementia risk score with cerebrospinal fluid biomarkers and cognition in adults without dementia
Functional neurological symptom disorder in a patient with hemibody weakness: A case report
Mild Cognitive Impairment: Clinical Presentation and Short-Term Comparative Outcome of Patients With Potentially Modifiable and Nonmodifiable Risk Factors
Tele-rehabilitation for Type II diabetics with heart failure with preserved ejection fraction
Enhancing predictive validity of motoric cognitive risk syndrome for incident dementia and all-cause mortality with handgrip strength: insights from a prospective cohort study
Risk factors and biomarkers of Alzheimer's disease
Decrease in the usual walking speed and body fat percentage associated with a deterioration in long-term care insurance certification levels
The mind and motion: exploring the interplay between physical activity and Mild Behavioral Impairment in dementia-free older adults
Transcranial photobiomodulation therapy improves cognitive test scores in dogs with presumptive canine cognitive dysfunction: A case series of five dogs
EVALUATION OF THE EFFECTIVENESS OF A COMPREHENSIVE PROGRAM OF REHABILITATION OFFERED ACCORDING TO DIFFERENT THERAPEUTIC PROTOCOLS IN REDUCING CHRONIC FRONTAL SYNDROME IN PATIENTS 10-YEARS FOLLOWING NEUROSURGERY FOR POST-TRAUMATIC INTRACEREBRAL HEMATOMA

Time-varying lifestyle and mental-ill health risk factors for the longitudinal development of daily activity limitations among middle-aged and older adults in Thailand
Implications of helplessness in depression: diagnosing mild cognitive impairment and analyzing its effects on cognitive decline in older adults
Peripheral vascular dysfunction and the aging brain
Effectiveness of Early Multimodal Non-pharmacological Interventions in Cognitive Preservation in the Elderly
Prevalence and risk factors for falls among older Chinese adults in the community: findings from the CLHLS study
Incorporating Cognitive Training with Elderly People‚Äôs Everyday Use of Smartphones
Measuring Physical Functioning Using Wearable Sensors in Parkinson Disease and Chronic Obstructive Pulmonary Disease (the Accuracy of Digital Assessment of Performance Trial Study): Protocol for a Prospective Observational Study
Examining biopsychosocial predictors of risk for cognitive impairment among a racially diverse sample of men who have sex with men living with HIV
Identification of gene networks jointly associated with depressive symptoms and cardiovascular health metrics using whole blood transcriptome in the Young Finns Study
Patients Aged 80 or More With Distal Radius Fractures Have a Lower One-Year Mortality Rate Than Age- and Gender-Matched Controls: A Register-Based Study
The effect of weighted blankets on sleep and related disorders: a brief review
Cognitive reserve over the life course and risk of dementia: a systematic review and meta-analysis
ZORA robot to assist a caregiver in prospective memory tasks: A preliminary study: Prospective memory; humanoid robot; alzheimer‚Äôs disease
Branched-chain amino acids and the risks of dementia, Alzheimer‚Äôs disease, and Parkinson‚Äôs disease
Neutrophil-Lymphocyte Ratio as a Predictor of the Risk of Death in Severe Cases of COVID-19
Physical activity may a probably protective factor for postoperative delirium: the PNDABLE study
Irreversible atrophy in memory brain regions over 7 years is predicted by glycemic control in type 2 diabetes without mild cognitive impairment
Body Composition, Sarcopenic Obesity, and Cognitive Function in Older Adults: Findings From the National Health and Nutrition Examination Survey (NHANES) 1999‚Äì2002 and 2011‚Äì2014
Safety and efficacy of acupuncture for mild cognitive impairment: a study protocol for clinical study
The Association of Retinal Disease with Vision Impairment and Functional Status in Medicare Patients
Mitigating cellular aging and enhancing cognitive functionality: visual arts-mediated Cognitive Activation Therapy in neurocognitive disorders
Risk factors and cognitive correlates of white matter hyperintensities in ethnically diverse populations without dementia: The COSMIC consortium
A cross-sectional observational study for ethno-geographical disparities in sleep quality, brain morphometry and cognition (a SOLACE study) in Indians residing in India, and South Asians and Europeans residing in the UK ‚Äì a study protocol
Impact of mindfulness-based and health self-management interventions on mindfulness, self-compassion, and physical activity in older adults with subjective cognitive decline: A secondary analysis of the SCD-Well randomized controlled trial
Validation of a Computerized Cognitive Training Tool to Assess Cognitive Impairment and Enable Differentiation Between Mild Cognitive Impairment and Dementia
Multidimensional Sleep Health and Long-Term Cognitive Decline in Community-Dwelling Older Men
Using mHealth for Primary Prevention of Dementia: A Proof-of-Concept Study on Usage Patterns, Appreciation, and Beliefs and Attitudes Regarding Prevention
The Impact of Long Working Hours on Cognitive Function: A Follow-Up Study with Gender Stratification
Association Between Dietary Fish and PUFA Intake in Midlife and Dementia in Later Life: The JPHC Saku Mental Health Study
Observed Improvement in Cognition During a Personalized Lifestyle Intervention in People with Cognitive Decline
A Remotely Coached Multimodal Lifestyle Intervention for Alzheimer's Disease Ameliorates Functional and Cognitive Outcomes
Race Differences in the Association Between Sleep Medication Use and Risk of Dementia
Physical Activity and Trajectory of Cognitive Change in Older Persons: Mayo Clinic Study of Aging
Recruitment and baseline data of the Aging and Cognitive Health Evaluation in Elders (ACHIEVE) study: A randomized trial of a hearing loss intervention for reducing cognitive decline
Inflammation, Autoimmunity and Neurodegenerative Diseases, Therapeutics and Beyond
Gait and cognitive disorders revealing massive neurocysticercosis: a case report
Longitudinal clustering of health behaviours and their association with multimorbidity in older adults in England: A latent class analysis
Changes in instrumental activities daily living limitations and their associated factors according to gender in community-residing older adults: A longitudinal cohort study
Trajectory of multimorbidity before dementia: A 24-year follow-up study
Accelerometer-measured physical activity and posture among older adults in assisted-living residences
The semicircular canal function is preserved with little impact on falls in patients with mild Parkinson's disease
The impact of falls on activities of daily living in older adults: A retrospective cohort analysis
Visit-to-Visit Glucose Variability, Cognition, and Global Cognitive Decline: The Multi-Ethnic Study of Atherosclerosis
Association of Thyroid Hormone Medication Adherence With Risk of Dementia
Intradialysis exercise in haemodialysis patients: effective but complex and costly
Food Insecurity, Race and Ethnicity, and Cognitive Function Among United States Older Adults
Prevalence and correlates of frailty in older hypertensive outpatients according to different tools: the HYPER-FRAIL pilot study
Reduced tactile sensitivity is associated with mild cognitive impairment

New Psychometric Strategies for the Evaluation of Affective, Cognitive, and Psychosocial Functioning in Unipolar versus Bipolar Depression: Impact of Drug Treatment
Habitual coffee consumption and risk of dementia in older persons: modulation by CYP1A2 polymorphism
Transforming Perspectives in Cardiac Cell Therapy: Hypothesis and Commentary Following Updated Results of a Pilot Study Investigating Very Long-Term Clinical Outcomes in Severe AMI Patients Following Trans-Epicardial Injection of Peripheral Blood CD34+ Cells
Genome-wide association study of traumatic brain injury in U.S. military veterans enrolled in the VA million veteran program
Prosthesis use and the change in activities of daily living following below-knee amputation in patients undergoing hemodialysis
Risk, protective, and biomarkers of dementia in Indigenous peoples: A systematic review
How Modifiable Are Modifiable Dementia Risk Factors? A Framework for Considering the Modifiability of Dementia Risk Factors
Metabolic dysfunction-associated steatotic liver disease in older adults is associated with frailty and social disadvantage
Association of type 2 diabetes mellitus with dementia-related and non,Äidementia-related mortality among postmenopausal women: A secondary competing risks analysis of the women's health initiative
Genetic risk for Alzheimer,Äôs disease and adherence to the Mediterranean diet: results from the HELIAD study
Adequate dietary magnesium intake may protect females but not males older than 55 years from cognitive impairment
Locomotive syndrome affects the acquisition of long-term care insurance system certification
Does Physical Exercise Modify the Pathophysiology of Alzheimer's Disease in Older Persons?
Development and Validation of a Prediction Model for Mild Cognitive Impairment Among Middle-aged Chinese Adults
Use of Virtual Reality to Improve Spatial Orientation in Alzheimer's Disease and Mild Cognitive Impairment: A Systematic Review
Association between dietary fiber intake and all-cause and CVD-caused mortality among heart failure survivors: a cohort study from the NHANES database
Turning the world upside down: reversal-of-vision metamorphopsia in a patient with syncope
Case of Autosomal Dominant Alzheimer Disease With Negative Findings From PiB-PET Examination
A novel, multidomain, primary care nurse-led and mHealth-assisted intervention for dementia risk reduction in middle-aged adults (HAPPI MIND): study protocol for a cluster randomised controlled trial
An online intervention to improve the health and well-being of informal caregivers of individuals with Alzheimer's disease: A pilot study
Neutrophil counts and cardiovascular disease
Relationship between grip strength and minimal clinically important differences in cognitive function in older adults with dementia in a long-term residential facility
History of incarceration and age-related neurodegeneration: Testing models of genetic and environmental risks in a longitudinal panel study of older adults
Moderate and vigorous leisure time physical activity in older adults and Alzheimer's disease-related mortality in the USA: a dose,Àìresponse, population-based study
Chronic obstructive pulmonary disease: hiding in plain sight, a Statement from the COPD Foundation Medical and Scientific Advisory Committee
Longitudinal associations of loneliness with mental ill-health, physical ill-health, lifestyle factors and mortality in ageing adults in Thailand
Family History of AD/Dementia, Polygenic Risk Score for AD, and Parkinson's Disease
Impact on physical function of the +AGIL Barcelona program in community-dwelling older adults with cognitive impairment: an interventional cohort study
Distinct effects of cholesterol profile components on amyloid and vascular burdens
Comparing chair stand test protocols: Fifth stand versus fifth sit
Recent updates in autonomic research: new insights into vagal nerve activity during exercise, cardiac autonomic neuropathy and silent myocardial infarction in diabetes, and timing of orthostatic blood pressure change and future risk of dementia
Relationship of Klotho with cognition and dementia: Results from the NHANES 2011,Äì2014 and Mendelian randomization study
Cognitive impact of multidomain intervention and omega 3 according to blood AŒ≤42/40 ratio: a subgroup analysis from the randomized MAPT trial
The mindfulness bridge: Exploring education and cognitive performance in older adults with low educational attainment
Association of personality facets and cognition in the Lifelines population-based cohort study
A nomogram for predicting mild cognitive impairment in older adults with hypertension
Understanding the role of psychiatrists in the diagnosis and management of mild cognitive impairment and mild Alzheimer,Äôs disease dementia: a cross-sectional survey
Association of PTSD history with confusion or memory loss among World Trade Center Health Registry enrollees
Minimal amount of exercise prevents incident dementia in cognitively normal older adults with osteoarthritis: a retrospective longitudinal follow-up study
Diagnosis and treatment of ,Äúchronic Lyme,Äù: primum non nocere
Association between Dental Diseases and Oral Hygiene Care and the Risk of Dementia: A Retrospective Cohort Study
Neuron-derived extracellular vesicles in blood reveal effects of exercise in Alzheimer,Äôs disease
From genetic correlations of Alzheimer,Äôs disease to classification with artificial neural network models
High-intensity walking in midlife is associated with improved memory in physically capable older adults
Association between serum lipid levels and lower-extremity functions in older adults with and without Alzheimer's dementia in South Korea: A cross-sectional analysis
Physical activity, biomarkers of brain pathologies and dementia risk: Results from the Memento clinical cohort
Identification of high likelihood of dementia in population-based surveys using unsupervised clustering: a longitudinal analysis
Physical and brain frailty in ischaemic stroke or TIA: Shared occurrence and outcomes. A cohort study
Relation of sedentary behaviour to physical function in phase I cardiac rehabilitation

Effects of egg yolk choline intake on cognitive functions and plasma choline levels in healthy middle-aged and older Japanese: a randomized double-blinded placebo-controlled parallel-group study
Alcohol consumption and all-cause and cause-specific mortality among US adults: prospective cohort study
Novel cerebrospinal fluid biomarkers correlating with shunt responsiveness in patients with idiopathic normal pressure hydrocephalus
Deep venous thrombosis and hyponatremia associated with citalopram use for behavioral symptoms in Parkinson,Äôs disease: a case report
The impact of different care dependencies on people,Äôs willingness to provide informal care: a discrete choice experiment in Germany
Statistical analysis plan for HOMESIDE: a randomised controlled trial for home-based family caregiver-delivered music and reading interventions for people living with dementia
Associations between circulating cell-free mitochondrial DNA, inflammatory markers, and cognitive and physical outcomes in community dwelling older adults
The independent and joint association of accelerometer-measured physical activity and sedentary time with dementia: a cohort study in the UK Biobank
Resting-state MRI functional connectivity as a neural correlate of multidomain lifestyle adherence in older adults at risk for Alzheimer,Äôs disease
Sleep disorders of post-COVID-19 conditions
Association between chronic pain and risk of incident dementia: findings from a prospective cohort
Clinico-imaging features of subjects at risk of Lewy body disease in NaT-PROBE baseline analysis
Cognitive impairment according to Montreal Cognitive Assessment independently predicts the ability of chronic obstructive pulmonary disease patients to maintain proper inhaler technique
Cumulated ambulation score in hospitalized patients with osteoporotic vertebral fractures is an important predictor of returning home: a retrospective cohort study
Dopamine facilitates the translation of physical exertion into assessments of effort
Spinal pseudoarthrosis following osteoporotic vertebral fracture: prevalence, risk factors, and influence on patients,Äô activities of daily living 1¬†year after injury
Resting-state functional-MRI in iNPH: can default mode and motor networks changes improve patient selection and outcome? Preliminary report
Leading mediators of sex differences in the incidence of dementia in community-dwelling adults in the UK Biobank: a retrospective cohort study
Association of Whole Grain Consumption and Cognitive Decline: An Investigation from a Community-Based Biracial Cohort of Older Adults
Association of Sarcopenia and Its Defining Components with the Degree of Cognitive Impairment in a Memory Clinic Population
Prevalence and the association between clinical factors and Diabetes-Related Distress (DRD) with poor glycemic control in patients with type 2 diabetes: A Northern Thai cross-sectional study
Falls and Sleep Disorders in Spanish Alzheimer,Äôs Disease in Nursing Homes: An Observational Study
Association between Mineral Intake and Cognition Evaluated by Montreal Cognitive Assessment (MoCA): A Cross-Sectional Study
Developing a predictive model for mortality in patients with cognitive impairment
Cognitive decline in older adults in the UK during and after the COVID-19 pandemic: a longitudinal analysis of PROTECT study data
Chronic traumatic encephalopathy-neuropathologic change in a routine neuropathology service: 7-year follow-up
Time-efficient, high-resistance inspiratory muscle strength training increases cerebrovascular reactivity in midlife and older adults
Family caregivers' satisfaction with telerehabilitation and follow-up intervention for older people with dementia: Randomized clinical trial
Healthy brain aging and delayed dementia in Texas rural elderly
Social determinants of health and cognitive function: A cross-sectional study among men without dementia
Modifiable risk factors for dementia, cognition, and plasma phosphorylated tau 181 in a large-scale cohort of Australian older adults
Protocol for the Brain Health Support Program Study of the Canadian Therapeutic Platform Trial for Multidomain Interventions to Prevent Dementia (CAN-THUMBS UP): A Prospective 12-Month Intervention Study
Associations between psychotropic and anti-dementia medication use and falls in community-dwelling older adults with cognitive impairment
Alleviating the social, health, and economic costs of apathy in dementia
Association of Motor Function With Cognitive Trajectories and Structural Brain Differences A Community-Based Cohort Study
Association of Anemia with Cognitive Function and Dementia Among Older Adults: The Role of Inflammation
Cross-Sectional Analysis of Periodontal Disease and Cognitive Impairment Conducted in a Memory Clinic: The Pearl Study
Dietary Sugar Intake Associated with a Higher Risk of Dementia in Community-Dwelling Older Adults
Acute myocardial infarction is associated with faster age-related cognitive decline
Association of cardiometabolic multimorbidity with motoric cognitive risk syndrome in older adults
Long-term trajectories of frailty phenotype in older cancer survivors: a nationally representative longitudinal cohort study
Innovative approaches to analysing aged care falls incident data: international classification for patient safety and correspondence analysis
Minimal Clinically Important Differences in Gait and Balance Ability in Patients Who Underwent Corrective Long Spinal Fusion for Adult Spinal Deformity
Association between Mobile Phone Use and Incidence of Dementia: A Prospective Cohort Study Using the UK Biobank
Risk Factors of Progression to Cognitive Frailty: Singapore Longitudinal Ageing Study 2
Identification of preclinical dementia according to ATN classification for stratified trial recruitment: A machine learning approach
Effects of Polyphenol-Rich Foods on Chronic Diseases
Association of Lung Function with Cognitive Decline and Incident Dementia in the Atherosclerosis Risk in Communities Study
Frailty prevalence and efficient screening in primary care-based memory clinics
Walking path images from real-time location data predict degree of cognitive impairment
Comorbidity between major depressive disorder and physical diseases: a comprehensive review of epidemiology, mechanisms and management

Influence of exercise training on the left atrium: implications for atrial fibrillation, heart failure, and stroke
Association between dairy intake and risk of incident dementia: the Ohsaki Cohort 2006 Study
Cognitive impairment and health-related quality of life amongst older Australians: evidence from a longitudinal investigation
Metabolic syndrome and cognitive deficits in the Greek cohort of Epirus Health Study
Geriatric Services Hub ‚Äî A Collaborative Frailty Management Model between The Hospital and Community Providers
Associations between dietary patterns and dementia-related neuroimaging markers
Circulating Omega-3 and Omega-6 Fatty Acids, Cognitive Decline, and Dementia in Older Adults
Association of Long-term Exposure to Air Pollution and Dementia Risk: The Role of Homocysteine, Methionine, and Cardiovascular Burden
VRADA training system as a non-pharmacological dual intervention to alleviate symptoms of the pathophysiology of Mild Cognitive Impairment
Interactive association between insomnia symptoms and sleep duration for the risk of dementia‚Äîa prospective study in the Swedish National March Cohort
Prediction of injurious falls in older adults using digital gait biomarkers extracted from large-scale wrist sensor data
Hypotension and cognitive impairment among the elderly: Evidence from the CLHLS
Clinical Significance of Physical Frailty in Subjects With Subjective Cognitive Decline: A Prospective Study With Amyloid PET Data
Variety and Duration of Different Sedentary Behaviors, Inflammation, Genetic Susceptibility, and New-Onset Dementia in the Older Population
Cognitive disorders in patients with chronic kidney disease: Approaches to prevention and treatment
Weaving Evidence into Action for Veterans with Dementia (WEAVE): Evaluation of implementation into long-term care practice
Protecting cognitive function in older adults with age-related hearing loss: Insights from The Irish Longitudinal Study on Ageing (TILDA) and the role of hearing aids
Trajectories of Disability and Related Factors in Korean Adults Aged ‚â•75¬†years
Neuroimaging-Derived Predicted Brain Age and Alcohol Use Among Community-Dwelling Older Adults
Psychosocial interventions for reducing antipsychotic medication in care home residents
Incidence of Alzheimer's Disease in Men with Late-Life Hypertension Is Ameliorated by FOXO3 Longevity Genotype
Motoric Cognitive Risk Syndrome and Traffic Incidents in Older Drivers in Japan
Relationship of Neural Correlates of Gait Characteristics and Cognitive Dysfunction in Patients with Mild Cognitive Impairment
Associations of Depressive Symptoms with Subjective Cognitive Decline in Elderly People‚ÄîA Cross-Sectional Analysis from the AgeWell.de-Study
Type 2 diabetes remission trajectories and variation in risk of diabetes complications: A population-based cohort study
Intake of the Total, Classes, and Subclasses of (Poly)Phenols and Risk of Prostate Cancer: A Prospective Analysis of the EPIC Study
Cerebral autosomal recessive arteriopathy with subcortical infarcts and leukoencephalopathy (CARASIL): A challenging diagnosis and a rare multiple sclerosis mimic
The age-dependent association of Life's Simple 7 with transitions across cognitive states after age 60
Prediction of Incident Depression in Middle-Aged and Older Adults using Digital Gait Biomarkers Extracted from Large-Scale Wrist Sensor Data
The association between personality and plasma biomarkers of astrogliosis and neuronal injury
Socially Assistive Robots for People Living with Dementia in Long-Term Facilities: A Systematic Review and Meta-Analysis of Randomized Controlled Trials
A randomized controlled trial examining the effects of behavioral weight loss treatment on hippocampal volume and neurocognition
Physical activity in vascular cognitive impairment: Systematic review with meta-analysis
Potentially inappropriate medication use is associated with increased risk of incident disability in healthy older adults
Rest-Activity Rhythm Characteristics Associated With Depression Symptoms in Stroke Survivors
The association of MIND diet with cognitive resilience to neuropathologies
Obesity impacts the expression of Alzheimer's disease‚Äìrelated genes: The Framingham Heart Study
Sex differences in dementia risk and risk factors: Individual-participant data analysis using 21 cohorts across six continents from the COSMIC consortium
Biopsychosocial frailty and mild cognitive impairment subtypes: Findings from the Italian project on the epidemiology of Alzheimer's disease (IPREA)
De novo appearance of cerebral microbleeds in community-dwelling older adults. Neuroimaging and clinical correlates
Marital Histories and Associations With Later-Life Dementia and Mild Cognitive Impairment Risk in the HUNT4 70+ Study in Norway
Cohort profile: NgƒÅ Kawekawe o Mate Korona | Impacts of COVID-19 in Aotearoa - a prospective, national cohort study of people with COVID-19 in New Zealand
Development of a Gait Feature-Based Model for Classifying Cognitive Disorders Using a Single Wearable Inertial Sensor
Effect of exercise on quality of life of patients with Chronic Kidney Disease
Association of physical activity with dementia and cognitive decline in UK Biobank
Neutrophil‚ÄìLymphocyte Ratio as a Predictor of Cerebral Small Vessel Disease in a Geriatric Community: The I-Lan Longitudinal Aging Study
Managing diabetes and dementia, a challenging duo: a scoping review
Falls and Fall-Related Injuries in an Evidence-Based Tai Ji Quan Intervention in Rural West Virginia Churches
Association of Unhealthy Lifestyle and Genetic Risk Factors With Mild Cognitive Impairment in Chinese Older Adults
Functional topography of the neocortex predicts covariation in complex cognitive and basic motor abilities
Health effects of low emission and congestion charging zones: a systematic review
Elevated C-reactive protein mediates the liver-brain axis: a preliminary study

Health characteristics and aspirin use in participants at the baseline of the aspirin in reducing events in the elderly ,Äì eXTension (ASPREE-XT) observational study
Mid-life leukocyte telomere length and dementia risk: An observational and mendelian randomization study of 435,046 UK Biobank participants
Subjective Cognitive Decline as a predictor of Frailty in older adults: Hellenic Longitudinal Investigation of Aging and Diet study (HELIAD)
Social frailty as a predictor of adverse outcomes among older adults: a systematic review and meta-analysis
Associations of Midlife Dietary Patterns with Incident Dementia and Brain Structure: Findings from the UK Biobank Study
Educational Attainment and Dementia: Mediation by Mid-Life Vascular Risk Factors
Association between behavioural risks and Alzheimer's disease: Elucidated with an integrated analysis of gene expression patterns and molecular mechanisms
Letter to the editor regarding ,ÄúThe effect of depressive symptoms on disability-free survival in healthy older adults: A prospective cohort study by Roebuck et al,Äù
Loneliness and Dementia in Context
Body fat and components of sarcopenia relate to inflammation, brain volume, and neurometabolism in older adults
Examination of potentially modifiable dementia risk factors across the adult life course: The Framingham Heart Study
The relationship between objective physical activity and change in cognitive function
Predictors of falls in older adults with and without dementia
Eight-Year Trajectories of Late-Life Loneliness and Incident Dementia: A Nationally Representative Cohort Study
A propensity score-matched comparison of patient satisfaction following periacetabular osteotomy or total hip arthroplasty for developmental dysplasia of the hip in an Asian cohort
Prevalence of dementia and the attributable contributions of modifiable risk factors in China
Retinal Functional and Structural Neural Indices: Potential Biomarkers for the Monitoring of Cerebral Neurodegeneration: The Maastricht Study
Effects of insomnia and its subtypes on cognitive impairment in Chinese elderly: an analysis on data from a nationwide survey and a follow-up study in Hubei province
Role Of Vitalstim¬Æ In The Management Of Patients With Dysphagia: A Mini-Review
Association of Motoric Cognitive Risk Syndrome with Sarcopenia and Systemic Inflammation in Pre-Frail Older Adults
Influence of Sensory Impairments on Incidence of Dementia in the Korean Population
Multimorbidity Patterns in Older Adults: the Role of Social Variables and Lifestyle Behaviors
Earlier chronotype in midlife as a predictor of accelerated brain aging: a population-based longitudinal cohort study
Diabetes and Mental Health
Examining later-in-life health risks associated with sport-related concussion and repetitive head impacts: A systematic review of case-control and cohort studies
Serum Adiponectin and in Vivo Brain Amyloid Deposition in Cognitively Normal Older Adults: A Cohort Study
Awareness of diagnosis predicts changes in quality of life in individuals with mild cognitive impairment and mild stage dementia
A 92-year-old patient with thoracic empyema successfully treated by CT-guided insertion of a pigtail catheter
Cognitive Impairment After Ischemic and Hemorrhagic Stroke: A Scientific Statement From the American Heart Association/American Stroke Association
Combining exercise with cognitive training and vitamin D3 to improve functional brain connectivity (FBC) in older adults with mild cognitive impairment (MCI). Results from the SYNERGIC trial
Improvement in apathy and depression by non-pharmacological interventions in early-onset Alzheimer's disease: A longitudinal single-photon emission computed tomography study
Interrelation between functional decline and dementia: The potential role of balance assessment
How do forms and characteristics of Asian public housing neighbourhoods affect dementia risk among senior population? A cross-sectional study in Hong Kong
Longitudinal trajectories of dietary quality and cognitive performance in older adults: Results from a 6-year cohort study
Annual stability of the plasma A√ü40/42 ratio and associated factors
Former participation in professional football as an occupation in patients with isolated REM sleep behavior disorder leading to a synucleinopathy: a case,Äìcontrol study
Sex Hormones and Cardiovascular Disease in Relation to Menopause
The association between longitudinal changes in depressive symptoms and cognitive decline among middle-aged and older Chinese adults
Effectiveness of exercise programmes in improving physical function and reducing behavioural symptoms of community living older adults with dementia living in Asia, and impact on their informal carers: A systematic review and meta-analysis
Understanding changes in mental health symptoms from young-old to old-old adults by sex using multiple-group latent transition analysis
Is the Sport Concussion Assessment Tool 5th Edition a useful concussion assessment tool in head-injured patients presenting to the emergency department?
Cerebrospinal fluid irisin and lipoxin A4 are reduced in elderly Brazilian individuals with depression: Insight into shared mechanisms between depression and dementia
Prognostic implications of six-minute walking distance in patients with heart failure with preserved ejection fraction
Trimethylamine N-oxide is associated with long-Term mortality risk: The multi-ethnic study of atherosclerosis
Spatial orientation, postural control and the vestibular system in healthy elderly and Alzheimer,Äôs dementia
Quality of plant-based diet and the risk of dementia and depression among middle-aged and older population
Effects of Multidisciplinary Rehabilitation Enhanced with Neuropsychological Treatment on Post-Acute SARS-CoV-2 Cognitive Impairment (Brain Fog): An Observational Study
Blood biomarkers for Alzheimer,Äôs disease in clinical practice and trials

Detection and treatment of Alzheimer‚Äôs disease in its preclinical stage
A Case Report of Reversible Cognitive Decline in a Former Professional American-Style Football Player: Findings from the Football Players Health Study In-Person Assessments
The Impact of Atrial Fibrillation Treatment Strategies on Cognitive Function
Pre-fracture ambulation capacity, Charlson comorbidity index, and dementia as predictors of functional impairment after bipolar hemiarthroplasty for unstable intertrochanteric fracture. A retrospective analysis in 158 octogenarian patients
Association between hearing aid use and all-cause and cause-specific dementia: an analysis of the UK Biobank cohort
Toward developing care outcome quality indicators for home care for older people: A prospective cohort study in Japan
Neurocognitive test performance following cancer among middle-aged and older adults in the Hispanic Community Health Study/Study of Latinos (HCHS/SOL) and the SOL-Investigation of Neurocognitive Aging Ancillary Study
Design and development of the first randomized controlled trial of an intervention (IDEA) for sexual and gender minority older adults living with dementia and care partners
Identification of plasma metabolites associated with modifiable risk factors and endophenotypes reflecting Alzheimer‚Äôs disease pathology
Association of Alzheimer's dementia with oral bacteria, vitamin B12, folate, homocysteine levels, and insulin resistance along with its pathophysiology, genetics, imaging, and biomarkers
Alternative medicine therapies in neurological disorders: Prevalence, reasons and associated factors. A systematic review
Bridging the gap: Study protocol for development of an implementation strategy for evidence-informed reablement and rehabilitation for community-dwelling people with dementia
The Risk for Loneliness and Major Depression among Solo Agers
Adiposity in the older population and the risk of dementia: The Rotterdam Study
Informal care in Huntington's disease: Assessment of objective-subjective burden and its associated risk and protective factors
Association of Long-term Exposure to Ambient Air Pollution With Cognitive Decline and Alzheimer's Disease‚ÄìRelated Amyloidosis
Correlation between serum lipid profiles and cognitive impairment in old age: a cross-sectional study
Shared Risk Factors for Depressive Disorder among Older Adult Couples in Korea
Early and Accurate Diagnosis of a Neurological Disorder Epilepsy Using Machine Learning Techniques
Multifactorial effects of outpatient cardiac rehabilitation in patients with heart failure: a nationwide retrospective cohort study
Mid-and Late-Life Physical Activity and Neuropsychiatric Symptoms in Dementia-Free Older Adults: Mayo Clinic Study of Aging
Cognitive reserve and dementia risk management in people with an intellectual disability
App-based intervention for reducing depressive symptoms in postpartum women: Protocol for a feasibility randomized controlled trial
Order of Onset of Physical Frailty and Cognitive Impairment and Risk of Repeated Falls in Community-Dwelling Older Adults
The aging brain: Risk factors and interventions for long term brain health in women
2023 Alzheimer's disease facts and figures
Patient-level and system-level determinants of stroke fatality across 16 large hospitals in Ghana and Nigeria: a prospective cohort study
Impact of interventions scenarios targeting three main vascular risk factors on the future burden of dementia in France
Cognitive Decline Before and During COVID-19 Pandemic Among Older People With Multimorbidity: A Longitudinal Study
Mediterranean diet in the management and prevention of obesity
Linking early-life bilingualism and cognitive advantage in older adulthood
Plasma inflammation-related biomarkers are associated with intrinsic capacity in community-dwelling older adults
Brain connectivity in frailty: Insights from The Irish Longitudinal Study on Ageing (TILDA)
Obesity, multiple chronic conditions, and the relationship with physical function: Data from the national health and aging trends survey
Effects of telehealth on functional capacity, mental health and quality of life among older people with dementia: LAPESI telehealth protocol for a randomized controlled trial
Associations of grip strength, walking pace, and the risk of incident dementia: A prospective cohort study of 340212 participants
Associations between physical activity and cognitive dysfunction in older companion dogs: results from the Dog Aging Project
Personally tailored activities for improving psychosocial outcomes for people with dementia in long-term care
Association of Plasma High-Density Lipoprotein Cholesterol Level with Risk of Fractures in Healthy Older Adults
Studying and learning
Relative importance of potential risk factors for dementia in patients with hypertension
Physical Activity Is Associated With Macular Thickness: A Multi-Cohort Observational Study
Association of Religious Service Attendance and Neuropsychiatric Symptoms, Cognitive Function, and Sleep Disturbances in All-Cause Dementia
Crosstalk between Oxidative Stress and Aging in Neurodegeneration Disorders
Sensorimotor and Frontoparietal Network Connectivity Are Associated With Subsequent Maintenance of Gait Speed and Episodic Memory in Older Adults
Midlife omega-3 fatty acid intake predicts later life white matter microstructure in an age- and APOE-dependent manner
Exercise Protects Against Cognitive Injury and Inflammation in Alzheimer's Disease Through Elevating miR-148a-3p
Current Evidence on Prevention of Atrial Fibrillation: Modifiable Risk Factors and the Effects of Risk Factor Intervention
Efficacy and safety of oxiracetam in patients with vascular cognitive impairment: A multicenter, randomized, double-blinded, placebo-controlled, phase IV clinical trial

Association of gait speed with regional brain volumes and risk of dementia in older Japanese: The Hisayama study
Know diabetes by heart: Education about the linkage between cardiovascular disease and diabetes
Physical activity and brain health in patients with atrial fibrillation
Influence of Sense of Competence, Empathy and Relationship Quality on Burden in Dementia Caregivers: A 15 Months Longitudinal Study
The impact of the metabolic score for insulin resistance on cardiovascular disease: a 10-year follow-up cohort study
Cardiometabolic multimorbidity accelerates cognitive decline and dementia progression
Cardiometabolic multimorbidity and incident dementia: The Swedish twin registry
The complexity of inflammatory activity in caregivers: Commentary on Associations of perceived stress, depressive symptoms, and caregiving with inflammation: A longitudinal study by Elayoubi et al.
Characteristics of Cognitive Function Changes and Related Factors in Individuals With Cognitive Impairment During the Pandemic of COVID-19: A Retrospective Chart Review Study
Association between stressful life events and non-optimal lipid levels among women with hyperlipidaemia
Association of Sarcopenia with Cognitive Function and Dementia Risk Score: A National Prospective Cohort Study
‚ÄúWe Just Don‚Äôt Know Where They Are‚Äù: The Geographical Distribution of Exercise Classes for Older People, Including Those Living with Dementia in the East Midlands
Urinary Hydration Biomarkers and Water Sources in Older Adults with Neurocognitive Disorder
Systemic exertion intolerance disease associated to neuroendocrine dysfunction and cortical atrophy: a case report
Long-Term Mortality of Tuberculosis Survivors in Korea: A Population-based Longitudinal Study
Air pollution and plasma amyloid beta in a cohort of older adults: Evidence from the Ginkgo Evaluation of Memory study
Prevalence of Drug‚ÄìDrug Interactions in Older Community-Dwelling Individuals: A Systematic Review and Meta-analysis
Neural signature of mobility-related everyday function in older adults at-risk of cognitive impairment
Oral frailty as a risk factor for mild cognitive impairment in community-dwelling older adults: Kashiwa study
A systematic review and meta-analysis of the prevalence and correlation of mild cognitive impairment in sarcopenia
Progression of frailty as measured by a cumulative deficit index: A systematic review
Low Evidence for Tinnitus Risk Factors: A Systematic Review and Meta-analysis
Sense of meaning and purpose in life and risk of incident dementia: New data and meta-analysis
Cardiometabolic diseases, polygenic risk score, APOE genotype, and risk of incident dementia: A population-based prospective cohort study
Reduced calf pump function and proximal deep vein incompetence are predictors for ipsilateral deep vein thrombosis
Cognitive decline among older adults with heart diseases before and during the COVID-19 pandemic: A longitudinal cohort study
Development of risk prediction model for cognitive impairment in patients with coronary heart disease: A study protocol for a prospective, cross-sectional analysis
Intracranial artery stenosis is associated with cortical thinning in stroke-free individuals of two longitudinal cohorts
Executive function and relation to static balance metrics in chronic mild TBI: A LIMBIC-CENC secondary analysis
Impact of multidomain preventive strategies on functional brain connectivity in older adults with cognitive complaint: Subset from the Montpellier center of the ancillary MAPT-MRI study
Effectiveness of physical rehabilitation in improving physical functioning and quality of life for long-term-care residents with dementia: a systematic review protocol
Prevention of physical restraints in the acute care setting (PROTECT): study protocol for a cluster-randomised controlled pilot study
Childhood Maltreatment and Dementia Risk Factors in Midlife: A Prospective Investigation
Effects of remote combine exercise-music training on physical and cognitive performance in patients with Alzheimer‚Äôs disease: a randomized controlled trial
Biais cognitifs dans le diagnostic de maladie d‚ÄôAlzheimer
The function of previously unappreciated exerkines secreted by muscle in regulation of neurodegenerative diseases
Fasting glucose variability and risk of dementia in Parkinson‚Äôs disease: a 9-year longitudinal follow-up study of a nationwide cohort
Multi-modal sleep intervention for community-dwelling people living with dementia and primary caregiver dyads with sleep disturbance: protocol of a single-arm feasibility trial
An Online Shared Decision-making Intervention for Dementia Prevention: A Parallel-group Randomized Pilot Study
Association between the triglyceride glucose index and cognitive impairment and dementia: a meta-analysis
New anti-aging strategies: a narrative review
Relationship between hippocampal subfield volumes and cognitive decline in healthy subjects
The frail patient undergoing cardiac surgery: lessons learned and future perspectives
Association between non-cystic fibrosis bronchiectasis and the risk of incident dementia: A nationwide cohort study
Diabetes status and cognitive function in middle-aged and older adults in the Canadian longitudinal study on aging
The predictive validity of a Brain Care Score for dementia and stroke: data from the UK Biobank cohort
The Effect of Tele-Savvy on Sleep Quality and Insomnia in Caregivers of Persons Living with Dementia
A Wearable System for Continuous Monitoring and Assessment of Speech, Gait, and Cognitive Decline for Early Diagnosis of ADRD
Associations of sarcopenia, sarcopenia parameters and motoric cognitive risk syndrome in Chinese older adults
Somatic comorbidity and the progression of cognitive impairment
Ranking the risk factors for Alzheimer's disease; findings from the UK Biobank study

The impact of loneliness and social isolation during COVID-19 on cognition in older adults: a scoping review
Associations between circulating cardiovascular disease risk factors and cognitive performance in cognitively healthy older adults from the NuAge study
Behaviour Disturbances (Dementia)
An explainable machine learning based prediction model for Alzheimer's disease in China longitudinal aging study
The Neuropathological Impacts of COVID-19: Challenges and Alternative Treatment Options for Alzheimer‚Äôs Like Brain Changes on Severely SARS-CoV-2 Infected Patients
Rationale and design of STAMINA: Senolytics to alleviate mobility issues and neurological impairments in aging, a geroscience feasibility study
Analysis on Association Between Vascular Risk Factors and Lifestyle Factors with the Risk of Dementia/Alzheimer‚Äôs Disease Using Medical Ontologies
Uncovering Methods and Outcomes of Palliative Care for Geriatric Patients: A Scoping Review
Analysis of clinical characteristics and risk factors of sarcopenia in elderly patients with chronic kidney disease stage 3-4
Pose2Gait: Extracting Gait Features from¬†Monocular Video of¬†Individuals with¬†Dementia
Risk Factors and Prediction Nomogram of Cognitive Frailty with Diabetes in the Elderly
Addressing the Chronic Pain-Early Cognitive Decline Comorbidity Among Older Adults: Protocol for the Active Brains Remote Efficacy Trial
Corticobasal Syndrome as Alzheimer‚Äôs Disease Subtype: A Case Report
Impact of a multidomain lifestyle intervention on white matter integrity: the SUPERBRAIN exploratory sub-study
The impact of neuropsychological functions on self-care/self-management of type 2 diabetes in middle-aged people: a scoping review and meta-analysis
Functional Dependency and Its Associated Factors among Older Adults in Indonesia
An Automated Digital Biomarker of Mobility
Assessment of factors influencing glymphatic activity and implications for clinical medicine
Cardiorespiratory Fitness Attenuates the Deleterious Effects of Sleep Apnea on Cerebral Structure and Perfusion in the Wisconsin Sleep Cohort Study
Association of Physical Inactivity with MRI Markers of Brain Aging: Assessing Mediation by Cardiometabolic and Epigenetic Factors
ADULT-GERONTOLOGY PRACTICE GUIDELINES: THIRD EDITION
Factors associated with cognitive impairment in Latin American older adults: A cross-sectional observational study of COVID-19 confinement
Multimorbid Patterns and Cognitive Performance in the Presence of Informative Dropout Among Community-Dwelling Taiwanese Older Adults
Editorial: Movement behaviors (sleep, sedentary behavior and physical activity) and physical and mental/cognitive health
Prevalence of dementia and its correlation with multimorbidity in community?dwelling older adults aged 65 and over in Hubei Province
Observed Improvement in Cognition during a Personalized Lifestyle Intervention in People with Cognitive Decline
Using Hypothesis-Led Machine Learning and Hierarchical Cluster Analysis to Identify Disease Pathways Prior to Dementia: Longitudinal Cohort Study
A Study on the Effect of Traditional Chinese Exercise Combined With Rhythm Training on the Intervention of Older Adults With Mild Cognitive Impairment
Chronic Neurocognitive, Neuropsychological, and Pulmonary Symptoms in Outpatient and Inpatient Cohorts After COVID-19 Infection
Effectiveness of a short-term multimodal prehabilitation program in adult patients awaiting selective cardiac surgery: study protocol for an open-label, pilot, randomized controlled trial
Gulf War Illness: A Randomized Controlled Trial Combining Mindfulness Meditation and Auricular Acupuncture
The effect of physical activity on white matter integrity in aging and prodromal to mild Alzheimer‚Äôs disease with vascular comorbidity
Winning the race with aging: age-related changes in gait speed and its association with cognitive performance in dogs
The Prevalence and Agreement of Sarcopenic Obesity Using Different Definitions and Its Association with Mild Cognitive Impairment
Obesity in Late-Life as a Protective Factor Against Dementia and Dementia-Related Mortality
Best Medicine for Dementia: The Life-Long Defense of the Brain
Leg Length Discrepancy After Hip Fracture Repair is Associated With Reduced Gait Speed
The impact of non-drug treatments on the quality of life of patients with non-dementia vascular cognitive impairment
Cognitive Reserve: A Life-Course Perspective
Case report: Treatment of advanced CSF1-receptor associated leukoencephalopathy with hematopoietic stem cell transplant
Clinical Trials in Fragile X-Associated Tremor/Ataxia Syndrome
Depression and Anxiety in Older Adults with Dementia During the COVID-19 Pandemic
Cognitive impairment in long-COVID and its association with persistent dysregulation in inflammatory markers
A cross-sectional study of knowledge, attitude, behaviour and preventive measures for COVID-19 infection in Lebanon
Vulnerability of Parkinson's Patients to COVID-19 and Its Consequences and Effects on Them: A Systematic Review
A Multidomain Intervention Program for Older People with Dementia: A Pilot Study
Network analysis of the associations between personality traits, cognitive functioning, and inflammatory markers in elderly individuals without dementia
Case report: A longitudinal study of an unusual rapidly progressive dementia case
Visual-somatosensory integration (VSI) as a novel marker of Alzheimer‚Äôs disease: A comprehensive overview of the VSI study
Efficacy of a combination of non-drug therapies in patients with non-dementia vascular cognitive impairment
2nd International Conference on ICT for Health, Accessibility and Wellbeing, IHAW 2022
The Importance of Dopamine Deficiency Evaluation in Non-Alzheimer Disease Dementias

A non-pharmacological multidomain intervention of dual-task exercise and social activity affects the cognitive function in community-dwelling older adults with mild to moderate cognitive decline: A randomized controlled trial
A digital health home intervention for people within the Alzheimer‚Äôs disease continuum: results from the Ability-TelerehABILITation pilot randomized controlled trial
Determinants of participation in cardiac telerehabilitation during the first surge of COVID-19
An atypical ALS with PSP-like symptoms caused by ANXA11 p.D40G mutation: A case report and literature review
The Independent Walking for Brain Health Intervention for Older Adults: Protocol for a Pilot Randomized Controlled Trial
Carotid Intima-Media Thickness (cIMT) and Cognitive Performance: A Population-Based Cross-Sectional Study from North India
Preoperative Vitamin D Deficiency is Associated with Increased One-Year Mortality in Chinese Geriatric Hip Fracture Patients ‚Äì A Propensity Score Matching Study
Topical Review of Hobbies and Cognitive Health
Rationale and Design of the PARTNER Trial: Partnered Rhythmic Rehabilitation for Enhanced Motor-Cognition in Prodromal Alzheimer's Disease
Data-driven decision making for the screening of cognitive impairment in primary care: a machine learning approach using data from the ELSA-Brasil study
Mild Cognitive Impairment, Reversion Rates, and Associated Factors: Comparison of Two Diagnostic Approaches
How to Improve the Quality of Life of Patients with Prostate Cancer Treated with Hormone Therapy?
The association of chronotype and social jet lag with body composition in German students: The role of physical activity behaviour and the impact of the pandemic lockdown
Sex Differences in the Burden of Hip Fractures on Functional Status in Older Age
Association of Physical Activity With Neurofilament Light Chain Trajectories in Autosomal Dominant Frontotemporal Lobar Degeneration Variant Carriers
Concordance of Alzheimer's Disease-Related Biomarkers between Intraventricular and Lumbar Cerebrospinal Fluid in Idiopathic Normal Pressure Hydrocephalus
Low Serum Vitamin D Status Is Associated with Incident Alzheimer‚Äôs Dementia in the Oldest Old
Neighborhood greenspace and cognition: The cardiovascular health study
Trends in Heart Failure Outcomes From a Large, Geographically Diverse Cohort During the COVID-19 Pandemic
Factors associated with cognitive impairment at 3, 6, and 12 months after the first stroke among Lebanese survivors
4. Comprehensive Medical Evaluation and Assessment of Comorbidities: Standards of Care in Diabetes‚Äî2023
Physical activity and diabetes mortality in people with type 2 diabetes: a prospective cohort study of 0.5 million US people
The Association between Metabolic Syndrome, Frailty and Disability-Free Survival in Healthy Community-dwelling Older Adults
Cognitive trajectories preluding the imminent onset of Alzheimer‚Äôs disease dementia in individuals with normal cognition: results from the HELIAD cohort
Long-term depressive symptoms and midlife brain age
The long-term relation between physical activity and executive function in the Rotterdam Study
Peripheral Vascular Disease is Associated with Impaired Activities of Daily Living in the Elderly with Dementia: A Prospective Study
Remotely Administered Resilience- and Wisdom-Focused Intervention to Reduce Perceived Stress and Loneliness: Pilot Controlled Clinical Trial in Older Adults
Effects of Client-Centered Occupational Therapy on Behavioral Psychological Symptoms, Social Interaction, Occupational Performance, Quality of Life, and Caregiver Burden among the Individuals with Dementia
Association of dietary inÔ¨Çammatory index and leukocyte telomere length with mild cognitive impairment in Chinese older adults
Study protocol for the BRAIN Training Trial: A randomised controlled trial of Balance, Resistance, and INterval training on cognitive function in older adults with mild cognitive impairment
The role of Mediterranean diet in the course of subjective cognitive decline in the elderly population of Greece: results from a prospective cohort study
Effects of Mindfulness Training and Exercise on Cognitive Function in Older Adults: A Randomized Clinical Trial
Prevalence and risk associations of mild cognitive impairment in community-dwelling elderly people
Prevalence and Influencing Factors of Motoric Cognitive Risk Syndrome in the Elderly with Subjective Cognitive Decline in the Community
Five-year outcomes of major lower extremity amputation and two-staged admission pathway for new transtibial amputees in a local hospital
To Study the efficacy of Plumbum Metallicum Homoeopathic Medicine in improving the Cognitive function in Elderly
Risk of dementia according to the smoking habit change after ischemic stroke: a nationwide population-based cohort study
Remission of Type 2 Diabetes: Diabetes Canada Clinical Practice Guidelines Expert Working Group:
Real-World Results from Combined Screening for Monogenic Genomic Health Risks and Reproductive Risks in 300 Adults
Differences in Subjective and Objective Cognitive Decline Outcomes Are Associated with Modifiable Protective Factors: A 4-Year Longitudinal Study
Multi-organ imaging demonstrates the heart-brain-liver axis in UK Biobank participants
Dual tasking paradigm in clinical stages of early Alzheimer‚Äôs disease
Health behaviors and advanced planning in cognitively unimpaired older adults following amyloid PET disclosure: Preliminary findings from the Wisconsin Registry for Alzheimer‚Äôs Prevention
Interplay of the human exposome, metabolome and gut microbiome in dementia and major depression
Diabetes and hypertension are associated with elevated beta-amyloid burden: evidence from the prospective population-based Rotterdam Study
Spousal caregiver burden among patient with cognitive impairment: effects on nutrition and other lifestyle factors
Association between loneliness and dementia risk: A systematic review and meta-analysis of cohort studies
Food intake and prevalence of malnutrition in nursing homes. A multicenter observational study
Modified dementia risk score as a tool for the prediction of dementia: a prospective cohort study of 239745 participants

Older adults with perivascular spaces exhibit cerebrovascular reactivity deficits
Myocardial contraction fraction predicts mortality in the oldest old
Convergent validity of EQ-5D with core outcomes in dementia: a systematic review
Patterns of polyphenol intake and risk of depressive symptomatology in a population-based cohort of older adults
Epidemiology and Risk Factors for Dementia
Age-Related Association between Multimorbidity and Mortality in US Veterans with Incident Chronic Kidney Disease
Randomised controlled trials for the prevention of cognitive decline or dementia: A systematic review
A potential biomarker of cognitive impairment: The olfactory dysfunction and its genes expression
Proinflammatory dietary pattern and depression risk in older adults: Prospective analyses from the Seniors-ENRICA studies
Wasting syndrome and associated factors in hospitalized older people
Ambient air pollution and risk of incident dementia in older men living in a region with relatively low concentrations of pollutants: The Health in Men Study
Association of cardiovascular health with the risk of dementia in older adults
Effects of a mindfulness-based versus a health self-management intervention on objective cognitive performance in older adults with subjective cognitive decline (SCD): a secondary analysis of the SCD-Well randomized controlled trial
A systematic review on the risk of neurodegenerative diseases and neurocognitive disorders in professional and varsity athletes
Gender-Specific Association Between Nighttime Sleep Duration and Falls Among Community-Dwelling Middle-Aged and Older Adults in China
Social determinants and lifestyle factors for brain health: implications for risk reduction of cognitive decline and dementia
The effects of exercise programs on cognition, activities of daily living, and neuropsychiatric symptoms in community-dwelling people with dementia,Äîa systematic review
Low physical activity is the strongest factor associated with frailty phenotype and frailty index: data from baseline phase of Birjand Longitudinal Aging Study (BLAS)
Type 1 neurodegeneration with brain iron accumulation: a case report
Use of massage therapy by mid-aged and older Australian women
An updated systematic review of interventions to increase awareness of mental health and well-being in athletes, coaches, officials and parents
Explaining the association between social and lifestyle factors and cognitive functions: a pathway analysis in the Memento cohort
Effects of aerobic exercise on quality of life of people with HIV-associated neurocognitive disorder on antiretroviral therapy: a randomised controlled trial
Time spent in outdoor light is associated with the risk of dementia: a prospective cohort study of 362094 participants
Three-week exercise and protein intake immediately after exercise increases the 6-min walking distance with simultaneously improved plasma volume in patients with chronic cerebrovascular disease: a preliminary prospective study
Association Between Physical Exercise Interventions Participation and Functional Capacity in Individuals with Type 2 Diabetes: A Systematic Review and Meta-Analysis of Controlled Trials
Cerebrospinal fluid biomarkers that reflect clinical symptoms in idiopathic normal pressure hydrocephalus patients
Psychotherapeutic interventions in individuals at risk for Alzheimer,Äôs dementia: a systematic review
My Healthy Brain: Rationale and Case Report of a Virtual Group Lifestyle Program Targeting Modifiable Risk Factors for Dementia
Ketogenic diet for human diseases: the underlying mechanisms and potential for clinical implementations
MIND diet and the risk of dementia: a population-based study
Depressive symptoms and physical function among the elderly in nursing homes during the COVID-19 pandemic in China: A cross-sectional study
Prevalence and possible factors of cognitive frailty in the elderly with hypertension and diabetes
CNS Superficial Siderosis Mimicking a Motor Neuron Disease
Empirical Dietary Inflammatory Pattern Scores Are Not Associated with Worse Cognitive Performance in the Nurses,Äô Health Study
Protocol for a pre-post, mixed-methods feasibility study of the Brain Bootcamp behaviour change intervention to promote healthy brain ageing in older adults
Cost-Effectiveness of the Comprehensive Interdisciplinary Program-Care in Informal Caregivers of People with Alzheimer,Äôs Disease
The Impact of a Yoga-Based Mindfulness Intervention versus Psycho-Educational Session for Older Adults with Mild Cognitive Impairment: The Protocol of a Randomized Controlled Trial
Digital Devices for Assessing Motor Functions in Mobility-Impaired and Healthy Populations: Systematic Literature Review
Hormone Therapy for the Primary Prevention of Chronic Conditions in Postmenopausal Persons: US Preventive Services Task Force Recommendation Statement
Resting Heart Rate and Cognitive Decline: A Meta-Analysis of Prospective Cohort Studies
LETTERS TO THE EDITOR Idiopathic normal pressure hydrocephalus: is 1-year follow-up enough?
Six-month change in gait speed to discriminate between those with and without falls history in older people with Mild Cognitive Impairment and mild Alzheimer disease
A Systematic Review of the Current Evidence from Randomised Controlled Trials on the Impact of Medication Optimisation or Pharmacological Interventions on Quantitative Measures of Cognitive Function in Geriatric Patients
Rehabilitation services and related health databases, Japan
Elevated plasma zonulin and CAF22 are correlated with sarcopenia and functional dependency at various stages of Alzheimer's diseases
B vitamin intakes modify the association between particulate air pollutants and incidence of all-cause dementia: Findings from the Women's Health Initiative Memory Study
Low untreated systolic blood pressure over 18 years is associated with survival free of dementia age 90+
Rhythmic auditory cueing in atypical parkinsonism: A pilot study

Evaluation of noise regression techniques in resting-state fMRI studies using data of 434 older adults
Effect of Exercise Training or Complex Mental and Social Activities on Cognitive Function in Adults with Chronic Stroke: A Randomized Clinical Trial
Oral anticoagulants in patients with atrial fibrillation at low stroke risk: A multicentre observational study
Horizons in neuroscience research
Neurodegenerative disease risk among former international rugby union players
Association of the Composite dietary antioxidant index with all-cause and cardiovascular mortality: A prospective cohort study
Risk scores of incident mild cognitive impairment in a Beijing community-based older cohort
Hallervorden-Spatz Disease: Case Report based on Radiological and Genetic Analytical Findings
Prevalence and Risk Factors of Cognitive Impairment and its Effect on Quality of Life: A Cross-Sectional Analysis of the TILDA Cohort
Effectiveness of Computerized Cognitive Training in Delaying Cognitive Function Decline in People with Mild Cognitive Impairment: Systematic Review and Meta-analysis
Chemical and physical restraint use during acute care hospitalization of older adults: A retrospective cohort study and time series analysis
Developing a Non-Pharmacological Intervention Programme for Wandering in People with Dementia: Recommendations for Healthcare Providers in Nursing Homes
Effects of Branched-Chain Amino Acids on Skeletal Muscle, Glycemic Control, and Neuropsychological Performance in Elderly Persons with Type 2 Diabetes Mellitus: An Exploratory Randomized Controlled Trial
Prodromal Œ±-Synucleinopathies
Sex-Specific Associations of Dietary Iron Intake with Brain Iron Deposition on Imaging and Incident Dementia: A Prospective Cohort Study
Self-reported sleepiness associates with greater brain and cortical volume and lower prevalence of ischemic covert brain infarcts in a community sample
Hearing and Mobility in Aging,ÄîThe Moderating Role of Neuropsychological Function
Characterizing Lewy Pathology in 231 Essential Tremor Brains From the Essential Tremor Centralized Brain Repository
Clinical Efficacy of Multidomain Interventions among Multimorbid Older People Stratified by the Status of Physio-Cognitive Declines: A Secondary Analysis from the Randomized Controlled Trial for Healthy Aging
Is 112 the New 10000? - Step Count and Dementia Risk in the UK Biobank
Severe low back or lower limb pain is associated with recurrent falls among older Australians
Clinically meaningful change for the chair stand test: monitoring mobility in integrated care for older people
Biological determinants of blood-based cytokines in the Alzheimer's disease clinical continuum
Pressure Injury on Poststroke Admission Assessment to Skilled Nursing Facilities: Risk Factors, Management, and Impact on Rehabilitation
Modifiable risk factors for incident dementia and cognitive impairment: An umbrella review of evidence
Physical inactivity is associated with Parkinson's disease mild cognitive impairment and dementia
Validation of a ,Äúsubjective motoric cognitive risk syndrome,Äù screening tool for motoric cognitive risk syndrome,ÄîA prospective cohort study
Sleep, physical activity, sedentary behavior, and risk of incident dementia: a prospective cohort study of 431,924 UK Biobank participants
Salivary Alpha-Amylase Activity and Mild Cognitive Impairment among Japanese Older Adults: The Toon Health Study
Lean mass, grip strength, and hospital-associated disability among older adults in Health ABC
Vascular Brain Lesions, Cognitive Reserve, and Their Association with Cognitive Profile in Persons with Early-Stage Cognitive Decline
Physical activity, memory function, and hippocampal volume in adults with Down syndrome
Association Between Accelerometer-Derived Physical Activity Measurements and Brain Structure: A Population-Based Cohort Study
Plasma metabolomics and lipidomics signatures of motoric cognitive risk syndrome in community-dwelling older adults
Research trends and frontiers in exercise for movement disorders: A bibliometric analysis of global research from 2010 to 2021
A Case of Possible Chronic Traumatic Encephalopathy and Alzheimer's Disease in an Ex-Football Player
Safety and efficacy of plasma transfusion from exercise-trained donors in patients with early Alzheimer's disease: protocol for the ExPlas study
Association of Ultraprocessed Food Consumption With Risk of Dementia: A Prospective Cohort Study
Frequency and gender differences in the use of professional home care in late life. Findings from three German old-age cohorts
Functional mobility decline and incident mild cognitive impairment and early dementia in community-dwelling older adults: the Singapore Longitudinal Ageing Study
Quality of Life and Depression Assessment in Patients with Acute Coronary Syndrome: A Cross-Sectional Study
Diabetes and atrial fibrillation: does the type of diabetes matter?
Identification of Community-Dwelling Older Adults With Shoulder Dysfunction: A Pilot Study to Evaluate the Disabilities of the Arm, Shoulder and Hand Survey
Associations of the Lipidome with Ageing, Cognitive Decline and Exercise Behaviours
Follow-Up of a Cohort of Patients with Post-Acute COVID-19 Syndrome in a Belgian Family Practice
Moderate increase of serum uric acid within a normal range is associated with improved cognitive function in a non-normotensive population: A nationally representative cohort study
Carotid Arterial Compliance and Aerobic Exercise Training in Chronic Traumatic Brain Injury: A Pilot Study
A Simple Resident Need-for-Physical-Assistance Scale in Eldercare: Validation Using 4716 Observation Sequences of Caring Activities
Accelerated Cognitive Function Decline in Community-Dwelling Older Adults during COVID-19 Pandemic: The Korean Frailty and Aging Cohort Study (KFACS)
Life course, genetic, and neuropathological associations with brain age in the 1946 British Birth Cohort: a population-based study
Fear of falling does not predict self-reported physical activity: an observational study with community-dwelling older adults

Black-White variation in the relationship between early educational experiences and trajectories of cognitive function among US-born older adults
PROtein enriched MEDiterranean diet to combat undernutrition and promote healthy neuroCOGnitive ageing in older adults: The PROMED-COG consortium project
Social network and the risk for developing mild cognitive impairment and dementia among older adults
Frailty and dementia risks in asymptomatic cerebral small vessel disease: A longitudinal cohort study
CSF tap test in idiopathic normal pressure hydrocephalus: still a necessary prognostic test?
The gut microbiome as a modulator of healthy ageing
Slowing gait speed precedes cognitive decline by several years
Plasma biomarkers and their correlation in adult children of parents with Alzheimer‚Äôs disease
Using Bayesian networks with Max-Min Hill-Climbing algorithm to detect factors related to multimorbidity
Association of Healthy Lifestyles With Risk of Alzheimer Disease and Related Dementias in Low-Income Black and White Americans
Leisure-time sedentary behaviors are differentially associated with all-cause dementia regardless of engagement in physical activity
Interventions for preventing and reducing the use of physical restraints of older people in general hospital settings
Effects of Sex, APOE4, and Lifestyle Activities on Cognitive Reserve in Older Adults
The effect of risk factors on cognition in adult cochlear implant candidates with severe to profound hearing loss
Ketones: Potential to achieve brain energy rescue and sustain cognitive health during ageing
The Elderly Nutritional Index for Geriatric Malnutrition Assessment (ENIGMA): Concurrent, construct and predictive validity in an external evaluation cohort of community-dwelling older persons
Nonalcoholic Fatty Liver Disease: Does This Metabolic Condition Have Brain Function Sequelae?
Exploration of combined physical activity and music for patients with Alzheimer‚Äôs disease: A systematic review
Measuring the Frailty Phenotype and its Association with Cognition in Mid-Life and Older Age
Life's Essential 8: Updating and Enhancing the American Heart Association's Construct of Cardiovascular Health: A Presidential Advisory from the American Heart Association
Gradient and Acceleration of Decline in Physical and Cognitive Functions in Older Adults: A Disparity Analysis
Screening Tool to Identify Patients with Advanced Aortic Valve Stenosis
NADPH and Mitochondrial Quality Control as Targets for a Circadian-Based Fasting and Exercise Therapy for the Treatment of Parkinson‚Äôs Disease
Frailty: understanding the difference between age and ageing
Cerebellar atrophy and its implications on gait in cerebral amyloid angiopathy
Gender-specific impact of cognitive impairment on all-cause mortality in older persons: A meta-analysis
Subclinical cognitive deficits are associated with reduced cerebrovascular response to visual stimulation in mid-sixties men
Vitamin D and brain health: an observational and Mendelian randomization study
Longitudinal associations of absolute versus relative moderate-to-vigorous physical activity with brain microstructural decline in aging
Self-reported measures of limitation in physical function in late midlife are associated with incident Alzheimer‚Äôs disease and related dementias
Macrostructural brain alterations at midlife are connected to cardiovascular and not inherited risk of future dementia: the PREVENT-Dementia study
Psychosocial determinants for adherence to a healthy lifestyle and intervention participation in the FINGER trial: an exploratory analysis of a randomised clinical trial
Osteoporosis and Related Health Status Among the Elderly Urban Residents in Elderly-Care Inns in Beijing, a Multicenter DXA Survey
Essential tremor patients experience significant burden beyond tremor: A systematic literature review
Emergency Room Evaluation and Recommendations and Risk Screening of Incident Major Neurocognitive Disorders in Older Females: Results of an Observational Population-Based Cohort Study
Neurocognitive health of older adults experiencing homelessness in Oakland, California
Effects of combined cognitive and physical intervention on enhancing cognition in older adults with and without mild cognitive impairment: A systematic review and meta-analysis
Light intensity physical activity is beneficially associated with brain volume in older adults with high cardiovascular risk
Case Report: Rapid Progression of Cognitive Dysfunction as an Initial Feature of Systemic Lupus Erythematosus With Leukoencephalopathy: A Case Report and Literature Review
Identification of a Novel Nonsense Mutation in PLA2G6 and Prenatal Diagnosis in a Chinese Family With Infantile Neuroaxonal Dystrophy
Post-stroke apathy: A case series investigation of neuropsychological and lesion characteristics
Cardiovascular events in patients with chronic myeloid leukaemia treated with tyrosine kinase inhibitors in Taiwan: a nationwide population-based study
Adherence to MIND Diet, Genetic Susceptibility, and Incident Dementia in Three US Cohorts
Lifestyle modification and cognitive function among individuals with resistant hypertension: cognitive outcomes from the TRIUMPH trial
Cohort study evaluation of New Chinese Diabetes Risk Score: a new non-invasive indicator for predicting type 2 diabetes mellitus
Effects of a multicomponent day-care program on cerebral blood flow in patients with mild cognitive impairment
The hands I've been dealt
The Relationship Between Light Exposure before Bedtime and Daytime Sleepiness Among People Living With Cognitive Impairment
Short- and long-term prognostic factors associated with functional recovery in elderly patients with hip fracture: A systematic review
Association of Posttraumatic Stress Disorder with Accelerated Cognitive Decline in Middle-aged Women

Development and Validation of a Predictive Nomogram for Possible REM Sleep Behavior Disorders
Associations between Handgrip Strength and Dementia Risk, Cognition, and Neuroimaging Outcomes in the UK Biobank Cohort Study
Social Isolation and Incident Dementia in the Oldest-Old,ÄîA Competing Risk Analysis
Association Between Social Network and Cognitive Function: A Cross-Sectional Assessment From the Cardiovascular and Metabolic Diseases Etiology Research Center Cohort (2013,Äì2018)
Hearing and Dementia: Is There a Connection?
Postcovid Syndrome ,Äì The New Reality
Best Practice Recommendations for Geriatric Dysphagia Management with 5 Ws and 1H
Chinese expert consensus on the diagnosis and treatment of chronic heart failure in elderly patients (2021)
Functional Improvement Trajectories After Surgery (FIT After Surgery) study: protocol for a multicentre prospective cohort study to evaluate significant new disability after major surgery in older adults
Vitamin D Status and Parkinson,Äôs Disease
Prediction of disability-free survival in healthy older people
Mediation Analyses of the Role of Apathy on Motoric Cognitive Outcomes
Circulating Levels of Apelin, GDF-15 and Sarcopenia: Lack of Association in the MAPT Study
Neuroprotection in aortic arch surgery: untold flaws and future directions
Neurocognitive Profile of the Post-COVID Condition in Adults in Catalonia,ÄîA Mixed Method Prospective Cohort and Nested Case,ÄìControl Study: Study Protocol
The Effectiveness of Multi-Component Interventions on the Positive and Negative Aspects of Well-Being among Informal Caregivers of People with Dementia: A Systematic Review and Meta-Analysis
Physical Activity, Body Mass Index, and Blood Progranulin in Older Adults: Cross-Sectional Associations in the MAPT Study
The end of aspirin for dementia prevention in diabetes?
The challenge of cardiovascular prevention in the elderly: importance of randomized clinical trials
Effect of a multi-domain lifestyle intervention on cardiovascular risk in older people: the FINGER trial
The Multicomponent Day-Care Program Prevents Volume Reduction in a Memory-Related Brain Area in Patients with Mild Cognitive Impairment
Is it possible to prevent cognitive decline among middle-aged and older hypertensive individuals?
Feasibility and Acceptability of a Group Music Intervention in Memory Care Communities
Cerebrovascular response to exercise interacts with individual genotype and amyloid-beta deposition to influence response inhibition with aging
Effectiveness of autonomous home hazard reduction on fear of falling in community-dwelling older women
Risk factors, neuroimaging correlates and prognosis of the motoric cognitive risk syndrome: A population-based comparison with mild cognitive impairment
Orthostatic intolerance: a frailty marker for older adults
Transition from mild cognitive impairment to normal cognition: Determining the predictors of reversion with multi-state Markov models
Effects of Intermittent Hypoxia-Hyperoxia Exposure Prior to Aerobic Cycling Exercise on Physical and Cognitive Performance in Geriatric Patients,ÄîA Randomized Controlled Trial
Clinical effect of ultrasound-guided nerve block and dexmedetomidine anesthesia on lower extremity operative fracture reduction
Higher Serum Soluble TREM2 as a Potential Indicative Biomarker for Cognitive Impairment in Inadequately Controlled Type 2 Diabetes Without Obesity: The DOR-KyotoJ-1
Mortality, Morbidity and Health-Related Outcomes in Informal Caregivers Compared to Non-Caregivers: A Systematic Review
Effects of Two Short-Term Aerobic Exercises on Cognitive Function in Healthy Older Adults during COVID-19 Confinement in Japan: A Pilot Randomized Controlled Trial
Clinical trials in older people
Prognostic Factors for Cervical Spinal Cord Injury without Major Bone Injury in Elderly Patients
Handgrip Strength and Cognitive Function among Elderly Koreans: Insights from the Korean Longitudinal Study of Ageing
Associations of cognitive performance with cardiovascular magnetic resonance phenotypes in the UK Biobank
The Unified Batten Disease Rating Scale (UBDRS): Validation and reliability in an independent CLN3 disease sample
Sarcopenic obesity is associated with cognitive impairment in community-dwelling older adults: The Bunkyo Health Study
Sunbeam Program Reduces Rate of Falls in Long-Term Care Residents With Mild to Moderate Cognitive Impairment or Dementia: Subgroup Analysis of a Cluster Randomized Controlled Trial
Association between depression and motoric cognitive risk syndrome among community-dwelling older adults in China: A 4-year prospective cohort study
Estimating Parkinsonism Severity in Natural Gait Videos of Older Adults With Dementia
Creativity, leisure activities, social engagement and cognitive impairment: the AGES-Reykjav√≠k study
Influence of Cardiovascular Risk Burden on Motor Function Among Older Adults: Mediating Role of Cardiovascular Diseases Accumulation and Cognitive Decline
Management of acute coronary syndromes in older adults
Balance on the Brain: A randomised controlled trial evaluating the effect of a multimodal exercise programme on physical performance, falls, quality of life and cognition for people with mild cognitive impairment-study protocol
Editorial: Automations in Long-Term Neurorehabilitation
Modifiable psychosocial risk factors and delayed onset of dementia in older populations: analysis of two prospective US cohorts

Application of persuasive systems design principles to design a self-management application user interface for Hispanic informal dementia caregivers: User preferences and perceptions
Prefrontal hyperactivation during dual-task walking related to apathy symptoms in older individuals
Simple screening models for cognitive impairment in community settings: The IRIDE Cohort Study
Longitudinal Relationship of Leisure Activity Engagement With Cognitive Performance Among Non-Demented, Community-Dwelling Older Adults
Clinical characteristics of older adults with hypertension and unrecognized cognitive impairment
Use of a nutrition-focused quality improvement program for community-living older adults at malnutrition risk is associated with better nutritional outcomes
APP and DYRK1A regulate axonal and synaptic vesicle protein networks and mediate Alzheimer,Äôs pathology in trisomy 21 neurons
Predicting the long-term cognitive trajectories using machine learning approaches: A Chinese nationwide longitudinal database
Metabolic phenotypes explain the relationship between dysglycaemia and frailty in older people with type 2 diabetes
The prospective association between periodontal disease and brain imaging outcomes: The Atherosclerosis Risk in Communities study
Associations among locus coeruleus catecholamines, tau pathology, and memory in aging
Independent, Differential Effects of Delirium on Disability and Mortality Risk After Hip Fracture
Adaptation and Pilot Study of a Behavioral Intervention Targeting Morning Activation Deficits in Dementia Caregivers: Scheduling Activity and Monitoring Mornings (SAMM)
Reported organic food consumption and metabolic syndrome in older adults: cross-sectional and longitudinal analyses
The serum metabolome mediates the concert of diet, exercise, and neurogenesis, determining the risk for cognitive decline and dementia
Is Dementia-Specific Caregiving Compared With Non-Dementia Caregiving Associated With Physical Difficulty Among Caregivers for Community-Dwelling Adults?
Risk factors for second hip fracture in elderly patients: an age, sex, and fracture type matched case,Äìcontrol study
Protocol for SYNchronising Exercises, Remedies in GaIt and Cognition at Home (SYNERGIC@Home): feasibility of a home-based double-blind randomised controlled trial to improve gait and cognition in individuals at risk for dementia
Geriatric assessment predicts nonfatal toxicities and survival for intensively treated older adults with AML
Respiratory function tests in amyotrophic lateral sclerosis: The role of maximal voluntary ventilation
Respiratory Muscle Training Can Improve Cognition, Lung Function, and Diaphragmatic Thickness Fraction in Male and Non-Obese Patients with Chronic Obstructive Pulmonary Disease: A Prospective Study
Long-term physical activity participation trajectories were associated with subsequent cognitive decline, risk of dementia and all-cause mortality among adults aged ,â•50 years: a population-based cohort study
Association between urinary N-acetyl-Œ≤glucosaminidase activity-urinary creatinine concentration ratio and risk of disability and all-cause mortality
Depression, anxiety, stress, and suicidal behavior among Bangladeshi undergraduate rehabilitation students: An observational study amidst the COVID-19 pandemic
Impact of Biological and Lifestyle Factors on Cognitive Aging and Work Ability in the Dortmund Vital Study: Protocol of an Interdisciplinary, Cross-sectional, and Longitudinal Study
Digital Health in Primordial and Primary Stroke Prevention: A Systematic Review
Association between Oral Frailty and Physical Frailty among Rural Middle,ÄêOld Community,ÄêDwelling People with Cognitive Decline in Taiwan: A Cross,ÄêSectional Study
Predictors of Referral to Cardiac Rehabilitation in Patients following Hospitalisation with Heart Failure: A Multivariate Regression Analysis
Multiple-region grey matter atrophy as a predictor for the development of dementia in a community: the Hisayama Study
Evaluation of a virtual coaching system eHealth intervention: A mixed methods observational cohort study in the Netherlands
Can exercise training teach us how to treat Alzheimer's disease?
Associations of Hearing Loss and Dual Sensory Loss with Mortality: A Systematic Review, Meta-analysis, and Meta-regression of 26 Observational Studies with 1213756 Participants
The association of motoric cognitive risk with incident dementia and neuroimaging characteristics: The Atherosclerosis Risk in Communities Study
Exposure to urban green space may both promote and harm mental health in socially vulnerable neighborhoods: A neighborhood-scale analysis in New York City
Increase in anticholinergic burden from 1990 to 2015: Age-period-cohort analysis in UK biobank
Limitations in activities of daily living increase the risk of stroke in older Chinese adults: A population-based longitudinal study
Investigation of the relationships between sleep behaviors and risk of healthspan termination: a prospective cohort study based on 323,373 UK-Biobank participants
Association of Glycative Stress With Motor and Muscle Function
Engaging Stakeholders in Realist Programme Theory Building: Insights from the Prospective Phase of a Primary Care Dementia Support Study
Psychological Resilience and Cognitive Function Among Older Military Veterans
Role of a Urinary Biomarker in the Common Mechanism of Physical Performance and Cognitive Function
Using Health Check Data to Understand Risks for Dementia and Cognitive Impairment Among Torres Strait Islander and Aboriginal Peoples in Northern Queensland,ÄîA Data Linkage Study
Case Report: Neurodegenerative Diseases After Severe Acute Respiratory Syndrome Coronavirus 2 Infection, a Report of Three Cases: Creutzfeldt,ÄìJakob Disease, Rapidly Progressive Alzheimer's Disease, and Frontotemporal Dementia
Bony hypertrophy of the forearm in bareback rodeo athletes
Subjective cognitive decline and total energy intake: Talk too much?
Goal management training and psychoeducation/mindfulness for treatment of executive dysfunction in Parkinson's disease: A feasibility pilot trial
Survival of Frail Elderly with Delirium

Risk Factors of Long-Term Care Insurance Certification in Japan: A Scoping Review
Clinical Disease Severity Mediates the Relationship between Stride Length and Speed and the Risk of Falling in Parkinson‚Äôs Disease
Insomnia symptom subtypes and manifestations of prodromal neurodegeneration: a population-based study in the Canadian Longitudinal Study on Aging
Trials and Treatments for Vascular Brain Health: Risk Factor Modification and Cognitive Outcomes
Clinical implications of assessing frailty in elderly patients treated with permanent cardiac pacing
Long-term ozone exposure and cognitive impairment among Chinese older adults: A cohort study
Cardiometabolic risk factors associated with brain age and accelerate brain ageing
Alzheimer Disease: Recent Updates on Apolipoprotein E and Gut Microbiome Mediation of Oxidative Stress, and Prospective Interventional Agents
Association between community-level social capital and frailty onset among older adults: A multilevel longitudinal study from the Japan Gerontological Evaluation Study (JAGES)
Association of dietary sulfur amino acid intake with mortality from diabetes and other causes
Digital Biomarkers of Cognitive Frailty: The Value of Detailed Gait Assessment beyond Gait Speed
Prevalence of usage of hearing aids and its association with cognitive impairment in Japanese community-dwelling elders with hearing loss
Model Testing of the Factors That Influence Performance of Function Focused Care and Function Among Assisted Living Residents
Aerobic Exercise Training-Induced Changes on DNA Methylation in Mild Cognitively Impaired Elderly African Americans: Gene, Exercise, and Memory Study - GEMS-I
Characteristics of Dysregulated Proinflammatory Cytokines and Cognitive Dysfunction in Late-Life Depression and Amnestic Mild Cognitive Impairment
Characterising activity and diet compositions for dementia prevention: Protocol for the ACTIVate prospective longitudinal cohort study
Cognitive Training and Aerobic Exercise as Intervention Techniques for Mild Cognitive Impairment: A Research Protocol
Alzheimer's Disease Prediction using Machine Learning and Transfer Learning Models
Relationship between Sleep Disorders and Neuropsychiatric Symptoms in Parkinson's Disease: A Narrative Review
The impact of hypertension on mortality and the risk of developing geriatric syndromes
Life satisfaction across the entire trajectory of Alzheimer's disease: A mediation analysis
Cardiorespiratory fitness and cognition in persons at risk for Alzheimer's disease
Physical activity is associated with increased resting-state functional connectivity in networks predictive of cognitive decline in clinically unimpaired older adults
The Coaching for Cognition in Alzheimer's (COCOA) trial: Study design
Combination of gait speed and grip strength to predict cognitive decline and dementia
Associations of the Lifestyle for Brain Health index with longitudinal cognition and brain amyloid beta in clinically unimpaired older adults: Findings from the Wisconsin Registry for Alzheimer's Prevention
Socioeconomic Inequalities in Cognitive Functioning Only to a Small Extent Attributable to Modifiable Health and Lifestyle Factors in Individuals Without Dementia
EFFECT OF MICROSCOPIC KEYHOLE SURGERY ON NEUROLOGICAL FUNCTION AND PROGNOSIS OF PATIENTS WITH INTRACRANIAL SMALL MENINGIOMA
Pathways explaining racial/ethnic disparities in incident all-cause and Alzheimer's disease dementia among older US men and women
Utility of Actimetry to Detect Apathy in Old-Age Depression: a Pilot Study
The Effect of the Continuous Care on the Self-Care Behavior in Hematopoietic Stem Cell Transplantation (HSCT) Recipients: A Semi-Experimental Study
Models to predict prognosis in older patients with heart failure complicated by pre-frailty and frailty: a pilot prospective cohort study
The Effect of Therapeutic Exercise on Fall Prevention and Balance in Older Adult Patients with Dementia: A Narrative Review
Impact of pharmacist intervention in patients with alzheimer‚Äôs disease.
Population Attributable Fractions for Modifiable Risk Factors of Incident Dementia in Cognitively Normal and Mild Cognitively Impaired Older Adults: Data from Two Cohort Studies
Evaluating the Use of Digital Biomarkers to Test Treatment Effects on Cognition and Movement in Patients with Lewy Body Dementia
Facets of Personality and Risk of Cognitive Impairment: Longitudinal Findings in a Rural Community from Sardinia
Are Wearable Sensors Useful to Assess the Psychophysical Fatigue Due to Physical Activity in Elderly People with Mild Cognitive Impairment? A Preliminary Study
Daily Physical Activity Patterns as a Window on Cognitive Diagnosis in the Baltimore Longitudinal Study of Aging (BLSA)
Influence of selected factors on the prevalence of cognitive disorders and depression among patients over 60 years of age
Internal fixation or hip replacement for undisplaced femoral neck fractures? Pre-fracture health differences reflect survival and functional outcome
Participation in Social Group Activities and Risk of Dementia: A Systematic Review
Late-life depression and the family physician
Cost-Effectiveness of a Multidisciplinary Lifestyle-Enhancing Treatment for Inpatients with Severe Mental Illness: The MULTI Study v
Causal structure discovery identifies risk factors and early brain markers related to evolution of white matter hyperintensities
Endurance and gait speed relationships with mild cognitive impairment and dementia
A Pilot and¬†Feasibility Study of¬†Virtual Reality as¬†Gamified Monitoring Tool for¬†Neurorehabilitation
Alzheimer's Disease Detection Using Comprehensive Analysis of Timed Up and Go Test via Kinect V.2 Camera and Machine Learning
Associations Between Behavioral Flexibility and Health Behavior in Cardiac Patients in the Do CHANGE Trials
Basal Cortisol Levels Are Increased in Patients with Mild Cognitive Impairment: Role of Insomnia and Short Sleep Duration

The Current Landscape of Prevention Trials in Dementia
Healthy lifestyle and life expectancy with and without Alzheimer's dementia: Population based cohort study
Changes in Callosal Angle and Evans,Äô Index After Placing a Lumboperitoneal Shunt in Patients with Idiopathic-Normal-Pressure Hydrocephalus
Late-Life Physical Activities Moderate the Relationship of Amyloid-Œ≤ Pathology with Neurodegeneration in Individuals Without Dementia
A Systematic Review on Serious Games in Attention Rehabilitation and Their Effects
Role of Inflammatory Cytokines in the Conversion of Mild Cognitive Impairment to Dementia: A Prospective Study
The Association between Patterns of Social Engagement and Conversion from Mild Cognitive Impairment to Dementia: Evidence from the Health and Retirement Study
The Bite Is Worse Than the Bark: Associations of Personality and Depressive Symptoms With Memory Discrepancy
Age of Symptom Onset and Longitudinal Course of Sporadic Alzheimer's Disease, Frontotemporal Dementia, and Vascular Dementia: A Systematic Review and Meta-Analysis
A multidomain decision support tool to prevent falls in older people: the FinCH cluster RCT
Body Mass Index and Mild Cognitive Impairment among Middle-Aged and Older Adults from Low- and Middle-Income Countries
Association of serum 25-hydroxyvitamin D concentrations with risk of dementia among individuals with type 2 diabetes: A cohort study in the UK Biobank
Effects of functional task exercise on everyday problem-solving ability and functional status in older adults with mild cognitive impairment - A randomised controlled trial
Multimodal Preventive Trial for Alzheimer,Äôs Disease: MIND-ADmini Pilot Trial Study Design and Progress
Association between Late-Onset M√©ni√®re,Äôs Disease and the Risk of Incident All-Cause Dementia
Serum Vitamin D as a Biomarker in Autoimmune, Psychiatric and Neurodegenerative Diseases
Early Detection of Pneumonia with the Help of Dementia in Geriatric Hip Fracture Patients
Nutrition senolytics-illusion or reality for cognitive ageing?
Clinical Impact of Measures for Frailty Severity in Poor-Risk Patients Undergoing Revascularization for Chronic LimbThreatening Ischemia
Impact of the iCODEX tool in routine clinical practice in Spain
Blood pressure levels and risks of dementia: A nationwide study of 4.5 million people
Household-level lifestyle interventions for the prevention of cognitive decline; A Systematic review
Cerebrovascular Reactivity (CVR) in Aging, Cognitive Impairment, and Dementia
Pre-Operative Predictors for Discharge to Post-Acute Care Facilities After Total Knee Arthroplasty
Frailty but not sarcopenia nor malnutrition increases the risk of developing COVID-19 in older community-dwelling adults
Annual reports on hypertension research 2020
The Effect of Multidomain Interventions on Global Cognition, Symptoms of Depression and Apathy ,Äî A Pooled Analysis of Two Randomized Controlled Trials
DCE-MRI of Brain Fluid Barriers: In Vivo Water Cycling at the Human Choroid Plexus
Slowing: A Vascular Geriatric Syndrome?
Dietary sugar intake and risk of Alzheimer's disease in older women
Observational assessment of communication to empower patients with dementia to make legally effective decisions,Äìrevalidation of CODEMamb : Full-Length Research Report
Alzheimer's disease research progress in Australia: The Alzheimer's Association International Conference Satellite Symposium in Sydney
Role of plant-based diet in late-life cognitive decline: results from the Salus in Apulia Study
Caregiver Burden in Late-Stage Parkinsonism and Its Associations
Dietary antioxidants associated with slower progression of parkinsonian signs in older adults
THE ROLE OF PHYSICAL ACTIVITY IN MANAGING DEPRESSION IN ELDERLY POPULATIONS.
Physical Activity and Dementia in Africa: The Missing Evidence and the Path Forward.
Physical Activity, Aging and Brain Health.
Systematic review and meta-analysis on population attributable fraction for physical inactivity to dementia
Effects of Combined Interventions with Aerobic Physical Exercise and Cognitive Training on Cognitive Function in Stroke Patients: A Systematic Review
Calanus Oil Supplementation Does Not Further Improve Short-Term Memory or Brain-Derived Neurotrophic Factor in Older Women Who Underwent Exercise Training
Multi-component exercise training improves the physical and cognitive function of the elderly with mild cognitive impairment: a six-month randomized controlled trial
Effects of low-intensity home-based exercise on cognition in older persons with mild cognitive impairment: a direct comparison of aerobic versus resistance exercises using a randomized controlled trial design
Effects of a Home-Based Physical Training and Activity Promotion Program in Community-Dwelling Older Persons with Cognitive Impairment after Discharge from Rehabilitation: A Randomized Controlled Trial
The Role of Smoking, Obesity, and Physical Inactivity in Cognitive Performance and Decline: A Multicohort Study
5 Years of Exercise Intervention Did Not Benefit Cognition Compared to the Physical Activity Guidelines in Older Adults, but Higher Cardiorespiratory Fitness Did. A Generation 100 Substudy
Effects of home-based computerized cognitive training and tai chi exercise on cognitive functions in older adults with mild cognitive impairment
Cerebral small vessel disease, cardiovascular risk factors, and future walking speed in old age: a population-based cohort study
Physical Inactivity Links Depressive Symptoms and Cognitive Functioning Among Individuals With Parkinson's Disease

Investigating the effects of a randomized, double-blinded aerobic, resistance, and cognitive training clinical trial on neurocognitive function in older adults with cardiovascular risk factors: the ACTIONcardioRisk protocol
Physical activity levels and brain structure in middle-aged and older adults: a bidirectional longitudinal population-based study
Effects of different dual task training on dual task walking and responding brain activation in older adults with mild cognitive impairment
The role of physical exercise in modulating peripheral inflammatory and neurotrophic biomarkers in older adults: A systematic review and meta-analysis
Exploring the associations between physical activity level, cognitive performance, and response to computerized cognitive training among chronic stroke patients
The influence of a 16-week exercise program, APOE status, and age on executive function task performance: A randomized trial
Midlife aerobic exercise and brain structural integrity: Associations with age and cardiorespiratory fitness
Bi-directional association between outdoor or social activities and cognitive function: do the PM2.5 exposure catalyze the detrimental inactivity-poor cognition cycle?
Cognitive Function is a Predictor of the Daily Step Count in Patients With Subacute Stroke With Independent Walking Ability: A Prospective Cohort Study
Symposium Resistance exercise to reduce risk of falls in people with Alzheimer's disease: a randomised clinical trial
The Benefits of Physical Activity in Individuals with Cardiovascular Risk Factors: A Longitudinal Investigation Using fNIRS and Dual-Task Walking
Association between walking energy utilisation and longitudinal cognitive performance in older adults
The effects of Baduanjin exercise on the subjective memory complaint of older adults A randomized controlled trial
The Benefits of High-Intensity Interval Training on Cognition and Blood Pressure in Older Adults With Hypertension and Subjective Cognitive Decline: Results From the Heart & Mind Study
Comparative effects of different physical exercises on cognitive function and intervention adherence in older adults with cognitive impairment: A systematic review and network meta-analysis
Shared Risk Factors between Dementia and Atherosclerotic Cardiovascular Disease
Predictive Validity of Different Walking Measures to Identify the Incident Long-Term Care Needs in Older Adults
Combined Training Improves Executive Functions Without Changing Brain-Derived Neurotrophic Factor Levels of Middle-Aged and Older Adults with Type 2 Diabetes
The benefits of physical exercise on older adults ' cognitive function: A cohort study exploring potential mechanisms
Baseline Global Cognitive Function Affects Cognitive and Functional Outcomes of Combined Physical and Cognitive Training Among Older Adults With Cognitive Decline
Gamified Dual-Task Training for Individuals with Parkinson Disease: An Exploratory Study on Feasibility, Safety, and Efficacy
Mediating role of frailty/pre-frailty on the association between exercise participation and traffic crashes among Japanese community-dwelling older drivers
Computerized Cognitive Training (CCT) versus Yoga Impact on 12 Month Post Intervention Cognitive Outcome in Individuals with Mild Cognitive Impairment
The role of structured exercise interventions on cognitive function in older individuals with stable Chronic Obstructive Pulmonary Disease: A scoping review
Interaction between physical exercise and APOE gene polymorphism on cognitive function in older people
Physical activity modifies cognitive impairment-associated mortality risks among chronic kidney disease
Fatness but Not Fitness Linked to BrainAge: Longitudinal Changes in Brain Aging during an Exercise Intervention
Effects of square-stepping exercise on executive function in individuals with Parkinson's disease: A randomized controlled pilot study
Intervention of computer-assisted cognitive training combined with occupational therapy in people with mild cognitive impairment: a randomized controlled trial
Staying active, staying sharp: the relationship between physical activity and health-related quality of life for people living with cognitive impairment
The Complexity of Blood Pressure Fluctuation Mediated the Effects of Hypertension on Walking Speed in Older Adults
Effects of a 16-week multimodal exercise program on activities of daily living in institutionalized individuals with dementia A multicenter randomized controlled trial
Association between walking and square dancing-oriented leisure-time physical activity and cognitive function among middle-aged and elderly people in Southwest China
Eight-week exercise program improved the quality of life of Alzheimer's patients through functional, cognitive, and biochemical parameters
Impact of type, intensity, frequency, duration and volume of physical activity on dementia and mild cognitive impairment in older adults: protocol for a systematic review and meta-analysis
The impact of physical activities on cognitive function among older adult populations: evidence from China
Effects and influencing factors of Internet-based training on primary prevention of dementia among primary health care workers
Effects of Yoga and Aerobic Exercise on Verbal Fluency in Physically Inactive Older Adults: Randomized Controlled Trial (FitForAge)
Effects of Aerobic Exercise on Brain Age and Health in Middle-Aged and Older Adults: A Single-Arm Pilot Clinical Trial
Relationship between Walking and Driving, and Cognitive Functioning Common to Both Modes of Mobility, in Healthy Older Adults
Dual-Task Zumba Gold for Improving the Cognition of People With Mild Cognitive Impairment: A Pilot Randomized Controlled Trial
Walking Reduced the Risk of Parkinson's disease (PD) Dementia: A Retrospective Longitudinal Follow-up Study
Foreign Language Learning as Cognitive Training to Prevent Old Age Disorders? Protocol of a Randomized Controlled Trial of Language Training vs. Musical Training and Social Interaction in Elderly With Subjective Cognitive Decline
Effect of 5 Years of Exercise Intervention at Different Intensities on Brain Structure in Older Adults from the General Population: A Generation 100 Substudy
Physical activity in an air-polluted environment: behavioral, psychological and neuroimaging protocol for a prospective cohort study (Healthy Aging in Industrial Environment study - Program 4)
Physical exercise and goal attainment after shunt surgery in idiopathic normal pressure hydrocephalus: a randomised clinical trial
Perspectives of Barry A. Franklin, PhD: A World-Renowned Professor, Author, and Speaker
Effects of aerobic, strength, and combined exercise on cognitive functions in older adults: a systematic review

Comparative effectiveness of open and closed skill exercises on cognitive function in young adults: a fNIRS study
Can Task Specificity Impact tDCS-Linked to Dual Task Training Gains in Parkinson's Disease? A Protocol for a Randomized Controlled Trial
Lack of physical activity, neuropsychiatric symptoms and the risk of incident mild cognitive impairment in older community-dwelling individuals A prospective cohort study
Association of physical activity with socio-economic status and chronic disease in older adults in China: cross-sectional findings from the survey of CLASS 2020 after the outbreak of COVID-19
Greater hepatic lipid saturation is associated with impaired glycaemic regulation in men with metabolic dysfunction-associated steatotic liver disease but is not altered by 6 weeks of exercise training
Effects of nurse-led cognitive-motor dual-task training based on mobile health technology on the older adults with cognitive frailty: A quasi-experimental study
Improvement of nutritional status enhances cognitive and physical functions in older adults with orthostatic hypotension
Twenty key insights into neighborhood walking experiences of people living with dementia in Metro Vancouver: a quantitative analysis of qualitative data
How older adults negotiate constraints to leisure-time physical activities following a heart attack
Modifiable Risk Factors Associated With Cognitive Decline in Late Life Depression: Findings From the Canadian Longitudinal Study on Aging
Effect of Regular Aerobic Exercise on Cognitive Function, Depression Level and Regulative Role of Neurotrophic Factor: A Prospective Cohort Study in the Young and the Middle-Aged Sample
Daily function outcomes in adults with mild cognitive impairment due to Alzheimer's disease after two years of processing speed training versus a control training protocol
Multimodal Image Analysis of Apparent Brain Age Identifies Physical Fitness as Predictor of Brain Maintenance
Compensatory and Lifestyle-Based Brain Health Program for Subjective Cognitive Decline: Self-Implementation versus Coaching
EFFECTS OF UPPER LIMB VIBRATORY STIMULATION TRAINING ON MOTOR SYMPTOMS IN PARKINSON'S DISEASE: AN OBSERVATIONAL STUDY
Impact of Behavioral Assessment and Re-Test as Functional Trainings That Modify Survival, Anxiety and Functional Profile (Physical Endurance and Motor Learning) of Old Male and Female 3xTg-AD Mice and NTg Mice with Normal Aging
Comparison of risk factors for dementia among rural and urban elderly adults-data from two cohort studies in India
Next Steps for Clinical Xenotransplantation in the United States
The effects of sedentary behaviour (sitting time) on health. What prevention?
Associations between resting heart rate and cognitive decline in Chinese oldest old individuals: a longitudinal cohort study
Study Protocol of a Comprehensive Activity Promotion
Enhancing Neurocognitive Health via Activity, Nutrition and Cognitive Exercise (ENHANCE): A Randomized Controlled Trial
Atrial Fibrillation Follow-up Investigation to Recover Memory and Learning Trial (AFFIRMING) : Rationale and Design of a Multi-center, Double-blind, Randomized Controlled Trial
Relating Global Cognition With Upper-Extremity Motor Skill Retention in Individuals With Mild-to-Moderate Parkinson's Disease
Transcranial direct current stimulation as an adjunct to cognitive training for older adults with mild cognitive impairment: A randomized controlled trial
Non-motor impairments affect walking kinematics in Parkinson disease patients: A cross-sectional study
Association between denture use, chewing ability, and all-cause mortality in middle-aged and older adults who exercised regularly in Korea
Cognitive Function Changes in Older People. Results of Second Wave of Cognition of Older People, Education, Recreational Activities, NutritIon, Comorbidities, fUnctional Capacity Studies (COPERNICUS)
The Association Between Injurious Falls and Older Adults' Cognitive Function: The Role of Depressive Mood and Physical Performance
Influence of cognitive and emotional factors on motor dual-task performance in nursing home residents. The mediating and moderating roles of cognition, concerns about falling, well-being, and depressive symptoms. A cross-sectional observational study
A framework for rehabilitation for older adults living with dementia
Evolving dementia care: An explorative study on the lived experience of older adults living with dementia in nursing homes using observational and biometric sensor data
The Effects of Multidisciplinary Intensive Rehabilitation on Cognitive and Executive Functions in Parkinson's Disease: A Clinical Database Analysis
Cognitive and social activities and long-term dementia risk: the prospective UK Million Women Study
Grip Strength and Sarcopenia Predict 2-Year Cognitive Impairment in Community-Dwelling Older Adults
Association of Light-Intensity Physical Activity With Mortality in the Older Population: A Nationwide Cohort Study
Caregivers facing violence in long-term care setting: A cross analysis of incident reports and caregivers speech
Can Physical Activity Reduce the Risk of Cognitive Decline in Apolipoprotein e4 Carriers? A Systematic Review
Protocol of the MOVI-ageing randomized controlled trial: a home-based e-Health intervention of cognitively demanding exercise for the improvement of cardiorespiratory fitness and cognitive function in older individuals
Effect of an 8-Week Mindfulness Meditation Training Program on Psychological Distress in Caregivers and on Behavioral and Psychological Symptoms in People with Alzheimer's Disease: A Randomized Controlled Trial
Comparing Occupational Adaptation-Based and Traditional Training Programs for Dementia Care Teams: An Embedded Mixed-Methods Study
Association of Stair Use With Risk of Major Chronic Diseases
Age-Related Differences in Healthy Adults Walking Patterns Under a Cognitive Task With Deep Neural Networks
A narrative review of risk factors and interventions for cancer-related cognitive impairment
Sleep disturbance predicts worse cognitive performance in subsequent years: A longitudinal population-based cohort study
Professionals' Self-Reported Difficulties towards Integrating Dual Task Training in Care for People with Parkinson's Disease

Activity engagement and cognitive function among chinese older adults: moderating roles of gender and age
Cognitive-exercise dual-task intervention ameliorates cognitive decline in natural aging rats through reducing oxidative stress and enhancing synaptic plasticity
Cardiometabolic and Vascular Disease Factors and Mild Cognitive Impairment and Dementia
Hobby Engagement and Risk of Disabling Dementia
Prospective Associations between Physical Activity and Memory in the Canadian Longitudinal Study on Aging: Examining Social Determinants
Cognitive Benefit of a Multidomain Intervention for Older Adults at Risk of Cognitive Decline: A Cluster- Randomized Controlled Trial
Cross-sectional and longitudinal interaction effects of physical activity and APOE-Œµ4 on white matter integrity in older adults: The MAPT study
Can Digital Mobility Assessment Enhance the Clinical Assessment of Disease Severity in Parkinson's Disease?
Exploring life-space in the nursing home. An observational longitudinal study
Impacts of stroke and cognitive impairment on activities of daily living in the Taiwan longitudinal study on aging
Well-designed manufacturing work improves some cognitive abilities in individuals with cognitive impairments
Occupational adaptation as a social process for dementia care teams
Cognitive frailty in older adults users of primary health care
Diabetes and hypertension are related to amyloid-beta burden in the population-based Rotterdam Study
The health status: the ignored risk factor in dementia incidence. NEDICES cohort
Multimorbidity, lifestyle, and cognitive function: A cross-cultural study on the role of diabetes, cardiovascular disease, cancer, and chronic respiratory diseases
Behavioral Rehabilitation of Swallowing Disorders in Patients with Neurocognitive Disorders
Association of physical performance with cognitive impairment in middle-aged to older haemodialysis patients: a multicentre cross-sectional observational study
Progression of Gait Changes in Older Adults With Mild Cognitive Impairment: A Systematic Review
New Disability in a Cohort Study of Older Men-The Manitoba Follow-Up Study
Predictors of cognitive impairment in patients undergoing ileostomy for colorectal cancer: a retrospective analysis
Association of lifelong cognitive reserve with dementia and mild cognitive impairment among older adults with limited formal education: a population-based cohort study
Internal consistency and reliability of the lifetime and modified current cognitive activity questionnaires and their association with cognitive performance: a six-year follow up of the Brain in Motion study
Physical Activity and Cognitive Decline Among Older Adults A Systematic Review and Meta-Analysis
The HOMESIDE Music Intervention: A Training Protocol for Family Carers of People Living with Dementia
Cognitive Screening via Comprehensive Geriatric Assessment of Older Patients for the Risk Factors of Hospital Revisit/Readmission after Emergency Department Visit at 3-Month Follow-Up
The potential dangers of not understanding COVID-19 public health restrictions in dementia: "It's a groundhog day - every single day she does not understand why she can't go out for a walk"
A Narrative Review of Physical Performance Changes in Dementia: Differences between Community and Nursing Home Setting
Aerobic exercise and brain structure among military service members and Veterans with varying histories of mild traumatic brain injury: A LIMBIC-CENC exploratory investigation
Mediation of modifiable risk factors in two multidomain dementia prevention trials
Temporal changes in personal activity intelligence and
Association between anemia, physical performance and cognitive function in Iranian elderly people: evidence from Bushehr Elderly Health (BEH) program
Lifestyle Influence on Mild Cognitive Impairment Progression: A Decision Tree Prediction Model Study
Hearing and cognitive scores measured with the Montreal Cognitive Assessment Scale in The HUNT Study, Norway
Exposure to ambient air pollution and cognitive function: an analysis of the English Longitudinal Study of Ageing cohort
How are hearing loss and physical activity related? Analysis from the English longitudinal study of ageing
Ultra-high-field 7T MRI reveals changes in human medial temporal lobe volume in female adults during menstrual cycle
Low Plasma Ergothioneine Predicts Cognitive and Functional Decline in an Elderly Cohort Attending Memory Clinics
Determinants of clinical response in possible normal pressure hydrocephalus
MemAID: Memory advancement with intranasal insulin vs. placebo in type 2 diabetes and control participants: a randomized clinical trial
The effect of first-time 4-wheeled walker use on the gait of younger and older adults
Tourism experiences reduce the risk of cognitive impairment in the Chinese older adult: a prospective cohort study
Central obesity and elevated blood pressure in middle life are associated with physical and cognitive impairment in later life: A retrospective design with repeated measures
Cross-sectional associations between cortical thickness and physical activity in older adults with spontaneous memory complaints: The MAPT Study
The effects of dementia on the prognosis and mortality of hip fracture surgery: a systematic review and meta-analysis
Exploring Cognitively Loaded Physical Activity Compared With Control to Improve Global Cognitive Function in Older Community-Dwelling Adults With Mild Cognitive Impairment: Systematic Review With Meta-Analysis
Association between F2-Isoprostane Metabolites and Weight Change in Older Women: A Longitudinal Analysis
The Effect of Eight Weeks of High-Intensity Interval Training on Plasma LRP1, AB1-42, and Insulin Resistance in Obese Elderly Diabetic Rats
Dementia Rehabilitation Training for General Practitioners and Practice Nurses: Does It Make a Difference?
Wheelchair dependence in patients with dementia: Focus on kinematic gait analysis using simple wearable accelerometers and gyroscopes

Association between gait features assessed by artificial intelligent system and cognitive function decline in patients with silent cerebrovascular disease: study protocol of a multicenter prospective cohort study (ACCURATE-2)
A Cumulative Muscle Index and Its Parameters for Predicting Future Cognitive Decline: Longitudinal Outcomes of the ASPRA Cohort
Factors Influencing Change in Brain-Predicted Age Difference in a Cohort of Healthy Older Individuals
Relationship Between Fasting Blood Glucose Levels in Middle Age and Cognitive Function in Later Life: The Aichi Workers' Cohort Study
Mild cognitive impairment predicts the onset of Sarcopenia: a longitudinal analysis from the English Longitudinal Study on Ageing
Deltoid muscle strength and autonomic dysfunction as independent risk factors for invasive mechanical ventilation in patients with Guillain-Barre syndrome
Impacts of the National Your Brain Matters Dementia Risk Reduction Campaign in Australia Over 2 Years
Onset of cognitive impairment, diet quality and adherence to dietary guidelines over 12 years: the Personality and Total Health Cohort Study
Cross-sectional and longitudinal association between accelerometer-measured light-intensity physical activity and cognitive function in older adults
Risk factors of cognitive frailty in elderly chronic obstructive pulmonary disease patients and its implication for death: a cohort study
Frailty, Cognitive Impairment, and Incident Major Neurocognitive Disorders: Results of the NuAge Cohort Study
Estimating the effect of physical activity on cognitive function within the UK Biobank cohort
Effects of a Combined Motor Imagery and Action Observation Intervention on Vascular Cognitive Impairment A Randomized Pilot Study
Cognitive Improvement After Multi-Domain Lifestyle Interventions in an APOE Œμ4 Homozygous Carrier with Mild Cognitive Impairment: A Case Report and Literature Review
A Three-Year Longitudinal Follow-Up Study: Does Mild Cognitive Impairment Accelerate Age-Related Changes in Physical Function and Body Composition?
The Effect of Multimodal Non-pharmacological Interventions on Cognitive Function Improvement for People With Dementia: A Systematic Review
Peak Expiratory Flow Predicts Incident Dementia in a Representative Sample of US Older Adults: The National Health and Aging Trends Study (NHATS).
RETRACTED: 6-month consequences of COVID-19 in patients discharged from hospital: a cohort study (Publication with Expression of Concern. See vol. 401, pg. 90, 2023) (Retracted Article)
An Increased Anticholinergic Drug Burden Index Score Negatively Affect Nutritional Status in Older Patients Without Dementia
Depression and Functional Mobility Decline in Long Term Care Home Residents with Dementia: a Prospective Cohort Study
The prodromal phase of hereditary spastic paraplegia type 4: the preSPG4 cohort study
Association between garden work and risk of incident dementia in an older population in China: a national cohort study
Sex-specific longitudinal association between baseline physical activity level and cognitive decline in Chinese over 45 years old: Evidence from the China health and retirement longitudinal study
Incidence and Prevalence of Dementia Over the Life Course of Aging Men: The Manitoba Follow-Up Study
Are Care-Recipient Outcomes Attributable to Improved Caregiver Well-Being? A Cluster-Randomized Controlled Trial of Benefit-Finding Intervention
Effects of global cognitive function on the prevalence of fear of falling in older adults
From basic care to beyond: A Q methodology study into the English communication needs among Thai caregivers of foreign older adults
Predictive power of gait and gait-related cognitive measures in amnestic mild cognitive impairment: a machine learning analysis
Dementia Risk Score for a Population in Southern Europe Calculated Using Competing Risk Models
A non-linear relationship between blood pressure and mild cognitive impairment in elderly individuals: A cohort study based on the Chinese longitudinal healthy longevity survey (CLHLS)
Relation between physical activity and cerebral small vessel disease: A nine-year prospective cohort study
Effects of Mindfulness-Based Tai Chi Chuan on Physical Performance and Cognitive Function among Cognitive Frailty Older Adults: A Six-Month Follow-Up of a Randomized Controlled Trial
Longitudinal Association of Remnant Cholesterol With Cognitive Decline Varies by Lipid-Lowering Drugs: A Population-Based Cohort Study
A Multicomponent Cognitive Intervention May Improve Self-Reported Daily Function of Adults With Subjective Cognitive Decline
Longitudinal observation of radiation-induced cognitive impairment and emotional dysfunction: Based on animal model and clinical cohort
Long-term television viewing patterns and gray matter brain volume in midlife
Domains of Sedentary Behavior and Cognitive Function: The Health, Aging, and Body Composition Study, 1999/2000 to 2006/2007
Effects of reversible cognitive frailty on disability, quality of life, depression, and hospitalization: a prospective cohort study
Longitudinal Associations Between Self-reported Exercise Levels And Alzheimers Related Biomarkers In Autosomal Dominant Alzheimer'S Disease
Pulmonary function is associated with cognitive decline and structural brain differences
Cognitive frailty and 5-year adverse health-related outcomes for the Malaysian elders longitudinal research (MELoR) study
Association of Early-, Middle-, and Late-Life Depression With Incident Dementia in a Danish Cohort
Plasma proteomic characterization of motoric cognitive risk and mild cognitive impairment
Association between sleep duration and disability in activities of daily living among Chinese older adults: a nationwide observational study
Physical activity trajectories and cognitive function: A national cohort study
Respiratory Function is Associated with Cognitive Function Change in Japanese Community-Dwelling Older People: A 1-Year, Longitudinal, Observational Study
Changes in Self-estimated Step-Over Ability Among Older Adults: A 3-Year Follow-up Study
Role of learning experiences of care knowledge and skills on caregiver burden and self-esteem among informal caregivers: an observational cross-sectional study
Joint exposure to outdoor ambient air pollutants and incident chronic kidney disease: A prospective cohort study with 90,032 older adults
Body mass index in early adulthood and dementia in late life: Findings from a pooled cohort
Total cerebral small vessel disease score and all-cause mortality in older adults of Amerindian ancestry: The Atahualpa Project

A Longitudinal Study of the Effect of Memory on the Quality of life of European Adults and Older Adults
Associations of cognitive impairment and longitudinal change in cognitive function with the risk of fatal stroke in middle-aged to older Chinese
Prevalence and incidence of possible vascular dementia among Mexican older adults: Analysis of the Mexican Health and Aging Study
Degenerative adversarial neuroimage nets for brain scan simulations: Application in ageing and dementia
Interventions for reducing anticholinergic medication burden in older adults-a systematic review and meta-analysis
Observational evidence of the association between physical and psychological determinants of aging with cognition in older adults
ApoE Mimetic Peptides to Improve the Vicious Cycle of Malnutrition and Enteric Infections by Targeting the Intestinal and Blood-Brain Barriers
Multi-component cognitive intervention for older adults with mixed cognitive levels: implementation and preliminary effectiveness in real-world settings
Prevalence/potential risk factors for motoric cognitive risk and its relationship to falls in elderly Chinese people: a cross-sectional study
Risk of Dementia According to Surgery Type: A Nationwide Cohort Study
Health-related heterogeneity in brain aging and associations with longitudinal change in cognitive function
Cognitive leisure activity and all-cause mortality in older adults: a 4-year community-based cohort
Mediating role of instrumental activities of daily living ability on cognitive function of older adults living alone A 4-year longitudinal study from the Kasama study
Modifiable Risk Factors in Alzheimer Disease and Related Dementias: A Review
Warfarin Versus Non-Vitamin K Inhibitors Medications and Cognitive Disease Progression in Atrial Fibrillation
Incidence and risk factors associated with progression to mild cognitive impairment among middle-aged and older adults
Arteriolosclerosis in CNS Tissues Outside the Cerebrum and Late-Life Motor Impairment
Rethinking the education potential of older adults to delay the onset of dementia
Mild Cognitive Impairment: the Manchester consensus
Association between healthy lifestyle and memory decline in older adults: 10 year, population based, prospective cohort study
Alzheimer Disease: Standard of Diagnosis, Treatment, Care, and Prevention
Ozone Exposure, Outdoor Physical Activity, and Incident Type 2 Diabetes in the SALSA Cohort of Older Mexican Americans
Does Late-Life Depression Counteract the Beneficial Effect of Physical Activity on Cognitive Decline? Results From the NESDO Study
Association of midlife air pollution exposures and residential road proximity with incident dementia: The Atherosclerosis Risk in Communities (ARIC) study
Number of teeth and functional disability in community-dwelling older adults
A Controlled Pilot Study of the Wish Outcome Obstacle Plan Strategy for Spouses of Persons With Early-Stage Dementia
Exploring Older Adults' Pre-Intervention Motivations, Attitudes, Expectations, and Barriers to Participation in the FINGER-NL Lifestyle Intervention to Maintain Optimal Cognitive Functioning: A Qualitative Interview Study
Integration of Virtual Reality into Transcranial Magnetic Stimulation Improves Cognitive Function in Patients with Parkinson's Disease with Cognitive Impairment: A Proof-of-Concept Study
Efficacy of short-term multidisciplinary intensive rehabilitation in patients with different Parkinson's disease motor subtypes: a prospective pilot study with 3-month follow-up
What's behind the Dashboard? Intervention Mapping of a Mobility Outcomes Monitoring System for Rehabilitation
Development of multi-component counseling program for enhancing resilience among Thai caregivers of older persons with dementia
Determinants of physical activity in community-dwelling older adults: an umbrella review
Accelerated Pace of Aging in Schizophrenia: Five Case-Control Studies
Association between neighborhood availability of physical activity facilities and cognitive performance in older adults
Brain 18F-fluorodeoxyglucose positron emission tomography: An efficient tool at the initial diagnosis of nonlesional late onset epilepsy
Impact of discontinuing non-pharmacological interventions on cognitive impairment in dementia patients by COVID-19 lockdown. A pilot observational, longitudinal, retrospective study carried out in an adult day center in Spain during the COVID-19 pandemic
Measuring cognitive performance in older adults through completion time and accuracy in brain games
Changes in Brain Volume Resulting from Cognitive Intervention by Means of the Feuerstein Instrumental Enrichment Program in Older Adults with Mild Cognitive Impairment (MCI): A Pilot Study
A socially prescribed community service for people living with dementia and family carers and its long-term effects on well-being
Functional Decline and Body Composition Change in Older Adults With Alzheimer Disease: A Retrospective Cohort Study at a Japanese Memory Clinic
The Feasibility of Differentiating Lewy Body Dementia and Alzheimer's Disease by Deep Learning Using ECD SPECT Images
Association of Low Muscle Mass With Cognitive Function During a 3-Year Follow-up Among Adults Aged 65 to 86 Years in the Canadian Longitudinal Study on Aging
Impact of day care services on physical and cognitive functions in old people with dementia in a medical center in central Taiwan
Self-reported mid- to late-life physical and recreational activities: Associations with late-life cognition
Combined association of gait speed and processing speed on cardiometabolic disease mortality risk in the US older adults: a prospective cohort study from NHANES
Mediation of Post-Stroke Function by Cognition in the Canadian Longitudinal Study on Aging
Leisure-Time Physical Activity May Attenuate the Impact of Diabetes on Cognitive Decline in Middle-Aged and Older Adults: Findings From the ELSA-Brasil Study
Functional connectome predicting individual gait function and its relationship with molecular architecture in Parkinson's disease
Estimating global prevalence of mild cognitive impairment and dementia in elderly with overweight, obesity, and central obesity: A systematic review and meta-analysis

Sleep, 24-Hour Activity Rhythms, and Subsequent Amyloid-Œ≤ Pathology
Analysis of Factors Affecting Depression in Older Adults in South Korea
Lifespace metrics of older adults with mild cognitive impairment and dementia recorded via geolocation data
Effects of Leisure Activities on the Cognitive Ability of Older Adults: A Latent Variable Growth Model Analysis
Long Non-Coding RNAs and Alzheimer's Disease: Towards Personalized Diagnosis
A 20-s Video-Based Assessment of Cognitive Frailty: Results from a Cohort Study within the Precision Aging Network
Combined effects of maximal oxygen uptake and glucose status on mortality: The Prospective KIHD cohort study
Effects of folic acid supplementation on cognitive impairment: A meta-analysis of randomized controlled trials
Longitudinal cognitive decline characterizes the profile of non-PD-manifest GBA1 mutation carriers
Metabolic dysfunction-associated steatotic liver disease as a predictor of cognitive performance: An 11-year population-based follow-up study
Metabolically Healthy Overweight and Obesity, Transition to Metabolically Unhealthy Status and Cognitive Function: Results from the Framingham Offspring Study
Mediterranean diet and risk for dementia and cognitive decline in a Mediterranean population
Relationships Between Functional Impairment, Depressive Symptoms, and Ageing Attitudes in Older Adults
Cognitive Digital Intervention for Older Patients with Parkinson's Disease during COVID-19: A Mixed-Method Pilot Study
Marital Status-Specific Associations Between Multidomain Leisure Activities and Cognitive Reserve in Clinically Unimpaired Older Adults: Based on a National Chinese Cohort
Dietary patterns and trajectories of global- and domain-specific cognitive decline in the Lothian Birth Cohort 1936
Physical frailty predicts cognitive decline among community-dwelling older Japanese women: A prospective cohort study from the Otassha study
Specific neuropsychiatric symptoms are associated with functional decline trajectories in Alzheimer's disease and Lewy body dementia: a five-year follow-up study
The association between psychological distress and risk of incident functional disability in elderly survivors after the Great East Japan Earthquake: The mediating effect of lifestyle and bodily pain
Examining Physical Wellness as the Fundamental Element for Achieving Holistic Well-Being in Older Persons: Review of Literature and Practical Application in Daily Life
Association of Dietary and Supplement Intake of Antioxidants with Risk of Dementia: A Meta-Analysis of Cohort Studies
Influence of Mild Cognitive Impairment on LSVT¬Æ BIG Therapy Effectiveness in People with Parkinson's Disease: A Retrospective Cohort Study
Preclinical Stage Alzheimer's Disease Detection Using Magnetic Resonance Image Scans
Sex differences in dementia and response to a lifestyle intervention: Evidence from Nordic population-based studies and a prevention trial
Parkinsonian gait in elderly people: Significance of the threshold value of two and more falls per year
Frailty and its combined effects with lifestyle factors on cognitive function: a cross-sectional study
Clinical trials in vascular cognitive impairment following SPRINT-MIND An international perspective
Effects of osteoporosis treatment and multicomponent integrated care on intrinsic capacity and happiness among rural community-dwelling older adults: the Healthy Longevity and Ageing in Place (HOPE) randomised controlled trial
Impact of Cognitive Reserve and Premorbid IQ on Cognitive and Functional Status in Older Outpatients
Associations between Sedentary Duration and Cognitive Function in Older Adults: A Longitudinal Study with 2-Year Follow-Up
Historic Cognitive Function Trajectories as Predictors of Sedentary Behavior and Physical Activity in Older Adults
Cardiovascular-kidney-metabolic syndrome and incidence of dementia among older adults
Temporal trends and disparities of population attributable fractions of modi fi able risk factors for dementia in China: a time-series study of the China health and retirement longitudinal study (2011 - 2018)
Impact of Physical Activity on All-Cause Mortality According to Specific Cardiovascular Disease
Changes in Satisfaction with Meaningful Activities and Onset of Depressive Symptoms among Community-Dwelling Japanese Older Adults: A Population-Based Study Before and During the COVID-19 Pandemic
Breakfast Skipping and Declines in Cognitive Score Among Community-Dwelling Older Adults: A Longitudinal Study of the HEIJO-KYO Cohort
Body mass index and cognitive decline among community-living older adults: the modifying effect of physical activity
Automated detection of cerebral microbleeds on T2*-weighted MRI
Characterizing the physical function decline and disabilities present among older adults with fecal incontinence: a secondary analysis of the health, aging, and body composition study
Association between frequency of going out and mild cognitive impairment in community-dwelling older adults: a pilot study in frailty prevention groups
Education and Cardiovascular Health as Effect Modifiers of APOE Œµ4 on Dementia: The Atherosclerosis Risk in Communities Study
Domain-Specific Evaluation of Exergame Metrics Among Older Adults With Mild Neurocognitive Disorder: Secondary Analysis of 2 Randomized Controlled Trials
The Diabetes Prevention Program and Its Outcomes Study: NIDDK's Journey Into the Prevention of Type 2 Diabetes and Its Public Health Impact
Association of Pulmonary Function With Cognitive Decline in Older Adults: A Nationwide Longitudinal Study in China
Cognitive Health and Quality of Life After Surviving Sepsis: A Narrative Review
Cooking with biomass fuels increased the risk for cognitive impairment and cognitive decline among the oldest-old Chinese adults (2011-2018): A prospective cohort study
Predicting individual brain MRIs at any age using style encoding generative adversarial networks
Effects of a multidomain intervention against cognitive decline on dementia risk profiles - Results from the AgeWell.de trial

Methods for Robot Behavior Adaptation for Cognitive Neurorehabilitation
Predictive value of synaptic plasticity for functional decline in patients with multiple sclerosis
Longitudinal and Combined Smartwatch and Ecological Momentary Assessment in Racially Diverse Older Adults: Feasibility, Adherence, and Acceptability Study
Transportation physical activity earlier in life and areas of the brain related to dementia later in life
Prevalence and persistent prescription of analgesic drugs in persons admitted with dementia to a nursing home - A longitudinal study
Prediction of Cognitive Impairment Risk among Older Adults: A Machine Learning-Based Comparative Study and Model Development
Association between perceived value of adopting new behaviors and incident disability among Japanese community-dwelling older adults
Associations between Recreational Screen Time and Brain Health in Middle-Aged and Older Adults: A Large Prospective Cohort Study
Engaging caregivers to use an evidence-based intervention for medicaid beneficiaries with Alzheimer's disease: a pilot study
Association of Predicted Visceral Fat Percentage With Dementia Risk in Older Adults
Predictors of emergency department visit among people with dementia in Taiwan
Super Movers: Epidemiology and Biology of a Novel Exceptional Aging Phenotype
Unraveling the link between physical activity and cognitive function: the mediating impact of depressive symptoms
Reliability of Gait Analysis Using ORPHE ANALYTICS in Patients with Type 2 Diabetes: A Single-center Observational Study
Systematic identification and quantification of factors and their interactions with age, sex, and panel wave influencing cognitive function in Korean older adults
Do cerebral microbleeds increase the risk of dementia? A systematic review and meta-analysis
Association of Subjective Memory Complaints With White Matter Hyperintensities and Cognitive Decline Among Older Adults in Chicago, Illinois
Building research in diet and cognition (BRIDGE): Baseline characteristics of older obese African American adults in a randomized controlled trial to examine the effect of the Mediterranean diet with and without weight loss on cognitive functioning
An 18-Year Study of Changes in Neurocognitive Function and Associations with Repetitive Head Trauma among Former Collegiate American Football Players: A Case Series
Implementing Dementia Care Management into routine care: protocol for a cohort study in Siegen-Wittgenstein, Germany (RoutineDeCM)
How Selection Over Time Contributes to the Inconsistency of the Association Between Sex/Gender and Cognitive Decline Across Cognitive Aging Cohorts
A nutritionally focused program for community-living older adults resulted in improved health and well-being
Daily Level Association of Physical Activity and Performance on Ecological Momentary Cognitive Tests in Free-living Environments: A Mobile Health Observational Study
Nutritional Status and Risks of Cognitive Decline and Incident Neurocognitive Disorders: Singapore Longitudinal Ageing Studies
Epidemiology of Motoric Cognitive Risk Syndrome in the Kerala Einstein Study: Protocol for a Prospective Cohort Study
Normative Aging for an Individual's Full Brain MRI Using Style GANs to Detect Localized Neurodegeneration
Predictors of improvement in urinary incontinence in the postacute setting: A Canadian cohort study
Unsupervised multimodal modeling of cognitive and brain health trajectories for early dementia prediction
Rothman-Keller model for risk prediction of motoric cognitive risk syndrome in older Chinese adults: A proof-of-concept study
Motoric Cognitive Risk Syndrome and Cognitive Frailty
Association of cognitive reserve with transitions across cognitive states and death in older adults: A 15-year follow-up study
Uncoupling of Ca2+ sparks from BK channels in cerebral arteries underlies hypoperfusion in hypertension-induced vascular dementia
Altered Motor Activity Patterns within 10-Minute Timescale Predict Incident Clinical Alzheimer's Disease
Association of step counts with cognitive function in apparently healthy middle-aged and older Japanese men
Sense of purpose in life and allostatic load in two longitudinal cohorts
End-of-Life Symptoms in Persons Dying With Advanced Dementia in the Community Setting: Findings From IN-PEACE
Physical Frailty Phenotype and the Development of Geriatric Syndromes in Older Adults with Coronary Heart Disease
Efficacy of computerized cognitive training using mobile devices to promote cognitive functioning in community-dwelling older adults with or without risk of mild cognitive impairment: A multi-centre longitudinal study
A Multicomponent Home-Based Intervention for Neuropsychiatric Symptoms in People With Dementia and Caregivers' Burden and Depression: A 6-Month Longitudinal Study
Association between Marital Status and Cognitive Impairment in a Multi-Ethnic Asian Population
Higher Serum Brain-Derived Neurotrophic Factor Levels Are Associated With a Lower Risk of Cognitive Decline: A 2-Year Follow Up Study in Community-Dwelling Older Adults
Contribution of social activity participation to the relationship between sensory impairment, physical performance and cognitive decline: a longitudinal study in China
Physical Performance and Cognition as Predictors of Instrumental Activities of Daily Living After Stroke: A Prospective Multicenter Cohort Study
Older Bereaved Individuals' Experiences of Cognitive-Behavioral Therapy for Complicated Grief Reactions: A Qualitative Multistage Focus Group Approach
Cardiovascular Risk Factors and Risk of Alzheimer Disease and Mortality: A Latent Class Approach
Association of cognitive and structural correlates of brain aging and incident epilepsy. The Framingham Heart Study
The influence of adiposity on the interactions between strength, physical function and cognition among older adults in the Australian Diabetes, Obesity and Lifestyle (AusDiab) study
Regional network covariance patterns of white matter integrity related to cardiorespiratory fitness in healthy aging
A Brief History of the Progress in Our Understanding of Genetics and Lifestyle, Especially Diet, in the Risk of Alzheimer's Disease

Trajectories of Cognition and Daily Functioning Before and After Incident Diabetes
Handgrip strength but not SARC-F score predicts cognitive impairment in older adults with multimorbidity in primary care: a cohort study
Impact of mild cognitive impairment on unplanned readmission in patients with coronary artery disease
Clustering on longitudinal lifestyle trajectories and their impact on cognitive performance
Bidirectional relationships between television viewing and loneliness in middle-aged and older men and women
Association of circadian rhythms with brain disorder incidents: a prospective cohort study of 72242 participants
Falls in the Oldest Old: Role of Gender, Living Situation, and Assistive Devices
Health-related behaviours and depression incidence amongst UK adults aged 50+: Evidence from the English Longitudinal Study of Ageing
Progress and Challenges in the Biology of FNDC5 and Irisin
The association between oral health and mild cognitive impairment among Iranian older adults: A cross-sectional study from Ardakan Cohort Study on Aging (ACSA)
Association of low birth weight with cardiometabolic diseases in Swedish twins: a population-based cohort study
Summer of Translational Aging Research for Undergraduates (STAR U): short-term outcomes of a training program to advance diversity in aging research
More fit KL-VS heterozygotes have more favorable AD-relevant biomarker profiles
The association between activities and cognitive health: Stratified analysis by APOE Œµ4 status
Transition to healthier lifestyle associated with reduced risk of incident dementia and decreased hippocampal atrophy
Melatonin for preventing postoperative delirium in elderly patients: A multicenter randomized placebo-controlled pilot study
Normal Pressure Hydrocephalus Overshadowed by Traumatic and Degenerative Spinal Diseases: A New Assessment Proposal
Association between the frailty index and vascular brain damage: The Treviso Dementia (TREDEM) registry
Enhancing Spatiotemporal Disease Progression Models via Latent Diffusion and Prior Knowledge
Reformulating NLP tasks to Capture Longitudinal Manifestation of Language Disorders in People with Dementia.
Cardiovascular Health and Near Visual Impairment Among Older Adults in the Republic of Congo:A Population-Based Study
Pain in Dementia: An Empirical Test of a Common Assumption
Associations of midlife fitness and obesity profiles with cognitive function
Association between drinking water sources and cognitive functioning in Chinese older adults residing in rural areas
Association of leisure activity changes and reversion from mild cognitive impairment to normal cognitive function among older adults: A prospective cohort study
Enriching Social Sharing for the Dementia Community: Insights from In-Person and Online Social Programs
Modelling analysis of dietary behaviors and oral health status to assess the impact on the mental well-being of elderly individuals: a cross-sectional survey study
Pathways explaining racial/ethnic and socio-economic disparities in incident all-cause dementia among older US adults across income groups
Functional fitness, lifestyle and demographic factors as predictors of perceived physical and mental health in older adults: A structural equation model
Circadian Rhythm Changes in Healthy Aging and Mild Cognitive Impairment
Feasibility, Acceptability, and Preliminary Impact of Full-Body Interaction on Computerized Cognitive Training Based on Instrumental Activities of Daily Living: A Pilot Randomized Controlled Trial with Chronic Psychiatric Inpatients
Association Between Mobility Limitations and Cognitive Decline in Community-Dwelling Older Adults: The English Longitudinal Study of Ageing
Mapping 15-year depressive symptom transitions in late life: population-based cohort study
Diet Quality and Cognitive Performance in Australian Adults Aged 55-85 Years: A Cross-Sectional Analysis of the Hunter Community Study Cohort
One-Year Change in Locus of Control among People with Dementia
Mismatch of MRI White Matter Hyperintensities and Gait Function in Patients With Cerebral Small Vessel Disease
Association Between Social Participation and Disability-free Life Expectancy in Japanese Older People: The Ohsaki Cohort 2006 Study
Prospective Study of Engagement in Leisure Activities and All-Cause Mortality Among Older Japanese Adults
Association between Accelerometer-Measured Light-Intensity Physical Activity and Cognitive Function in Older Adults
IADL for identifying cognitive impairment in Chinese older adults: insights from cross-lagged panel network analysis
Elder Mistreatment Experienced by Older Caregiving Adults: Results from a National Community-Based Sample
A fast approximate EM algorithm for joint models of survival and multivariate longitudinal data
A unique neuropsychophysiological approach to objectify emotion (dys)regulation in healthy older adults during the COVID-19 pandemic
Intrinsic capacity and its associations with incident dependence and mortality in 10/66 Dementia Research Group studies in Latin America, India, and China: A population-based cohort study
Longitudinal association between depressive symptoms and cognitive decline among middle-aged and elderly population
Cross-sectional associations between neighborhood characteristics, cognition and dementia risk factor burden in middle-aged and older Australians
Transferability of Alzheimer?s disease progression subtypes to an independent population cohort
The Neurocognitive Study for the Aging: Longitudinal Analysis on the Contribution of Sex, Age, Education and APOE Œµ4 on Cognitive Performance
From hypoxic pockets to daily routines: linking brain oxygenation and cognitive resilience
Relationship between sleep, physical fitness, brain microstructure, and cognition in healthy older adults: A pilot study
Leisure Activities, APOE Œµ4, and Cognitive Decline: A Longitudinal Cohort Study
Associations Between Blood-Based Biomarkers and Cognitive and Functional Trajectories Among Participants of the MEMENTO Cohort

The factors associated with cognitive function among community-dwelling older adults in Taiwan
Efficacy and Feasibility of Remote Cognitive Remediation Therapy in Parkinson's Disease: A Randomized Controlled Trial
Uncontrolled hypertension among adult hypertensive patients in Addis Ababa public hospitals: A cross-sectional study of prevalence and associated factors
Early detection of COVID-19 in the UK using self-reported symptoms: a large-scale, prospective, epidemiological surveillance study
Associations between Physical Performance Tests with Cognitive Changes: The Moderating Effect of Cognitive Status
Development of prognostic models for survival and care status in sporadic Creutzfeldt-Jakob disease
Association between osteoarthritis and urinary tract infection in older adults: A nationwide population-based cohort study
Rural versus Urban Residence in Adulthood and Incident Cognitive Impairment
Cognitive rehabilitation for people with mild to moderate dementia
Development of a Palliative Care Approach for Primary Progressive Aphasia: My Experience as a Person Living With This Rare Disorder
A multimodal non-pharmacological intervention for persons with dementia in nursing homes: a multicentre randomised controlled trial
Joint daily functional trajectory and risk of new-onset Alzheimer ' s disease and related dementias in older adults with normal and abnormal weight
Association of drug burden index with delirium in community-dwelling older adults with dementia: a longitudinal observational study
Mortality rates in Alzheimer's disease and non-Alzheimer's dementias: a systematic review and meta-analysis
Associations of Subjective Memory with Life Space and Neighborhood Built Environment in Older Adults in the ACTIVE Study
Association between teeth loss and nasogastric tube feeding dependency in older adults from Taiwan: a retrospective cohort study
Cardiovascular Risk Factor Trajectories Since Childhood and Cognitive Performance in Midlife The Cardiovascular Risk in Young Finns Study
Correlation of frailty assessment metrics in one-year follow-up of aged care residents: a sub-study of a randomised controlled trial
Revision total hip arthroplasty for aseptic loosening compared with primary total hip arthroplasty for osteoarthritis: long-term clinical, functional and quality of life outcome data
A scoring system and seven factors associated with certification for Japanese long-term care insurance in older people
Distinguishing Healthy Ageing from Dementia: A Biomechanical Simulation of Brain Atrophy Using Deep Networks
Residential greenness, air pollution and incident neurodegenerative disease: A cohort study in China
segcsvdWMH: A Convolutional Neural Network-Based Tool for Quantifying White Matter Hyperintensities in Heterogeneous Patient Cohorts
Association of Posture, Gait, and Auditory Functioning with Cognitive Status in a Cohort of Community-Dwelling Older Adults
Longitudinal Associations Between Sleep and Cognitive Function in a Cohort of Older Puerto Rican Adults: Sex and Age Interactions
What Tools Can We Use to Screen for Fall Risk in Older Patients with Mild Cognitive Impairment? Findings from the MEMENTO Cohort
The circadian rest-activity pattern predicts cognitive decline among mild-moderate Alzheimer's disease patients
Cognitive decrement in older adults with symptomatic peripheral artery disease
Modifiable risk factors for mild cognitive impairment among cognitively normal community-dwelling older adults: A systematic review and meta-analysis
Quantifying the Varying Predictive Value of Physical Activity Measures Obtained from Wearable Accelerometers on All-Cause Mortality over Short to Medium Time Horizons in NHANES 2003-2006
Participatory and Receptive Arts Engagement in Older Adults: Associations with Cognition Over a Seven-Year Period
The Canadian platform for research online to investigate health, quality of life, cognition, behaviour, function, and caregiving in aging (CAN-PROTECT): Study protocol, platform description, and preliminary analyses
Can Older Adult Patients with Hip Fractures Have Their Discharge Destination Predicted by Physical Performance Measures?
Benefits of nostalgia in vulnerable populations
CogTrack: A Proof of Concept for Cognition Tracker
Association of Loneliness with Functional Connectivity MRI, Amyloid-Œ≤ PET, and Tau PET Neuroimaging Markers of Vulnerability for Alzheimer's Disease
Understanding the Experiences, Challenges, and Needs of Dementia Caregivers in the Indian Subcontinent
Predictors for the development of motoric cognitive risk syndrome in older adults
Prospective association of general anesthesia with risk of cognitive decline in a Chinese elderly community population
A WeChat-based Intervention, Wellness Enhancement for Caregivers (WECARE), for Chinese American Dementia Caregivers: Pilot Assessment of Feasibility, Acceptability, and Preliminary Efficacy
Co-Designing a Palliative Dementia Care Framework to Support Holistic Assessment and Decision Making: The EMBED-Care Framework
Change in Healthy Lifestyle and Subsequent Risk of Cognitive Impairment Among Chinese Older Adults: A National Community-Based Cohort Study
Inverse Association between Cheese Consumption and Lower Cognitive Function in Japanese Community-Dwelling Older Adults Based on a Cross-Sectional Study
A cross-sectional survey of a public, evidence-based multimodal program for cognitive health in older adults
Variability in body weight and body composition and cognitive trajectories in older adults in the United States
The age-related contribution of cognitive function to dual-task gait in middle-aged adults in Spain: observations from a population-based study
Cerebrovascular co-pathology and cholinergic white matter pathways along the Lewy body continuum
Substitutions of Sleep, Physical Activity, and Sedentary Behaviour and the Risk of Dementia and Stroke
A Variational Approximation for Analyzing the Dynamics of Panel Data
Internet use, physical activity, and cognitive function in Chinese older adults: a cross-lagged panel analysis
Daily fluid intake and brain amyloid deposition: A cohort study

Handgrip Strength, a Component of Physical Frailty Contributing Significantly To Cognitive Impairment: Evidence from LASI Wave 1 (2017-18)
Attitudes towards dementia prevention in Austria-Results of an exploratory study
Associated factors of new subjective cognitive decline complaints after a 6-month period among community-dwelling older adults during the COVID-19 pandemic in Japan
Antidepressant Use and Progression of Mild to Moderate Alzheimer's Disease: Results from the European ICTUS Cohort
Investigating the prevalence of cognitive impairment and dementia in the Northern Ireland Cohort for the Longitudinal Study of Ageing (NICOLA): the Harmonised Cognitive Assessment Protocol (HCAP) cross-sectional substudy
Palliative Care Program for Community-Dwelling Individuals With Dementia and Caregivers The IN-PEACE Randomized Clinical Trial
Nutrition state of science and dementia prevention: recommendations of the Nutrition for Dementia Prevention Working Group
Effects of an integrated social-art intervention on cognitive and psychosocial outcomes among older adults with mild cognitive impairment in nursing homes: a mixed methods study
Longitudinal Changes in Dual Decline in Memory and Gait Speed Association with Instrumental Activities of Daily Living: Findings from the National Social Life, Health, and Aging Project
Diet and lifestyle impact the development and progression of Alzheimer's dementia
Long-term intake of total energy and fat in relation to subjective cognitive decline
Home-based family caregiver-delivered music and reading interventions for people living with dementia (HOMESIDE trial): an international randomised controlled trial
Risk of Parkinson's disease in people aged ,â• 50 years with new-onset anxiety: a retrospective cohort study in UK primary care
Circadian Rest-Activity Rhythms, Delirium Risk, and Progression to Dementia
The associations of life quality, depression, and cognitive impairment with mortality in older adults with COVID-19: a prospective, observational study
Care for older adults with dementia: PACE day care or residential dementia care units?
Handgrip Strength Is Related to Hippocampal and Lobar Brain Volumes in a Cohort of Cognitively Impaired Older Adults with Confirmed Amyloid Burden
A longitudinal observational study of home-based conversations for detecting early dementia: protocol for the CUBOId TV task
Neighborhood resources and risk of cognitive decline among a community-dwelling long-term care population in the U.S.
Dietary amino acid intake and sleep duration are additively involved in future cognitive decline in Japanese adults aged 60 years or over: a community-based longitudinal study
A Machine Learning Model to Harmonize Volumetric Brain MRI Data for Quantitative Neuroradiologic Assessment of Alzheimer Disease
Rehabilitation after intercurrent morbid event of patients suffering from neurocognitive disorders in a pilot unit: management and outcomes
Demonstration of age-related blood-brain barrier disruption and cerebromicrovascular rarefaction in mice by longitudinal intravital two-photon microscopy and optical coherence tomography
Association between anxiety symptoms and Alzheimer?s disease biomarkers in cognitively healthy adults: A systematic review and meta-analysis
Can predicting COVID-19 mortality in a European cohort using only demographic and comorbidity data surpass age-based prediction: An externally validated study
Can Leucine Supplementation Improve Frailty Index Scores?
Denture Use Mitigates the Cognitive Impact of Tooth Loss in Older Adults
Effects of physical activity on cognitive function among patients with diabetes in China: a nationally longitudinal study
Muscle weakness is a prognostic indicator of disability and chronic disease multimorbidity
Ikigai and subsequent health and wellbeing among Japanese older adults: Longitudinal outcome-wide analysis
Appendicular Lean Mass, Grip Strength, and the Incidence of Dementia Among Older Adults in the Health ABC Study
Interocular retinal asymmetry and cognitive frailty: A prospective cohort study
Is treated hypertension associated with a lower 1-year mortality among older multimorbid residents of long -term care facilities?
Psychological Resilience, Cognitive Function, and Physical Activity: A Longitudinal Mediation Study
Health, functioning and social engagement among older people living in long-term care facilities during the COVID-19 lockdown in Finland: a register-based cohort study
PREDICTING CONVERSION OF MILD COGNITIVE IMPAIRMENT TO ALZHEIMER'S DISEASE BY MODELLING HEALTHY AGEING TRAJECTORIES
Association of resting heart rate with cognitive decline and dementia in older adults: A population-based cohort study
Investigating cognition in people with diabetes-related foot ulcers: a study protocol
The relationship between cardiovascular risk and lifestyle activities on hippocampal volumes in normative aging
Longitudinal reciprocal associations between volunteering, health and well-being: evidence for middle-aged and older adults in Europe
Risk Factors of Skilled Nursing Facility Admissions and the Interrelation With Hospitalization and Amount of Informal Caregiving Received
Do High Mental Demands at Work Protect Cognitive Health in Old Age via Hippocampal Volume? Results From a Community Sample
Physical activity partly mediates the association between cognitive function and depressive symptoms
ICAM-Reg: Interpretable Classification and Regression With Feature Attribution for Mapping Neurological Phenotypes in Individual Scans
Longitudinal neighbourhood determinants with cognitive health and dementia disparities: protocol of the Multi-Ethnic Study of Atherosclerosis Neighborhoods and Aging prospective cohort study
Sex differences in the single-nuclei hippocampal neurovascular unit transcriptomic response to type 2 diabetes
Change in cognitive performance during seven-year follow-up in midlife is associated with sex, age, and education - The Cardiovascular Risk in Young Finns Study
ORAL CARE AND DENTAL INTERVENTIONS SHOW MIXED AND UNCLEAR EFFECTS ON COGNITIVE FUNCTION

Impact of air pollution and behavioral factors on cognitive decline among middle-aged and elderly populations in China: a retrospective cohort study based on CHARLS
Social support and cognitive activity and their associations with incident cognitive impairment in cognitively normal older adults
Association of Therapy Time and Cognitive Recovery in Stroke Patients in Post-Acute Rehabilitation
Association of solid fuel use with cognitive function and the modifying role of lifestyle: A nationwide cohort study in China
Association between sedentary behavior and dementia: a systematic review and meta-analysis of cohort studies
A large-scale multi-centre study characterising atrophy heterogeneity in Alzheimer's disease
Correlation Between Oral Health Behaviors and the Susceptibility to Cognitive Function Impairment in the Taiwanese Older Population
Profile of non-invasive physical health indicators associated with cognitive performance in Chinese older adults: evidence from the China Health and Retirement Longitudinal Study
Benefits of an educational intervention on functional capacity in community-dwelling older adults with frailty phenotype: A randomized controlled trial
Profile for Brain Disease Research Infrastructure for Data Gathering and Exploration (BRIDGE) Platform
Adherence to the Mediterranean diet and waist-to-hip ratio in middle-aged postmenopausal women are the main factors associated with semantic verbal fluency 12 years later
Free-living wrist and hip accelerometry forecast cognitive decline among older adults without dementia over 1-or 5-years in two distinct observational cohorts
Cognition in People Aged 80 Years and Older: Determinants and Predictors of Change from a Population-Based Representative Study in Germany
Association of Physical Activity and APOE Genotype With Longitudinal Cognitive Change in Early Parkinson Disease
A Study on Feasibility and Acceptability of an AI-Powered VR/AR Cognitive Rehabilitation Platform for Patients with Alzheimer's Disease and Dementia
My experience of person-centered and personalized care in early-stage primary progressive aphasia
Association Between New-Onset Medicaid Home Care and Family Caregivers' Health
A meta-analysis of technology use and cognitive aging
Content-Based Brain Magnetic Resonance Image Retrieval and Classification With the Proposed Deep Learning and Tissue-Based System
Neighborhood Perceptions Are Associated With Intrinsic Amygdala Activity and Resting-State Connectivity With Salience Network Nodes Among Older Adults
Association of Sex Mismatch Between Patients and Speech-Language Therapists with Favorable Outcome in Eating and Swallowing Functions in Dementia Care: A Pilot Study
Interrelation between domains of functioning and white matter hyperintensities in geriatric memory clinic patients: a holistic approach through network analysis
An individualized telephone-based care support program for rural family caregivers of people with dementia: study protocol for a cluster randomized controlled trial
Cross-cohort generalizability of deep and conventional machine learning for MRI-based diagnosis and prediction of Alzheimer's disease
Effects of leisure activities and general health on the survival of older people: a cohort study in China
Women With Cerebral Infarction Feature Worse Clinical Profiles at Admission but Comparable Success to Men During Long-Term Inpatient Neurorehabilitation
Health literacy, dementia knowledge and perceived utility of digital health modalities among future health professionals
Vitamin D Concentration and Motoric Cognitive Risk in Older Adults: Results from the Gait and Alzheimer Interactions Tracking (GAIT) Cohort
Factors Contributing to Health Deterioration in the Japanese Long-Term Care Insurance System in Terms of Instrumental Activities of Daily Living
Disentangling relationships between Alzheimer's disease plasma biomarkers and established biomarkers in patients of tertiary memory clinics
Using shoe-mounted inertial sensors and stepping exergames to assess the motor-cognitive status of older adults: A correlational study
Bodyweight change and cognitive performance in the older population
The impact of malnutrition on cognition in older adults: A systematic review
Unravelling Shared Pathways Linking Metabolic Syndrome, Mild Cognitive Impairment, Dementia, and Sarcopenia
A systematic review and meta-analysis of reablement interventions for people in permanent residential aged care homes
Race, polygenic risk and their association with incident dementia among older US adults
Individual and joint associations between sleep duration and physical activity with cognitive function: a longitudinal analysis among middle-aged and older adults in China
Dementia is a surrogate for frailty in hip fracture mortality prediction
Risk Factor Profile in Youth, Genetic Risk, and Adulthood Cognitive Function: The Cardiovascular Risk in Young Finns Study
It's Not Too Late: Physical and Social Activity Slow Cognitive Decline in Those Living With Mild Cognitive Impairment
Data-driven clustering approach to identify novel clusters of high cognitive impairment risk among Chinese community-dwelling elderly people with normal cognition: A national cohort study
Fifteen Years of the Australian Imaging, Biomarkers and Lifestyle (AIBL) Study: Progress and Observations from 2,359 Older Adults Spanning the Spectrum from Cognitive Normality to Alzheimer's Disease
The A-to-Z factors associated with cognitive impairment. Results of the DeCo study
SARC-F predicts poor motor function, quality of life, and prognosis in older patients with cardiovascular disease and cognitive impairment
Demo Abstract: An LLM-Powered Multimodal Mobile Sensing System for Personalized and Interactive Health Behavior Analysis
A Workshop on Cognitive Aging and Impairment in the 9/11-Exposed Population
Large inter-arm systolic blood pressure difference is associated with cognitive impairment in older adults: a cross-sectional study in rural southwest China
Living with dementia during the COVID-19 pandemic: insights into identity from the IDEAL cohort
Leisure engagement in older age is related to objective and subjective experiences of aging
Sex-specific relationships between obesity, physical activity, and gray and white matter volume in cognitively unimpaired older adults

The Mediterranean-DASH Intervention for Neurodegenerative Delay (MIND) diet is associated with physical function and grip strength in older men and women
The combined effect of physical activity and fruit and vegetable intake on decreasing cognitive decline in older Taiwanese adults
Behavioral Differences Between Subject Groups Identified Using Smart Homes and Change Point Detection
Leisure Participation and Cognitive Impairment Among Healthy Older Adults in China
Impact of COVID-19 on 'Living Well' with Mild-to-Moderate Dementia in the Community: Findings from the IDEAL Cohort
Effects of APOE84 genotype on age-associated change in cognitive functions among Japanese middle-aged and older adults: A 20-year follow-up study
Psychological Stress Detection in Older Adults with Cognitive Impairment Using Photoplethysmography
Associations of lifetime concussion history and repetitive head impact exposure with resting-state functional connectivity in former collegiate American football players: An NCAA 15-year follow-up study
Translating lifestyle interventions for optimal brain health in Africa
Observing videos of mastication in dementia: Results of a clustered randomised controlled trial
Diagnosis of Alzheimer's Disease Using Atlas-Based Volume Measurement Method on 3D T1 Weighted MR Images
Is there a combined effect of depression and cognitive reserve on cognitive function? Findings from a population-based study
Relationship between domain-specific physical activity and cognitive function in older adults - findings from NHANES 2011-2014
Dose-response association of the duration and volume of physical activity with incident dementia: A cosmic collaborative cohort study
Predicting positron emission tomography brain amyloid positivity using interpretable machine learning models with wearable sensor data and lifestyle factors
Relationship between different physical activity parameters and cognitive impairment in middle-aged and older adults: insights from a 4-year longitudinal study
Early Benefits with Potential Long-Term Risks of a Comprehensive Intervention on Serum Cortisol Levels and Cognitive Performance in Patients with Alzheimer's Disease
Cohort and Period Effects as Explanations for Declining Dementia Trends and Cognitive Aging
Reduced levels of angiogenesis biomarkers predict increased symptom severity in Chinese Americans with Alzheimer's disease with demographic-specific effect
Conditional VAEs for Confound Removal and Normative Modelling of Neurodegenerative Diseases
Association between physical activity measured by accelerometry and cognitive function in older adults: a systematic review
The personhood in patients with dementia assessed in Italian healthcare professionals: an explorative study
Employing Deep-Learning Approach for the Early Detection of Mild Cognitive Impairment Transitions through the Analysis of Digital Biomarkers
Lockdown Effects on Healthy Cognitive Aging During the COVID-19 Pandemic: A Longitudinal Study
A Multi-User Virtual Reality Social Connecting Space for People Living with Dementia and Their Support Persons: A Participatory Action Research Study
Anti-dementia drug-induced mania in elderly. Systematic review of case reports
Learning to synthesise the ageing brain without longitudinal data
Sarcopenia is associated with incident Alzheimer's dementia, mild cognitive impairment, and cognitive decline
Understanding engagement in diet and dementia prevention research among British South Asians: a short report of findings from a patient and public involvement group
Discontinuity of social support among US adults with cognitive impairment before and after the confirmed diagnosis of dementia: a matched ambidirectional cohort study
Risk factors for inadequate bowel preparation before colonoscopy: A meta-analysis
Lifestyles, Left Atrial Structure and Function, and Cognitive Decline in Adults with Metabolic Syndrome
Dietary patterns from youth to adulthood and cognitive function in midlife: The cardiovascular risk in Young Finns Study
Behavioral Phenotyping in a Murine Model of GBA1-Associated Parkinson Disease
Having hobbies and the risk of cardiovascular disease incidence: A Japan public health center-based study
High Cardiorespiratory Fitness Protects against Molecular Impairments of Metabolism, Heart, and Brain with Higher Efficacy in Obesity-Induced Premature Aging
A comparison over 2 decades of disability-free life expectancy at age 65 years for those with long-term conditions in England: Analysis of the 2 longitudinal Cognitive Function and Ageing Studies
Nativity differences in the relationship between handgrip strength and cognitive impairment in older Mexican Americans over 20 years of follow-up
Impact of physical activity on the depression and self-care ability among Chinese older adults during the COVID-19 pandemic: propensity score matching analysis
Sociodemographic characteristics, comorbidity, and baseline functional status of older patients treated in emergency departments during the COVID-19 pandemic and associations with mortality: an analysis based on the EDENCOVID cohort
Late-life physical activity, midlife-to-late-life activity patterns, APOE Œµ4 genotype, and cognitive impairment among Chinese older adults: a population-based observational study
Neuromuscular electrical stimulation to combat cognitive aging in people with spinal cord injury: protocol for a single case experimental design study
Relationship between falls, cognitive decline, and dementia in older adults: Insights from the Korean longitudinal study of aging, 2006-2020
Experiences of family caregivers of persons living with dementia with and without a smart- clothes assisted home nursing program during the heightened COVID-19 alert
Cognitive deficits in people who have recovered from COVID-19
Data-driven Development of Digital Health Applications on the Example of Dementia Screening
Sleep duration and leisure activities are involved in regulating the association of depressive symptoms, muscle strength, physical function and mild cognitive impairment
Cognitive Impairment Before Cardiac Surgery: A Prospective Single-Center Observational Analysis

Effect of self-determination theory-based integrated creative art (SDTICA) program on older adults with mild cognitive impairment in nursing homes: Study protocol for a cluster randomised controlled trial
Dietary patterns in middle age: effects on concurrent neurocognition and risk of age-related cognitive decline
Improving information sharing in Medicaid home and community-based services with learning health systems: Implications for older adults and individuals living with disabilities and dementia
A study of turn bias in people with idiopathic Parkinson's disease
Association between healthy lifestyle and cognitive decline, all-cause mortality, and mortality from cardiovascular and cerebrovascular diseases: a 10-year population-based prospective cohort study
Association of Loneliness With the Incidence of Disability in Older Adults With Hearing Impairment in Japan
Urban-Rural Disparity in Cognitive Performance Among Older Chinese Adults: Explaining the Changes From 2008 to 2018
Relationship between physical activity and cognitive functioning among older Indian adults
Original research: initial development of a pragmatic tool to estimate cognitive decline risk focusing on potentially modifiable factors in Parkinson's disease
Differentiating the influence of sedentary behavior and physical activity on brain health in late adulthood
Head Impact Exposure, Gray Matter Volume, and Moderating Effects of Estimated Intelligence Quotient and Educational Attainment in Former Athletes at Midlife
Prediction of Parkinson Disease Using Long-Term, Short-Term Acoustic Features Based on Machine Learning
Prevalence and Outcomes of Major Psychiatric Disorders Preceding Index Surgery for Degenerative Thoracic/Lumbar Spine Disease
Loneliness, depression, and cognitive function among Iranian older adults: is gender important?
A Mindfulness-Based Lifestyle Intervention for Dementia RiskReduction:Protocol for the My Healthy Brain FeasibilityRandomized Controlled Trial
Efficacy of faecal microbiota transplantation in patients with progressive supranuclear palsy-Richardson's syndrome: a phase 2, single centre, randomised clinical trial
A Shift Toward Supercritical Brain Dynamics Predicts Alzheimer's Disease Progression
Dementia and risk of visual impairment in Chinese older adults
Association of Hand Grip Strength with Mild Cognitive Impairment in Middle-Aged and Older People in Guangzhou Biobank Cohort Study
Association of cataract surgery with stroke among older adults in the United States
RETRACTED: Effects of Tai Chi on Patients with Mild Cognitive Impairment: A Systematic Review and Meta-analysis of Randomized Controlled Trials (Retracted Article)
Association between oral health and physio-cognitive decline syndrome of older adults in China and its sex differences: a cross-sectional study
Personalised treatment for cognitive impairment in dementia: development and validation of an artificial intelligence model
Water quality and neurodegenerative disease risk in the middle-aged and elderly population
Decreased Doublecortin (DCX) immunoreactivity in hippocampus after profound sensorineural hearing loss and vestibular dysfunction in adult mice
Learning patterns of the ageing brain in MRI using deep convolutional networks
Sedation-Associated Medications at Dementia Diagnosis, Their Receptor Activity, and Associations With Adverse Outcomes in a Large Clinical Cohort
Differential associations between simple physical performance tests with global and specific cognitive functions in cognitively normal and mild cognitive impairment: a cross-sectional cohort study of Asian community-dwelling older adults
Effects of a multicomponent intervention to slow mild cognitive impairment progression: A randomized controlled trial
Functional Decline Over Time and Change in Family and Other Unpaid Care Provided to Community-Dwelling Older Adults Living With and Without Dementia
Predictors of cognitive functioning trajectories among older Americans: A new investigation covering 20 years of age- and non-age-related cognitive change
Vitamin D and white matter hyperintensities: results of the population-based Heinz Nixdorf Recall Study and 1000BRAINS
Associations Between Hospice Care and Scary Family Caregiver Experiences
Cognitive Trajectories in Community-Dwelling Older Adults and Incident Dementia, Disability and Death: A 10-Year Longitudinal Study
Towards Real-World Dementia Care: Encouraging Activity Engagement Using an Intelligent Humanoid Robot
Cohort profile: Norwegian survey of health and ageing (NORSE)
Premature Cardiovascular Disease and Brain Health in Midlife The CARDIA Study
Barriers and Facilitators of Self-Management in Older People with Type 1 Diabetes: A Narrative Review Focusing on Cognitive Impairment
Modifiable Risk Factors for Cognitive Decline in Community-Dwelling Older Adults Differ by Sex and APOE4
A multi-omics analysis for the prediction of neurocognitive disorders risk among the elderly in Macao
Prevalence of cognitive frailty and its associated factors in a population of Iranian older adults
Evaluation of the feasibility of an Education-Career pathway in Healthcare for Older People (ECHO) for early career nurses
Machine learning functional impairment classification with electronic health record data
Association between physical activity levels and mild cognitive impairment in Chinese older adults: a cross-sectional study from the China health and retirement longitudinal study
Creating livable cities for healthy ageing: Cognitive health in older adults and their 15-minute walkable neighbourhoods
SMART-PET: a Self-SiMilARiTy-aware generative adversarial framework for reconstructing low-count [18F]-FDG-PET brain imaging
Utility of Clinical Frailty Scale in Intensive Care Unit
Association between solid cooking fuel and cognitive decline: Three nationwide cohort studies in middle-aged and older population
Overcoming Pandemic-Related Challenges in Recruitment and Screening
Association between multimorbidity trajectories and subsequent cognitive decline: evidence from two nationwide longitudinal cohorts

Longitudinal analysis of aging in place at TigerPlace: Resident function and well-being
CALS and ACB Scales are Associated with Physical and Cognitive Impairment and Predict Mortality in Nursing Home Residents
Timed Up and Go in People with Subjective Cognitive Decline Is Associated with Faster Cognitive Deterioration and Cortical Thickness
Gender differences in the relationship between informal caregiving and subjective health: the mediating role of health promoting behaviors
Glucose hypometabolism in the Auditory Pathway in Age Related Hearing Loss in the ADNI cohort
Effect of leisure activities on cognitive aging in older adults: A systematic review and meta-analysis
Severe menopausal symptoms linked to cognitive impairment: an exploratory study
Alignment of Dietary Patterns With the Dietary Guidelines for Americans 2015-2020 and Risk of All-Cause and Cause-Specific Mortality in the Women's Health Initiative Observational Study
Association between Sleep Duration and Mild Cognitive Impairment at Different Levels of Metabolic Disease in Community-Dwelling Older Chinese Adults
Voice Disorders and Hearing Loss May Be Additive Risk Factors for Depression in a National Cohort
Gait and Age-Related Hearing Loss Interactions on Global Cognition and Falls
Cognitive aging and reserve factors in the Metropolit 1953 Danish male cohort
Periodontal health, cognitive decline, and dementia: A systematic review and meta-analysis of longitudinal studies
Cohort profile: the Norwegian Registry of Persons Assessed for Cognitive Symptoms (NorCog) - a national research and quality registry with a biomaterial collection
Associations of handgrip strength asymmetry with physical frailty, cognitive impairment, and cognitive frailty: A cross-sectional finding from the Longitudinal Ageing Study in India (LASI), Wave 1 (2017-18)
Effects of cognitive interventions on quality of life among adults with mild cognitive impairment: A systematic review and meta-analysis of randomised controlled trials
Prior Incarceration and Performance on Immediate and Delayed Verbal Recall Tests: Results From National Longitudinal Study of Adolescent to Adult Health-Parent Study
Examining the risk of substance use and lifestyle factors on cognitive decline among older people in India
Applying dense-sampling methods to reveal dynamic endocrine modulation of the nervous system
Untargeted Metabolomic Profiling Reveals Differentially Expressed Serum Metabolites and Pathways in Type 2 Diabetes Patients with and without Cognitive Decline: A Cross-Sectional Study
Biodynamic Lighting to Support the Wellbeing of People Living with Dementia in Care Facilities
Microstructural Brain Changes Associated With Frailty in Older Adults: Insights From Diffusion Tensor Imaging
Freezing of gait in idiopathic normal pressure hydrocephalus
The alterations in multiple neurophysiological procedures are associated with frailty phenotype in older adults
Accuracy of Tau Positron Emission Tomography as a Prognostic Marker in Preclinical and Prodromal Alzheimer Disease A Head-to-Head Comparison Against Amyloid Positron Emission Tomography and Magnetic Resonance Imaging
Motor-Cognitive Interventions May Effectively Improve Cognitive Function in Older Adults with Mild Cognitive Impairment: A Randomized Controlled Trial
Influencing health behaviour using smart building interventions for people with dementia and mild cognitive impairment: expert interviews and a systematic literature review
The longitudinal relationship between levels of leisure-time physical activity and positive and negative affect among older foreign-born adults with mild cognitive impairment
The Impact of the COVID-19 Pandemic on Oldest-Old Social Capital and Health and the Role of Digital Inequalities: Longitudinal Cohort Study
Associations of baseline glycemic status and its transitions with cognitive and physical functioning decline
A Longitudinal Study on the Association of Interrelated Factors Among Frailty Dimensions, Cognitive Domains, Cognitive Frailty, and All-Cause Mortality
Very low levels of physical activity among patients hospitalized following hip fracture surgery: a prospective cohort study
Hippocampal vascularization patterns exert local and distant effects on brain structure but not vascular pathology in old age
Combinations of light, moderate, and vigorous physical activity in midlife and old age: implications for healthy cognitive aging
Role of leisure and sleep in promoting daily physical activity in adults with down syndrome
Feasibility of virtual Alzheimer's biomarker disclosure: Findings from an observational cohort
An online, public health framework supporting behaviour change to reduce dementia risk: interim results from the ISLAND study linking ageing and neurodegenerative disease
Design of the Think PHRESH longitudinal cohort study: Neighborhood disadvantage, cognitive aging, and Alzheimer's disease risk in disinvested, Black neighborhoods
Decoding the radiomic features of dorsolateral prefrontal cortex in individuals with accelerated cortical changes: implications for personalized transcranial magnetic stimulation
Determinants of Physical Functioning and Health-Related Quality of Life among Sexual and Gender Minority Older Adults with Cognitive Impairment
Exploring the Impact of Social Connection Dimensions on Cognitive Impairment in Older Adults: Observational Cohort Study
Longitudinal relationship between healthy lifestyle and cognitive function mediated by activities of daily living among middle-aged and older Chinese adults
Patterns, predictors, and outcomes of frailty trajectories in community-dwelling older adults: Results from the FREEDOM Cohort Study
Association of Plasma Amyloid-Œ≤ and Dementia Among Black and White Older Adults
Deep normative modelling reveals insights into early-stage Alzheimer's disease using multi-modal neuroimaging data
Long-term environmental enrichment is associated with better fornix microstructure in older adults
EPIC-Potsdam sub-cohort study: The early role of trace elements, oxidative stress, and anthropometrics in Alzheimer's disease and dementia onset

The influence of healthy lifestyle behaviors on cognitive function among older Chinese adults across age and gender: Evidence from panel data
Improving Cognitive Function with Nutritional Supplements in Aging: A Comprehensive Narrative Review of Clinical Studies Investigating the Effects of Vitamins, Minerals, Antioxidants, and Other Dietary Supplements
Association between long-term visit-to-visit resting heart rate variability and cognitive decline: evidence from two national cohorts
A cross-sectional study of the major risk factor at different levels of cognitive performance within Chinese-origin middle-aged and elderly individuals
Minimum toe clearance variability in older adults with mild cognitive impairment: Differences to healthy controls and effects of a dance intervention
Association of memory function with COVID-19 outcomes in adults aged 50 years and older: analysis of three prospective cohorts
GPS driving: a digital biomarker for preclinical Alzheimer disease
Sex differences in the confounders influencing the relationships linking socioeconomic factors and cognitive performance with family history of Alzheimer's disease and related dementias
Glycemic Dysregulations Are Associated With Worsening Cognitive Function in Older Participants at High Risk of Cardiovascular Disease: Two-Year Follow-up in the PREDIMED-Plus Study
Strong evidence for age as the single most dominant predictor of medically supervised driving test-mini mental status test outcomes provide only weak but significant moderate additional predictive value
Effects of interventions on cerebral perfusion in the Alzheimer?s disease spectrum: A systematic review
Socioeconomic inequalities in physical and cognitive functioning: cross-sectional evidence from 37 cohorts across 28 countries in the ATHLOS project
Risk of Psychosis among Patients with Dementia: the Roles of Loneliness, Life Satisfaction, Autonomy, Physical Health and Eyesight Loss
Blood Œ≤-synuclein is related to amyloid PET positivity in memory clinic patients
Adipose tissue coregulates cognitive function
Objectively assessed physical activity and sedentary behavior and global cognitive function in older adults: a systematic review
Association of Osteoarthritis With Changes in Structural Neuroimaging Markers Over Time Among Non-demented Older Adults
Estimating Prognosis and Frailty in Persons Aged ‚â•75 Years in the Emergency Department: Further Validation of Dynamic Silver Code
Investigation of the Effects of Polypharmacy on Cognitive Functions: Cohort Study
Loneliness and social isolation correlate with multiple modifiable risk factors for chronic diseases including dementia
A Decade Later on How to "Use It" So We Don't "Lose It": An Update on the Unanswered Questions about the Influence of Activity Participation on Cognitive Performance in Older Age
Oxidative damage in the gastrocnemius predicts long-term survival in patients with peripheral artery disease
Dual-task gait has prognostic value for cognitive decline in Parkinson's disease
TaKeTiNa Music Therapy for Outpatient Treatment of Depression: Study Protocol for a Randomized Clinical Trial
Change in cardiovascular health and rate of cognitive decline in older adults: a 15-year population-based study
Tooth Loss as a Predictor of Long-Term Care Requirements in the Elderly: A Study in Kobe City, Japan
The prevalence of non-pharmacological interventions in older homecare recipients: an overview from six European countries
Cognitive and emotional benefits of individual reminiscence therapy in elderly stroke survivors: study protocol for a randomized controlled study
Myelin Content and Gait Impairment in Older Adults with Cerebral Small Vessel Disease and Mild Cognitive Impairment
Diogenes syndrome in a 10-year retrospective observational study: An elderly case series in Tokyo
A proper combination of movement and stillness: the interaction effect of sleep duration and physical activity on longitudinal cognitive performance in Chinese middle-aged and older adults
SOCIOFAMILY RISK AND COGNITIVE IMPAIRMENT IN ELDERLY ADULTS TREATED AT THE GERIATRIC CENTER OF THE PERUVIAN AIR FORCE IN THE PERIOD 2017-2020
The association of agricultural and non-agricultural work on the healthy ageing of older adults in Japan: A 6-year longitudinal study from the Japan Gerontological evaluation study
Drugs to Treat Neuroinflammation in Neurodegenerative Disorders
Differential associations of physical job demands with cognitive impairment in Korean workers aged 45 and older: a 14-year longitudinal study using the Korean Longitudinal Study of Aging (KLoSA)
Sex modifies the association between cardiorespiratory fitness and insulin resistance in adults at risk for Alzheimer's disease
Feasibility and acceptability of a telephone-based ACT intervention for caregivers (TACTICs) of adults with Alzheimer's disease and related dementias (ADRD): a randomized pilot during the COVID-19 pandemic
The Effects of Social Interaction Intervention on Cognitive Functions Among Older Adults Without Dementia: A Systematic Review and Meta-Analysis
Resting State Networks Related to the Maintenance of Good Cognitive Performance During Healthy Aging
Joint developmental trajectories and temporal precedence of physical function decline and cognitive deterioration: A longitudinal population-based study
Functional Reserve: The Residual Variance in Instrumental Activities of Daily Living Not Explained by Brain Structure, Cognition, and Demographics
Driving Assessment for Persons with Dementia: How and when?
Frequency, duration, and type of physiotherapy in the week after hip fracture surgery-analysis of implications for discharge home, readmission, survival, and recovery of mobility
Job-related exhaustion risk variant in UST is associated with dementia and DNA methylation
Association between Active Mobility Index and sarcopenia among Japanese community-dwelling older adults
Cognitive impairment negatively impacts allied health service uptake: Investigating the association between health and service use
The Impact of COVID-19 Confinement on Cognition and Mental Health and Technology Use Among Socially Vulnerable Older People: Retrospective Cohort Study

White matter fibre density in the brain's inhibitory control network is associated with falling in low activity older adults
Mediating role of obstructive sleep apnea in altering slow-wave activity and elevating Alzheimer's disease risk: Pilot study from a northern Taiwan cohort
Association between obesity, physical activity, and cognitive decline in Chinese middle and old-aged adults: a mediation analysis
Can Virtual Reality Help Improve Motor and Cognitive Function in Active Aging in Older Adults? A Scoping Review
Differential physical activity patterns in cognitively impaired vs healthy older adults using accelerometers
Efficacy of Acupuncture Treatment for Incidence of Poststroke Comorbidities: A Systematic Review and Meta-Analysis of Nationalized Cohort Studies
Incidence, risk, and associated risk factors of stroke among people with different disability types and severities: A national population- based cohort study in Taiwan
Sleep Characteristics and Hearing Loss in Older Adults: The National Health and Nutrition Examination Survey 2005-2006
Microbiome signatures of virulence in the oral-gut-brain axis influence Parkinson's disease and cognitive decline pathophysiology
Associations of dietary markers with brain volume and connectivity: A systematic review of MRI studies
Transfer learning for cognitive reserve quantification
The role of diet on the risk of dementia in the oldest old: The Monzino 80-plus population-based study
COGNITIVE FUNCTION AND INDEPENDENCE IN THE OLDEST-OLD: REVIEW STUDY BASED ON AGING IN PLACE
Carnitine supplementation in progressive supranuclear palsy
The Association Between Leisure Activity Engagement and Health-Related Quality of Life in Middle-Aged and Older People With HIV
Enhancing Enrollment and Adherence in Long-Term Wearable Research on Dementia: Qualitative Systematic Review and Meta-Synthesis
Comparison of Two-Dimensional- and Three-Dimensional-Based U-Net Architectures for Brain Tissue Classification in One-Dimensional Brain CT
Sports-related concussion not associated with long-term cognitive or behavioural deficits: the PROTECT-TBI study
Altruistic Social Activity, Depressive Symptoms, and Brain Regional Gray Matter Volume: Voxel-Based Morphometry Analysis From 8,695 Old Adults
Brain reserve theory: Are adults with intellectual disability more vulnerable to age than peers with typical development?
Patient-Reported Functional Impairment Due to Hearing Loss and Tinnitus After Cisplatin-Based Chemotherapy
The Effects of the Health System Response to the COVID-19 Pandemic on Chronic Disease Management: A Narrative Review
Examining the role of race and quality of home health agencies in delayed initiation of home health services for individuals with Alzheimer's disease and related dementias (ADRD)
Investigating reliable amyloid accumulation in Centiloids: Results from the AMYPAD Prognostic and Natural History Study
Deep learning from MRI-derived labels enables automatic brain tissue classification on human brain CT
The association between sports social capital and cognitive health: A longitudinal study of middle-aged and elderly adults in China
Health Behavior Change to Mitigate Dementia Risk: An Updated Review
Accelerometer-measured physical activity and sedentary behavior in nonagenarians: Associations with self-reported physical activity, anthropometric, sociodemographic, health and cognitive characteristics
Biomarkers of Importance in Monitoring Heart Condition After Acute Myocardial Infarction
Older Adults' Engagement and Mood During Robot-Assisted Group Activities in Nursing Homes: Development and Observational Pilot Study
A 4-Bed Close-Observation Pod model of multidisciplinary care in hospital: A mixed methods study
Source-Specific Air Pollution and Loss of Independence in Older Adults Across the US
Chronic adolescent stress causes sustained impairment of cognitive flexibility and hippocampal synaptic strength in female rats
Effects of ambient temperature on hospital admissions and mortality among older adults with and without dementia in South Korea
Short-term ozone exposure and serum neural damage biomarkers in healthy elderly adults: Evidence from a panel study
Leisure-time physical activity mitigated the cognitive effect of PM2.5 and PM2.5 components exposure: Evidence from a nationwide longitudinal study
Sex- and age-related patterns in the use of analgesics in older patients in the emergency department
Evaluation of environmental assessment tools for settings for individuals living with dementia
Military Injuries-Understanding Posttraumatic Epilepsy, Health, and Quality-of-Life Effects of Caregiving: Protocol for a Longitudinal Mixed Methods Observational Study
Sarcopenia and diabetes-induced dementia risk
Control of hippocampal synaptic plasticity by microglia-dendrite interactions depends on genetic context in mouse models of Alzheimer's disease
Older Workers with Physically Demanding Jobs and their Cognitive Functioning
Impact of subclinical hypothyroidism on cognitive functions among the elderly
CogAx: Early Assessment of Cognitive and Functional Impairment from Accelerometry
Testing a Single-Case Experimental Design to Study Dynamic Light Exposure in People with Dementia Living at Home
Cognitive frailty in relation to vitamin B12 and 25-hydroxyvitamin D in an elderly population: a cross-sectional study from NHANES
A Longitudinal Study of Cognitive and Instrumental Activities of Daily Living Disablement Among the Oldest Mexican Americans
Siamese Graph Convolutional Network quantifies increasing structure-function discrepancy over the cognitive decline continuum
Group-based trajectory modelling for cognitive changes in middle-aged and older adults: A systematic review
Routinization: risk factor or marker of adjustment to negative health issues?
The effect of all-cause hospitalization on cognitive decline in older adults: a longitudinal study using databases of the National Health Insurance Service and the memory clinics of a self-run hospital
12-month trajectory and predictors of affect balance in nursing home residents living with dementia

Characteristics and cognition of older adults engaged in lifelong learning
Evaluation of cognitive function in the Dog Aging Project: associations with baseline canine characteristics
Does cognitive impairment precede self-reported poor hearing? Results from the English longitudinal study of ageing
Cross-scanner harmonization methods for structural MRI may need further work: A comparison study
Assessment of Daily Routine Uniformity in a Smart Home Environment Using Hierarchical Clustering
Development and validation of the dialysis dementia risk score: A retrospective, population-based, nested case-control study
Country-level incidence of Alzheimer disease and related dementias is associated with increased omega-6-PUFA consumption
The role of physical function and physical activity on cognitive function in the elderly
Association between dietary folate intake and cognitive impairment in older US adults: National Health and Nutrition Examination Survey
A new behavioural intervention to enhance memory in older people-evening autobiographical recall
Using MemTrax memory test to screen for post-stroke cognitive impairment after ischemic stroke: a cross-sectional study
A national longitudinal dyadic analysis of spousal education and cognitive decline in the United States*
Mediterranean diet and structural neuroimaging biomarkers of Alzheimer's and cerebrovascular disease: A systematic review
Longitudinal trajectories of physical functioning among Chinese older adults: the role of depressive symptoms, cognitive functioning and subjective memory
Association of Seizure Control with Mortality, Cognition, and Function in People With Dementia
Robust parametric modeling of Alzheimer's disease progression
Individual and environmental factors associated with cognitive function in older people: a longitudinal multilevel analysis
Long-term effect analysis of a cognitive stimulation program in mild cognitive impairment elderly in Primary Care: A randomized controlled trial
Longitudinal amyloid and tau PET imaging in Alzheimer's disease: A systematic review of methodologies and factors affecting quantification
Self-Perceptions of Aging: A Systematic Review of Longitudinal Studies
Research on Individuals Aged One Hundred and Over: Protocol from the Sevilla and Castilla y Leon Centenarian Studies
Factors Associated with Falls in Canadian Long Term Care Homes: a Retrospective Cohort Study
The Relationship Between Cardiovascular Health and Rate of Cognitive Decline in Young-Old and Old-Old Adults: A Population-Based Study
Impact of Polypharmacy on Symptoms and Health Outcomes in Older Adults With and Without Alzheimer's Disease and Related Dementias
Identifying Predictive Risk Factors for Future Cognitive Impairment Among Chinese Older Adults: Longitudinal Prediction Study
Association of cardiovascular diseases with cognitive performance in older adults
The relationship between hand grip strength and cognitive function in middle-aged and elderly people in China: A cross-sectional analysis based on CHARLS data
Examining the Relationships Between Indoor Environmental Quality Parameters Pertaining to Light, Noise, Temperature, and Humidity and the Behavioral and Psychological Symptoms of People Living With Dementia:Scoping Review
The Therapeutic Potential of Yoga for Alzheimer's Disease: A Critical Review
Development of the First WHO Guidelines for Risk Reduction of Cognitive Decline and Dementia: Lessons Learned and Future Directions
Online cognitive monitoring technology for people with Parkinson's disease and REM sleep behavioural disorder
Mediation analysis of leisure activities on the association between cognitive function and mortality: a longitudinal study of 42,942 Chinese adults 65 years and older
Predicting cognitive decline in a low-dimensional representation of brain morphology
The fusion of multi-omics profile and multimodal EEG data contributes to the personalized diagnostic strategy for neurocognitive disorders
A systematic review of urban green and blue spaces and cognitive function including discussion of mechanistic pathways
Modifiable lifestyle factors and lifetime risk of atrial fibrillation: longitudinal data from the Korea NHIS-HealS and UK Biobank cohorts
Cognitive frailty in older adults: examining the impact of frailty criteria on neuropsychological profile, functional outcomes, activity levels, and quality of life
Association Between Healthy Lifestyle and Cognitive Function in Middle-Aged and Older Adults
The impact of COVID-19 restrictions and care home strategies on residents with dementia as experienced by family carers in Italy
Cognitive reserve in individuals with frontotemporal dementia: a systematic review
Mediating role of homebound status between depressive symptoms and cognitive impairment among community-dwelling older adults in the USA: a cross-sectional analysis of a cohort study
The Association Between the Duration, Treatment, Control of Hypertension and Lifestyle Risk Factors in Middle-Aged and Elderly Patients with Mild Cognitive Impairment: A Case-Control Study
Advance Care Planning, End-of-Life Preferences, and Burdensome Care A Pragmatic Cluster Randomized Clinical Trial
Software compatibility analysis for quantitative measures of [18F]flutemetamol amyloid PET burden in mild cognitive impairment
The Association of Service Use and Other Client Factors with the Time to Transition from Home Care to Facility-Based Care
The role of purpose in life in healthy aging: implications for the Semmelweis Study and the Semmelweis-EUniWell Workplace Health Promotion Model Program
Predictors of the rate of cognitive decline in older adults using machine learning
Developing Biomarkers for the Skin: Biomarkers for the Diagnosis and Prediction of Treatment Outcomes of Alzheimer's Disease
Physical Frailty and Cognitive Function among Older Chinese Adults: The Mediating Roles of Activities of Daily Living Limitations and Depression
Depressive Signs and Daily Life of Residents When Relocating from a Regular to an Innovative Nursing Home
Molecular basis of complex relationships between climate change, sleep disorders, and Alzheimer's disease
The relationship between vitamin D deficiency and mortality in older adults before and during COVID-19 pandemic

Types of social networks and starting leisure activities in later life: A longitudinal Japan Gerontological Evaluation Study (JAGES)
Quality of dietary fat and risk of Alzheimer's disease and dementia in adults aged ‚â•50 years: a systematic review
Remembering the past, enhancing the present and sharing the future: a qualitative study of the impact of film screenings in care home communities
Incorporating frailty to address the key challenges to geriatric economic evaluation
Top 20 Research Studies of 2023 for Primary Care Physicians
Anorexia in Medicare Fee-for-Service Beneficiaries: A Claims-Based Analysis of Epidemiology and Mortality
Cognitive impairment and sedentary behavior predict health-related attrition in a prospective longitudinal Parkinson's disease study
Healthy lifestyle and cognitive decline in middle-aged and older adults residing in 14 European countries
Predictors of Changes in Cognitive Function in Older Korean Adults: The 2006-2018 Korean Longitudinal Study of Aging
Influencing factors on instrumental activities of daily living functioning in people with mild cognitive disorder - a secondary investigation of cross-sectional data
Association Between 24-Hour Movement Behavior and Cognitive Function in Brazilian Middle-Aged and Older Adults: Findings From the ELSA-Brasil
Near‚ÄÉInfrared Light‚ÄÉBased Approaches for Brain Functional Imaging and Neuromodulation (Invited)
Longitudinal impact of volunteering on the cognitive functioning of older adults: A secondary analysis from the US Health and Retirement Study
Within-Person Associations Among Self-Perceptions of Memory, Depressive Symptoms, and Activity Participation in Older Adults
Associations of an individual's need for cognition with structural brain damage and cognitive functioning/impairment: cross-sectional population-based study
Association between perceived risk of Alzheimer's disease and related dementias and cognitive function among US older adults
Mastication Influences Human Brain Anatomy
Gait metrics analysis utilizing single-point inertial measurement units: a systematic review br
High lifelong cognitive reserve prolongs disability-free survival: The role of cognitive function
Symptom-led staging for semantic and non-fluent/agrammatic variants of primary progressive aphasia
Classifying and characterizing the development of self-reported overall quality of life among the Chinese elderly: a twelve-year longitudinal study
Sustainable Participation in Community Health Programs to Promote a Healthy Lifestyle and Prevent and Protect against Dementia among Rural Taiwanese Middle-Aged and Older Adults
Association Between Platelet and Cerebral Small Vessel Disease: A Secondary Analysis Based on a Retrospective Cross-Sectional Study in Korean Adults
Multimodal cognitive and behavioral interventions for patients with MCI: a systematic review and meta-analysis on cognition and mood
Association of diet quality score with the risk of mild cognitive impairment in the elderly
Impact of caregiving on mental, self-rated, and physical health: evidence from the China health and retirement longitudinal study
Factors Associated with Denture Non-use in Older Adults Requiring Long-Term Care
Resistance and resilience to Alzheimer's disease in Down syndrome
Age and Marital Status Predict Mild Cognitive Impairment During Acute Coronary Syndrome Admission An Observational Study of Acute Coronary Syndrome Inpatients
Construct Validity, Test-Retest Reliability, Sensitivity to Change, and Feasibility of the Patient-Specific Functional Scale in Acutely Hospitalized Older Patients With and Without Cognitive Impairment
Aging Well and Autism: A Narrative Review and Recommendations for Future Research
Investigating associations between blood metabolites, later life brain imaging measures, and genetic risk for Alzheimer's disease
Social Isolation Changes and Long-Term Outcomes Among Older Adults
Early Predictors for Discharge to Geriatric Rehabilitation after Hip Fracture Treatment of Older Patients
Investigating cognition in midlife
Mediterranean Diet, Alzheimer Disease Biomarkers, and Brain Atrophy in Old Age
A Review of miRNAs as Biomarkers and Effect of Dietary Modulation in Obesity Associated Cognitive Decline and Neurodegenerative Disorders
Determination of Factors Associated With Urinary Incontinence in Older Individuals With Alzheimer's Disease: A Cross-Sectional Study
Machine Learning Model to Predict Diagnosis of Mild Cognitive Impairment by Using Radiomic and Amyloid Brain PET
Neighbourhood walkability and mental health in older adults: A cross-sectional analysis from EpiFloripa Aging Study
Caregiving and changes in health-related behaviour
Eye-tracking indices of impaired encoding of visual short-term memory in familial Alzheimer's disease
Multivariate decomposition of gender differentials in cognitive impairment among older adults in India based on Longitudinal Ageing Study in India, 2017-2018
Favourable Lifestyle Protects Cognitive Function in Older Adults With High Genetic Risk of Obesity: A Prospective Cohort Study
At-admission prediction of mortality and pulmonary embolism in an international cohort of hospitalised patients with COVID-19 using statistical and machine learning methods
Impact of the COVID-19 pandemic on some modifiable risk factors of dementia in an aging, rural Indian population
Acute effects of ambient nitrogen dioxide exposure on serum biomarkers of nervous system damage in healthy older adults
Association between Anemia and Risk of Parkinson Disease
Visuospatial and Verbal Memory Differences in Amish Individuals With Alzheimer Disease and Related Dementias
Multicohort cross-sectional study of cognitive and behavioural digital biomarkers in neurodegeneration: the Living Lab Study protocol
The impact of preventive behaviors on self-rated health, depression symptoms, and daily functioning among middle-aged and elderly Chinese: An empirical study
Machine Learning for Prediction of Cognitive Health in Adults Using Sociodemographic, Neighbourhood Environmental, and Lifestyle Factors

Virtual Reality for Veteran Relaxation: Can VR Therapy Help Veterans Living With Dementia Who Exhibit Responsive Behaviors?
Modifiable risk factor profiles moderate the effect of Œ≤-amyloid pathology on cognition in aging
Osteoporosis and Dementia: Establishing a Link
Association between falls in Alzheimer disease and scores on the Balance Evaluation Systems Test (BESTest) and MiniBESTest
Five-Factor Model Personality Traits and the Trajectory of Episodic Memory: Individual-Participant Meta-Analysis of 471,821 Memory Assessments from 120,640 Participants
Social interaction level modulates the impact of frailty on cognitive decline: a longitudinal study
Association between physical activity and cognitive function in post-menopausal women with high parity: the chain-mediating effects of nutritional status and depression
Differences in Plasma Lactoferrin Concentrations Between Subjects with Normal Cognitive Function and Mild Cognitive Impairment: An Observational Study
Identification of Factors in Moderate-Severe TBI Related to a Functional Decline in Cognition Decades After Injury
Prediction of Mild Cognitive Impairment Status:Pilot Study ofMachine Learning Models Based on Longitudinal Data FromFitness Trackers
Contribution of social and lifestyle factors to cognitive status and 5-year change among middle-aged and older Americans
Plasma p-tau181 and GFAP reflect 7T MR-derived changes in Alzheimer's disease: A longitudinal study of structural and functionalMRI andMRS
Participation in physical, cognitive, and social activities and the association with mobility disability transitions in older adults
The association between vitamin D and BDNF on cognition in older adults in Southern Brazil
Impact of Mediterranean Diet on Chronic Non-Communicable Diseases and Longevity
Hearing impairment and diverse health outcomes An umbrella review of meta-analyses of observational studies
Network propagation of rare variants in Alzheimer's disease reveals tissue-specific hub genes and communities
An examination of the prevalence of metabolic syndrome in older adults in Ireland: Findings from The Irish Longitudinal Study on Ageing (TILDA)
Outdoor green space exposure and brain health measures related to Alzheimer's disease: a rapid review
Predicting cognitive frailty in community-dwelling older adults: a machine learning approach based on multidomain risk factors
Associations between physical activity levels and ATPase inhibitory factor 1 concentrations in older adults
Quality of Life Predictors in a Group of Informal Caregivers during the COVID-19 Pandemic
Disability-Adjusted Life Expectancy and Cognitive Function among Community-Dwelling Adults
The Role of Internet Use and Offline Social Engagement in the Health of Chinese Older Adults: Evidence from Nationally Representative Samples
Association of age-related eye diseases with cognitive frailty in older adults: a population-based study
Factors associated with healthy aging in Latin American populations
Cognitive decline among European retirees: impact of early retirement, nation-related and personal characteristics
Energy expenditure, body composition and dietary habits in progressive supranuclear palsy
Nutrition risk and cognitive performance in community-living older adults without cognitive impairment: a cross-sectional analysis of the Canadian Longitudinal Study on Aging
Sex-Driven Differences in the Effectiveness of Individualized Clinical Management of Alzheimer's Disease Risk
Does tandem balance test predict cognitive impairment among older adults? Findings from Longitudinal Ageing Study in India, 2017-18
The Link Between Physical Function, Œ≤-Amyloid, and Cognitive Aging in Women
Bidirectional Relationships Between Sleep Quality and Cognitive Function in Older Chinese Adults: Observational Analysis of SAWA Trial
Adopting a multidisciplinary telemedicine intervention for fall prevention in Parkinson's disease. Protocol for a longitudinal, randomized clinical trial
Light Treatment for Cognitive Improvement Through Multiple Mechanisms
Relationship between hearing loss and prevalence of depressive symptoms in Japan: Baseline data from the Aidai Cohort study in Yawatahama and Uchiko
Association of Tai Chi and Square Dance with Cognitive Function in Chinese Older Adults
A Multimodal Risk Network Predicts Executive Function Trajectories in Non-demented Aging
Associations between lifestyle and amyotrophic lateral sclerosis stratified by C9orf72 genotype: a longitudinal, population-based, case-control study
Plasma nervonic acid levels were negatively associated with attention levels in community-living older adults in New Zealand
How does urbanization affect the cognitive function among older adults: A geospatial analysis in China
The Relationship Between Subjective Cognitive Decline and Cognitive Leisure Activity Engagement: A Systematic Review
Supporting older people with cognitive impairment during and after hospital stays with intersectoral care management (intersec-CM)-results of a randomised clinical trial
Cognitive and Functional Differences in Aging with and without Intellectual Disabilities: Observational Study
Receptive and participatory arts engagement and subsequent healthy aging: Evidence from the Health and Retirement Study
Poststroke cognitive outcome is better accounted for by white matter abnormalities automated segmentation than visual analysis
Impact of Caregiving on Cognitive Functioning: Evidence From the China Health and Retirement Longitudinal Study
Longitudinal Associations Between Vision Neuropsychiatric, Musculoskeletal, and Cardiometabolic Chronic Diseases
Gait characteristics in community-dwelling older persons with low skeletal muscle mass and low physical performance
The Alusti Test, a Functional Assessment Scale Applicable to the Geriatric Population
Social participation patterns and associations with subsequent cognitive function in older adults with cognitive impairment: a latent class analysis

Comparing relationships between health-related behaviour clustering and episodic memory trajectories in the United States of America and England: a longitudinal study
Examining the directional associations of handgrip strength asymmetry and cognitive function in American males and females
The effect of vitamin D on neurocognitive functions in older vitamin D deficient adults: a pilot longitudinal interventional study
A Pilot randomized clinical trial of adapted tango to improve cognition and psychosocial function in African American women with family history of Alzheimer's disease (ACT trial)
The relationship between healthy lifestyles and cognitive function in Chinese older adults: the mediating effect of depressive symptoms
Cognitive performance in dialysis patients - "when is the right time to test?"
Specific Dimensions of Depression Have Different Associations With Cognitive Decline in Older Adults With Type 2 Diabetes
Preventing Cognitive Decline in Older Latino Adults With HIVThrough a Culturally Tailored Health Promotion Intervention:Protocol for a Single-Arm Pilot Trial
Predictors of caregiver burden in aged caregivers of demented older patients
A Systematic Review of Glucose Transport Alterations in Alzheimer's Disease
No Differences in Urine Bisphenol A Concentrations between Subjects Categorized with Normal Cognitive Function and Mild Cognitive Impairment Based on Montreal Cognitive Assessment Scores
Pain coping strategies and their association with quality of life in people with Parkinson's disease: A cross-sectional study
Contributions of early-life cognitive reserve and late-life leisure activity to successful and pathological cognitive aging
Microvascular dysfunction and neurovascular uncoupling are exacerbated in peripheral artery disease, increasing the risk of cognitive decline in older adults
Relationship Between Mild Cognitive Impairment and Activities of Daily Living in Older Patients in Convalescent Rehabilitation Wards
Genome-wide association analysis of cognitive function in Danish long-lived individuals
Between-Person and Within-Person Associations Among Sensory Functioning and Attitude Toward Own Aging in Old Age: Evidence for Mutual Relations
Association between frailty and cognitive function in older Chinese people: A moderated mediation of social relationships and depressive symptoms
Liver disease-linked metabolic and behavioral factors associated with cognitive performance in an observational study of community dwelling adults
Comparing quality of care outcomes between assisted living and nursing homes before and during the COVID-19 pandemic
Association of vitamin D and cognition in people with type 2 diabetes: a systematic review
Associations between white matter hyperintensities, lacunes, entorhinal cortex thickness, declarative memory and leisure activity in cognitively healthy older adults: A 7-year study
Blood pressure variability and structural brain changes: a systematic review
Development and Validation of the Disability Index Among Older Adults
Gender differences in the correlation between body mass index and cognitive impairment among the community-dwelling oldest-old in China: a cross-sectional study
Gender specific factors contributing to cognitive resilience in APOE e4 positive older adults in a population-based sample
Tipping Points-Do the Prognostic Values of Multimorbidity and Functional Status Vary with Age?
Associations among drinking water quality, dyslipidemia, and cognitive function for older adults in China: evidence from CHARLS
Topography of associations between cardiovascular risk factors and myelin loss in the ageing human brain
Treatment-resistant late-life depression prevalence and clinical/ sociodemographic correlates: An electronic health records study
Prevalence and functional impact of parkinsonian signs in older adults from the Good Aging in Skane study
Fostering an active daily life: An ethnographic study unravelling the mechanisms of Green Care Farms as innovative long-term care environment for people with dementia
The association between family doctor contract services and the health of middle-aged and older people in China: an instrumental variables analysis
Sex and gender differences in cognitive and brain reserve: Implications for Alzheimer's disease in women
A systematic review of the relationship between night shift work and oxidative stress
Glutathione Depletion and Concomitant Elevation of Susceptibility in Patients with Parkinson's Disease: State-of-the-Art MR Spectroscopy and Neuropsychological Study
Exploring Interrelationships between Mental Health Symptoms and Cognitive Impairment in Aging People Living with HIV in China
Risk factors for hospitalization-associated disability among older patients: A systematic review and meta-analysis
Incidence of Parkinson disease in North America
Association Between Central and Peripheral Age-Related Hearing Loss and Different Frailty Phenotypes in an Older Population in Southern Italy
Feasibility Randomized Controlled Trial of a Mind-Body Activity Program for Older Adults With Chronic Pain and Cognitive Decline: The Virtual "Active Brains" Study
Protein Intake and Cognitive Function in Older Adults: A Systematic Review and Meta-Analysis
A change in social participation affects cognitive function in middle-aged and older Chinese adults: analysis of a Chinese longitudinal study on aging (2011-2018)
Interaction effect of midday napping duration and depressive symptoms on subjective memory impairment among older people in China: evidence from the China health and retirement longitudinal study database
Late-life psychiatric factors and life satisfaction are associated with cognitive errors: evidence from an experimental module of a large-scale survey in India
Association of quality of life with structural, functional and molecular brain imaging in community-dwelling older adults
Leisure activities and cognitive impairment in old age: The role of life course socioeconomic status
Long-term risk of cardiovascular disease after traumatic brain injury: screening and prevention
Association between hypothalamic Alzheimer's disease pathology and body mass index: The Hisayama study

Integrating Machine Learning and Environmental and Genetic Risk Factors for the Early Detection of Preclinical Alzheimer's Disease
The impact of sensory impairments and eye diseases on cognitive function in elderly Chinese: The mediating effects of social participation
The effects of olive oil consumption on cognitive performance: a systematic review
Associations between dietary fatty acid patterns and cognitive function in the Hispanic Community Health Study/Study of Latinos
Addressing the disparities in dementia risk, early detection and care in Latino populations: Highlights from the second Latinos & Alzheimer's Symposium
Association between the mediterranean diet and cognitive health among healthy adults: A systematic review and meta-analysis
Association Between Cognitive Status and Falls With and Without Injury During a Skilled Nursing Facility Short Stay
Protocol for a meta-review of interventions to prevent and manage ICU delirium
Genetically proxied lean mass and risk of Alzheimer's disease: mendelian randomisation study
Interaction of greenness and polygenic risk score of Alzheimer's disease on risk of cognitive impairment
Caregiving Needs Are Unmet for Many Older Homeless Adults: Findings from the HOPE HOME Study
Cerebrospinal fluid tau levels are associated with abnormal neuronal plasticity markers in Alzheimer's disease
Geriatric assessment tool application in treatment recommendations for older women with breast cancer
Exploring the design and evaluation of social exergames for older adults: a systematic review
Relationship between occlusal force and psychological frailty in Japanese community-dwelling older adults: The Septuagenarians, Octogenarians, Nonagenarians Investigation with Centenarians study
Joint Association of Combined Healthy Lifestyle Factors and Hearing Loss With Cognitive Impairment in China
Association between self-perception of aging and cognitive function in Chinese older adults: The mediation effect of health behaviors
Prevalence and Predictors of Self-Reported Swallowing Difficulties in Community-Dwelling Older Adults: A Population-Based Study from the National Health and Aging Trends Study (NHATS)
Association between sedentary behavior and risk of cognitive decline or mild cognitive impairment among the elderly: a systematic review and meta-analysis
Non-linear relationship between calf circumference and global cognition in Chinese population: a cross-sectional study of 12,102 Chinese older adults
Polygenic coronary artery disease association with brain atrophy in the cognitively impaired
Cognitive Function and Self-Management Behaviors in Older Adults With Type 2 Diabetes
Early to Midlife Smoking Trajectories and Cognitive Function in Middle-Aged US Adults: the CARDIA Study
A mixed methods pilot randomised controlled trial to develop and evaluate the feasibility of a Mediterranean diet and lifestyle education intervention 'THINK-MED' among people with cognitive impairment
Low testosterone levels relate to poorer cognitive function in women in an APOE-Œµ4-dependant manner
Efficacy of wooden toy training in alleviating cognitive decline in elderly individuals with cognitive impairment: A cluster randomized controlled study
Smaller spared subcortical nuclei are associated with worse post-stroke sensorimotor outcomes in 28 cohorts worldwide
Cognitive impairment in adult CHD survivors: A pilot study
Home-care robots - Attitudes and perceptions among older people, carers and care professionals in Ireland: A questionnaire study
Cognitive impairment in the elderly patient with symptomatic severe aortic stenosis. Therapeutic decisions and impact on one-year mortality
Prevalence of cognitive impairment and its related factors among Chinese older adults: an analysis based on the 2018 CHARLS data
First-Line Immunotherapy with Check-Point Inhibitors: Prospective Assessment of Cognitive Function
Replication and reliability of Parkinson's disease clinical subtypes
The prevalence and determinants of inappropriate sexual behaviour in people with acquired brain injury in nursing homes
Late-midlife lifestyle and brain and cognitive changes in individuals on the AD versus non-AD continuum
Longitudinal associations of reproductive factors and exogeneous estrogens with neuroimaging biomarkers of Alzheimer's disease and cerebrovascular disease
Mediterranean Diet and Ultra-Processed Food Intake in Older Australian Adults-Associations with Frailty and Cardiometabolic Conditions
Longitudinal Impacts of Precision Greenness on Alzheimer's Disease
Green but grey: green areas, air pollution, and children's cognitive development in Santiago, Chile
Cognitive function in men with non-motor features of Parkinson's disease
The Lifelong Health Support 10: a Japanese prescription for a long and healthy life
Dietary patterns and cognitive function in older New Zealand adults: the REACH study
Cognitive decline trajectories and influencing factors in China: A non-normal growth mixture model analysis
Integration of Complementary Medicine in Geriatric Nursing Homes: A Prospective Comparative Observational Study
What role do metals play in Alzheimer's disease?
Cognitive and functional evolution in older adults with and without intellectual disability using a multicomponent intervention: A prospective longitudinal study
Case report: The Circle Game
Association between basal metabolic rate and cognitive function in Chinese older adults: the mediating effect of sarcopenia
Associations between serum lipid levels and cognitive impairment in hypertensive patients: a case-control study
Sex steroids and the female brain across the lifespan: insights into risk of depression and Alzheimer's disease
Hippocampal Neuronal Integrity and Functional Connectivity Within the Default Mode Network in Mild Cognitive Impairment: A Multimodal Investigation
A Novel Way of Measuring Dual-Task Interference: The Reliability and Construct Validity of the Dual-Task Effect Battery in Neurodegenerative Disease

MIND diet and cognitive performance in older adults: a systematic review
Role of Music Therapy Combined with Dyadic Coping in Enhancing Psychosocial Adaptation and Marital Well-being for Young and Middle-aged Patients Returning to Work after Acute Myocardial Infarction
Impairment of the Functional Status and Decrease in Albumin in Frail Older People After a COVID-19 Outbreak: A Descriptive Study in a Long-Term Care Facility in Chile
High Frequency Repetitive Transcranial Magnetic Stimulation Improves Cognitive Performance Parameters in Patients with Alzheimer's Disease - An Exploratory Pilot Study
Lecithin Alleviates Memory Deficits and Muscle Attenuation in Chinese Older Adults and SAMP8 Mice
Survival Bias and Crosstalk between Chronological and Behavioral Age: Age- and Genotype-Sensitivity Tests Define Behavioral Signatures in Middle-Aged, Old, and Long-Lived Mice with Normal and AD-Associated Aging
Self-Report Alzheimer's Disease Statuses in UK Biobank Distort Downstream Analyses
Purpose in life and markers of immunity and inflammation: Testing pathways of episodic memory
Circulating micro-RNAs Differentially Expressed in Korean Alzheimer's Patients With Brain AŒ≤ Accumulation Activate Amyloidogenesis
Patterns of social relationships among community-dwelling older adults in Japan: latent class analysis
Long-term exposure to traffic-related air pollution is associated with impaired odor identification: Results from the population-based KORA FIT study in Augsburg, Germany
Effects of Lifetime Exposure to Sports-Related Head Impacts on Brain Injury and Inflammatory Blood Biomarkers Among Former Middle-Aged Athletes
Leukocyte telomere length and memory circuitry and cognition in early aging: Impact of sex and menopausal status
Genome-Wide Association Study of Alzheimer's Disease Brain Imaging Biomarkers and Neuropsychological Phenotypes in the European Medical Information Framework for Alzheimer's Disease Multimodal Biomarker Discovery Dataset
Repeated Sub-Concussive Impacts and the Negative Effects of Contact Sports on Cognition and Brain Integrity
Vitamin D supplementation and cognition-Results from analyses of the D-Health trial
Proteomic Profiling of Plasma Biomarkers Associated With Return to Sport Following Concussion: Findings From the NCAA and Department of Defense CARE Consortium
Alzheimer's disease heterogeneity revealed by neuroanatomical normative modeling
Tau follows principal axes of functional and structural brain organization in Alzheimer's disease
Safety, Tolerability, Pharmacokinetics and Quantitative Electroencephalography Assessment of ACD856, a Novel Positive Allosteric Modulator of Trk-Receptors Following Multiple Doses in Healthy Subjects
A metabolomic signature of the APOE2 allele
Walking pace, handgrip strength, age, APOE genotypes, and new-onset dementia: the UK Biobank prospective cohort study
The relationship between leisure time physical activity patterns, Alzheimer's disease markers and cognition
NMR metabolomic modeling of age and lifespan: A multicohort analysis
Assessing modifiable risk factors for dementia in the Czech Republic: findings from the Survey of Health, Ageing and Retirement in Europe study
Sex Differences in Dementia
The impact of estimated cardiorespiratory fitness on Alzheimer's disease biomarkers and their relationships with cognitive decline
Association between cognitive diagnosis and a range of significant life events in an elderly essential tremor cohort: a longitudinal, prospective analysis
TWINGEN: protocol for an observational clinical biobank recall and biomarker cohort study to identify Finnish individuals with high risk of Alzheimer's disease
Effect of gamification with a support partner to increase physical activity in older adults at risk for Alzheimer's disease: The STEP 4Life randomized clinical trial
Uncemented hemiarthroplasty may have a role in the treatment of unstable intertrochanteric fractures in elderly patient. A survival complications and functional outcomes analysis
Global consensus on optimal exercise recommendations for enhancing healthy longevity in older adults (ICFSR).
Structured vs Self-Guided Multidomain Lifestyle Interventions for Global Cognitive Function: The US POINTER Randomized Clinical Trial.
Individual and additive effects of vitamin D, omega-3 and exercise on DNA methylation clocks of biological aging in older adults from the DO-HEALTH trial.
Public attitudes toward dementia risk prediction: A mixed-methods study.
Accelerometer-measured physical activity timing with incident dementia.
Physical Activity, Alzheimer Plasma Biomarkers, and Cognition.
Active Travel Mode and Incident Dementia and Brain Structure.
Longitudinal Blood-Based Biomarkers and Clinical Progression in Subjective Cognitive Decline.
Physical activity as a modifiable risk factor in preclinical Alzheimer's disease.
Acute effects of lactate infusion on metabolism, AD biomarkers, and cognition: The LEAN study.
Cognitive Decline and Dementia in Chronic Widespread Pain: A Longitudinal Population-based Study.
Associations between night/shift working and late-life brain health.
Exploring causal gut-brain axes in Alzheimer's disease using mediation Mendelian randomization analysis.
Integrated Multi-Omics Analysis and Cross-Model Validation Reveal Mitochondrial Signatures in Alzheimer's Disease.
Physical activity changes during midlife link to brain integrity and amyloid burden.
Association of Frailty With Dementia and the Mediating Role of Brain Structure and Immunometabolic Signatures.
Longitudinal Association Between Multimorbidity, Participating Activity, and Cognitive Function: Cross-lagged Mediation Analysis.
Association of Alzheimer's Disease Blood Biomarkers With Sarcopenia Incidence and Progression: A 12-Year Population-Based Study.

Guidelines for the standardization of pre-analytical variables for salivary biomarker studies in Alzheimer's disease research: An updated review and consensus of the Salivary Biomarkers for Dementia Research Working Group.
Associations of handgrip strength, walking times, and dementia among Chinese adults: A cross-sectional analysis from CHARLS.
Neuroimaging correlates and biomarker performance of a fully automated plasma p-tau217/AŒ≤42 ratio assay in a clinical cohort with Alzheimer's disease.
Interplay between age, APOE Δê4 and the metabolome in plasma and brain in Alzheimer's disease.
Serum ergothioneine and risk of dementia in a general older Japanese population: the Hisayama Study.
Periodontal disease and brain amyloid pathology in mild cognitive impairment.
Genetic background and multidomain interventions in mild cognitive impairment.
Accelerometer-derived "weekend warrior" physical activity, sedentary behavior, and risk of dementia.
Intrinsic Capacity Defined Using 4 Domains, Genetic Risk, and Incident Parkinson Disease: A Prospective Cohort Study.
Brain patterns and risk factors in the FINGER RCT multimodal lifestyle intervention.
Recent advances in the detection and management of motor dysfunction in Alzheimer's disease.
Moderate-to-Vigorous Physical Activity at any Dose Reduces All-Cause Dementia Risk Regardless of Frailty Status.
Correlation Between Irisin and Cognitive Functions in Alzheimer Dementia.
Longitudinal Proteomic Profiling of Cognition across an Aerobic Exercise Intervention.
Integrative machine learning approach to risk prediction for dementia and Alzheimer's disease.
Causal cardiovascular risk factors for dementia: insights from observational and genetic studies.
Polyphenol Intake and Risk of Dementia and Cognitive Decline in Older Adults: Principal Component Analysis Approach.
Hearing loss, diet, and cognitive decline: interconnections for dementia prevention.
Associations between white matter hyperintensities and physical activity: A sectional study in UK Biobank participants.
Do exercise interventions improve functional mobility and balance in Alzheimer's patients? A systematic review.
Longitudinal investigation of gait and Alzheimer's disease in adults with Down syndrome.
Progress on exercise therapy in type 2 diabetes mellitus with cognitive impairment.
Stroke-heart syndrome and risk of incident dementia among patients with first-ever ischemic stroke: A territory-wide population-based cohort study.
Long-term cumulative physical activity associated with less cognitive decline: Evidence from a 16-year cohort study.
Association of Physical Exercise With Structural Brain Changes and Cognitive Decline in Patients With Early Parkinson Disease.
Migraine and the Risk of Dementia in the General Population.
Prevalence and co-occurrence of dementia risk factors in Denmark: A nationwide study.
Machine learning and data-driven inverse modeling of metabolomics unveil key processes of active aging.
Relationship of Alzheimer's Disease and Related Dementias Plasma Biomarkers With Mobility in Cognitively Unimpaired Older Adults.
Combined mind-body physical exercise, cognitive training, and nurse-led risk factor modification to enhance cognition among older adults with mild cognitive impairment in primary care: a¬†three-arm randomised controlled trial.
Geriatric Cognitive Decline and Polypharmacy.
Prioritizing Modifiable Risk Factors for Dementia Prevention across the Spectrum of Genetic Susceptibility: A Prospective Cohort Study.
Longitudinal relationships between daily activity and hippocampal atrophy in Japanese dwellers.
Association of cardiorespiratory fitness with dementia risk across different levels of genetic predisposition: a large community-based longitudinal study.
Associations between moderate-to-vigorous physical activity, p-tau181, and cognition in healthy older adults with memory complaints: a secondary analysis from the MAPT.
Potentially modifiable risk factors for dementia in Norway (HUNT4 70+): a retrospective cohort study.
The Association of Apathy With Incident Dementia: A Multiple Mediation Analysis of Cardiovascular Risk Factors.
Impact of Alzheimer's disease on sleep in adults with Down syndrome.
Resistance exercise to reduce risk of falls in people with Alzheimer's disease: a randomised clinical trial.
Daily-life walking speed, running duration and bedtime from wrist-worn sensors predict incident dementia: A watch walk - UK biobank study.
Dementia Risk and Machine Learning-Derived Brain Age Index from Sleep Electroencephalography: A Pooled Cohort Analysis of Over 7,000 Individuals Across Five Community Cohorts.
Adherence to four dietary indices and the risk of all-cause and cause-specific dementia: Findings from the UK Biobank study.
The association between lifestyle factors and mortality in Huntington's disease.
The effect of resistance training for older adults with cognitive frailty: a randomized controlled trial.
Cognitive frailty and functional disability in older adults: A 10-year prospective cohort study in Japan.
Effect of happiness and positive emotions on dementia.
Green tea consumption and dementia risk in community-dwelling Japanese people aged 40-74 years: A 12-year cohort study.
Mediators of educational differences in dementia risk later in life: evidence from the HUNT study.
Transethnic analysis identifies SORL1 variants and haplotypes protective against Alzheimer's disease.
Cerebrospinal fluid neuronal pentraxin levels are associated with tau pathology via microglia-astrocyte signaling in alzheimer's disease.
The overlooked trio: sleep duration, sampling time and physical exercise alter levels of olink-assessed blood biomarkers of cardiovascular risk.
Longitudinal associations between self-reported exercise levels and cognition in ADAD.

Association of sarcopenic obesity with dementia risk in a cohort of older women.
Directionality between cognitive function and daily physical activity patterns.
Older Adults with Diabetes in Korea: Latest Clinical and Epidemiologic Trends.
Western Diet and Cognitive Decline: A Hungarian Perspective-Implications for the Design of the Semmelweis Study.
Accelerometry-derived features of physical activity, sleep and circadian rhythm relate to non-motor symptoms in individuals with isolated REM sleep behavior disorder.
Gait speed, handgrip strength, and their combination, and risk of depression in later life: Evidence from a prospective study of community-dwelling older adults.
Life satisfaction and risk of dementia in older adults: findings from 15 European countries and meta-analysis.
Longitudinal study of body mass index in relation to Alzheimer's disease pathology and symptomatology in Down syndrome.
Middle-age cerebral small vessel disease and cognitive function in later life: a population-based prospective cohort study.
DNA methylation signatures of Life's Essential 8 and their implications for dementia.
Association of accelerometer-measured step count and intensity with mental health: A prospective cohort study.
Effects of multidomain lifestyle intervention on frailty among older men and women - a secondary analysis of a randomized clinical trial.
Sex-specific decline in prefrontal cortex mitochondrial bioenergetics in aging baboons correlates with walking speed.
Identifying the optimal combinations of modifiable dementia risk factors to target in multidomain intervention - Three-year longitudinal findings from the Canadian longitudinal study on aging.
Longitudinal Engagement in Modifiable Lifestyle Behaviors and Racial-Ethnic Differences in Dementia Risk.
Apathy in dementia: pharmacological and nonpharmacological treatment strategies.
Dementia risk reduction in China: Country-specific estimates of modifiable risk factors and population attributable fractions (PAFs).
Aerobic exercise facilitates p300 nuclear translocation via ADRB2-AMPKŒ± signaling, leading to enhanced histone acetylation and mitigation of cognitive decline in APP/PS1 mice.
Intervention Effects of the Holistic Physio-Cognitive Rehabilitation for Alzheimer Disease and Mild Cognitive Impairment.
The biomarker and clinical changes across the Alzheimer's continuum study (BCAS): rationale, design, and baseline characteristics of the first 1,013 participants.
Predicting the onset of dementia with longitudinal performance-based measures of physical function.
Cardiovascular Risk Factors, Alzheimer's Disease, and the MIND Diet: A Narrative Review from Molecular Mechanisms to Clinical Outcomes.
Twelve-year nationwide cohort study identifying risk factors for conversion from mild cognitive impairment to Alzheimer's disease.
Chronotype as a potential risk factor for cognitive decline: The mediating role of sleep quality and health behaviours in a 10-year follow-up study.
Effectiveness of Walking Prescription Using Mobile Health Technology on the Changes in Daily Steps in Older Adults With Cognitive Impairment: Randomized Controlled Study.
Regular physical activity affects brain activities in old individuals: an observational study.
The role of APOE Œµ4 in modulating the relationship between non-genetic risk factors and dementia: a system review and meta-analysis.
Effects of maximal speed locomotor training on spatiotemporal gait changes in individuals with chronic stroke: A secondary analysis of a randomized controlled trial.
The Effects of a Multidomain Lifestyle Intervention on Brain Function and Its Relation With Immunometabolic Markers and Intestinal Health in Older Adults at Risk of Cognitive Decline: Study Design and Baseline Characteristics of the HELI Randomized Controlled Trial.
Incident dementia in ischaemic stroke patients with early cardiac complications: A propensity-score matched cohort study.
Physical and mental demands of work associated with dementia risk in later life.
Risk factors for early-onset and late-onset dementia: a prospective cohort study.
Impact of lifestyle factors on quantitative motor and cognitive performance: insights from a longitudinal study on healthy ageing.
Associations of proteomic age with mortality and incident chronic diseases in the European Prospective Investigation into Cancer and Nutrition (EPIC).
The Anti-Inflammatory Actions of Soluble Klotho in Brain Aging and Its Main Associated Diseases.
Prevalence, incidence and modifiable factors for subtypes of mild cognitive impairment: results from the Longitudinal Ageing Study in China.
How physical activity influences motoric cognitive risk syndrome in older adults.
Fall risk stratification in older adults: low and not-at-risk status still associated with falls and injuries.
Longitudinal Trends in Physical Activity Among Older Adults With and Without HIV in Uganda.
Minimal amount of exercise reduces incident dementia in adults with cerebrovascular disease.
Long-term proton pump inhibitor use and risk of dementia: a focused review on pantoprazole.
Elecsys CSF AD immunoassays: Sample stability for a new pre-analytical protocol for fresh CSF.
Walking Aids and Locomotion Training in the Emergency Department: A Randomized Clinical Trial.
Association of Self-Reported and Accelerometer-Measured Physical Activity With Cognitive Performance in the ELSA-Brasil Study.
Biomarkers for Alzheimer's disease across diverse biological domains: an umbrella review and evidence map.
The effect of tablet-based multimodal training on cognitive functioning in Alzheimer's disease: A randomized controlled trial.
Proton pump inhibitors in systemic sclerosis: should we exercise caution? Insights from a large-scale data analysis.
Chronic pain in mental disorders: An umbrella review of the prevalence, risk factors, and treatments across 957,168 people with mental disorders and 16,606,910 controls.
The Influence of Music on Mental Health Through Neuroplasticity: Mechanisms, Clinical Implications, and Contextual Perspectives.
Association between pet ownership and incident dementia: A systematic review.

Dose-Response Association of Handgrip Strength With Alzheimer's Disease: A Longitudinal Study Involving 85,979 Adults.
The Telephysiotherapy for Older People (TOP-UP) program for improving mobility in people receiving aged care: a hybrid type 1 effectiveness-implementation randomised controlled trial.
Moving beyond the prevalent exposure design for causal inference in dementia research.
Physical Activity Moderates the Relationship between Cardiovascular Disease Risk Burden and Cognition in Older Adults.
Trajectories of frailty, grip strength and gait speed preceding dementia: a nested case-control study.
Dose- and pattern- physical activity is associated with lower risk of dementia.
Thirty-seven-year trends in the prevalence, incidence, and prognosis of dementia in a Japanese community: the Hisayama study.
Chronic Aerobic Exercise Alleviates Amyloid-induced Capillary Dysfunction.
Daily physical activity and trajectories of care service use among older adults: the HUNT4 Trondheim 70+ study.
Exercise Interventions Involving Minimal Equipment for Community-Dwelling Adults With Dementia: A Systematic Review and Meta-analysis.
Voluntary Swimming Reduces Amyloid Pathology in an Alzheimer's Mouse Brain: An Integrated Amyloid PET/CT and Light-Sheet Microscopy Evaluation.
Two Decades of the Walking While Talking Test: A Narrative Review.
Wearable Sensor Technologies and Gait Analysis for Early Detection of Dementia: Trends and Future Directions.
A Mobile App-Based Individualized Nonpharmacological Intervention for Behavioral and Psychological Symptoms in Dementia: Pilot Randomized Controlled Trial.
Population attributable fractions of modifiable dementia risk factors for cognitive impairment-the ELSA-Brasil cohort study.
Neuroimaging Correlates of 3 Distinct Physical-Cognitive Phenotypes in Cognitively Normal Older Adults: The Gothenburg H70 Cohort Study.
Modulating glymphatic clearance in Alzheimer's disease: Molecular mechanisms, imaging biomarkers, and emerging interventions.
Replacement of Accelerometer-Measured Sedentary Behavior With Physical Activity Reduces Dementia Risk.
Association of motoric cognitive risk syndrome with falls in older adults: findings from three longitudinal studies.
The cost-effectiveness of an online intervention to prevent dementia: Results from the Maintain Your Brain (MYB) randomised controlled trial.
Amyloid pathology and modifiable risk factors in cognitive decline among cognitively unimpaired older adults.
A physically and mentally active lifestyle relates to younger brain and cognitive age.
Construction of a classification model for dementia among Brazilian adults aged 50 and over.
Association of Cardiovascular-Kidney-Metabolic Health With Structural Brain Imaging and the Risk of Specific-Cause Dementia: A Cohort Study.
Lifetime walking and Alzheimer's pathology: A longitudinal study in older adults.
Impaired Windkessel function and proximal aortic stiffness: Linking vascular ageing to cognitive decline.
Making time for brain health: recognising temporal inequity in dementia risk reduction.
Trajectories of muscle strength and physical performance preceding dementia in older US and European populations.
Evaluating the Accuracy and Reliability of Real-World Digital Mobility Outcomes in Older Adults After Hip Fracture: Cross-Sectional Observational Study.
High circulating adropin levels predict long-term blood pressure variability in old adults: evidence from the Multidomain Alzheimer Prevention Trial.
AI and Wearables for Early Detection of Cognitive Impairment and Dementia: Systematic Review.
Protocol of a randomized, single-blind, controlled trial of an 18-month, home-based, high-intensity, exercise intervention in older, cognitively unimpaired APOE Œµ4 carriers.
Forecasting long-term care demands for dementia in China (2020-2040): A Markov model analysis of urban-rural disparities.
Comparisons of four cognitive-frailty measures in predicting dementia and disability.
Are sleep disturbances modifiable risk factors for mild cognitive impairment and dementia? A systematic review of large studies.
Midlife financial stress and cognitive and physical impairments in older age: The role of potentially modifying factors.
Preoperative identification of low-risk patients for major complications after acute hip fracture surgery in geriatric patients: an observational cohort study.
Cognitive Impairment Highly Prevalent and Associated With Reduced Physical Function in Older Veteran Clinical Exercise Program.
Recommendations from the European interdisciplinary council on ageing on physical activity and diet for mental health conditions in older adults.
Neurologic dance training and home exercise improve motor-cognitive dual-task function similarly, but through potentially different mechanisms, among breast cancer survivors with chemotherapy-induced neuropathy: Initial results of a randomized, controlled clinical trial.
Healthy lifestyle mitigates the adverse association of particulate matter with cognitive function: a 10-year longitudinal analysis of older adults in China.
Behavioral and psychological symptoms of dementia and Alzheimer's disease progression in Down syndrome.
Sarcopenia association with physical and psychological indices in community-dwelling aged population, Birjand Longitudinal Aging Study (BLAS).
Prevalence of dementia risk factors in the Oxford Brain Health Clinic.
Targeting brain health in subjective cognitive decline: insights from a multidomain randomized controlled trial.
Trajectory of Cardiometabolic Disease-Related Hospitalizations Before and After Dementia Diagnosis: A Matched Cohort Study.
Bidirectional impact of dementia and aspiration pneumonia on cognitive decline in older adults: A retrospective cohort analysis.
Chronic physical activity and the prevention of Alzheimer's disease.
Subcutaneous application of Helleborus niger 12x in older patients with dementia-a retrospective cohort study.
Longitudinal, Bidirectional Association between Gait Speed and Cognitive Function in Community-Dwelling Older Adults without Dementia.
Plasma and MRI biomarkers capture neuronal damage in former professional boxers.
Sociodemographic Factors in Older Adults' Gait Speed Decline: A Gender Disaggregate Growth Curve Analysis of the Ibadan Longitudinal Study of Ageing.

Associations Between Walking Pace, APOE-Œµ4 Genotype, and Brain Health in Middle-Aged to Older Adults.
Association of Circulating Vitamin D in Midlife With Increased Tau-PET Burden in Dementia-Free Adults.
Cardiovascular Risk as a Moderator of the Relationship Between Plasma Alzheimer Disease Biomarkers and Cognitive Status.
Lifestyle for Brain health (LIBRA) score, cognition, brain volume, and dementia risk in the UK Biobank.
Association between marital status and dementia among older adults population: the Chinese longitudinal healthy longevity survey.
Streamlined resource-efficient plasma amyloid-beta mass spectrometry assay has improved biomarker performance in preclinical Alzheimer's disease.
Associations between white matter lesions, adiposity, and systemic inflammation in late adulthood: Results from the IGNITE study.
Healthy Cognitive Aging Through Movement: A Practical Approach of Light-Intensity Aerobic Dance for Older Adults.
Tongue strength and cognitive decline in older adults: A scoping review.
Mediation of modifiable risk factors in two multidomain dementia prevention trials.
Care for older adults living with dementia in the emergency department: a systematic review and meta-synthesis of care partner roles and perspectives.
Objectively Measured Physical Activity Using Wrist-Worn Accelerometers as a Predictor of Incident Alzheimer's Disease in the UK Biobank.
The efficacy of reminiscence therapy on the cognition of older patients with cognitive impairment or dementia: a meta-analysis based on regulatory factors.
Minimum physical activities protective against Alzheimer's disease in late life: a systematic review.
Comparing the incidence rates of dementia: Association of social participation among older adults in Japan.
A feasibility study on the efficacy of a VR-based mindfulness intervention for dementia caregivers in the home environment: A pilot randomized control trial protocol.
Impact of frailty status on the effect of a multidomain lifestyle intervention on cognition.
The Temporal Relation of Physical Function with Cognition and the Influence of Brain Health in the Oldest-Old.
Role of light walking pace on cognition: Findings from the Maine-Syracuse Longitudinal Study.
Trajectories of Cardiorespiratory Fitness Measured by Metabolic Equivalents and the Risk of Alzheimer's and Related Dementias.
Associations of Mid- and Late-Life Physical Activities With New-Onset Depression and Anxiety Among Older Adults.
A rapid scoping review on operationalizing cognitive and social activities in research on dementia risk reduction.
Long-Term Exposure to Non-Steroidal Anti-Inflammatory Medication in Relation to Dementia Risk.
Immediate and Long-Term Effectiveness of a Therapeutic Exercise Protocol in Patients with Dementia.
Association Between Physical Activity During Active Commuting and Leisure Time With Cognitive Decline: Findings of the ELSA-Brasil Study.
Meta-Analysis of Transcriptomic Studies of Blood and Six Brain Regions Identifies a Consensus of 15 Cross-Tissue Mechanisms in Alzheimer's Disease and Suggests an Origin of Cross-Study Heterogeneity.
Ambient Air Pollution, Cognitive Impairment, and Dementia Among Older Patients Being Evaluated for Kidney Transplantation.
Midlife as the Critical Window for Women's Stroke and Dementia Prevention: Pivotal Advances and Implementation Priorities.
Joint Associations of Physical Activity and Sleep Quality With Cognitive Function Among Older Adults in China.
Menopausal hormone therapy is associated with worse levels of Alzheimer's disease biomarkers in APOE Œµ4-carrying women: An observational study.
Association of genetic predisposition to dyslipidemia and physical activity with incident dementia.
Associations of Muscle-Strengthening Activity and Cognitive Function in Community-Dwelling Middle-Aged and Older Adults.
Can vigorous physical activity mitigate the effect of systemic inflammation on cognitive performance? Results from a large older community dwelling population in The Netherlands.
Relationship between Physical Function at Admission and Walking Ability at Discharge in Older Adults with Vertebral Compression Fractures: An Analysis Using Propensity Score Matching.
Impact of Mouthwash-Induced Oral Microbiome Disruption on Alzheimer's Disease Risk: A Perspective Review.
Associations of plasma Neurofilament light chain with sarcopenia and physical capacity in patients with Alzheimer's diseases.
Predictors of Mortality in a Large Cohort of Elders with Essential Tremor.
Enhancing dementia prediction: A 19-year validation of the CAIDE risk score with insulin resistance and APOE Œµ4 integration in a population-based cohort.
Association between housework frequency and cognitive impairment among Chinese older adults: a prospective cohort study based on CLHLS.
Decreased Physical Activity as an Early Digital Biomarker in Huntington's Disease: A One-Year Observational Study.
Physical activity and Alzheimer's disease risk across genetic susceptibility: a prospective UK Biobank study using accelerometer data.
Maintaining level of modifiable dementia risk scores is associated with better cognitive outcomes than increasing risk scores: A population-based prospective cohort study.
Long-term changes in cardiorespiratory fitness and incidence of Alzheimer's disease and related dementias among US Veterans.
A longitudinal study of frailty reversibility through a multi-component dementia prevention program.
Insulin resistance as a mediator of physical activity's effects on beta-amyloid accumulation and tau phosphorylation: A scoping review.
Rationale and protocol of the LEAD 2.0 study: a randomized controlled trial assessing the feasibility of a virtually delivered 6-month exercise and nutrition intervention in older adults with subjective cognitive decline (SCD).
Post-cardiovascular disease healthy lifestyle, inflammation and metabolic biomarkers, and risk of dementia: a population-based longitudinal study.
Ultra-processed food consumption and risk of dementia and Alzheimer's disease: The Framingham Heart Study.
Smaller cingulate grey matter mediates the association between dual-task gait and incident dementia.
Association of open skill exercise and long-chain polyunsaturated fatty acid intake with brain volume changes among older community-dwelling Japanese individuals.

Trends and forecasts of the prevalence and mortality of Alzheimer's disease and other dementias in China.
Sleep and physical activity trade-offs and dementia risk: a prospective cohort study in UK Biobank participants.
Physical activity may protect myelin via modulation of high-density lipoprotein.
Heart rate response and recovery during exercise and dementia risk: a prospective UK biobank study.
Association of oral frailty with falls in long-term care residents.
Association of Alzheimer's Disease Polygenic Risk Score with Concussion Severity and Recovery Metrics.
A 6-Month Evaluation of the Peer-Ceived Momentary Assessment Method in a Small Sample of Liver Transplant Patients and Their Support Persons: Longitudinal Observational Study.
Dementia Moves: protocol for a feasibility study testing a physical rehabilitation program for long-term care residents with moderate to severe dementia.
Interdaily variability of rest-activity patterns and risk of dementia in older adults: The ARIC Neurocognitive Study.
Methods for measuring interpersonal behavioral and neural synchrony during group music therapy for individuals with dementia and their caregivers: A case series study.
Associations between physical activity and brain structure in a large community cohort.
Physical activity and the risk of dementia in end-stage renal disease patients undergoing hemodialysis: a nationwide population-based study.
Outdoor physical activity, residential green spaces and the risk of dementia in the UK Biobank cohort.
Physical Activity Over the Adult Life Course and Risk of Dementia in the Framingham Heart Study.
Which Type of Group Activity has the Greatest Impact on the Well-Being of Persons With Dementia? An Exploration of a Study of Enhanced Group Activity Kits.
Magnetic resonance spectroscopy as a non-invasive tool for assessing brain and muscle adaptation to exercise training in older age: a scoping review into existing research.
Number of children and dementia risk: a causal mediation analysis using data from the HUNT study linked with national registries in Norway.
mHealth Intervention for Dementia Prevention through lifestyle Optimisation (MIND-PRO) in a primary care setting: protocol for a randomised controlled trial in people with low SES and/or migration background.
Optimizing treatment of cardiovascular risk factors in cerebral small vessel disease using genetics.
Sleep efficiency in community-dwelling persons living with dementia: exploratory analysis using machine learning.
A Program for Reinforcing Lifestyle Change Motivation and Lifestyle Behavior to Prevent Dementia in Community-Dwelling Middle-Aged and Older Adults: Applying the Health Belief Model.
Neuropathology-based approach reveals novel Alzheimer's Disease genes and highlights female-specific pathways and causal links to disrupted lipid metabolism: insights into a vicious cycle.
Concurrent associations between visit-to-visit changes in actigraphy-based physical activity and cognitive aging in older adults.
Alzheimer's Disease Sequencing Project release 4 whole genome sequencing dataset.
Biomarkers of cellular senescence predict risk of mild cognitive impairment: Results from the lifestyle interventions for elders (LIFE) study.
The COVID-19 pandemic's effects on cognition and 24-hour movement behaviours: Findings from the CLSA.
Cardiorespiratory fitness and risk of dementia: a longitudinal cohort study.
Young-onset dementia: Investigating timelines of admission to aged residential care and health outcomes.
Impact of an exercise and nutrition program on caregiver time with residents in institutional care-A secondary analysis.
The pain identification and communication toolkit: a training program to support family caregivers of persons with ADRD.
Implementing innovative technology promoting self-awareness of brain health and self-determination in obtaining a timely dementia diagnosis: protocol for a multimethods, concurrent, two-part observational study.
Protocol for an intergenerational randomized controlled trial to enhance physical activity in older adults at risk for Alzheimer's disease.
Modifiable risk factors for early- and late-onset dementia using the Korean national health insurance service database.
Association among cardiorespiratory fitness, plasma biomarkers of pathology and astrocyte reactivity, and cognition in autosomal-dominant Alzheimer's disease.
Implementing brain health strategies in primary care: methods and baseline findings from the BHAM registry.
Impaired sleep, depressive symptoms, and pain as determinants of physical activity and exercise intervention adherence: an exploratory analysis of a randomized clinical trial.
Development and evaluation of a free e-learning program on dementia risk reduction for the general public: A pre-post study.
Integration of genetic risk, cardiometabolic diseases, and cognitive impairment among African American adults.
Biobehavioral and Interpersonal Predictors of Cognitive Impairment in Older Adults With Type 2 Diabetes: A Systematic Review of Cohort Studies.
Mortality following hip fracture surgery in patients with dementia: a Swedish multiple national register study.
Smart ring-based assessment of physical activity intensity and sleep disturbances in older adults with mild cognitive impairment.
Lifestyle, sleep quality, and cognitive function in resistant hypertension: One-year follow-up from the TRIUMPH trial.
Dietary magnesium intake and dementia risk in community-dwelling people aged 40-74 years: an 8-year cohort study.
Prevalence of Pathogenic Variants and Eligibility Criteria for Genetic Testing in Patients Who Visit a Memory Clinic.
Metabolic benefits of therapeutic exercise in older adults with type 2 diabetes mellitus and multimorbidity: a retrospective cohort study.
Cognitive remediation for patients with late-life schizophrenia: A follow-up pilot study.
Psychological resilience trajectories after the COVID-19 pandemic: the role of lifestyle and psychosocial factors in a cohort at increased alzheimer's disease risk.
A dose-response meta-analysis of physical activity and the risk of alzheimer's disease in prospective studies.
Associations of modifiable dementia risk factors with dementia and cognitive decline: evidence from three prospective cohorts.

Preserved Thermoregulation in Huntington's Disease: Insights from an Observational Case-Control Study.
Integrating traditional Chinese Medicine reduced the risk of hip fracture in patients with dementia.
Longitudinal deterioration of subjective cognitive decline in apolipoprotein Œµ4 carriers and improvement of subjective cognitive decline by multi-domain intervention for prevention of dementia: The cognitive function instrument assessment.
The association between midlife neighbourhood walkability and Alzheimer's disease in women: a prospective nested case-control study.
Modified Cued Recall Test: Longitudinal Analysis of Test Versions and Item Recall in Adults With Down Syndrome.
The Relationships between physical activity, sedentary behaviour, sleep, and dementia: A systematic review and meta-analysis of cohort studies.
White matter integrity and motor function: a link between cerebral myelination and longitudinal changes in gait speed in aging.
Protocol for a single-arm, multi-component behavior change technique (BCT) intervention to develop a walking habit among caregivers for persons with Alzheimer disease and related dementias (ADRD).
Self-reported motoric cognitive risk syndrome predicts long-term mortality in older adults.
Non-pharmacological interventions for sleep disorders in patients with Alzheimer's disease: An integrative review.
One-year exercise improves cognition and fitness and decreases vascular stiffness and reactivity to CO2 in amnestic mild cognitive impairment.
Terrapino: a mobile application for Alzheimer's risk assessment and cognitive health promotion.
High Physical Fitness Is Associated With Better Cognitive Function in Older Adults With Dementia: A Cross-Sectional Study.
Exploring experiences of times without care and encounters in dementia: Findings from a living evidence map.
Predicting mortality risk in Alzheimer's disease using machine learning based on lifestyle and physical activity.
Salivary Antimicrobial Peptide in Patients With Dementia Before and After Clinical Oral Rehabilitation Programme: A Randomised Controlled Trial.
Relationship Between Cognitive Ability and Phase Angle in Older Adults with Cardiovascular Disease: A Cross-Sectional Study.
Physical Function, Life Expectancy, and Cognitive Impairment in Older Adults: A 12-Year Longitudinal Cohort Study.
OAS1: A Protective Mechanism for Alzheimer's Disease? An Exploration of Data and Possible Mechanisms.
Physical activity complexity, cognition, and risk of cognitive impairment and dementia in the Baltimore Longitudinal Study of Aging.
Knowledge and practice of healthy behaviors for dementia and stroke prevention in a United States cohort.
Mapping dementia research in Indonesia: A scoping review of evidence, gaps, and future directions.
"I Aim to Fulfill My Promise": Dementia Caregiving from the Perspective of Spouses and Partners.
Sustained Improvement of Cognition, Mood and Plasma Markers Three Years After Metabolic Bariatric Surgery. The BARICO Study.
Variation of the Risk Associated With Potentially Modifiable Risk Factors for Dementia Between Ethnic Groups Within One Country. A Retrospective Cohort Study Using Routinely Collected Health Data in Aotearoa New Zealand.
Predicting cognitive decline from neuropsychiatric symptoms and Alzheimer's disease biomarkers: A machine learning approach to a population-based data.
Is the 10-Year Trajectory of Physical Activity Associated With the Incidence of Mild Cognitive Impairment in Older Adults?
Physical activity, muscle strength, sedentary behavior, sleep, and genetic risk of stroke and dementia: findings from a large cohort study.
Testing the iMplementation Framework fOr behavioral and LIfestyLe interventions in AlZheimer's DiseasE (MOBILIZE) via the ACT randomized controlled trial.
A two-year longitudinal study of the impact of cognitive status and depression on frailty status in older adults following hip fracture.
Combined effects of physical activity, cognitive activity, and dietary patterns on cognitive impairment in older adults with consideration of APOE genotype: a cohort study.
Physical activity may mitigate sleep-related cognitive deficits in older adults: Findings from the IGNITE study.
Clinical trial characteristics and intervention profiles for mild cognitive impairment: a systematic analysis of WHO ICTRP registry.
Alzheimer's disease may develop from changes in the immune system, cell cycle, and protein processing following alterations in ribosome function.
Assessing modifiable risk factors for dementia in the Czech Republic: findings from the Survey of Health, Ageing and Retirement in Europe study.
Reallocation of time between accelerometer-derived movement behaviors, genetic susceptibility, and risk of incident dementia, mortality, and premature death: a longitudinal cohort study.
Impact of smoking, diabetes, and exercise on dementia risk in women with osteoporosis: a nationwide cohort study.
Brain health in Norwegian female former top-level football players: a protocol for a longitudinal cohort study.
A social return on investment analysis of the ENJOY Seniors Exercise Park IMP-ACT project: improving older people's health through physical activity.
Nomogram for predicting changes in cognitive function in community dwelling older adults with mild cognitive impairment based on Korea Longitudinal Study of Ageing Panel Data: a retrospective study.
A Critical Appraisal of the Links Between Video Gaming, Lifestyle Factors, Diet and Eating Behaviour: A Narrative Review.
Sex-specific modulation of FOLR1 and its cycle enzyme genes in Alzheimer's disease brain regions.
Life-course leisure-time physical activity benefits cognitive health through decelerating epigenetic ageing acceleration: results from a large prospective cohort study.
Mid-life cardiorespiratory fitness and risk of late-onset dementia incidence.
Effects of 5-Year Exercise Training on Cognition in Older Adults: 10-Years Follow-Up from the Generation 100 Study.
High-Density Lipoprotein Cholesterol and Cognitive Function in Older Korean Adults Without Dementia: Apolipoprotein E4 as a Moderating Factor.
Examining changes in neurofilament light chain following a behavioral weight loss intervention for adults with multiple sclerosis and obesity.
Individual and joint associations between sleep duration and physical activity with cognitive function: A longitudinal analysis among middle-aged and older adults in China.
Impact of Helicobacter pylori eradication on age-specific risk of incident dementia in patients with peptic ulcer disease: a nationwide population-based cohort study.

Association of life's simple 7 with cognitive function in a multi-ethnic cohort.
Agile Implementation of a Digital Cognitive Assessment for Dementia in Primary Care.
Five-Year Cognitive Trajectories in Individuals with Mild Cognitive Impairment and Mild Dementia: Associations with Attendance in a Multimodal Day-Care Program.
Chronic neck pain is associated with altered gait performance compared to healthy controls: Cross-sectional observations.
The mediating role of life course cognitive reserve-enhancing factors in the relationship between adverse childhood experiences and dementia among older adults: evidence from a prospective cohort study in the United States.
Subcortical gray matter volumes and 5-year dementia risk in individuals with subjective cognitive decline or mild cognitive impairment: A multi-cohort analysis.
Neuroinflammation as a Central Mechanism in Alzheimer's Disease: Therapeutic Insights from Schiff Base Derivatives.
Association between high plasma p-tau181 level and gait changes in patients with mild cognitive impairment.
Association of pulmonary function with the risk of incident Alzheimer's disease: a prospective cohort and Mendelian randomization study.
Subjective motoric cognitive risk syndrome: Preliminary prevalence from an online survey of a German cohort aged 50.
Associations of the 'weekend warrior' physical activity pattern with mild dementia: findings from the Mexico City Prospective Study.
Effects of a person-centered music-based intervention in the rehabilitation of older adults with mild to moderate dementia.
Sensory Difficulty, Social and Physical Activity, and Dementia Risk Among Older Adults.
The effects of traditional mind-body exercises on cognitive function in neurodegenerative diseases or prodromal cognitive decline: a meta-analysis.
Physician referral is associated with recruitment, motivation, and adherence in an exercise intervention study for older adults.
Association of long-term glycemic variability with longitudinal motor and nonmotor progression in patients with Parkinson's disease: an 8-year follow-up.
Changes in older persons' lifestyle and perceived health over time and during the COVID-19 pandemic: findings from the extended follow-up of the FINGER randomized controlled trial from 2009 to 2020.
Value of muscle strength in improving the predictive capability for cognitive decline in older women by established risk factors: A prospective cohort study in the Otassha Study.
Association of Medial Temporal Lobe Cerebrovascular Reactivity and Memory Function in Older Adults With and Without Cognitive Impairment.
Change in sarcopenia and the risk of dementia: evidence from the English Longitudinal Study of Ageing with a 10-year follow-up.
Depression and Cognitive Outcomes: Linking the Mayo Clinic Study of Aging and Rochester Epidemiology Project.
The brain structure underlying the nonlinear association between early-life tobacco smoke exposure and the risk for cognitive decline and dementia in adulthood: a large prospective cohort study.
Study protocol for a randomized controlled trial assessing clinical efficacy of digital cognitive rehabilitation for preclinical and mild clinical stages of alzheimer's disease continuum: the MI-RICORDO project.
Associations between self-reported change in lifestyle behaviors and brain health: Findings from the Healthy Brain Initiative.
Healthy lifestyle habits, educational attainment, and the risk of 45 age-related health and mortality outcomes in the UK: A prospective cohort study.
Association of high-sensitivity cardiac troponin I levels below the sex-specific 99th percentile with late-life dementia: the Perth Longitudinal Study of Ageing Women.
Light-Sheet Fluorescence Imaging Reveals Three-Dimensional Amyloid Burden Reduction Following Five Weeks of Swimming Exercise in Alzheimer's Mouse.
Psychometric Validation of the Bedford Alzheimer Nursing-Severity Scale in Community-Dwelling Persons With Severe Dementia.
Physically demanding work and cognitive impairment in late life: Results from the Brazilian longitudinal study of aging.
Physical activity, neuropsychiatric symptoms, and physical function in nursing home residents: the HUNT 70+ study.
The predictive power of brain-predicted age and leg strength on mobility decline in aging: findings from the Baltimore Longitudinal Study of Aging.
Effects of Combined Physical and Cognitive Interventions on Older Adults With Dementia: A Systematic Review and Meta-Analysis.
Brain amyloid-Œ≤ deposition, severity of subjective cognitive decline and gait speed in cognitively unimpaired oldest-old.
Minimum combined sleep, physical activity, and nutrition variations associated with lifeSPAN and healthSPAN improvements: a population cohort study.
Patterns of Factors in the National Institute on Aging Health Disparities Research Framework Domains and Mild Cognitive Impairment Risk.
Independent and joint association of accelerometer-measured sedentary behaviour and physical activity with mild cognitive impairment and intrinsic capacity decline among Chinese older adults: study protocol for a cross-sectional study.
Establishing global standards on wearable technology for measuring mobility in ageing populations: an international consensus exercise.
Handgrip strength and gait speed relationships with cognitive impairment in Iranian older adults: Birjand longitudinal aging study.
ZORA robot to assist a caregiver in prospective memory tasks: A preliminary study.
Physical activity as a resistance or resilience mechanism in Down syndrome Alzheimer's disease.
What Combinations of Modifiable Risk Factors Are Most Prevalent and Most Strongly Associated with Low Cognition? Results from Canadian Longitudinal Study on Aging.
Disparities in lifestyle among community-dwelling older adults with or without mild cognitive impairment: a population-based study in north-western China.
The associations between sedentary behavior and cognition in a population cohort of older adults.
Enhancing Alzheimer's disease prediction using random forest: A novel framework combining backward feature elimination and ant colony optimization.
Effects of Hand Strength and Walking Speed Combined and in Isolation on the Prediction of Cognitive Decline and Dementia in Middle-Aged and Older Adults: A Systematic Review and Meta-Analysis.
The impact of unpaid caring on cognitive function: a rapid review.
Cumulative burden of lifestyle risk factors on Alzheimer's disease in older Korean adults: A nationwide retrospective cohort study.
Comparing the Informative Value of 2-Minute Segments of the 6-Minute Walk Test: Insights into a Prospective Study on Parkinson's Disease.

Socioeconomic status modifies the association between adherence to the Mediterranean diet and cognitive outcomes: results from the Collaborative PROMED-COG Pooled Cohorts Study.
Motor features that distinguish isolated REM sleep behavior disorder patients from healthy controls: A systematic review.
Dementia risk literacy in older Australians: beyond knowledge.
Activity variability: A novel physical activity metric and its association with cognitive impairment.
Investigation of the gait parameters after donepezil treatment in patients with alzheimer' s disease.
The Association Between Exercise and the Well-Being of Dementia Patients' Caregivers: An Observational Study.
Relationships between fine memory discrimination and tau burden in two independent cohorts of older adults.
Effects of Combined Exercise and Cognitive Interventions on Cognition, Function, and Quality of Life in Dementia Patients.
Knowledge graph-augmented large language models for reconstructing life course risk pathways: a gestational diabetes mellitus-to-dementia case study.
Associations of Accelerometer-Measured Physical Activity With Dementia, Anxiety, and Depression Among Older Adults.
Behavioral Monitoring in Transient Ischemic Attack and Stroke Patients: Exploratory Micro- and Macrostructural Imaging Insights for Identifying Post-Stroke Depression with Accelerometers in UK Biobank.
Effectiveness of digital screening tools in detecting cognitive impairment among community-dwelling elderly in Northern China: A large cohort study.
Real-World Multimodal Day-Care Intervention for Mild Cognitive Impairment with Lewy Bodies: A Prospective 3-Year Comparative Cognitive Trajectory Study.
The association between sugar- and artificially sweetened beverages and risk of Alzheimer's disease: systematic review and dose-response meta-analysis of prospective cohort studies.
Time-restricted eating in early-stage Huntington's disease: A 12-week interventional clinical trial protocol.
Longitudinal relationships between depressive symptoms, functional impairment, and physical activity in later late life.
Temporal dynamics of white matter hyperintensities related to Alzheimer's disease in adults with Down syndrome.
A phase I, open-label study of intravenous human dental pulp stem cells (NestaCell¬Æ) at two dose levels in patients with Huntington's disease.
Decision-making involvement and quality of life in people with dementia: the mediating role of psychological needs.
Reallocating Time Between 24-h Movement Behaviors for Obesity Management Across the Lifespan: A Pooled Data Meta-Analysis of More Than 9800 Participants from Seven Countries.
Longitudinal associations of psychological distress with subsequent cognitive decline and dementia: a multi-cohort study.
Baseline and follow-up change of cholesterol levels predict dementia risk and progression in older adults: a U-shaped relationship.
Association of physical activity with incident dementia and cognitive decline among Australian older adults.
Association Between Physical Activity and the Risk of Dementia Among Patients With Depression: A Nationwide Cohort Study.
Levodopa-accelerated frailty: a hypothesis for a cumulative iatrogenic burden in Parkinson's disease.
Interaction of endocrine therapy for breast cancer with APOE4 status on cognition over 5-year follow-up.
Differences in lean mass and sarcopenia between individuals with Alzheimer's disease and those without dementia: A systematic review and meta-analysis of observational studies.
Clinical characteristics of pathological confirmed DLB without parkinsonism.
Association of Physical Activity with Dementia Risk in Cancer Survivors: A Korean Nationwide Cohort Study.
Cognitive function and its longitudinal predictability by intensity of physical activity in Chinese middle-aged and older adults.
Repeated measures of physical activity before dementia diagnosis in community-dwelling older adults: a longitudinal study.
Sustained social participation and dementia: evidence from a Japanese longitudinal cohort study with a time-varying exposure analysis.
Longitudinal transitions in sedentary behavior and physical activity in relation to all-cause and cause-specific mortality among postmenopausal women.
The use of restrictive practices in the everyday care of people living with dementia in hospital settings: an ethnographic study.
Association of wearable sensor-based gait analysis with phenoconversion trajectories in idiopathic REM sleep behavior disorder.
Association between adult education, brain volume and dementia risk: longitudinal cohort study of UK Biobank participants.
Mild cognitive impairment, Alzheimer's disease dementia, and predictors of driving cessation: A 7-year longitudinal prospective study.
Dancing through time: Cognitive changes over six years of community dance in Parkinson's disease.
Associations Between Physical Activity and Symptoms of Mental Health Disorders in Adolescence: Evidence From the Longitudinal Swedish Twin Register.
Association Between Gout and the Risk of Dementia: A Meta-Analysis of Observational Studies and Biological Mechanisms.
Accelerometer-based physical activity, air pollution and risk of dementia subtypes: a prospective study using UK biobank.
Independent associations of sleep and physical activity with cognition are mediated by hippocampal microstructure in middle-aged and older adults.
Ten-year longitudinal effects of physical activity and apolipoprotein E ..4 genotype on precuneus atrophy in Japanese older adults.
The CAIDE dementia risk score indicates elevated cognitive risk in late adulthood: a structural and functional neuroimaging study.
Walking reduces the risk of dementia in patients with Parkinson's disease: a longitudinal follow-up study.
Body composition differences in older adults with and without Alzheimer's disease: A systematic review and meta-analysis of observational studies.
Causal machine learning analysis identifies subpopulations most likely to benefit from healthy lifestyles for cognitive outcomes.
Synergistic impacts of physical activity and sleep on risk of dementia and all-cause mortality in Chinese older stroke survivors.
Cognitive reserve and its impact on cognitive and functional abilities, physical activity and quality of life following a diagnosis of dementia: longitudinal findings from the Improving the experience of Dementia and Enhancing Active Life (IDEAL) study.
Associations Between 10-Year Physical Performance and Activities of Daily Living Trajectories and Physical Behaviors in Older Adults.

The Association Between Body Weight Variability and Dementia and Cognitive Decline: A Systematic Review and Meta-Analysis of Cohort Studies With the GRADE Assessment.
Sex and gender differences in access, quality of care, and effectiveness of treatment in dementia: a scoping review of studies up to 2024.
Associations between accelerometer-derived physical activity complexity, cognitive function, and plasma biomarkers of Alzheimer's disease.
Tailored Online Physical Activity Coaching for Middle-Aged and Older Adults With Cognitive and Mental Health Concerns: Single-Arm Pre-Post Intervention Study.
The Associations of Sensory Impairment With 10-Year Risk of Dementia and Alzheimer's Disease: The Health and Retirement Study, 2010-2020.
Neurodegenerative Diseases in Male Former First-Class New Zealand Rugby Players.
Dexterity and Bimanual Coordination, Cognitive Function, and Mental and Cognitive Well-Being in People with Young Onset Dementia: A Case-Control Study.
Midlife and old-age cardiovascular risk factors, educational attainment, and cognition at 90-years - population-based study with 48-years of follow-up.
Antipsychotics and Change of Weight, Menstrual Cycle, and Metabolic Syndrome Components Among Schizophrenic Women: A 12-Week Longitudinal Prospective Study in Tien Giang, Vietnam.
Bidirectional associations of physical activity and cognitive function in midlife adults: a longitudinal analysis across 26¬†years follow-up.
Associations of Wearable-Measured Sleep and Physical Activity With Memory Performance in Older Adults: Cross-Sectional Study With Actigraphy and MRI.
Dynamic Relationship Between Sleep Patterns and Behavioral and Psychological Symptoms of Dementia: Longitudinal Observational Study.
Measurement Practice of Slow Gait Speed for Motoric Cognitive Risk Syndrome: A Systematic Review.
Prognostic Value of Plasma NfL and GFAP for Conversion to Alzheimer's Disease and Dementia in MCI: A Systematic Review and Robust Bayesian Meta-Analysis.
Longitudinal Estimation of Adequate Physical Activity Levels to Reduce the Risk of Alzheimer's Disease and Other Dementias in Older Adults With Mild Cognitive Impairment.
Cyclic 2.5D Perceptual Loss for Cross-Modal 3D Medical Image Synthesis: T1w MRI to Tau PET.
Association between long-term exposure to air pollutant mixture and the risk of Alzheimer's disease among older people: a prospective cohort study in China.
Risks and Benefits of Gastrostomy in Non-Motor Neurone Disease Progressive Neurological Diseases: A Systematic Review.
Effects of extreme heat on the onset of dementia and all-cause mortality: A 3-year follow-up study in a large population-based cohort in Japan.
Menstrual and reproductive factors and risk of Alzheimer's disease in elderly women: a cohort study in Eastern China.
A 10-week physical therapist-supervised exercise program for nursing home residents with dementia: a single arm, observational feasibility study.
Changes in Clinical Parameters During Low-Frequency Outpatient Pulmonary Rehabilitation for Male Patients With Chronic Obstructive Pulmonary Disease.
Identifying sex- and gender-specific endocrinological, lifestyle, psychosocial, and socio-cultural targets for Alzheimer's disease prevention in Africans: The Female Brain Health and Endocrine Research in Africa (FemBER-Africa) project.
Lifestyle factors and DNA methylation-based aging clocks: cross-sectional and longitudinal associations in the Singapore diet and healthy aging cohort.
The Benson Complex Figure Test for the Differential Diagnosis of Dementias.
Use of posterior probabilities from a long short-term memory network for characterizing dance behavior with multiple accelerometers.
Association between sleep disorder, sarcopenia, physical activity and clinical progression in people with Huntington's disease: A one-year longitudinal study using wearable technology.
Accelerometer-derived hourly segmentation of daily physical activity frequency and brain health: A prospective cohort study in middle-aged/older adults.
¬°Mi Vida Saludable!: Results of a Randomized, Controlled, 2 √ó 2 Factorial Trial of an In-Person and eHealth Diet and Physical Activity Intervention in Latina Breast Cancer Survivors.
Effects of energy intake on individual activities of daily living in patients with subacute stroke.
Mapping the neural substrate of high dual-task gait cost in older adults across the cognitive spectrum.
Gait variability as a marker of white matter integrity in individuals with Down syndrome.
Physical exercise as a non-pharmacological strategy to enhance glymphatic function.
Physical activity and blood-based biomarkers of neurodegeneration in community dwelling Australians from ISLAND (Island Study Linking Ageing and Neurodegenerative Disease).
Rural-urban differences in lipid abnormalities among middle-aged and older Indians.
Long-term risk of dementia with angiotensin receptor blockers versus angiotensin-converting enzyme inhibitors in hypertensive patients: A 15-year follow-up using the 45 and Up Study.
Cognitive Resilience for the Prevention of Mild Cognitive Impairment in Subjective Cognitive Decline: A Monte Carlo Simulation of a Digital Therapeutic Targeting Dementia Risk Factors.
The effect of aerobic exercise on cerebral perfusion in patients with vascular cognitive impairment, the Excersion-VCI randomised controlled clinical trial.
In-Hospital Physical Activity Predicts Swallowing Function at Discharge in Patients With Hip Fracture: A Prospective Study in Convalescent Rehabilitation Wards.
Modifiable lifestyle factors for dementia risk in an online cohort assessed by the MoCA Cognitive Health Assessment Index (MoCA-CHAI).
Multimorbidity patterns and 15-year trajectories of physical performance: a population-based study.
Individual Sleep Problems Are Associated With an Accelerated Decline in Multiple Cognitive Functions in Older Adults.
The 24-Hour Movement Behavior Composition and the Risk of Dementia.
Volume vs intensity of physical activity and risk of cardiovascular and non-cardiovascular chronic diseases.
Effects of self-managed lifestyle behavioral changes on cognitive impairment control in Chinese older adults: a population-based prospective study.
Prevalence of cognitive frailty among Chinese older adults: A systematic review and meta-analysis.
Biological aging and its association with serum neurofilament light chain levels in middle-aged African Americans: a prospective observational study.

Reduced Complexity of Pulse Rate Is Associated With Faster Cognitive Decline in Older Adults.
Longitudinal Associations between Cardiorespiratory Fitness and Cognition in Older Adults: A 10-Year Follow-Up from the Generation 100 Study.
Differential effects of a multidomain intervention on cognitive decline in older adults with type 2 diabetes according to white matter hyperintensity status: A secondary analysis of the J-MIND-Diabetes.
Multimodal Associations of Modifiable Risk Factors on White Matter Injury: The SOL-INCA-MRI Study (HCHS/SOL).
The relationship between age and physical activity as objectively measured by accelerometers in older adults with and without dementia.
Glymphatic System Dysfunction in Alzheimer's Disease: Insights into its Mechanisms, Diagnostic Imaging, and Therapeutic Perspectives: A Systematic Review.
Mentally Active Versus Passive Sedentary Behavior and Risk of Dementia: 19-Year Cohort Study.
Prevalence, aetiology, and service mapping of dementia in rural Uganda. Part of DEPEND Uganda (Dementia Epidemiology, unmet Need and co-Developing Solutions in Uganda).
How does social health impact cognitive function and brain reserve? Findings from the SHARED Consortium.
Digital Health Literacy and Physical Activity Programme for Improvement of Quality of Life in Caregivers of People with Dementia (CAREFIT): Study Protocol.
Effects of traditional Thai folk dance combined with cognitive stimulation program on behavior and cognition among older adults with cognitive decline: A randomized controlled trial.
The relationship between levels of physical activity, adherence to the MIND diet, and cognitive impairment in adults aged 65 years or older in Pakistan.
Cardiovascular Health and Biomarkers of Neurodegenerative Disease in Older Adults.
Assessing Caregiver Burden, Tasks, and Heart Rate Using Wearable Sensors: A Longitudinal Study of Informal Caregivers of Persons With Dementia and Older Adults.
Global Burden of Neurological Diseases Attributable to Behavioral Risks, 1990-2021.
Bidirectional Associations Between End-of-Life Communication and Depressive Symptoms in Older Adults.
Midlife plasma proteomic profiles indicate altered amyloid and tau processing in former elite rugby players.
Long-Term Physical Exercise for Preventing CKD in Older Adults: A Randomized Clinical Trial.
Ten-Year Cognitive Trajectories and Determinants in Chinese Older Adults Without Formal Schooling: Chinese Longitudinal Healthy Longevity Survey 2008-2018.
Association between plant-based dietary patterns and dementia among Chinese older adults.
Habitual physical activity and sedentary behavior among women with and without premenopausal bilateral oophorectomy: an exploratory study.
Effectiveness of exercise and nutrition interventions for cognitive function in older adults: A systematic review and meta-analysis.
Turning versus straight walking under dual-task conditions: a preliminary study for identifying mild cognitive impairment and Alzheimer's disease.
Standardization and clinical applications of retinal imaging biomarkers for cardiovascular disease: a Roadmap from an NHLBI workshop.
Gait and movement analysis for discrimination between people with dementia and healthy control persons based on pose estimation and machine learning.
Sleep characteristics and brain structure: A systematic review with meta-analysis.
Plant-Based Diets and Mental and Neurocognitive Health Outcomes: A Systematic Review With Meta-Analysis.
Comprehensive metabolomics profiling reveals novel biomarkers and pathways for early detection of Alzheimer's disease.
Sensor-based motion analysis for dementia detection: a systematic review.
Plasmaphosphorylated tau as biomarkers for multiple sclerosis diagnosis, subtyping, and prognosis.
Improving information sharing in Medicaid home and community-based services with learning health systems: Implications for older adults and individuals living with disabilities and dementia.
Harmonizing late-life cognitive performance data across two population-based cohort studies: The Health and Retirement Study and National Health and Aging Trends Study.
Development of a predictive model for long-term care and support eligibility certification associated with dementia-related functional impairments: the IRIDE cohort study.
Deciphering the mechanisms: Pathophysiology of migraine-related cognitive dysfunction.
Imaging brain fluid dynamics and waste clearance involving perivascular spaces in cerebral small vessel disease.
Detecting Dementia Using Lexical Analysis: Terry Pratchett's Discworld Tells a More Personal Story.
A pilot study investigating the effectiveness, appreciation, and feasibility of a cognitive stimulation program in dementia patients: online versus face-to-face.
Correction to "Cardiovascular health and rate of cognitive decline in preclinical dementia: A 12-year population-based study" by Speh et al. (2024).
Associations of Physical Activity Trajectories With Transitions Across Cognitive States: A Multinational Cohort Study.
Multimodal passive smartphone sensing in older adults: a guide for clinical scientists based upon an ongoing cohort study.
Evaluation of the Falls Management Exercise (FaME) programme implemented across three United Kingdom regions: a prospective cohort study.
Neuroprotective Dietary Patterns and Longitudinal Changes in Cognitive Function in Older Adults.
Association of body shape index and body fat percentage with geriatric assessment outcomes.
Healthy Lifestyle, multimorbidity network, and all-cause mortality among older Chinese: a longitudinal analysis in Chinese longitudinal healthy longevity survey.
Mapping Evidence on the Effectiveness of Mind-Body Interventions and Brain Gym on Cognitive Function in Older Adults: A Scoping Review.
Socio-emotional well-being is associated with cognitive function and informant-rated cognitive decline: Results from the Harmonized Cognitive Assessment Protocol in Europe.
Mitochondrial Dysfunction Correlates with Brain Amyloid Binding, Memory, and Executive Function in Down Syndrome: Implications for Alzheimer's Disease in Down Syndrome.
Effectiveness of a brain exercise program using game-based cognitive enhancement to reduce mild cognitive impairment among older adults in Pathum Thani Province, Thailand: a quasi-experimental study.

Critical Review of the Methodological Shortcoming of Ambulatory Blood Pressure Monitoring and Cognitive Function Studies.
Associations between life course exposure to ambient air pollution with cognition and later-life brain structure: a population-based study of the 1946 British Birth Cohort.
Modifiable lifestyle factors influencing neurological and psychiatric disorders mediated by structural brain reserve: An observational and Mendelian randomization study.
Brain age gap as a predictive biomarker that links aging, lifestyle, and neuropsychiatric health.
The associations of early and surgical menopause with 10-year employment trajectories bracketing final menstruation or surgery.
APOE4 genotype and MAPT haplotype modify repetitive head impact biomarkers in retired professional fighters.
Relationships among young adult general cognitive ability, midlife physical activity, and early late-life cognitive functioning: A four-decade longitudinal cohort study in men.
The role of Life's Crucial 9 in cardiovascular disease incidence and dynamic transitions to dementia.
Prevalence and symptoms of incidental meningiomas: a population-based study.
Predictors of Fall-Related Injuries in Fallers-A Study in Persons with Cognitive Impairment.
Risk factors for Alzheimer's disease and cognitive function before middle age in a U.S. representative population-based study.
Enhancing functional recovery after ACL injury. A protocol for a randomized control trial of transcranial direct current stimulation over the motor cortex.
Daily steps and health outcomes in adults: a systematic review and dose-response meta-analysis.
Amyloid-related changes in fluency in patients with subjective cognitive decline.
Differences in Long COVID severity by duration of illness, symptom evolution, and vaccination: a longitudinal cohort study from the INSPIRE group.
Association of Urinary Equol Concentration with Frailty in Community-Dwelling Older Adults: The Itabashi Longitudinal Study on Aging.
The association between sleep parameters, cognitive functioning, and markers of brain morphology: The Maastricht Study.
Cardiorespiratory Fitness, Multimorbidity Risk, and 15-Year Trajectories in Chronic Disease Accumulation: A Prospective Longitudinal Study.
Dual-Site Pain in the Low Back and Knee as a Risk Factor for Dementia: A Population-Based Cohort Study.
The simple task of lifting five kilograms serves as a predictor of age-related disorders in old adults.
Factors Associated With Being Perceived as Vocally Young in Older Adults.
Clinical study on gray matter volume reduction, gait disorders, and fall risk in patients with Alzheimer's disease.
Prehospital pain management in older adults with proximal Femur fractures: current practices and implications for delirium prevention.
Associations of lifestyle and health factors with subjective memory complaints in a large cohort of mHealth users.
Functional Recovery Predictors in Hip Fractures: Insights from a Community Clinical Pathway.
Effects of neighborhood amenities, services, and built infrastructure on cognitive health: A longitudinal study of older Chinese immigrants in Chicago, United States.
Associations between contralesional neuroplasticity and motor impairment through deep learning-derived MRI regional brain age in chronic stroke (ENIGMA): a multicohort, retrospective, observational study.
Cross-sectional and longitudinal associations between subthreshold depressive symptoms and cognition, physical health, and quality of life in older adults aged 55+: a retrospective study.
Two-year follow-up assessment of a randomized controlled trial evaluating the durability of Tai Chi on cognitive function in older adults with type 2 diabetes and mild cognitive impairment.
Characterization of cognitive decline in long-duration type 1 diabetes by cognitive, neuroimaging, and pathological examinations.
Unhealthy lifestyle factors and the risk of incident depressive symptomatology in older adults: Findings from the Three-City study.
Motor function tests as early indicators of cognitive and functional decline in older adults: A correlational study.
The impact of Internet use on cognitive function in older adults: Based on the cognitive reserve hypothesis.
Sex and All-Cause Mortality in the US, 1999 to 2019.
Associations of physical activity, sedentary behavior, and sleep patterns with cognitive function among middle-aged and older adults.
Association between unhealthy lifestyles and young-onset dementia: a nationwide cohort study.
The moderation and mediation roles of white matter insults in relationship of APOE Œµ4 genotype with Alzheimer's disease and related phenotypes: two longitudinal studies.
The impact of high-intensity interval training on cerebrovascular function in the APP/PS1 mice.
Comparative efficacy of cognitive training modalities in cognitive impairment: A systematic review and network meta-analysis.
Association Between Dietary Patterns and Cognitive Function in Midlife Adults: The Bogalusa Heart Study.
A deep-learning based biomarker of systemic cellular senescence burden to predict mortality and health outcomes.
Risk of Falls and Need of Walking Aid in Parkinson's Disease: Incidence and Impact of Comorbidities.
Impact of mid-life cardiovascular health on cognitive change in a bi-ethnic cohort.
The use of fully immersive virtual reality for screening neurodegenerative diseases: A systematic review of behavioral and diagnostic outcomes.
Intention to use and perceived satisfaction with a digital tool for dementia prevention among the Dutch general public: a cross-sectional study.
Survival of the fittest? Peak oxygen uptake and all-cause mortality among older adults in Norway.
Long-term Alzheimer's disease mortality prediction in adults aged ‚â•60 years: A prospective cohort study benchmarking survival machine learning algorithms.
Domain-specific longitudinal associations between brain volume, white matter lesions, and cognitive function changes.
Microbiome functional gene pathways predict cognitive performance in older adults with Alzheimer's disease.

Latent Profiles Based on Combined Risk Factors for Cognitive Decline in European Older Adults: A Retrospective Study Based on the SHARE HCAP Project.
Impacts of an antioxidant-rich diet and lifestyle factors on gut microbiota diversity and brain health: An exploratory analysis from the NutBrain study.
Western Mediterranean diet predicts 9-year changes in episodic memory in an adult lifespan sample of Americans.
The Impact of Transcranial Direct Stimulation Therapy Combined with Intranasal Near-Infrared Stimulation on Cognitive Performance in Patients with Mild Cognitive Impairment and Alzheimer's Disease: A Pilot Randomized, Double-Blind, Placebo-Controlled Study.
The Multifaceted Role of Irisin in Neurological Disorders: A Systematic Review Integrating Preclinical Evidence with Clinical Observations.
The association between cognitive function and physical performance in established users of a lower limb prosthesis.
Cognitive behavioral therapy for insomnia in neurodegenerative disorders-targeting sleep disturbances in Alzheimer's and Parkinson's disease: a scoping review.
High estimated pulse-wave velocity is associated with lower brain white matter microstructural integrity twelve years later.
Apathy is distinct from depression or fatigue and is associated with poor physical health in an older community cohort.
Assessing the feasibility of interactions within a computer-generated virtual reality for people with dementia.
TaiChi-MSS protocol: enhancing cognitive and brain function in MCI patients through Tai Chi exercise combined with multisensory stimulation.
Association of the cumulative modified life's Essential 8 score with cognitive change: Results from two longitudinal cohorts.
Bridging Pain and Motoric Cognitive Risk Syndrome: Mediating Effect of Handgrip Strength.
Recognizing, managing, and preventing cognitive sequelae after stroke. A narrative review.
Association Between Dietary Variety and Masticatory Behaviors Measured Using Wearable Device Among Community-Dwelling Older Adults in Japan: A Multilevel Meal-by-Meal Analysis.
The proportion of plant-based food consumption during midlife and cognitive health in later life in Australian women: data from the Women's Healthy Ageing Project (WHAP).
Repetitive Transcranial Magnetic Stimulation and Tai Chi Chuan for Older Adults With Sleep Disorders and Mild Cognitive Impairment: A Randomized Clinical Trial.
Cognitive Impairment-Associated Risk Factors of Parkinson's Disease: A Hospital-Based Study in a Cohort of Upper Egypt Parkinson's Patients.
Effects of Resistance Training on Motor and Cognitive Function in Older Adults with Alzheimer's Disease: A Systematic Review.
Poor sleep health is associated with older brain age: the role of systemic inflammation.
Neighborhood deprivation on increasing deficit accumulation in older breast cancer survivors and noncancer control individuals.
Leisure activity engagement attenuates the risks of cognitive impairment induced by unhealthy plant-based diets: a nationwide cohort study.
Association of tongue, handgrip, and pinch strength with blood-based phosphorylated-tau 181 in cognitively healthy older adults.
Brain-rejuvenating factor TIMP2 is associated with brain health and neuroprotective lifestyle in aged subjects.
Egg intake and cognitive function in healthy adults: A systematic review of the literature.
The Role of Fatty Acid Binding Proteins in Neuropsychiatric Diseases: A Narrative Review.
Modeling the importance of life exposure factors on memory performance in diverse older adults: A machine learning approach.
Development and validation of a machine learning model for early screening of high-risk mild cognitive impairment from the multi-cohort data.
Amyloid-Beta Pathology and Cognitive Performance in Centenarians.
Survival and long-term (minimum 10-year) outcomes of bony increased-offset-reverse shoulder arthroplasty.
Effects of a Digital, Person-Centered, Photo-Activity Intervention on the Social Interaction of Nursing Home Residents with Dementia, Their Informal Carers and Formal Carers: An Explorative Randomized Controlled Trial.
Emergency department visits due to severe falls: comparing patient self- reports and general practitioner records: A cross-sectional study.
Nivolumab-induced Limbic Encephalitis in Gastric Cancer: A Case Report and Literature Review.
Dietary patterns and endothelium dysfunction: a literature review.
Cost-utility of implantable tibial neuromodulation (Revi) versus conservative therapy for urgency urinary incontinence in adults: a US payer perspective.
The association between medical fitness facility attendance and incident mental disorders.
Co-designing an interprofessional care pathway for (risk of) malnutrition and sarcopenia in community-dwelling older adults.
Factors related to blood-based biomarkers for neurodegenerative diseases and their intergenerational associations in the Young Finns Study: a cohort study.
A systematic review and meta-analysis of the impact of transcranial direct current stimulation on cognitive function in older adults with cognitive impairments: the influence of dosage parameters.
Wearables-derived risk score for unintrusive detection of Œ±-synuclein aggregation or dopaminergic deficit.
Associations between life-course cognitive reserve and cognitive domains over 8 years in a large cohort of middle-aged and older adults: The Brazilian Longitudinal Study of Adult Health (ELSA-Brasil).
Loss of microstructural integrity in left hemispheric white matter tracts is associated with poorer digits in noise understanding.
Role of leisure and sleep in promoting daily physical activity in adults with down syndrome.
Association between enlarged perivascular spaces in the basal ganglia and the Frontal Assessment Battery: A cross-sectional study.
Eight decades of follow-up link life course exposures to proteomic organ ageing and longevity.
Smartphone self-testing of hand and speech motor functions: a study of reliability and usability in older adults.
Feasibility of assessing cognitive impairment via distributed camera network and privacy-preserving edge computing.
Linking VR performance to cognitive ability: The significance of ACC-PCL connectivity in aging populations.
Prevalence and healthy life expectancy among elderly with mild cognitive impairment in Chinese rural areas.
Low intrinsic capacity is associated with risk of developing mild cognitive impairment in the English Longitudinal Study of Ageing (ELSA).

An Immersive Virtual Reality Room to Enhance Positive Affect and Engagement in Nursing Home Residents with Neurocognitive and Psychological Disorders: A Feasibility Study.
Assessment of changes in fatigue and cognitive functioning in the absence of progression independent of relapse activity.
Vascular risk factor variability and cognitive impairment in older adults a community-based retrospective study.
Evaluating the impact of a national brain health education course for older adults with intellectual and developmental disabilities and caregivers: Brain Health-IDD Program protocol.
Exercise-based modulation of the gut-brain axis: a new direction for neurorehabilitation in Alzheimer's disease.
Genetic predisposition to hand grip strength predicts cognitive decline.
Dementia, Systemic Biomarkers, and Risk of 30-Day Readmission After Pneumonia: A Multi-Center Cohort Study.
Preoperative Cognitive Optimization and Postoperative Cognitive Outcomes: A Narrative Review.
[Effects of a One-Week Inpatient Health Programme for Family Caregivers and Persons in Need of Care (SVLFG Care Tandem) - A Quasi-Experimental Study].
Short-term and long-term reversion rates to normal cognition and their contributing factors among individuals with mild cognitive impairment in a Japanese community: the Hisayama study.
The relationship between cognitive screeners and everyday functioning in amyloid-positive participants from the Amsterdam Dementia Cohort.
Clinical importance of simple muscular fitness tests to predict long-term health conditions: a systematic review and meta-analysis of 94 cohort studies.
Neural and motor mechanisms of handwriting: from healthy aging to neurodegenerative disorders.
Frailty subtypes and white matter alterations in older adults without dementia.
The association of ultra-processed food intake with neurodegenerative disorders: a systematic review and dose-response meta-analysis of large-scale cohorts.
Differential association of ultraprocessed food categories with risk of developing cognitive impairment in middle-aged and older adults in a longitudinal panel study.
Genetic susceptibility to Alzheimer's disease and cardiometabolic risk from childhood.
A timeline of symptom onset and disease progression in CLN3 disease.
Stage-specific temporal associations between body mass index trajectories and Alzheimer's disease pathologies.
Age-dependent phenotypes of cognitive impairment as sequelae of SARS-CoV-2 infection.
Diurnal dynamics of multilayer brain networks predict cognitive trajectories in aging.
Relationship between Depressive Moods and Oral Frailty in a Frailty Outpatient Clinic: A Cross-Sectional Study.
All are not created equal: Method descriptions in an epidemiology publication differ among media summaries - A case study comparison.
Association between indoor ventilation frequency and low muscle mass among older adults in China: a national cross-sectional research.
Is the freezing index a valid outcome to assess freezing of gait during turning in Parkinson's disease?
Disentangling causality in brain aging: The complex interplay between glial senescence, neuroinflammation, and neurodegeneration.
Risk factors for surgical site infection following treatment of proximal femoral fracture: a matched-pair analysis.
Data from multi-national aging cohorts show dysregulated metabolic pathways in people with physio-cognitive decline.
Development and validation of screening tools for motoric cognitive risk syndrome in community settings.
Determinants of medical costs in patients with and without fractures using Korean Senior Cohort Study.
The longitudinal association between dyslipidemia and cognitive trajectory.
Clinical and cost-effectiveness of a home-based health promotion intervention for older people with mild frailty in¬†England: a multicentre, parallel-group, randomised controlled trial.
Transcranial Pulse Stimulation in Alzheimer's: Long-Term Feasibility and a Multifocal Treatment Approach.
Mild Behavioral Impairment: A Population-Based Study.
Gut microbiome and cognitive function in the Hispanic Community Health Study/Study of Latinos.
Relationship between social determinants of health and cognitive performance in an older American population: a cross-sectional NHANES study.
Double-edged sword effect of Sutterella in neurological disorders: implications for the gut-brain axis and neuroimmune interactions.
A Longitudinal Investigation of Prediabetes, Diabetes, HbA1c and Cognitive Trajectories Among Cognitively Unimpaired Individuals.
Effects of the MIND Diet on the Cognitive Function of Older Adults: A Systematic Review.
Transcriptomic profiles in major depressive disorder: the role of immunometabolic and cell-cycle-related pathways in depression with different levels of inflammation.
Dynamics of oral language and speech production through neuromodulation: A systematic review of non-invasive brain stimulation in neurodegeneration.
[Cognitive and psychiatric evolution in autoimmune encephalitis in a school-aged child: Clinical case].
The role of nutrition and multimodal lifestyle interventions in Alzheimer's prevention and management: a mini-review.
Rapid Access Atrial Fibrillation Clinics in Australia - Modelling Outcomes and Cost-Effectiveness.
Circulating immune cell phenotypes are associated with inflammatory biomarkers in dementia-free participants from the Framingham Heart Study Offspring cohort.
The impact of APOE Œµ4 on cognition in Brazilians with type 2 diabetes: exploring ethnicity effects.
Sex-stratified mortality estimates in people with schizophrenia: A systematic review and meta-analysis of cohort studies of 2,700,825 people with schizophrenia.
Harnessing Geospatial Artificial Intelligence (GeoAI) for Environmental Epidemiology: A Narrative Review.
Brain Age as a Biomarker in Alzheimer's Disease: Narrative Perspectives on Imaging, Biomarkers, Machine Learning, and Intervention Potential.
Mechanistic research on the vestibular-hippocampal pathway in neurodegenerative diseases: an integrative perspective from molecular to behavioral levels.

Canadian Stroke Best Practice Recommendations Rehabilitation, Recovery, and Community Participation Following Stroke, Part Three: Optimizing Activity and Community Participation Following Stroke , 7th Edition Update, 2025.
Higher dietary vitamin D intake influences brain and mental function in elderly Americans: a cross-sectional analysis.
DXA and cardiovascular disease in rheumatoid arthritis: A scoping review.
Œônnovative Health Promotion Strategies: A 6-Month Longitudinal Study on Computerized Cognitive Training for Older Adults with Minor Neurocognitive Disorders.
Longitudinal Changes of Quantitative Brain Tissue Properties Induced by Balance Training.
Proteomic signatures of the APOE Œµ4 and APOE Œµ2 genetic variants and Alzheimer's disease.
Comorbid health outcomes in patients with schizophrenia: an umbrella review of systematic reviews and meta-analyses.
Individual and Institutional Factors Associated With Urinary Incontinence Among Nursing Home Residents: A Multilevel Analysis.
Association between Obesity and Cognitive Function in Chinese Older Adults: The Mediating Effects of Sleep Quality and Blood Pressure.
Baseline predictors of cross-sectional and longitudinal performance on the symbol digit modalities test in individuals with multiple sclerosis.
Trends in cognitive impairment among older adults in China from 2002 to 2022: Evaluating the impact of the COVID-19 pandemic.
Assessment of Blood Glucose Measurement Using New Noninvasive Technology: Protocol and Methodology.
Interventions for preventing falls in older people in care facilities.
Clinical and radiographic outcomes of shoulder hemiarthroplasty for patients with glenoid medialization.
Effects of multidomain intervention on cognitive function in older adults with cognitive impairment: A multicenter, four-arm, cluster randomized trial.
The Role of the Glymphatic System in Alzheimer's Disease: Mechanisms, Evidence, and Therapeutic Implications.
Adhering to Healthy Dietary Patterns Prevents Cognitive Decline of Older Adults with Sarcopenia: The Mr. OS and Ms. OS Study.
Do Predictors of Life Satisfaction Change in the Last Years Leading Up to Retirement? The Case of Job Satisfaction and Leisure Satisfaction.
The interplay between sleep and neural respiratory drive in COPD: contribution of semi-automated analysis of long duration recordings.
High-dimensional proteomic analysis for pathophysiological classification of traumatic brain injury.
Diagnostic accuracy of the Montreal Cognitive Assessment in screening for cognitive impairment in initially hospitalized COVID-19 patients: Findings from the prospective multicenter NeNeSCo study.
The Clinical Trial Landscape in Autoimmune Encephalitis: Challenges and Opportunities.
Hand grip strength and cognitive dysfunction amongst older Africans in Nigeria.
Construct validity, intrarater and interrater reliability of the Italian version of the systematic screening of handwriting difficulties (SOS) test in people with Parkinson's disease.
Neuronal Cell-Cycle Re-entry Defines Divergent Outcomes Through Replication-Dependent DNA Damage in ALS.
Adherence to Life's Essential 8 enhances gut microbiota diversity and cognitive performance.
Social network size and cognitive decline in older adults experiencing depressive symptoms and loneliness.
Herpes zoster vaccination and the risk reduction of ophthalmic diseases: A nationwide emulated target trial in South Korea.
Methodological standards for body composition assessment - an expert-endorsed guide for research and clinical applications: bioimpedance, dual-energy X-ray absorptiometry, computerized tomography, and ultrasound methods.
Stroke Action Plan for Europe 2018-2030 (SAP-E): mid-term review and update.
Evaluation and quality improvement projects (EQUIP) bootcamp: development, implementation, and evaluation with learners in geriatrics.
Geo-Mamba: Geometry-informed state-space learning of functional brain organization.
Clinical and MRI Correlates of Œ≤-Amyloid Load Inconsistent With Its Presumed Neurotoxicity in Cognitively Healthy Ageing.
Exposure to Cadmium and Cerebrovascular Mortality in the United States.
Trends in cause-specific mortality among people with and without diabetes in high-income settings: a multinational, population-based study.
Age-dependent remodeling of the sciatic proteome in 5xFAD mice can be attenuated by exercise or donepezil treatment to maintain neuromuscular function.
The Impact of Chronic Pain on Cognitive Function.
Physical activity and APOE neuropathology score modify the association of age and [(11)C]-PiB-PET amyloid burden in a cohort enriched with risk for Alzheimer's disease.
Sleep Duration, Sleep Timing, and Self-Reported Wellbeing in Collegiate Women Volleyball Athletes: A Prospective Study Using a Consumer-Grade Wearable.
Speech-based digital biomarkers for early etiological stratification of Alzheimer's disease and frontotemporal degeneration: a biomarker-confirmed prospective study.
The association between the MIND-NL diet, Dutch dietary guidelines, and global cognitive function in an older population at risk for cognitive decline.
Clinicopathological study of chronic traumatic encephalopathy pathology in a population-based cohort.
Dynamic neurocognitive adaptation: A follow-up exposome investigation in aging.
Motor learning induces myelin-related white matter changes revealed by MRI-based in vivo histology.
Epidemiological Principles in Claims of Causality: An Enquiry into Repetitive¬†Head Impacts (RHI) and Chronic Traumatic Encephalopathy (CTE).
Marijuana use and subjective cognitive decline in middle-aged and older adults: Analysis of the behavioral risk factor surveillance system survey.
Environmental toxicants and Parkinson's disease: recent evidence, risks, and prevention opportunities.
Nuclear BIN1 isoforms regulate c-Myc-mediated cell cycle control in oligodendrocytes.
Caffeine Intake and Cognitive Function Among US Adults Aged 60+: Insights From NHANES 2011-2014.
Causal factors for migraine in Mendelian randomization studies: a systematic review and meta-analysis.

Catecholamine Dysregulation in Former American Football Players: Findings From the DIAGNOSE CTE Research Project.
Development of a neural network-based risk prediction model for mild cognitive impairment in older adults with functional disability.
Cognitive Function as a Predictor of Incident Hypertension in Hispanic/Latino Adults: Results From the Community Health Study/Study of Latinos.
Large-Scale Gene-Smoking Interactions and Fine Mapping Study Identifies Multiple Novel Blood Pressure Loci in over 1 Million Individuals.
Lipoprotein(a)-Associated Proteomic Signature Predicts Cardiovascular Disease in Young Adults.
Social Connectedness in Older Adults: The Potential of Social Internet Use to Maintain a Strong and Stable Personal Network.
Disruption of circadian rhythms is associated with cognitive impairment during gestation.
Analysis of Preventive Effect of Bisphosphonate for Osteoporotic Fracture in Patients with Alzheimer's Disease and Patient Mortality.
Health characteristics and health behaviours in male former contact sports participants: comparison with general population controls in a Finnish cohort study.
Cardiovascular risk factors are associated with lower posterior-medial network functional connectivity in older adults.
Association of impaired olfactory identification with prevalent mild cognitive impairment and regional brain atrophy: the Hisayama Study.
Medical Retirement From Sport After Concussion: A Survey of Perspectives and Experiences of Collegiate Physicians and Athletic Trainers.
Global environmental change and the gut-kidney-brain axis: a review and framework of vulnerability and resilience.
Body understanding measure for pregnancy scale (BUMPS): Psychometric properties and predictive validity with postpartum anxiety, depression and body appreciation among Italian peripartum women.
Changes in Traumatic Brain Injury Patterns Before and During the COVID-19 Pandemic: A Single-Center Study in Brazil.
Cognitive deterioration in patients undergoing hemodialysis: how variations in age influence the development of new mechanisms and treatment approaches?
IDEA-FAST clinical study protocol: Identifying digital end-points of fatigue, sleep quality and daytime sleepiness in N,Äâ=,Ää2000.
Establishment and verification of reference intervals for Alzheimer's disease plasma biomarkers based on evaluation of pre-analytical procedures: a multicenter study.
Nonlinear Gait Variability and the Role of Cognitive-Physical Exercise in Mitigating Mobility Decline in Institutionalized Older Adults with Cognitive Impairment.
Association of Gait Speed With Cognitive Outcomes in Older Adults With Hypertension: A Secondary SPRINT MIND Analysis.
Promoting Physical Activity to Lower the Incidence of Major Non-Communicable Diseases - A Literature Review to Inform a Simulation Model for Policy Purposes.
Imaging the later-life white matter pathologies of repetitive head impacts: A novel pattern revealed through T2 FLAIR MRI.
Effects Of Telehealth Program On Physical Activity Level In Older Adults With Dementia During The Pandemic: Randomized Clinical Trial: 2328.
An international multi-cohort investigation of self-reported sleep and future depressive symptoms in older adults.
Surface-based morphometry reveals divergent aging trajectories in veterans with and without traumatic brain injury.
Association between dynapenic abdominal obesity and mild cognitive impairment among middle-aged and older community-dwelling adults.
"SHANK3 deficiency alters early progenitor dynamics and reveals shared pathways with neurodegeneration".
Mapping the causal chain from genetic risk variants to lipid dysmetabolism in Parkinson's disease.
Sphingolipid and ceramide associations with tau pathology vary across diverse ethnoracial groups in postmortem brain tissue.
Identifying Subclinical Transthyretin Cardiac Amyloidosis in V142I TTR Carriers: Design, Rationale, and Methods of the VISTA (Variant Imaging of Subclinical Transthyretin Amyloidosis) Study.
Polygenic prediction of body mass index and obesity through the life course and across ancestries.
Salt-sensitive hypertension promotes neuronal mitochondrial stress and neurodegenerative alterations via neuro-vascular metabolic reprogramming and local RAS signaling.
Gene-by-Sleep Duration Interaction for Glycemic Traits in over 480,000 Individuals.
Changing life expectancy in European countries 1990-2021: a subanalysis of causes and risk factors from the Global Burden of Disease Study 2021.
Genome-wide gene-sleep interaction study identifies novel lipid loci in 732,564 participants.
Faecal mucoprotein MUC2 is decreased in multiple sclerosis and is associated with mucin degrading bacteria.
Burden of 375 diseases and injuries, risk-attributable burden of 88 risk factors, and healthy life expectancy in 204 countries and territories, including 660 subnational locations, 1990-2023: a systematic analysis for the Global Burden of Disease Study 2023.
Menopausal hormone therapy and the female brain: Leveraging neuroimaging and prescription registry data from the UK Biobank cohort.
Characterising acute and chronic care needs: insights from the Global Burden of Disease Study 2019.
Arterial stiffness moderates the link between NfL and cognition: The IGNITE study.
Duration of contact sports play associated with aberrant DNA methylation in human frontal cortex.
Biological Ageing Acceleration and Functional Capacities Across the Lifespan in the INSPIRE-T Cohort.
Chronic Traumatic Encephalopathy Neuropathologic Change is Infrequent Following Isolated Moderate-Severe Traumatic Brain Injury.
Chronic traumatic encephalopathy neuropathologic change in the Late Effects of Traumatic Brain Injury project: Findings in a community autopsy cohort.
Ambulatory blood pressure monitoring, European guideline targets, and cardiovascular outcomes: an individual patient data meta-analysis.
Global, regional, and national progress towards the 2030 global nutrition targets and forecasts to 2050: a systematic analysis for the Global Burden of Disease Study 2021.
Global burden of 292 causes of death in 204 countries and territories and 660 subnational locations, 1990-2023: a systematic analysis for the Global Burden of Disease Study 2023.
CNM-Au8 in Amyotrophic Lateral Sclerosis: The HEALEY ALS Platform Trial.
Subjective Cognitive Decline in Brazilian Adults: Prevalence and Associated Social, Lifestyle, and Health-Related Factors: A Nationally Representative Cross-Sectional Analysis from the ELSI-Brazil Cohort

Accelerometer-measured physical activity, sedentary behavior, and incident neuropsychiatric diseases: a large prospective cohort study of 73411 participants
Effectiveness of a 52-week multidomain intervention to maintain cognitive function in patients with mild cognitive impairment: a randomized controlled trial
Beyond Somatic Multimorbidity: Longitudinal Effects of Edentulism and Mental Health on Cognition in Older Adults
Alzheimer Caf√©: A Pilot Study in Italy
Context Is Medicine: Integrating the Exposome into Neurorehabilitation
Cognitive and physical functions among Chinese community-dwelling older adults with motoric cognitive risk syndrome: A prospective cohort study
Prevalence and Risk Factors of Cognitive Impairment Among the Aging Population in India: A Cross-Sectional Study
Disease clusters and their genetic determinants following a diagnosis of depression: analyses based on a novel three-dimensional disease network approach
Cognitive decline before and after incident chronic respiratory disease
Cognitive changes and emotional heart rate variability dynamics in subjective cognitive decline: An exploratory longitudinal neuropsychophysiological study
Stem cell therapy use in patients with dementia: a systematic review
The perioperative impact of frailty and cognitive impairment in older surgical patients: A multicentered longitudinal cohort study
Cognitive remediation of attention in patients with Alzheimer's disease: A randomized clinical trial
Glycemic status, early-onset and late-onset dementia, dementia-free lifespan, and brain structure: A population-based cohort study
Region-specific brain structural modulation and amyloid-Œ≤ pathology associated with dietary biotin: insights into dementia neuropathology
Prevention of dementia using mobile phone applications (PRODEMOS) ‚Äì a health-economic cost-utility analysis in people aged 55‚Äì75 years with low socio-economic status
Workplace health promotion for healthy aging: a randomized controlled trial within the Semmelweis‚ÄìEUniWell Workplace Health Promotion Model Program comparing email-based and face-to-face motivational interviewing to increase step count
Trajectories of depressive symptoms in older adults: Correlates and consequences for mortality
Baseline characteristics of the SINgapore GERiatric Intervention Study to Reduce Cognitive Decline and Physical Frailty (SINGER) multidomain dementia prevention randomized controlled trial and insights from the recruitment process
Prediction Model for eGFR Thresholds Guiding the Optimal Timing of Hemodialysis Preparation in Chronic Kidney Disease
Prediction of all-cause mortality in Parkinson‚Äôs disease with explainable artificial intelligence using administrative healthcare data
Six-Month Prognostic Tool for Community-Dwelling People With Dementia
Metabolomic biomarkers of rest-activity rhythms in older women: Results from the Women's Health Initiative study
The association of metabolic syndrome and its components with brain volumes and structural connections: a systematic review and meta-analysis of observational studies
Dividing lives: The impacts of delayed school tracking on inequalities in cognitive aging in Europe
Associations Between Growth Differentiating Factor-15 and Frailty in Older Adults From the MAPT Study
Neighborhood food store and subsequent health and well-being of older adults in Japan: an outcome-wide study
Association between midlife intake of ultra-processed foods and later-life subjective cognitive complaints: Evidence from the new York University women's health study
Rising energetic cost of walking predicts cognitive impairment
Taurine Intake, Plasma Taurine Concentration, and Dementia Risk: Findings From the Malm√∂ Diet and Cancer Study
Blood-brain barrier disruption, traumatic encephalopathy, and cognitive decline in retired athletes
Sham surgery as an alternative procedure for unexplained pain following transvenous implantable cardioverter defibrillator implantation: case report
A complex intervention to reduce hospital admissions for people living with dementia in shared-housing arrangements in Germany: results of the multicenter, cluster-randomized controlled DemWG-study
Wearable technology for mobility measurement in orthopedics and traumatology: a comparison of commercially available systems
An interdisciplinary rehabilitation program for adults with dementia‚ÄîA randomized controlled pilot trial evaluating social participation, loneliness and mental health
Prospective association of depression symptoms with exceptional longevity among older women
The COMFORTage project: study protocol for the integration of multiple sources towards personalised preventions at Ace Alzheimer Center Barcelona
Estimated effects of food taxes and subsidies on health, economics, and equity in Australia: a modelling study
Genetic mechanisms, brain structures, and peripheral biomarkers mediate the relationship between physical frailty and neuropsychiatric disorders
Predictors of shunt response in iNPH: differentiating responders, early and late non-responders, and the role of valve adjustments
Sarcopenia in atrial fibrillation: a prospective study
Multidimensional analysis of the association between metabolic abnormality combinations and depressive symptoms in overweight and obese populations: Cross-sectional evidence from two multinational cohorts
Systematic review and meta-analysis reveal positive therapeutic effects of music in brain damage rehabilitation
A randomized feasibility pilot trial of a remotely delivered physical activity intervention for adults with epilepsy
Digital biomarkers for brain health: passive and continuous assessment from wearable sensors
Nutrition effects on brain outcomes and recovery in stroke after hospitalization (NOURISH): Randomized controlled trial protocol and rationale
Effects of short-term bed rest on skeletal muscle function in patients with diabetes mellitus
A program of SUPPORT-DTM: Feasibility and acceptability of an early palliative care intervention for those living with dementia and caregivers
Peak Expiratory Flow and Its Trajectories Associated With Frailty in Older Adults: A Prospective Cohort Study

Obesity, metabolic health, and brain health: Insights from a prospective cohort study
Can we better prevent cardiovascular disease? 'Stayin' healthy to stay alive'
Artificial Intelligence‚ÄìEnabled Dementia Risk Prediction for Smart and Sustainable Healthcare: An Interpretable Machine Learning Study Using NHATS
Type 2 Diabetes Mellitus, Cognitive Performance, and Incident Dementia; Identifying Mediating Pathways and Biomarkers From the Plasma Proteome
Cognitive function and dementia risk factors among older people in nursing homes: An observational cohort study in Medan, Indonesia
Serum 25-hydroxyvitamin D concentrations, sleep characteristics and the risk of incident dementia
Analysis of Determinants Based on the Health Belief Model: A Study Predicting Cognitive Impairment Among Community-Dwelling Older Adults in China
Total energy expenditure and physical activity levels in functionally independent and limited older adults: A cross-sectional comparative study
Effects of Internet-Based Dementia Risk Reduction Education on Risk and Protective Factor Knowledge, Intentions, and Health Behaviors: Randomized Controlled Trial
The role of genomic-informed risk assessments in predicting dementia outcomes
Comparative efficacy of NIBS and Physical Exercise on cognitive function in patients with MCI or AD: a systematic review and meta-analysis
Mediating Role of Physical Capabilities in the Association Between Perceived Physical Fatigability and Cognitive Frailty in Older Adults
Association of neuroticism with incident dementia and cognitive function: 26-year follow-up of EPIC-Norfolk study
Exploring relationships between sleep quality and clinical depression in the Canadian Longitudinal Study on Aging
Cellular circadian period and its deviation associate with Alzheimer‚Äôs pathology and brain aging in cognitively impaired older adults
Cardiorespiratory fitness and risk of mental disorders and dementia: a systematic review and meta-analysis
Differential Improvement in Memory and Executive Functions: Personalized Exercise versus Acupuncture for Mild Cognitive Impairment in Stroke-prone Individuals
IGROOVE: Protocol for a randomized, controlled, single-blind trial of dance intervention in older adults with subjective cognitive decline
Individual and neighborhood socioeconomic inequality and the risk of dementia: A 14-year follow-up study
Cerebral small vessel disease unveils a vascular pathway to motoric cognitive risk in aging
Plasma Proteomic Signatures of Physical Activity Provide Insights into Biological Impacts and Its Protective Role Against Dementia
Combining cross-sectional and longitudinal genomic approaches to identify determinants of cognitive and physical decline
Association between hepatitis C virus infection and cognitive decline in seniors: further evidence from Taiwan
Inflammation as a mediator of air pollution's role in Alzheimer's disease and related dementias
Resistance training and subcortical vascular cognitive impairment: A 12-month randomized trial
The moderating effect of cognitive reserve on the association between neuroimaging biomarkers and cognition: A systematic review
Association between plasma p-tau181 levels and the evolution of cognitive and physical performance in a cohort of cognitively impaired older adults with frailty criteria: Longitudinal data from the CogFrail study
Atrial Fibrillation and Cognitive Decline: Mechanisms, Evidence, and Preventive Strategies‚ÄîA Narrative Review
Mortality following ground-level falls in trauma patients with dementia
Systematic review of the efficacy of pharmacological and non-pharmacological interventions for improving quality of life of people with dementia
Research on Threshold Optimization and Variability-Based Digital Biomarker Approaches Through MMSE-Lifelog Multimodal Integrated Analysis from a Clinical Screening Perspective
Molecular mechanisms and biomarkers in neurodegenerative disorders: a comprehensive review
Development and validation of a prediction model for postoperative delirium in elderly spinal patients: a retrospective case-control study
Sleep quality, APOE Œµ4, and Alzheimer‚Äôs disease: associations from two prospective cohort studies and mechanisms by plasma proteomic analysis
Physical activity interventions for hospitalised people living with dementia: systematic review and meta-analysis
The role of lifestyle in mitigating cognitive decline in older adults with cardiometabolic multimorbidity (CMM): A protocol of systematic review and meta-analysis
Validation status of cognitive digital assessments by the FDA BEST framework and context of use in preclinical AD studies: A systematic review
Effect of a home-based, dynamic, multidomain intervention on cognitive function in patients with cardiometabolic multimorbidity and their caregivers: The ‚ÄòLifeWise‚Äô randomized controlled trial protocol
Profile for Brain Disease Research Infrastructure for Data Gathering and Exploration (BRIDGE) Platform
Changes in Smoking Status in Depressed Patients and the Risk of Dementia
Risk factors and mental health comorbidities associated with subjective cognitive decline in Chinese older adults: A propensity score-matched case-control study
From Metabolism to Mind: The Cardio‚ÄìMetabolic‚ÄìBrain Axis and the Role of Insulin Resistance‚ÄîA Review
Community-based participatory research on Alzheimer's disease and related dementias among Tibetan Buddhist monks in South India: The Lifestyle Assessment and Monastic Aging Study (LAMAS)
Functional Decline and Loss of Independence After Traumatic Injury in Older Adults With Dementia
Mapping the oxidative stress metabolome in neurology by gas chromatography‚Äìmass spectrometry: a systematic review on signature-driven diagnosis and disease monitoring
Feasibility, safety, and effects of a step training program in community-dwelling older adults with mild-to-moderate dementia: A feasibility wait-list controlled trial
Evaluating the Effectiveness of 2024‚Äì2025 Seasonal mRNA-1273 Vaccination Against COVID-19-Related Hospitalizations and Medically Attended COVID-19 Among Adults Aged¬†‚â•¬†18¬†years in the United States: An Observational Matched Cohort Study
Cross-sectional associations of epigenetic clocks with intrinsic capacity and functional ability in older adults with frailty and cognitive impairment: the COGFRAIL study
Multimodal Autonomic Biomarkers Predict Phenoconversion in Pure Autonomic Failure

Genetic variation in TMEM106B alters microglial activation and cytokine responses in chronic traumatic encephalopathy
‚ÄòI Am No Longer Anxious When I Speak‚Äô: Experiences of People with Primary Progressive Aphasia Taking Part in a Biographic-Narrative Therapy (Cope PPA)
Burden of post-acute sequelae of COVID-19 in patients with type 2 diabetes: a binational cohort study in South Korea and Japan
Gender differences in quality of life and contributions of biopsychosocial factors
A narrative review of aging-targeted mechanisms and interventions in Alzheimer‚Äôs disease
Impact of Postoperative Chronic Back Pain Syndrome on the Incidence of Psychiatric and Brain Disorders, and All-Cause Mortality
‚ÄòSmall Steps‚Äô towards improving 24-hour time-use behaviours to decrease the risk of dementia: protocol for a personalised, web-based randomised controlled trial in community-dwelling older adults
Centenarians and near-centenarians in India: empirical insights on health and well-being characteristics
The economic burden of subjective cognitive decline, mild cognitive impairment and Alzheimer's dementia: excess costs and associated clinical and risk factors
Past Meal-Skipping Habits Associate With Physical Frailty in Later Life: A Retrospective Cohort Study
History of cannabis use and cognitive function in older adults: findings from the UK biobank
Challenges and opportunities for monitoring diet and physical activity in younger adults as part of a future brain health study: a UK and US survey
Promotion of muscle-strengthening exercise for public health: a systematic review of real-life interventions in healthy adults
The Impact of Fatigue on Health, Function, and Survival Between Ages 70 and 100
The relationship between plasma oxidized low-density lipoprotein and all-cause cognitive impairment: A population-based cross-sectional study in a rural area of Xi‚Äôan, China
A multi-modal medical management and lifestyle intervention increase cerebral blood flow and lowers diabetic risk in persons with early Alzheimer's disease: Mid-trial results from the PREVENTION trial
Cross-sectional associations between lifecourse dementia risk factors, plasma neurodegenerative biomarkers, and cognition in older adults in India
Retrospective analysis of neurofilament-light chain in patients with inflammatory bowel disease ‚Äì A pilot study
Associations between serum pigment epithelium-derived factor and physical performance in older women: The Otassha study
Cachexia risk classification using the Asian Working Group for cachexia criteria in patients with dysphagia: A retrospective cohort study
Gut microbiota and brain aging: a comparative review of African and western populations
Cycling and cognition in middle-aged and older adults: A scoping review
Exploring the relationship between Alzheimer‚Äôs disease and colorectal/breast cancers using SEER database, Mendelian randomization, and transcriptomic data
Metabolites and MRI-derived markers of dementia risk in a Puerto Rican cohort
Mapping brain change longitudinally: physical activity intervention reshapes structural alterations and functional connectivity in Alzheimer's disease
Dementia Risk According to Indices of Insulin Sensitivity and Beta-Cell Function in Individuals With Newly Diagnosed Type 2 Diabetes: A Cohort Study
Editorial: The early detection of neurodegenerative diseases: an aging perspective
Behavioral Rehabilitation of Swallowing Disorders in Patients with Neurocognitive Disorders
Living Longer With Purpose: Ikigai, Sleep, and Longevity
Association between personality profiles and motoric cognitive risk syndrome in community-dwelling older adults: a person-centered approach
Psychosocial hierarchies of modifiable risk for Alzheimer's disease: A networks analysis
Incidence and synergistic Association of Type 2 diabetes and apolipoprotein E epsilon 4 with dementia risk in the Kunshan aging research with E-health cohort study
Life activities of elderly patients with operated nonfunctional pituitary adenoma
Individual-Level Factors Associated With 10-Year Incidence of Alzheimer Disease and Related Dementias in the VA Million Veteran Program
A novel virtual reality-integrated multi-modal intervention for community-dwelling older adults with mild cognitive impairment: protocol for a randomized controlled trial
Kufor-Rakeb syndrome: a cohort-based clinical, imaging and genetic profile
Meaning in life and Parkinson's disease: Evidence from a 17-year study across 14 countries
Skeletal muscle adiposity and cognitive decline in a biracial cohort: Insights from coronary artery risk development in young adults (CARDIA) study
Relationship Between Comprehensive Assessment and Cognitive Decline in Older Adults: A Single-Center Cross-Sectional Study
A scalable step count-based predictor of biological age: development and validation of MoveIt! Age in community-dwelling adults and geriatric rehabilitation inpatients
Longitudinal online weekly reports of non-cognitive symptoms and events can differentiate incident mild cognitive impairment from stable cognitively healthy older adults
Organ-specific proteomic aging clocks predict disease and longevity across diverse populations
Older Adults, Anti-Amyloid Therapy, and Frailty: What Oncology Can Teach Us
Real-world prediction of early-onset dementia by health record data: A multi-center machine learning study
Social frailty and its association with cognitive trajectories in older adults: a prospective cohort study
Substitutions of Sleep, Physical Activity, and Sedentary Behaviour and the Risk of Dementia and Stroke
NORRISK 2 score is associated with dementia and MCI‚Äîthe HUNT study
Association of metabolic obesity phenotypes with cognitive decline in the ELSA-Brasil study
Preserved cognitive‚Äìfunctional phenotype in centenarians and its association with modifiable risk factors for dementia: Results from the Colombian centenarians project

Prevalence of intrinsic capacity decline and its association with activities of daily living among pre-frail and frail older adults in a community-based geriatric services hub model
The association of sarcopenia with surgical outcomes and complications following reverse total shoulder arthroplasty: a matched cohort study with comparative analysis against anatomic total shoulder arthroplasty
The Four-Square Step Test With and Without Dual Tasks Among Older Adults With and Without a Fall History: A Retrospective Cohort Study
The Relationships Between MASLD, Extrahepatic Multimorbidity, and All-Cause Mortality in the UK Biobank Cohort
Association of frailty with outcomes of patients undergoing transvenous lead removal for cardiac implantable electronic device infections in the United States
Association of weight-adjusted waist index with motoric cognitive risk syndrome in Chinese adults Aged ,â• 60 years
Birth cohort divergence in English and European multimorbidity trajectories
Late-onset unexplained epilepsy as a risk factor for cognitive impairment and dementia: Protocol for a multi-center prospective longitudinal observational study (ELUCID)
The impact of volunteering on cognition and cognitive decline in older diverse cohorts: KHANDLE and STAR
Mediterranean dietary pattern and risk of neurodegenerative diseases in a cohort of Swedish women
The long-term clinical course of non-surgically treated lumbar spinal stenosis: a systematic review and meta-analysis
Protective Effects of Socioeconomic Status and Lifestyle on Amyloid- and White Matter Hyperintensity-Related Longitudinal Brain Atrophy and Cognitive Decline
Activation of the AMPK Signaling and Brain Function: A Friend or Foe?
Food insecurity, food assistance, and physical and cognitive functioning among older Americans
Risk Factors and Annual Rates of Cognitive Decline in Elderly Mild Cognitive Impairment: A Retrospective Cohort Study
Frequency of daily conversation and risk of dementia: A population-based cohort study in Japan
Sex differences in the relationship between dynapenic abdominal obesity and the risk of motoric cognitive risk syndrome in older Chinese adults: A national prospective cohort study
Cognitive Reserve and Its Relationship With Memory Changes: An Analysis of the Survey of Health, Aging, and Retirement in Europe (SHARE)
How weather affects cognitive and physical outcomes in older adults
CardioMetAge estimates cardiometabolic aging and predicts disease outcomes
Bridging systemic metabolic dysfunction and Alzheimer,Äôs disease: the liver interface
Cardiometabolic indicators of cognitive impairment in the Cameron County Hispanic Cohort
Cardiovascular-kidney-metabolic syndrome, dementia risk, cognition, and neuroimaging outcomes
Wellbeing trajectories and dementia risk among Mexican-origin adults living in the U.S.
Risk factors associated with frailty and hypertension in older adults: a path analysis
Delineating white matter phenotypes of sensori-/psychomotor functioning in large-scale cohorts of healthy individuals and patients with mental disorders across the lifespan (whiteSPAN): rationale and methods of an interdisciplinary bicentric project
Characterizing Intervention Components and Complexity of Nonpharmacologic Healthcare Interventions to Manage Distress Behaviors in Older Adults
Interplay of diet, nutritional status, and psychological well-being in mild cognitive impairment among urban elders in Hyderabad, India: a cross-sectional study
The interactive effects of low testosterone and sleep insufficiency on cognitive impairment: Evidence from the NHANES 2011,Äì2014
Social Asymmetry and Risk of Morbidity and Mortality
Asymmetric hearing loss and the risk of stroke: evidence from a longitudinal cohort study
Exploratory functional and quality of life outcomes with daily consumption of the ketone ester bis-octanoyl (R)-1,3-butanediol in healthy older adults: a randomized, parallel arm, double-blind, placebo-controlled study
Strategies for recruiting participants underrepresented in clinical research: A scoping review
Prognostic Value of Low Alanine Aminotransferase in Patients Undergoing Transcatheter Aortic Valve Replacement
Exploring the knowledge, attitudes, and practices of physical therapists in care facilities assisting individuals with Alzheimer's disease
Association of oral health and chewing ability with cognitive impairment and dementia in older adults: Longitudinal findings from a national panel study
Exploring the effect of pre-clinical Alzheimer,Äôs disease on blood pressure using Mendelian randomisation and parental dementia as an instrumental variable in UK Biobank
High-sensitivity cardiac troponin I and risk of dementia: the 25-year longitudinal Whitehall II study
Green but grey: green areas, air pollution, and children,Äôs cognitive development in Santiago, Chile
Association of Modifiable Risk Factors Measured With the Brain Care Score and Incident Stroke in the REGARDS Cohort
Book of abstracts of the 6th CHRC Annual Summit
Safety of Minimally Invasive Sublobar Pulmonary Metastasectomy in the Frail Patient
Effectiveness of nature-based group intervention in loneliness and health-related quality-of-life in lonely older adults living in assisted living facilities,Äîa randomised controlled trial
Associations of early-life factors and risks of incident dementia: A cohort study of 247,841 participants
The Intersection of Physical Inactivity, Dementia, and Gender: A Population-Based Study of All-Cause Mortality in Middle-Aged and Older Adults in Korea
Impact of ethnicity and antihyperglycemic medications on dementia incidence in older adults with type 2 diabetes
Sensorimotor Impairment and Incident Dementia in the US Medicare Beneficiaries
Exploring the Role of a Dietitian in an Early Intervention Program for People With Dementia and Their Care Partners: A Composite Case Series
Advances in Research on Brain Health and Dementia: Prevention and Early Detection of Cognitive Decline and Dementia: Series II

Development and validation of a prediction model for long-term cognitive frailty risk in stroke patients based on CHARLS data
QT Interval Prolongation and Dementia in a General Japanese Population: the Hisayama Study
Associations of healthy lifestyle and genetic susceptibility with risks of osteoarthritis: A prospective cohort study
Promoting activity, independence and stability in early dementia and mild cognitive impairment: the PrAISED research programme including an RCT
Mendelian randomization reveals that Alzheimer‚Äôs disease, Lewy body dementia, frontotemporal dementia, and vascular dementia affect human lifespan and mediate the impact of modifiable risk factors on lifespan
Physical activity attenuates the association between allostatic load and early Alzheimer's disease-related biomarkers in older adults
The impact of bedtime alignment on sleep health in older couples: gender-sensitive analysis
Reproductive Lifespan and Reproductive Factors in Relation to Dementia Risk in Postmenopausal Women With Type 2 Diabetes
Association between oral health and physio-cognitive decline syndrome of older adults in China and its sex differences: a cross-sectional study
The mediating role of biological age in the impact of mood instability symptoms on neurodegenerative disease and mortality
Phase II pilot randomized trial of zonisamide for disease modification in prodromal Lewy body disease
The role of sleep in dementia prevention: building the case for causality
Association of ultra-processed food consumption with prodromal, incident Parkinson‚Äôs disease and mortality
Faster but less accurate: An explorative study on the effects of three weeks of ketogenic diet on cognitive functions in undergraduate students
Aging, dementia, and care models: Global perspectives with insights from India
Associations between neuropsychiatric symptoms and motoric cognitive risk syndrome
Effects of Tai Chi and conventional exercise on improving cognitive performance in older adults with mild cognitive impairment: Study protocol for a randomized controlled trial
Detection of balance in the elderly under the influence of stress (DEPIE): A cross-sectional study protocol
Development and Validation of a Machine Learning Model for Predicting Osteoporosis in Patients with Parkinson‚Äôs Disease
The Effects of 8-Week Creatine Hydrochloride and Creatine Ethyl Ester Supplementation on Cognition, Clinical Outcomes, and Brain Creatine Levels in Perimenopausal and Menopausal Women (CONCRET-MENOPA): A Randomized Controlled Trial
Healthcare utilization and chronic condition clusters in multimorbidity patients using weighted k-means: a register-based study in Denmark
Digital technology use and dementia among older adults: the moderating role of activity limitations
Circulating neurodegenerative brain injury markers and hip fracture and fall hospitalizations: the Cardiovascular Health Study
The Potential of Focal Muscle Vibration Therapy in the Management of Parkinson‚Äôs Disease: A Systematic Review
Activity Engagement Across Stages of Cognitive Health
Assessment of Risk Factors for Cognitive Impairment Accounting for Genetic and Environmental Influences: An Italian Population-Based Twin Study
The relationship between air pollution and dementia in mood disorders: The moderating role of sleep patterns‚Äî‚Äî A prospective study from the UK Biobank
Comparing the Relationship Between Social Determinants of Health and Frailty Status of Medicare Beneficiaries in Rural and Urban Areas in the United States
Consequences of closed head injury: a systematic review
Sex differences in allostatic load profiles and incident dementia: The AGES-Reykjavik Study
Racial and ethnic differences in the impact of air pollution on the risk of Alzheimer‚Äôs disease and related dementias in the Multiethnic Cohort Study
Longitudinal sex differences in cerebrovascular ageing in older adults: results from the brain in motion study
Low health literacy limits behavioral changes during phase I cardiac rehabilitation: a multicenter clinical study
Maternal Age at Childbirth and Offspring Cognitive Function in Middle and Older Age: A Longitudinal Cohort Study
Association between sedentary behavior and dementia: a systematic review and meta-analysis of cohort studies
Association between high carbohydrate to dietary fiber ratio and risk of dementia in older adults: analysis from the UK biobank
Right ventricular function in pulmonary hypertension and obesity: a cross-sectional cohort study with survival follow-up
Carotid and Vertebral Flow Velocities: Relationships With Cognitive Function in Wisconsin Native American Population
Physical activity linked to slower tau protein accumulation and cognitive decline
Individual and environmental stressors in life course cognitive health disparities: Evidence from the Dutch Lifelines Cohort Study
Clinical biomarker potential of spectrin breakdown degradation products: a systematic review
Understanding Cerebral Blood Flow Dynamics for Alzheimer's Disease Prevention Through Acute Exercise (flADex): Protocol for a Randomized Crossover Trial
Multi-dimensional social frailty index for predicting disability and mortality in community-dwelling older adults: JAGES cohort
Retinal microvascular alterations in motoric cognitive risk syndrome: an OCT angiography study
Multilingualism protects against accelerated aging in cross-sectional and longitudinal analyses of 27 European countries
Cognitive frailty and pre-cognitive frailty in Chinese middle-aged and older adults: prevalence, risk factors, mortality
RADAR-AD: assessment of multiple remote monitoring technologies for early detection of Alzheimer‚Äôs disease
Combined effects of particulate and gaseous pollutants on Alzheimer's disease-related brain imaging markers and cognitive function
Intra-individual variability in cognitive performance predicts falls in older adults with chronic stroke
Predicting dementia severity changes after shunt surgery for idiopathic normal-pressure hydrocephalus: Role of the tap test and cognitive assessments
Apathy Is Associated with Slower Gait and Subjective Cognitive Complaints in a South Indian Community-Dwelling Cohort
When the clock shifts: menopause timing is associated with reduced cognitive performance and gray matter volume in a population-based cohort

Evaluating sex and gender as separate and interactive predictors of memory aging trajectory classes: an integrative data-driven approach
Effectiveness of technology-assisted nonpharmacological interventions for cognitive impairment in older adults: A systematic review and network meta-analysis
Metabolic dysfunction-associated steatotic liver disease and the longitudinal progression of atrial fibrillation: a prospective study
Epigenetic Clocks of Biological Aging and Risk of Incident Mild Cognitive Impairment and Dementia: The Women's Health Initiative Memory Study
Associations of life course social determinants of health, social mobility with cognitive function and roles of social participation and lifestyle: a multicohort study
Polygenic risk for white matter hyperintensities is associated with early cerebrovascular events partly through hemodynamic measures in cognitively unimpaired middle-aged and older adults with low cardiovascular risk
A hypothetical intervention analysis for the effects of healthy dietary patterns on reducing major chronic diseases and mortality associated with air pollutant mixtures
Hot topics in valvular heart disease and dyslipedaemias
Long-Term Urban Air Pollution Drives Multi-Stage Neuropsychiatric Disorder Trajectories: A Prospective Cohort Study
Circulating Biomarkers of Neurodegeneration and All-Cause and Dementia-Specific Mortality
Gait speed and muscle mass as dual mediators in the link between white matter hyperintensities and Alzheimer's disease
Mutual effects of sleep duration and traits on the risk of dementia: A prospective study in the UK Biobank
Group-based trajectory modelling for cognitive changes in middle-aged and older adults: A systematic review
Relation of frailty and polypharmacy on cognitive impairment in Chilean Older Adults
Monitoring of wheelchair use in long-term memory care units
Right Ventricular Function and Its Association With Outcomes in Advanced Heart Failure
Correction: Hearing impairment and dementia: cause, catalyst or consequence? (Journal of Neurology, (2025), 272, 6, (402), 10.1007/s00415-025-13140-x)
Geriatric syndromes in intestinal failure: An accelerated aging phenomenon in middle adulthood patients?
How prevalent are modifiable dementia risk factors in Ireland? A 12-year observational study in community-dwelling older adults
Cardiovascular risk factors, aging, and incidence of dementia (CAIDE) risk score and its association with cognitive performance and volumetric brain measures in mild cognitive impairment
Utilizing Perceived Fatigability to Identify Cognitive and Mobility Impairments in Aging and Multiple Sclerosis
Weekend warriors and preventing crises
Impact of Frailty on Outcomes Following Percutaneous Left Atrial Appendage Occlusion: A Propensity-Score Matched Analysis
Nuts4Brain-RCT: Protocol for a Three-Arm Randomized Controlled Trial Examining the Dose‚ÄìResponse Effects of Nut Consumption on Mental Health in Young Adults
Effect of CPAP treatment on subjective cognitive decline in patients with mild obstructive sleep apnea syndrome
Plasma fatty acids levels and risk of schizophrenia in the UK Biobank: a prospective cohort study
Association of maximal tongue pressure with dysphagia and prognosis in patients with acute heart failure
The frequency of adding salt to food and incident neuropsychiatric disorders: a large prospective cohort study of 426,832 participants
Metabolic dysfunction-associated steatotic liver disease, metabolic alcohol-related liver disease, and incident dementia: a nationwide cohort study: MASLD, MetALD, and dementia risk
Longitudinal MRI study of hippocampal subfields Morphometry in early Parkinson's disease
Prevalence and Characteristics of Prehabilitation Utilization Among Older Medicare Beneficiaries Undergoing Elective Inpatient Lower Extremity Joint Replacement
Development of machine learning models for prediction of current and future dementia
Antipsychotic use and dysphagia risk in acute heart failure: a prospective cohort study
Acute exercise alters brain glucose metabolism in aging and Alzheimer's disease
Dietary inflammatory potential and depression in middle-aged and older adults: A population-based study of associations and comorbid disease network
Interventions for promoting physical activity and reducing sedentary time among people aged 55 years and older: an overview of systematic reviews
Long-term outcomes of two-stage revision with positive cultures at reimplantation
Racial disparities in the association of epigenetic age acceleration with the transition between normal cognition, mild cognitive impairment and dementia
Association between wearable device-measured weekend catch-up sleep and brain health in UK Biobank participants
Musical intervention to reduce stress during botulinum toxin injection for spasticity: Protocol for a randomized controlled trial (MUSIBOT)
Chinese guidelines for diagnosis and treatment of intracranial atherosclerotic stenosis
Physical Health and Cognitive Outcomes in Elderly School Members: Insights from a Cross-Sectional Study
Association Between Cognitive and Physical Functions in Patients Undergoing Haemodialysis
Mapping urban emotion from geotagged social media data: Age, gender and spatial heterogeneity
Standardised and systematic sampling of public engagement in brain health ‚Äí Slovenian example
A Non-Randomized Pilot Trial of Brain-WISE: A Group-Based Program for Brain Health and Dementia Risk Reduction in Community Settings
Recent Articles‚ÄîSkeletal Muscle and Other Topics in Diabetes
Personality and cognitive resilience to accelerated epigenetic aging
ROLE OF AGEING IN ALZHEIMER‚ÄôS THERAPEUTICS
Non-pharmacological measures for sleep problems in dementia: Time to forget sleeping pills?
The association between depression, anxiety, sleep disturbances and dementia: a prospective cohort study of 350,186 adults

Postoperative cognitive dysfunction and neurodegeneration: From inflammation to precision medicine
An assessment of intrinsic capacity among older Indian adults from the Longitudinal Ageing Study in India
Commentary: The Effect of Stress on All-Cause Dementia: A Longitudinal Analysis from the Australian Longitudinal Study on Women,Äôs Health
The impact of cognitive impairment and low muscle mass on all-cause mortality among older adults in China: An empirical analysis based on CLHLS cohort data
Long-term air pollution exposure and cognitive impairment risk in Chinese middle-aged and older adults
Meta-analysis of risk factors related to secondary fracture in elderly patients with hip fractures
Palliative care in emergency departments could improve patient care and reduce medical costs
Endogenous Œ≤-hydroxybutyrate and the risk of cognitive decline: a nested case-control study in the UK Biobank cohort
Unequal expression: Social position modulates APOE genotype risk of dementia
Ambient air pollution exposure accelerates the occurrence of 78 non-communicable chronic diseases: an accelerated failure time analysis of a nationwide cohort
Feasibility of the MAINTAIN intervention to support independence after a fall for people with dementia: a pilot cluster randomised controlled trial in participants,Äô own homes
Cognitive Level Enhancement through Vision Exams and Refraction (CLEVER): study protocol for a randomised controlled trial
Oral health conditions and nutritional, functional and cognitive status - An approach to the institutionalized older adult
The Impact of Frailty, Activity of Daily Living, and Malnutrition on Mortality in Older Adults with Cognitive Impairment and Dementia
Association between insomnia and its symptoms and cognitive impairment in community-dwelling older adults in China: A multicenter study
Identifying modifiable factors and their joint effect on frailty: a large population-based prospective cohort study
Physical inactivity, depressive symptoms, and progression to sarcopenia in older adults: a 4-year longitudinal study
Establishing the TSH reference intervals for healthy adults aged over 70 years: The Australian ASPREE cohort study
Physical activity and cognition in older adults: research hotspot and topic evolution
How to Break Stubborn Association Between Hearing Loss and Cognitive Impairment: A Systematic Review and Meta-Analysis of Moderators and Mediators
Ultra-processed food intake and brain health in middle-aged and older adults
Use of the Brain Care Score to Estimate the Risk of Incident Cerebrovascular Events in Middle-Aged Women
Rehabilitation Protocols for Surgically Treated Acetabular Fractures in Older Adults: Current Practices and Outcomes
Biological age acceleration mediates effects of household air pollution from solid fuels on dementia risk: A prospective cohort study in China
How Do Occupational Sedentary Behavior and Occupational Cognitive Complexity Relate to Cognitive Function? A Cross-Sectional Study
Differential physical activity patterns in cognitively impaired vs healthy older adults using accelerometers
Cognitive leisure and dementia risk: Evidence beyond reverse causation
Biological, Psychosocial, and Microbial Determinants of Childhood-Onset Obsessive,ÄìCompulsive Disorder: A Narrative Review
Genetic association and potential mediators between subjective well-being and cardiovascular events: A bidirectional two-sample, two-step Mendelian randomization study
Television watching and cognitive outcomes in adults and older adults: A systematic review and dose-response meta-analysis of observational studies
Influence of Sociodemographic Variables on the Lifestyle of the Adult Population: A Multicenter Observational Study
Estimated Cardiorespiratory Fitness and Grip Strength: Independent and Combined Associations With Multimorbidity Risk and Patterns in a Large-Scale Prospective Cohort Study
Proteomic signatures of grip strength and healthy aging among older adults: A prospective cohort study in the UK Biobank
Years of education and dementia risk in community-dwelling Japanese adults
Association of Alzheimer's disease concerns with amyloid burden and lifestyle behaviors in cognitively unimpaired older adults
Mortality and ocular parameters and diseases
Association between inflammatory biomarkers and the cognitive response to a multidomain intervention: secondary longitudinal analyses from the MAPT study
Construction of a nomogram prediction model for Alzheimer's disease among the elderly in community
Extending the prevention paradigm for cardiometabolic diseases to target dementia through multiple low-risk lifestyle behaviors: a story of adherence
Comparison of multiple quantitative strategies for neuropathologic image analyses
Association between early retirement and risk of frailty in later life: A population-based longitudinal study
Joint association of cognitive reserve and frailty with incident Alzheimer's disease and related dementias
Low intrinsic capacity is associated with a blunted exercise-induced cognitive enhancement in physically active middle-aged and older adults
Association of cystatin C kidney function measures with motoric cognitive risk syndrome: evidence from two cohort studies
Survival predictors of older cancer patients in Bangladesh: A multicenter study
Therapeutic potential of exercise-hormone irisin in Alzheimer's disease
The Accessible Vascular Indicators for Mild Cognitive Impairment Detection: The Predictive Value of the Ankle-Brachial Index
Comment on: Change in physical activity and the risk of depressive symptoms in mid-older aged adults
Effect of Social Activity on All-Cause Dementia Among South Korean Elders: A Retrospective Cohort Study
Changes in senescence markers after a weight loss intervention in older adults with obesity
Associations of loneliness and depression with increased risks of incident dementia: A prospective study from three older adult cohorts
Living in a 20-min neighborhood and brain health and resilience in older adults: The Healthy Brain Initiative

Region-Based Analysis with Functional Annotation Identifies Genes Associated with Cognitive Function in South Asians from India
Examining the Effect of SNAP-Multibehaviours on Multimorbidity Risk: A Cross-Sectional Study in Three General Practices,Äô Electronic Health Records
Development of a midlife-specific CogDrisk algorithm (CogDrisk-ML) to enable validated implementation of dementia risk assessment from midlife to late life
Association of neighborhood physical environment with falls and fear of falling in older adults: A prospective cohort study
The effect of social exclusion on the cognitive health of middle-aged and older adults: A systematic review
Calcium supplementation and the risk of dementia in the Perth Longitudinal Study of Aging Women: a post-hoc analysis of a randomised clinical trial for fracture prevention
The relationship between trajectories of serum lipids and cognitive impairment in middle aged and elderly: A 6-year prospective cohort study in Xi'an, China
The cardiometabolic "bottleneck" on cognitive function in aging
Handgrip Strength, a Component of Physical Frailty Contributing Significantly To Cognitive Impairment: Evidence from LASI Wave 1 (2017-18)
Frailty and social contact with dementia risk: A prospective cohort study
The missing cohort: Forcibly displaced populations with disability in dementia research and policy
Assessing diet in epidemiologic studies
Effects of divided attention on kinematic adaptations during obstacle-crossing in mild cognitive impairment
Comprehensive intervention combining group and personalized language therapy in primary progressive aphasia: Quantitative and qualitative findings
Sex differences in modifiable dementia risk factors: Findings from the Rush Memory and Aging Project
The Association of Niacin Use with Kidney Outcomes and Mortality
Functional Near-Infrared Spectroscopy Signal as a Potential Biomarker for White Matter Hyperintensity Progression in Patients With Subcortical Vascular Cognitive Impairment: A Pilot Study
Self reported hypoglycemia in patients with type 2 diabetes mellitus taking oral anti-diabetics
Moving Towards a Medicine of Dance: A Scoping Review of Characteristics of Dance Interventions Targeting Older Adults and a Theoretical Framework
Deep Learning based Gait Analysis for Early Detection and Progression Monitoring of Alzheimer's Disease
Prevalence and Associated Factors of Chronic Depression Among Older Adults: A Systematic Review, Meta-Analysis, and Meta-Regression
Memantine-Associated Decrease in Renal Function and Hyperuricemia
Associations of Frailty Status and Sleep Quality With Incident Delirium: A Prospective Study in the UK Biobank
Impact of Physical and Cognitive Frailty on Long-Term Mortality in Older Patients undergoing Elective Non-cardiac Surgery
Data-driven discovery of midlife cardiometabolic profile associated with incident early-onset and late-onset dementia
Association of Early- to Midlife Weight Trajectories With Mid- to Late-Life Cognitive Decline in the ELSA-Brasil Study
Associations of Objective and Subjective Physical Function With Quality of Life and Well-Being: A Cross-Sectional Study From Ardakan Cohort Study on Aging (ACSA)
Association of dietary macronutrients with MRI-detected vascular brain injury and cognition in 9886 middle-aged participants from four countries: for the Canadian Alliance of Healthy Hearts and Minds (CAHHM) and the Prospective Urban Rural Epidemiological (PURE) Study Investigators
Cognitive cerebellum dominates motor cerebellum in functional decline of older adults with mild cognitive impairment
Magnesium Depletion Score as a Novel Predictor of Cognitive Impairment: A Population-Based Cross-Sectional Study From NHANES
Nut consumption is associated with a lower risk of all-cause dementia in adults: a community-based cohort study from the UK Biobank
Reducing the burden of informal care through lecanemab: A case of a patient with mild cognitive impairment who continues to drive
Addressing global diversity in dementia research with the COSMIC collaboration
Determinants of limitations in activities of daily living among community-dwelling persons aged 80 years and older: Longitudinal national evidence from the Health, Aging and Retirement in Thailand study, 2015,Äì2022
The association between primary ovarian insufficiency and increased multimorbidity in a large prospective cohort (Canadian Longitudinal Study on Aging)
Primary Prevention of Fragility Hip Fracture in High-Risk Elderly by Village Health Volunteers in Nan Province, Thailand
Anthropometric metabolic subtypes and health outcomes: A data-driven cluster analysis
Physical activity modifies associations between cerebrospinal fluid tau measures and executive function
Age-stratified validation of the performance index (P-Index) as a metric for dual-task cost in functional mobility across the adult lifespan
Adherence to life's essential 8 is associated with delayed white matter aging
The efficacy, mechanisms and implementation of physical activity as an adjunctive treatment in mental disorders: a meta-review of outcomes, neurobiology and key determinants
EPIC-Potsdam sub-cohort study: The early role of trace elements, oxidative stress, and anthropometrics in Alzheimer's disease and dementia onset
Exploring the association between physical activity and cognitive function among people living with dementia
APOE Œµ4-independent pathways predominate in the association between retinal nerve fiber layer thinning and 12-year incident dementia
Motoric Cognitive Risk Syndrome Associated With Risk of Frailty and Likelihood of Reversion in Older Adults
Pharmacist intervention for polypharmacy improves activities of daily living in older patients with musculoskeletal disorders: A multicenter retrospective cohort study using propensity score matching
Effect of Partial Foot Amputation Level on Gait Independence in Patients With Chronic Lower Extremity Wounds: A Retrospective Analysis of a Japanese Multicenter Database
Comment on ‚ÄúRelationships between muscle strength, lung function, and cognitive function in Chinese middle-aged and older adults: A study based on the China health and retirement longitudinal study (CHARLS),Äù: Subtitle: Muscle strength, lung function, and cognitive function.
Accelerated retinal ageing and multimorbidity in middle-aged and older adults

Cancer Screenings, Cognitive Rehabilitation, Morton Neuroma, Wearable Smart Devices, Falls in Older Adults, Obsessive-Compulsive Disorder
Sarcopenia Is Poorly Documented in Geriatric Rehabilitation Inpatients: Restoring Health of Acutely Unwell Adults (RESORT)
DunedinPACNI estimates the longitudinal Pace of Aging from a single brain image to track health and disease
Systolic blood pressure variability: risk of cardiovascular events, chronic kidney disease, dementia, and death
Circulating Biomarkers of Neurodegeneration and Risk of Cognitive Impairment
Association Between Lifestyle at Different Life Periods and Brain Integrity in Older Adults
Insights into dentatorubral-pallidoluysian atrophy from a new Drosophila model of disease
Physical Activity and Neurocognitive Symptoms in Older Adults During COVID-19 Pandemic
Association between trajectories of life-course group sports participation and dementia: A 3-year longitudinal study
A comprehensive investigation to examine the factors associated with previous falls, recurrent falls, and concerns about falling among outpatient older individuals: a cross-sectional study
Association between Metabolic Syndrome and Young-Onset Dementia: A Nationwide Population-Based Study
A lifespan perspective on cognitive reserve and risk for dementia
Comparative effects of different physical exercises on cognitive function and intervention adherence in older adults with cognitive impairment: A systematic review and network meta-analysis
The promotion of physical activity for use in Alzheimer's disease prevention trials in adults with Down syndrome: Results from a 12-month randomized trial
Plant and Animal Protein Intake and Transitions From Multimorbidity to Frailty and Mortality in Older Adults
Aerobic training versus mobile application based cognitive training: impact on cognition, aerobic capacity, and quality of life of older adults ,Äì An experimental study
Annual level changes of serum neuronal and glial biomarkers in a German professional football club
More fit KL-VS heterozygotes have more favorable AD-relevant biomarker profiles
Trajectories of general and central obesity beyond middle age in relation to late-life cognitive decline and dementia
Association of environmental exposome and cognitive function among older adults with and without preclinical Alzheimer's disease
Predicting Mortality in Older Adults Using Comprehensive Geriatric Assessment: A Comparative Study of Traditional Statistics and Machine Learning Approaches
Psychological well-being trajectories preceding incident mild cognitive impairment and dementia
Synergistic effects of exercise, cognitive training and vitamin D on gait performance and falls in mild cognitive impairment - Secondary outcomes from the SYNERGIC trial
Plasma proteomic signatures of dual cognitive and mobility decline in older adults
Misfolded Œ±-synuclein co-occurrence with Alzheimer's disease proteinopathy
Beyond diabetes: Testing long-term metformin adherence for dementia prevention
Management of Patients With Mild Cognitive Impairment in the Era of Anti-Amyloid Therapy A Worldwide Neurology Survey
Associations of the LIBRA index with cognitive resilience to genetic susceptibility to dementia
Changes in anticholinergic cognitive burden and risk of single and recurrent falls: Population-based cohort study
Two cases illustrating different neurological aspects of HIV-escape syndrome
Hospitalization-Associated Disability and 1-Year Mortality Risk in Older Patients With Heart Failure
Among 69,178 UK Residents Ages 65+ Years, Frailty Associates Significantly With Lifestyle Behaviors and Depression: A Cross-Sectional Study
Is waist to height ratio better at assessing cause-specific mortality risk than body mass index or waist circumference? A prospective analysis in a large U.S.-based cohort
Dietary inflammatory potential and the risk of cognitive impairment: A meta-analysis of prospective cohort studies
Prevalence and Misperception: Exploring the Gap Between Objective and Subjective Assessment of Sleep Apnea in a Population at Increased Risk for Dementia
Association of Cardiovascular Autonomic Failure With Progression and Phenoconversion in Isolated REM Sleep Behavior Disorder
Contribution of depression and cardiometabolic diseases and the role of depression treatment in survival and functioning in older adults
Bidirectional Associations Between Urinary Incontinence and Poor Mental Health Among Middle-Aged and Older Adults in Thailand
Cerebral small vessel disease and its relationship with all-cause mortality risk: Results from the Amsterdam Ageing cohort
Cohort Profile: The Nijmegen Exercise Study (NES)
Apathy, gait slowness, and executive dysfunction (AGED) triad: opportunities to predict and delay dementia onset
Plasma Proteins Associated with the Mediterranean-Dietary Approaches to Stop Hypertension Intervention for Neurodegenerative Delay (MIND) Diet and Incident Dementia
Diffusion Tensor Imaging Along the Perivascular Space (DTI-ALPS)
Blood pressure and all-cause mortality in dementia: a nationwide cohort study
Microstructural Brain Changes Associated With Frailty in Older Adults: Insights From Diffusion Tensor Imaging
Spontaneous remission of dropped head syndrome following short-term bed rest in acute encephalopathy: a case report
Effects of exercise on cognition and Alzheimer's biomarkers in a randomized controlled trial of adults with mild cognitive impairment: The EXERT study
Rapid recommendations: Updates from 2024 guidelines: part 3
Change in D3Cr muscle mass in oldest old men and its association with changes in grip strength and walking speed
Quadratic associations between sleep and multimorbidity among the older population in China: Evidence from CLHLS 2011 to 2018
Days at home after treatment of spinal metastases: measurement and validation of a novel patient centered outcome

Older Amyloid Beta as a Candidate Blood Biomarker of Early Cognitive Decline in the Elderly,ÄîA Preliminary Study
Functional and radiological outcomes of percutaneous wiring for two and three part fractures of the proximal humerus
Combined healthy lifestyle behaviors and all-cause mortality risk in middle-aged and older US adults: A longitudinal cohort study
Letter to the Editor: Effect of Social Activity on All-Cause Dementia Among South Korean Elders: A Retrospective Cohort Study
Adult-onset metachromatic leukodystrophy: a novel genotype with a distinct phenotype
Community-based going-out program and dementia onset in Japanese older adults: A longitudinal observational study
Exploring the Dietary Patterns and Health Behaviours of Centenarians in Ourense (Spain): Adherence to the Southern European Atlantic Diet
Mediterranean-DASH Intervention for Neurodegenerative Delay (MIND) Trial: Genetic Resource for Precision Nutrition
Relationship between development trajectories of leisure activity and sleep time on incident cognitive impairment: A study based on the Chinese Longitudinal Healthy Longevity Survey (CLHLS)
The arrhythmia of bodily urgency: Using rhythmanalysis to understand the organisation of care people living with dementia experience within acute hospital wards
Trajectory of Cognitive Function after Incident Heart Failure
The impact of estimated cardiorespiratory fitness on Alzheimer's disease biomarkers and their relationships with cognitive decline
Changes in Loneliness, Social Isolation, and Social Support: A Gender-Disaggregated Analysis of Their Associations With Dementia and Cognitive Decline in Older Adults
Advancing fall risk prediction in older adults with cognitive frailty: A machine learning approach using 2-year clinical data
Metabolic Dysfunction-Associated Steatotic Liver Disease Is Associated With Accelerated Brain Ageing: A Population-Based Study
The Association Between Sensory Impairment and Cognitive Impairment Among Older Adults: Insights From a National Cohort Study in China
Effect of cognitive reserve on cognitive function and cognitive deterioration in Parkinson's disease: a longitudinal cohort study
Association of mid-age Life's Essential 8 score with digital cognitive performance and incident Alzheimer's disease: The Framingham Heart Study
Daily fluid intake and brain amyloid deposition: A cohort study
Adherence to intervention components: The key to success? Analysis on health-related outcomes of the AgeWell.de intervention to preserve cognition
SHIP-AGE: Frailty, renal function, and multi-component primary care in rural Mecklenburg-West Pomerania (MV-FIT)study protocol
Association of body age and multiorgan ages with cognitive decline in older adults: a nationwide longitudinal study
Health literacy in relation to web-based measurement of cognitive function in the home: UK Women's Cohort Study
Neurodegenerative Disease and Association Football (NDAF): Systematic Review and Meta-Analysis
Association of Cardiac Biomarkers With Structural Brain Changes and Cognitive Impairment: Results From the Hamburg City Health Study
The impacts of multimorbidity trajectories and patterns on functional limitations over time in middle-aged and older adults
Objective physical activity and Alzheimer's disease burden in the population-based Rotterdam Study
Independent and combined effects of depressive symptoms and cardiometabolic risk factors on dementia incidence: a cross-country comparison in England, the United States and China
Primary care insights on the management of diabetes: Results from a mixed method study of care changes and impacts during the COVID-19 pandemic
Determinants of Brain Atrophy in People Living with HIV: The Role of Lifestyle, Demographics, and Comorbidities
Effect of combined aerobic exercise and cognitive training in older adults with post,Äëstroke cognitive impairment
The trajectories of depression and multimorbidity affect the patterns of cognitive decline: a prospective cohort study
Racial Inequities in Cognitive Decline of Middle-Aged and Older Adults: Findings of the ELSA-Brasil Study
Lifestyle, genetic risk, plasma pTau217, and incident cognitive impairment in WRAP
The adaptation and evaluation of a culturally grounded lifestyle intervention to mitigate the risk of Alzheimer's disease and related dementias in Native Hawaiians and Pacific Islanders: A study protocol
Database Analysis of Application Areas and Global Trends in Ketogenic Diets from 2019 to 2024
Cardiovascular health and its association with dementia, Parkinson's Disease, and mortality among UK older adults
Association of a DASH diet and magnetoencephalography in dementia-free adults with different risk levels of Alzheimer,Äôs disease
A Qualitative Study of Factors Influencing Continued Participation in a One-Year Dementia Prevention Class for Community-Dwelling Older Adults
Mortality Outcomes in a Large Population with and without Covert Cerebrovascular Disease
Constipation preceding stroke, dementia and Parkinson's disease in middle-aged and older adults: A population-based cohort study
Effects of exercise versus usual care on older adults with amnestic mild cognitive impairment: EXERT versus ADNI
Neuroticism is associated with future disease and mortality risks
Brain health concerns in former rugby players: clinical and cognitive phenotypes
Impact of pharmacological and non-pharmacological strategies on postoperative cognitive disfunction (POCD) in cardiac surgery
Dietary changes following a lifestyle-based intervention for dementia risk reduction ,Äì results from the AgeWell.de study
Through the Center of Cardiovascular Research: My Journey with Big Data and Bioengineering: The 2024 J. Earl Wynands Lecture (Society of Cardiovascular Anesthesiologists)
Longitudinal associations between lipid panel and cognitive decline modified by APOE 4 carrier status in biracial community-dwelling older adults: Findings from the Chicago health and aging project
Identifying lifestyle risk factors that discriminate memory trajectories: Differential findings across racial and ethnic groups in older adults
Comment on: Changes in Leisure Activity, All-Cause Mortality, and Functional Disability in Older Japanese Adults: The JAGES Cohort Study
Dance-like state detection, curiosity, and decentering: A pilot study on artful movement and mindfulness

Association between cardiovascular and cognitive health among older Indigenous Africans: Data from an urban Nigerian settlement participating in the VALIANT study
White Matter Hyperintensities Are Associated with Slower Gait Speed in Older Adults without Dementia
Prevalence of malnutrition and risk of malnutrition in complex chronic outpatients
Modifiable risk factor profiles moderate the effect of Œ≤-amyloid pathology on cognition in aging
The Brain Care Score and its associations with cardiovascular disease and cancer
Risk of suicide and all-cause death in patients with mental disorders: a nationwide cohort study
Breaking prolonged sitting with high-intensity interval training to improve cognitive and brain health in middle-aged and older adults: A protocol for the pilot feasibility HIIT2SITLess trial
Living arrangements and chronic disease accumulation among native-born and immigrant older adults in Europe
Progranulin Mutation Manifesting as Parkinson Disease: A Case Series from the PADUA-CESNE Cohort
Exploring Older Adults,Äô Pre-Intervention Motivations, Attitudes, Expectations, and Barriers to Participation in the FINGER-NL Lifestyle Intervention to Maintain Optimal Cognitive Functioning: A Qualitative Interview Study
Association between walking speed and multimorbidity patterns in community-dwelling older adults with diabetes and/or hypertension: a latent class analysis
Associated Factors of Cognitive Frailty in People Living with HIV Aged 50 and Older: A Cross-Sectional Study
Associations between the 24-h Activity Daily Cycle and Incident Dementia
Post-COVID-19 Condition Prediction in Hospitalised Cancer Patients: A Machine Learning-Based Approach
Nutrient-Wide Association Study for Dementia Risks: A Prospective Cohort Study in Middle-Aged and Older Adults
Modifiable factors associated with cognitive health trajectories among Indigenous, Hispanic, Black, and White older adults: an exploratory longitudinal panel analysis of the health and retirement study with a focus on Indigenous peoples
Investigation of risk of Alzheimer,Äôs disease diagnosis and survival based on variation to life experiences used to operationalize cognitive reserve
Residential environment and risk of chronic diseases: A prospective study
Integrating a motor domain enhances disease severity scales in an FTD-ALS spectrum cohort
Editor,Äôs view: delving deeper into physical activity and frailty research
Frailty incidence by diabetes treatment regimens in older adults with diabetes mellitus in the ASPirin in Reducing Events in the Elderly Study
Variation in depression's impact on dementia risk by age in adults aged ‚â•75 years
Chinese healthy diet and cardiometabolic multimorbidity risks: a prospective cohort study from the China Health and Nutrition Survey
Evaluating the impact of caloric restriction, body mass index and exercise on primary open-angle glaucoma: A review
Risk Factors for Falls in Community-Dwelling Older Adults: An Umbrella Review
The potential for dementia prevention in Brazil: a population attributable fraction calculation for 14 modifiable risk factors
Estimating the impact of risk factor reduction on dementia prevalence in New Zealand
Screening mild cognitive impairment using aspects of personal, social, and functional lifestyle: Machine Learning Approaches
Translating lifestyle interventions for optimal brain health in Africa
Epidemiology of falls in community-dwelling older adults in Europe: a systematic review and meta-analysis
The Relationship Between Diet and the Neuropathological Hallmarks of Alzheimer,Äôs Disease in Cognitively Normal Adults: A Systematic Narrative Review
How the combined approach of subjective and functional health improves the early identification of the older adults at risk of adverse events?
Biological brain age and resilience in cognitively unimpaired 70-year-old individuals
Dietary patterns and accelerated multimorbidity in older adults
Association between balance impairment and incidence of motoric cognitive risk syndrome in the China Health and Retirement Longitudinal Study
Brain and Body Fitness Group for Those With Dementia and Their Caregivers Through Community Partnership: A Program Evaluation
Falls among older adults: An exploration of trends, clinical outcomes, predisposing risk factors, and intervention strategies
Sleep disorders increase the risk of dementia, Alzheimer,Äôs disease, and cognitive decline: a meta-analysis
Study protocol using informatics to identify and recruit a cohort of older adults in Florida to develop teleneuropsychological norms
Can non-pharmacological interventions change levels of neurofilament light in older adults at risk of dementia? A secondary analysis of the SCD-Well randomized clinical trial
Uremic Toxins, CKD, and Cognitive Dysfunction
Dementia after stroke-risk prediction and management
Association of peripheral neuropathy with dementia in type 2 diabetes: a 5-year follow-up study using the national health insurance service-national health information database
Impact of social isolation on change in brain volume in community-dwelling older Japanese people: The NEIGE Study
Relationship of urate-lowering drugs with cognition and dementia: A Mendelian randomization and observational study
An inflammatory fingerprint in mild cognitively impaired patients is reversed by physical and cognitive training
Lessons from a Pilot Study of a Culturally Tailored Financial Well-Being Intervention Among Latino Family Caregivers
Potential Mediating Role of Polygenic Hazard Score in the Association Between Neurofilament Light Chain and Default Mode Network Connectivity Across the Alzheimer's Disease Continuum
JCS/JHRS 2024 Guideline Focused Update on Management of Cardiac Arrhythmias

Benefits of Multimodal Exercise Intervention for BDNF and Cytokines Levels, Cognitive Function, and Motor Functionality in Alzheimer‚Äôs Disease: A Preliminary Study
Demo Abstract: An LLM-Powered Multimodal Mobile Sensing System for Personalized and Interactive Health Behavior Analysis
Association between varicose veins and occurrence of dementia: A nationwide population-based cohort study
A Frailty-Based Plasma Proteomic Signature Capturing Overall Health and Well-Being in Older Adults
Atmospheric Pollutants, Cardiometabolic Disorders, and Dementia in Trajectory Analysis: A 13-Year Longitudinal Cohort Study
Influence of inspiratory muscle strength on 6-minute walk distance in patients with acute heart failure
Association of Predicted Visceral Fat Percentage With Dementia Risk in Older Adults: The Role of Genetic Risk and Lifestyle
Organic food consumption is positively associated with cognitive function among middle-aged and older adults: cross-sectional and longitudinal analyses
A systematic exposure-wide framework leveraging machine learning to identify multidomain exposure factors and their joint influence on cognitive function: Evidence from a neurological cohort
Multimorbidity, Muscle Strength, and Falls among Older Mexican Americans
Family history and genetic risk score combined to guide cancer risk stratification: A prospective cohort study
Genetic and Lifestyle Risks for Coronary Artery Disease and Long-Term Risk of Incident Dementia Subtypes
Salt added at the table, APOE genotype and incident dementia
Multidomain interventions for healthy aging: From neuroplasticity to population health
A Proteomics-Based Approach for Prediction of Different Cardiovascular Diseases and Dementia
Association of metabolic dysfunction-associated fatty liver disease with white matter hyperintensity and cognitive decline: A longitudinal cohort study
Alcohol consumption, drinking patterns and cause-specific mortality in an Australian cohort of 181,607 participants aged 45 years and over
Adherence to lifestyle intervention activities in the SINgapore GERiatric to reduce cognitive decline and physical frailty (SINGER) study: A one-year preliminary analysis of process evaluation
Heart Rate Variability, Cognitive Performance and Cognitive Impairment in Older Adults: Cross-Sectional Results From the Einstein Aging Study
Effect of Preinfection Health Status on COVID-19 Severity and Cognitive Function
Ethnic Differences in Dementia Diagnosis and Treatment in Israel
Association of Cognitive Frailty With Subsequent All-Cause Mortality Among Middle-Aged and Older Adults in 17 Countries
Navigating the brain: How cerebral blood flow shifts with task complexity
Long-Term cardiovascular outcomes after exercise-based cardiac rehabilitation among coronavirus disease 2019 survivors: A nationwide cohort study
Personalised Prevention of Falls in Persons with Dementia‚ÄîA Registry-Based Study
Discovering subtypes with imaging signatures in the Motoric Cognitive Risk Syndrome Consortium using weakly supervised clustering
The Impact of Physical Activity on Metabolic Health and Cognitive Function in Postmenopausal Women: A Cross-Sectional Study
Effects of a 6-month dual-task, power-based exercise program on cognitive function, neurological and inflammatory markers in older adults: secondary analysis of a cluster randomised controlled trial
Air pollution and cognitive performance in late life: the role of lipid profile ‚Äì results from the Multi-Ethnic Study of Atherosclerosis (MESA)
Higher vitamin B12 from mid- to late life is related to slower rates of cognitive decline
Association between depression and multimorbidity in Chinese middle-aged and older adults: a prospective cohort study
Sleep Fragmentation, 24-hr Rest‚ÄìActivity Patterns, and Cognitive Function in Premanifest Huntington‚Äôs Disease: An Actigraphy Study
Evaluation of Minds in Motion, a fitness program for people living with dementia and their caregivers
Participation in physical, cognitive, and social activities and the association with mobility disability transitions in older adults
Resistance training protects the hippocampus and precuneus against atrophy and benefits white matter integrity in older adults with mild cognitive impairment
The prevalence of chronic traumatic encephalopathy in a historical epilepsy post-mortem collection
Olfaction and Plasma Biomarkers of Alzheimer Disease and Neurodegeneration in the Atherosclerosis Risk in Communities Study
Scoping review exploring the impact of hip fracture in older adults with cognitive impairment or dementia
Long-term exposure to low-level nitrogen dioxide and risks of neurodegenerative diseases among middle-aged and older adults in the UK
The influencing factors of multiple disease burdens among middle-aged and elderly people in China
South Korean study to prevent cognitive impairment and protect brain health through multidomain interventions via face-to-face and video communication platforms in mild cognitive impairment (SUPERBRAIN-MEET): A randomized controlled trial
Prevalence of Cognitive Impairment/Dementia and Associated Risks Factors Among Community-Dwelling Elderly People Aged 65 or Above in the Community Centers in Hong Kong: A Cross-Sectional Study
A proteomic signature of healthspan
Features of sarcopenic obesity in rheumatoid arthritis patients
Effectiveness of group-delivered cognitive behavioural therapy for insomnia in primary care: a pragmatic, multicentre randomised controlled trial
Associations of Pain Characteristics With Physical Capacity Trajectories Among Older Adults in the United States
The joint effect of weight-adjusted waist index and physical activity on all-cause mortality in Chinese elderly patients with multimorbidity: A study based on the CLHLS from 2011 to 2018
ICTUSCOG: Poststroke cognitive impairment following nondisabling stroke: Study protocol of a spanish multicentre prospective cohort study
Exploring predementia: Understanding the characteristics of subjective cognitive decline plus from India
Incidence of liver complications with hemochromatosis-associated HFE p.C282Y homozygosity: The role of central adiposity

Association between nutrient patterns and odds of depressive symptomatology: a population-based cohort of older adults followed during 15-y
An online multidomain lifestyle intervention to prevent cognitive decline in at-risk older adults: a randomized controlled trial
Dietary patterns and transition from normal cognition to mild cognitive impairment and its reversion: A longitudinal study of older adults
Associations of social engagement and loneliness with the progression and reversal of frailty: longitudinal investigations of 2 prospective cohorts from the UK and the USA
Cardiometabolic diseases and dynamic transitions of neuropsychiatric disorders: A longitudinal trajectory analysis
Stratifying dementia risk factors: A prediction model and hypothesis-driven analysis
Is there a relationship between anesthesia/surgery and dementia?
Diagnostic Utility of Vestibular Markers in Identifying Mild Cognitive Impairment and Early Alzheimer,Äôs Disease in Older Adults
Exploring the landscape of social epidemiologic research: a scoping review of AJE publications
Socioeconomic area deprivation and its relationship with dementia, Parkinson's Disease and all-cause mortality among UK older adults: a multistate modeling approach
Joint effect of polysocial risk score, lifestyle and genetic susceptibility with the risk of dementia: A prospective cohort study
Blue spaces and incident dementia: Differences by geospatial and historical contexts
Sex Hormones and Risk of Incident Dementia in Men and Postmenopausal Women
Relationship of Lifespan Physical Activity Engagement With Quality of Life in Community-Dwelling Adults: A Retrospective Study
Early-life infectious disease exposure, the ,Äúhygiene hypothesis,,Äù and lifespan: Evidence from hookworm disease
The obesity paradox in cognitive decline: Impact of BMI dynamics and APOE genotypes across various cognitive status
Abdominal myosteatosis is associated with lower processing speed in a multiethnic cohort of older adults
Association between vertebral fractures and brain volume: insights from a community cohort study
Weight Loss Intentionality and Frailty are Associated with Pre,ÄìKidney Transplant Outcomes
Effective Solutions for Caregivers of Older Adults: A Review of Systematic Reviews
Cognitive reserve, genetic risk, and lifestyle in the incidence and progression of type 2 diabetes: A large community-based longitudinal study
Particulate matter, socioeconomic status, and cognitive function among older adults in China
Alzheimer, Menopause, and Hormone Therapy: Where are We Now? A Literature Review
Association of body composition with neuroimaging biomarkers and cognitive function; a population-based study of 70-year-olds
Metabolome informs about the chemical exposome and links to brain health
Association of healthy lifestyle with excess risk of dementia in individuals with hypertension
Association of trajectories of physical frailty status and incident disability in community-dwelling older adults: A longitudinal study
Can Hearing Aids Improve Physical Activity in Adults with Hearing Loss? A Feasibility Study
Early cognitive ability and dementia risk: Response to Chen and colleagues
Nutritional interventions for preventing cognitive decline in patients with mild cognitive impairment and Alzheimer's disease: A comprehensive network meta-analysis and Mendelian Randomization study
Subclinical left ventricular dysfunction in rheumatoid arthritis: findings from the prospective Porto-RA cohort
Pace of Aging analysis of healthspan and lifespan in older adults in the US and UK
Implementation and Outcomes of a Perioperative Geriatrics Strategy, PRIME, for Older Adults Undergoing Gastrointestinal Cancer Surgery
The independent and combined associations of nocturnal sleep duration, sleep midpoint, and sleep onset latency with global cognitive function in older Chinese adults
Cross-sectional associations of plasma vitamin D with plasma phosphorylated tau-181 and plasma neurofilament light chain in older adults with memory complaints
Mitochondrial blood-based biomarker is related to cardiorespiratory fitness and aging in a sex-dependent manner
Proteomic signatures of healthy dietary patterns are associated with lower risks of major chronic diseases and mortality
Risk of Dementia and Its Associated Factors Among the Patients With Coronary Artery Disease Attending a Tertiary Cardiac Hospital of Dhaka City: A Cross-Sectional Study
Multimorbidity and Peak Oxygen Uptake in Patients Undergoing Cardiac Rehabilitation
Detecting the fractal physical activity pattern in aged adults with cerebral small vessel disease
Auricular acupressure combined with auricular acupoint massage enhances cognitive function in night shift nurses: a P300 wave analysis
Impact of intermediate home-based care on functional health of older adults with stroke in low-income and middle-income countries: A systematic review
Circulating lipocalin-2 across the adult lifespan
Female Brain and Endocrinological Research,ÄìVeteran (FemBER-Vet) study: A study protocol for identifying endocrinological, lifestyle and psychosocial determinants of brain health outcomes in female veterans for future intervention success
Impact of Changes in Patient Reported Outcomes on Healthcare Use After a Self-Management Supportive Intervention for Individuals with Low Back Pain
Incidence trends and age-period-cohort analysis of Alzheimer,Äôs disease and other dementias in the world and China from 1990 to 2021: analyses based on the Global Burden of Disease Study 2021
How feasible is it to introduce routine cognitive screening in a prison setting?: a six-stage scoping review
Protecting the Heart in Motion: The Role of Physical Activity and Cardiorespiratory Fitness in Preventing Sudden Cardiac Death
Inequalities in Mild Cognitive Impairment Risk Among Chinese Middle-Aged and Older Adults: Insights from an Integrated Learning Model
Unmasking frailty in coronary artery disease: impact on outcomes after lower limb amputation

Health-related behaviours and depression incidence amongst UK adults aged 50+: Evidence from the English Longitudinal Study of Ageing
Leukocyte telomere attrition in cognitive decline: associations with APOE genotype and cardiovascular risk factors
Evaluating the safety and feasibility of remote ischemic conditioning for slowing cognitive decline in mild Alzheimer‚Äôs dementia
A Study on Feasibility and Acceptability of an AI-Powered VR/AR Cognitive Rehabilitation Platform for Patients with Alzheimer‚Äôs Disease and Dementia
Spirituality-based palliative care education on quality of life, death anxiety, and resilience of heart failure patients: Randomized controlled clinical trial
‚ÄúRest of the folks are tired and weary,‚Äù: The impact of historical lynchings on biological and cognitive health for older adults racialized as Black
Association of memory function with COVID-19 outcomes in adults aged 50‚ÄØyears and older: analysis of three prospective cohorts
The Machine Learning Methods in Rating Cognitive State of Elderly People
Effectiveness of an exercise and nutrition intervention for older adults with mild cognitive impairment: an open-label double-arm clinical trial
Impact of Glucose Profile, Fasting Insulin, and Renal Function on Sarcopenia in Elderly at Single Centered Nursing Home: A Cross-Sectional Structural Equation Model Analysis
Cardiovascular Health, 2010 to 2020: A Systematic Review of a Decade of Research on Life‚Äôs Simple 7
Predictors of 15-year transitions across living and care settings in a population of Swedish older adults
Feasibility of expiratory muscle strength training to address oropharyngeal dysphagia in a patient living with mixed dementia: A case report
Cognitive Function Profiles and Associated Factors in Older Adults Living with Type 2 Diabetes: A Latent Profile Analysis
Global Burden of Alzheimer‚Äôs Disease Attributable to High Fasting Plasma Glucose: Epidemiological Trends and Machine Learning Insights
Evaluating Barriers to Achieving the Minimal Important Change in Older Patients With Hip Fractures After Post-Acute Care
Cognitive profile and frailty in patients with idiopathic normal pressure
Non-pharmacological Approaches: Brain Stimulations, Vagus Nerve Stimulation, Magnetic and Electrical Transcranial Stimulations, Etc
Intrinsic capacity and ageing well for Aboriginal people in remote Western Australia: a longitudinal cohort study
8th International Workshop on ArtifiAI for Aging Rehabilitation and Intelligent Assisted Living, ARIAL 2025, Held in Conjunction with the International Joint Conference on Artificial Intelligence, IJCAI 2025
A brief literacy-independent screening tool to detect cognitive impairment
Exploring Factors Related to Social Isolation Among Older Adults in the Predementia Stage Using Ecological Momentary Assessments and Actigraphy: Machine Learning Approach
Olfaction and diabetes among older adults
Enlarged cavum septum pellucidum as a neuroimaging signature of head impact exposure
Association of vitamin D with risk of dementia: a dose-response meta-analysis of observational studies
Gait characteristics and factors associated with fall risk in patients with dementia with Lewy bodies
Assessing the effects of antidepressant use on stroke recurrence and related outcomes in ischemic stroke patients: a propensity score matched analysis
From Ageing Biology to Alzheimer‚Äôs Therapy: Integrating Geroscience, Lifestyle and Pharmacological Strategies
Coffee Consumption Correlates With Better Cognitive Performance in Patients With a High Incidence for Stroke
Cognitive and Alzheimer's disease biomarker effects of oral nicotinamide riboside (NR) supplementation in older adults with subjective cognitive decline and mild cognitive impairment
Does Sleep Moderate the Effects of Exercise Training or Complex Mental and Social Activities on Cognitive Function in Adults With Chronic Stroke? Secondary Analysis of a Randomized Trial
Yoga/Meditation and Physical Exercises and Associated Cognitive Function Among Older Adults: Findings from Baseline Wave of the Longitudinal Aging Study in India
Multi-pathway blood biomarkers to target and monitor multidimensional prevention of cognitive and functional decline (nested in the IN-TeMPO study framed within the world-wide FINGERS network)
Effects of Prolonged Antibiotic Therapy in Lung Abscesses‚ÄîAnalysis of Case Series
Development and validation of deep learning- and ensemble learning-based biological ages in the NHANES study
Early detection and management of hearing loss to reduce dementia risk in older adults with mild cognitive impairment: findings from the treating auditory impairment and cognition trial (TACT)
How older adults negotiate constraints to leisure-time physical activities following a heart attack
Association of reported sleep disturbances with objectively assessed mild cognitive impairment among adults in the United States
Geroscience-guided approaches for Alzheimer‚Äôs disease: targeting aging mechanisms for therapeutic intervention
Hekun decoction versus Femoston for women with amnestic mild cognitive impairment in early menopause: a randomized, three-arm, double-blind clinical trial
Editorial: Exercising body & brain: the effects of physical exercise on brain health
Editorial: An update on neurological disorders post COVID-19 infection vol 2: cardiovascular effects, neuro-cardiac and neuro-respiratory autonomic dysfunctions
Association of cognitive and structural correlates of brain aging and incident epilepsy. The Framingham Heart Study
Vitamin D deficiency may accelerate cognitive decline in female apolipoprotein E Œµ4 non-carriers
Association of hemorrhoidal disease with dementia risk: a nationwide cohort study
ADL+: A Digital Toolkit for Multidomain Cognitive, Physical, and Nutritional Interventions to Prevent Cognitive Decline in Community-Dwelling Older Adults
Late-life social activity and subsequent risk of dementia and mild cognitive impairment
The winter is coming: seasonal variations in BDNF levels among older adults in a high-latitude region ‚Äî a preliminary study
Subjective Memory Complaints and the Effect of a Multidomain Lifestyle Intervention on Cognition: The FINGER Trial

Perspectives on the Dementia Public Health Exposome
Life-space mobility and cognition in community-dwelling late-life women: A cross-sectional analysis
Water quality and neurodegenerative disease risk in the middle-aged and elderly population
Effectiveness of VR-based cognitive training and games on cognitive rehabilitation in patients with MCI: a systematic review and meta-analysis
Associations Between Traumatic Brain Injury Characteristics and Memory Outcomes: Insights from the Health and Retirement Study
Tea consumption may improve psychological resilience among older adults with chronic diseases: a prospective cohort study
Moderate alcohol consumption does not protect cognitive function when controlling for income and cultural factors
Mechanistic exploration of obesity-related indicators and motor cognitive risk syndrome: a mediated effect based on C-reactive protein triglyceride glucose index
Updated LIfestyle for BRAin health (LIBRA2) Score, Genetics, and Risk of Alzheimer Disease, Vascular Dementia, and Stroke in Older Adults
Race, diabetes, and cognitive function: a cross-sectional analysis of intersecting disparities in the NHANES cohort
Impact of Exercise on Cognitive Function and Motor Impairment in Patients with Type 2 Diabetes Mellitus: A Systematic Review and Meta-analysis
Mood index, sleep duration and risk of all-cause and cause-specific dementia: a prospective analysis of 429,761 UK Biobank participants
Examining the independent and moderating effects of arterial stiffness and cerebral blood flow on total hippocampal and hippocampal subfield volumes
Hyperuricemia reduces the risk of MCI but not dementia: a cross-sectional study in Liuyang
Beyond the sum of their parts: The combined association of dementia and chronic pain with self-care limitations in older Australians
Regional network covariance patterns of white matter integrity related to cardiorespiratory fitness in healthy aging
The relationship between cognitive function and muscle mass in older adults: a longitudinal study based on CLHLS
The mediating role of accelerated biological aging in the association between household air pollution from solid cooking fuels and neuropsychiatric disorders
The Rapid Online Cognitive Assessment for the Detection of Neurocognitive Disorder: Open-Label Study
Association between combined healthy lifestyle and cognitive function: Evidence based on Chinese Longitudinal Healthy Longevity Survey
Twenty key insights into neighborhood walking experiences of people living with dementia in Metro Vancouver: a quantitative analysis of qualitative data
Herbal medicine and acupuncture for mild cognitive impairment: a retrospective study of 2,242 for older adults in Republic of Korea
Translation, reliability, and validation of the Dutch Safe Use of Mobility Aid Checklist (SUMAC-NL) for walker use in people living with dementia
Healthy Cardiovascular Status Attenuates the Detrimental Association Between Osteosarcopenic Adiposity and Alzheimer‚Äôs Disease-Related Dementia: A UK Biobank Cohort Study
Forecasting Subjective Cognitive Decline: AI Approach Using Dynamic Bayesian Networks
THE ROLE OF PHYSICAL ACTIVITY IN MANAGING DEPRESSION IN ELDERLY POPULATIONS
Factors Associated with Long-Term Hospitalization in Older Patients with Heart Failure in Japan
Effects of Yoga-Like Exercises on Mild and Moderate Alzheimer Disease: A Randomized Controlled Trial Protocol
Possible influence of sex on the relationship between dual-task gait costs and cognitive decline in older adults
Long-Term Effect of Intensive vs Standard Blood Pressure Control on Mild Cognitive Impairment and Probable Dementia in SPRINT
Association of education level and depression with cognitive decline: findings from the examining cognitive health outcomes in heart failure study
The adaptation of meaning centered psychotherapy to develop RELOAD-C: a web-based tool to reduce loneliness in caregivers of persons with Alzheimer‚Äôs disease and related dementias
Gender differences in the association between smoking and the risk of suicide in depressed patients: a longitudinal national cohort study
The increased risk of exposure to fine particulate matter for depression incidence is mediated by elevated TNF-R1: the Healthy Aging Longitudinal Study
The association between chronic rhinosinusitis and the risk of dementia: a longitudinal study
Comparing machine learning classifier models in discriminating cognitively unimpaired older adults from three clinical cohorts in the Alzheimer‚Äôs disease spectrum: demonstration analyses in the COMPASS-ND study
Self-Monitoring and Its Effects on Physical Activity, Health Literacy, and Health-Related Quality of Life in Older Adults Receiving Long-Term Care
Associations and interactions between APOE e4 genotype and lifestyle with brain structural phenotypes
Lifetime occupation assignment and hypertension analysis in a survivor cohort of older adults
Moderate tea consumption and dementia-related neuroimaging markers
Long-Term Intake of Red Meat in Relation to Dementia Risk and Cognitive Function in US Adults
The Kashima Scan Study 2: a protocol for a prospective observational cohort study of cerebral small vessel disease in neurologically healthy adults
Correlation Between Oral Health Behaviors and the Susceptibility to Cognitive Function Impairment in the Taiwanese Older Population
COGNITIVE DECLINE AND LIFESTYLE FACTORS
The cost of a Mediterranean Diet and walking intervention to reduce risk of dementia and improve quality of life in independent living populations in Australia: The MedWalk randomised controlled trial.
A Low Anterior Thigh Muscle Thickness on the Nonaffected Side, but Not on the Affected Side, Predicts Lower Activities of Daily Living in Patients With Hip Fractures in Acute Care Settings.
Innovations in Dementia Empowerment and Action: RCT for Underserved Communities.
Usability and feasibility of INFORMA platform for computerized cognitive stimulation in mild cognitive impairment and early dementia: an 8-week multicenter study.
Cycling into Cognitive Health: Lessons from the U.K. Biobank.
Characterizing Human Oxidative, Anabolic and Glycolytic Metabolism in Athletes with Extreme Physiologies.
Skeletal Muscle Mass and Dementia Risk: Causal Estimates From Mendelian Randomization.

Accelerated Cognitive Decline in Pain-Insomnia-Depression Syndrome: Longitudinal Evidence and Protective Effects of Healthy Lifestyles.
Pain assessment and management in patients with dementia in Taiwan: a best practice implementation project.
Watching sports and subsequent health and well-being in older adults: a longitudinal outcome-wide study.
Cognitive decline, depression, and protective factors among middle aged and older Indigenous women.
Physical Activity in Mid- and Late Life Linked to Markedly Lower Risk of Dementia.
The Impact Of Strength Training In An Elderly Cohort With Dementia: 2006...American College of Sports Medicine (ACSM) Annual Meeting, May 27-30, 2025, Atlanta, Georgia.
Residential segregation of Black and Latinx older adults and brain imaging outcomes.
Association between muscle strength and dementia in middle-aged and older adults: A nationwide longitudinal study.
Fractional amplitude of low-frequency fluctuations in right dorsal cingulum bundle associated with depression symptoms in AD patients: Effects of donepezil intervention.
Physical activity partially mediates the association between health literacy and mild cognitive impairment in older adults: Cross-sectional evidence from Switzerland.
Passive measures of physical activity and cadence as early indicators of cognitive impairment: Observational study.
Diet quality and nutritional status variability predictors for neurodegenerative diseases and cognitive decline among older adults.
The prevalence and 2-year incidence of cognitive impairment and its association with depression and polygenic risk score in the United States: The health and retirement study, 2018-2020.
Cross-sectional and longitudinal associations between physical activity and intrinsic capacity in healthy older adults from the MAPT study.
Neighborhood physical activity facilities predict risk of incident mixed and vascular dementia: The cardiovascular health cognition study.
Patterns, determinants, and outcomes of early use of antidementia drugs: A 6-year multicenter cohort study in Thailand.
Corrigendum: Exercise program to reduce the risk of cognitive decline and physical frailty in older adults: Study protocol for an open label double-arm clinical trial.
Online chair yoga and digital learning for rural underserved older adults at risk for Alzheimer,Äôs disease and related dementias.
Boston university Alzheimer's disease research center clinical core: Infrastructure to facilitate research on post,Äêtraumatic Alzheimer's disease and related dementias.
Searching for responders to multidomain dementia prevention in late life: A pooled analysis of individual participant data from the MAPT and preDIVA trials.
Association between hospital-treated infectious diseases and risk of neurodegenerative disease among patients with prediabetes and diabetes: A prospective cohort study in UK Biobank.
Mediation of the association between APOE Œµ4 genotype, cognition, and dementia by neuropathology imaging markers in the Rotterdam study.
Cerebellar subregional structural changes across the Alzheimer,Äôs disease continuum: a longitudinal analysis of cognitive and behavioural correlates.
Ten,Äêyear outcomes of anticholinergic use among older adults with intellectual disability: Findings from the intellectual disability supplement to the irish longitudinal study on ageing (ids,Äêtilda).
Selected social and lifestyle correlates of brain healthmarkers: The Cross-Cohort Collaboration Consortium.
Development and validation of a convenient dementia risk prediction tool for diabetic population: A large and longitudinal machine learning cohort study.
Association between a healthy lifestyle and dementia in older adults with obesity: A prospective study in the UK biobank.
Blood biomarkers to detect Alzheimer disease.
Multidomain therapy for Alzheimer,Äôs disease: A scoping review of cognitive decline trials.
Neuroimaging biomarkers in understanding the relationship between metabolic health and Alzheimer,Äôs disease.
Cognitive and physical activities are associated with cognitive resilience in a memory clinic cohort.
Time-based and event-based prospective memory in mild cognitive impairment and Alzheimer,Äôs disease patients: A systematic review and meta-analysis.
Comparison of face to face and online delivery of a dementia-specific experiential learning activity.
GLP-1RA and SGLT2i medications for type 2 diabetes and Alzheimer disease and related dementias.
Early cognitive ability and dementia risk: Patterns beyond education and mortality.
Biomarker evidence of neurodegeneration in mid-life former rugby players.
Frailty and risks of dementia among middle-aged and older adults: Evidence from 3 prospective cohort studies.
Investigating the use of [18-F] tau-PET imaging as a biomarker of tau pathology in retired athletes at risk of neurodegeneration.
Dementia arts mapping: Observational methods for documenting impacts of poetry and recreation in care settings.
Virtual reality-based training has potential to improve dementia knowledge, attitudes, and sense of competence of physical therapists and occupational therapists.
Neuropathological changes and amyloid-related imaging abnormalities in Alzheimer's disease treated with aducanumab versus untreated: A retrospective case,Äìcontrol study.
Apathy, depression, and dementia risk in older adults: A global collaborative study.
Risk factors of dementia in a cohort of individuals with mild cognitive impairment in the Italian interceptor project.
Associations of longitudinal changes in blood biomarkers of dementia with the proportion of genetically inferred African ancestry.
The relationship between leisure time physical activity patterns, Alzheimer,Äôs disease markers and cognition.
Regional brain volume changes in Hakim,Äôs disease versus Alzheimer,Äôs and mild cognitive impairment.
Cognitive reserve predicts baseline tau burden in the U.S. POINTER trial imaging cohort.
Walking time and genetic predisposition for Alzheimer,Äôs disease: Results from the HELIAD study.
White matter trajectories in Down syndrome and Alzheimer's disease: Insights from diffusion tensor,Äìbased morphometry.

Frailty trajectories preceding dementia in the US and UK.
Physical and cognitive activities and trajectories of AD neuroimaging biomarkers: Longitudinal analysis in the Mayo Clinic study of aging.
Increased sedentary behavior is associated with neurodegeneration and worse cognition in older adults over a 7,Äêyear period despite high levels of physical activity.
Associations between accelerated forgetting, amyloid deposition and brain atrophy in older adults.
From physical activity patterns to cognitive status: Development and validation of novel digital biomarkers for cognitive assessment in older adults.
The risk of delirium or dementia,Äêrelated hospitalization among individuals living with dementia after long,Äêterm care entry: A population,Äêbased risk prediction model.
Investigating syndemic effects of air pollution and physical inactivity on cognitive decline in older adults.
Longitudinal comparative analysis of antidepressants and anti-anxiety medications in Alzheimer's and Parkinson's diseases: insights from French and British health records.
Deferiprone in Alzheimer disease: A randomized clinical trial.
The association of diabetes with Alzheimer's disease biomarkers and vascular burden across European aging and memory clinic cohorts.
Feasibility of high intensity exercise training in people with dementia and Mild Cognitive Impairment: a systematic review
Physical activity-based interventions in the management of dementia or cognitive impairment in sub-Saharan Africa: a scoping review protocol
Precision exercise in older adults with early Alzheimer's disease: The study protocol of the FIT-AD Sequential, Multiple Assignment, Randomized Trial (SMART)
The effect of hearing ability on dual-task performance following multi-domain training in older adults with mild cognitive impairment: findings from the SYNERGIC trial
Optimizing exercise for brain health: cortical activation and connectivity across different exercise modalities
The effects of structured aerobic exercise and mind-body exercise on cognitive function in older adults with MCI: Systematic review and meta-analysis
A Comparison of Cardiopulmonary Exercise Testing and Shuttle Walk Test in Older Adults With Amnestic Mild Cognitive Impairment
Frailty, grip strength, blood-based biomarkers of neuropathology, and incident dementia: a 17-year longitudinal population study
Suppressed brain-derived neurotrophic factor response to 6 min walking exercise in mild cognitive impairment
The comprehensive impact of exercise interventions on cognitive function and quality of life in alzheimer's disease patients: a systematic review and meta-analysis
Effects of exercise on cognitive function in older patients with Alzheimer's disease: a meta-regression and meta-analysis
Enhancing physical fitness in older adults: a six-month medium intensity training intervention yields significant improvements
Walking to protect against cognitive decline: the role of APOE genotype and sex
Effects of aerobic exercise interventions on cognitive function, sleep quality, and quality of life in older adults with mild cognitive impairment: a systematic review and meta-analysis
Effectiveness of an exergame-based training program on physical and cognitive function in older adults with cognitive impairment: a randomized controlled trial in rural China
Effects of aerobic exercise and computer-based cognitive training on cognition, functional independence, quality of life, and salivary cortisol levels in older adults with mild cognitive impairment: a randomized trial
Physical Activity Patterns and Brain Health: A Systematic Review About Weekend Warrior vs Regular Exercise on Neuropsychiatric Disorders
"Walking Really Helps me to Center Myself": Exploring the Psychosocial Aspects of Walking Experience in the Neighbourhood Among People Living With Dementia
Movement and brain health in perimenopausal and early postmenopausal females: A scoping review
Neuroprotective synergy of vitamin D and exercise: a narrative review of preclinical and clinical evidence on aging-related neuroplasticity and cognitive health
Effects of virtual reality walking-in-place exercise and seated cycling on grip strength, lower limb strength, and five times sit-to-stand test in elderly individuals with dementia: A parallel randomized controlled trial
A study on the effectiveness of individual centered goal-oriented cognitive rehabilitation in vascular dementia
Walking towards psychosocial well-being? Unveiling psychosocial impacts of a group-based walking program with and without cognitive enrichment in older adults-a mixed-methods randomized controlled trial
Neurovascular de-coupling underlies dual-task cost across cognitive abilities
Effectiveness of the Combined Exercise and Cognitive Training System in Enhancing Frontal Lobe Function in Older Adults: A Randomized Control Study
Association between vitamin D deficiency, low high density lipoprote in cholesterol and higher risk of activities of daily living disability and dementia in Chinese adults aged over 65 years
Physical exercise mitigates motor and muscular deficits in the 3xTg-AD model of Alzheimer's disease
Differences in Cognitive Health and Brain Activity According to Mild Cognitive Impairment and Physical Activity Levels in Older Women
The Functional Connectome Mediates Associations Between Fitness and Cognition Across the Adult Lifespan
Independent and joint associations of volume and intensity of physical activity on cognitive impairment among middle-aged and elderly Chinese adults: A national longitudinal study
Effects of two different doses of HIFT on physical function, cognitive performance, and quality of life in older adults with mild cognitive impairment: A randomized controlled trial
Insights from the care home staff on the use of observational risk assessment of contractures: Longitudinal evaluation (ORACLE): A qualitative study
RETRACTED: Effects of Tai Chi on Cognitive Function in Older Adults With Type 2 Diabetes Mellitus: Randomized Controlled Trial Using Wearable Devices in a Mobile Health Model (Retracted article. See vol. 27, 2025)
Muscle-brain axis mechanisms linking community-based exercise to cognitive function in older adults: A five-arm randomized controlled trial
Exploring the awareness of changes in outdoor mobility and the influence of the neighbourhood environment among people living with dementia in British Columbia, Canada

Onset of Depressive Symptoms and Leisure-Time Physical Activity Habits and Willingness Among Community-Dwelling Older Adults: A Prospective Longitudinal Cohort Study
Supporting safe walking and managing missing incidents in dementia: a qualitative narrative synthesis of current evidence
Reactive turning during walking improves in healthy older adults with a novel task-switching step exercise
Exploring the cognitive aspects of walking in the neighborhoods among people living with dementia
Protocol for a Pilot Two-Arm Crossover Randomized Controlled Trial of the ACTIVE Intervention for Older Adults with and Without Mild Dementia and Their Care Partners
"I wasn't broken": Addressing stigma associated with dementia while Walking the Talk for Dementia
Perceived rehabilitation needs of older people with dementia
The Gallery Walk as a method for patient and public involvement in health research: a case study in dementia care
Cost-Effectiveness of a Mobile Health Program for Pre-elderly Adults
Exercise-Induced Oxygen Desaturation and Cognitive Performance in Patients with Parkinson's Disease: A Prospective Observational Study
Walking the Talk for Dementia: A Catalyst For Change in the Global Dementia Landscape
Investigating the dynamic association of multiple risk factors on cognitive impairment in older people: a retrospective cohort study in Baisha Town, Hepu County, China
The scope and impact of Walking the Talk for Dementia 2024
Physical activity and subjective cognitive decline among US older adults: A population-based analysis of the 2023 behavioral risk factor surveillance system
Factors influencing the relationship between influenza vaccination and the risk of developing dementia: A systematic review
Rediscover Neighborhood: A Synthesis of Lived Experiences Following Dementia Diagnosis Through Walking Interview
Neighborhood History-based Social Walking for Caregivers and People Living with Early-Stage Dementia
Enhancing dementia risk prediction with heart rate and machine learning in the Canadian Longitudinal Study on Aging
Sex and APOE Œµ2 Interactive Effects on the Longitudinal Change in Cognition in a Population-Based Cohort of Older Adults with Vascular Risk Factors
Physical inactivity and depressive symptoms in older adults: A cross-national analysis of cohort studies in high- and low-income countries
'It keeps your body strong, your muscles strong, your brain strong:' perceptions about the role of physical activity and nutrition in brain health among middle-aged Latinos
The potential for dementia prevention in Japan: a population attributable fraction calculation for 14 modifiable risk factors and estimates of the impact of risk factor reductions
Fazekas score predicts cognitive decline & frailty in older adults: insights from the SAGE-AF cohort study
Agricultural or gardening physical activity may slow neurovascular aging and prevent stroke and dementia: an experimental and cross-sectional study
Physical activity across the life course and neural biomarkers
Interactive Associations of Age, Apolipoprotein E Œµ4 Gene, Physical Activity, and Physical Functioning on Processing Speed
Longitudinal Estimation of Adequate Physical Activity Levels to Reduce the Risk of Dementia in Older Adults with MCI
Impact of physical activity, health literacy and cognitive health on quality of life in dyslipidemia: an observational study
Differentiating gait behaviors between early-stage dementia with Lewy bodies and Parkinson's disease
Classification of dementia risk in the elderly through gait analysis with machine learning algorithms
Dual-Task Walking Across Task Combinations in Older Adults With Mild Cognitive Impairment
Association Between Self-Reported Sleep Quality and Conversion From Mild Cognitive Impairment to Dementia: A Retrospective Cohort Study Using the English Longitudinal Study of Ageing
Persistent sleep symptoms in post-COVID syndrome: a Brazilian prospective clinical study
Response Facilitation in Dementia Care: Exploring Engagement Through Social Contexts: A Qualitative Study in Dutch Nursing Homes
Measuring the care risk in older adults with dementia: a scoping review
Investigating the trivariate reciprocal longitudinal relationships between purpose, loneliness, and cognitive function
Extraordinary abilities, creativity, and love, in the path of dementia: a perspective article on the Walking the Talk for Dementia 2025 initiative
The Mechanism of Exerkines on The Comorbidity of Sarcopenia and Cognitive Impairment
Cardiorespiratory fitness does not predict longitudinal changes in manual dexterity, cognition or corticospinal excitability in multiple sclerosis
Age-Dependent Functional Decline and Recovery Patterns After Dementia Diagnosis in the Oldest-Old
Can dual-task high-velocity exercise training improve cognitive function in older adults? Secondary analysis of an 18-month cluster randomized controlled trial
Sex-Specific Contributions to Single-and Dual-Task Walking in Adults at Risk of Alzheimer Disease and Related Dementias
The Brain Resilience Study protocol: Building a dataset of the biological and sociocultural factors affecting brain health in older adults
Integrating neighbourhood walkability perceptions of people living with dementia in urban design and planning in British Columbia, Canada ,òÜ
Dual-task walking for early detection of Alzheimer's disease: comparative analysis of tasks using whole-body gait variables
Mapping the evidence on virtual reality for dementia caregivers: a scoping review
Development and validation of a risk prediction model for mild cognitive impairment in older Chinese adults with chronic diseases
Public library visits and later-life health and well-being: evidence from a longitudinal study of older adults in Australia
Effects of cognitive rehabilitation and exercise on brain structure in progressive multiple sclerosis: results from the CogEx trial
Exploring How Nuanced Agency Enables Citizenship of People Living With Dementia in Their Home Gardens
Gait speed and domain-specific cognitive performance in a diverse cohort of the oldest-old: the LifeAfter90 Study

Variability in spatial and temporal gait dynamics during aging: a narrative review on the role of the neural system
Left ventricular remodeling and diastolic dysfunction predict cognitive decline in older adults
Process Evaluation of a Dementia Prevention Program for Aboriginal Australians (DAMPAA) Using a Theory of Change Framework
Differential profiles of motor dysfunction in amnestic versus non-amnestic mild cognitive impairment-The Vietnam Era Twin Study of Aging
Prediction of safety accident subtypes for persons with dementia using sensors and machine learning: an observational study
Mediating pathways of physical capabilities: linking perceived physical fatigability to motoric cognitive risk syndrome in community older adults
Beyond Memory: psychogeography, photo-elicitation and dementia
Polypharmacy and cardiovascular co-morbidity as key contributors to frailty in geriatric patients with AF - insights of the Dutch-GERAF cohort study
Cognitive reserve, frailty status, and risk of neurodegenerative diseases: a prospective cohort study
How to Safely and Effectively Apply High-Intensity Strength Training in Older Adults with Alzheimer's Disease: A Case Report
Livable City and Depressive Symptoms among Older Adults in China--Evidence from the China Health and Retirement Longitudinal Studies (CHARLS)
Exploring the Relative Contributions of Multiple Modifiable Physical Health Variables to Executive Function and Episodic Memory Performance in Older Adults
Childhood maltreatment confers long-term risk for cognitive impairment: A prospective investigation
Sensorized Motor and Cognitive Dual Task Framework for Dementia Diagnosis: Preliminary Insights From a Cross-Sectional Study
Lower CD8+T-cell senescence partially mediates the neuroprotection of higher aerobic fitness
Longitudinal bidirectional dynamic association between handgrip strength and cognitive function in Chinese older adults: a cross-lag modeling study
Dementia-specific adaptations to physical performance tests of balance, mobility, and lower limb strength and function: a reliability study in people with dementia
Going for walks measurably slows down preclinical Alzheimer's disease.
Real-world walking patterns are associated with regional brain atrophy and white matter lesions in middle-aged and older people: a Watch Walk-UK Biobank study
Way-Finding and Sensory Landscapes: Designing Navigable and Stimulating Outdoor Spaces for Older Adults with Mild Cognitive Impairment
Care Needs for Patients Screened Positive for Cognitive Impairment and Delirium: A Cross-Sectional Observational Study
Characteristics of Australian cognitive super-agers using different measurement approaches
Long-term adherence and changes in the Mediterranean and MIND diets in relation to dementia risk and cognitive function
The Influence of Work-Related Activities on Mental Well-Being in Individuals With Dementia or Mild Cognitive Impairment: A Multicentre Cross-Sectional Study in Japan
Trajectories of Social Activity Engagement and Physical and Cognitive Function During the Last Years of Life
Clinical, social, molecular, and genetic predictors of cognitive resilience in long-living adults without dementia
Ambient air pollution and incident dementia: exploration of relevant exposure windows
Association between physical activity and cognitive impairment in Chinese older adults: A cross-sectional study
Toward assessing functional decline in mild cognitive impairment using wearable sensors and explainable machine learning
From images to health insight: integrating MLLM, NLP, and objective Q-sorting of nursing-home built environment orientations
Sex differences in the associations among grip strength, walking speed, sarcopenic obesity, and mild cognitive impairment: Evidence from the Qingdao (Laoshan) Aging and Cognition Screening (QLACS) Project
Gait in Older People with Cognitive Impairments During Rhythmic Auditory Cueing: A Scoping Review
Influence of Behavioral and Psychological Symptoms of Dementia Clusters on Activities of Daily Living in a Subacute Rehabilitation Setting
Predicting safe street-crossing for older adults using a multifactorial model: The role of visual perceptual, cognitive and physical factors
Effect of Du-Moxibustion Combined With Spine-Pinching Therapy on Cognitive Frailty in Older Adults With Prefrailty: Protocol for a Randomized Controlled Trial
Characterizing motoric cognitive risk syndrome beyond traditional criteria
Plant-Based Diet Quality, Healthy Lifestyle, and Dementia Risk in Older Adults With Cardiometabolic Diseases
Can Turn Duration and Step Parameters During the Timed Up and Go Test With and Without a Dual-Task Discriminate Between Individuals With Different Cognitive Abilities? An Explorative Study
Lightening the Load: The Relationship Between Gait and Cognition for Persons Living with Dementia Engaged in a Non-Pharmacological Intervention
Activity participation reduces the risk of late-life depression: a sex-specific analysis of the AgeCoDe/AgeQualiDe study
Improving Quality of Life in Dementia through Tailored Interventions: A Systematic Review from Saudi Arabia
Examining the Impact of a Virtual Dementia Simulation on Occupational Therapy Students: A Mixed Methods Study of Empathy Cultivation
Mechanisms and Impact of Cognitive Reserve in Normal Aging and Alzheimer's Disease
Primitive reflexes as behavioral biomarkers of cognitive aging: associations with physical activity and resilience-a pilot study
A systematic review and meta-analysis of the relationship between midlife physical activity and later-life cognition: Is sex an overlooked factor?
Donepezil treatment, alone or combined with exercise, enhances skeletal muscle function in healthy and advanced aging disease mouse models
Association between ranolazine therapy and cognitive decline in elderly patients with ischemic heart disease
Evaluation of Frequency, Severity and Spectrum of Cognitive Impairment and Its Associated Risk Factors in Elderly Individuals with Diabetes in India: A Prospective Observational Cohort Study
Immersive, Open-World Virtual Reality for Dementia Care: NeuroVRX Pilot Study
Active aging activities and genetic determinants of cognitive health: findings from the Kuakini Honolulu-Asia Aging Study
Association of Physical Frailty With Serum Growth Differentiation Factor-15 and Cognitive Decline: A Cross-Sectional Study

Exploring Factors Associated With Cognitive Impairment in Rheumatoid Arthritis
The association between amyloid-beta deposition on dual-task gait performance is partially moderated by cognitive functions in healthy older adults
Chronic pain and subjective memory problems are dynamic risk factors for functional decline: An ACTIVE longitudinal study
Strategies to support safe wandering in care homes for older adults - what works, for whom, and in which circumstances?: A realist synthesis
Association of physical activity and life space with cognitive frailty in patients on hemodialysis: the REPnet-HD study
Smartphone-based gait analysis with rhythmic auditory cueing discriminates cognitive impairment stages in a resource-limited African setting
The longitudinal mediating effect of life-space mobility on the relationship between nutritional status and cognitive function in community-dwelling older stroke patients
Advocates, Academics, Survivors and Clinicians to END Intimate Partner Violence (ASCEND-IPV) initiative: a prospective observational case-control study protocol to identify plasma biomarkers of intimate partner violence (IPV)-caused brain injury (BI)
Comparing voice-only navigation and paper maps in middle-aged and older adults: A randomized crossover field study of wayfinding, mood, usability, and text-mined user experiences
The association between handgrip strength, physical, and cognitive function among hospitalized older adults in Tanzania
A multimodal sleep foundation model for disease prediction
Association of moderate to vigorous physical activity with cognitive performance and risk of cognitive impairment: A cross-sectional study and Mendelian randomization analysis
Digital Reminiscence for Predeath Grief Among Family Caregivers of Patients With Dementia
Impact of Lifestyle Activity Engagement on Incident Disability: A Stratified Analysis by Cardiovascular Disease Risk
Characterization of motor and non-motor features associated with bilateral nigral degeneration due to A53T alpha-synuclein in female rats
Technology-Enhanced Dual-Task Testing for Alzheimer's Disease and Related Dementias: A Review of Trends, Tools, and Emerging Directions
Challenges of Dementia Care among Older Adults Living Alone in Japan: Insights from a Memory Clinic Study
Cognitive impairment and associated factors among adult diabetes mellitus patients in Amhara National Regional State referral hospitals, Ethiopia: a multicenter institution-based cross-sectional study
Association of cognitive reserve with 9-year domain-specific cognitive trajectories and risk of cognitive impairment in Mexican older adults
Synthesising evidence on the association between the structure, function and quality components of social connection and quality of life for people with dementia: a scoping review protocol
Recent advances and ongoing challenges in diabetes prevention and control in China
Rest-Activity Rhythm Characteristics and Cognitive Function in Cognitively Healthy Older Adults
The importance of modifiable lifestyle factors for episodic memory: a gradient boosted tree analysis
Research Among People Living With Dementia or Mild Cognitive Impairment in Physiotherapy Journals: A Scoping Review
How well do social frailty indices predict incident dementia in older adults?
What Visually Directed Action Reveals About Perception of Ambulatory Space
Associations between 10-second standing balance tests and mild cognitive impairment in older adults in Colombia: A cross-sectional national survey
Trajectories of social participation and risk of cognitive impairment in Chinese older adults: A six-year longitudinal study
The association between sarcopenia, sedentary behavior, and the motor cognitive risk syndrome: A Mendelian randomization study
Gamma sensory stimulation in mild Alzheimer's dementia: An open-label extension study
Accelerometer-Based Gait Analysis as a Predictive Tool for Mild Cognitive Impairment in Older Adults
Emergency Physicians' Ethical Duty to Patients with Undiagnosed Idiopathic Normal Pressure Hydrocephalus
Multidimensional Factors Are Related to Caregiver Burden: Findings From a National Experience Sampling Survey
Cognitive impairment among older adults in India: understanding the role of substance use and lifestyle factors
Long-Term Effects of Mobile-Based Metamemory Cognitive Training in OlderAdults With Mild Cognitive Impairment: 15-Month Prospective Single-Arm Longitudinal Study
Longitudinal Associations of Social Isolation, Systemic Inflammation, and Cognitive Function Among Older Adults With Diabetes: Cross-Lagged Examination
Clinical clusters during acute illness predict long-term mortality in older patients
A Systematic Review and Meta-Analysis of the Effect of Virtual Reality on Cognitive Function and Instrumental Activities of Daily Living in Older Adults With Mild Cognitive Impairment
The role of sleep disorders in the motoric and cognitive trajectories of older physically frail sarcopenic and healthy active subjects: SOMNUS-DARE, a prospective cohort study
Referrals to allied health professionals for people with dementia: an analysis of general practitioner data from two Australian primary health networks
Sensor systems for the care of people with wandering behavior in hospital: A qualitative study on subjective experiences
Healthy lifestyle partly mediates the association between self-rated health and risk of overall and cause-specific mortality
Psychological and mental states mediated the association between physical activity and self-rated health of Chinese centenarians
Intra-individual variability in non-motor neurodegenerative disorders: A scoping review of current methodology and exploration of differential sensitivity along the spectrum of neurocognitive decline
Relationship between sedentary time and cognitive decline in hypertensive patients-mediating role of the weight-adjusted waist circumference index
Micronutrient balance and brain function: neuropsychological, metabolic, and clinical interactions
Research on Alzheimer Disease in Italy: A Narrative Review of Pharmacological and Non-Pharmacological Interventions
Cost-effectiveness of multimodal intervention for the prevention of dementia in Japan

The Results of a 12-Month Open-Label Follow-Up Study with MRI Monitoring of Patients with Parkinson's Disease After MRI-Guided FUS
The primary hospitals should take gait speed as a routine test for elderly patients
Half a century of research for mind,Äìbody interventions: A scientometric analysis.
Longitudinal association of inflammatory diets on cognition in older adults: Insights from the oral,Äìgut,Äìbrain axis.
Social determinants of health and mild cognitive impairment in a diverse community sample.
Development and Validation of Machine Learning Models for Predicting Early Cognitive Decline Using Home Sensor-Derived Behavioral Data: Sensors in-Home for Elder Wellbeing (SINEW) Cohort Study
Associations between major depressive disorders and Parkinson's Disease and impact of their comorbidity sequence.
Hearing loss and its relation to longitudinal changes in white matter microstructure in older adults: The Rotterdam Study.
Sociodemographic Characteristics and Preference Indicators Among Individuals Requesting Advance Directives
Cardiovascular disease as a mediator in the relationship between lifestyle risk factors and cognitive outcomes: A scoping review.
The mediating role of anxiety and depressive symptoms on the relationship between physical limitations and cognitive impairment among older adults in China: differences based on religious perspective
Delirium in the long-term care: a challenge for clinicians and researchers-the negative outcomes related to delirium: a scoping review
Association of an in vivo classifier for ARTerioloSclerosis (ARTS) with cortical thickness and cognition in older adults.
Impact of Hypertension on Physical and Cognitive Performance Under Single- and Dual-Task Conditions in Older Adults
Role of B vitamins inmodulating homocysteine and metabolic pathways linked to brain atrophy: Metabolomics insights from the VITACOG trial.
Patterns of Social Participation Among Survivors of Stroke in China and the Relationship With Personal and Environmental Factors: A Latent Class Analysis
Distinct white matter abnormalities and cognitive impairments in deficit schizophrenia: A cross-sectional diffusion tensor imaging study.
Plasma neurology-related proteins associated with cognition are modulated by lifestyle in adults
Longitudinal associations of life space mobility and domain-specific cognitive measures in ACTIVE.
Slowing cognitive decline in major depressive disorder and mild cognitive impairment: A randomized clinical trial.
Fear of falling impairs spatiotemporal gait parameters, mobility, and quality of life in Parkinson's disease: A cross-sectional study.
Regional brain structural alterations in reward and salience networks in asthma.
Does estradiol administration influence memory performance after menopause?
Hippocampal-subfield macro- and microstructural changes in cerebral small vessel disease with mild cognitive impairment.
Pragmatic questionnaire-based evaluation of auditory function in individuals with major neurocognitive disorders and hearing loss in diverse contexts.
A song for the mind: A literature review on singing and cognitive health in aging populations.
A rising drug burden index is related with malnutrition in community-dwelling older adults with dementia.
Feasibility and acceptability of a technology-mediated fall risk prevention intervention for older adults with mild cognitive impairment.
Age-related eye diseases and subsequent risk of mental disorders in older adults: A real-world multicenter study.
Pharmacological interventions and telomere length in patients with schizophrenia and bipolar disorder: A systematic review and meta-analysis.
Decomposing racial and ethnic disparities in risk and protective factors of dementia in the u.S.
Freezing of gait is associated with daily activity limitations among individuals with Parkinson,Äôs disease and mild cognitive impairment.
Chronic use of benzodiazepine in older adults and its relationship with dementia: A systematic review and meta-analysis.
REM sleep without atonia and neurocognitive function in isolated REM sleep behaviour disorder: Cross,Äêsectional and longitudinal study.
Improving Vietnamese Immigrants' Cognitive Health Literacy Through University-Community Partnerships
Systematic review of actigraphy measures associated with behavioral and psychological symptoms of dementia.
Relationship between social activities and cognitive impairment in Chinese older adults: The mediating effect of depressive symptoms.
Connecting the dots: Microstructural properties of white matter hyperintensities predict longitudinal cognitive changes in ageing.
Associations of childhood economic and psychosocial conditions with later-life cognitive function: a longitudinal analysis of the China health and retirement longitudinal study (2011-2020)
The association between gait speed and depressive disorders ,Äì A cross-sectional analysis of very old adults in the 21st century.
The Association of Dysphagia with Social Isolation, Mental Health, and Cognition in Older Adults
Sex differences in mortality and health care utilization after dementia diagnosis.
Characteristic activation pattern and network connectivity of brain in type 2 diabetes mellitus patients with mild cognitive impairment: A functional near-infrared spectroscopy study
Association of midlife air pollution and road proximity exposure with 29-year cognitive decline
An Instrumental Activities of Daily Living Decline Detection Analysis Pipeline Using Temporal Changes in In-Home Sensor Data
Motor Neuron Disease with Guillain-Barr√© Syndrome? Motor Band Sign with Anti-GQ1b Antibodies
From Dogs to Robots: Pet-Assisted Interventions for Depression in Older Adults-A Network Meta-Analysis of Randomized Controlled Trials
Multimorbidity patterns and cognitive frailty in adults aged over 50 years: China perspective
User experience with two computerized cognitive intervention programs for people with mild cognitive impairment
Cognitive dysfunction in aging dogs and cats: diagnosis and management perspectives
The mediating effect of activities of daily living (ADL) disability on the association between depressive symptoms and cognitive impairment in older adults: a National study

Frequency and factors related to very early return of older persons to the emergency department: A separate analysis of men and women (EDEN-26 study)
Insights on Recruitment, Implementation, and Movement Pattern Detection by Exploring the Feasibility of Sensor-Based Insole Technology in Long-Term Care: Mixed Methods Feasibility Study
Transdiagnostic neuroanatomical risk in schizophrenia: integrating regional vulnerability indices with anthropometric and fitness-based markers of cardiometabolic health
The impact of osteoarthritis and geriatric depression scale on mini-mental state examination trajectories over seven years
QENNA: A quantum-enhanced neural network for early Alzheimer's detection using magnetic resonance imaging
The association between social engagement and cognition in older adults: examining sex and ethnoracial group as moderators
Memantine modulates neuroinflammation and motor coordination in a Parkinson's disease model
Decomposition and comparative analysis of differences in loneliness between urban and rural older adults: evidence from a national survey
Disentangling the inverse relationship between cancer and Alzheimer's or Parkinson's disease: A systematic review on Mendelian randomization studies
Motor, not cognitive, performance relates to amyloid status in normal older adults
Association between depressive symptoms and cognitive impairment among older adults in China: a mediation analysis of proactive health
Heterogeneity in the association between retirement and cognitive function: a machine learning analysis across 19 countries
Chronotype, sleep quality, and functional health in active older adults: A cross-sectional study
Integrative gene co-expression network analysis reveals protein-coding and LncRNA genes associated with Alzheimer's disease pathology
Ternary hybrids accelerated photoelectrochemical biosensor for sensitive detection of Alzheimer's disease biomarker
Harnessing copper: Innovative approaches to combat neurodegenerative diseases and cancer (Review)
Plasma Alzheimer's biomarkers and physical functions in aging adults with and without motoric cognitive risk syndrome
Safety, tolerability and biomarker results of bepranemab in participants with progressive supranuclear palsy: a randomised, multicentre, double-blind, placebo-controlled, phase 1b trial
Neuroimaging-based data-driven subtypes of spatiotemporal atrophy due to Parkinson‚Äôs disease.
The effectiveness and economic evidence of organizational and management interventions to promote mental wellbeing and resilience in elderly care workers and informal caregivers - a systematic review
Brain Imaging Biomarkers and Cognitive Outcomes in a Multidomain Lifestyle Intervention
The Role of Oligodendrocytes in Alzheimer's Disease Pathogenesis and Therapy
Efficacy of branched chain amino acid administration on cognitive function: a systematic review
Artificial Intelligence for Detection of Parkinson's Disease From Speech Signals-A Comprehensive Review
Your brain doesn't look a day past 70! Cross-sectional associations with brain-predicted age in the cognitively-intact oldest-old
Molecular mechanisms underlying obesity-induced memory dysfunction: a comprehensive narrative review
Retinal nerve fibre layer thickness reflects characteristics of brain grey and white matter
Olfactory and Cognitive Performance Improvement After Oxygen-Ozone Major Autohemotherapy in Mild Cognitive Impairment: A Retrospective Cohort Study
Molecular Mechanisms Underlying Sleep Deprivation-induced Acceleration of Alzheimer's Disease Pathology
Cognitive frailty and its influencing factors in the elderly rural community residents of inner Mongolia, China
Examining the role of systemic inflammation as a mediator of the glycaemia-brain volume associations in women
Pittsburgh Brain Health Initiative (BHI): protocol and methods for an observational study of cognitive function in former professional football players and controls
Apathy in Cerebral Small Vessel Disease Stroke Is a Predictor of Quality of Life, Mood and Distress in Both Patients and Carers
Integrating Clinical Judgment and Objective Measures in Normal Pressure Hydrocephalus: Key Predictors of Shunt Referral
Associations between osteosarcopenia and cognitive function in community-dwelling older adults: evidence from a US cohort
Plasma brain-derived tau: analytical and clinical validation of the first commercial immunoassay

Supplementary Methods S10. Selection and Exclusion of Overlapping Cohort Studies

To ensure the statistical independence of our results and avoid the duplication of participant data, we implemented a systematic hierarchy for selecting a single representative study when multiple publications utilized the same cohort. Priority was given based on: (1) methodological quality score, (2) duration of follow-up, and (3) sample size, in that order.[1,13] Studies that met general eligibility criteria but were redundant due to cohort overlap were excluded as follows:

UK Biobank: Zhu et al.[14] was retained for the final analysis. Hou et al. [15] was excluded due to a lower methodological quality score, and Petermann-Rocha et al. [16] was excluded because Zhu et al.[14] provided a more extensive follow-up period.

Korea National Health Insurance Service (KNHIS): Jung et al. [5] was retained for the final analysis. Overlapping analyses by Park et al. [17] and Yoon et al. [18] were excluded due to lower methodological quality scores**.**

Framingham Heart Study: Li et al.[8] was retained for the final analysis. Giorgio et al.[19] was also excluded due to a lower quality rating. Hwang et al. [20] and Marino et al.[21] were excluded due to shorter follow-up durations.

Atherosclerosis Risk in Communities (ARIC) Study: Li et al.[8] was retained for the final analysis, while Palta et al.[22] was excluded due to a shorter follow-up period.

Japan Gerontological Evaluation Study (JAGES): Sato et al.[23] was retained for the final analysis. It demonstrated higher quality than Du et al.[24] and provided a longer follow-up duration than Kanamori et al.[25]

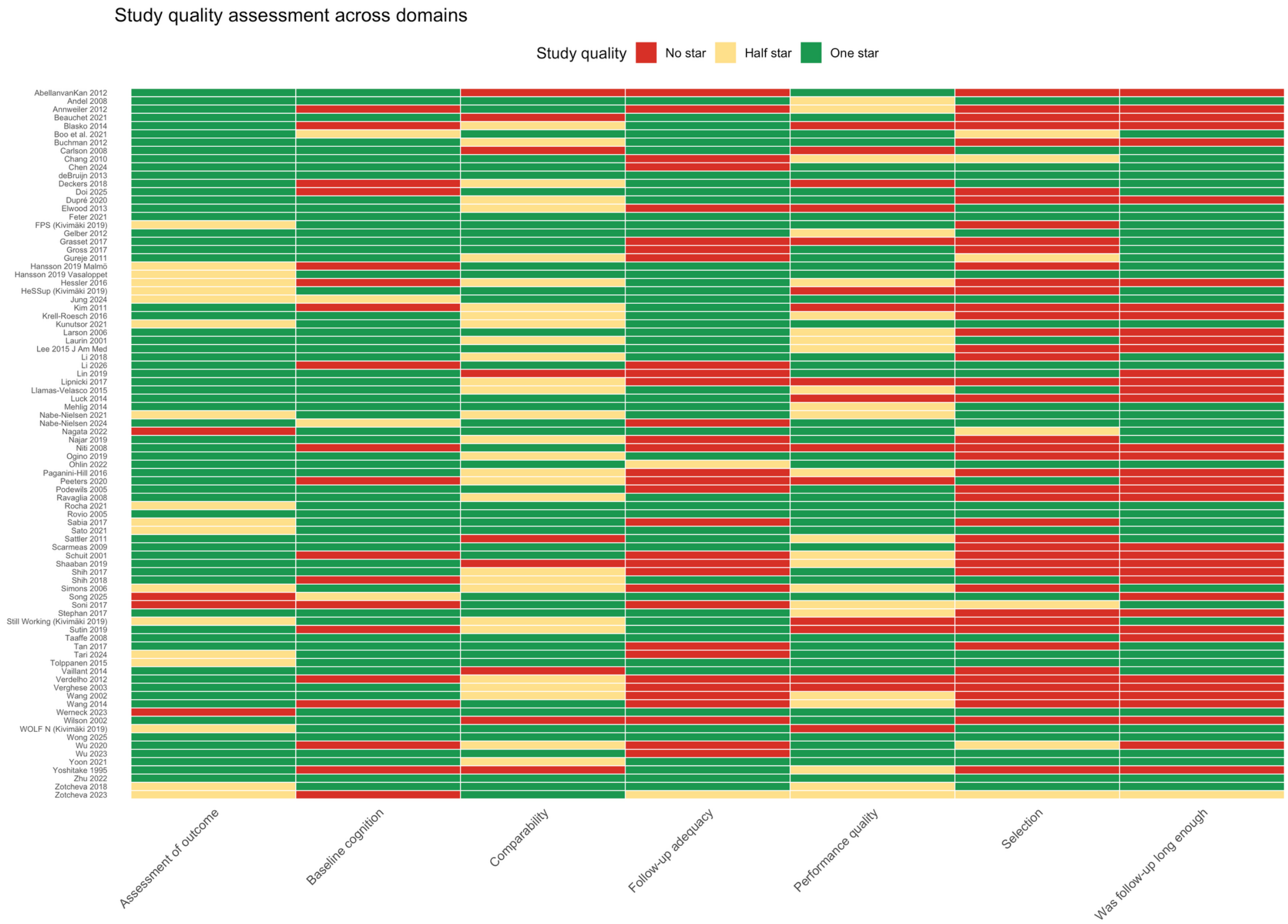

Supplementary Figure S1: Study quality assessment across methodological domains

This figure summarizes the methodological quality of the studies included in the meta-analysis using a modified Newcastle–Ottawa–type star-based framework. Each row represents an individual study and each column a quality domain (selection, performance quality, baseline cognition, comparability, assessment of outcome, adequacy of follow-up length, and follow-up completeness). Cells are colour-coded to indicate the level of quality assigned to each domain: no star (red), half a star (yellow), and one star (green).

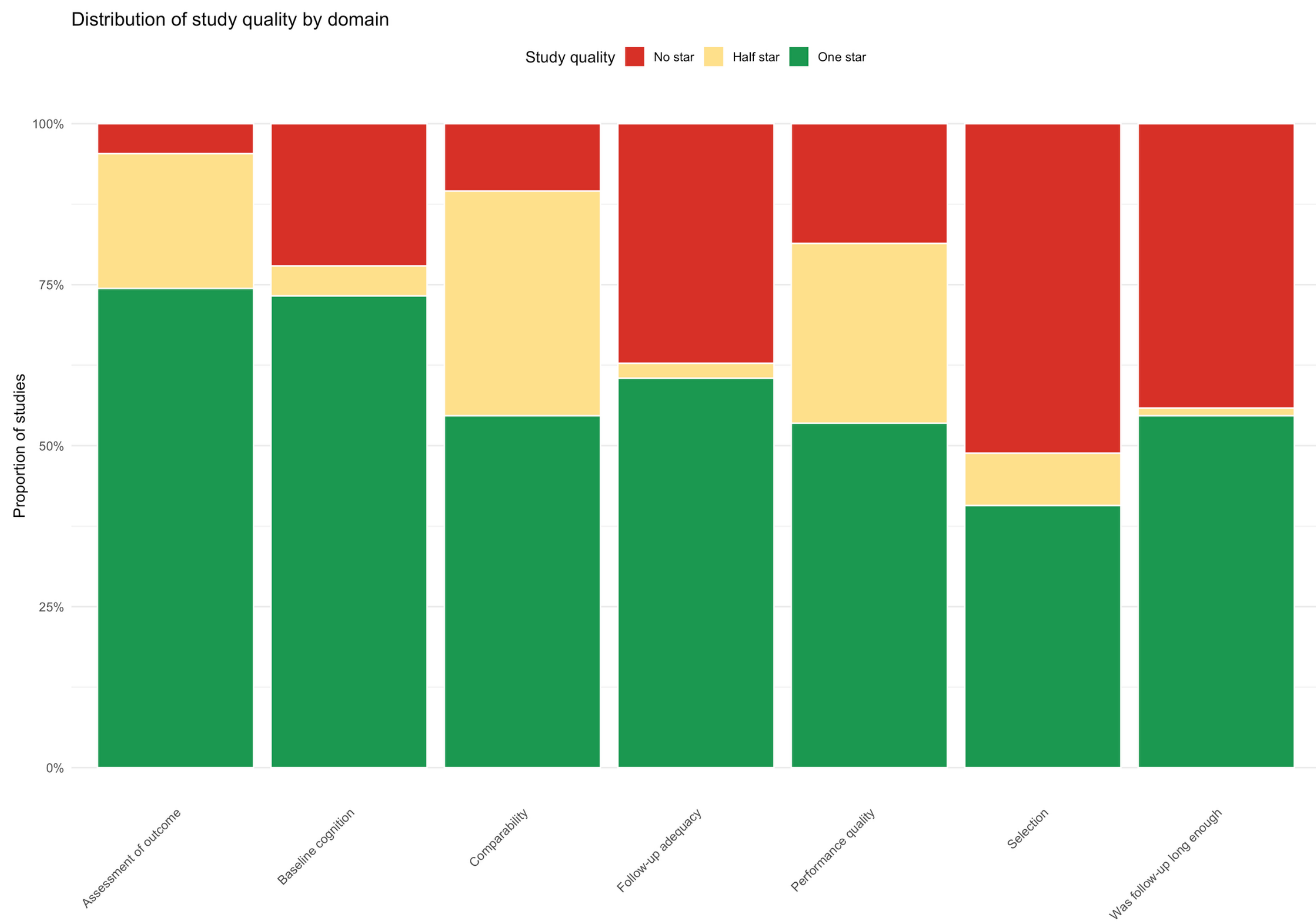

Supplementary Figure S2. Distribution of study quality ratings by methodological domain

This stacked bar chart shows the proportion of studies rated as no star, half a star, or one star within each methodological domain of the quality assessment. Bars represent the relative contribution of each rating category to the total number of included studies per domain.

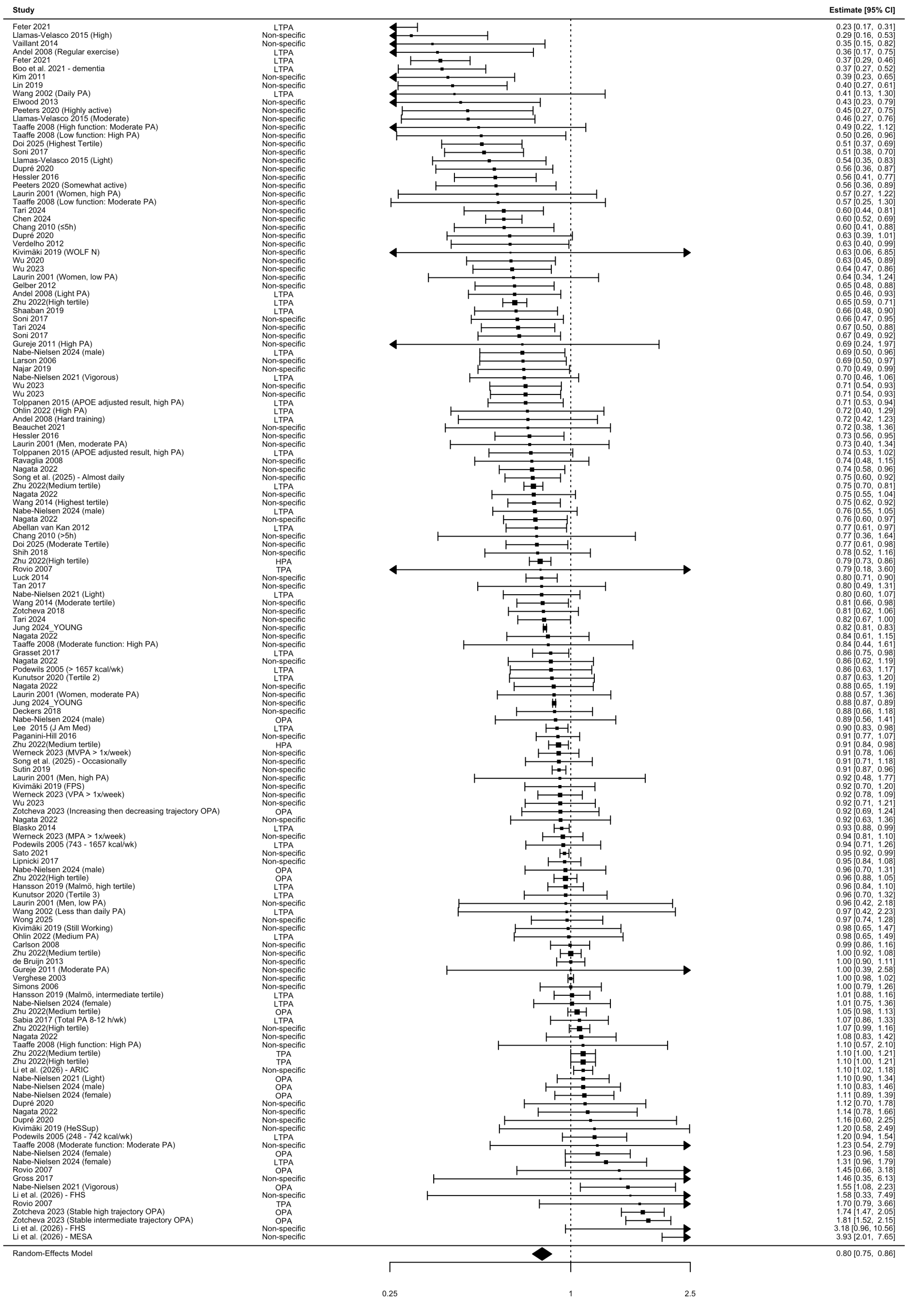

Supplementary Figure S3. Pooled association between physical activity and all-cause dementia risk.

LTPA: leisure-time physical activity, OPA: occupational physical activity, HPA: household physical activity, TPA: travel physical activity

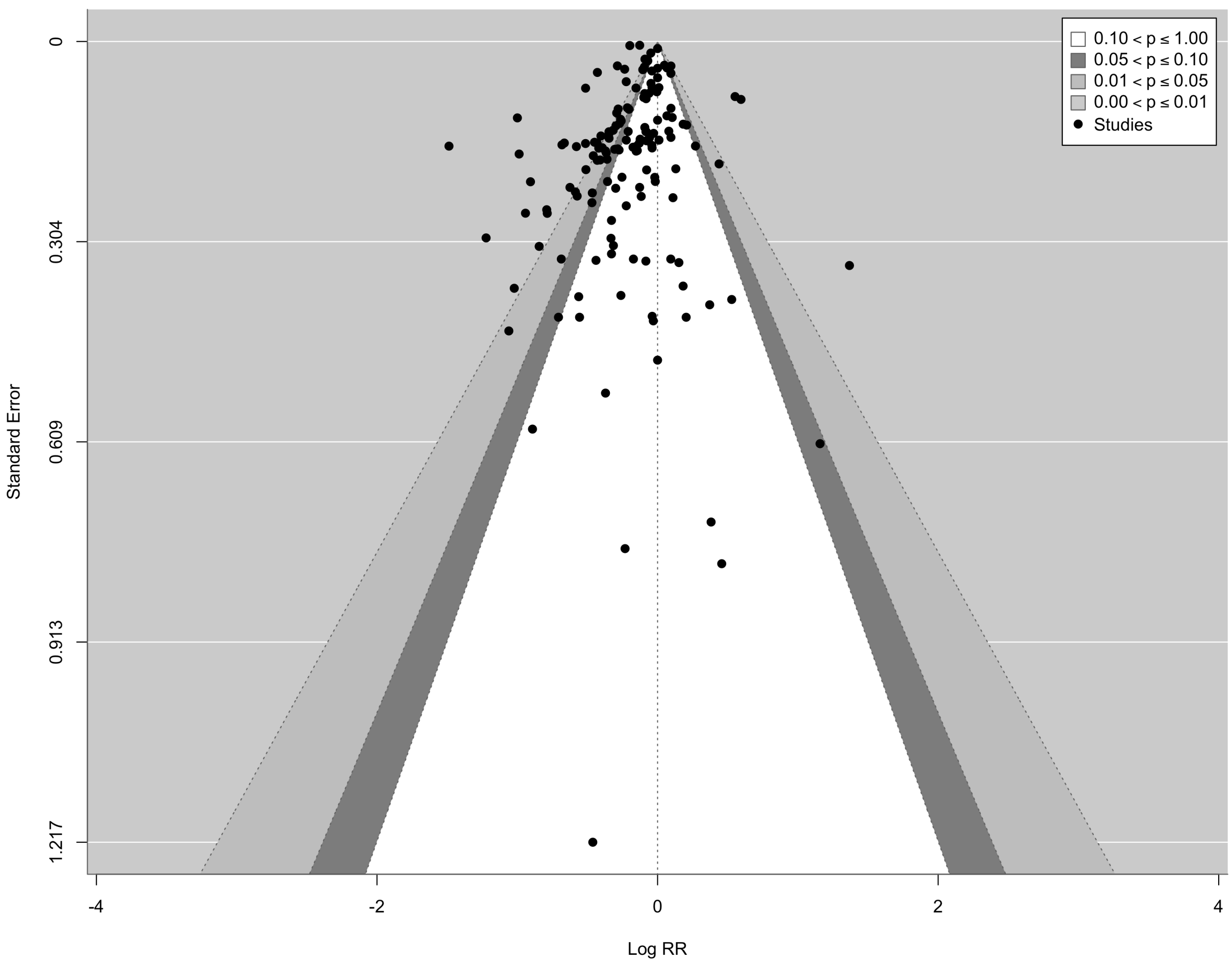

Supplementary Figure S4. Contour-enhanced funnel plot for assessment of publication bias in studies assessing the association between physical activity and the risk of all-cause dementia.

Contour-enhanced funnel plot of log relative risks against their standard errors. The vertical dashed line indicates the pooled effect estimate, and the diagonal lines represent the expected 95% confidence limits in the absence of heterogeneity. Shaded regions correspond to levels of statistical significance (white: $p \geq 0·10$; dark gray: $0·05 \leq p < 0·10$; light gray: $p < 0·05$).

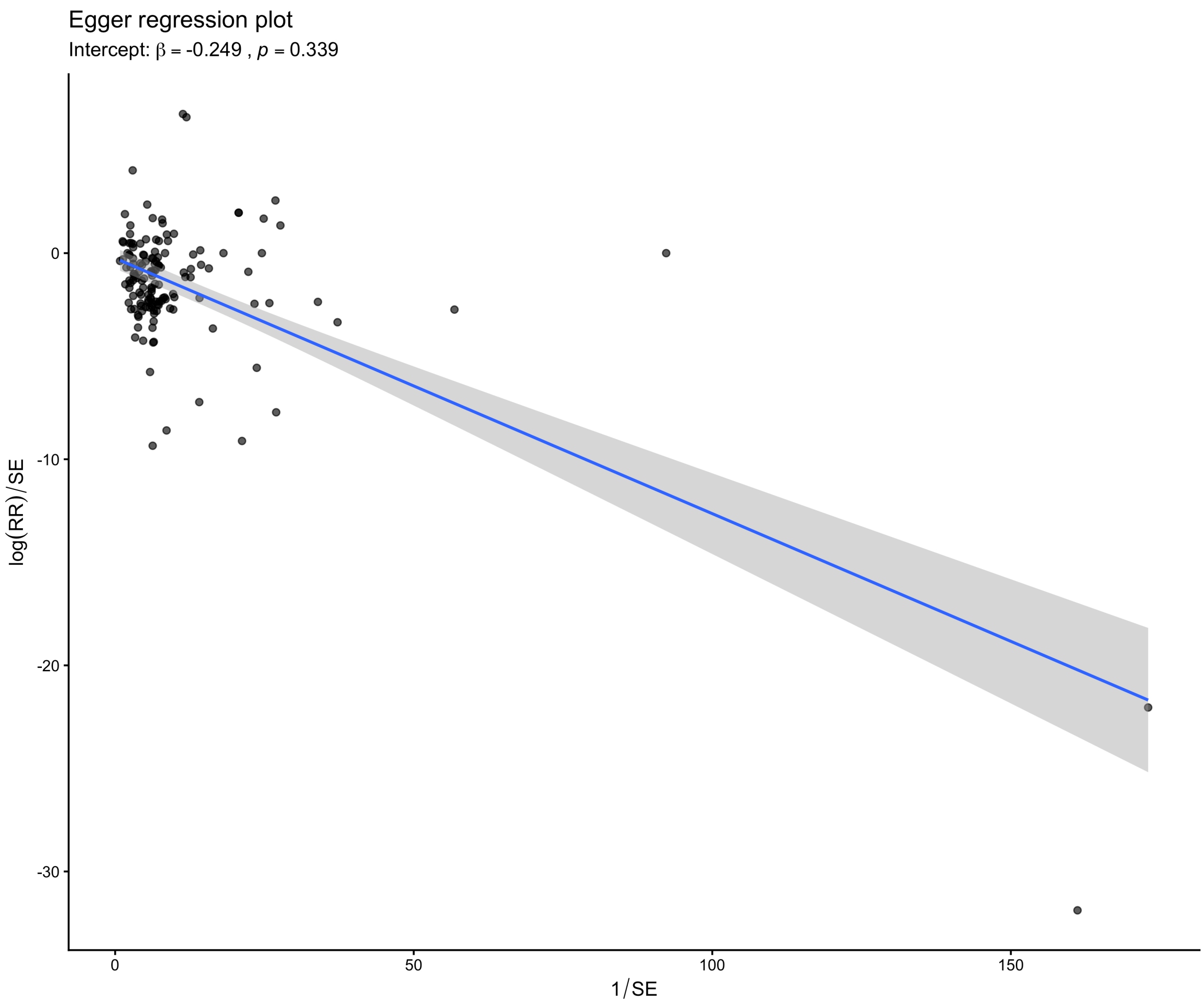

Supplementary Figure S5. Egger regression plot assessing small-study effects in the association between physical activity and all-cause dementia.

Egger regression plot showing the association between study precision (1/standard error) and the standardized effect size (log relative risk divided by standard error). Each point represents an individual study. The solid blue line corresponds to the fitted linear regression, with the shaded area indicating the 95% confidence interval.

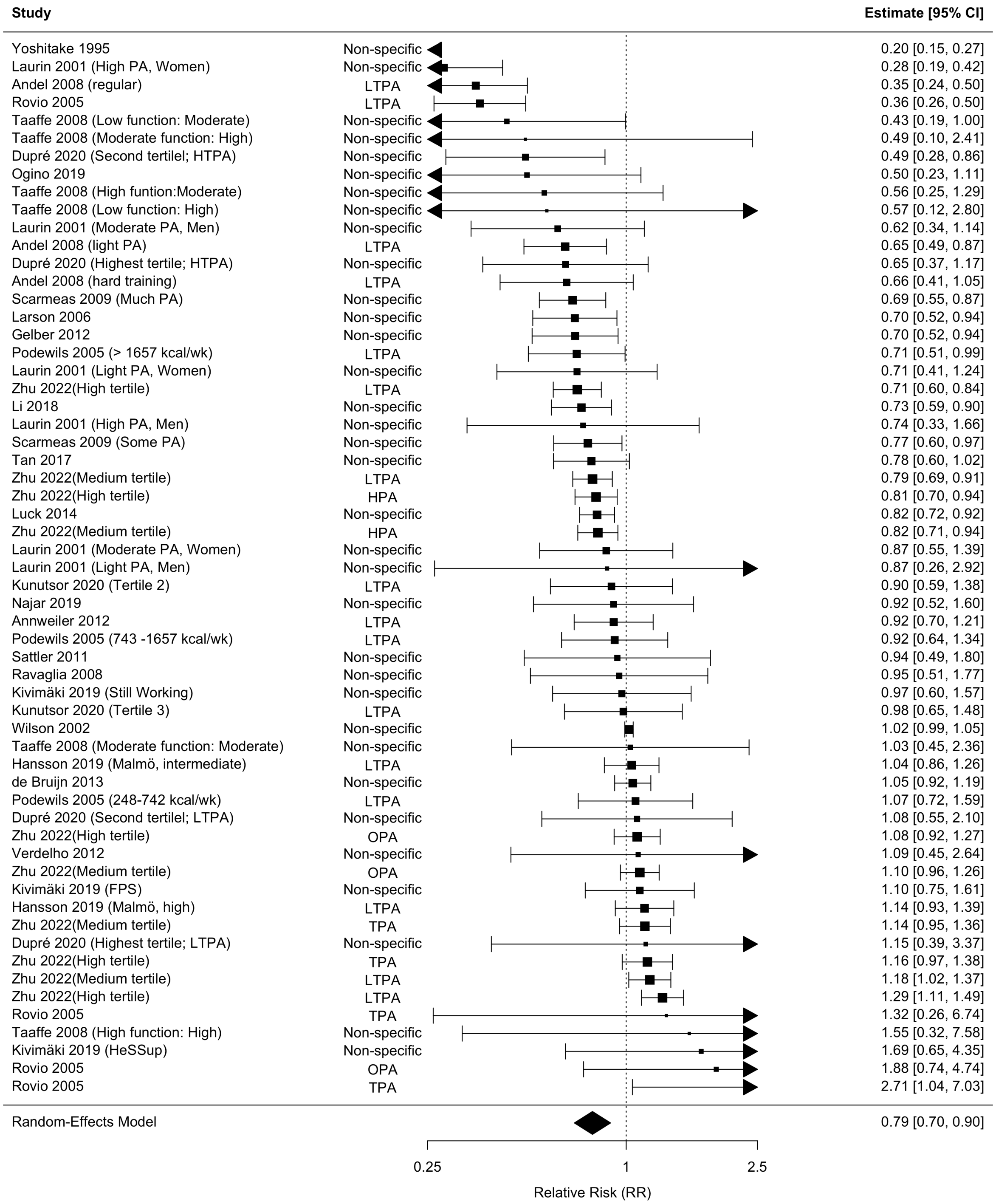

Supplementary Figure S6. Pooled association between physical activity and risk of Alzheimer's disease.

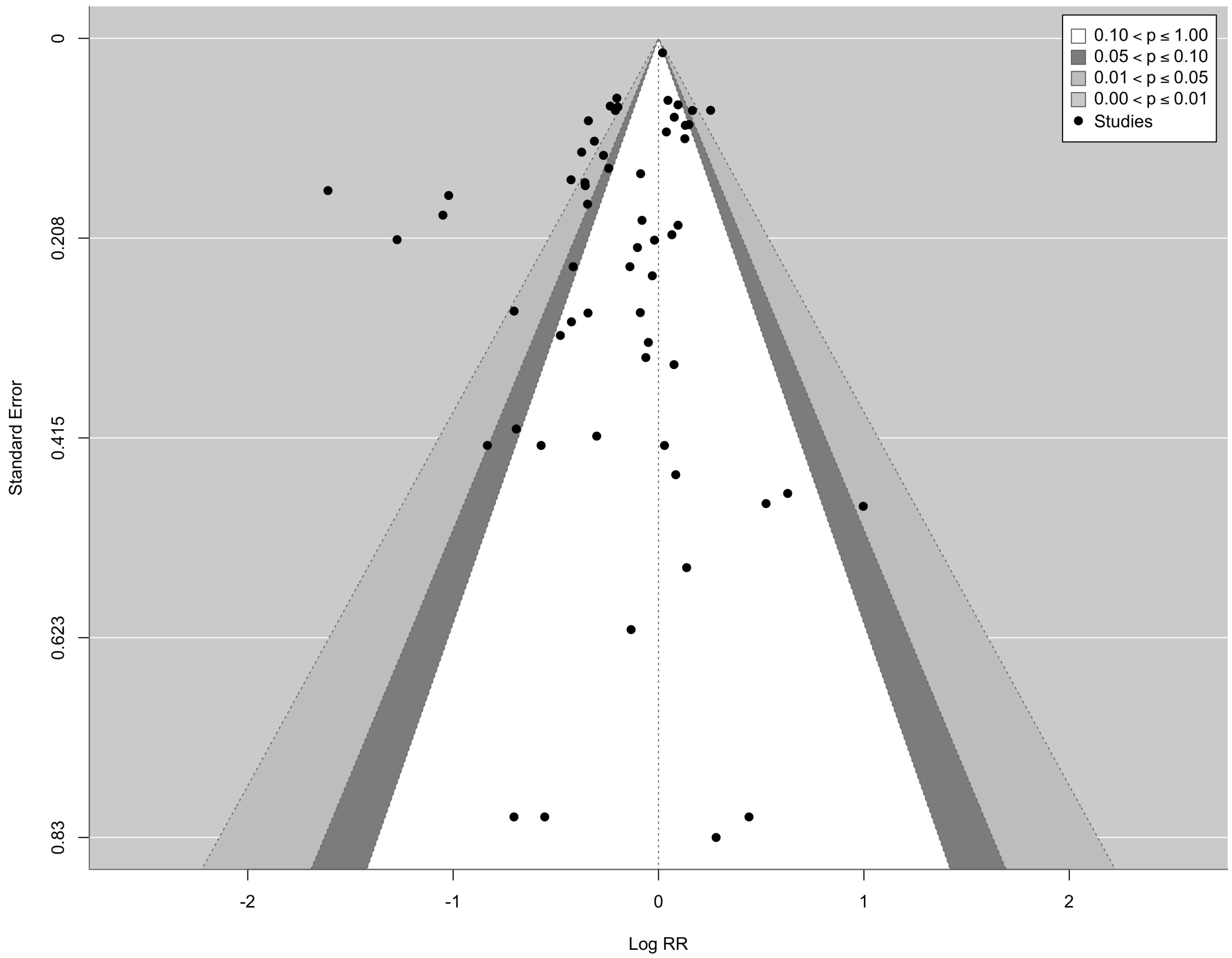

Supplementary Figure S7. Contour-enhanced funnel plot for assessment of publication bias in studies assessing the association between physical activity and the risk of Alzheimer's disease.

Contour-enhanced funnel plot of log relative risks against their standard errors. The vertical dashed line indicates the pooled effect estimate, and the diagonal lines represent the expected 95% confidence limits in the absence of heterogeneity. Shaded regions correspond to levels of statistical significance (white: $p \geq 0{\cdot}10$; dark gray: $0{\cdot}05 \leq p < 0{\cdot}10$; light gray: $p < 0{\cdot}05$).

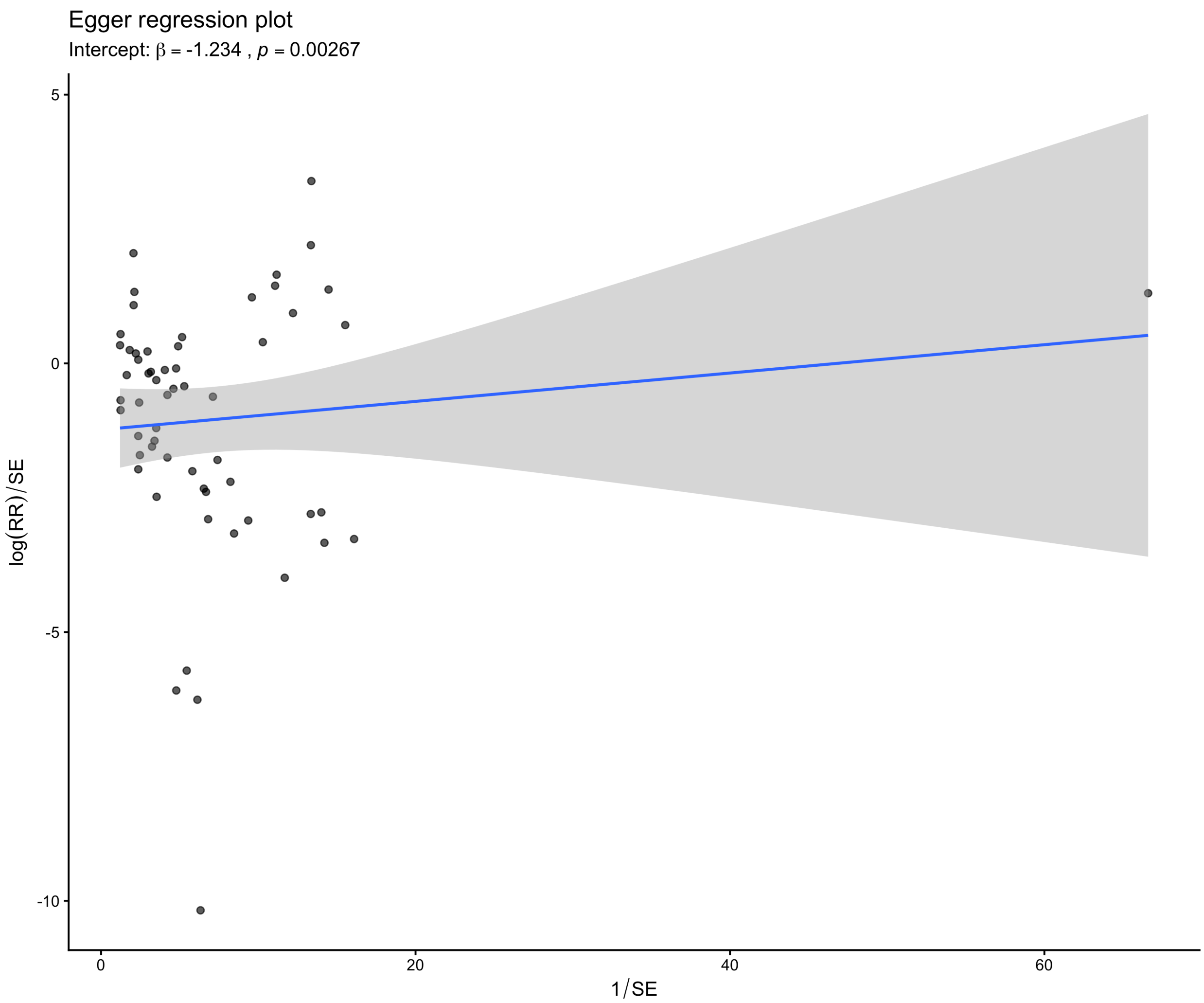

Supplementary Figure S8. Egger regression plot assessing small-study effects in the association between physical activity and Alzheimer's disease.

Egger regression plot showing the association between study precision (1/standard error) and the standardized effect size (log relative risk divided by standard error). Each point represents an individual study. The solid blue line corresponds to the fitted linear regression, with the shaded area indicating the 95% confidence interval.

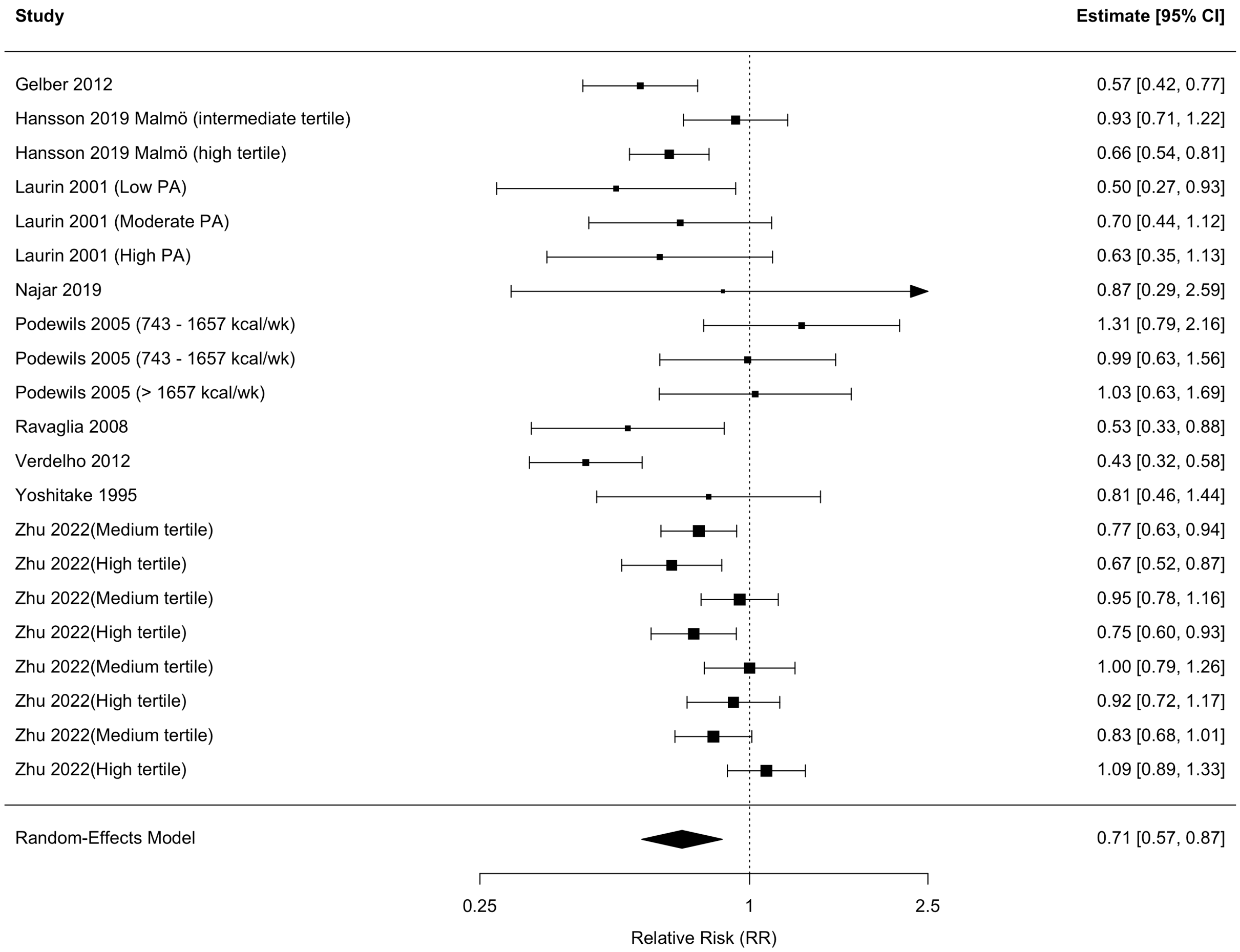

Supplementary Figure S9. Pooled association between physical activity and risk of vascular dementia.

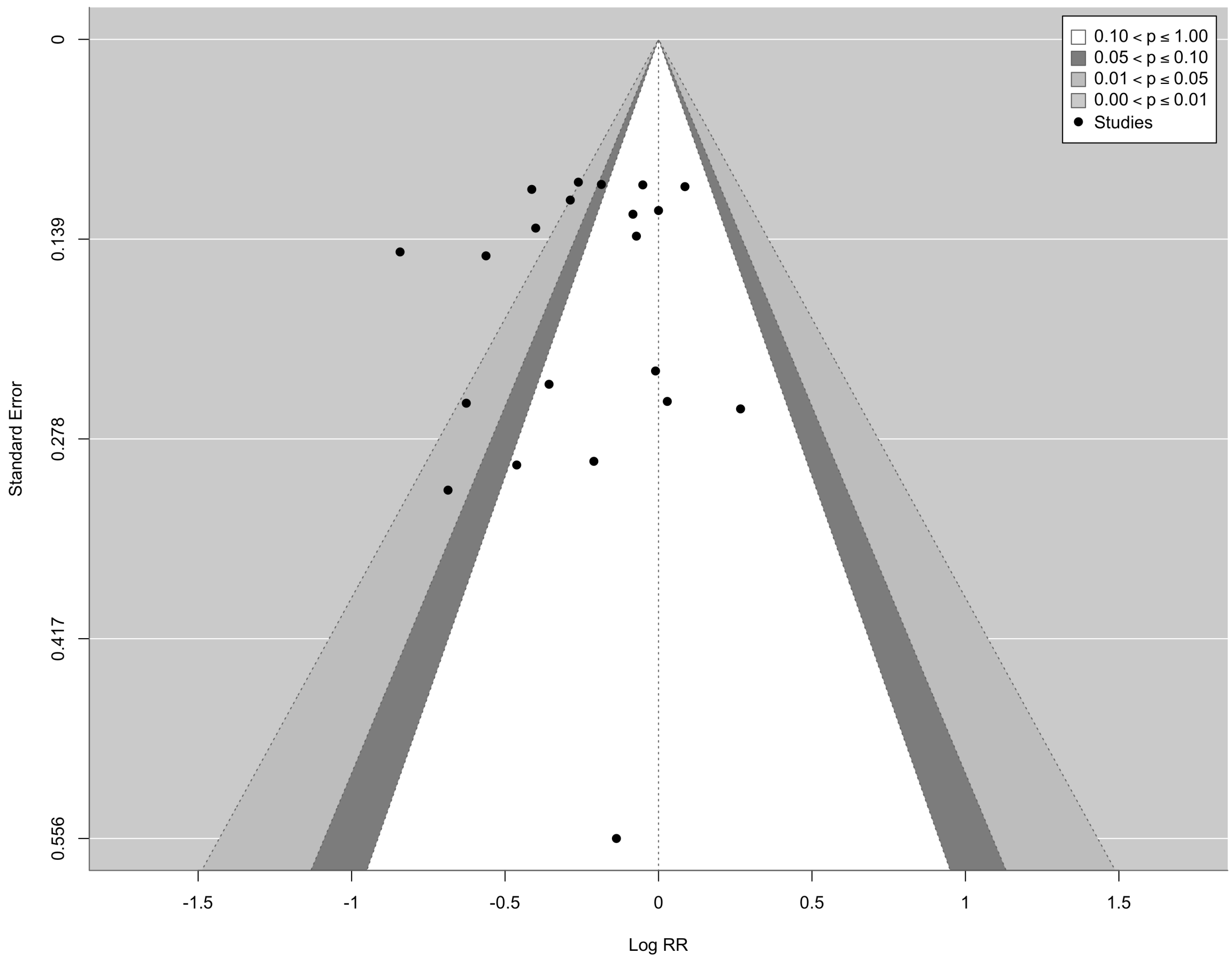

Supplementary Figure S10. Contour-enhanced funnel plot for assessment of publication bias in studies assessing the association between physical activity and the risk of vascular dementia.

Contour-enhanced funnel plot of log relative risks against their standard errors. The vertical dashed line indicates the pooled effect estimate, and the diagonal lines represent the expected 95% confidence limits in the absence of heterogeneity. Shaded regions correspond to levels of statistical significance (white: $p \geq 0{\cdot}10$; dark gray: $0{\cdot}05 \leq p < 0{\cdot}10$; light gray: $p < 0{\cdot}05$).

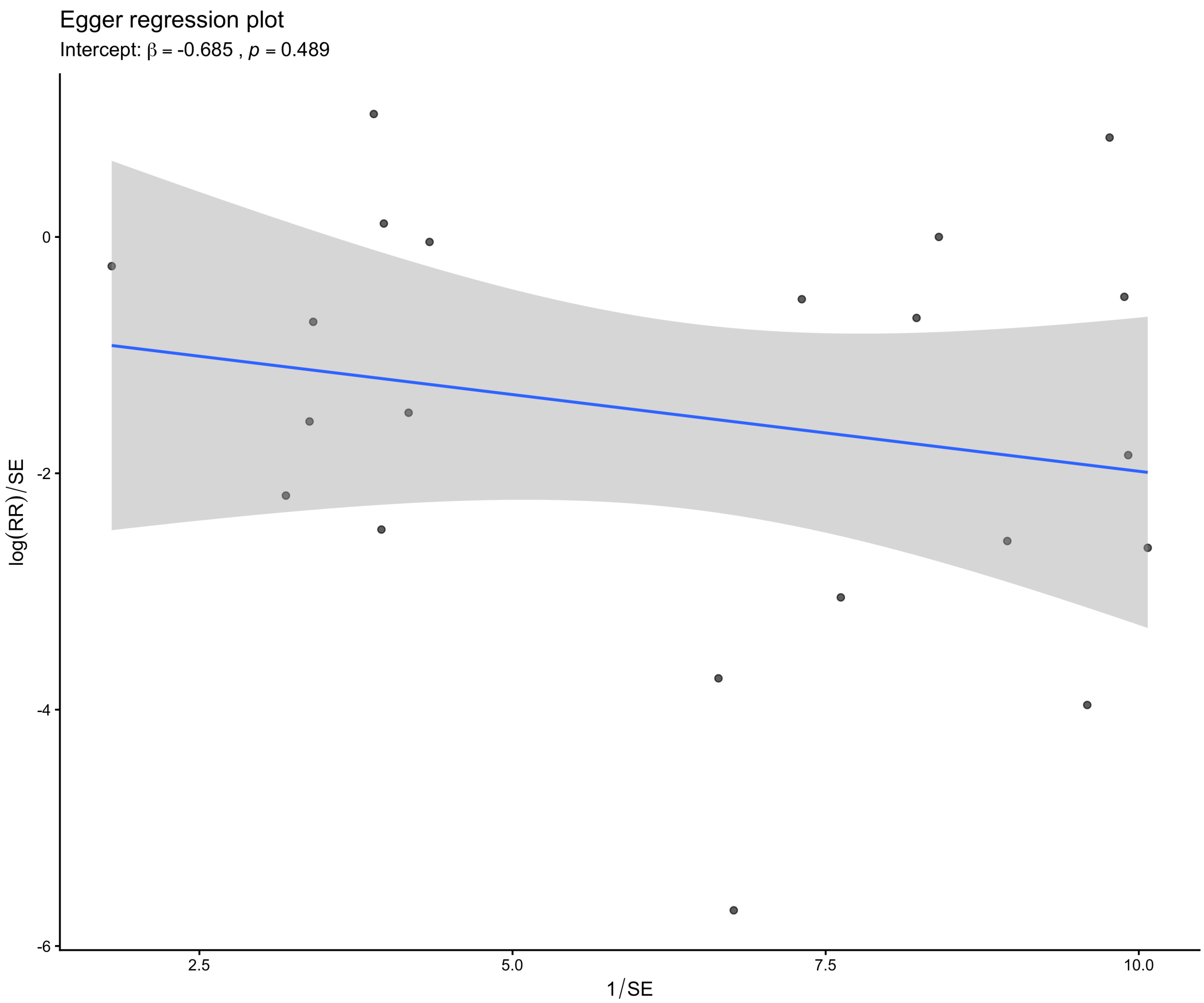

Supplementary Figure S11. Egger regression plot assessing small-study effects in the association between physical activity and vascular dementia.

Egger regression plot showing the association between study precision (1/standard error) and the standardized effect size (log relative risk divided by standard error). Each point represents an individual study. The solid blue line corresponds to the fitted linear regression, with the shaded area indicating the 95% confidence interval.

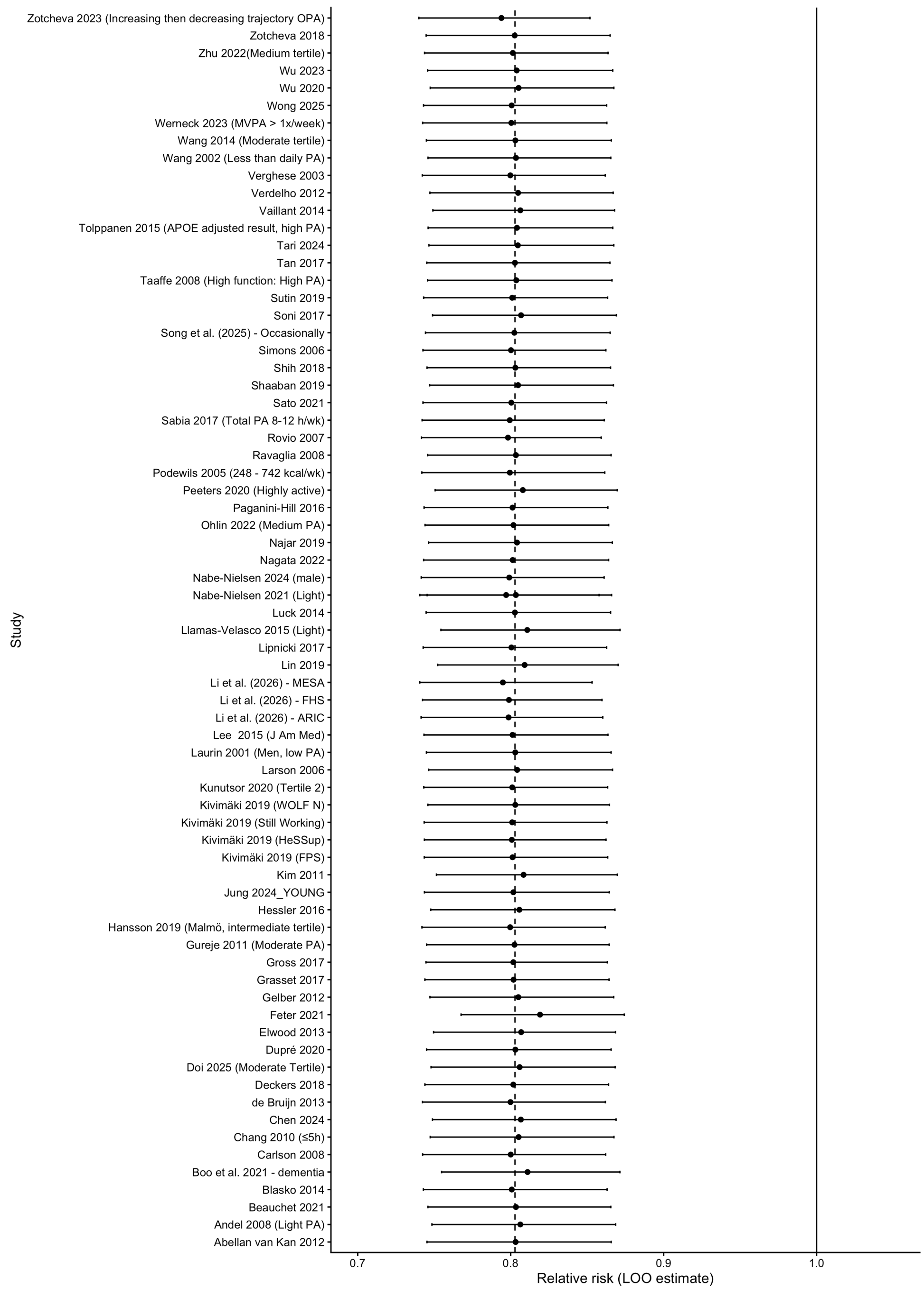

Supplementary Figure S12. Leave-one-out sensitivity analysis for the association between physical activity and risk of all-cause dementia.

The plot shows how the removal of each study affects the pooled log relative risk (RR) estimated using a random-effects multilevel model. For each omitted study, the point estimate and 95% confidence interval (CI) are displayed. The dashed line marks the pooled estimate using all studies, while the solid line represents the null value. The analysis indicates that no single study disproportionately influenced the overall findings.

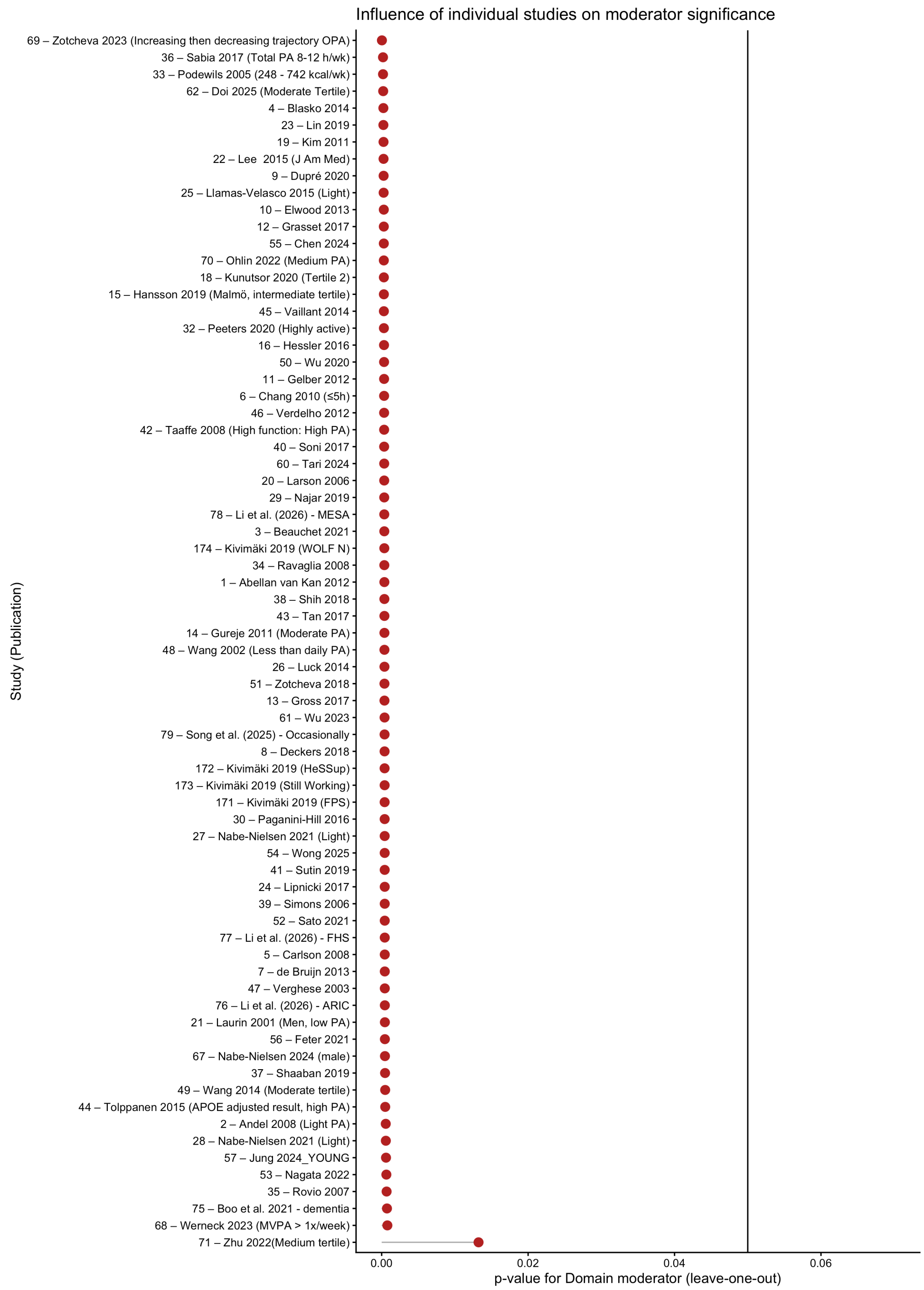

Supplementary Figure S13. Influence of individual studies on the statistical significance of the moderating effect of physical activity domains.

The figure presents a leave-one-out sensitivity analysis in which each study was sequentially removed, and the p-value of the moderator was re-estimated using a multilevel random-effects model. Points denote the p-value for each iteration, and horizontal lines show the range from zero to the observed value. The solid vertical line at p=0·05 indicates the conventional significance threshold. All re-estimated p-values remained below this threshold, demonstrating that the moderating effect was robust and not driven by any single study.

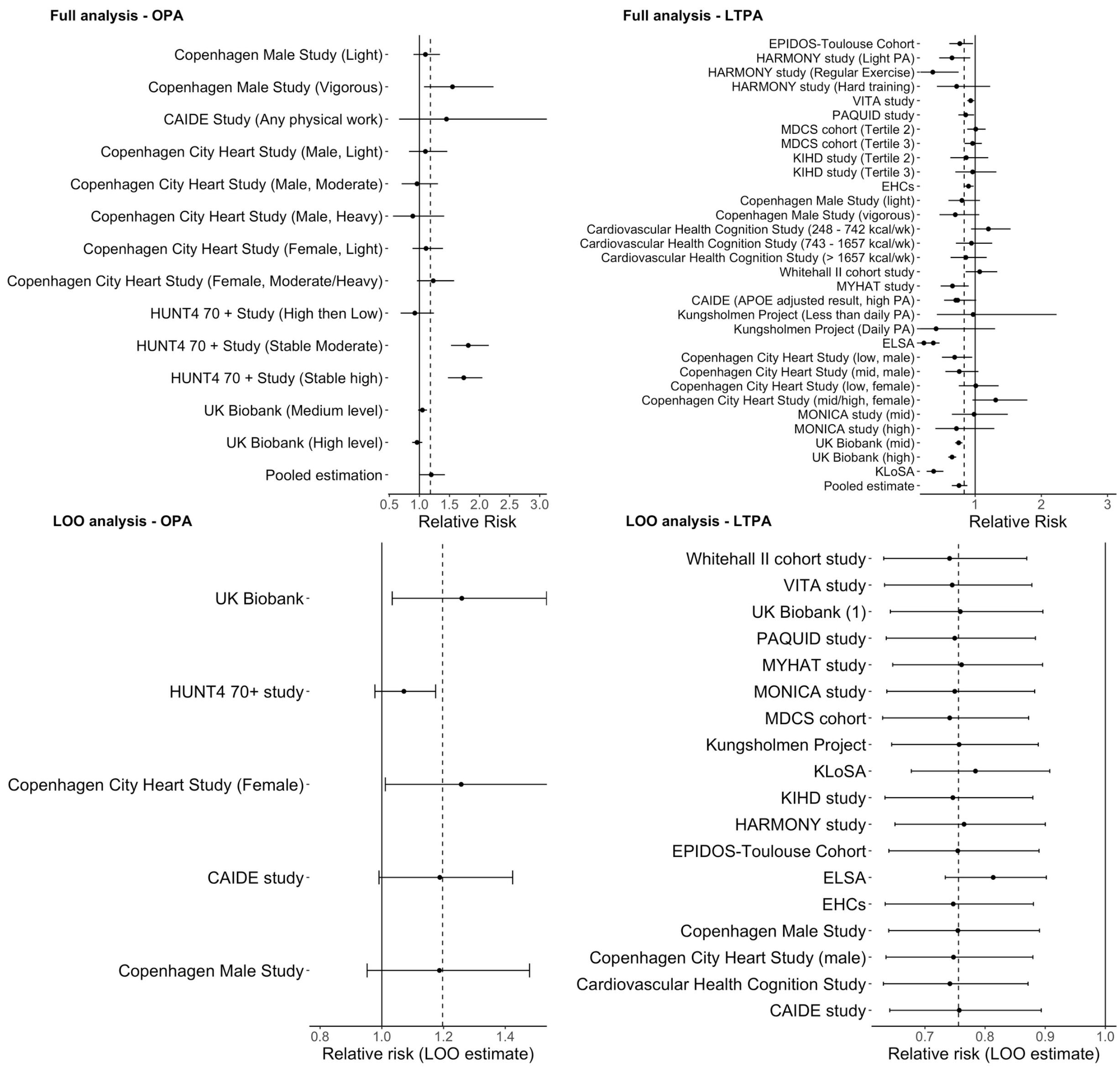

Supplementary Figure S14: Association between domain-specific physical activity and dementia risk, including leave-one-out sensitivity analyses.

Forest plots show study-specific and pooled relative risks (RRs) for the association between physical activity and incident dementia, stratified by physical activity domain. Panels display occupational physical activity (upper left), leisure-time physical activity (upper right), and corresponding leave-one-out (LOO) sensitivity analyses for occupational (lower left) and leisure-time physical activity (lower right).

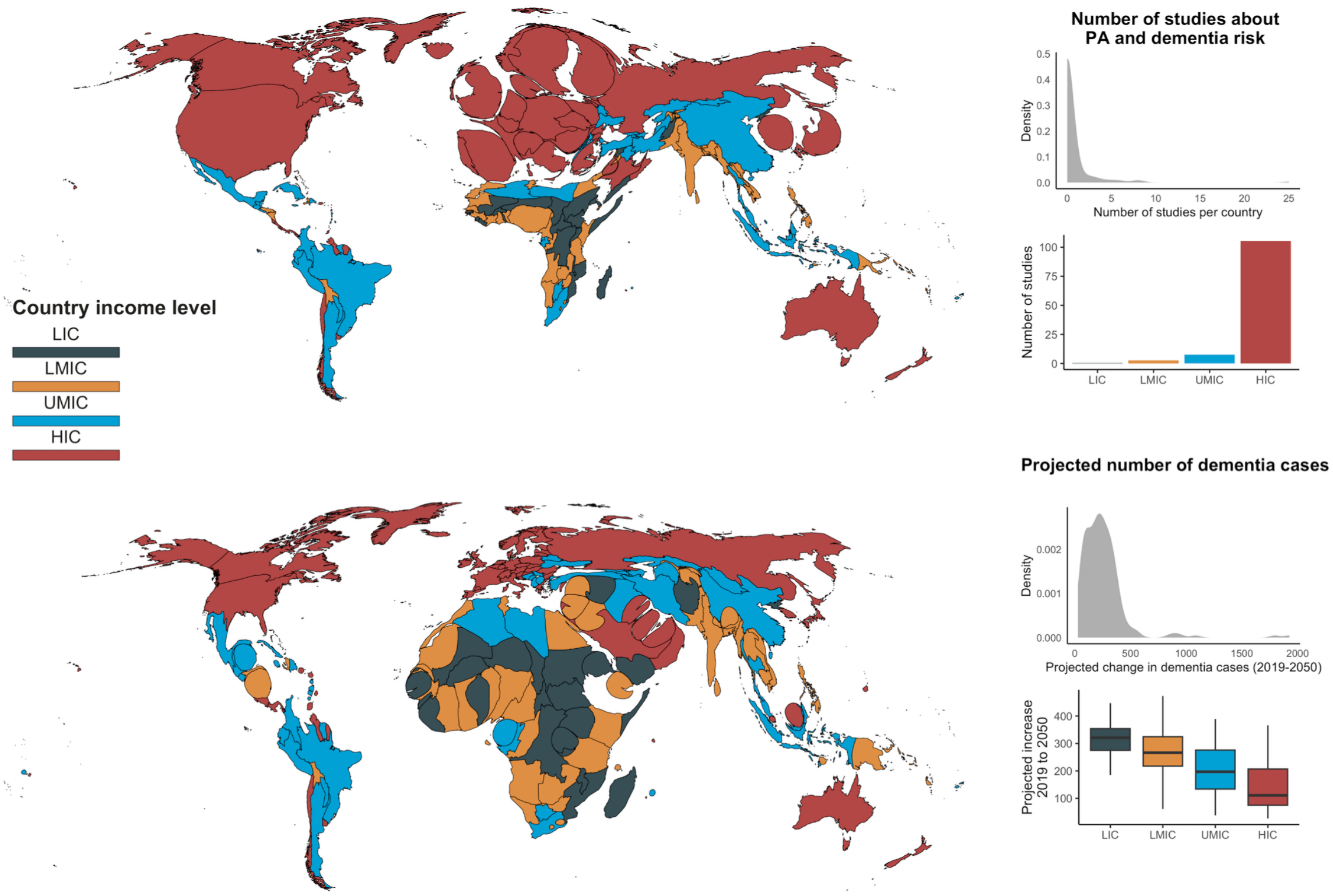

Supplementary Figure S15. Mismatch between the projected number of cases of dementia from 2019 to 2050 (upper map) and the number of studies conducted in each country (lower map) by the World Bank's country income level.

Projected changes in the number of dementia cases from 2019 to 2050 extracted from a previous publication using data from the Global Burden of Disease Study 2019.[26] Values were reported as relative changes (%) in the number of cases from 2019 to 2050. At the top: country sizes are distorted based on the number of studies on physical activity and dementia risk. The density plot shows the distribution of the number of studies per country. The box plot illustrates the number of studies by country income level. At the bottom: country sizes are distorted to reflect the projected increase in the number of people living with dementia between 2019 and 2050. The density plot shows the distribution of the projected relative change across countries. The box plot illustrates the projected relative change by country income level. LIC: low-income countries; LMIC: lower-middle-income countries; UMIC: upper-middle-income countries; HIC: high-income countries.

Supplementary Table S1. Characteristics of the included studies.

Author (year)	Design	Country of origin	N	cases	% women	Dementia criteria	Reference category	Exposure	Age at baseline	PA DOMAINS[1]				Follow-up (years)	Quality
										LTPA	OPA	HPA	CPA		
Abellan van Kan et al.[27]	Prospective cohort study	France	1462	145	100.0%	DSM-IV/NIH-AA criteria	< 1h of PA/week	At least one PA for at least 1 hour a week	at least 75 years	X	-	-	-	7	Poor
Andel et al.[28]	Case-control study	Sweden	3134	264	61.0%	Clinical evaluation	Self-report of "Hardly any PA"	Self-report of walking or light gardening/ regular exercise/ hard training	48.1 (4.9 SD)	X	-	-	-	31	Moderate
Annweiler et al.[29]	Prospective cohort study	France	489	70	100.0%	Clinical evaluation	Lower 4 quintiles combined	Highest quintile of vitamin D dietary intakes	mean 79.8 ± 3.8 years	-	-	-	-	7	
Beauchet et al.[30]	Prospective cohort study	Canada	1098	39	52.2%	MMSE+IADL	PA for the Elderly Score : score below the lowest tertile	PA for the Elderly Score: score above the lowest tertile	73.8	-	-	-	-	3	Poor
Blasko et al.[31]	Prospective cohort study	Austria	399	117	59.8%	Clinical evaluation		Composite score of PA (diversity)	75.8 years	X	-	-	-	5	Poor
Boo et al.[32]	Prospective cohort study	South Korea	4289		52.5%	MMSE	no regular exercise	regular exercise	45 or older	X	-	-	-	0	Poor
Carlson et al.[33]	Case-control study	United States	294	147	0.0%	DSM-III R		Summary score of gardening, sports and outdoor activities	44.7 years	-	-	-	-	28	Poor
Chang et al.[34]	Prospective cohort study	Iceland	4761	184	42.1%	DSM-IV	None	≤5h of PA per week/ > 5h of PA per week	51 years	-	-	-	-	26	Moderate
Chen et al.[35]	Prospective	United States	5199	1089	58.0%	Cognitive test	No moderate activity 5 days a week or vigorous 1 day a week	Physically active(moderate exercise 5 days/week or vigorous 1 day/week	60+(73.4)	-	-	-	-	14	Moderate
de Bruijn et al.[36]	Prospective cohort study	the Netherlands	4406	583	59.0%	DSM-III-R		SDs of log transformed MET hours per week	72.7 (7.2 SD) years	-	-	-	-	8.8	Good
Deckers et al.[37]	Prospective cohort study	United Kingdom	323	76	71.2%	DSM-III-R	No form of PA or exercise during the preceding week	One or more forms of PA or exercise (e.g. walking, cycling, do-it-yourself, gardening, etc.) during the preceding week	88.0	-	-	-	-	18	Poor
Doi et al.[38]	Prospective	Japan	2740	326	58.8%	Long-term care insurance certification + health records	Lowest tertile	AMI physical subscore: second tertile; AMI physical subscore: third tertile	74.4	-	-	-	-	4.475	Poor

Author (year)	Design	Country of origin	N	cases	% women	Dementia criteria	Reference category	Exposure	Age at baseline	PA DOMAINS[1]				Follow-up (years)	Quality
										LTPA	OPA	HPA	CPA		
Dupré et al.[39]	Prospective cohort study	France	1550	117	63.6%	DSM-IV/NIH-AA criteria	None	Total PA amount second tertile (intensity*(times/month)*(months/year))/Total PA amount highest tertile (intensity*(times/month)*(months/year))	72-97 years (mean not given)	-	-	-	-	4.6	Moderate
Elwood et al.[40]	Prospective cohort study	United Kingdom	1225	79	0.0%	Clinical evaluation	< 2miles walking to work daily and < 10 miles cycling to work daily and vigorous exercise is not a habit	walking ≥2 miles to work each day, or cycling ≥10 miles to work each day, or 'vigorous' exercise described as a regular habit	45-59 years (mean, spread not told)	-	-	-	-	30	Moderate
Feter et al.[41]	Prospective	United Kingdom	9275	631	56.2%	Self-reported + IQCODE	Responded hardly ever or never exercised	Low activity; Moderate/High activity	50+(63.8)	X	-	-	-	14	Good
Gelber et al.[42]	Case-control study nested in prospective cohort study	United States	3468	223	0.0%	DSM-IV/NIH-AA criteria	Three lower quartiles (the highest mean 7.2h light PA or 4.4h moderate PA per day)	Highest quartile of light or moderate PA (mean 7.2 h light PA/day or 4.4 h of moderate activity)	52 years	-	-	-	-	25	Good
Grasset et al.[43]	Longitudinal cohort study	France	3595	859	59.2%	DSM-III-R	Self-report of "no sports practice"	Self-report of "sports practice"	At least 65 years (mean not presented and a reference article is not available)	X	-	-	-	11.57	Poor
Gross et al.[44]	Prospective cohort study	United States	646	28	7.4%	Clinical evaluation	Lowest 90% of MET h/day	Highest 10% of MET h/day	47.1 (5.1 SD)	-	-	-	-	33	Moderate
Gureje et al.[45,46]	Prospective cohort study	Nigeria	2149	85	53.8%	DSM-IV	< 6 MET-h/week	≥3 days of VPA of at least 20 min/day OR ≥5 days of MPA or walking of ≥ 30 min/day/ VPA≥3 days and accumulating at least 1500 MET-min/week or any combination of light, MPA or VPA ≥ 3000 MET-min/week	74.5 (SD 8.4)	-	-	-	-	3.3	Moderate
Hansson et al.[46]	Prospective cohort study	Sweden	20639	1375	60.0%	DSM-5	Lowest tertile	Second tertile of MET/ Highest tertile of MET	median 57.5 (IQR 51.0–63.8)	X	-	-	-	15	Poor

Author (year)	Design	Country of origin	N	cases	% women	Dementia criteria	Reference category	Exposure	Age at baseline	PA DOMAINS[1]				Follow-up (years)	Quality
										LTPA	OPA	HPA	CPA		
Hessler et al.[47]	Prospective population-based study	Germany	3547	296	59.2%	Health records	No VPA	VPA ≥ 3 times per week; VPA 1-2 times/week	67.28 (SD 7.57)	-	-	-	-	6.7	Poor
Jung et al.[5]	Prospective	South Korea	3248171	22152	53.6%	Health records	Sedentary(No engagement in PA)	Insufficient(Some PA but does not meet standards; Active(5x/week for 30mins or high intensity 3x/week for 20 mins	40-59	-	-	-	-	10	Good
Kim et al.[48]	Prospective cohort study	South Korea	518	45	57.3%	Clinical evaluation		a 4-point scale (very active, fairly active, not very active, not at all active) of PA	71.8 years (SD 4.9 -5.7)	-	-	-	-	2.4	Poor
Kivimäki et al.[49] (FPS)	Prospective cohort study	Finland	46529	306	80.7%	Health records	Less than 0.5 hour of each (brisk walking, jogging, or running) per week	More than 0.5 hour of any of the following (brisk walking, jogging, or running) per week.	44.6	-	-	-	-	15.5	Good
Kivimäki et al.[49] (HeSSup)	Prospective cohort study	Finland	23842	81	59.1%	Health records	Less than 0.5 hour of each (brisk walking, jogging, or running) per week.	More than 0.5 hour of any of the following (brisk walking, jogging, or running) per week.	36.7	-	-	-	-	14.8	Poor
Kivimäki et al.[49] (Still Working)	Prospective cohort study	Finland	9058	138	22.3%	Health records	Sport activities less than a couple of times per month	Sport activities more than a couple of times per month	40.9	-	-	-	-	21.6	Moderate
Kivimäki et al.[49] (WOLF N)	Prospective cohort study	Sweden	4699	9	16.7%	Health records	No or very little exercise, only occasional walks.	More than no or very little exercise, only occasional walks.	44.1	-	-	-	-	12.6	Moderate
Kunutsor et al.[50]	Prospective cohort study	Finland	2394	208	0.0%	Health records	Lowest tertile (0.53-239.42 MET-h/y)	Conditioning LTPA: Middle tertile (239.43-677.64 MET-h/y); Conditioning LTPA: Highest tertile (677.65-8981.03 MET-h/y)	53	X	-	-	-	24.9	Moderate
Larson et al.[51]	Prospective cohort study	United States	1740	158	60.2%	DSM-IV/NIH-AA criteria	Exercise < 3 times/week (15min duration)	PA ≥3 times per week	73.8 years	-	-	-	-	6.2	Poor
Laurin et al.[52]	Prospective cohort study	Canada	4615	285	60.3%	DSM-IV	No PA	PA <3 times per week; PA ≥3 times per week at an intensity of walking; PA ≥3 times per week at an intensity greater than walking	Not told from at least 65 years to over 85 years	-	-	-	-	5	Moderate

Author (year)	Design	Country of origin	N	cases	% women	Dementia criteria	Reference category	Exposure	Age at baseline	PA DOMAINS[1]				Follow-up (years)	Quality
										LTPA	OPA	HPA	CPA		
Lee et al.[53]	Prospective cohort study	Hong Kong, China	15589	1350	63.9%	Clinical evaluation	No "aerobic exercise" (not specified further)	Aerobic exercise (not specified further)	74,2 years (range or SD not given)	X	-	-	-	6	Moderate
Li et al.[54]	Prospective longitudinal cohort	United States	1097	121	74.0%	NINCDS-ADRDA clinical criteria	Preserved fractal regulation of motor activity (higher FR)	Degraded fractal regulation of motor activity (10th percentile FR)	~80.9 ± 7.2 years	-	-	-	-	11-13	Moderate
Li et al.[8] (ARIC)	Prospective cohort study	United States	15549	2463	55.11%	DSM-IV	MET min/week = 0	MET min/week > 0	54.1± 5.7 years	-	-	-	-	23.4	Moderate
Li et al.[8] (FHS – Original cohort)	Prospective cohort study	United States	3142	498	55.90%	DSM-IV	MET min/week = 0	MET min/week > 0	47.9 ± 3.5 years combining Original and Offspring) – 45-64 age range	-	-	-	-	41.3	Moderate
Li et al.[8] (FHS – Offspring cohort)	Prospective cohort study	United States	4044	640	55.90%	DSM-IV	MET min/week = 0	MET min/week > 0	± 7.2 years combining Original and Offspring) – 45-64 age range	-	-	-	-	37.9	Moderate
Li et al.[8] (MESA)	Prospective cohort study	United States	3819	605	53.23%	DSM-IV	MET min/week = 0	MET min/week > 0	54.5± 5.6 years	-	-	-	-	14.3	Moderate
Lin et al.[55]	Prospective cohort study	China	2074	91	47.1%	MMSE/ MOCA	Physical activities of (1) Time: <20 min/day or Intensity: lower than moderate intensity; (2) Frequency <1 day/week.	PA meeting the following criteria: (1) Time: ≥20 min/day; (2) Intensity: moderate intensity (i.e., brisk walking, jogging, climbing stairs, etc.) and/or vigorous intensity (i.e., long-distance running, rope skipping, basketball, etc.); (3) Frequency ≥4 days/week.	70.3 years	-	-	-	-	1	Poor
Lipnicki et al.[56]	Prospective cohort study	Australia	873	83	56.1%	DSM-IV		Number of PA activities	78.7 (4SD 4.8) years	-	-	-	-	6	Poor
Llamas-Velasco et al.[57]	Prospective cohort study	Spain	3105	134	56.6%	DSM-IV	Sedentary lifestyle (i.e., only minimal house chores or short walks at home)	Regular house chores, walks independently at home/ Regular house chores, walks up to one kilometer per day/	75,96 years (SD, range not given)	-	-	-	-	3.2	Moderate

Author (year)	Design	Country of origin	N	cases	% women	Dementia criteria	Reference category	Exposure	Age at baseline	PA DOMAINS[1] LTPA	OPA	HPA	CPA	Follow-up (years)	Quality
								Heavy housework, walks more than one kilometer or practices any sport regularly							
Luck et al.[58]	Prospective cohort study	Germany	2492	278	64.7%	DSM-III-R		Number of PA activities	81.1 (SD 3.5) years	-	-	-	-	4.5	Poor
Nabe-Nielsen et al.[59]	Prospective cohort study	Denmark	6603	1431	52.3%	Health records	LTPA: Sedentary; OPA: Sedentary	LTPA: Moderate PA, High PA;<br>OPA: Light physical work, Moderate physical work, Heavy physical work	51.7	X	X	-	-	20.2-24.8 for men; 21.7-26.7 for women	Moderate
Nabe-Nielsen et al.[6]	Prospective cohort study	Denmark	4721	697	0.0%	Health records	Mainly sedentary	LTPA: Light PA at least 4 hours per week/ VPA for at least 3 hours per week or high intensity, competitive sport regularly (that is several times/week); OPA: Light physical work/ Heavy physical work	48.9	X	X	-	-	29.3	Moderate
Nagata et al.[60]	Prospective	Japan	3722	471	46.4%	Long-term care insurance certification	0 min/week	MPA: 10-149 min/week/150-299 min/week/>=300 min/week;<br>VPA: 10-74 min/week/75-149 min/week/>=150 min/week;<br>Total MVPA: 33-599 min/week/600-1199 min/week/ >=1200 min/week	mean (SD), 73.00 (6.0)	-	-	-	-	7.38	Moderate
Najar et al.[61]	Prospective cohort study	Sweden	800	194	100.0%	Health records	Completely inactive, for example at most watching television and going to the movies	Light PA for a minimum of 4 h/week, such as walking, gardening, bowling, or cycling for half an hour a day, or regular physical training, such as running, tennis, or swimming, for at least 2–3 h/week, or regular–intense physical training such as heavy exercise, for example running or swimming several times/week, or	47.2	-	-	-	-	44	Moderate

Author (year)	Design	Country of origin	N	cases	% women	Dementia criteria	Reference category	Exposure	Age at baseline	PA DOMAINS[1]				Follow-up (years)	Quality
										LTPA	OPA	HPA	CPA		
								engaging in competitive sports.							
Ogino et al.[62]	Prospective cohort study	United States	1345	106	68.0%	DSM/ NINCDS-ADRDA criteria	Low LTPA (lowest category per weekly LTPA)	High LTPA (highest level of self-reported current and past LTPA)	75.1 ± 6.30 years	X	-	-	-	4.13	Moderate
Ohlin et al.[63]	Prospective cohort study	Sweden	541	175	56.2%	DSM-IV	Low PA	Medium PA/ High PA	64.9	X	-	-	-	19	Moderate
Paganini-Hill et al.[64]	Prospective cohort study	United States	587	268		DSM-IV	at most 1h of vigorous exercise/week	≥2 h/d of vigorous exercise	93 (SD 2.6, range 90-103) years	-	-	-	-	23	Poor
Peeters et al.[65]	Prospective cohort study	Cuba	1846	169	66.3%	DSM-IV	Not very active	Somewhat active/ Highly active	70	-	-	-	-	4.5	Poor
Podewils et al.[66]	Prospective cohort study	United States	3375	480	59.1%	NINCDS-ADRDA	Lowest quartile (PA energy expenditure < 248 kcal/week)	PA energy expenditure (248 - 742 kcal/week)/ PA energy expenditure (743 - 1657 kcal/week)/ PA energy ependiture (> 1657 kcal/week)	74.8 years (SD, range not given)	X	-	-	-	6	Poor
Ravaglia et al.[67]	Prospective cohort study	Italy	749	86	53.5%	DSM-IV	Not meeting the PA guidelines	Meets the PA quidelines	73.2 (SD 6.0) years	-	-	-	-	3.9	Moderate
Rovio et al.[68]	Prospective cohort study	Finland	1449	117		Clinical evaluation	Inactive at midlife (no/low LTPA)	LTPA ≥ twice per week at midlife	65-79 years	X	-	-	-	21	Moderate
Rovio et al.[69]	Prospective cohort study	Finland	1158	44	61.0%	DSM-IV	Sedentary work	Physical work	50.4 (6.0 SD) years	-	X	-	-	21	Good
Sabia et al.[70]	Prospective cohort study	United Kingdom	10308	329	33.1%	Health records	MVPA < 2.5h/week	MVPA at least 2.5 h/week	Dementia analyses: 45,0 years	X	-	-	-	26.6	Good
Sato et al.[23]	Prospective	Japan	73260	8714		Health records	None	Each additional one day/week of PA	73.7(65 or older)	-	-	-	-	5	Good
Sattler et al.[71]	Prospective cohort study	Germany	500	137		Clinical evaluation	Failed balance test/physically inactive	Passed balance test (objective PF); sport ≥2 h/week	~61-64 years	X	-	-	-	12	Moderate
Scarmeas et al.[72]	Prospective cohort study	United States	1880	282		Clinical evaluation	No PA (defined as median 0 h/week)	Some or much PA (measured by weekly participation in light/moderate/vigorous PA)	Mean ~77.2 yrs (SD 6.6)	X	-	-	-	5.4	Moderate
Shaaban et al.[73]	Prospective cohort study	United States	1701	109	62.3%	CDR		PA min/week	mean 78 years (interquartile	X	-	-	-	5	Poor

Author (year)	Design	Country of origin	N	cases	% women	Dementia criteria	Reference category	Exposure	Age at baseline	PA DOMAINS[1]				Follow-up (years)	Quality
										LTPA	OPA	HPA	CPA		
									range 71 – 83 years)						
Shih et al.[74]	Prospective cohort study	United States	1438	136	58.4%	DSM-IV	Lowest tertile (< 35 MET-h/week)	Two highest tertiles (≥ 35 MET-hours/week)	70.0	-	-	-	-	6.5	Moderate
Simons et al.[75]	Prospective cohort study	Australia	2805	285	56.0%	Health records	Walking weekly or less often	Walking daily	60 years and older (mean, range, SD not told)	-	-	-	-	16	Poor
Song et al.[76]	Prospective cohort study	China	8758	637	54.9%	Reports from family members or caregivers, confirmed diagnoses by qualified medical institutions, or the use of dementia drugs.	Almost never	Occasionally, Almost daily	Median: 85 years	-	-	-	-	4.3	Poor
Soni et al.[77]	prospective cohort study	United Kingdom	11391	388	56.0%	IQCODE	PA < 1 time/week	Mild PA 1x/week; MVPA 1x/week; MVPA > 1 time/week	65,2 years (Sd and range not given)	-	-	-	-	10	Poor
Sutin et al.[78]	Prospective cohort study	United States	12053	1108	60.0%	Clinical evaluation	Ordinal	Ranging from 1=hardly ever or never to 4=more than once a week	67.31	-	-	-	-	7.4	Poor
Taaffe et al.[79]	Prospective cohort study	United States	2263	173	0.0%	DSM-III-R	Lowest tertile	PA score: highest tertile (frequency, duration, intensity)/ PA score: second tertile (frequency, duration, intensity)	mean 76,6 (range 71-92) years	-	-	-	-	6.1	Moderate
Tan et al.[80]	Prospective cohort study	United States	3714	236	54.4%	DSM-IV	Lowest quintile	Quintiles 2-5	70 (7 SD) years	-	-	-	-	22	Poor
Tari et al.[81]	Prospective	Norway	29826	1998	44.6%	Health records	0 PAI	<= 50 PAI; 51-99 PAI; >= 100 PAI	~50	-	-	-	-	11	Poor
Tolppanen et al.[82]	Prospective cohort study	Finland	1511	250	37.7%	Health records	PA never or few times a year	≤1 time/week or 2-3 times/month PA causing sweating and breathlessness; ≥ 2-3 times/week PA causing sweating and breathlessness	CAIDE target population analyses: 51,2 years CAIDE participants: 50.6 (6.0 SD) years	X	-	-	-	24.4	Good

Author (year)	Design	Country of origin	N	cases	% women	Dementia criteria	Reference category	Exposure	Age at baseline	PA DOMAINS[1]				Follow-up (years)	Quality
										LTPA	OPA	HPA	CPA		
Vaillant et al.[83]	Prospective cohort study	United States	84	44	0.0%	Cognitive test	< 500 kcal/week	≥ 500 kcal of PA/week	45- 60 years	-	-	-	-	30	Poor
Verdelho et al.[84]	Prospective cohort study	Finland, Austria, Sweden, Italy, Switzerland, France, Portugal, Germany, the Netherlands, Denmark	639	90	55.0%	NINCDS-ADRDA	< 30min duration or < 3 times/week	≥ 30 min on ≥ 3 days in a week	74.1 (5 SD) years	-	-	-	-	3	Poor
Verghese et al.[85]	Prospective cohort study	United States	469	124	65.0%	Clinical evaluation		Physically active days (swimming, tennis, babysitting, housework)/week	79.1	-	-	-	-	6.6	Poor
Wang et al.[86]	Prospective cohort study	Sweden	855	123	74.4%	DSM-III-R	None	PA less than daily but not none/<br>PA daily	81.1 (4.9 SD) years	X	-	-	-	6.4	Poor
Wang et al.[87]	Prospective cohort study	United States				DSM-IV	Lowest tertile	Moderate tertile/<br>Highest tertile	83.3 (2.8 SD) years	-	-	-	-	5	Poor
Werneck et al.[88]	Prospective cohort study	Austria, Belgium, Czech Republic, Denmark, France, Germany, Greece, Israel, Italy, Netherlands, Poland, Spain, Sweden, and Switzerland	19686	778	58.2%	Self-reported	No MVPA	MVPA > 1x/week	64.9	-	-	-	-	10.2	Moderate
Wilson et al.[89]	Prospective cohort study	United States	842	139		NINCDS-ADRDA	Lowest PA	Higher levels of PA (and/or cognitive activity)	~65+ years	X	-	-	-	4	Moderate
Wong et al.[90]	Prospective cohort study	United States	6155	874	55.4%	Cognitive test	None	PA defined as: walking or intentional exercise that elevated heart rate, such as working out, playing a sport, or walking	74.3	-	-	-	-	11	Poor

Author (year)	Design	Country of origin	N	cases	% women	Dementia criteria	Reference category	Exposure	Age at baseline	PA DOMAINS[1]				Follow-up (years)	Quality
										LTPA	OPA	HPA	CPA		
Wu et al.[91]	Prospective cohort study	China	1648	166	54.5%	DSM-IV	Low PA: <10.5 METs/week	PA: ≥10.5 METs/week	71.5	-	-	-	-	5.3	Poor
Wu et al.[92]	Prospective	Multiple	11988	800	57.9%	Mixed	0 MET-hrs/week	.1-9 MET-hrs/week; 9.1-18.0 MET-hrs/week; 18.1-36.0 MET-hrs/week; >36.0 MET-hrs/week	72.1	-	-	-	-	5	Moderate
Yoon et al.[18]	Retrospective cohort study	Korea	62286	3757	60.4%	Health records	0 MET/min/week	1-499 MET-min/week; 500-999 MET-min/week; ≥1000 MET-min/week; 1-299 MET-min/week LPA; ≥300 MET-min/week LPA	73.2	X	-	-	-	3.5	Moderate
Yoshitake et al.[93]	Prospective cohort study	Japan	828	103	59.5%	DSM-III-R	No daily LTPA or moderate/heavy PA at work	Daily leisure-time exercise or moderate to severe PA at work	74 years (range 65-98)	-	-	-	-	7	Poor
Zhu et al.[14]	Prospective	United Kingdom	500270	5185	54.4%	Health records	Lowest tertile	Medium and third tertile PA	56.53	X	X	X	X	10.7	Good
Zotcheva et al.[94]	Prospective cohort study	Norway	28916	359	50.2%	Clinical evaluation	MVPA (out of breath and break into sweat) less than once a week	MVPA (out of breath and break into sweat) at least once a week	43.4	-	-	-	-	25.2	Good
Zotcheva et al.[4]	Prospective cohort study	Norway	7005	902	49.8%	Health records	Stable low trajectory OPA	Increasing then decreasing trajectory OPA/ Stable intermediate trajectory OPA/ Stable high trajectory OPA	75.6/ 78.7/ 77.5	-	X	-	-		Moderate

X: Domain assessed; -: Domain not assessed.

[1] If no specific domain is marked (i.e., all columns are marked as '-'), the study assessed overall (non-domain-specific) physical activity.

References: Numbering matches the Appendix citation list.

CPA, commuting physical activity; DSM, Diagnostic and Statistical Manual of Mental Disorders; HPA, household physical activity; LTPA, leisure-time physical activity; MET, metabolic equivalent of task; MPA, moderate physical activity; MVPA, moderate-to-vigorous physical activity; NINCDS-ADRDA, National Institute of Neurological and Communicative Disorders and Stroke–Alzheimer's Disease and Related Disorders Association; OPA, occupational physical activity; PA, physical activity; SD, standard deviation; VPA, vigorous physical activity.

Study quality: High quality: Selection = 2.5–3 stars, Comparability = 1 star, Outcome = 2.5–3 stars; Moderate quality: Selection = 2–3 stars, Comparability = 0.5–1 star, Outcome = 2–3 stars; Low quality: studies not meeting criteria for moderate/high quality

Supplementary Table S2. Sensitivity analysis estimating the pooled relative risk for all-cause dementia, Alzheimer's disease, and vascular dementia associated with physical activity accounting for distinct covariates.

Sensitivity analysis	All-cause dementia			Alzheimer's disease			Vascular dementia		
	Cohorts/ Number of studies/ ES	RR (95% CI)	I^2	Cohorts/ Number of studies/ ES	RR (95% CI)	I^2	Cohorts/ Number of studies/ ES	RR (95% CI)	I^2
Covariates									
Age									
Adjusted	65/139	0.82 (0.76, 0.89)	97.2%	26/58	0.79 (0.69, 0.90)	91.5%	9/21	0.71 (0.57, 0.87)	77.5%
Not adjusted	7/7	0.59 (0.44, 0.80)	87.0%	1/1	0.94 (0.47, 1.77)	NA	0/0	NA	NA
Education									
Adjusted	60/132	0.82 (0.75, 0.89)	95.1%	23/54	0.81 (0.72, 0.90)	82.0%	7/19	0.69 (0.55, 0.87)	85.2%
Not adjusted	12/14	0.75 (0.65, 0.87)	99.1%	4/5	0.65 (0.30, 1.40)	96.6%	2/2	0.82 (0.49, 1.37)	0.0%
Chronic diseases									
Adjusted	45/103	0.81 (0.74, 0.89)	98.3%	18/48	0.74 (0.63, 0.87)	89.8%	7/17	0.73 (0.62, 0.86)	78.1%
Not adjusted	27/43	0.79 (0.71, 0.89)	79.4%	9/11	0.93 (0.82, 1.06)	40.1%	2/4	0.69 (0.28, 1.71)	89.9%
APOE ε4 status									
Adjusted	19/39	0.84 (0.75, 0.93)	84.2%	13/27	0.79 (0.69, 0.90)	80.2%	3/5	0.71 (0.45, 1.12)	73.1%
Not adjusted	53/110	0.78 (0.72, 0.86)	98.0%	14/32	0.79 (0.61, 1.02)	93.6%	6/16	0.70 (0.55, 0.89)	78.1%

ES: effect sizes; NA: not applicable due to insufficient studies.

Supplementary Table S3. Sensitivity analysis estimating the risk of all-cause and specific-cause dementia associated with physical activity using studies that used baseline age, education, chronic diseases, or APOE ε4 status as covariates.

Sensitivity analysis	All-cause dementia			Alzheimer's disease			Vascular dementia		
	Cohorts/ Number of studies/ ES	RR (95% CI)	I^2	Cohorts/ Number of studies/ ES	RR (95% CI)	I^2	Cohorts/ Number of studies/ ES	RR (95% CI)	I^2
Covariates									
Age									
Leisure	14/29	0.78 (0.66, 0.93)	90.9%	7/16	0.78 (0.60, 1.03)	91.3%	3/7	0.83 (0.66, 1.05)	63.2%
Occupation	5/13	1.20 (1.01, 1.42)	85.6%	2/3	1.17 (0.82, 1.65)	75.1%	1/2	0.95 (0.73, 1.24)	72.1%
Education									
Leisure	14/28	0.74 (0.61, 0.90)	92.9%	6/14	0.76 (0.56, 1.04)	93.1%	3/7	0.83 (0.66, 1.05)	63.2%
Occupation	5/13	1.20 (1.01, 1.42)	85.6%	2/3	1.17 (0.82, 1.65)	75.1%	1/2	0.95 (0.73, 1.24)	72.1%
Chronic diseases									
Leisure	11/20	0.76 (0.61, 0.93)	93.5%	6/13	0.77 (0.55, 1.06)	94.2%	2/4	0.74 (0.65, 0.85)	32.2%
Occupation	3/6	1.25 (0.92, 1.70)	95.1%	2/3	1.17 (0.82, 1.65)	75.1%	1/2	0.95 (0.73, 1.24)	72.1%
APOE ε4 status									
Leisure	2/5	0.86 (0.62, 1.20)	70.4%	2/4	0.57 (0.24, 1.34)	91.9%	1/2	0.95 (0.73, 1.24)	0%
Occupation	1/1	1.45 (0.66, 3.17)	NA	0/0	NA	NA	0/0	NA	NA

NA: not applicable due to insufficient studies.

Supplementary Table S4. Sensitivity analysis excluding studies rated as low quality.

	All-cause dementia			Alzheimer's disease			Vascular dementia		
	Cohorts/ Number of studies/ ES	RR (95% CI)	P value for moderation	Cohorts/ Number of studies/ ES	RR (95% CI)	P value for moderation	Cohorts/ Number of studies/ ES	RR (95% CI)	P value for moderation
Overall	39/100	0.84 (0.75, 0.95)		15/41	0.74 (0.63, 0.87)		4/13	0.68 (0.53, 0.88)	
Domains			<0.0001			NA*			NA*
Leisure PA	10/21	0.74 (0.59, 0.92)		4/10	0.67 (0.43, 1.04)		1/2	0.73 (0.63, 0.85)	
Occupational PA	5/13	1.20 (1.01, 1.42)		1/2	1.09 (0.98, 1.21)		1/2	0.95 (0.73, 1.24)	

*NA: not applicable due to insufficient studies.

Supplementary Table S5. Multivariate meta-regression of physical activity domains on dementia risk, adjusted for physical activity volume.

	All-cause dementia			Alzheimer's disease		
	RR	(95% CI)	z-value	RR	(95% CI)	z-value
Intercept	0.87	0.82, 0.92	-4.90	1.00*	1.00*, 1.00*	0.98
Leisure-time physical activity	1.08	0.99, 1.17	1.71	1.00*	1.00*, 1.00*	0.99
Occupational physical activity	1.30	1.16, 1.45	-0.66	1.00*	1.00*, 1.00*	0.93
Total Dose (MET-min/week)	1.00*	1.00*, 1.00*	4.70	1.00*	1.00*, 1.00*	0.96

Note: R^2 = 20.0% for all-cause dementia; R^2= 0.0% for Alzheimer's disease. Insufficient data were available to perform this analysis for vascular dementia. RR: Relative risk; CI: Confidence Interval. *Values rounded to two decimal places; actual estimate is 0.9999.

Supplementary Table S6. Summary of Findings Table (GRADE)

Outcome	Studies (k)	Participants (N) / Events	Certainty Assessment Factors	Certainty
**All-cause dementia**				
Leisure-time physical activity	23	596 677 / 14509	Downgrade for heterogeneity (I^2: 93%). Upgrade for Dose-Response Evidence.	Low ⊕⊕◯◯
Occupational physical activity	5	519 757 / 8259	Downgrade for heterogeneity (I^2: 86%). Downgrade for Imprecision (small k). Upgrade for Dose-Response Evidence.	Low ⊕⊕◯◯
Household physical activity	1	500 270 / 5185	Downgrade for Imprecision (Single study).	Very Low ⊕◯◯◯
Commuting physical activity	2	501 719 / 5302	Downgrade for Inconsistency and Imprecision (small *k*).	Very Low ⊕◯◯◯
**Alzheimer's disease**				
Leisure-time physical activity	7	528 566 / 4651	Downgrade for heterogeneity (I^2: 54%).	Very Low ⊕◯◯◯
Occupational physical activity	2	501 417 / 1594	Downgrade for heterogeneity (I^2: 54%) and Imprecision (small *k*).	Very Low ⊕◯◯◯
Household physical activity	1	500 270 / 1561	Downgrade for Imprecision (Single study).	Very Low ⊕◯◯◯
Commuting physical activity	2	501 417 / 1594	Downgrade for heterogeneity (I^2: 54%) and Imprecision (small *k*).	Very Low ⊕◯◯◯
**Vascular dementia**				
Leisure-time physical activity	3	521389 / 2075	Downgrade for heterogeneity (I^2: 54%).	Very Low ⊕◯◯◯
Occupational physical activity	1	500 270 / 1561	Downgrade for Imprecision (Single study).	Very Low ⊕◯◯◯
Household physical activity	1	500 270 / 1561	Downgrade for Imprecision (Single study).	Very Low ⊕◯◯◯
Commuting physical activity	1	500 270 / 1561	Downgrade for Imprecision (Single study).	Very Low ⊕◯◯◯

REFERENCES

1 Iso-Markku P, Kujala UM, Knittle K, Polet J, Vuoksimaa E, Waller K. Physical activity as a protective factor for dementia and Alzheimer's disease: systematic review, meta-analysis and quality assessment of cohort and case–control studies. *Br J Sports Med* 2022; : bjsports-2021-104981.

2 Viechtbauer W. Conducting Meta-Analyses in R with the metafor Package. *J Stat Softw* 2010; **36**: 1–48.

3 Stanley TD, Doucouliagos H. Meta-regression approximations to reduce publication selection bias. *Res Synth Methods* 2014; **5**: 60–78.

4 Zotcheva E, Bratsberg B, Strand BH, *et al.* Trajectories of occupational physical activity and risk of later-life mild cognitive impairment and dementia: the HUNT4 70+ study. *The Lancet Regional Health - Europe* 2023; **34**. DOI:10.1016/J.LANEPE.2023.100721/ATTACHMENT/782545C1-001B-4A5D-9CA2-D85E330DF7E8/MMC1.DOCX.

5 Jung W, Park SH, Kim S, *et al.* Predicting mid- and late-life dementia risk in primary care: A prognostic study from a national health screening cohort. *Psychiatry Res* 2024; **342**: 116237.

6 Nabe-Nielsen K, Holtermann A, Gyntelberg F, *et al.* The effect of occupational physical activity on dementia: Results from the Copenhagen Male Study. *Scand J Med Sci Sports* 2021; **31**: 446–55.

7 Christensen U, Krølner R, Nilsson CJ, *et al.* Addressing social inequality in aging by the Danish occupational social class measurement. *J Aging Health* 2014; **26**: 106–27.

8 Li JM, Gauen AM, Zmora R, *et al.* Midlife and late-life population attributable fractions of risk factors for dementia in the United States: The Dementia Risk Prediction Project. *Alzheimers Dement* 2026; **22**. DOI:10.1002/ALZ.71065.

9 Zhang J, Yu KF. What's the Relative Risk?: A Method of Correcting the Odds Ratio in Cohort Studies of Common Outcomes. *JAMA* 1998; **280**: 1690–1.

10 VanderWeele TJ. On a Square-Root Transformation of the Odds Ratio for a Common Outcome. *Epidemiology* 2017; **28**: E58–60.

11 Shor E, Roelfs D, Vang ZM. The "Hispanic mortality paradox" revisited: Meta-analysis and meta-regression of life-course differentials in Latin American and Caribbean immigrants' mortality. *Soc Sci Med* 2017; **186**: 20–33.

12 Herrmann SD, Willis EA, Ainsworth BE, *et al.* 2024 Adult Compendium of Physical Activities: A third update of the energy costs of human activities. *J Sport Health Sci* 2024; **13**: 6–12.

13 Iso-Markku P, Aaltonen S, Kujala UM, *et al.* Physical Activity and Cognitive Decline Among Older Adults: A Systematic Review and Meta-Analysis. *JAMA Netw Open* 2024; **7**: e2354285–e2354285.

14 Zhu J, Ge F, Zeng Y, *et al.* Physical and Mental Activity, Disease Susceptibility, and Risk of Dementia: A Prospective Cohort Study Based on UK Biobank. *Neurology* 2022; **99**: E799–813.

15 Hou C, Zhang Y, Zhao F, *et al.* Active Travel Mode and Incident Dementia and Brain Structure. *JAMA Netw Open* 2025; **8**: e2514316–e2514316.

16 Petermann-Rocha F, Lyall DM, Gray SR, *et al.* Dose-response association between device-measured physical activity and incident dementia: a prospective study from UK Biobank. *BMC Med* 2021; **19**: 1–13.

17 Park D, Bang M, Kim HS, Kim JH. Modifiable risk factors for early- and late-onset dementia using the Korean national health insurance service database. *J Prev Alzheimers Dis* 2025; **12**: 100032.
18 Yoon M, Yang PS, Jin MN, *et al.* Association of Physical Activity Level With Risk of Dementia in a Nationwide Cohort in Korea. *JAMA Netw Open* 2021; **4**. DOI:10.1001/JAMANETWORKOPEN.2021.38526.
19 Hwang PH, Ang TFA, De Anda-Duran I, *et al.* Examination of potentially modifiable dementia risk factors across the adult life course: The Framingham Heart Study. *Alzheimers Dement* 2023; **19**: 2975–83.
20 Marino FR, Lyu C, Li Y, Liu T, Au R, Hwang PH. Physical Activity Over the Adult Life Course and Risk of Dementia in the Framingham Heart Study. *JAMA Netw Open* 2025; **8**: e2544439–e2544439.
21 Giorgio K, Stephen JJ, Mansolf M, *et al.* Risk factors for early-onset and late-onset dementia: a prospective cohort study. *Lancet Healthy Longev* 2026; **7**. DOI:10.1016/j.lanhl.2026.100831.
22 Palta P, Sharrett AR, Deal JA, *et al.* Leisure-time physical activity sustained since midlife and preservation of cognitive function: The Atherosclerosis Risk in Communities Study. *Alzheimers Dement* 2019; **15**: 273–81.
23 Sato K, Kondo N, Hanazato M, Tsuji T, Kondo K. Potential causal effect of physical activity on reducing the risk of dementia: a 6-year cohort study from the Japan Gerontological Evaluation Study. *Int J Behav Nutr Phys Act* 2021; **18**. DOI:10.1186/S12966-021-01212-W.
24 Du Z, Sato K, Tsuji T, Kondo K, Kondo N. Sedentary behavior and the combination of physical activity associated with dementia, functional disability, and mortality: A cohort study of 90,471 older adults in Japan. *Prev Med (Baltim)* 2024; **180**: 107879.
25 Kanamori S, Kawaguchi K, Tsuji T, *et al.* Taiso practice and risk of functional disability and dementia among older adults in Japan: The JAGES cohort study. *SSM Popul Health* 2024; **28**: 101731.
26 Global Burden of Diseases. Estimation of the global prevalence of dementia in 2019 and forecasted prevalence in 2050: an analysis for the Global Burden of Disease Study 2019. *Lancet Public Health* 2022.
27 Van Kan GA, Rolland Y, Gillette-Guyonnet S, *et al.* Gait Speed, Body Composition, and Dementia. The EPIDOS-Toulouse Cohort. *The Journals of Gerontology: Series A* 2012; **67A**: 425–32.
28 Andel R, Crowe M, Pedersen NL, Fratiglioni L, Johansson B, Gatz M. Physical Exercise at Midlife and Risk of Dementia Three Decades Later: A Population-Based Study of Swedish Twins. *The Journals of Gerontology: Series A* 2008; **63**: 62–6.
29 Annweiler C, Rolland Y, Schott AM, *et al.* Higher Vitamin D Dietary Intake Is Associated With Lower Risk of Alzheimer's Disease: A 7-Year Follow-up. *The Journals of Gerontology: Series A* 2012; **67**: 1205–11.
30 Beauchet O, Sekhon H, Cooper-Brown L, *et al.* Motoric cognitive risk syndrome and incident dementia in older adults from the Québec NuAge cohort. *Age Ageing* 2021; **50**: 969–73.
31 Blasko I, Jungwirth S, Kemmler G, Weissgram S, Tragl KH, Fischer P. Leisure time activities and cognitive functioning in middle European population-based study. *Eur Geriatr Med* 2014; **5**: 200–7.

32 Boo YY, Jutila OE, Cupp MA, Manikam L, Cho S Il. The identification of established modifiable mid-life risk factors for cardiovascular disease which contribute to cognitive decline: Korean Longitudinal Study of Aging (KLoSA). *Aging Clin Exp Res* 2021; **33**: 2573–86.
33 Carlson MC, Helms MJ, Steffens DC, Burke JR, Potter GG, Plassman BL. Midlife activity predicts risk of dementia in older male twin pairs. *Alzheimer's and Dementia* 2008; **4**: 324–31.
34 Chang M, Jonsson P V., Snaedal J, *et al.* The Effect of Midlife Physical Activity on Cognitive Function Among Older Adults: AGES—Reykjavik Study. *The Journals of Gerontology: Series A* 2010; **65A**: 1369–74.
35 Chen S, Chen S, Hanewald K, *et al.* Social Environment, Lifestyle, and Genetic Predisposition With Dementia Risk: A Long-Term Longitudinal Study Among Older Adults. *The Journals of Gerontology: Series A* 2024; **79**: 128.
36 De Bruijn RFAG, Schrijvers EMC, De Groot KA, *et al.* The association between physical activity and dementia in an elderly population: the Rotterdam Study. *European Journal of Epidemiology 2013 28:3* 2013; **28**: 277–83.
37 Deckers K, Köhler S, van Boxtel M, Verhey F, Brayne C, Fleming J. Lack of associations between modifiable risk factors and dementia in the very old: findings from the Cambridge City over-75s cohort study. *Aging Ment Health* 2018; **22**: 1272–8.
38 Doi T, Makino K, Tomida K, *et al.* Life-Space Activities and Incident Dementia Among Older Adults: Insights From a Cohort Study. *J Am Med Dir Assoc* 2025; **26**. DOI:10.1016/j.jamda.2024.105416.
39 Dupré C, Bongue B, Helmer C, *et al.* Physical activity types and risk of dementia in community-dwelling older people: The Three-City cohort. *BMC Geriatr* 2020; **20**: 1–9.
40 Elwood P, Galante J, Pickering J, *et al.* Healthy Lifestyles Reduce the Incidence of Chronic Diseases and Dementia: Evidence from the Caerphilly Cohort Study. *PLoS One* 2013; **8**: e81877.
41 Feter N, Mielke GI, Leite JS, Brown WJ, Coombes JS, Rombaldi AJ. Physical activity in later life and risk of dementia: Findings from a population-based cohort study. *Exp Gerontol* 2021; **143**: 111145.
42 Gelber RP, Petrovitch H, Masaki KH, *et al.* Lifestyle and the risk of dementia in Japanese-American men. *J Am Geriatr Soc* 2012; **60**: 118–23.
43 Grasset L, Joly P, Jacqmin-Gadda H, *et al.* Real benefit of a protective factor against dementia: Importance of controlling for death. Example of sport practice. *PLoS One* 2017; **12**: e0174950.
44 Gross AL, Lu H, Meoni L, Gallo JJ, Schrack JA, Sharrett AR. Physical Activity in Midlife is not Associated with Cognitive Health in Later Life Among Cognitively Normal Older Adults. *J Alzheimers Dis* 2017; **59**: 1349–58.
45 Gureje O, Ogunniyi A, Kola L, Abiona T. Incidence of and risk factors for dementia in the Ibadan study of aging. *J Am Geriatr Soc* 2011; **59**: 869–74.
46 Hansson O, Svensson M, Gustavsson AM, *et al.* Midlife physical activity is associated with lower incidence of vascular dementia but not Alzheimer's disease. *Alzheimer's Research & Therapy 2019 11:1* 2019; **11**: 87-.
47 Hessler JB, Ander KH, Brönner M, *et al.* Predicting dementia in primary care patients with a cardiovascular health metric: a prospective population-based study. *BMC Neurology 2016 16:1* 2016; **16**: 116-.

48 Kim JM, Stewart R, Bae KY, *et al.* Role of BDNF val66met polymorphism on the association between physical activity and incident dementia. *Neurobiol Aging* 2011; **32**: 551.e5-551.e12.

49 Kivimäki M, Singh-Manoux A, Pentti J, *et al.* Physical inactivity, cardiometabolic disease, and risk of dementia: An individual-participant meta-analysis. *BMJ (Online)* 2019; **365**. DOI:10.1136/bmj.l1495.

50 Kunutsor SK, Laukkanen JA, Kauhanen J, Willeit P. Physical activity may not be associated with long-term risk of dementia and Alzheimer's disease. *Eur J Clin Invest* 2021; **51**. DOI:10.1111/ECI.13415.

51 Larson EB, Wang L, Bowen JD, *et al.* Exercise Is Associated with Reduced Risk for Incident Dementia among Persons 65 Years of Age and Older. *https://doi.org/107326/0003-4819-144-2-200601170-00004* 2006; **144**: 73–81.

52 Laurin D, Verreault R, Lindsay J, MacPherson K, Rockwood K. Physical activity and risk of cognitive impairment and dementia in elderly persons. *Arch Neurol* 2001; **58**: 498–504.

53 Lee ATC, Richards M, Chan WC, Chiu HFK, Lee RSY, Lam LCW. Intensity and Types of Physical Exercise in Relation to Dementia Risk Reduction in Community-Living Older Adults. *J Am Med Dir Assoc* 2015; **16**: 899.e1-899.e7.

54 Li P, Yu L, Lim ASP, *et al.* Fractal regulation and incident Alzheimer's disease in elderly individuals. *Alzheimer's and Dementia* 2018; **14**: 1114–25.

55 Lin S, Yang Y, Qi Q, *et al.* The beneficial effect of physical exercise on cognitive function in a non-dementia aging Chinese population. *Front Aging Neurosci* 2019; **11**: 477181.

56 Lipnicki DM, Crawford J, Kochan NA, *et al.* Risk Factors for Mild Cognitive Impairment, Dementia and Mortality: The Sydney Memory and Ageing Study. *J Am Med Dir Assoc* 2017; **18**: 388–95.

57 Llamas-Velasco S, Contador I, Villarejo-Galende A, Lora-Pablos D, Bermejo-Pareja F. Physical Activity as Protective Factor against Dementia: A Prospective Population-Based Study (NEDICES). *Journal of the International Neuropsychological Society* 2015; **21**: 861–7.

58 Luck T, Riedel-Heller SG, Luppa M, *et al.* Apolipoprotein E epsilon 4 genotype and a physically active lifestyle in late life: analysis of gene-environment interaction for the risk of dementia and Alzheimer's disease dementia. *Psychol Med* 2014; **44**: 1319–29.

59 Nabe-Nielsen K, Garde AH, Gyntelberg F, *et al.* Effects of Occupational and Leisure-Time Physical Activity on the Risk of Dementia: Results From the Copenhagen City Heart Study. *Scand J Med Sci Sports* 2024; **34**: e14730.

60 Nagata K, Tsunoda K, Fujii Y, Tsuji T, Okura T. Physical Activity Intensity and Suspected Dementia in Older Japanese Adults: A Dose-Response Analysis Based on an 8-Year Longitudinal Study. *J Alzheimers Dis* 2022; **87**: 1055–64.

61 Najar J, Östling S, Gudmundsson P, *et al.* Cognitive and physical activity and dementia: A 44-year longitudinal population study of women. *Neurology* 2019; **92**: E1322–30.

62 Ogino E, Manly JJ, Schupf N, Mayeux R, Gu Y. Current and past leisure time physical activity in relation to risk of Alzheimer's disease in older adults. *Alzheimer's and Dementia* 2019; **15**: 1603–11.

63 Öhlin J, Toots A, Littbrand H, *et al.* Physical Activity in Late Middle- to Older-Aged People and Dementia, Cognitive, and Physical Function Two Decades Later. *Dement Geriatr Cogn Disord* 2022; **51**: 135–41.

64 Paganini-Hill A, Kawas CH, Corrada MM. Lifestyle factors and dementia in the oldest-old: The 90 + study. *Alzheimer Dis Assoc Disord* 2016; **30**: 21–6.
65 Peeters G, Almirall Sanchez A, Llibre Guerra J, *et al.* Risk Factors for Incident Dementia Among Older Cubans. *Front Public Health* 2020; **8**: 481.
66 Podewils LJ, Guallar E, Kuller LH, *et al.* Physical Activity, APOE Genotype, and Dementia Risk: Findings from the Cardiovascular Health Cognition Study. *Am J Epidemiol* 2005; **161**: 639–51.
67 Ravaglia G, Forti P, Lucicesare A, *et al.* Physical activity and dementia risk in the elderly: findings from a prospective Italian study. *Neurology* 2008; **70**: 1786–94.
68 Rovio S, Kåreholt I, Helkala E-L, *et al.* Leisure-time physical activity at midlife and the risk of dementia and Alzheimer's disease. *Lancet Neurol* 2005; **4**: 705–11.
69 Rovio S, Kåreholt I, Viitanen M, *et al.* Work-related physical activity and the risk of dementia and Alzheimer's disease. *Int J Geriatr Psychiatry* 2007; **22**: 874–82.
70 Sabia S, Dugravot A, Dartigues JF, *et al.* Physical activity, cognitive decline, and risk of dementia: 28 year follow-up of Whitehall II cohort study. *BMJ* 2017; **357**. DOI:10.1136/BMJ.J2709.
71 Sattler C, Erickson KI, Toro P, Schröder J. Physical fitness as a protective factor for cognitive impairment in a prospective population-based study in germany. *Journal of Alzheimer's Disease* 2011; **26**: 709–18.
72 Scarmeas N, Luchsinger JA, Schupf N, *et al.* Physical Activity, Diet, and Risk of Alzheimer Disease. *JAMA* 2009; **302**: 627–37.
73 Shaaban CE, Jia Y, Chang CCH, Ganguli M. Independent and joint effects of vascular and cardiometabolic risk factor pairs for risk of all-cause dementia: a prospective population-based study. *Int Psychogeriatr* 2019; **31**: 1421–32.
74 Shih IF, Paul K, Haan M, Yu Y, Ritz B. Physical activity modifies the influence of apolipoprotein E ε4 allele and type 2 diabetes on dementia and cognitive impairment among older Mexican Americans. *Alzheimer's and Dementia* 2018; **14**: 1–9.
75 Simons LA, Simons J, McCallum J, Friedlander Y. Lifestyle factors and risk of dementia: Dubbo study of the elderly. *Medical Journal of Australia* 2006; **184**: 68–70.
76 Song Y, Yu Z, Lou S, Liu D. Association between the Frequency of Individual Outdoor Activity and Risk of Incident Dementia in an Elderly Population in China: A National Cohort Study. *Neuroepidemiology* 2025. DOI:10.1159/000548330.
77 Soni M, Orrell M, Bandelow S, *et al.* Physical activity pre- and post-dementia: English Longitudinal Study of Ageing. *Aging Ment Health* 2019; **23**: 15–21.
78 Sutin AR, Stephan Y, Robinson E, Daly M, Terracciano A. Perceived weight discrimination and risk of incident dementia. *International Journal of Obesity 2018 43:5* 2018; **43**: 1130–4.
79 Taaffe DR, Irie F, Masaki KH, *et al.* Physical Activity, Physical Function, and Incident Dementia in Elderly Men: The Honolulu–Asia Aging Study. *The Journals of Gerontology: Series A* 2008; **63**: 529–35.
80 Tan ZS, Spartano NL, Beiser AS, *et al.* Physical Activity, Brain Volume, and Dementia Risk: The Framingham Study. *The Journals of Gerontology: Series A* 2017; **72**: 789–95.
81 Tari AR, Selbæk G, Franklin BA, *et al.* Temporal changes in personal activity intelligence and the risk of incident dementia and dementia related mortality: A prospective cohort study (HUNT). *EClinicalMedicine* 2022; **52**. DOI:10.1016/j.eclinm.2022.101607.

82 Tolppanen AM, Solomon A, Kulmala J, *et al.* Leisure-time physical activity from mid- to late life, body mass index, and risk of dementia. *Alzheimer's and Dementia* 2015; **11**: 434-443.e6.

83 Vaillant GE, Okereke OI, Mukamal K, Waldinger RJ. Antecedents of intact cognition and dementia at age 90 years: A prospective study. *Int J Geriatr Psychiatry* 2014; **29**: 1278–85.

84 Verdelho A, Madureira S, Ferro JM, *et al.* Physical activity prevents progression for cognitive impairment and vascular dementia: Results from the LADIS (Leukoaraiosis and Disability) study. *Stroke* 2012; **43**: 3331–5.

85 Verghese J, Lipton RB, Katz MJ, *et al.* Leisure activities and the risk of dementia in the elderly. *NEW ENGLAND JOURNAL OF MEDICINE* 2003; **348**: 2508–16.

86 Wang L, Van Belle G, Kukull WB, Larson EB. Predictors of functional change: A longitudinal study of nondemented people aged 65 and older. *J Am Geriatr Soc* 2002; **50**: 1525–34.

87 Wang S, Luo X, Barnes D, Sano M, Yaffe K. Physical activity and risk of cognitive impairment among oldest-old women. *American Journal of Geriatric Psychiatry* 2014; **22**: 1149–57.

88 Werneck AO, Araujo RHO, Silva DR, Vancampfort D. Handgrip strength, physical activity and incident mild cognitive impairment and dementia. *Maturitas* 2023; **176**. DOI:10.1016/J.MATURITAS.2023.107789.

89 Wilson RS, Bennett DA, Bienias JL, *et al.* Cognitive activity and incident AD in a population-based sample of older persons. *Neurology* 2002; **59**: 1910–4.

90 Wong R, Mikhailova T, Hudson D, Park S, Guo S. Longitudinal Engagement in Modifiable Lifestyle Behaviors and Racial-Ethnic Differences in Dementia Risk. *J Aging Health* 2025; **37**: 22S.

91 Wu W, Ding D, Zhao Q, *et al.* Medium-to-High Late-Life Physical Activity Is Associated with Lower Risk of Incident Dementia: The Shanghai Aging Study. *Journal of Alzheimer's Disease* 2020; **73**: 751–8.

92 Wu W, Ding D, Zhao Q, *et al.* Dose-response relationship between late-life physical activity and incident dementia: A pooled analysis of 10 cohort studies of memory in an international consortium. *Alzheimers Dement* 2023; **19**: 107–22.

93 Yoshitake T, Kiyohara Y, Kato I, *et al.* Incidence and risk factors of vascular dementia and Alzheimer's disease in a defined elderly Japanese population. *Neurology* 1995; **45**: 1161–8.

94 Zotcheva E, Bergh S, Selbaek G, *et al.* Midlife Physical Activity, Psychological Distress, and Dementia Risk: The HUNT Study. *J Alzheimers Dis* 2018; **66**: 825–33.
